# National, regional, and global levels and trends in neonatal mortality between 1990 and 2017, with scenario-based projections to 2030: a systematic analysis

**DOI:** 10.1016/S2214-109X(19)30163-9

**Published:** 2019-05-13

**Authors:** Lucia Hug, Monica Alexander, Danzhen You, Leontine Alkema, UN Inter-agency Group for Child Mortality Estimation

**Affiliations:** a Data and Analytics Section, UN Children's Fund, New York, NY, USA; b University of Toronto, Toronto, ON, Canada; c University of Massachusetts Amherst, Amherst, MA, USA

## Abstract

**Background:**

Reducing neonatal mortality is an essential part of the third Sustainable Development Goal (SDG), to end preventable child deaths. To achieve this aim will require an understanding of the levels of and trends in neonatal mortality. We therefore aimed to estimate the levels of and trends in neonatal mortality by use of a statistical model that can be used to assess progress in the SDG era. With these estimates of neonatal mortality between 1990 and 2017, we then aimed to assess how different targets for neonatal mortality could affect the burden of neonatal mortality from 2018 to 2030.

**Methods:**

In this systematic analysis, we used nationally-representative empirical data related to neonatal mortality, including data from vital registration systems, sample registration systems, and household surveys, to estimate country-specific neonatal mortality rates (NMR; the probability of dying during the first 28 days of life) for all countries between 1990 (or the earliest year of available data) and 2017. For our analysis, we used all publicly available data on neonatal mortality from databases compiled annually by the UN Inter-agency Group for Child Mortality Estimation, which were extracted on or before July 31, 2018, for data relating to the period between 1950 and 2017. All nationally representative data were assessed. We used a Bayesian hierarchical penalised B-splines regression model, which allowed for data from different sources to be weighted differently, to account for variable biases and for the uncertainty in NMR to be assessed. The model simultaneously estimated a global association between NMR and under-5 mortality rate and country-specific and time-specific effects, which enabled us to identify countries with an NMR that was higher or lower than expected. Scenario-based projections were made at the county level by use of current levels of and trends in neonatal mortality and historic or annual rates of reduction that would be required to achieve national targets. The main outcome that we assessed was the levels of and trends in neonatal mortality and the global and regional NMRs from 1990 to 2017.

**Findings:**

Between 1990 and 2017, the global NMR decreased by 51% (90% uncertainty interval [UI] 46–54), from 36·6 deaths per 1000 livebirths (35·5–37·8) in 1990, to 18·0 deaths per 1000 livebirths (17·0–19·9) in 2017. The estimated number of neonatal deaths during the same period decreased from 5·0 million (4·9 million–5·2 million) to 2·5 million (2·4 million–2·8 million). Annual NMRs vary widely across the world, but west and central Africa and south Asia had the highest NMRs in 2017. All regions have reported reductions in NMRs since 1990, and most regions accelerated progress in reducing neonatal mortality in 2000–17 versus 1990–2000. Between 2018 and 2030, we project that 27·8 million children will die in their first month of life if each country maintains its current rate of reduction in NMR. If each country achieves the SDG neonatal mortality target of 12 deaths per 1000 livebirths or fewer by 2030, we project 22·7 million cumulative neonatal deaths by 2030. More than 60 countries need to accelerate their progress to reach the neonatal mortality SDG target by 2030.

**Interpretation:**

Although substantial progress has been made in reducing neonatal mortality since 1990, increased efforts to improve progress are still needed to achieve the SDG target by 2030. Accelerated improvements are most needed in the regions and countries with high NMR, particularly in sub-Saharan Africa and south Asia.

**Funding:**

Bill & Melinda Gates Foundation, United States Agency for International Development.

## Introduction

Improving neonatal mortality (ie, within the first 28 days of life) is an essential part of reducing under-5 mortality. To further advance child survival goals among newborns, the UN Secretary-General Ban Ki-moon launched the Global Strategy for Women's and Children's Health and Every Woman Every Child^1^ movement in 2010, the A Promise Renewed^2^ commitment to child survival in 2012, and the Every Newborn Action Plan^3^ in 2014. The A Promise Renewed and Every Newborn Action Plan initiatives set specific targets to reduce under-5 and neonatal mortality, and these targets were reflected in the Sustainable Development Goals (SDGs), which call for ending preventable deaths of newborn babies and children younger than 5 years by 2030. The SDGs specify that all countries should aim to reduce the neonatal mortality rate (NMR) to 12 deaths per 1000 livebirths or fewer and under-5 mortality to 25 deaths per 1000 livebirths or fewer in 2030.

**Research in context**

**Evidence before this study**

We used all publicly available data on under-5 and neonatal mortality from the UN Inter-agency Group for Child Mortality Estimation (IGME), which are compiled into databases annually. This database is maintained to enable timely estimates of mortality, with the goal of improving monitoring of progress in reducing child mortality. Nationally representative vital registration data on neonatal mortality are available for about 60 countries. For the remaining countries, data on neonatal mortality is collected via household surveys, vital registration, sample vital registration systems, or a combination of these. We collected all available data on Aug 1, 2018, on which date the UN IGME global database contained about 5700 observations spanning from July 1, 1950 (or earlier), to July 1, 2017. All nationally representative data were included in the database. Previous estimates of neonatal mortality rates from this database had relatively large uncertainty intervals and the models did not account for data-driven trends in neonatal mortality over time.

**Added value of this study**

Our study extended the data in the existing UN IGME global database by including new and updated observations and it incorporated updated HIV/AIDS estimates from UNAIDS and revised population numbers from the UN Population Division. To our knowledge, our findings represent the first comprehensive analysis of estimates of neonatal mortality rates up to 2017, and the first scenario-based projections from 2018 to 2030. We obtained estimates of neonatal mortality rates for 195 countries between 1990 (or earlier) and 2017, by use of a Bayesian hierarchical penalised B-splines regression model, which allowed for data from different sources to be weighted differently and for the uncertainty in these data to be assessed. Estimates of the mortality rates and associated indicators, such as annual rates of change, aggregated regional outcomes, and uncertainty intervals, help to assess the levels of and trends in neonatal mortality and to monitor progress in child survival. Our estimation method improves on previous methods by incorporation of data-driven changes in the empirical data on neonatal mortality over time as an input in the model, accounting for sampling errors, and producing more realistic uncertainty intervals. Our model allows identification of countries and regions with outlying patterns of neonatal mortality.

**Implications of all the available evidence**

Although substantial progress has been made in reducing neonatal mortality since 1990, increased efforts to improve progress are still needed to achieve the Sustainable Development Goal (SDG) target. Without any acceleration in the pace of reduction of neonatal mortality, we project that 1·8 million neonates (ie, those in the first 28 days of life) will die in 2030. However, if each country achieved the SDG target by 2030, there would be about 1·2 million neonatal deaths in 2030. However, achieving this goal requires increased efforts to enable continued improvements in child survival, especially in the high-burden regions of south Asia and sub-Saharan Africa.

The UN Inter-agency Group for Child Mortality Estimation (IGME) has estimated the NMR for 195 countries since 2011, producing trend estimates from 1990 to 2017. Before 2015, a generalised linear model was used to estimate NMR, by use of UN IGME under-5 mortality rate (U5MR) estimates as a predictor.^4^ The model differed on the basis of data available; for countries and regions with survey data, the relationship between NMR and U5MR was modelled, with country-specific intercept parameters. For countries with high-quality vital registration, random-effects parameters for slope or trend parameters in a country were simultaneously added. Although the previous model worked well in estimating overall trends, the model had the limitation that it did not capture empirical data-driven country-specific trends in countries without vital registration systems because the estimates were driven by the U5MR estimates in the model, and hence the previous model could not capture country-specific trends in the NMR that diverged from the changes expected in the U5MR.

Since 2015, a Bayesian model for estimating NMR for all countries from 1990 (or earlier) onwards has been used by the UN IGME. This method is similar to that used by the UN IGME to estimate both U5MR and sex-specific child mortality.^5^, ^6^ This model has the advantage that, compared with the previous model used by the UN IGME,^4^ it can capture empirical data trends in NMRs, both within countries and over time for all countries with appropriate data. Often NMR is highest in locations where high-quality vital registration data are absent, thus a model to assess trends in neonatal mortality in the SDG era was developed, to better capture trends in empirical data and to avoid treating datapoints from different sources equally. The model also makes use of the entire data series for each survey and does not only use the most recent datapoint.

We aimed to estimate the current state of and trends in neonatal mortality at global, regional, and country levels since 1990 or earlier. We also aimed to develop projections of NMR and the associated numbers of deaths from 2018 to 2030 under various scenarios to provide insight into the burden of neonatal deaths after 2017.

## Methods

### Overview

The UN IGME was established in 2004, to report on progress towards child survival goals, to improve methods for estimating child mortality, and to enhance country capacity to produce timely and properly assessed estimates of child mortality. The UN IGME is led by the UN International Children's Emergency Fund (UNICEF), and it includes members from WHO, the World Bank Group, and the Population Division of the UN Department of Economic and Social Affairs as full members. The UN IGME Technical Advisory Group, which consists of leading academic scholars and independent experts in demography and biostatistics, provides guidance on estimation methods, technical issues, and strategies for data analysis and data quality assessment.

The UN IGME updates its child mortality estimates annually after reviewing newly available data and assessing its quality and following consultations with Member States. These estimates are widely used in UNICEF's flagship publications, the UN Secretary-General's SDG report, and publications by other UN agencies, governments, and donors.

### Data sources

The UN IGME maintains a publicly-available dataset that contains nationally-representative empirical data relevant to neonatal mortality, including data from vital registration systems, sample registration systems, and household-based surveys. The full database used in our analysis is available on the UN IGME web portal. The neonatal mortality database contains datapoints from more than 5700 country-years, including 651 datasets and data in 190 countries between 1990 (or earlier) and 2017 (appendix pp 23–39). Most survey data came from Demographic and Health Surveys or variants thereof (eg, national Demographic and Health Surveys or World Fertility Surveys). Multiple Indicator Cluster Surveys were another source of survey data. A detailed description of data sources is available elsewhere.^7^

Our study focused on modelling the ratio of NMR to mortality from ages 1 month to 5 years (59 months), which we defined as NMR/(U5MR–NMR). This ratio is modelled on the logit scale, and data used was also on the logit scale. Estimates of mortality ratios from surveys and associated sampling errors were calculated for an optimised duration, according to the method developed by Pedersen and Liu.^8^ On the basis of a quality assessment of the survey and vital registration datasets, we included or excluded data from the model. LH, MA, and DY were involved in data extraction. Inclusion and exclusion of data was reviewed and agreed upon by the UN IGME. Annual vital registration data were included unless the coefficient of variation (the ratio of the standard error to the value of the observation) was greater than 10%, in which cases the ratio was calculated for longer periods by combining the observed neonatal deaths with deaths in the previous observation years and dividing by the sum of the births in the combined years. For countries affected by HIV/AIDS, the observed ratio of NMR to other child mortality was adjusted to account for the under-reporting associated with high maternal mortality in these countries.^9^ In the NMR model, country-year specific U5MR estimates were used as explanatory variables and to obtain final estimates. These estimates of U5MR were obtained from the UN IGME 2018 Round of estimation.^10^

### Data analysis

We estimated the NMR for each country during 1990–2017, or earlier if data were available, by use of a Bayesian hierarchical model.^11^ For our analysis, we used all publicly available data on neonatal mortality, which were extracted on or before Aug 1, 2018, for data relating to the period between July 1, 1950, and July 1, 2017. We estimated the ratio of NMR to other child mortality (ie, [U5MR–NMR]) and obtained estimates of NMR by recombining the ratios with U5MR estimates. The technical details on the model specification, implementation, and validation are available elsewhere.^11^

The ratio was modelled as the product of two components: the expected ratio and a country-year-specific multiplier. The expected ratio provides the expected association between the ratio and the current U5MR in a particular country and year. We explored parametric and non-parametric forms for the global association between the expected ratio and U5MR, and we found that the association between the log (ratio) and log (U5MR) is well captured by a linear function with a changing slope, suggesting a constant ratio at low U5MR and a decreasing ratio as the U5MR increases.

The second component, the country-year-specific multiplier, allows countries to have ratios that are higher or lower than expected given the current U5MR, compared with the global association. The country-specific multipliers were modelled with penalised splines regression, and they can be interpreted as a country-specific intercept plus fluctuations over time.^12^ The splines regression intercepts, and smoothing parameters were modelled hierarchically, and the fluctuations were penalised to ensure smooth trajectories over time. The inclusion of this country-year-specific multiplier in the model allows for data-driven changes in the NMR across countries and over time within countries. This component also allows for the identification of outlying countries—ie, those with relatively large (or small) higher-than-expected (or lower than expected) ratios given the current U5MR, compared with the global association.

NMR estimates were based on data from many sources. As part of the modelling process, a data model was used to capture and account for uncertainty in observations. The data model incorporated stochastic error for vital registration data (to capture uncertainty in the outcomes of random events) and sampling error and non-sampling error (eg, misreporting of age and sex and survivor selection bias) for survey data from different sources. Inclusion of these types of uncertainty in the data model allowed for the downweighting of observations that are less informative of the true ratio, compared with more informative observations.

We used a Markov chain Monte Carlo algorithm, implemented with JAGS software version 4.3.0^13^ within R version 3.3.3, to generate samples from the posterior distributions of the NMR ratios, and combined these samples with posterior samples of the U5MR to obtain samples of the NMR. The result was a set of trajectories of NMR over time for each country. The best estimate was taken to be the median of these trajectories, and 90% uncertainty intervals were computed by use of the 5th and 95th percentiles of the samples from the posterior distributions. Estimates and associated uncertainty of neonatal deaths for each country over time were obtained by combining the trajectories of NMR with livebirths. Adjustments to crisis-affected country-years were made after estimation.

We used the global association between the ratio of neonatal mortality to under-5 mortality and the U5MR under-5 mortality to calculate the expected NMR. We defined countries as outlying if the absolute difference between the point estimates of the estimated and the expected NMR was larger than one per 1000 livebirths, and if the lower (or upper) bound of the 90% uncertainty interval for the estimated NMR was at least 10% higher (or lower) than the expected NMR—ie, if the posterior probability that the ratio of the estimated NMR to the expected NMR was at least 1·1 (or, at most, 0·9) was more than 95%. Identifying outliers allows us to pinpoint countries with unusually low or high neonatal mortality, given their overall under-5 mortality (appendix pp 3, 4).

### Scenario-based projections from 2018 to 2030

We projected NMRs and the number of neonatal deaths under five scenarios from 2018 to 2030. Neonatal mortality was projected based on either a constant NMR or a decreasing NMR using an annual rate of reduction (ARR) defined as ARR=log(NMRt_2_/NMRt_1_)/(t_1_ – t_2_), where t_1_ and t_2_ refer to different years with t_1_<t_2_.

In the first scenario, we estimated neonatal mortality if the NMR were to remain at 2017 rates in all countries. In the second scenario, we assumed that the ARR in each country from 2000 to 2017 would continue from 2018 to 2030. If the ARR from 2000 to 2017 was negative, we kept the country's NMR constant and, for all countries, we constrained that the ratio of NMR/U5MR to ensure that it did not exceed the highest ratios in a country with good vital registration data (Finland, 0·78) during the projection period. In the third scenario, we projected the ARR in each country if it were equal to the ARR from the best performing country in the region—ie, the country with the highest ARR in 2000–17. In the fourth scenario, we calculated the necessary ARR for each country to achieve the SDG target of an annual NMR of 12 deaths per 1000 livebirths or fewer by 2030. Finally, for the fifth scenario, we calculated the necessary ARR for each country to achieve the current average NMR in the group of high-income countries of three deaths per 1000 livebirths by 2030.^10^ For countries that had already reached or would reach the targets in scenarios 4, 5, or both, projections from scenario 2 were used. In all scenarios, if a country reached the current lowest NMR observed among countries with more than 10 000 livebirths (namely, 0·9 deaths per 1000 livebirths annually in Japan), its NMR remained at that level for the rest of the projected period.

We calculated the number of neonatal deaths in each scenario from the medium variant population projections from the UN Population Division.^14^ We did not consider other effects, including changing mortality rates, on population numbers. Thus, the scenarios do not indicate the real number of lives to be saved, but they illustrate the potential number of lives that could be saved by reductions in neonatal mortality while maintaining the median number of births reported to the UN Population Division. Our projections for neonatal mortality reductions are based on past trends, but they do not consider how improvement in intervention coverage and quality of care could advance newborn survival.

### Role of the funding source

The funders of the study had no role in study design, data collection, data analysis, data interpretation, or writing of the report. The corresponding author had full access to all data in the study and had final responsibility for the decision to submit for publication.

## Results

**Figure 1:**
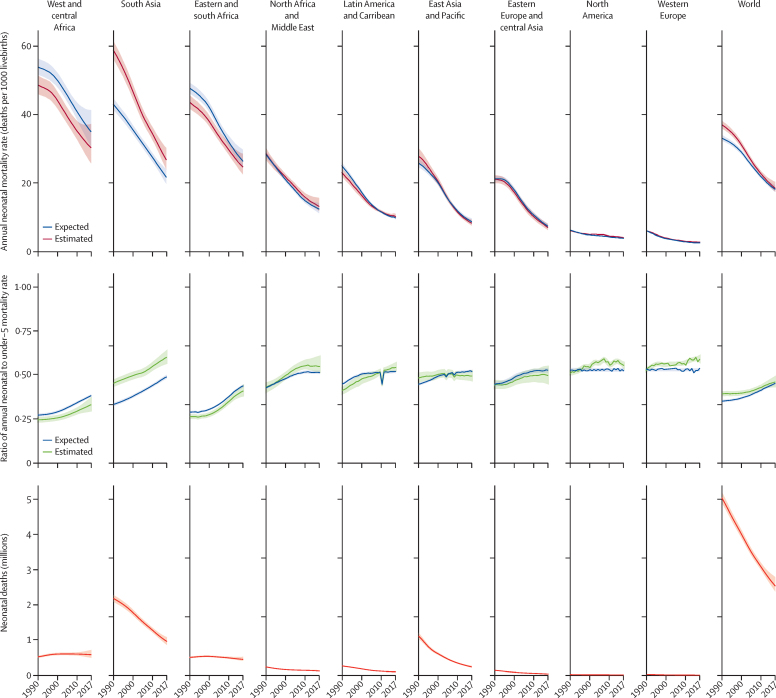
Neonatal mortality rate and deaths, including relative to under-5 mortality The expected annual neonatal mortality rate represents the annual neonatal mortality rate based on the annual under-5 mortality rate and the observed global association between the ratio of neonatal and under-5 mortality.

Between 1990 and 2017, the global annual NMR decreased by 51% (90% uncertainty interval 46–54), from 36·6 deaths per 1000 livebirths (35·5–37·8) to 18·0 deaths per 1000 livebirths (17·0–19·9; figure 1; table). The estimated number of neonatal deaths was reduced from 5·0 million deaths (4·9 million–5·2 million) in 1990 to 2·5 million deaths (2·4 million–2·8 million) in 2017. During this period, the global annual U5MR decreased by 58% (55–60), from 93·2 deaths per 1000 livebirths (92·0–94·7) to 39·1 deaths per 1000 livebirths (37·3–42·3).^10^

**Table:** Neonatal mortality rate and number of neonatal deaths, globally and by region and income group, in 1990, 2000, and 2017

		**Neonatal mortality rate**				**Neonatal deaths**				
		1990	2000	2017	Percentage decrease between 1990 and 2017, %	1990	2000	2017	Percentage decrease between 1990 and 2017, %	Proportion of total deaths worldwide in 2017, %
**By region**										
World (all regions)		36·6 (35·5 to 37·8)	30·6 (29·8 to 31·6)	18·0 (17·0 to 19·9)	51% (46 to 54)	5038 (4891 to 5202)	3997 (3891 to 4115)	2533 (2381 to 2789)	50% (45 to 53)	100% (100 to 100)
Sub-Saharan Africa		45·7 (44·0 to 47·6)	40·7 (39·2 to 42·4)	27·2 (24·7 to 31·6)	40·0% (31 to 46)	1033 (995 to 1075)	1141 (1100 to 1188)	1038 (940 to 1205)	−1% (−17 to 9)	41% (35 to 42)
	East and south Africa	43·2 (41·4 to 45·2)	37·5 (36·1 to 39·2)	24·2 (22·1 to 28·1)	44·0% (35 to 49)	509 (488 to 533)	538 (517 to 562)	453 (413 to 525)	11% (−3 to 20)	18% (16 to 18)
	West and central Africa	48·6 (46·0 to 51·3)	44·0 (41·7 to 46·5)	30·2 (25·7 to 37·2)	38·0% (23 to 47)	524 (496 to 554)	603 (572 to 638)	586 (498 to 721)	−12% (−38 to 5)	23% (20 to 23)
North Africa and Middle East		28·2 (26·3 to 29·8)	21·2 (20·3 to 22·1)	12·6 (11·3 to 15·1)	55·0% (45 to 60)	241 (225 to 256)	165 (158 to 173)	124 (111 to 148)	49% (37 to 55)	5% (4 to 5)
South Asia		58·6 (56·1 to 61·3)	46·6 (44·5 to 48·7)	26·9 (24·1 to 30·3)	54·0% (48 to 59)	2184 (2090 to 2282)	1773 (1695 to 1853)	961 (862 to 1082)	56% (50 to 61)	38% (34 to 40)
East Asia and Pacific		27·4 (25·4 to 29·8)	20·0 (19·0 to 21·1)	7·8 (7·1 to 8·8)	71·0% (67 to 75)	1112 (1032 to 1209)	611 (580 to 644)	241 (218 to 271)	78% (75 to 81)	10% (9 to 10)
Latin America and Caribbean		22·6 (21·4 to 23·8)	15·8 (14·9 to 16·7)	9·6 (9·2 to 10·4)	57·0% (53 to 60)	268 (254 to 283)	183 (173 to 194)	103 (97 to 110)	62% (58 to 64)	4% (4 to 4)
North America		5·6 (5·5 to 5·8)	4·5 (4·4 to 4·7)	3·6 (3·4 to 3·8)	36·0% (33 to 39)	24 (24 to 25)	20 (19 to 20)	16 (15 to 17)	34% (31 to 37)	1% (1 to 1)
Europe and central Asia		14·1 (13·4 to 14·8)	10·4 (9·9 to 10·9)	4·6 (4·2 to 5·2)	67·0% (63 to 70)	175 (167 to 184)	105 (100 to 111)	51 (46 to 57)	71% (67 to 74)	2% (2 to 2)
	Eastern Europe and central Asia	20·6 (19·6 to 21·9)	16·8 (15·9 to 17·8)	6·5 (5·8 to 7·5)	68·0% (63 to 72)	145 (137 to 154)	88 (83 to 94)	39 (35 to 45)	73% (68 to 76)	2% (1 to 1)
	Western Europe	5·5 (5·5 to 5·6)	3·5 (3·4 to 3·5)	2·3 (2·2 to 2·4)	58·0% (57 to 60)	30 (30 to 31)	17 (17 to 17)	11 (11 to 12)	62% (61 to 64)	<1% (<1 to <1)
**By income group**										
Low income		47·8 (46·1 to 49·7)	40·5 (39·2 to 42·2)	26·4 (24·2 to 30·4)	45% (36 to 50)	748 (722 to 778)	783 (757 to 816)	671 (615 to 772)	10% (−3 to 18)	27% (22 to 26)
Lower-middle income		48·8 (47·2 to 50·6)	40·0 (38·6 to 41·5)	23·9 (21·9 to 26·8)	51% (45 to 56)	2971 (2870 to 3081)	2498 (2409 to 2592)	1566 (1433 to 1753)	47% (41 to 52)	62% (56 to 65)
Upper-middle income		26·0 (24·3 to 28·1)	18·5 (17·7 to 19·5)	7·1 (6·6 to 7·7)	73% (69 to 75)	1221 (1139 to 1320)	655 (624 to 690)	255 (238 to 279)	79% (76 to 81)	10% (9 to 10)
High income		6·8 (6·5 to 7·1)	4·6 (4·4 to 4·7)	3·0 (3·0 to 3·5)	55% (48 to 57)	96 (92 to 101)	61 (59 to 63)	41 (40 to 48)	57% (51 to 59)	2% (2 to 2)

Data are presented with 90% uncertainty intervals. Annual neonatal mortality rate is shown as deaths per 1000 livebirths. Neonatal deaths are shown in thousands.

In 2017, the annual NMR was highest in west and central Africa, at 30·2 deaths per 1000 livebirths (90% uncertainty interval 25·7–37·2), and in south Asia, at 26·9 deaths per 1000 livebirths (24·1–30·3; figure 1; table). The annual NMR in these regions was more than 9 times higher than the average NMR in high-income countries, which was 3·0 deaths per 1000 livebirths (3·0–3·5). Together, south Asia and sub-Saharan Africa accounted for 79% of the total burden of neonatal deaths, and south Asia alone accounted for 38% of neonatal deaths (34–39), west and central Africa for 23% of neonatal deaths (20–23) and east and south Africa for 18% of neonatal deaths (16–18). At the country level, annual NMRs ranged from 44·2 to 0·9 deaths per 1000 livebirths (appendix p 52). Countries with the highest NMRs were concentrated in sub-Saharan Africa and south Asia. However, since 1990, most regions have made substantial progress in reducing neonatal mortality.

Globally, the ARR in NMR increased from 1·8% (90% uncertainty interval 1·5–2·1) in the 1990s to 3·1% (2·5–3·5) in 2000–17 (appendix p 53). At the country level, annual NMRs ranged from 44·2 to 0·9 deaths per 1000 livebirths; in 17 countries the annual NMR was more than 30 deaths per 1000 livebirths whereas, in 17 countries, the annual rate was less than 2 deaths per 1000 livebirths. Progress in reducing neonatal mortality between 1990 and 2017 was slower than progress in reducing mortality among children aged 1 to 59 months, particularly from 2000 to 2017, when the ARR in mortality among children aged 1 to 59 months (4·7, 90% uncertainty interval 4·1–5·1) was 1·5 times higher than that in neonatal mortality (3·1, 2·5–3·5). In 1990–2017, the ARR in the NMR was 2·6% (2·3–2·9), versus 3·7% (3·4–4·0) in the ARR of the mortality rate of children aged 1 to 59 months, a difference of 1·1% (0·7–1·5; appendix p 53). As a result, the global ratio of NMR to under-5 mortality increased by 0·07 (90% uncertainty interval 0·04–0·10), from 0·39 (0·38–0·40) in 1990 to 0·46 (0·43–0·49) in 2017 (figure 1; table).

At a regional level, the highest accelerations in ARR since 2000 have been noted in eastern Europe and central Asia, where the ARR has increased from 2·1% (90% uncertainty interval 1·3–2·8) in 1990–2000 to 5·6% (4·7–6·3) in 2000–17 (appendix p 53). In all regions, apart from east Asia and the Pacific, the ratio of NMR to U5MR has substantially increased since 1990, and differences across the regions have narrowed (table). In 2017, the ratio of NMR to U5MR was more than 0·5 in all regions, apart from those in sub-Saharan Africa, which still have the world's highest U5MRs. Among many low-income and lower middle-income countries with high under-5 mortality in 1990, decreases in under-5 mortality were faster than those in neonatal mortality, resulting in an increase in this ratio. This trend is reflected in the proportion of deaths in children younger than 5 years occurring in the neonatal period. In 2017, 47% of the global under-5 deaths (44–50) were in neonates, versus 40% of those (39–41) in 1990.

**Figure 2:**
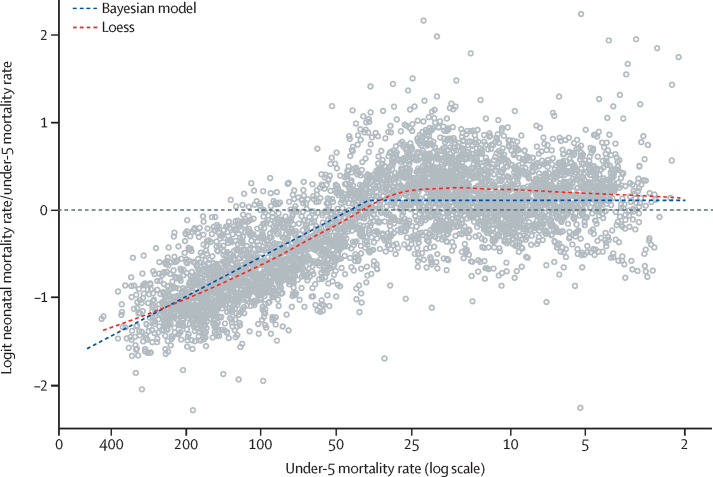
Estimated to expected ratios for a given under-5 mortality, based on the global association between the under-5 mortality rate and the ratio of neonatal mortality rate to under-5 mortality rate Loess= locally estimated scatterplot smoothing.

The estimated to expected ratios for a given under-5 mortality, based on the global association between U5MR and the ratio of NMR to U5MR, are shown in figure 2. The estimated global association from data from all country-years suggest that, with decreasing U5MR, the ratio of NMR to U5MR increases until the U5MR rate reaches a threshold: the piecewise-linear model suggests that the association between the logit ratio of NMR to U5MR is constant up to a U5MR of 37·2 deaths per 1000 livebirths annually (90% uncertainty interval 36·8–37·8). This threshold is equivalent to an NMR to U5MR ratio of 0·53 (0·52–0·54). Above this threshold, the estimated linear negative association indicates that a 1% increase in the log U5MR is associated with a 0·65% (0·62–0·69) decrease in the logit ratio.

The expected NMR estimates are based on U5MR and the observed global association between U5MR and the ratio of neonatal to under-5 mortality. A comparison of estimated with expected data thus allows us to pinpoint countries with NMRs that are higher or lower than expected when compared with the U5MR-implied outcomes. Globally, the ratio of the estimated NMR to expected NMR was significantly more than 1·12 (90% uncertainty interval 1·10–1·14) in 1990 but, in 2017, this ratio was no longer significantly different from 1, at 1·02 (0·97–1·08; figure 1; appendix pp 11–12). However, different patterns of estimated to expected NMR ratios applied to different countries and regions. In 1990, this ratio was 1·36 (1·33–1·40) times higher than expected in south Asia, and 1·08 (1·02–1·14) times higher than expected in east Asia and the Pacific, whereas this ratio was significantly lower than expected in east and south Africa, Latin America and the Caribbean, North America, and west and central Africa. By 2017, only south Asia had a high estimated NMR to expected NMR ratio, which was 1·23 (1·16–1·30) times higher than expected, and only west and central Africa had a ratio that was lower than expected, of 0·87 (0·78–0·96) times lower than expected (figure 1; appendix pp 11–12).

**Figure 3:**
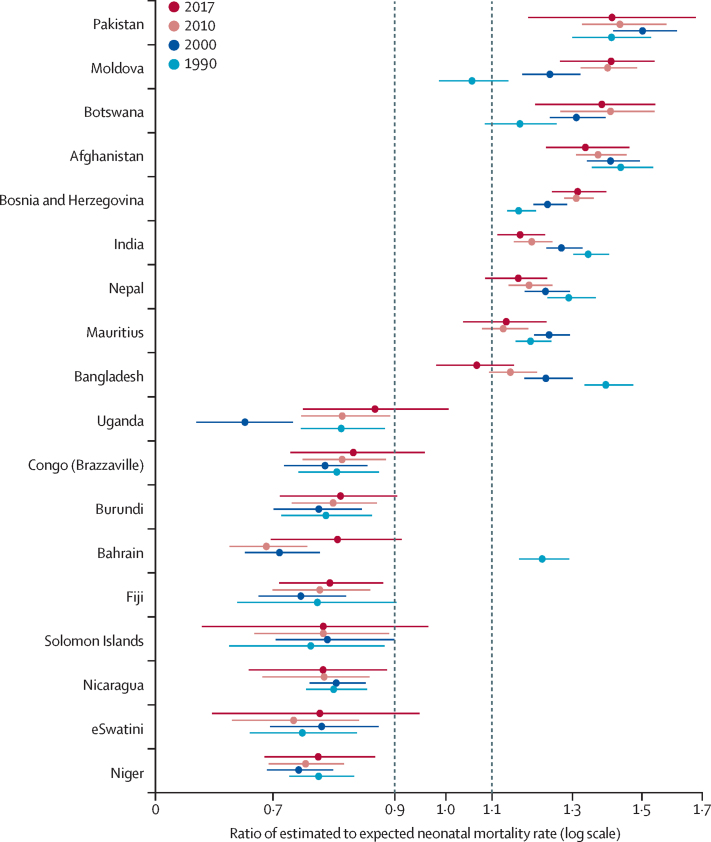
Patterns of ratios of estimated to expected neonatal mortality rates for countries with outlying ratios in 1990, 2000, and 2017 or 2000, 2010, and 2017 Error bars are 90% uncertainty intervals.

Patterns of estimated NMR to expected NMR ratios in south Asia were driven by country-specific outlying NMRs. Among the countries in south Asia (Afghanistan, Bangladesh, Bhutan, India, the Maldives, Nepal, Pakistan, and Sri Lanka), we found consistently and significantly higher ratios of NMR to U5MRs over the last 25 years in Afghanistan, India, and Pakistan (figure 3) and in Bangladesh and Nepal in 1990–2010. Overall, fewer countries showed higher or lower NMRs than expected in 2017 than in 1990. The number of countries with 10% higher-than-expected NMRs, which represented an absolute difference between the point estimates of the estimated annual NMR and the expected annual NMR of at least one death per 1000 livebirths and more than 10 000 livebirths in 2017, decreased from 14 countries to 12 countries between 1990 and 2017, whereas the number of countries with 10% lower-than-expected NMRs decreased from 25 countries to five countries.

**Figure 4:**
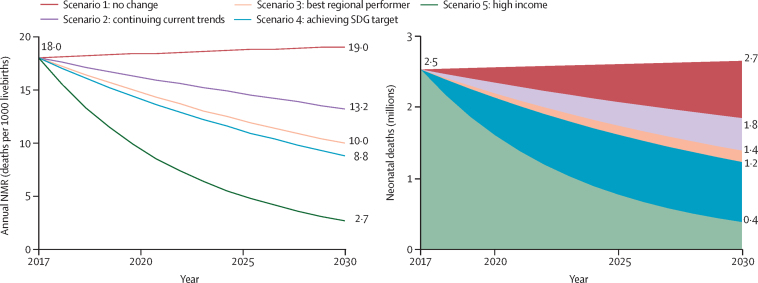
Projected NMR and number of neonatal deaths under different scenarios, from 2018 to 2030 Scenario 1 assumes a continuation of 2017 NMRs. Scenario 2 assumes a continuation of the 2000–17 annual rate of reduction in NMRs. Scenario 3 assumes an annual rate of reduction in NMR in each country that is equal to that of the country in the region that had the best annual rate of reduction in 2000–17. Scenario 4 assumes the necessary annual rate of reduction to achieve the Sustainable Development Goal target of 12 deaths per 1000 livebirths or fewer by 2030, across all countries. Scenario 5 assumes the necessary annual rate of reduction to achieve the 2017 average annual NMR across high-income countries—three deaths per 1000 livebirths—across all countries. NMR=neonatal mortality rate.

In our projection, we found that, if countries maintained their 2017 NMRs (scenario 1), the global annual NMR would increase to 19·0 deaths per 1000 livebirths and we would expect 2·7 million neonatal deaths in 2030 (figure 4; appendix pp 12–14). These numbers are higher than the 2017 estimates because of projected increases in the proportions of livebirths occurring in countries with high mortality. In this first scenario, during the entire period from 2018 to 2030, 37·6 million neonates would die. Under scenario 2, if each country maintained their current ARR, by 2030, the global annual NMR would decrease to 13·2 deaths per 1000 livebirths and an estimated 1·8 million neonates would die. The cumulative 27·8 million neonatal deaths occurring during 2018–30 would account for almost half of the projected 56 million deaths in children younger than 5 years under a similar scenario.^10^ In this scenario, the proportion of neonatal deaths in the overall under-5 deaths would increase from 47% in 2017 to 53% in 2030. Under scenario 3, assuming that each country would have the same ARR in neonatal mortality as the best performing country in the region, the annual NMR would decrease to 10·0 deaths per 1000 livebirths and 1·4 million children would die in 2030, with an estimated 24 million neonatal deaths between 2018 to 2030. By our estimates for scenario 4, which projects all countries achieving the SDG target (12 deaths per 1000 livebirths) by 2030, the global annual NMR would further decrease to 8·8 deaths per 1000 livebirths and 1·2 million neonates would die in 2030. Between 2018 and 2030, we estimated that the cumulative neonatal deaths under scenario 4 would be 22·7 million, about 5 million fewer than under scenario 2, which assumed that countries maintained their 2017 ARR. To achieve this SDG goal by 2030, more than 60 countries need to accelerate progress (appendix p 59), a higher number of countries than for the U5MR target (U5MR of 23 or lower; 51 countries).^10^ Among countries with more than 10 000 livebirths in 2017, 15 countries will not achieve the neonatal mortality SDG target at their estimated NMR under scenario 2, but these countries would achieve the under-5 mortality SDG target by 2030. 42 of more than 60 countries need to more than double their past reductions in NMR; the required ARR to meet the SDG target by 2030 is at least two times larger than the observed ARR in these countries (appendix p 61). Even more deaths could be averted if every country would at least reach the annual NMR of high-income countries (3 deaths per 1000 livebirths) by 2030, as we projected under scenario 5: the global NMR would further decrease to 2·7 deaths per 1000 livebirths and 0·4 million neonates would die in 2030. In this scenario, we would project 13·5 million cumulative deaths during 2018–30.

## Discussion

Despite substantial progress, 2·5 million neonates died in 2017 alone, and huge disparities in neonatal mortality persist across regions and countries. At the country level, annual NMRs ranged from 44·2 to 0·9 deaths per 1000 livebirths in 2017; in 17 countries the annual NMR was more than 30 deaths per 1000 livebirths whereas, in 17 countries, the annual rate was less than 2 deaths per 1000 livebirths (appendix pp 14–23). Countries with the highest NMRs were concentrated in sub-Saharan Africa and south Asia, and countries with the lowest NMRs were in western Europe.

The survival of neonates has improved substantially between 1990 and 2017: we found a 51% reduction in the NMR and a decrease in the annual number of deaths from 5 million to 2·5 million. An estimated 103 million neonates died during this time and, although an acceleration in neonatal mortality reductions (ie, the ARR) since 2000 can be observed, the acceleration was less pronounced in neonates than among other children younger than 5 years. Progress in reducing neonatal mortality in the high-mortality regions in sub-Saharan Africa has been modest, with a regional average ARR of 1·9% during 1990–2017. However, current neonatal mortality remains high; in almost a third of the countries in the region, annual NMRs were more than 30 deaths per 1000 livebirths in 2017, and two-thirds of countries that are at risk of missing the SDG neonatal mortality target are in sub-Saharan Africa, according to our projections.

Focusing on the neonatal period implies that policy makers need to address the main causes of neonatal mortality, which differ from the causes of deaths of older children. Neonates predominantly die because of preterm birth and intrapartum-related complications and infections, such as sepsis, meningitis, and pneumonia. According to estimates by WHO and the Maternal and Child Epidemiology Estimation group, 35% of all neonatal deaths in 2017 were due to complications associated with preterm birth; 24% of deaths were associated with intrapartum events, such as birth asphyxia; 14% of deaths were due to sepsis or meningitis; and 11% were associated with congenital anomalies.^15^ More neonates survived birth and avoided infectious diseases in 2017 than in 2000, and the proportion of deaths associated with premature births and congenital anomalies increased during this time. Prevention of neonatal deaths due to prematurity and congenital anomalies could help to reduce neonatal mortality, even in low-mortality settings. Efforts are also needed to address disparities within countries, since children born in poorer resource settings often have worse health outcomes than children with more resources.^16^, ^17^

To improve neonatal survival, it is crucial to ensure every pregnant woman and neonate has access to lifesaving interventions.^18^, ^19^, ^20^, ^21^ A substantial proportion of neonatal deaths can be prevented by relatively straightforward but effective interventions delivered along the continuum of care during pre-pregnancy, antenatal, intrapartum, delivery, postpartum, and postnatal periods for mothers and their newborns. It is essential that newborn babies receive appropriate care and nutritional support.^22^ Prematurity and low birthweight can be largely addressed through interventions related to antenatal care, education, nutrition, and maternal health. Further improvements in neonatal survival will require a higher proportion of deliveries occurring in well equipped facilities with high-quality care. Considerable investments in terms of training and health infrastructure are needed to enable skilled birth attendants to deliver lifesaving interventions, especially during delivery and the first week of life.^23^ Additionally, improvements in postnatal care interventions need to be scaled up, particularly in settings with high neonatal mortality, to increase the prevalence of neonatal survival beyond the first week.^24^

A focus of health-care interventions on births is essential, since about a third of all neonatal deaths globally occur on the day of birth and almost three-quarters of neonates who die do so during the first week of life.^24^, ^25^ Neonatal deaths often happen quickly, caused by an illness presenting as an emergency, either soon after the birth or later, due to infections such as tetanus or community-acquired infections.^26^ Improved data on where and when neonatal death occur and what causes delays is key to designing context-specific community and health system strategies. To do so, it is necessary to focus on homes and facilities as well as on communication and transportation, to reduce delays in service deliveries, but also to intensify research to identify obstacles in service deliveries.

Our estimates suggest that south Asia is a notable outlier with respect to its ratio of neonatal to under-5 mortality and overall level of under-5 mortality; we found that this region has one of the highest ratios of NMR to U5MR and a high overall U5MR compared with other regions. The neonatal mortality in south Asian countries tends to be higher than expected given the global pattern. Household survey data that track important components of health for mothers and neonates indicate that the prevalence of low birthweights (ie, less than 2·5 kg) for the years 2009–13 was highest in south Asia, with around a quarter of births with a low birthweight, and a prevalence of low birthweight of more than 10% of births in sub-Saharan Africa.^27^ Further coverage data from household surveys suggest that the skilled birth attendance is low, with about 73% of births attended by skilled health personnel in south Asia (with a range of 51–86% in the countries).^28^ Besides coverage, attention needs to be placed on the quality of care, since studies^23^, ^29^ show that survival benefits expected for neonates delivered by skilled health personnel are not always met in sub–Saharan Africa and Asia, due to a lower quality of care. Exclusively breastfeeding with breastmilk for the first 6 months, which reduces the risk of infection-related neonatal mortality compared with partial breastfeeding,^26^ is practised for around 33% of children born in west and central Africa, 52% in south Asia and around 56% in east and south Africa. Maternal health and nutritional status are important factors in neonatal health that could contribute to higher NMRs. Additionally, national averages can hide disparities within regions. According to the Million Death study^17^ in India, the neonatal mortality caused by prematurity or low birthweight was increasing in rural areas and poorer states from 2000 to 2015, while it decreased in urban areas and richer states. Evidence is not sufficient to conclude on the related factors, and poor availability of high-quality data make it challenging to identify the underlying causes for this pattern. More research is required to identify the constraints in implementing interventions to reduce neonatal mortality and to understand the mechanism behind disparities in regions and countries and within countries.

In the absence of reliable and standardised vital registration and administrative data in many countries, modelling of NMRs remains necessary for public health policy and priority setting and monitoring. Only around 70% of children younger than 5 years have a birth certificate,^30^ and most neonatal deaths do not result in the issue of a death certificate.^25^ Data on causes of neonatal deaths and the timing around neonatal deaths are often sparse and less reliable than all-cause mortality data, and these data result in uncertain estimates, which posess substantial challenges to the generation of evidence-based interventions to prevent neonatal deaths. Self-reported neonatal deaths in surveys can result in misclassification errors,^31^ particularly regarding stillbirths. Given the error in national data collection systems and surveys, considerable uncertainty around both mortality rates and progress in reducing them remains. For example, in our data based on point estimates, more than 102 countries halved their NMR since 1990, but only 55 countries halved their rate with 95% probability. More investment is urgently required to improve data collection and data quality, to better distinguish real country-level effects from data issues. Additionally, more in-depth studies are required to analyse potential reporting biases for stillbirths, early neonatal deaths, and livebirths in surveys, surveillance data, and vital registration data. Even in high-quality civil registration systems, large international differences in recording of births and deaths exist between countries, which can lead to variation in NMRs and stillbirth rates, particularly at extremely early gestations when survival is low.^32^ To better estimate neonatal mortality, improvements to counting methods for neonatal deaths and stillbirths is crucial for tracking SDG targets and improving vital statistics.

Fewer countries showed an outlying pattern in 2017 than in 1990. This decrease was mostly due to fewer countries with a lower-than-expected NMR given the level of under-5 mortality in 2017 than in 1990. One could assume that under-reporting of neonatal deaths is more likely than over-reporting, and this bias would result in lower-than-expected neonatal mortality estimates in our model rather than in higher-than-expected neonatal mortality estimates. With improved reporting of neonatal deaths over time, we would expect fewer outliers with lower-than-expected neonatal mortality for recent years than for past years. Given that we cannot clearly distinguish data quality issues from abnormal country patterns, more in-depth country analysis is needed to support this conclusion of improved data quality.

At the global level, our neonatal mortality estimates for 2017 are similar to those produced for the same year in 2018 by the Global Burden of Disease (GBD) study.^33^ The GBD global neonatal mortality was estimated as 16·8 deaths per 1000 livebirths^34^ in 2017 and the annual U5MR was estimated as 38·9 deaths per 1000 livebirths (or 40·6 in the appendix of the same Article).^33^ Additionally, the projections by the Institute of Health Metrics and Evaluation (IHME) in 2017, based on past trends, suggest that the global NMR would decrease to 11·8 deaths per 1000 livebirths by 2030 from 16·8 deaths per 1000 livebirths in 2017.^34^ These results are similar to our scenario 2, of maintaining current trends in ARRs of NMR, in which we projected a global annual NMR of 13·2 deaths per 1000 livebirths in 2030. The correlation coefficient of the two sets of country-specific point estimates for the 187 countries assessed for 1990–2017 is 0·97 for NMR, 0·98 for U5MR, and 0·90 for the ratio of NMR to U5MR. Differences between the two sets of estimates arise through differences in input data, processing of input data, and modelling approaches. For most countries, differences in the neonatal and under-5 mortality rates are small. Large differences are found for some countries between UN IGME and IHME GBD estimates: 29 countries showed an absolute difference in NMR between the datasets that was more than five deaths per 1000 livebirths and relative differences greater than 10% in 2017, and 32 countries showed an absolute difference in the U5MR between the datasets that was more than ten deaths per 1000 livebirths and a relative difference greater than 10% in 2017. Differences in the mortality rates tend to occur in countries with no recent available input data, small populations, or recent crises. Finally, for the ratio of NMR to U5MR, 23 countries showed an absolute difference between these datasets of 0·1 and a relative difference greater than 10% in 2017. Differences in the ratios occur mostly at very low levels of mortality. A comparison of the estimates for NMR, U5MR, and ratio of NMR to U5MR for all 187 countries for the years 1990, 2000, and 2017 is shown in the appendix (pp 39–51). A previous decomposition study on the differences in under-5 mortality estimates between the UN IGME and GBD shows that differences in input data contributed substantially to the discrepancies in the estimates.^35^ We expect that, with increasing data availability, the estimates will further converge.

To conclude, to advance child survival and achieve the SDG goals, it is important to focus on neonatal survival. Without intensified commitment to neonatal survival, many countries will not be able to meet the SDG goal to end preventable child deaths. Our projections suggest that more countries are at risk of missing the neonatal mortality SDG target than the under-5 mortality target (appendix pp 60–61). Half of the more than 60 countries that would not achieve the neonatal mortality SDG target if they continue their current ARR in NMR would only achieve the target after 2050. Almost two-thirds of these countries are in sub-Saharan Africa and two are in south Asia. Without acceleration, our projections indicate that, with no improvement in neonatal mortality, 27·8 million neonates will die between 2018 and 2030. If interventions were scaled-up and the quality of care increased, then the lives of thousands of newborns could be saved and the SDG target achieved in countries that are behind in reducing neonatal mortality. If the SDG target would be achieved on time in the countries that are at risk of falling behind, the lives of 5 million neonates could be saved from 2018 to 2030. Far more newborn lives could be saved if every country achieved the average NMR of high-income countries, indicating that progress should not end with achieving the SDG neonatal mortality target.

For the **Sustainable Development goals** see https://sustainabledevelopment.un.org/

For **UN IGME child mortality data** see www.childmortality.org
