# Correction to *Lancet Glob Health* 2019; 7: e710–20

**DOI:** 10.1016/S2214-109X(19)30251-7

**Published:** 2019-06-10

*Hug L, Alexander M, You D, Alkema L, on behalf of the UN Inter-agency Group for Child Mortality Estimation. National, regional, and global levels and trends in neonatal mortality between 1990 and 2017, with scenario-based projections to 2030: a systematic analysis.* Lancet Glob Health *2018; **7**: e710–20*—In this Article, data in the appendix (pp 10–11) were incorrect. These corrections have been made as of June 10, 2019.

