# Supplementary material for: National, regional, and global levels and trends in neonatal mortality between 1990 and 2017, with scenario-based projections to 2030: a systematic analysis
Source: Lancet Glob Health. 2019 May 13;7(6):e710–20. doi: 10.1016/S2214-109X(19)30163-9 (PMC6527519; doi:10.1016/S2214-109X(19)30163-9)
Supplement: Supplementary appendix [file mmc1.pdf]

THE LANCET
Global Health

Supplementary appendix

This appendix formed part of the original submission and has been peer reviewed. We post it as supplied by the authors.

Supplement to: Hug L, Alexander M, You D, Alkema L, on behalf of the UN Inter-agency Group for Child Mortality Estimation. National, regional, and global levels and trends in neonatal mortality between 1990 and 2017, with scenario-based projections to 2030: a systematic analysis. *Lancet Glob Health* 2019; **7:** e710–20

Web appendix

National, regional, and global levels and trends in neonatal mortality between 1990 and 2017, with scenario-based projections to 2030: a systematic analysis by the United Nations Inter-agency Group for Child Mortality Estimation

Hug, L., Alexander, M., You, D., Alkema, L. for the United Nations Inter-agency Group for Child Mortality Estimation (UN IGME)

Contents

1. Abbreviations 2
2. Methods 2
 2.1. Overview 2
 2.1 Data sources 2
 2.3 Adjustment for missing mothers in high-HIV settings 3
 2.2. Methods 3
 2.2.1 Model 3
 2.2.2 HIV/AIDS and crisis adjustment 4
 2.3 Calculation of deaths and regional aggregates 5
3. Regional classification 6
 3.1. UNICEF classification 6
 3.2 World Bank Group income classification 7
4. Supplementary Tables 9
5. Supplementary Figures 52
References 61

1. Abbreviations

DHS	Demographic and Health Survey
FBH	Full Birth History
NMR	Neonatal mortality rate
MICS	Multiple Indicator Cluster Survey
U5MR	Under-5 mortality rate
UN IGME	United Nations Inter-agency Group for Child Mortality Estimation
SVR	Sample Vital Registration
VR	Vital Registration

2. Methods

2.1. Overview

UN IGME follows the following broad strategy to arrive at annual estimates of child mortality:

1. Compile and assess the quality of all available nationally-representative data relevant to the estimation of child mortality including data from vital registration systems, population censuses, household surveys and sample registration systems.

2. Assess data quality, recalculate data inputs and make adjustments if needed by applying standard methods.

3. Fit a statistical model to these data to generate a smooth trend curve that averages over possibly disparate estimates from the different data sources for a country, taking account of data quality issues.

4. Extrapolate the model to a target year, in this case 2017.

To increase the transparency of the estimation process, the UN IGME has developed a child mortality web portal: CME Info (www.childmortality.org). It includes all available data and shows estimates for each country as well as which data are currently officially used by UN IGME. Once the new estimates are finalized, CME Info will be updated to reflect all available data and the new estimates.

2.2 Data sources

Nationally-representative empirical data relevant to neonatal mortality including data from vital registration (VR) systems, sample registration systems (SVR) and household-based surveys. Table 1 describes the underlying data by source for the neonatal mortality estimation.

For vital registration and sample vital registration data neonatal and under-five mortality rates were calculated from the counts of deaths and births and population.

For data from household-based surveys the full birth history (FBH) is used to calculate neonatal mortality rates and under-five mortality rates. In the FBH women are asked for the date of birth of each of their children, whether the child is still alive, and if not, the age at death. FBH data, collected by all Demographic and Health Surveys (DHS) surveys and increasingly also Multiple Indicator Cluster Surveys (MICS) surveys, allow the calculation of child mortality indicators for specific time periods in the past. Thus DHS and MICS publish under-five child mortality estimates for five 5-year periods before the survey, that is, 0 to 4, 5 to 9, 10 to 14 etc. We re-calculated estimates for calendar year periods, using single calendar years for periods shortly before the survey, and then gradually increasing the number of years for periods further in the past, whenever microdata from the survey is available. The cut-off points for a given survey for

shifting from estimates for single calendar years to two years, or two years to three, etc., are based on the coefficients of variation (a measure of sampling uncertainty) of the estimates[1].

Source	Number of country-years available
VR	3307
SVR	91
DHS (with sampling errors)	1323
DHS (without sampling errors)	59
Other DHS (with sampling errors)	278
Other DHS (without sampling errors)	91
MICS (with sampling errors)	214
MICS (without sampling errors)	37
Others (with sampling errors)	155
Others (without sampling errors)	186

*Table A1: **Summary of neonatal mortality data availability by source type.** 'Other DHS' refers to non-standard* demographic and health surveys*, that is, Special Interim and National DHS, Malaria Indicator Surveys, AIDS Indicator Surveys and World Fertility Surveys (WFS). 'Others' includes surveys such as the Reproductive Health Survey and the other national representative surveys.*

2.3 Adjustment for missing mothers in high-HIV settings

In populations severely affected by HIV/AIDS, HIV-positive (HIV+) children will be more likely to die than other children, and will also be less likely to be reported since their mothers will have been more likely to die also. Child mortality estimates from birth histories will thus be biased downwards. The magnitude of the bias will depend on the extent to which the elevated under-five mortality of HIV+ children is not reported because of the deaths of their mothers. The U5MR adjusts for HIV/AIDS related mortality for each survey data observation from full birth histories during HIV/AIDS epidemics (1980-present), by adopting a set of simplified but reasonable assumptions about the distribution of births to HIV+ women, primarily relating to the duration of their infection, vertical transmission rates, and survival times of both mothers and children from the time of the birth[2]. This method was applied to all World Fertility Surveys, as well as the DHS and MICS.

2.4. Methods

The neonatal mortality rate (NMR) is defined as the probability of dying before 28 days per 1000 live births. In 2015 the UN IGME method for estimating NMR were updated. A complete technical description of the new model is available elsewhere[3]; however, the following sections summarize the main approach.

2.4.1 Model

We model the ratio $R_{c,t}$, which refers to the ratio of NMR to the difference of U5MR and NMR in country *c* and year *t*, i.e. $R_{c,t} =$ NMR/(U5MR - NMR). For each country-year, we assume that the ratio is given by:

$$R_{c,t} = W_{c,t} \times P_{c,t}$$

where

- $W_{c,t}$ refers to the expected ratio for that country-year,
- Country multiplier $P_{c,t}$ represents country-specific trends in the ratio over time that differ from the expected level.

Modeling was done on the log scale to ensure that the ratio is always positive. As U5MR decreases, the proportional share of mortality in the first month of life tends to increase (see Figure 5 in the main text). The $W_{c,t}$ term accounts for this relationship; it is the expected ratio for the country-year based on the UN IGME-estimated U5MR for that country-year. It is modeled as a linear function of U5MR on the log scale with a changing slope:

$$\log W_{c,t} = \beta_0 \qquad \text{if } U5MR_{c,t} < U_{cut}$$

$$\log W_{c,t} = \beta_0 + \beta_1 U5MR_{c,t} \qquad \text{if } U5MR_{c,t} \geq U_{cut}$$

U_{cut} is an estimated constant that represents the level of U5MR after which as U5MR increases, the ratio NMR/(U5MR - NMR) decreases. The parameters of this model are estimated based on all available data such that $W_{c,t}$ represents a 'global relation' between the ratio and U5MR.

The country multiplier $P_{c,t}$ is modeled on the log scale with a B-splines regression model:

$$\log P_{c,t} = \sum_{k=1}^{K_c} B_{c,k}(t)\alpha_{c,k}$$

The $B_{c,k}(t)$ refers to the *k*-th B-spline function for country *c* evaluated at time *t* and $\alpha_{c,k}$ is the *k*-th splines coefficient for country *c*. The spline coefficients are modeled as a combination of an overall mean, λ_c, which can be interpreted as a country-specific intercept, and fluctuations around that mean:

$$\alpha_{c,k} = \lambda_c + \Delta\varepsilon_{c,k}$$

Both the country-specific intercept and fluctuations are modeled hierarchically. For any particular country, the ratio can overall be higher- or lower-than-expected given the level of U5MR in that country, but the fluctuations allow this relationship to change over time within a country. By modeling the fluctuations hierarchically, a degree of smoothness is imposed to ensure relatively smooth trajectories for any given country through time. We model the ratio of NMR/(U5MR - NMR); estimates of NMR are obtained by recombining the estimates of the ratio with UN IGME-estimated U5MR.

2.4.2 HIV/AIDS and crisis adjustment

2.4.2.1 HIV/AIDS adjustment

For neonatal mortality in HIV-affected populations, the ratio is estimated initially for AIDS free mortality. The NMR ratio for HIV/AIDS affected countries is based on HIV/AIDS-free input data, using HIV/AIDS-free U5MR rates to obtain the expected ratio. No HIV/AIDS-related deaths were deducted from the input NMR, thereby assuming that HIV/AIDS-related deaths only affect child mortality after the first month of life[4]. Therefore, it was also assumed that HIV/AIDS does not increase the NMR, hence no post-estimation processing was carried out.

2.4.2.2 Crisis adjustment

For some countries, there are known natural or political crises that have caused an excess of deaths that are not sufficiently captured in survey data. Adjustments were made to the relevant crisis country-years, based on estimates obtained from a variety of sources. Estimated deaths for major crises were derived from various data sources from 1990 to present for the age groups 0-4 and an equal split was used to obtain the number of neonatal deaths. Natural disaster deaths were obtained from the CRED International Disaster Database[5], with under-5 proportions estimated as described elsewhere[6], and conflict deaths were taken from and conflict deaths were taken from Uppsala Conflict Data Program/Peace Research Institute Oslo datasets as well as reports prepared by the United Nations and other organizations. Estimated child deaths due to major crises were included if they met the following criteria:

1. The crisis was isolated to a few years

2. Under-five crisis deaths were >10% of under-five non-crisis deaths

3. Crisis U5MR > 0.2 per 1,000

4. Number of under-five crisis deaths >10 deaths.

or

1. High quality vital registration data are available and should not be smoothed by the NMR model

These criteria resulted in 16 different crises being explicitly incorporated into the NMR estimates. NMR/U5MR ratios for crisis country-years was firstly done based on crisis-free input data, using crisis-free U5MR and NMR estimates. Adjustments to crisis-affected country-years were then made post-estimation. Crisis neonatal deaths are added back on to the neonatal deaths to compute the total estimated neonatal mortality rate.

2.5 Calculation of deaths and regional aggregates

A birth-week cohort method is used to calculate the absolute number of deaths among neonates and regional aggregates. First, each annual birth cohort is divided into 52 equal birth-week cohorts. Then each birth-week cohort is exposed throughout the first month of life to the appropriate calendar year- and age-specific mortality rates depending on cohort age. The annual number of live births estimate in each country from the World Population Prospects: the 2017[7] revision is used to calculate to numbers of deaths.

3. Regional classification

3.1. UNICEF classification

East Asia and Pacific

Australia; Brunei Darussalam; Cambodia; China; Cook Islands; Democratic People's Republic of Korea; Fiji; Indonesia; Japan; Kiribati; Lao People's Democratic Republic; Malaysia; Marshall Islands; Micronesia (Federated States of); Mongolia; Myanmar; Nauru; New Zealand; Niue; Palau; Papua New Guinea; Philippines; Republic of Korea; Samoa; Singapore; Solomon Islands; Thailand; Timor-Leste; Tokelau; Tonga; Tuvalu; Vanuatu; Viet Nam

Europe and Central Asia

Eastern Europe and Central Asia; Western Europe

Eastern Europe and Central Asia

Albania; Armenia; Azerbaijan; Belarus; Bosnia and Herzegovina; Bulgaria; Croatia; Georgia; Kazakhstan; Kyrgyzstan; Montenegro; Republic of Moldova; Romania; Russian Federation; Serbia; Tajikistan; the former Yugoslav Republic of Macedonia; Turkey; Turkmenistan; Ukraine; Uzbekistan

Western Europe

Andorra; Austria; Belgium; Cyprus; Czechia; Denmark; Estonia; Finland; France; Germany; Greece; Holy See; Hungary; Iceland; Ireland; Italy; Latvia; Liechtenstein; Lithuania; Luxembourg; Malta; Monaco; Netherlands; Norway; Poland; Portugal; San Marino; Slovakia; Slovenia; Spain; Sweden; Switzerland; United Kingdom

Latin America and Caribbean

Anguilla; Antigua and Barbuda; Argentina; Bahamas; Barbados; Belize; Bolivia (Plurinational State of); Brazil; British Virgin Islands; Chile; Colombia; Costa Rica; Cuba; Dominica; Dominican Republic; Ecuador; El Salvador; Grenada; Guatemala; Guyana; Haiti; Honduras; Jamaica; Mexico; Montserrat; Nicaragua; Panama; Paraguay; Peru; Saint Kitts and Nevis; Saint Lucia; Saint Vincent and the Grenadines; Suriname; Trinidad and Tobago; Turks and Caicos Islands; Uruguay; Venezuela (Bolivarian Republic of)

Middle East and North Africa

Algeria; Bahrain; Egypt; Iran (Islamic Republic of); Iraq; Israel; Jordan; Kuwait; Lebanon; Libya; Morocco; Oman; Qatar; Saudi Arabia; State of Palestine; Syrian Arab Republic; Tunisia; United Arab Emirates; Yemen

North America

Canada; United States of America

South Asia

Afghanistan; Bangladesh; Bhutan; India; Maldives; Nepal; Pakistan; Sri Lanka

Sub-Saharan Africa

Eastern and Southern Africa; West and Central Africa

Eastern and Southern Africa

Angola; Botswana; Burundi; Comoros; Djibouti; Eritrea; Eswatini; Ethiopia; Kenya; Lesotho; Madagascar; Malawi; Mauritius; Mozambique; Namibia; Rwanda; Seychelles; Somalia; South Africa; South Sudan; Sudan; Uganda; United Republic of Tanzania; Zambia; Zimbabwe

West and Central Africa

Benin; Burkina Faso; Cabo Verde; Cameroon; Central African Republic; Chad; Congo; Côte d'Ivoire; Democratic Republic of the Congo; Equatorial Guinea; Gabon; Gambia; Ghana; Guinea; Guinea-Bissau; Liberia; Mali; Mauritania; Niger; Nigeria; Sao Tome and Principe; Senegal; Sierra Leone; Togo

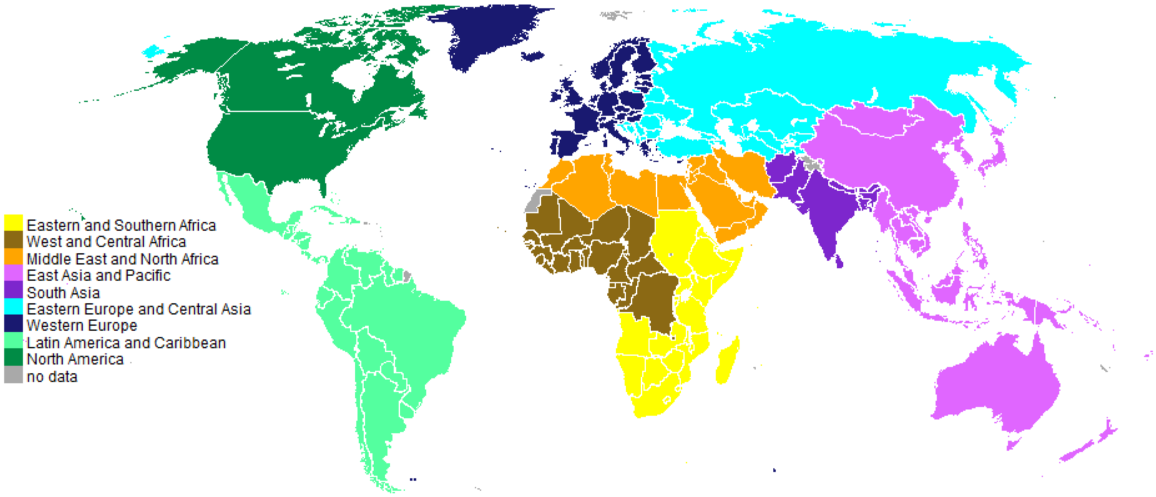

Map 1: UNICEF regional classifications. This map does not reflect a position by UN IGME agencies or those of the institutions to which the authors are affiliated on the legal status of any country or territory or the delimitation of any frontiers.

3.2 World Bank Group income classification

Low income

Afghanistan; Benin; Burkina Faso; Burundi; Central African Republic; Chad; Comoros; Democratic Republic of the Congo; Eritrea; Ethiopia; Gambia; Guinea; Guinea-Bissau; Haiti; Democratic People's Republic of Korea; Liberia; Madagascar; Malawi; Mali; Mozambique; Nepal; Niger; Rwanda; Senegal; Sierra Leone; Somalia; South Sudan; United Republic of Tanzania; Togo; Uganda; Zimbabwe

Lower middle income

Angola; Armenia; Bangladesh; Bhutan; Bolivia (Plurinational State of); Cambodia; Cameroon; Cape Verde; Congo; Côte d'Ivoire; Djibouti; Egypt; El Salvador; Eswatini; Federated States of Micronesia; Georgia; Ghana; Guatemala; Honduras; India; Indonesia; Jordan; Kenya; Kiribati; Kyrgyzstan; Lao People's Democratic Republic; Lesotho; Mauritania; Moldova; Mongolia; Morocco; Myanmar; Nicaragua; Nigeria; Pakistan; Papua New Guinea; Philippines; Sao Tome and Principe; Solomon Islands; Sri Lanka; State of Palestine; Sudan; Syrian Arab Republic; Tajikistan; Timor-Leste; Tunisia; Ukraine; Uzbekistan; Vanuatu; Viet Nam; Yemen; Zambia

Upper-middle income

Albania; Algeria; Argentina; Azerbaijan; Belarus; Belize; Bosnia and Herzegovina; Botswana; Brazil; Bulgaria; China; Colombia; Costa Rica; Croatia; Cuba; Dominica; Dominican Republic; Ecuador; Equatorial Guinea; Fiji; Gabon; Grenada; Guyana; Iran (Islamic Republic of); Iraq; Jamaica; Kazakhstan; Lebanon; Libya; Malaysia; Maldives; Marshall Islands; Mauritius; Mexico; Montenegro; Namibia; Nauru; Panama; Paraguay; Peru; Romania; Russian Federation; Saint Lucia; Samoa; Serbia; South Africa; Saint Vincent and the Grenadines; Suriname; Thailand; The former Yugoslav Republic of Macedonia; Tonga; Turkey; Turkmenistan; Tuvalu; Venezuela (Bolivarian Republic of)

High income

Andorra; Antigua and Barbuda; Australia; Austria; Bahamas; Bahrain; Barbados; Belgium; Brunei; Canada; Chile; Cyprus; Czechia; Denmark; Estonia; Finland; France; Germany; Greece; Hungary; Iceland; Ireland; Israel; Italy; Japan; Republic of Korea; Kuwait; Latvia; Liechtenstein; Lithuania; Luxembourg; Malta; Monaco; Netherlands; New Zealand; Norway; Oman; Palau; Poland; Portugal; Qatar; Saint Kitts and Nevis; San Marino; Saudi Arabia;

Seychelles; Singapore; Slovakia; Slovenia; Spain; Sweden; Switzerland; Trinidad and Tobago; United Arab Emirates; United Kingdom; United States of America; Uruguay

3.3 Sustainable Development Goal regional classification

Sub-Saharan Africa

Angola; Benin; Botswana; Burkina Faso; Burundi; Cabo Verde; Cameroon; Central African Republic; Chad; Comoros; Congo; Côte d'Ivoire; Democratic Republic of the Congo; Djibouti; Equatorial Guinea; Eritrea; Eswatini; Ethiopia; Gabon; Gambia; Ghana; Guinea;; Guinea-Bissau; Kenya; Lesotho; Liberia; Madagascar; Malawi; Mali; Mauritania; Mauritius; Mozambique; Namibia; Niger; Nigeria; Rwanda; Sao Tome and Principe; Senegal; Seychelles; Sierra Leone; Somalia; South Africa; South Sudan; Togo; Uganda; United Republic of Tanzania; Zambia; Zimbabwe

Northern Africa and Western Asia

Northern Africa

Algeria; Egypt; Libya; Morocco; Sudan; Tunisia

Western Asia

Armenia; Azerbaijan; Bahrain; Cyprus; Georgia; Iraq; Israel; Jordan; Kuwait; Lebanon; Oman; Qatar; Saudi Arabia; State of Palestine; Syrian Arab Republic; Turkey; United Arab Emirates; Yemen

Central and Southern Asia

Central Asia

Kazakhstan; Kyrgyzstan; Tajikistan; Turkmenistan; Uzbekistan

Southern Asia

Afghanistan; Bangladesh; Bhutan; India; Iran (Islamic Republic of); Maldives; Nepal; Pakistan; Sri Lanka

Eastern and South-Eastern Asia

Eastern Asia

China; Democratic People's Republic of Korea; Japan; Mongolia; Republic of Korea

South-Eastern Asia

Brunei Darussalam; Cambodia; Indonesia; Lao People's Democratic Republic; Malaysia; Myanmar; Philippines; Singapore; Thailand; Timor-Leste; Viet Nam

Latin America and the Caribbean

Antigua and Barbuda; Argentina; Bahamas; Barbados; Belize; Bolivia (Plurinational State of); Brazil; Chile; Colombia; Costa Rica; Cuba; Dominica; Dominican Republic; Ecuador; El Salvador; Grenada; Guatemala; Guyana; Haiti; Honduras; Jamaica; Mexico; Nicaragua; Panama; Paraguay; Peru; Saint Kitts and Nevis; Saint Lucia; Saint Vincent and the Grenadines; Suriname; Trinidad and Tobago; Uruguay; Venezuela (Bolivarian Republic of)

Oceania

Australia and New Zealand

Australia; New Zealand

Oceania (excluding Australia and New Zealand)

Cook Islands; Fiji; Kiribati; Marshall Islands; Micronesia (Federated States of); Nauru; Niue; Palau; Papua New Guinea; Samoa; Solomon Islands; Tonga; Tuvalu; Vanuatu

Europe and Northern America

Northern America

Canada; United States of America

Europe

Albania; Andorra; Austria; Belarus; Belgium; Bosnia and Herzegovina; Bulgaria; Croatia; Czechia; Denmark; Estonia; Finland; France; Germany; Greece; Hungary; Iceland; Ireland; Italy; Latvia; Lithuania; Luxembourg; Malta; Monaco; Montenegro; Netherlands; Norway; Poland; Portugal; Republic of Moldova; Romania; Russian Federation; San Marino; Serbia; Slovakia; Slovenia; Spain; Sweden; Switzerland; The former Yugoslav Republic of Macedonia; Ukraine; United Kingdom of Great Britain and Northern Ireland

4. Supplementary Tables

Table A.2. Global and SDG regional neonatal mortality rate (deaths per 1,000 live births) and number of neonatal deaths, ratio of neonatal to under-five mortality rate and share of neonatal deaths in under-five deaths, 1990, 2000 and 2017 with 90% uncertainty intervals

	Neonatal mortality rate (deaths per 1,000 live births)				Neonatal deaths (thousands)				
	1990	2000	2017	% decrease 1990 to 2017 (%)	1990	2000	2017	% decrease 1990 to 2017 (%)	Share of total deaths worldwide in 2017 (%)
**World**	36.6	30.6	18.0	51	5038	3997	2533	50	100
	(35.5, 37.8)	(29.8, 31.6)	(17.0, 19.9)	(46, 54)	(4891, 5202)	(3891, 4115)	(2381, 2789)	(45, 53)	(100, 100)
**By region**									
Sub-Saharan Africa	45.9	40.8	27.2	41	997	1100	999	0	39
	(44.2, 47.8)	(39.3, 42.6)	(24.5, 31.6)	(31, 47)	(959, 1039)	(1060, 1147)	(902, 1164)	(-17, 10)	(34, 40)
Northern Africa and Western Asia	30.7	23.2	14.2	54	287	215	161	44	6
	(29.5, 31.9)	(22.3, 24.1)	(12.8, 16.5)	(46, 58)	(277, 298)	(206, 223)	(146, 188)	(34, 49)	(6, 7)
Northern Africa	33.1	26.3	16.3	51	155	115	94	39	4
	(31.6, 34.7)	(24.9, 27.8)	(14.1, 19.2)	(41, 58)	(148, 162)	(109, 122)	(81, 111)	(28, 48)	(3, 4)
Western Asia	28.2	20.3	11.9	58	132	99	67	49	3
	(26.6, 29.9)	(19.3, 21.5)	(10.4, 15.1)	(46, 63)	(125, 140)	(94, 105)	(59, 85)	(35, 56)	(2, 3)
Central and Southern Asia	56	45.1	25.7	54	2276	1828	991	56	39
	(53.6, 58.5)	(43.2, 47.2)	(23.1, 28.9)	(48, 59)	(2180, 2377)	(1750, 1910)	(891, 1115)	(51, 61)	(35, 41)
Central Asia	28.1	26.5	11.6	59	44	32	18	59	1
	(25.5, 31.0)	(23.8, 29.6)	(9.4, 14.8)	(47, 67)	(40, 49)	(29, 36)	(15, 23)	(47, 67)	(1, 1)
Southern Asia	57.1	45.7	26.3	54	2232	1796	973	56	38
	(54.7, 59.7)	(43.7, 47.8)	(23.6, 29.6)	(48, 59)	(2136, 2332)	(1718, 1877)	(873, 1095)	(51, 61)	(34, 40)
Eastern and South-Eastern Asia	27.6	20.1	7.8	72	1105	603	234	79	9
	(25.6, 30.0)	(19.1, 21.3)	(7.0, 8.8)	(67, 75)	(1025, 1202)	(572, 637)	(211, 264)	(75, 82)	(8, 10)
Eastern Asia	27.6	19.7	4.5	84	772	364	84	89	3
	(24.7, 31.0)	(18.2, 21.4)	(3.9, 5.3)	(80, 87)	(693, 867)	(335, 395)	(71, 98)	(87, 91)	(3, 4)
South-Eastern Asia	27.6	20.8	12.8	54	333	239	150	55	6
	(26.4, 29.0)	(19.8, 21.9)	(11.3, 15.1)	(45, 59)	(318, 349)	(228, 251)	(132, 176)	(47, 61)	(5, 6)
Latin America and the Caribbean	22.6	15.8	9.6	58	268	183	103	62	4
	(21.4, 23.8)	(14.9, 16.7)	(9.2, 10.4)	(53, 60)	(254, 283)	(173, 194)	(97, 110)	(58, 64)	(4, 4)
Oceania	13.5	13.5	10.3	24	7	7	7	3	0

	(12.1, 14.9)	(11.9, 15.5)	(6.7, 17.3)	(-29, 51)	(6, 8)	(7, 9)	(4, 11)	(-63, 38)	(0, 0)
Australia and New Zealand	4.6	3.5	2.3	50	1	1	1	40	0
	(4.4, 4.7)	(3.4, 3.6)	(2.1, 2.4)	(46, 54)	(1, 1)	(1, 1)	(1, 1)	(35, 44)	(NA, NA)
Oceania (exc. Australia and New Zealand)	27	25.8	21.1	22	6	6	6	-6	0
	(23.6, 30.5)	(22.1, 30.3)	(12.6, 37.1)	(-40, 54)	(5, 6)	(5, 7)	(4, 10)	(-91, 37)	(0, 0)
Europe and Northern America	7.3	5.2	3.1	58	98	60	39	61	2
	(7.0, 7.6)	(5.0, 5.4)	(3.0, 3.3)	(54, 60)	(94, 102)	(58, 62)	(36, 41)	(58, 64)	(1, 1)
Europe	8	5.5	2.9	64	73	41	22	69	1
	(7.7, 8.5)	(5.3, 5.8)	(2.6, 3.1)	(60, 68)	(70, 78)	(39, 43)	(20, 24)	(66, 73)	(1, 1)
Northern America	5.6	4.5	3.6	36	24	20	16	34	1
	(5.5, 5.8)	(4.4, 4.7)	(3.4, 3.8)	(33, 39)	(24, 25)	(19, 20)	(15, 17)	(31, 37)	(1, 1)
Landlocked developing countries	47.7	41.5	25.3	47	527	522	403	23	16
	(45.9, 49.7)	(39.9, 43.2)	(23.3, 28.8)	(39, 52)	(507, 549)	(503, 544)	(370, 459)	(13, 30)	(14, 17)
Least developed countries	51.9	41.8	26.2	50	1129	1076	838	26	33
	(50.3, 53.8)	(40.5, 43.3)	(24.2, 29.8)	(43, 54)	(1093, 1169)	(1043, 1115)	(775, 953)	(16, 32)	(28, 33)
Small island developing States	26.7	23.4	19.6	27	32	28	24	26	1
	(25.3, 28.2)	(22.1, 24.9)	(16.8, 24.6)	(8, 38)	(31, 34)	(27, 30)	(21, 30)	(7, 37)	(1, 1)

	**Ratio neonatal to under-five mortality**					**Share of neonatal deaths in under-five deaths (%)**				
	1990	2000	2017	% increase 1990 to 2017 (%)	Absolute increase 1990 to 2017 (%)	1990	2000	2017	% increase 1990 to 2017 (%)	Absolute increase 1990 to 2017 (%)
**World**	0.39	0.40	0.46	15	0.07	40	41	47	14	7
	(0.38, 0.40)	(0.39, 0.41)	(0.43, 0.49)	(9, 20)	(0.04, 0.10)	(39, 41)	(40, 42)	(44, 50)	(9, 20)	(4, 10)
**By region**										
Sub-Saharan Africa	0.24	0.25	0.33	30	0.11	42	46	53	28	10
	(0.25, 0.26)	(0.26, 0.27)	(0.36, 0.39)	(24, 36)	(0.08, 0.14)	(40, 43)	(45, 48)	(50, 57)	(22, 34)	(8, 14)
Northern Africa and Western Asia	0.42	0.47	0.56	21	0.11	40	45	52	21	11
	(0.40, 0.41)	(0.44, 0.46)	(0.49, 0.53)	(17, 28)	(0.08, 0.16)	(39, 41)	(43, 47)	(49, 56)	(16, 27)	(8, 15)
Northern Africa	0.41	0.46	0.56	24	0.13	39	42	47	24	12
	(0.38, 0.39)	(0.43, 0.44)	(0.49, 0.52)	(18, 30)	(0.09, 0.17)	(36, 42)	(38, 45)	(38, 55)	(18, 30)	(9, 17)
Western Asia	0.45	0.49	0.59	17	0.09	26	27	37	17	9
	(0.41, 0.43)	(0.45, 0.47)	(0.49, 0.54)	(12, 28)	(0.06, 0.16)	(25, 27)	(27, 28)	(34, 40)	(11, 27)	(6, 16)
Central and Southern Asia	0.44	0.48	0.56	25	0.15	39	42	47	24	14
	(0.45, 0.47)	(0.50, 0.51)	(0.60, 0.63)	(19, 29)	(0.11, 0.18)	(36, 42)	(38, 45)	(38, 55)	(18, 29)	(11, 18)
Central Asia	0.39	0.42	0.5	22	0.11	42	46	53	21	10
	(0.42, 0.36)	(0.45, 0.39)	(0.57, 0.43)	(7, 33)	(0.03, 0.19)	(40, 43)	(45, 48)	(50, 57)	(6, 33)	(2, 18)
Southern Asia	0.45	0.5	0.6	25	0.15	40	45	52	24	14
	(0.47, 0.44)	(0.51, 0.48)	(0.64, 0.57)	(19, 30)	(0.11, 0.19)	(39, 41)	(43, 47)	(49, 56)	(18, 29)	(11, 18)
Eastern and South-Eastern Asia	0.48	0.51	0.5	3	0.02	26	27	37	3	2
	(0.51, 0.46)	(0.53, 0.49)	(0.53, 0.47)	(-6, 11)	(-0.03, 0.06)	(25, 27)	(27, 28)	(34, 40)	(-6, 11)	(-3, 6)
Eastern Asia	0.54	0.57	0.51	-9	-0.04	39	42	47	-7	-4
	(0.59, 0.50)	(0.61, 0.54)	(0.55, 0.45)	(-25, 6)	(-0.12, 0.03)	(36, 42)	(38, 45)	(38, 55)	(-24, 6)	(-11, 3)

South-Eastern Asia	0.4 (0.37, 0.38)	0.44 (0.41, 0.43)	0.53 (0.46, 0.49)	22 (16, 28)	0.11 (0.07, 0.14)	42 (40, 43)	46 (45, 48)	53 (50, 57)	22 (15, 27)	11 (7, 14)
Latin America and the Caribbean	0.39 (0.41, 0.43)	0.45 (0.48, 0.50)	0.53 (0.55, 0.57)	24 (20, 29)	0.13 (0.11, 0.16)	26 (25, 27)	27 (27, 28)	37 (34, 40)	24 (20, 28)	13 (11, 16)
Oceania	0.35 (0.39, 0.41)	0.37 (0.41, 0.44)	0.37 (0.46, 0.55)	16 (-4, 33)	0.07 (-0.01, 0.18)	40 (39, 41)	45 (43, 47)	52 (49, 56)	15 (-5, 32)	7 (-2, 17)
Australia and New Zealand	0.47 (0.48, 0.49)	0.53 (0.55, 0.56)	0.57 (0.60, 0.62)	21 (16, 23)	0.13 (0.09, 0.14)	39 (36, 42)	42 (38, 45)	47 (38, 55)	19 (16, 23)	11 (9, 14)
Oceania (exc. Australia and New Zealand)	0.33 (0.37, 0.40)	0.35 (0.39, 0.43)	0.35 (0.44, 0.54)	17 (-6, 36)	0.08 (-0.02, 0.19)	26 (25, 27)	27 (27, 28)	37 (34, 40)	16 (-7, 35)	7 (-2, 19)
Europe and Northern America	0.54 (0.50, 0.52)	0.56 (0.52, 0.54)	0.57 (0.52, 0.55)	4 (-3, 11)	0.02 (-0.01, 0.06)	39 (36, 42)	42 (38, 45)	47 (38, 55)	6 (-1, 12)	3 (-1, 7)
Europe	0.5 (0.52, 0.55)	0.51 (0.53, 0.56)	0.49 (0.54, 0.58)	5 (-9, 13)	0.03 (-0.05, 0.07)	40 (39, 41)	45 (43, 47)	52 (49, 56)	5 (-7, 14)	3 (-3, 8)
Northern America	0.5 (0.51, 0.52)	0.54 (0.55, 0.56)	0.54 (0.56, 0.57)	8 (6, 10)	0.04 (0.03, 0.06)	42 (40, 43)	46 (45, 48)	53 (50, 57)	8 (5, 10)	4 (3, 6)
Landlocked developing countries	0.29 (0.28, 0.28)	0.31 (0.29, 0.30)	0.45 (0.39, 0.42)	32 (26, 37)	0.13 (0.10, 0.16)	40 (39, 41)	45 (43, 47)	52 (49, 56)	30 (25, 35)	13 (10, 16)
Least developed countries	0.31 (0.29, 0.30)	0.31 (0.30, 0.30)	0.43 (0.37, 0.40)	25 (19, 31)	0.1 (0.07, 0.13)	42 (40, 43)	46 (45, 48)	53 (50, 57)	24 (18, 30)	10 (7, 13)
Small island developing States	0.35 (0.32, 0.34)	0.4 (0.36, 0.38)	0.51 (0.42, 0.47)	27 (18, 35)	0.12 (0.08, 0.18)	26 (25, 27)	27 (27, 28)	37 (34, 40)	26 (17, 34)	12 (7, 18)

Table A.3. Global and regional ratio of estimated to expected neonatal mortality rate 1990, 2000 and 2017 with 90% uncertainty intervals.

	Ratio of estimated-to-expected neonatal mortality rate			
	1990	2000	2017	Change (1990–2017)
World	1.12	1.07	1.02	-0.10
	(1.10, 1.14)	(1.06, 1.09)	(0.97, 1.08)	(-0.16, -0.03)
**By region**				
Sub-Saharan Africa	0.91	0.89	0.89	-0.01
	(0.89, 0.93)	(0.87, 0.91)	(0.83, 0.97)	(-0.08, 0.07)
Eastern and Southern Africa	0.91	0.90	0.93	0.02
	(0.89, 0.94)	(0.88, 0.93)	(0.88, 1.00)	(-0.05, 0.10)
West and Central Africa	0.90	0.88	0.87	-0.04
	(0.87, 0.93)	(0.85, 0.91)	(0.78, 0.96)	(-0.13, 0.07)
Middle East and North Africa	1.01	1.04	1.07	0.06
	(0.94, 1.06)	(1.00, 1.07)	(1.00, 1.17)	(-0.02, 0.19)
South Asia	1.36	1.31	1.23	-0.13
	(1.33, 1.40)	(1.27, 1.35)	(1.16, 1.30)	(-0.22, -0.05)
East Asia and Pacific	1.08	1.02	0.95	-0.13
	(1.02, 1.14)	(0.98, 1.06)	(0.90, 1.00)	(-0.22, -0.04)
Latin America and Caribbean	0.92	0.95	1.04	0.12
	(0.88, 0.95)	(0.90, 0.99)	(1.02, 1.09)	(0.08, 0.19)
North America	0.97	1.02	1.06	0.09

	(0.96, 0.98)	(1.03, 1.05)	(1.03, 1.07)	(0.06, 0.11)
Europe and Central Asia	0.99	0.98	0.98	-0.01
	(0.95, 1.04)	(0.93, 1.02)	(0.91, 1.06)	(-0.10, 0.08)
Eastern Europe and Central Asia	0.99	0.96	0.94	-0.05
	(0.94, 1.05)	(0.91, 1.01)	(0.86, 1.04)	(-0.16, 0.07)
Western Europe	0.98	1.06	1.10	0.11
	(0.99, 1.00)	(1.05, 1.06)	(1.09, 1.15)	(0.09, 0.15)
**By income group**				
Low income	0.95	0.92	0.92	-0.03
	(0.93, 0.97)	(0.90, 0.94)	(0.86, 1.00)	(-0.10, 0.05)
Lower middle income	1.20	1.15	1.08	-0.12
	(1.17, 1.23)	(1.13, 1.18)	(1.01, 1.14)	(-0.19, -0.05)
Upper middle income	1.07	1.03	0.99	-0.08
	(1.02, 1.13)	(0.99, 1.07)	(0.94, 1.04)	(-0.17, 0.00)
High income	0.97	1.02	1.03	0.06
	(0.92, 1.02)	(0.97, 1.04)	(1.03, 1.21)	(0.05, 0.24)

Table A.4. Projected neonatal mortality rate and neonatal deaths under different scenarios for 2030 and cumulative number of deaths from 2018-2030 by region.

Note: The number of neonatal deaths has been rounded to the nearest 1000.

	Neonatal mortality rate			Neonatal deaths rounded *1000			Cumulative neonatal deaths (in millions) 2018-2030
**Region**	**2020**	**2025**	**2030**	**2020**	**2025**	**2030**	
			**Scenario 1: No change**				
**World**	**18.3**	**18.7**	**19.0**	**2,563,000**	**2,607,000**	**2,654,000**	**33,781,000**
Sub-Saharan Africa	27.3	27.3	27.4	1,085,000	1,167,000	1,249,000	14,962,000
Eastern and Southern Africa	24.2	24.3	24.3	472,000	504,000	535,000	6,470,000
West and Central Africa	30.2	30.2	30.2	613,000	663,000	714,000	8,492,000
Middle East and North Africa	12.7	12.9	13.0	122,000	119,000	120,000	1,566,000
South Asia	26.9	26.9	27.0	955,000	935,000	911,000	12,195,000
East Asia and Pacific	7.9	8.0	8.1	234,000	225,000	218,000	2,956,000
Latin America and Caribbean	9.7	9.7	9.7	101,000	98,000	94,000	1,276,000
North America	3.6	3.6	3.6	17,000	17,000	17,000	218,000
Europe and Central Asia	4.6	4.6	4.5	49,000	46,000	44,000	607,000
Eastern Europe and Central Asia	6.5	6.6	6.6	38,000	35,000	33,000	465,000
Western Europe	2.3	2.3	2.3	11,000	11,000	11,000	142,000
			**Scenario 2: Maintaining current trends**				
**World**	**16.7**	**14.9**	**13.2**	**2,346,000**	**2,074,000**	**1,848,000**	**27,793,000**
Sub-Saharan Africa	25.4	22.7	20.3	1,012,000	969,000	926,000	12,710,000

Eastern and Southern Africa	22.5	19.9	17.6	438,000	412,000	387,000	5,424,000
West and Central Africa	28.3	25.4	22.8	574,000	557,000	539,000	7,286,000
Middle East and North Africa	11.6	10.2	9.0	111,000	95,000	83,000	1,289,000
South Asia	24.4	20.8	17.9	866,000	723,000	603,000	9,833,000
East Asia and Pacific	6.9	5.7	4.9	205,000	161,000	131,000	2,243,000
Latin America and Caribbean	8.9	7.8	6.9	93,000	78,000	67,000	1,063,000
North America	3.4	3.2	3.0	16,000	15,000	14,000	199,000
Europe and Central Asia	4.0	3.2	2.6	43,000	32,000	25,000	455,000
Eastern Europe and Central Asia	5.6	4.3	3.4	32,000	23,000	17,000	332,000
Western Europe	2.1	1.9	1.7	10,000	9,000	8,000	123,000
**Scenario 3: Best regional performer**							
**World**	**15.7**	**12.5**	**10.0**	**2,196,000**	**1,740,000**	**1,391,000**	**24,096,000**
Sub-Saharan Africa	23.9	19.2	15.5	951,000	822,000	707,000	11,061,000
Eastern and Southern Africa	20.8	16.2	12.6	406,000	336,000	277,000	4,580,000
West and Central Africa	26.9	22.1	18.2	546,000	485,000	430,000	6,481,000
Middle East and North Africa	10.5	7.7	5.6	101,000	71,000	52,000	1,030,000
South Asia	23.2	18.2	14.3	823,000	631,000	482,000	8,820,000
East Asia and Pacific	6.1	4.0	2.6	180,000	111,000	70,000	1,696,000
Latin America and Caribbean	8.3	6.6	5.2	87,000	66,000	50,000	928,000
North America	3.4	3.2	3.0	16,000	15,000	14,000	197,000
Europe and Central Asia	3.6	2.4	1.6	38,000	24,000	16,000	363,000
Eastern Europe and Central Asia	5.1	3.4	2.3	30,000	18,000	11,000	279,000
Western Europe	1.8	1.2	0.9	9,000	5,000	4,000	84,000
**Scenario 4: Achieving SDG target**							
**World**	**15.2**	**11.6**	**8.8**	**2,134,000**	**1,613,000**	**1,229,000**	**22,692,000**
Sub-Saharan Africa	22.4	16.3	11.9	892,000	694,000	541,000	9,660,000
Eastern and Southern Africa	20.4	15.4	11.7	397,000	320,000	257,000	4,397,000
West and Central Africa	24.4	17.1	12.0	495,000	375,000	284,000	5,263,000
Middle East and North Africa	11.3	9.5	7.9	108,000	88,000	74,000	1,212,000
South Asia	22.1	16.0	11.8	784,000	557,000	396,000	8,007,000
East Asia and Pacific	6.8	5.5	4.6	202,000	155,000	122,000	2,176,000
Latin America and Caribbean	8.6	7.1	5.9	90,000	72,000	57,000	988,000
North America	3.4	3.2	3.0	16,000	15,000	14,000	199,000

Europe and Central Asia	4.0	3.2	2.5	42,000	32,000	25,000	450,000
Eastern Europe and Central Asia	5.5	4.2	3.3	32,000	23,000	16,000	327,000
Western Europe	2.1	1.9	1.7	10,000	9,000	8,000	123,000
				**Scenario 5: High income**			
**World**	**11.5**	**5.5**	**2.7**	**1,607,000**	**769,000**	**384,000**	**13,485,000**
Sub-Saharan Africa	16.3	7.0	3.0	649,000	298,000	137,000	5,336,000
Eastern and Southern Africa	14.9	6.7	3.0	290,000	138,000	66,000	2,418,000
West and Central Africa	17.7	7.3	3.0	359,000	160,000	71,000	2,917,000
Middle East and North Africa	8.9	5.0	2.9	85,000	47,000	27,000	759,000
South Asia	16.1	6.9	3.0	571,000	240,000	101,000	4,542,000
East Asia and Pacific	5.7	3.5	2.1	170,000	97,000	57,000	1,543,000
Latin America and Caribbean	7.2	4.6	3.0	76,000	46,000	29,000	711,000
North America	3.4	3.2	3.0	16,000	15,000	14,000	198,000
Europe and Central Asia	3.7	2.7	2.0	40,000	27,000	19,000	396,000
Eastern Europe and Central Asia	5.0	3.3	2.2	29,000	18,000	11,000	273,000
Western Europe	2.1	1.9	1.7	10,000	9,000	8,000	123,000

Table A.5. Number of neonatal mortality rates and neonatal deaths, by country or territory
Note: Numbers in brackets are 90% uncertainty intervals.

	Neonatal mortality rate (deaths per 1,000 live births)			Neonatal deaths		
**Country or territory**	**1990**	**2000**	**2017**	**1990**	**2000**	**2017**
Afghanistan	73.5 (65.7, 82.4)	60.8 (55.3, 67.2)	39.2 (29.7, 49.9)	46806 (41851, 52472)	60411 (54888, 66725)	45219 (34219, 57539)
Albania	12.6 (9.9, 15.6)	11 (8.7, 13.6)	6.1 (5.5, 6.6)	996 (781, 1236)	563 (445, 694)	208 (189, 229)
Algeria	23.5 (20.9, 26.0)	21.4 (19.7, 22.9)	14.9 (12.6, 17.1)	19540 (17436, 21670)	13036 (12000, 13999)	13678 (11595, 15685)
Andorra	3.9 (2.2, 6.8)	2.1 (1.4, 3.0)	1.4 (0.8, 2.4)	3 (2, 5)	2 (1, 2)	1 (0, 1)
Angola	54 (44.4, 66.4)	50.7 (42.4, 60.8)	29.1 (13.7, 54.5)	34899 (28684, 42904)	41171 (34446, 49376)	35914 (16915, 67191)
Antigua and Barbuda	14.4 (9.7, 20.8)	8.6 (7.3, 10.3)	3.8 (2.3, 5.9)	19 (13, 27)	15 (12, 18)	6 (4, 10)
Argentina	15 (14.5, 15.4)	10.6 (10.2, 10.9)	5.9 (5.4, 6.5)	10691 (10377, 11001)	7595 (7373, 7814)	4439 (4054, 4865)
Armenia	23 (20.0, 26.6)	15.7 (13.6, 18.0)	6.7 (4.8, 9.3)	1722 (1499, 1991)	622 (538, 714)	255 (182, 355)

Australia	4.6 (4.5, 4.8)	3.5 (3.4, 3.6)	2.1 (2.0, 2.3)	1180 (1140, 1221)	872 (844, 901)	674 (630, 719)
Austria	4.6 (4.4, 4.8)	3.1 (2.9, 3.2)	2.2 (2.0, 2.3)	409 (391, 428)	248 (236, 260)	182 (167, 198)
Azerbaijan	31.2 (25.8, 37.0)	34.1 (28.9, 40.4)	11.8 (7.5, 17.7)	6437 (5333, 7628)	4803 (4065, 5695)	1983 (1272, 2980)
Bahamas	13.7 (12.6, 14.9)	8.3 (7.6, 9.2)	4 (3.0, 5.1)	84 (77, 91)	44 (40, 48)	22 (17, 29)
Bahrain	15 (14.0, 16.0)	4.7 (4.3, 5.2)	3.1 (2.5, 3.9)	215 (202, 229)	70 (64, 77)	68 (55, 84)
Bangladesh	64.1 (60.3, 68.0)	42.4 (39.8, 45.2)	18.4 (16.2, 20.8)	240316 (226123, 255082)	154345 (144783, 164352)	56341 (49535, 63624)
Barbados	11.7 (10.8, 12.7)	9.2 (8.4, 10.1)	8.1 (6.1, 10.8)	49 (45, 53)	34 (31, 37)	27 (20, 36)
Belarus	8.2 (6.4, 10.3)	6 (5.2, 6.8)	1.5 (1.3, 1.8)	1184 (917, 1477)	536 (464, 607)	173 (144, 203)
Belgium	4.6 (4.4, 4.8)	3 (2.8, 3.1)	2.1 (1.8, 2.4)	551 (526, 576)	340 (324, 357)	272 (237, 310)
Belize	18.7 (15.9, 22.1)	11.9 (10.7, 13.1)	8.8 (7.6, 10.2)	125 (106, 147)	87 (78, 97)	74 (64, 86)
Benin	46.2 (41.6, 51.2)	40.1 (35.9, 44.6)	32.7 (23.9, 46.9)	10635 (9578, 11794)	11679 (10481, 12997)	13391 (9793, 19199)
Bhutan	42.7 (31.7, 54.9)	32.1 (26.4, 38.1)	16.9 (10.7, 25.8)	865 (643, 1110)	516 (424, 613)	244 (154, 373)
Bolivia (Plurinational State of)	41.8 (37.9, 45.8)	29.8 (25.8, 33.4)	18.5 (11.9, 28.4)	10146 (9214, 11120)	7690 (6658, 8613)	4697 (3032, 7202)
Bosnia and Herzegovina	11.3 (10.8, 11.7)	6.5 (6.2, 6.8)	4 (3.5, 4.6)	725 (695, 754)	269 (255, 282)	130 (113, 150)
Botswana	25.5 (21.7, 29.8)	26.3 (20.4, 31.8)	25.4 (11.4, 49.6)	1188 (1010, 1384)	1250 (973, 1515)	1346 (606, 2631)
Brazil	25.3 (22.2, 28.5)	18.3 (15.7, 20.9)	8.5 (7.6, 9.6)	94326 (82808, 106379)	64863 (55483, 73843)	24726 (22046, 27815)
Brunei Darussalam	5.7 (5.1, 6.3)	5 (4.6, 5.5)	4.7 (4.0, 5.6)	43 (39, 48)	36 (33, 39)	31 (26, 37)
Bulgaria	8 (7.7, 8.3)	7.9 (7.6, 8.2)	3.7 (3.4, 4.0)	844 (810, 878)	515 (493, 537)	244 (226, 263)
Burkina Faso	45.9 (41.1, 51.1)	41 (36.5, 46.0)	25.4 (17.8, 35.3)	19180 (17177, 21381)	22046 (19641, 24727)	18752 (13139, 26060)
Burundi	40 (34.6, 45.7)	37.1 (32.3, 42.2)	22.1 (15.0, 32.3)	10817 (9367, 12352)	10167 (8844, 11562)	10061 (6831, 14696)
Cabo Verde	20.6 (17.8, 23.4)	17.2 (15.1, 19.5)	10.4 (8.8, 12.1)	285 (247, 324)	218 (191, 248)	117 (99, 136)
Cambodia	40.2 (36.3, 44.7)	35.4 (31.4, 39.6)	14.9 (8.6, 25.9)	15363 (13878, 17083)	12031 (10675, 13439)	5458 (3144, 9496)
Cameroon	39.5	32.9	25.5	20636	20782	21957

	(35.4, 44.0)	(28.9, 37.2)	(17.8, 36.1)	(18499, 22988)	(18258, 23536)	(15360, 31057)
Canada	4.3	3.7	3.5	1685	1246	1342
	(4.2, 4.5)	(3.6, 3.8)	(3.2, 3.7)	(1631, 1738)	(1209, 1284)	(1238, 1455)
Central African Republic	51.6	49.3	41.5	6292	7406	6895
	(44.7, 59.6)	(39.9, 60.6)	(24.8, 70.8)	(5444, 7269)	(5994, 9101)	(4116, 11753)
Chad	52	44.6	34.8	15806	19027	22220
	(46.3, 58.6)	(39.5, 50.1)	(26.3, 45.6)	(14062, 17803)	(16868, 21374)	(16769, 29112)
Chile	8.6	5.5	5	2496	1422	1196
	(8.2, 8.9)	(5.3, 5.7)	(4.4, 5.8)	(2396, 2595)	(1368, 1477)	(1037, 1370)
China	29.5	21.4	4.7	753056	347408	78087
	(26.4, 33.2)	(19.7, 23.3)	(4.0, 5.6)	(673849, 847731)	(319539, 378381)	(65743, 92254)
Colombia	17.9	13.5	8	16218	11424	5850
	(16.3, 19.6)	(12.2, 14.9)	(5.9, 10.9)	(14796, 17748)	(10347, 12640)	(4291, 8000)
Comoros	50	40.9	31.7	877	831	834
	(41.8, 58.9)	(26.5, 56.1)	(12.7, 70.8)	(732, 1032)	(540, 1139)	(334, 1863)
Congo	28	31.5	19.4	2615	3992	3481
	(23.9, 32.7)	(27.4, 36.1)	(12.3, 30.7)	(2236, 3057)	(3476, 4575)	(2203, 5502)
Cook Islands	13.1	8.9	3.9	7	4	1
	(9.6, 16.6)	(6.0, 11.8)	(2.1, 7.0)	(5, 9)	(3, 5)	(1, 2)
Costa Rica	8.9	7.7	5.9	732	585	405
	(8.5, 9.3)	(7.3, 8.0)	(4.5, 7.6)	(700, 766)	(559, 613)	(310, 524)
Côte d'Ivoire	48.3	45.4	33.5	25704	30742	29743
	(43.4, 53.5)	(40.2, 50.8)	(25.1, 44.0)	(23067, 28472)	(27223, 34402)	(22324, 39097)
Croatia	8.1	5.6	2.9	438	250	112
	(7.7, 8.5)	(5.2, 5.9)	(2.5, 3.4)	(415, 461)	(236, 265)	(97, 128)
Cuba	6.8	4.1	2.2	1197	597	272
	(6.5, 7.0)	(4.0, 4.3)	(2.0, 2.4)	(1153, 1241)	(572, 623)	(252, 293)
Cyprus	5.4	3.1	1.6	79	39	20
	(4.0, 6.7)	(2.4, 3.7)	(1.2, 2.1)	(59, 98)	(30, 46)	(15, 27)
Czech Republic	7.2	2.7	1.7	919	238	186
	(7.0, 7.5)	(2.5, 2.9)	(1.6, 1.9)	(884, 955)	(224, 252)	(170, 203)
Democratic People's Republic of Korea	21.8	27	10	9163	11119	3525
	(14.9, 30.6)	(16.8, 40.3)	(5.9, 15.0)	(6284, 12875)	(6931, 16590)	(2077, 5261)
Democratic Republic of the Congo	41.8	38.8	28.9	67381	84582	98060
	(35.8, 48.6)	(33.4, 45.0)	(18.8, 43.4)	(57713, 78315)	(72759, 97944)	(63968, 147459)
Denmark	4.4	3.5	3.1	276	229	189
	(4.2, 4.6)	(3.3, 3.7)	(2.7, 3.7)	(261, 290)	(217, 241)	(162, 222)
Djibouti	49.6	44.2	32.4	1111	993	702
	(41.2, 59.5)	(36.2, 53.7)	(19.5, 51.7)	(923, 1332)	(813, 1204)	(422, 1120)
Dominica	10.6	11.3	27.3	16	13	30
	(9.4, 11.9)	(10.0, 12.8)	(21.6, 34.5)	(14, 18)	(11, 15)	(24, 38)
Dominican Republic	24.4	23.6	20.1	5222	5090	4286
	(22.2, 26.8)	(21.4, 25.8)	(14.6, 27.9)	(4748, 5742)	(4608, 5572)	(3107, 5961)
Ecuador	22.6	14.1	7.5	6951	4444	2464
	(19.3, 26.0)	(12.1, 16.4)	(7.0, 7.9)	(5947, 7994)	(3808, 5157)	(2315, 2618)
Egypt	33.3	22.5	11.6	64001	39866	29067

	(31.0, 35.8)	(20.8, 24.4)	(8.6, 15.7)	(59472, 68721)	(36876, 43240)	(21458, 39260)
El Salvador	22.8	14.7	7.1	3748	2141	827
	(20.1, 26.0)	(12.7, 16.7)	(4.5, 11.0)	(3301, 4274)	(1849, 2438)	(533, 1287)
Equatorial Guinea	48.2	44.6	30.9	869	1126	1314
	(38.1, 62.1)	(36.3, 55.4)	(17.2, 52.6)	(686, 1120)	(919, 1401)	(732, 2237)
Eritrea	33.8	26	17.8	4271	3144	2862
	(28.4, 39.2)	(22.4, 29.8)	(10.4, 30.0)	(3586, 4947)	(2702, 3601)	(1667, 4821)
Estonia	9.6	5.3	1.2	200	68	17
	(9.0, 10.2)	(4.9, 5.8)	(1.0, 1.6)	(187, 214)	(62, 74)	(14, 22)
Eswatini	21.9	26	17.4	790	900	675
	(18.1, 26.1)	(22.3, 30.4)	(10.5, 28.4)	(655, 942)	(771, 1052)	(409, 1103)
Ethiopia	59.5	48.7	28.9	136534	141752	94978
	(53.7, 65.7)	(44.1, 53.6)	(23.4, 36.1)	(123283, 150681)	(128561, 156073)	(76934, 118455)
Fiji	11.9	9	10.6	253	180	183
	(9.4, 15.1)	(8.1, 10.0)	(9.3, 12.1)	(201, 321)	(162, 199)	(160, 209)
Finland	3.8	2.7	1.8	245	157	110
	(3.6, 4.1)	(2.5, 2.9)	(1.7, 2.0)	(230, 261)	(147, 167)	(99, 122)
France	3.6	2.7	2.4	2696	2058	1858
	(3.5, 3.8)	(2.6, 2.8)	(2.3, 2.6)	(2587, 2811)	(1984, 2136)	(1713, 2010)
Gabon	31	28.8	21.5	1094	1185	1252
	(25.8, 37.0)	(23.9, 35.1)	(13.5, 33.2)	(912, 1307)	(984, 1446)	(788, 1934)
Gambia	49.9	40.7	27.6	2148	2280	2259
	(33.1, 71.6)	(25.1, 61.3)	(13.7, 52.6)	(1425, 3083)	(1405, 3431)	(1122, 4311)
Georgia	24.8	22.1	6.7	2258	1249	346
	(21.7, 28.4)	(19.1, 25.6)	(5.8, 8.0)	(1971, 2577)	(1083, 1450)	(301, 413)
Germany	3.4	2.8	2.2	2804	2075	1608
	(3.2, 3.5)	(2.7, 2.9)	(2.0, 2.5)	(2692, 2919)	(2003, 2148)	(1452, 1778)
Ghana	42.1	36.3	24.2	23960	24385	21313
	(38.5, 45.8)	(33.2, 39.5)	(19.1, 30.7)	(21895, 26077)	(22328, 26563)	(16802, 26999)
Greece	6.5	3.9	3	716	434	267
	(6.2, 6.7)	(3.8, 4.1)	(2.5, 3.6)	(688, 743)	(415, 453)	(224, 323)
Grenada	12	8.2	10.9	33	16	22
	(10.7, 13.3)	(7.2, 9.2)	(8.8, 13.4)	(30, 37)	(14, 18)	(17, 26)
Guatemala	28.5	21.2	12.9	10330	8762	5426
	(25.8, 31.4)	(19.0, 23.5)	(9.9, 16.6)	(9350, 11348)	(7858, 9717)	(4177, 7005)
Guinea	61.7	46.7	24.1	17796	17603	10897
	(54.9, 69.0)	(41.4, 52.4)	(17.8, 33.0)	(15838, 19888)	(15607, 19779)	(8029, 14905)
Guinea-Bissau	64.2	55	37.3	3014	2835	2495
	(55.2, 74.5)	(47.5, 63.3)	(24.3, 54.9)	(2594, 3500)	(2450, 3261)	(1628, 3674)
Guyana	30.8	26.9	18.9	655	523	299
	(26.9, 35.0)	(23.5, 31.0)	(12.1, 29.9)	(572, 743)	(457, 601)	(191, 474)
Haiti	38.9	30.1	28.3	10280	8017	7391
	(34.1, 43.8)	(26.3, 34.2)	(21.9, 37.0)	(9003, 11587)	(7002, 9103)	(5730, 9672)
Honduras	21.7	17.8	10	4157	3801	1996
	(19.1, 24.4)	(15.9, 19.9)	(6.8, 14.7)	(3661, 4682)	(3390, 4238)	(1356, 2914)

Hungary	11.2 (10.8, 11.5)	5.7 (5.5, 6.0)	2.4 (2.1, 2.8)	1391 (1346, 1438)	552 (528, 576)	211 (184, 241)
Iceland	3.4 (3.0, 3.8)	2.1 (1.8, 2.5)	1 (0.7, 1.4)	15 (13, 17)	9 (8, 10)	5 (3, 6)
India	57.4 (54.4, 60.6)	45.1 (42.6, 47.7)	24 (21.3, 26.8)	1572455 (1489883, 1659880)	1256685 (1186655, 1329493)	604726 (535195, 675041)
Indonesia	30.1 (28.0, 32.3)	22.3 (20.6, 24.0)	12.4 (9.4, 16.4)	140930 (131039, 151289)	102700 (95129, 110584)	60986 (46067, 80712)
Iran (Islamic Republic of)	26.1 (18.4, 32.1)	18.9 (15.6, 22.2)	9.1 (5.4, 15.0)	48105 (33851, 58977)	23487 (19362, 27562)	11700 (6967, 19293)
Iraq	26.1 (23.3, 29.1)	24.4 (22.0, 27.2)	17.1 (12.1, 24.4)	17317 (15452, 19273)	20441 (18432, 22786)	21376 (15098, 30479)
Ireland	4.7 (4.5, 5.0)	4 (3.8, 4.3)	2.2 (1.7, 2.8)	243 (228, 257)	233 (220, 246)	143 (113, 181)
Israel	6.2 (6.0, 6.5)	3.6 (3.4, 3.8)	2 (1.7, 2.3)	626 (600, 653)	457 (437, 478)	329 (288, 376)
Italy	6.4 (6.3, 6.6)	3.5 (3.4, 3.6)	2 (1.8, 2.2)	3589 (3496, 3685)	1846 (1791, 1899)	959 (858, 1068)
Jamaica	20.1 (15.5, 25.4)	17.3 (14.5, 20.3)	10.8 (5.9, 18.7)	1238 (955, 1562)	978 (823, 1151)	512 (279, 888)
Japan	2.5 (2.5, 2.6)	1.8 (1.7, 1.8)	0.9 (0.8, 0.9)	3285 (3173, 3400)	2055 (1982, 2127)	882 (818, 951)
Jordan	20.2 (18.3, 22.1)	16.7 (15.0, 18.6)	10.1 (6.7, 15.0)	2565 (2328, 2809)	2653 (2391, 2963)	2502 (1665, 3708)
Kazakhstan	22.2 (18.4, 26.6)	21 (15.9, 26.1)	5.3 (4.9, 5.8)	8502 (7027, 10165)	4997 (3788, 6229)	1989 (1813, 2175)
Kenya	29.7 (26.9, 32.8)	30.8 (27.9, 34.0)	20.9 (15.9, 27.6)	29434 (26610, 32465)	38542 (34947, 42558)	32051 (24429, 42328)
Kiribati	35.6 (27.5, 47.1)	29 (23.4, 35.6)	23 (12.7, 39.7)	94 (72, 124)	75 (60, 92)	75 (41, 129)
Kuwait	10.1 (9.5, 10.9)	6.5 (6.2, 6.9)	4.3 (3.7, 5.0)	457 (430, 493)	290 (274, 306)	280 (238, 327)
Kyrgyzstan	23.9 (20.0, 28.1)	21.6 (18.4, 24.8)	10.7 (8.9, 13.2)	3272 (2731, 3833)	2279 (1941, 2616)	1587 (1312, 1955)
Lao People's Democratic Republic	48.2 (42.9, 54.3)	40.1 (35.6, 45.2)	28.2 (19.0, 41.4)	8809 (7846, 9929)	6800 (6027, 7655)	4529 (3046, 6647)
Latvia	8.2 (7.7, 8.8)	7.2 (6.7, 7.8)	2.2 (1.7, 2.7)	297 (278, 316)	140 (129, 151)	43 (34, 53)
Lebanon	20.5 (17.8, 23.6)	11.8 (9.3, 14.5)	4.5 (2.1, 8.6)	1394 (1212, 1604)	733 (576, 900)	409 (189, 774)
Lesotho	38.6 (33.4, 43.9)	43.2 (38.2, 48.6)	37.9 (26.9, 52.2)	2205 (1904, 2506)	2553 (2260, 2871)	2331 (1654, 3209)
Liberia	58.4 (50.5, 67.2)	44.7 (39.1, 50.9)	25.1 (16.6, 37.6)	5548 (4797, 6382)	5272 (4610, 6009)	4046 (2666, 6051)
Libya	21	14.9	6.5	2739	1734	803

	(17.6, 25.1)	(13.5, 16.6)	(4.3, 10.0)	(2297, 3265)	(1569, 1928)	(525, 1233)
Lithuania	8 (7.5, 8.4)	4.6 (4.2, 5.0)	2 (1.7, 2.3)	435 (408, 461)	151 (139, 165)	62 (52, 72)
Luxembourg	4.1 (3.6, 4.7)	2.5 (2.1, 2.8)	1.7 (1.2, 2.2)	20 (17, 23)	13 (11, 15)	11 (8, 15)
Madagascar	39.8 (35.7, 44.6)	31.2 (27.8, 35.0)	18.4 (11.8, 28.2)	20537 (18407, 23000)	20338 (18075, 22813)	15509 (9974, 23761)
Malawi	49.9 (45.0, 54.9)	39.4 (35.3, 43.2)	22.7 (16.8, 30.1)	22179 (19995, 24406)	19805 (17770, 21744)	15386 (11429, 20469)
Malaysia	7.8 (6.6, 9.0)	5.3 (4.7, 5.9)	4.3 (3.8, 4.7)	3957 (3361, 4569)	2690 (2367, 3008)	2295 (2062, 2536)
Maldives	42.5 (35.9, 49.4)	25.4 (22.4, 29.0)	4.5 (3.3, 6.1)	388 (328, 451)	162 (143, 185)	34 (25, 46)
Mali	72.8 (64.9, 81.9)	56.8 (49.7, 64.5)	35.4 (19.3, 62.3)	30321 (27019, 34108)	30474 (26668, 34601)	27677 (15118, 48704)
Malta	7.6 (7.0, 8.2)	5.2 (4.7, 5.7)	4.4 (3.5, 5.4)	41 (38, 45)	23 (21, 26)	19 (15, 23)
Marshall Islands	19.5 (15.5, 24.5)	18.4 (14.9, 22.4)	15.7 (9.6, 25.1)	41 (33, 52)	39 (32, 47)	18 (11, 29)
Mauritania	46.1 (40.2, 53.0)	42.9 (36.3, 50.2)	33.8 (17.3, 64.3)	3834 (3341, 4400)	4467 (3781, 5227)	5032 (2575, 9571)
Mauritius	14.6 (13.7, 15.5)	12.2 (11.5, 13.0)	7.9 (6.3, 9.7)	324 (306, 344)	246 (231, 262)	105 (85, 130)
Mexico	22.3 (19.9, 24.9)	13 (11.8, 14.4)	7.6 (7.2, 8.0)	54436 (48683, 60696)	31952 (28882, 35269)	17431 (16536, 18310)
Micronesia (Federated States of)	25.6 (17.4, 36.4)	25 (14.3, 41.4)	16.5 (6.4, 42.4)	85 (57, 120)	81 (46, 133)	42 (16, 107)
Monaco	4.1 (3.0, 5.2)	2.7 (1.9, 3.6)	1.7 (1.0, 2.7)	1 (1, 2)	1 (1, 1)	1 (0, 1)
Mongolia	29.6 (25.6, 34.2)	24.6 (21.5, 28.2)	9.1 (5.8, 14.1)	2055 (1777, 2377)	1144 (999, 1307)	647 (409, 998)
Montenegro	10.6 (9.8, 11.5)	8.9 (8.1, 9.6)	2.3 (1.9, 2.8)	108 (100, 117)	75 (69, 81)	16 (13, 20)
Morocco	36.2 (32.9, 39.6)	27.4 (24.5, 30.5)	14.4 (11.3, 18.3)	26420 (24041, 28902)	17556 (15737, 19535)	10024 (7889, 12744)
Mozambique	58.1 (52.1, 65.1)	44 (39.6, 48.6)	26.9 (18.9, 38.0)	36233 (32486, 40587)	35945 (32319, 39654)	30780 (21697, 43477)
Myanmar	48.3 (42.0, 56.1)	37.9 (33.4, 42.7)	24.1 (17.3, 32.7)	53327 (46406, 61933)	42859 (37694, 48241)	22625 (16253, 30745)
Namibia	27.7 (24.5, 31.3)	22.9 (20.2, 26.5)	17.5 (11.3, 27.0)	1484 (1314, 1680)	1366 (1201, 1578)	1268 (822, 1961)
Nauru	29.5 (16.6, 50.4)	24.6 (20.3, 29.9)	20.5 (11.0, 36.6)	5 (3, 9)	5 (4, 6)	3 (2, 6)
Nepal	58.5	40.6	20.7	42500	30886	11842

	(53.9, 63.6)	(37.5, 44.1)	(16.7, 25.9)	(39146, 46163)	(28485, 33514)	(9549, 14790)
Netherlands	4.6 (4.4, 4.7)	3.8 (3.7, 4.0)	2.3 (2.1, 2.4)	879 (847, 912)	757 (730, 785)	407 (382, 432)
New Zealand	4.3 (4.1, 4.5)	3.5 (3.3, 3.7)	3 (2.4, 3.6)	247 (234, 261)	194 (184, 205)	182 (149, 222)
Nicaragua	23.2 (20.9, 25.7)	16.2 (14.4, 18.2)	7.1 (4.2, 11.9)	3447 (3115, 3818)	2190 (1942, 2455)	841 (503, 1412)
Niger	54.3 (48.8, 60.4)	43.1 (38.6, 48.0)	26 (19.9, 33.8)	24271 (21807, 26999)	26200 (23471, 29169)	26787 (20527, 34845)
Nigeria	50.3 (45.2, 55.7)	48.1 (43.6, 52.9)	32.9 (23.1, 47.1)	212140 (190646, 234670)	253992 (230322, 279638)	241021 (169116, 344954)
Niue	7.3 (4.6, 11.4)	12.2 (7.1, 20.1)	11.1 (4.5, 28.1)	0 (0, 1)	0 (0, 1)	0 (0, 1)
Norway	4 (3.7, 4.2)	2.7 (2.5, 2.9)	1.5 (1.2, 1.8)	231 (218, 246)	155 (146, 165)	95 (79, 114)
Oman	17.4 (14.7, 20.5)	7.3 (5.5, 9.2)	5.1 (3.2, 6.7)	1219 (1035, 1439)	407 (306, 510)	412 (259, 543)
Pakistan	63.6 (57.7, 69.4)	60.1 (55.5, 65.2)	44.2 (31.5, 61.5)	275664 (250073, 301126)	266267 (245692, 288695)	240808 (171585, 335294)
Palau	19 (13.8, 25.1)	14.2 (9.6, 19.1)	7.9 (3.6, 16.6)	6 (4, 8)	5 (3, 6)	2 (1, 5)
Panama	17 (13.5, 20.4)	14.5 (10.4, 18.5)	8.9 (4.4, 16.4)	1106 (877, 1323)	1028 (739, 1311)	698 (347, 1290)
Papua New Guinea	31.2 (26.5, 36.0)	29.8 (24.9, 35.6)	23.7 (13.0, 43.8)	4692 (3983, 5406)	5645 (4713, 6738)	5332 (2922, 9861)
Paraguay	22 (19.2, 25.1)	18 (14.5, 22.1)	11.1 (5.9, 21.3)	3114 (2715, 3551)	2574 (2084, 3167)	1562 (827, 3011)
Peru	28.1 (25.8, 30.5)	16.3 (14.6, 17.9)	7.3 (5.5, 10.1)	18532 (17051, 20139)	10152 (9145, 11147)	4473 (3358, 6120)
Philippines	19.3 (17.3, 21.3)	16.8 (15.2, 18.6)	13.6 (10.7, 17.6)	39514 (35465, 43587)	38815 (35037, 43053)	32836 (25816, 42349)
Poland	11.1 (10.8, 11.4)	5.7 (5.6, 5.9)	3 (2.8, 3.1)	6272 (6097, 6446)	2175 (2109, 2241)	1034 (991, 1080)
Portugal	7.2 (6.9, 7.6)	3.4 (3.2, 3.5)	2.1 (1.8, 2.4)	822 (784, 861)	380 (359, 402)	162 (138, 187)
Qatar	11.3 (10.0, 12.7)	6.6 (6.1, 7.2)	3.8 (3.3, 4.3)	119 (106, 133)	78 (72, 85)	99 (86, 112)
Republic of Korea	7.2 (6.2, 8.4)	3.4 (3.1, 3.7)	1.5 (1.4, 1.7)	4800 (4140, 5595)	1950 (1780, 2123)	695 (621, 773)
Republic of Moldova	18.6 (15.7, 22.0)	20.8 (17.1, 24.9)	11.6 (8.5, 15.8)	1503 (1269, 1777)	993 (817, 1190)	473 (346, 646)
Romania	15 (13.6, 17.3)	10.5 (9.7, 11.5)	3.7 (3.1, 4.2)	4792 (4340, 5496)	2335 (2153, 2548)	688 (592, 783)
Russian Federation	10.9 (9.3, 12.9)	9.5 (8.3, 10.7)	3.3 (2.5, 4.2)	21850 (18714, 25743)	12564 (11043, 14244)	6013 (4530, 7562)

Rwanda	40.1	38.8	16.4	12669	11931	6104
	(36.0, 44.1)	(34.4, 43.7)	(9.9, 27.3)	(11372, 13942)	(10563, 13425)	(3694, 10154)
Saint Kitts and Nevis	19.6	14.4	8.8	18	13	7
	(17.3, 22.4)	(12.4, 16.5)	(6.2, 12.5)	(16, 21)	(11, 15)	(5, 11)
Saint Lucia	12.5	11.3	12.4	48	33	26
	(11.4, 13.6)	(10.3, 12.5)	(9.5, 16.1)	(44, 53)	(30, 36)	(20, 35)
Saint Vincent and the Grenadines	12.4	13.3	9.6	33	29	16
	(11.0, 13.7)	(12.1, 14.7)	(7.4, 12.3)	(29, 37)	(26, 32)	(12, 21)
Samoa	16.1	11.5	8.6	87	62	41
	(11.8, 20.8)	(7.8, 15.5)	(4.7, 14.1)	(64, 113)	(42, 83)	(22, 67)
San Marino	5.2	2.8	1	1	1	0
	(3.6, 7.6)	(2.0, 4.0)	(0.4, 2.6)	(1, 2)	(1, 1)	(0, 1)
Sao Tome and Principe	26.6	22.8	14.4	122	127	98
	(20.6, 33.1)	(17.2, 28.3)	(8.7, 24.2)	(94, 151)	(96, 157)	(59, 165)
Saudi Arabia	22.2	11.6	3.9	12899	6397	2446
	(15.4, 30.4)	(7.8, 15.3)	(3.0, 13.8)	(8914, 17629)	(4336, 8455)	(1898, 8677)
Senegal	39.8	38.2	20.5	12967	14879	11368
	(36.8, 43.1)	(34.9, 41.7)	(16.1, 26.3)	(11996, 14069)	(13580, 16206)	(8903, 14569)
Serbia	17.3	7.7	3.7	2470	911	339
	(16.0, 18.5)	(7.3, 8.1)	(3.3, 4.1)	(2280, 2636)	(865, 954)	(302, 381)
Seychelles	11.4	8.6	8.6	19	13	13
	(10.2, 12.8)	(7.6, 9.8)	(6.5, 11.0)	(17, 21)	(12, 15)	(10, 16)
Sierra Leone	53.2	50.5	33.5	10462	10798	8730
	(46.0, 61.3)	(44.6, 57.2)	(26.1, 42.1)	(9037, 12036)	(9550, 12240)	(6803, 10965)
Singapore	4	1.6	1.1	220	81	52
	(3.8, 4.3)	(1.5, 1.8)	(0.9, 1.3)	(207, 234)	(74, 88)	(43, 64)
Slovakia	8.7	5.1	2.8	693	272	156
	(8.4, 9.0)	(4.8, 5.3)	(2.5, 3.0)	(670, 717)	(259, 284)	(144, 168)
Slovenia	5.8	3.3	1.2	130	57	24
	(5.4, 6.1)	(3.0, 3.6)	(1.0, 1.4)	(122, 138)	(53, 62)	(20, 29)
Solomon Islands	15.3	12.7	8.5	191	186	146
	(12.2, 18.7)	(10.8, 15.0)	(5.5, 12.5)	(153, 234)	(158, 221)	(94, 216)
Somalia	45	44.4	38.5	15879	19484	24498
	(34.7, 58.1)	(32.8, 60.1)	(18.2, 80.3)	(12252, 20501)	(14389, 26360)	(11599, 51034)
South Africa	20.9	17.8	10.7	22871	19792	12572
	(17.2, 25.0)	(14.7, 22.2)	(8.3, 13.6)	(18851, 27368)	(16290, 24624)	(9756, 15962)
South Sudan	65.8	57.2	39.6	17530	16360	17698
	(46.2, 87.9)	(42.7, 75.2)	(16.4, 81.9)	(12319, 23415)	(12215, 21496)	(7353, 36589)
Spain	4.9	2.8	1.7	1991	1124	699
	(4.7, 5.1)	(2.7, 2.9)	(1.5, 2.0)	(1915, 2068)	(1077, 1170)	(591, 814)
Sri Lanka	12.9	9.6	5.8	4609	3326	1830
	(12.3, 13.5)	(9.2, 10.0)	(5.0, 6.9)	(4393, 4830)	(3185, 3467)	(1554, 2152)
State of Palestine	21.9	16.2	11.3	2136	1954	1746

	(19.4, 24.5)	(14.5, 18.0)	(8.2, 15.7)	(1894, 2387)	(1752, 2174)	(1264, 2428)
Sudan	42.6 (38.0, 47.5)	37.2 (32.9, 41.5)	29.5 (22.5, 38.8)	36237 (32326, 40395)	40245 (35646, 44883)	38975 (29676, 51273)
Suriname	23.3 (16.5, 31.2)	18.2 (11.8, 26.1)	10.2 (4.4, 22.8)	261 (186, 349)	200 (130, 286)	103 (44, 230)
Sweden	3.5 (3.3, 3.7)	2.3 (2.2, 2.4)	1.7 (1.6, 1.8)	407 (386, 427)	211 (200, 223)	205 (189, 224)
Switzerland	3.9 (3.7, 4.1)	3.5 (3.3, 3.6)	3 (2.7, 3.3)	315 (299, 332)	265 (252, 278)	263 (238, 289)
Syrian Arab Republic	17 (15.0, 19.1)	12.4 (11.0, 14.0)	8.7 (6.1, 12.9)	7581 (6698, 8526)	6334 (5590, 7129)	3475 (2418, 5123)
Tajikistan	31.5 (26.4, 37.8)	28.4 (23.9, 33.5)	14.5 (10.2, 20.3)	6667 (5603, 8003)	5349 (4504, 6305)	3653 (2561, 5105)
Thailand	20.4 (16.3, 23.4)	12.1 (8.4, 14.8)	5.3 (3.1, 7.4)	22097 (17608, 25402)	11040 (7673, 13552)	3681 (2180, 5154)
The former Yugoslav Republic of Macedonia	17.4 (16.3, 18.5)	9.2 (8.6, 9.8)	10.5 (9.0, 12.1)	614 (576, 653)	243 (227, 259)	245 (210, 284)
Timor-Leste	55 (46.7, 65.2)	36.8 (31.8, 42.5)	20.7 (11.7, 34.6)	1821 (1545, 2158)	1450 (1253, 1671)	930 (526, 1553)
Togo	43.2 (38.6, 48.5)	36.1 (32.0, 40.6)	25.4 (18.8, 34.1)	6892 (6157, 7725)	7067 (6260, 7942)	6645 (4906, 8925)
Tonga	10.3 (7.8, 13.7)	7.6 (5.9, 9.6)	6.7 (3.5, 11.9)	31 (23, 41)	21 (16, 27)	17 (9, 30)
Trinidad and Tobago	21 (17.0, 25.1)	21 (16.6, 26.3)	16.6 (9.0, 31.1)	532 (431, 636)	393 (311, 492)	301 (162, 563)
Tunisia	27.4 (23.6, 32.0)	18 (15.2, 21.7)	7.5 (4.9, 11.1)	5869 (5057, 6838)	3022 (2544, 3636)	1535 (1013, 2275)
Turkey	33.8 (30.2, 37.8)	19 (16.7, 21.6)	5.9 (5.2, 6.7)	47092 (42162, 52659)	25995 (22950, 29599)	7571 (6624, 8643)
Turkmenistan	28.2 (23.5, 33.4)	29.9 (23.4, 38.1)	21.3 (8.5, 47.2)	3699 (3090, 4380)	3189 (2500, 4069)	3009 (1206, 6661)
Tuvalu	29.7 (23.9, 36.7)	25 (21.6, 28.7)	16 (8.9, 29.0)	7 (6, 9)	7 (6, 8)	4 (2, 7)
Uganda	40.1 (35.9, 44.7)	29.4 (25.9, 33.0)	20.2 (15.6, 25.9)	34908 (31329, 38920)	34725 (30644, 38980)	36056 (27889, 46361)
Ukraine	11.8 (10.0, 14.0)	11 (9.3, 13.0)	5.2 (3.4, 6.5)	7613 (6446, 9038)	4460 (3757, 5248)	2417 (1602, 3039)
United Arab Emirates	8.4 (6.9, 10.2)	5.9 (4.6, 7.7)	4.7 (2.9, 7.0)	405 (333, 493)	312 (243, 412)	422 (258, 624)
United Kingdom	4.5 (4.3, 4.6)	3.8 (3.7, 3.9)	2.6 (2.4, 2.8)	3498 (3383, 3609)	2627 (2550, 2706)	2121 (1969, 2284)
United Republic of Tanzania	37.8 (34.1, 41.5)	32.7 (29.7, 36.0)	21.1 (16.2, 27.5)	42657 (38527, 46859)	47152 (42812, 51864)	45691 (35160, 59477)
United States	5.8 (5.6, 5.9)	4.6 (4.5, 4.7)	3.6 (3.5, 3.8)	22793 (22146, 23436)	18287 (17800, 18767)	14839 (14093, 15632)

Uruguay	11.8<br>(11.0, 12.7)	8.3<br>(7.9, 8.7)	4.6<br>(4.3, 5.0)	672<br>(626, 721)	448<br>(426, 471)	222<br>(206, 240)
Uzbekistan	31<br>(26.0, 36.4)	28.4<br>(23.7, 34.1)	12.1<br>(8.2, 16.6)	22102<br>(18533, 25969)	16067<br>(13407, 19257)	7882<br>(5364, 10831)
Vanuatu	16.3<br>(12.7, 21.6)	12.3<br>(9.6, 15.4)	11.6<br>(6.7, 19.4)	87<br>(67, 115)	75<br>(58, 93)	82<br>(47, 137)
Venezuela (Bolivarian Republic of)	13<br>(12.4, 13.5)	10.9<br>(10.5, 11.3)	19.8<br>(18.4, 21.4)	7464<br>(7160, 7777)	6331<br>(6113, 6554)	11875<br>(10993, 12819)
Viet Nam	23.7<br>(21.0, 27.0)	14.8<br>(12.1, 17.6)	10.6<br>(8.1, 14.1)	46431<br>(41196, 52935)	20878<br>(16986, 24859)	16666<br>(12795, 22140)
Yemen	43.6<br>(39.6, 47.6)	37.2<br>(33.5, 41.2)	27<br>(18.1, 39.0)	27804<br>(25302, 30399)	26260<br>(23690, 29072)	23657<br>(15900, 34194)
Zambia	37.4<br>(33.7, 41.2)	37.2<br>(33.5, 41.4)	22.2<br>(15.3, 31.4)	13544<br>(12222, 14930)	17550<br>(15811, 19515)	14378<br>(9888, 20333)
Zimbabwe	25.5<br>(22.7, 28.6)	27.8<br>(24.7, 31.2)	22.4<br>(15.6, 30.8)	9655<br>(8607, 10830)	11320<br>(10087, 12718)	11932<br>(8321, 16431)

Table A.6: Series in NMR database by country or territory
Note: The series name refers to a specific data collection source and estimation method. There are several sources including vital registration data (including sample vital registration data) and sample surveys – where: vital registration series are based on birth and death registration data and survey series are based on data on births and deaths from full birth histories

Country or territory	Data series	Inclusion
Afghanistan	Multiple Indicator Cluster Survey 2003	0
	Afghanistan Mortality Survey (AMS) (NN adjusted) 2010	1
	Afghanistan Mortality Survey (AMS) 2010	0
	Demographic and Health Survey (NN adjusted) 2015	1
	Demographic and Health Survey 2015	0
	Reproductive Health Survey 2002	1
	Demographic and Health Survey 2008-2009	1
Albania	Vital Registration data submitted to WHO/UN IGME version 2018	1
	PAPCHILD Maternal and Child Health Survey 1992	1
Algeria	PAPFAM Family Health Survey 2002	1
	Multiple Indicator Cluster Survey 2012-2013	1
Andorra	Vital Registration data submitted to WHO/UN IGME version 2018	1
Angola	Malaria Indicator Survey 2011	0
	Inquerito de Indicadores Multiplos e de Saude 2015-2016	1
Antigua and Barbuda	Vital Registration data submitted to WHO/UN IGME version 2018	1
Argentina	Vital Registration data submitted to WHO/UN IGME version 2018	1
	Demographic and Health Survey 2000	1
	Demographic and Health Survey 2005	1
	Demographic and Health Survey 2010	1

	Demographic and Health Survey 2015-2016	0
	Vital Registration data from The Demographic Handbook of Armenia 2016 2016	0
Armenia	Vital Registration data submitted to WHO/UN IGME version 2018	0
Australia	Vital Registration data submitted to WHO/UN IGME version 2018	1
Austria	Vital Registration data submitted to WHO/UN IGME version 2018	1
	Reproductive Health Survey 2001	1
	Demographic and Health Survey 2006	1
	Azerbaijan National DHS 2011	1
Azerbaijan	Vital Registration data submitted to WHO/UN IGME version 2018	0
	VR from the State Statistical Committee 2018	0
	Adjusted National VR data 2018	1
Bahamas	Vital Registration data submitted to WHO/UN IGME version 2018	1
	Child Health Survey 1989	1
	Gulf Family Health Survey 1995	1
Bahrain	Vital Registration data submitted to WHO/UN IGME version 2018	1
Bangladesh	World Fertility Survey 1975-1976	1
	Demographic and Health Survey 1993-1994	1
	Demographic and Health Survey 1996-1997	1
	Demographic and Health Survey 1999-2000	1
	Maternal Health Services and Maternal Mortality Survey 2001	1
	Demographic and Health Survey 2004	1
	Demographic and Health Survey 2007	1
	Demographic and Health Survey 2011	1
	Demographic and Health Survey 2014	1
	SVR from Report on Sample Vital Registration System 2016	1
Barbados	Vital Registration data submitted to WHO/UN IGME version 2018	1
Belarus	Vital Registration data submitted to WHO/UN IGME version 2018	1
Belgium	Vital Registration data submitted to WHO/UN IGME version 2018	1
	Family Health Survey 1991	1
	Maternal and Child Mortality Survey 1996	0
	Multiple Indicator Cluster Survey 2015-2016	0
Belize	Vital Registration data submitted to WHO/UN IGME version 2018	1
Benin	World Fertility Survey 1981-1982	1
	Demographic and Health Survey 1996	1
	Demographic and Health Survey 2001	1
	Demographic and Health Survey 2006	1
	Demographic and Health Survey 2011-2012	0
	Multiple Indicator Cluster Survey 2014	1
	National Health Survey 2000	1
Bhutan	National Health Survey 2012	1
	Demographic and Health Survey 1989	1

	Demographic and Health Survey 1994	1
	Demographic and Health Survey 1998	1
	Demographic and Health Survey 2003	1
Bolivia (Plurinational State of)	Demographic and Health Survey 2008	1
Bosnia and Herzegovina	Vital Registration data submitted to WHO/UN IGME version 2018	1
	Family Health Survey 1988	1
Botswana	Family Health Survey 2007	1
	Demographic and Health Survey 1986	1
	Demographic and Health Survey 1996	1
Brazil	Vital Registration data submitted to WHO/UN IGME version 2018	0
	Data from Information System of Ministry of Health (Busca Ativa) 2018	1
Brunei Darussalam	Vital Registration data submitted to WHO/UN IGME version 2018	1
Bulgaria	Vital Registration data submitted to WHO/UN IGME version 2018	1
	Demographic and Health Survey 1993	1
	Demographic and Health Survey 1998-1999	1
	Demographic and Health Survey 2003	1
	Demographic and Health Survey 2010	1
Burkina Faso	Enquete Multisectorielle Continue (EMC) (Preliminary) 2015	1
	Demographic and Health Survey 1987	1
	Demographic and Health Survey 2011	1
Burundi	Demographic and Health Survey 2016-2017	1
Côte d'Ivoire	World Fertility Survey 1980-1981	1
	Demographic and Health Survey 1994	1
	Demographic and Health Survey 1998-1999	1
	AIDS Indicator Survey 2005	1
	Demographic and Health Survey 2011-2012	0
	Multiple Indicator Cluster Survey 2016	1
	Demographic and Reproductive Health Survey 1998	1
	Demographic and Health Survey 2005	1
Cabo Verde	Vital Registration data submitted to WHO/UN IGME version 2018	1
Cambodia	National Health Survey 1998	0
	Demographic and Health Survey 2000	1
	Demographic and Health Survey 2005	1
	Demographic and Health Survey 2010	1
	Demographic and Health Survey 2014	1
Cameroon	World Fertility Survey 1978	1
	Demographic and Health Survey 1991	1
	Demographic and Health Survey 1998	1
	Demographic and Health Survey 2004	1
	Demographic and Health Survey 2011	1

	Multiple Indicator Cluster Surveys 2014	1
Canada	Vital Registration data submitted to WHO/UN IGME version 2018	1
Central African Republic	Demographic and Health Survey 1994-1995	1
	Demographic and Health Survey 1996-1997	1
	Demographic and Health Survey 2004	1
Chad	Demographic and Health Survey 2014-2015	1
Chile	Vital Registration data submitted to WHO/UN IGME version 2018	1
China	NHFPC, Death Registration Data 2017	1
Colombia	World Fertility Survey 1976	1
	Demographic and Health Survey 1986	1
	Demographic and Health Survey 1990	1
	Demographic and Health Survey 1995	1
	Demographic and Health Survey 2000	1
	Demographic and Health Survey 2005	1
	Demographic and Health Survey 2010	1
	Vital Registration data submitted to WHO/UN IGME version 2018	0
	Demographic and Health Survey 1996	1
Comoros	Demographic and Health Survey 2012	0
	Demographic and Health Survey 2005	1
	Demographic and Health Survey 2011-2012	1
Congo	Multiple Indicator Cluster Survey 2014-2015	1
Cook Islands	Vital Statistics Report 1999-2013	0
Costa Rica	World Fertility Survey 1976	0
	Vital Registration data submitted to WHO/UN IGME version 2018	1
Croatia	Vital Registration data submitted to WHO/UN IGME version 2018	1
Cuba	Vital Registration data submitted to WHO/UN IGME version 2018	1
Cyprus	Vital Registration data submitted to WHO/UN IGME version 2018	1
Czechia	Vital Registration data submitted to WHO/UN IGME version 2018	1
Democratic People's Republic of Korea	Multiple Indicator Cluster Survey 2017	0
	Demographic and Health Survey 2007	1
Democratic Republic of the Congo	Demographic and Health Survey 2013-2014	1
Denmark	Vital Registration data submitted to WHO/UN IGME version 2018	1
	PAPFAM Family Health Survey 2002	1
Djibouti	PAPFAM Family Health Survey 2012	1
Dominica	Vital Registration data submitted to WHO/UN IGME version 2018	1
	Demographic and Health Survey 1986	1
	Demographic and Health Survey 1991	1
	Demographic and Health Survey 1996	1
	Demographic and Health Survey 1999	0
	Demographic and Health Survey 2002	1

	Multiple Indicator Cluster Survey 2006	1
	Demographic and Health Survey 2007	1
	Demographic and Health Survey 2013	1
	Multiple Indicator Cluster Survey 2014	1
Dominican Republic	Vital Registration data submitted to WHO/UN IGME version 2018	0
Ecuador	World Fertility Survey 1979-1980	1
	Demographic and Family Health Survey 1987	1
	Demographic and Maternal and Child Health Survey 1989	1
	Demographic and Maternal and Child Health Survey 1994	0
	Demographic and Maternal and Child Health Survey 1999	1
	Demographic and Maternal and Child Health Survey 2004	1
	Encuesta Nacional de Salud y Nutricion, Imputed birth and deaths 2012	1
	Encuesta Nacional de Salud y Nutricion 2012	0
	Vital Registration data submitted to WHO/UN IGME version 2018	0
	Adjusted Vital Registration data (Live births) 2018	1
Egypt	World Fertility Survey 1980	1
	Demographic and Health Survey 1988	1
	PAPCHILD Maternal and Child Health Survey 1991	1
	Demographic and Health Survey 1992	1
	Demographic and Health Survey 1995	1
	Demographic and Health Survey 1997	1
	Demographic and Health Survey 1998	1
	Demographic and Health Survey 2000	1
	Demographic and Health Survey 2003	1
	Demographic and Health Survey 2005	1
	Demographic and Health Survey 2008	1
	Demographic and Health Survey 2014	1
	Vital Registration data submitted to WHO/UN IGME version 2018	0
	National Family Health Survey 1985	0
	National Family Health Survey 1988	1
	National Family Health Survey 1993	1
	National Family Health Survey 1998	1
	National Family Health Survey 2003	1
	National Family Health Survey 2008	1
	Multiple Indicator Cluster Survey 2014	1
	Vital Registration data from Informe de Labores Ministerio de Salud 2015-2016 2015-2016	0
El Salvador	Vital Registration data submitted to WHO/UN IGME version 2018	0
Equatorial Guinea	Demographic and Health Survey 2011	1
	Demographic and Health Survey 1996	1
	Demographic and Health Survey 2002	1
Eritrea	Population and Health Survey 2010	1

Estonia	Vital Registration data submitted to WHO/UN IGME version 2018	1
	Demographic and Health Survey 2006-2007	1
	Multiple Indicator Cluster Survey 2010	1
Eswatini	Multiple Indicator Cluster Survey 2014	1
	Demographic and Health Survey 2000	1
	Demographic and Health Survey 2005	1
	Demographic and Health Survey 2011	1
Ethiopia	Demographic and Health Survey 2016	1
Fiji	World Fertility Survey 1974	0
	Vital Registration data submitted to WHO/UN IGME version 2018	1
Finland	Vital Registration data submitted to WHO/UN IGME version 2018	1
France	Vital Registration data submitted to WHO/UN IGME version 2018	1
	Demographic and Health Survey 2000	1
Gabon	Demographic and Health Survey 2012	1
Gambia	Demographic and Health Survey 2013	0
	Reproductive Health Survey 2000	1
	Reproductive Health Survey 2005	1
	Reproductive Health Survey 2010	1
Georgia	Vital Registration data submitted to WHO/UN IGME version 2018	0
	Vital Statistics of the National Center for Disease Control and Public Health 2018	1
Germany	Vital Registration data submitted to WHO/UN IGME version 2018	1
Ghana	World Fertility Survey 1979-1980	0
	Demographic and Health Survey 1988	1
	Demographic and Health Survey 1994	1
	Demographic and Health Survey 1999	1
	Demographic and Health Survey 2003	1
	Maternal Health Survey 2007	1
	Demographic and Health Survey 2008	1
	Multiple Indicator Cluster Survey 2011	1
	Demographic and Health Survey 2014	1
	Maternal Health Survey 2017	1
Greece	Vital Registration data submitted to WHO/UN IGME version 2018	1
Grenada	Vital Registration data submitted to WHO/UN IGME version 2018	1
	Demographic and Health Survey 1987	1
	Demographic and Health Survey 1995	1
	Demographic and Health Survey 1998-1999	1
	Reproductive Health Survey 2002	1
	Encuesta Nacional de Salud Materno Infantil (ENSMI) 2009	1
	Demographic and Health Survey 2014-2015	1
Guatemala	Vital Registration data submitted to WHO/UN IGME version 2018	0
	Demographic and Health Survey 1992	1

	Demographic and Health Survey 1999	1
	Demographic and Health Survey 2005	1
	Demographic and Health Survey 2012	1
Guinea	Multiple Indicator Cluster Survey 2016	1
	Multiple Indicator Cluster Survey 2010	1
Guinea-Bissau	Multiple Indicator Cluster Survey 2014	1
Guyana	World Fertility Survey 1975	1
	AIDS Indicator Survey 2005	1
	Demographic and Health Survey 2009	1
	Multiple Indicator Cluster Survey 2014	1
	Vital Registration data submitted to WHO/UN IGME version 2018	0
Haiti	World Fertility Survey 1977	1
	Mortality, morbidity and service utilization survey 1987	1
	Demographic and Health Survey 1994-1995	1
	Demographic and Health Survey 2000	1
	Demographic and Health Survey 2005-2006	1
	Demographic and Health Survey 2012	1
	Demographic and Health Survey (Preliminary) 2016-2017	1
	National Survey of Epidemiology and Family Health 1987	1
	National Survey of Epidemiology and Family Health 1996	1
	National Survey of Epidemiology and Family Health 2001	1
	Demographic and Health Survey 2005-2006	1
	Demographic and Health Survey 2011-2012	1
Honduras	Vital Registration data submitted to WHO/UN IGME version 2018	0
Hungary	Vital Registration data submitted to WHO/UN IGME version 2018	1
Iceland	Vital Registration data submitted to WHO/UN IGME version 2018	1
	National Family Health Survey 1992-1993	1
	National Family Health Survey 1998-1999	1
	National Family Health Survey 2005-2006	1
	National Family Health Survey 2015-2016	0
India	Sample Registration System 2016	1
Indonesia	World Fertility Survey 1976	1
	Demographic and Health Survey 1987	1
	Demographic and Health Survey 1991	1
	Demographic and Health Survey 1994	1
	Demographic and Health Survey 1997	1
	Demographic and Health Survey 2002-2003	1
	Demographic and Health Survey 2007	1
	Demographic and Health Survey 2012	1
	SVR from Report on Sample Vital Registration System 2014 2014	0
	Demographic and Health Survey 2000	1

Iran (Islamic Republic of)	National Multiple-Indicator Demographic and Health Survey 2010	1
	Child and Maternal Mortality Survey 1999	0
	Living Conditions Survey 2004	1
	Multiple Indicator Cluster Survey 2006	1
	Multiple Indicator Cluster Survey 2011	1
Iraq	Vital Registration data submitted to WHO/UN IGME version 2018	0
Ireland	Vital Registration data submitted to WHO/UN IGME version 2018	1
Israel	Vital Registration data submitted to WHO/UN IGME version 2018	1
Italy	Vital Registration data submitted to WHO/UN IGME version 2018	1
Jamaica	World Fertility Survey 1975-1976	1
	Reproductive Health Survey 2009	1
	Vital Registration data submitted to WHO/UN IGME version 2018	0
Japan	Vital Registration data submitted to WHO/UN IGME version 2018	1
Jordan	World Fertility Survey 1976	1
	Demographic and Health Survey 1990	1
	Living conditions survey 1996	0
	Demographic and Health Survey 1997	1
	Demographic and Health Survey 2002	1
	Demographic and Health Survey 2007	1
	Demographic and Health Survey 2009	1
	Demographic and Health Survey 2012	1
	Demographic and Health Survey (Preliminary) 2017	0
	Vital Registration data submitted to WHO/UN IGME version 2018	0
	Demographic and Health Survey 1995	1
	Demographic and Health Survey 1999	1
Kazakhstan	Vital Registration data submitted to WHO/UN IGME version 2018	1
Kenya	World Fertility Survey 1977-1978	1
	Demographic and Health Survey 1989	1
	Demographic and Health Survey 1993	1
	Demographic and Health Survey 1998	1
	Demographic and Health Survey 2003	1
	Demographic and Health Survey 2008-2009	1
	Demographic and Health Survey 2014	1
Kiribati	SPC Demographic and Health Survey 2009	1
Kuwait	Vital Registration data submitted to WHO/UN IGME version 2018	1
	Demographic and Health Survey 1997	1
	Demographic and Health Survey 2012	1
	Multiple Indicator Cluster Survey 2014	1
Kyrgyzstan	Vital Registration data submitted to WHO/UN IGME version 2018	0
	Reproductive Health Survey 2000	1

Lao People's Democratic Republic	Reproductive Health Survey 2005	1
	Lao Social Indicator Survey (combined MICS4/DHS) 2012	1
Latvia	Vital Registration data submitted to WHO/UN IGME version 2018	1
	PAPCHILD Maternal and Child Health Survey 1996	1
	PAPFAM Family Health Survey 2004	1
Lebanon	Vital Registration data submitted to WHO/UN IGME version 2018	0
Lesotho	World Fertility Survey 1977	1
	Demographic and Health Survey 2004	1
	Demographic and Health Survey 2009	1
	Demographic and Health Survey 2014	1
	Demographic and Health Survey 1986	1
	Demographic and Health Survey 2007	1
Liberia	Malaria Indicator Survey 2009	1
	Demographic and Health Survey 2013	1
	PAPCHILD Maternal and Child Health Survey 1995	1
Libya	PAPFAM Family Health Survey 2007	1
Lithuania	Vital Registration data submitted to WHO/UN IGME version 2018	1
Luxembourg	Vital Registration data submitted to WHO/UN IGME version 2018	1
	Demographic and Health Survey 1992	1
	Demographic and Health Survey 1997	1
	Demographic and Health Survey 2003-2004	1
	Demographic and Health Survey 2008-2009	1
Madagascar	National Survey on Monitoring the MDGs (ENSOMD) 2012-2013	0
	Demographic and Health Survey 1992	1
	Demographic and Health Survey 2000	1
	Demographic and Health Survey 2004	1
Malawi	Multiple Indicator Cluster Survey 2006	1
	Demographic and Health Survey 2010	1
	MDG Endline Survey 2013-2014	1
	Demographic and Health Survey 2015-2016	1
Malaysia	World Fertility Survey 1974	1
	Vital Statistics 2017	0
	Vital Registration data submitted to WHO/UN IGME version 2018	1
	Demographic and Health Survey 2009	1
Maldives	Vital Registration data submitted to WHO/UN IGME version 2018	1
	Demographic and Health Survey 1987	1
	Demographic and Health Survey 1995-1996	1
	Demographic and Health Survey 2001	1
	Demographic and Health Survey 2006	1
	Demographic and Health Survey 2012-2013	0
Mali	Multiple Indicator Cluster Survey 2015	0

Malta	Vital Registration data submitted to WHO/UN IGME version 2018	1
	Demographic and Health Survey 2007	1
Marshall Islands	Vital Registration data submitted to WHO/UN IGME version 2018	0
Mauritania	World Fertility Survey 1981-1982	1
	PAPCHILD Maternal and Child Health Survey 1990	1
	Demographic and Health Survey 2001	1
	EMIP survey 2003-2004	1
	Multiple Indicator Cluster Survey 2011	0
	Multiple Indicator Cluster Survey 2015	0
Mauritius	Vital Registration data submitted to WHO/UN IGME version 2018	1
Mexico	World Fertility Survey 1976	1
	Demographic and Health Survey 1987	1
	Encuesta Nacional de la Dinamica Demografica (ENADID) 1992	1
	Encuesta Nacional de la Dinamica Demografica (ENADID) 1997	1
	Encuesta Nacional de la Dinamica Demografica (ENADID) 2009	0
	Encuesta Nacional de la Dinamica Demografica (ENADID) 2014	1
	Vital Registration data submitted to WHO/UN IGME version 2018	0
	Vital Registration data from Mexico Ministry of Health 2018	1
	Reproductive Health Survey 1998	1
	Reproductive Health Survey 2003	0
	Reproductive Health Survey 2008	0
	Social Indicator Sample Survey (SISS) 2013-2014	1
Mongolia	Vital Registration data submitted to WHO/UN IGME version 2018	0
Montenegro	Vital Registration data submitted to WHO/UN IGME version 2018	1
Morocco	World Fertility Survey 1980	1
	Demographic and Health Survey 1987	1
	Demographic and Health Survey 1992	1
	Demographic and Health Survey 1995	1
	PAPG ENSME grand-echantillon 1996 1996	0
	PAPCHILD Maternal and Child Health Survey 1997	1
	Demographic and Health Survey 2003-2004	1
	National Survey on Population and Family Health 2011	1
	Vital Registration data submitted to WHO/UN IGME version 2018	0
	National Survey on Population and Family Health 2018	1
	Demographic and Health Survey 1997	0
	Demographic and Health Survey 2003-2004	1
Mozambique	Multiple Indicator Cluster Survey 2008	1
	Demographic and Health Survey 2011	1
	Fertility and Reproductive Health Survey 2001	1
Myanmar	Fertility and Reproductive Health Survey 2007	1
	Demographic and Health Survey 2015-2016	1

	Demographic and Health Survey 1992	1
	Demographic and Health Survey 2000	1
	Demographic and Health Survey 2006-2007	1
Namibia	Demographic and Health Survey 2013	1
	Demographic and Health Survey 2007	1
Nauru	Vital Statistics Report 2008-2013	0
Nepal	World Fertility Survey 1976	1
	Demographic and Health Survey 1996	1
	Demographic and Health Survey 2001	1
	Demographic and Health Survey 2006	1
	Demographic and Health Survey 2011	1
	Multiple Indicator Cluster Survey 2014	1
	Demographic and Health Survey 2016	1
Netherlands	Vital Registration data submitted to WHO/UN IGME version 2018	1
New Zealand	Vital Registration data submitted to WHO/UN IGME version 2018	1
	Family Health Survey 1993	1
	Demographic and Health Survey 1998	1
	Demographic and Health Survey 2001	1
	Nicaraguense de Demografia y Salud (ENDESA) 2006-2007	1
	Encuesta Nicaraguense de Demografia y Salud 2011-2012	1
Nicaragua	Vital Registration data submitted to WHO/UN IGME version 2018	0
	Demographic and Health Survey 1992	1
	Demographic and Health Survey 1998	1
	Demographic and Health Survey 2006	1
	Child Survival and Mortality Survey New 2010	1
	Demographic and Health Survey 2012	1
Niger	Etude Nationale d'Evaluation des Indicateurs Socioeconomiques et Demographiques (Preliminary) 2015	0
	Enquete Demographique et de Sante 2017	1
Nigeria	World Fertility Survey 1981-1982	0
	Demographic and Health Survey 1990	1
	Demographic and Health Survey 1999	0
	Demographic and Health Survey 2003	1
	Demographic and Health Survey 2008	1
	Malaria Indicator Survey 2010	1
	Demographic and Health Survey 2013	1
	Multiple Indicator Cluster Survey 2016-2017	0
Norway	Vital Registration data submitted to WHO/UN IGME version 2018	1
	Child Health Survey 1988	1
	Family Health Survey 1995	1
	Multiple Indicator Cluster Survey 2014	0
Oman	Vital Registration data submitted to WHO/UN IGME version 2018	0

Pakistan	World Fertility Survey 1975	1
	Demographic Survey 1984	1
	Demographic Survey 1985	1
	Demographic Survey 1986	1
	Demographic Survey 1987	1
	Demographic Survey 1990	1
	Demographic and Health Survey 1990-1991	1
	Pakistan Fertility and Family Planning Survey 1996	1
	Reproductive Health and Family Planning Survey 2000-2001	1
	Demographic Survey 2003	1
	Demographic Survey 2005	1
	Demographic Survey 2006	1
	Demographic and Health Survey 2006-2007	1
	Demographic Survey 2007	1
	Demographic and Health Survey 2012-2013	1
Panama	World Fertility Survey 1976	1
	Vital Registration data submitted to WHO/UN IGME version 2018	0
	Demographic and Health Survey 1996	1
Papua New Guinea	Demographic and Health Survey 2006	1
	Demographic and Health Survey 1990	1
	National Survey of Demography and Reproductive Health 1996	1
Paraguay	Reproductive Health Survey 2004	1
	Encuesta Nacional de Demografia y Salud Sexual y Reproductiva 2008	1
	Multiple Indicator Cluster Survey 2016	0
	Ministerio de Salud Pulbica y Bienestar Social 2016	0
Peru	World Fertility Survey 1977-1978	1
	Demographic and Health Survey 1986	1
	Demographic and Health Survey 1991-1992	1
	Demographic and Health Survey 1996	1
	Demographic and Health Survey 2000	1
	Demographic and Health Survey 2008	1
	Demographic and Health Survey 2009	1
	Demographic and Health Survey 2010	1
	Demographic and Health Survey 2011	1
	Demographic and Health Survey 2012	1
	Demographic and Health Survey 2013	1
	National Demographic and Health Survey 2014	1
	Demographic and Family Health Survey 2017	1
	Vital Registration data submitted to WHO/UN IGME version 2018	0
Philippines	World Fertility Survey 1978	1
	Demographic and Health Survey 1993	1

	Demographic and Health Survey 1998	1
	Demographic and Health Survey 2003	1
	Demographic and Health Survey 2008	1
	National Demographic and Health Survey 2013	1
	National Demographic and Health Survey (Preliminary) 2017	1
	Vital Registration data submitted to WHO/UN IGME version 2018	0
Poland	Vital Registration data submitted to WHO/UN IGME version 2018	1
Portugal	World Fertility Survey 1979-1980	0
	Vital Registration data submitted to WHO/UN IGME version 2018	1
	Gulf Family Health Survey 1998	0
	Vital Statistics, Annual Bulletin 2015	0
Qatar	Vital Registration data submitted to WHO/UN IGME version 2018	1
Republic of Korea	World Fertility Survey 1974	1
	Vital Registration data submitted to WHO/UN IGME version 2018	1
	Demographic and Health Survey 2005	1
	Multiple Indicator Cluster Survey 2012	1
Republic of Moldova	Vital Registration data submitted to WHO/UN IGME version 2018	0
	Reproductive Health Survey 1999	0
Romania	Vital Registration data submitted to WHO/UN IGME version 2018	1
Russian Federation	Vital Registration data submitted to WHO/UN IGME version 2018	1
Rwanda	World Fertility Survey 1983	1
	Demographic and Health Survey 1992	1
	Demographic and Health Survey 2000	1
	Demographic and Health Survey 2005	1
	Interim Demographic and Health Survey 2007-2008	1
	Demographic and Health Survey 2010	1
	Demographic and Health Survey 2014-2015	1
Saint Kitts and Nevis	Vital Registration data submitted to WHO/UN IGME version 2018	1
Saint Lucia	Vital Registration data submitted to WHO/UN IGME version 2018	1
Saint Vincent and the Grenadines	Vital Registration data submitted to WHO/UN IGME version 2018	1
	Demographic and Health Survey 2009	0
Samoa	Demographic and Health Survey 2014	0
San Marino	Vital Registration data submitted to WHO/UN IGME version 2018	1
	Demographic and Health Survey 2008-2009	1
	Multiple Indicator Cluster Survey 2014	1
Sao Tome and Principe	Vital Registration data submitted to WHO/UN IGME version 2018	0
Saudi Arabia	Family Health Survey 1996	0
	Demographic Survey 2016	0
Senegal	World Fertility Survey 1978	1
	Demographic and Health Survey 1986	1

	Demographic and Health Survey 1992-1993	1
	Demographic and Health Survey 1997	1
	Demographic and Health Survey 2000	1
	Demographic and Health Survey 2005	1
	Malaria Indicator Survey 2008-2009	1
	Demographic and Health Survey 2010-2011	1
	Demographic and Health Survey 2012-2013	1
	Demographic and Health Survey 2014	1
	Demographic and Health Survey 2015	1
	Demographic and Health Survey 2016	1
Serbia	Vital Registration data submitted to WHO/UN IGME version 2018	1
Seychelles	Vital Registration data submitted to WHO/UN IGME version 2018	1
	Demographic and Health Survey 2008	0
Sierra Leone	Demographic and Health Survey 2013	1
Singapore	Vital Registration data submitted to WHO/UN IGME version 2018	1
Slovakia	Vital Registration data submitted to WHO/UN IGME version 2018	1
Slovenia	Vital Registration data submitted to WHO/UN IGME version 2018	1
	Demographic and Health Survey 2007	1
Solomon Islands	Demographic and Health Survey 2015	1
Somalia	Multiple Indicator Cluster Survey 2006	1
	Demographic and Health Survey 1998	1
	Demographic and Health Survey 2004	0
	Demographic and Health Survey (Preliminary) 2016	0
	Data from District Health Information System 2016	1
South Africa	Vital Registration data submitted to WHO/UN IGME version 2018	0
South Sudan	Sudan Household Health Survey 2006	1
	Household Health Survey 2010	0
Spain	Vital Registration data submitted to WHO/UN IGME version 2018	1
Sri Lanka	World Fertility Survey 1975	1
	Demographic and Health Survey 1987	1
	Demographic and Health Survey 1993	1
	Demographic and Health Survey 2000	1
	Demographic and Health Survey 2006	1
	Demographic and Health Survey 2016	1
	Vital Registration data submitted to WHO/UN IGME version 2018	1
	Demographic Survey 1995	1
	Health Survey 2000	1
	Demographic and Health Survey 2004	1
State of Palestine	PAPFAM Family Health Survey 2006	1
	Multiple Indicator Cluster Survey-Family Health Survey 2010	1
	Multiple Indicator Cluster Survey 2014	1

	Demographic and Health Survey 1990	1
	PAPCHILD Maternal and Child Health Survey 1992	1
	Safe Motherhood Survey 1999	1
Sudan	Sudan Household Health Survey 2006	1
	Household Health Survey 2010	0
	Multiple Indicator Cluster Survey 2014	1
Suriname	Vital Registration data submitted to WHO/UN IGME version 2018	0
Sweden	Vital Registration data submitted to WHO/UN IGME version 2018	1
Switzerland	Vital Registration data submitted to WHO/UN IGME version 2018	1
Syrian Arab Republic	World Fertility Survey 1978	1
	PAPCHILD Maternal and Child Health Survey 1993	1
	PAPFAM Family Health Survey 2001	1
	Health survey for causes of child deaths 2008	1
	PAPFAM Family Health Survey 2009	1
	Vital Registration data submitted to WHO/UN IGME version 2018	0
Tajikistan	Tajikistan Living Standards Survey 2007	0
	Demographic and Health Survey 2012	1
	Demographic and Health Survey (Preliminary) 2017	1
Thailand	World Fertility Survey 1975	1
	Demographic and Health Survey 1987	1
	Vital Registration from Ministry of Public Health 2015	0
	Vital Registration data submitted to WHO/UN IGME version 2018	0
The former Yugoslav Republic of Macedonia	Vital Registration data submitted to WHO/UN IGME version 2018	1
	Demographic and Health Survey 2003	1
	Demographic and Health Survey 2010	1
Timor-Leste	Demographic and Health Survey 2016	0
	Demographic and Health Survey 1988	1
	Demographic and Health Survey 1998	1
Togo	Demographic and Health Survey 2013-2014	1
Tonga	National Demographic and Health Survey 2012	1
Trinidad and Tobago	World Fertility Survey 1977	1
	Demographic and Health Survey 1987	1
	Vital Registration data submitted to WHO/UN IGME version 2018	0
Tunisia	World Fertility Survey 1978	0
	Demographic and Health Survey 1988	1
	PAPCHILD Maternal and Child Health Survey 1994	1
	PAPFAM Family Health Survey 2001	1
	Multiple Indicator Cluster Survey 2011-2012	1
Turkey	World Fertility Survey 1978	1
	Turkey Population and Health Survey 1988	1

	Demographic and Health Survey 1993	1
	Demographic and Health Survey 1998	1
	Demographic and Health Survey 2004	1
	Turkey Demographic and Health Survey 2008	1
	Child Mortality Survey 2010	0
	Child Mortality Survey 2012	0
	Demographic and Health Survey 2013	1
	Recalculated WHO Vital Registration Data 2018 adjusted (2009-16) 2018	1
	Demographic and Health Survey 2000	1
Turkmenistan	Multiple Indicator Cluster Survey 2015-2016	0
Tuvalu	Demographic and Health Survey 2007	1
	Demographic and Health Survey 1988-1989	1
	Demographic and Health Survey 1995	1
	Demographic and Health Survey 2000-2001	1
	Demographic and Health Survey 2006	1
Uganda	Malaria Indicator Survey 2010	1
	Demographic and Health Survey 2011	1
	Demographic and Health Survey 2016	1
	Demographic and Health Survey 2007	1
Ukraine	Vital Registration data submitted to WHO/UN IGME version 2018	0
	Family Health Survey 1995	1
United Arab Emirates	Vital Registration data submitted to WHO/UN IGME version 2018	0
United Kingdom	Vital Registration data submitted to WHO/UN IGME version 2018	1
	Demographic and Health Survey 1991-1992	1
	Demographic and Health Survey 1996	1
	Demographic and Health Survey 1999	1
	Demographic and Health Survey 2004-2005	1
	AIDS Indicator Survey 2007-2008	1
	Demographic and Health Survey 2010	1
United Republic of Tanzania	Demographic and Health Survey 2015-2016	1
United States	Vital Registration data submitted to WHO/UN IGME version 2018	1
Uruguay	Vital Registration data submitted to WHO/UN IGME version 2018	1
	Demographic and Health Survey 1996	0
	Demographic and Health Survey 2002	1
	Vital registration from National Statistical Office 2017	0
Uzbekistan	Vital Registration data submitted to WHO/UN IGME version 2018	0
Vanuatu	Demographic and Health Survey 2013	1
	National Population and Family Survey 1998	1
	Vital Registration Data from Ministerio del Poder Popular para la Salud 2017	0
Venezuela (Bolivarian Republic of)	Vital Registration data submitted to WHO/UN IGME version 2018	1

	Demographic and Health Survey 1997	1
	Demographic and Health Survey 2002	1
Viet Nam	Multiple Indicator Cluster Survey 2013-2014	1
Yemen	World Fertility Survey 1979	1
	Demographic and Health Survey 1991-1992	1
	Demographic and Health Survey 1997	1
	PAPFAM Family Health Survey 2003	1
	Multiple Indicator Cluster Survey 2006	1
	Demographic and Health Survey 2013	1
	Demographic and Health Survey 1992	1
	Demographic and Health Survey 1996-1997	1
	Demographic and Health Survey 2001-2002	1
	Demographic and Health Survey 2007	1
Zambia	Demographic and Health Survey 2013-2014	1
	Demographic and Health Survey 1988-1989	1
	Demographic and Health Survey 1994	1
	Demographic and Health Survey 1999	1
	Demographic and Health Survey 2005-2006	0
	Multiple Indicator Cluster Survey 2009	1
	Demographic and Health Survey 2010-2011	1
Zimbabwe	Multiple Indicator Cluster Survey 2014	1
	Demographic and Health Survey 2015	1

Table A.7. UN IGME and IHME GBD estimates and their (relative) differences for NMR, U5MR and ratio of NMR/U5MR for 1990, 2000, and 2017. Relative differences in NMR of more than 10% or absolute differences of more than five deaths per 1,000 births, relative differences in the estimates of U5MR of more than 10% or absolute differences of more than 10 deaths per 1,000 births, as well as relative differences in the ratio of NMR/U5MR of more than 10% or of absolute differences of more than 0.1 are highlighted.

Note: IHME GBD NMR was calculated using published early and late neonatal mortality rates. Downloaded from https://vizhub.healthdata.org/mortality/5q0-analysis (as of December 25, 2018)

	1990											
	Neonatal mortality rate				Under-five mortality rate				Ratio			
**Country or territory**	**UN IGME**	**IHME GBD**	**Difference**	**Difference (%)**	**UN IGME**	**IHME GBD**	**Difference**	**Difference (%)**	**UN IGME**	**IHME GBD**	**Difference**	**Difference (%)**
Afghanistan	73.5	51.0	-22.5	-30.7	175.1	152.7	-22.4	-12.8	0.42	0.33	-0.09	-20.50
Albania	12.6	15.1	2.5	19.7	40.1	43.2	3.1	7.6	0.31	0.35	0.04	11.20
Algeria	23.5	20.6	-2.9	-12.5	49.6	47.4	-2.2	-4.5	0.47	0.43	-0.04	-8.40
Andorra	3.9	2.6	-1.3	-33.8	8.5	6.0	-2.5	-29.3	0.46	0.43	-0.03	-6.40
Angola	54.0	55.6	1.6	3.0	223.5	227.3	3.8	1.7	0.24	0.24	0.00	1.30
Antigua and Barbuda	14.4	7.7	-6.7	-46.2	26.3	13.5	-12.8	-48.9	0.55	0.58	0.03	5.20
Argentina	15.0	16.0	1.0	6.8	28.8	29.3	0.5	1.6	0.52	0.55	0.03	5.20
Armenia	23.0	20.3	-2.7	-11.5	49.6	41.9	-7.7	-15.4	0.46	0.49	0.02	4.60

Australia	4.6	4.8	0.2	4.1	9.2	9.5	0.3	2.9	0.50	0.51	0.01	1.20
Austria	4.6	4.7	0.1	2.9	9.5	9.5	0.0	0.3	0.48	0.50	0.01	2.60
Azerbaijan	31.2	31.5	0.3	1.1	95.3	78.4	-16.9	-17.8	0.33	0.40	0.08	22.90
Bahamas	13.7	12.4	-1.3	-9.7	23.5	22.4	-1.1	-4.7	0.58	0.55	-0.03	-5.20
Bahrain	15.0	12.6	-2.4	-16.2	23.0	22.8	-0.2	-0.9	0.65	0.55	-0.10	-15.40
Bangladesh	64.1	59.5	-4.6	-7.2	143.8	136.5	-7.3	-5.1	0.45	0.44	-0.01	-2.30
Barbados	11.7	12.1	0.4	3.6	17.9	18.2	0.3	1.5	0.65	0.67	0.01	2.10
Belarus	8.2	11.1	2.9	35.6	15.2	21.3	6.1	39.9	0.54	0.52	-0.02	-3.00
Belgium	4.6	4.7	0.1	2.0	10.0	9.7	-0.3	-2.7	0.46	0.48	0.02	4.80
Belize	18.7	14.9	-3.8	-20.4	38.5	38.3	-0.2	-0.6	0.49	0.39	-0.10	-19.90
Benin	46.2	45.9	-0.3	-0.7	177.8	180.7	2.9	1.6	0.26	0.25	-0.01	-2.30
Bhutan	42.7	59.1	16.4	38.4	127.9	132.8	4.9	3.9	0.33	0.45	0.11	33.30
Bolivia (Plurinational State of)	41.8	35.0	-6.8	-16.4	123.8	113.5	-10.3	-8.3	0.34	0.31	-0.03	-8.80
Bosnia and Herzegovina	11.3	12.3	1.0	8.9	18.2	16.3	-1.9	-10.5	0.62	0.75	0.13	21.60
Botswana	25.5	22.6	-2.9	-11.5	51.3	53.7	2.4	4.8	0.50	0.42	-0.08	-15.60
Brazil	25.3	26.3	1.0	3.9	63.1	69.5	6.4	10.2	0.40	0.38	-0.02	-5.70
Brunei Darussalam	5.7	5.5	-0.2	-4.3	13.4	11.5	-1.9	-14.0	0.43	0.47	0.05	11.30
Bulgaria	8.0	8.2	0.2	2.4	18.4	18.6	0.2	1.2	0.43	0.44	0.01	1.20
Burkina Faso	45.9	46.8	0.9	1.9	199.8	209.2	9.4	4.7	0.23	0.22	-0.01	-2.70
Burundi	40.0	42.8	2.8	7.1	174.6	180.3	5.7	3.3	0.23	0.24	0.01	3.70
Cabo Verde	20.6	19.2	-1.4	-6.6	62.7	64.6	1.9	3.0	0.33	0.30	-0.03	-9.30
Cambodia	40.2	41.6	1.4	3.5	115.9	119.4	3.5	3.0	0.35	0.35	0.00	0.40
Cameroon	39.5	39.3	-0.2	-0.5	138.6	135.6	-3.0	-2.2	0.28	0.29	0.00	1.70
Canada	4.3	4.6	0.3	6.1	8.3	8.4	0.1	1.3	0.52	0.54	0.02	4.70
Central African Republic	51.6	49.5	-2.1	-4.0	175.8	177.0	1.2	0.7	0.29	0.28	-0.01	-4.70
Chad	52.0	48.0	-4.0	-7.7	212.8	196.8	-16.0	-7.5	0.24	0.24	0.00	-0.20
Chile	8.6	9.0	0.4	4.2	19.1	19.4	0.3	1.4	0.45	0.46	0.01	2.80
China	29.5	16.0	-13.5	-45.6	53.8	49.9	-3.9	-7.2	0.55	0.32	-0.23	-41.40
Colombia	17.9	17.3	-0.6	-3.5	35.1	33.5	-1.6	-4.7	0.51	0.52	0.01	1.30
Comoros	50.0	47.7	-2.3	-4.5	124.8	121.7	-3.1	-2.5	0.40	0.39	-0.01	-2.00
Congo	28.0	26.3	-1.7	-6.2	90.0	99.2	9.2	10.2	0.31	0.26	-0.05	-14.90
Costa Rica	8.9	9.2	0.3	3.6	16.8	17.0	0.2	1.3	0.53	0.54	0.01	2.20
Côte d’Ivoire	48.3	45.8	-2.5	-5.1	151.9	148.4	-3.5	-2.3	0.32	0.31	-0.01	-2.90
Croatia	8.1	7.7	-0.4	-4.7	12.8	12.2	-0.6	-4.8	0.63	0.63	0.00	0.10
Cuba	6.8	6.7	-0.1	-1.2	13.2	13.4	0.2	1.2	0.52	0.50	-0.01	-2.40
Cyprus	5.4	7.8	2.4	44.7	11.1	13.9	2.8	25.5	0.49	0.56	0.07	15.30
Czechia	7.2	6.9	-0.3	-3.7	12.2	12.1	-0.1	-1.1	0.59	0.57	-0.02	-2.60
Democratic People's Republic of Korea	21.8	11.0	-10.8	-49.7	43.3	28.4	-14.9	-34.4	0.50	0.39	-0.12	-23.40
Democratic Republic of the Congo	41.8	36.7	-5.1	-12.1	186.3	160.6	-25.7	-13.8	0.22	0.23	0.00	2.00
Denmark	4.4	4.6	0.2	4.1	8.9	9.1	0.2	2.6	0.49	0.50	0.01	1.50
Djibouti	49.6	31.1	-18.5	-37.4	118.6	113.8	-4.8	-4.0	0.42	0.27	-0.15	-34.80
Dominica	10.6	10.9	0.3	2.6	17.1	18.8	1.7	9.8	0.62	0.58	-0.04	-6.60
Dominican Republic	24.4	26.3	1.9	7.8	60.1	65.9	5.8	9.6	0.41	0.40	-0.01	-1.60
Ecuador	22.6	15.1	-7.5	-33.3	54.2	41.7	-12.5	-23.1	0.42	0.36	-0.06	-13.30
Egypt	33.3	31.5	-1.8	-5.4	85.7	83.1	-2.6	-3.0	0.39	0.38	-0.01	-2.40
El Salvador	22.8	20.2	-2.6	-11.3	59.7	53.2	-6.5	-10.9	0.38	0.38	0.00	-0.50
Equatorial Guinea	48.2	51.9	3.7	7.7	178.7	193.7	15.0	8.4	0.27	0.27	0.00	-0.60

Eritrea	33.8	38.6	4.8	14.1	150.8	189.8	39.0	25.8	0.22	0.20	-0.02	-9.30
Estonia	9.6	8.9	-0.7	-6.9	17.7	17.0	-0.7	-4.2	0.54	0.53	-0.02	-2.80
Eswatini	21.9	24.6	2.7	12.2	68.6	69.6	1.0	1.4	0.32	0.35	0.03	10.60
Ethiopia	59.5	53.0	-6.5	-10.9	202.0	197.7	-4.3	-2.1	0.29	0.27	-0.03	-8.90
Fiji	11.9	12.8	0.9	7.2	29.1	26.6	-2.5	-8.5	0.41	0.48	0.07	17.10
Finland	3.8	4.0	0.2	5.6	6.7	6.9	0.2	2.9	0.57	0.58	0.01	2.60
France	3.6	3.8	0.2	5.5	9.0	8.8	-0.2	-2.3	0.40	0.43	0.03	8.00
Gabon	31.0	27.8	-3.2	-10.5	92.4	83.1	-9.3	-10.0	0.34	0.33	0.00	-0.50
Gambia	49.9	36.9	-13.0	-26.1	170.1	114.0	-56.1	-33.0	0.29	0.32	0.03	10.40
Georgia	24.8	21.9	-2.9	-11.6	47.7	41.2	-6.5	-13.7	0.52	0.53	0.01	2.40
Germany	3.4	3.9	0.5	14.1	8.5	8.7	0.2	2.0	0.40	0.45	0.05	11.90
Ghana	42.1	39.3	-2.8	-6.6	126.5	122.1	-4.4	-3.5	0.33	0.32	-0.01	-3.20
Greece	6.5	6.7	0.2	2.5	10.4	10.6	0.2	1.6	0.63	0.63	0.01	1.00
Grenada	12.0	12.2	0.2	1.8	23.4	23.9	0.5	2.0	0.51	0.51	0.00	-0.20
Guatemala	28.5	20.6	-7.9	-27.7	81.4	78.3	-3.1	-3.8	0.35	0.26	-0.09	-24.80
Guinea	61.7	59.0	-2.7	-4.3	234.6	223.1	-11.5	-4.9	0.26	0.26	0.00	0.60
Guinea-Bissau	64.2	59.3	-4.9	-7.7	223.9	206.6	-17.3	-7.7	0.29	0.29	0.00	0.10
Guyana	30.8	31.4	0.6	2.1	60.5	52.1	-8.4	-13.9	0.51	0.60	0.09	18.50
Haiti	38.9	41.6	2.7	6.9	144.8	148.0	3.2	2.2	0.27	0.28	0.01	4.60
Honduras	21.7	18.7	-3.0	-13.9	58.2	53.4	-4.8	-8.3	0.37	0.35	-0.02	-6.10
Hungary	11.2	11.1	-0.1	-0.6	17.3	17.2	-0.1	-0.8	0.65	0.65	0.00	0.20
Iceland	3.4	3.7	0.3	9.2	6.3	6.4	0.1	2.2	0.54	0.58	0.04	6.80
India	57.4	49.1	-8.3	-14.5	126.0	111.2	-14.8	-11.8	0.46	0.44	-0.01	-3.00
Indonesia	30.1	32.7	2.6	8.5	84.0	86.0	2.0	2.4	0.36	0.38	0.02	6.00
Iran (Islamic Republic of)	26.1	29.4	3.3	12.6	56.1	64.3	8.2	14.6	0.47	0.46	-0.01	-1.70
Iraq	26.1	24.2	-1.9	-7.3	53.9	51.6	-2.3	-4.2	0.48	0.47	-0.02	-3.20
Ireland	4.7	5.0	0.3	5.9	9.2	9.4	0.2	1.9	0.51	0.53	0.02	3.90
Israel	6.2	6.2	0.0	-0.6	11.6	11.8	0.2	2.1	0.53	0.52	-0.01	-2.60
Italy	6.4	6.2	-0.2	-2.7	9.7	9.5	-0.2	-2.0	0.66	0.65	0.00	-0.70
Jamaica	20.1	12.1	-8.0	-39.7	30.6	26.2	-4.4	-14.5	0.66	0.46	-0.19	-29.50
Japan	2.5	2.7	0.2	7.2	6.3	6.4	0.1	0.9	0.40	0.42	0.02	6.20
Jordan	20.2	19.3	-0.9	-4.5	36.4	34.5	-1.9	-5.3	0.55	0.56	0.00	0.70
Kazakhstan	22.2	17.4	-4.8	-21.8	52.5	38.0	-14.5	-27.6	0.42	0.46	0.03	8.00
Kenya	29.7	26.2	-3.5	-11.9	103.7	94.8	-8.9	-8.6	0.29	0.28	-0.01	-3.70
Kiribati	35.6	27.8	-7.8	-21.9	95.7	83.6	-12.1	-12.6	0.37	0.33	-0.04	-10.70
Kuwait	10.1	9.7	-0.4	-3.7	17.6	20.7	3.1	17.8	0.57	0.47	-0.10	-18.20
Kyrgyzstan	23.9	29.4	5.5	23.0	65.8	60.0	-5.8	-8.8	0.36	0.49	0.13	34.80
Lao People's Democratic Republic	48.2	63.5	15.3	31.8	154.2	182.5	28.3	18.3	0.31	0.35	0.04	11.40
Latvia	8.2	9.0	0.8	9.9	16.9	16.8	-0.1	-0.6	0.49	0.54	0.05	10.60
Lebanon	20.5	16.3	-4.2	-20.5	32.4	30.5	-1.9	-6.0	0.63	0.54	-0.10	-15.40
Lesotho	38.6	40.0	1.4	3.5	88.8	86.3	-2.5	-2.8	0.43	0.46	0.03	6.50
Liberia	58.4	53.4	-5.0	-8.6	260.5	221.8	-38.7	-14.9	0.22	0.24	0.02	7.40
Libya	21.0	18.3	-2.7	-12.7	41.8	39.4	-2.4	-5.8	0.50	0.47	-0.04	-7.30
Lithuania	8.0	7.6	-0.4	-4.7	15.2	14.3	-0.9	-6.1	0.53	0.53	0.01	1.40
Luxembourg	4.1	4.3	0.2	5.5	8.7	8.7	0.0	-0.1	0.47	0.50	0.03	5.70
Madagascar	39.8	37.5	-2.3	-5.8	160.0	146.6	-13.4	-8.4	0.25	0.26	0.01	2.80
Malawi	49.9	47.8	-2.1	-4.3	234.7	217.8	-16.9	-7.2	0.21	0.22	0.01	3.10
Malaysia	7.8	8.7	0.9	11.0	16.6	17.5	0.9	5.6	0.47	0.49	0.02	5.10

Maldives	42.5	28.9	-13.6	-32.1	93.8	63.5	-30.3	-32.3	0.45	0.45	0.00	0.20
Mali	72.8	65.4	-7.4	-10.1	254.0	235.6	-18.4	-7.3	0.29	0.28	-0.01	-3.10
Malta	7.6	7.5	-0.1	-1.4	11.3	10.9	-0.4	-3.2	0.67	0.69	0.01	1.90
Marshall Islands	19.5	15.1	-4.4	-22.8	48.9	33.0	-15.9	-32.4	0.40	0.46	0.06	14.30
Mauritania	46.1	42.7	-3.4	-7.3	117.9	108.8	-9.1	-7.7	0.39	0.39	0.00	0.50
Mauritius	14.6	13.9	-0.7	-4.5	23.0	21.7	-1.3	-5.8	0.63	0.64	0.01	1.40
Mexico	22.3	15.3	-7.0	-31.5	45.4	41.2	-4.2	-9.3	0.49	0.37	-0.12	-24.50
Micronesia (Federated States of)	25.6	18.8	-6.8	-26.4	55.1	43.0	-12.1	-21.9	0.46	0.44	-0.03	-5.80
Mongolia	29.6	29.0	-0.6	-2.0	107.8	94.5	-13.3	-12.3	0.27	0.31	0.03	11.70
Montenegro	10.6	11.1	0.5	4.7	16.6	18.3	1.7	10.4	0.64	0.61	-0.03	-5.10
Morocco	36.2	33.6	-2.6	-7.1	79.5	70.5	-9.0	-11.3	0.46	0.48	0.02	4.60
Mozambique	58.1	54.7	-3.4	-5.9	239.5	215.2	-24.3	-10.1	0.24	0.25	0.01	4.70
Myanmar	48.3	50.6	2.3	4.8	115.7	141.6	25.9	22.4	0.42	0.36	-0.06	-14.40
Namibia	27.7	27.4	-0.3	-1.0	72.7	70.3	-2.4	-3.3	0.38	0.39	0.01	2.40
Nepal	58.5	56.1	-2.4	-4.1	140.4	134.4	-6.0	-4.2	0.42	0.42	0.00	0.20
Netherlands	4.6	4.7	0.1	1.9	8.4	8.8	0.4	4.2	0.55	0.54	-0.01	-2.20
New Zealand	4.3	4.3	0.0	0.5	11.2	12.0	0.8	7.0	0.38	0.36	-0.02	-6.10
Nicaragua	23.2	21.2	-2.0	-8.7	66.9	64.6	-2.3	-3.4	0.35	0.33	-0.02	-5.50
Niger	54.3	46.1	-8.2	-15.1	326.5	294.3	-32.2	-9.9	0.17	0.16	-0.01	-5.80
Nigeria	50.3	48.8	-1.5	-2.9	211.9	201.3	-10.6	-5.0	0.24	0.24	0.01	2.20
Norway	4.0	4.7	0.7	16.8	8.7	8.8	0.1	1.0	0.46	0.53	0.07	15.60
Oman	17.4	21.6	4.2	24.0	39.2	48.1	8.9	22.6	0.44	0.45	0.00	1.10
Pakistan	63.6	53.2	-10.4	-16.4	139.0	111.9	-27.1	-19.5	0.46	0.47	0.02	3.80
Panama	17.0	13.1	-3.9	-22.9	30.7	27.2	-3.5	-11.4	0.55	0.48	-0.07	-13.00
Papua New Guinea	31.2	27.5	-3.7	-12.0	87.6	76.8	-10.8	-12.4	0.36	0.36	0.00	0.50
Paraguay	22.0	18.0	-4.0	-18.2	45.5	34.6	-10.9	-23.9	0.48	0.52	0.04	7.40
Peru	28.1	27.8	-0.3	-1.1	80.7	79.1	-1.6	-2.0	0.35	0.35	0.00	1.00
Philippines	19.3	19.1	-0.2	-1.2	57.6	56.0	-1.6	-2.8	0.34	0.34	0.01	1.60
Poland	11.1	11.7	0.6	5.0	17.3	18.1	0.8	4.4	0.64	0.65	0.00	0.60
Portugal	7.2	7.0	-0.2	-2.4	14.7	14.1	-0.6	-3.9	0.49	0.50	0.01	1.50
Qatar	11.3	11.3	0.0	0.0	20.8	20.9	0.1	0.7	0.54	0.54	0.00	-0.70
Republic of Korea	7.2	2.9	-4.3	-59.3	15.5	8.8	-6.7	-43.0	0.46	0.33	-0.13	-28.60
Republic of Moldova	18.6	17.3	-1.3	-6.8	33.2	34.1	0.9	2.6	0.56	0.51	-0.05	-9.20
Romania	15.0	9.6	-5.4	-36.1	31.1	32.3	1.2	3.8	0.48	0.30	-0.19	-38.40
Russian Federation	10.9	11.1	0.2	2.1	21.7	20.3	-1.4	-6.5	0.50	0.55	0.05	9.20
Rwanda	40.1	44.0	3.9	9.6	151.2	171.4	20.2	13.3	0.27	0.26	-0.01	-3.30
Saint Lucia	12.5	13.3	0.8	6.6	22.3	22.7	0.4	1.8	0.56	0.59	0.03	4.70
Saint Vincent and the Grenadines	12.4	15.6	3.2	25.4	24.3	29.3	5.0	20.7	0.51	0.53	0.02	3.90
Samoa	16.1	10.0	-6.1	-38.0	30.5	21.7	-8.8	-28.7	0.53	0.46	-0.07	-13.00
Sao Tome and Principe	26.6	20.6	-6.0	-22.5	108.6	101.0	-7.6	-7.0	0.24	0.20	-0.04	-16.70
Saudi Arabia	22.2	21.0	-1.2	-5.5	44.4	46.9	2.5	5.6	0.50	0.45	-0.05	-10.50
Senegal	39.8	37.9	-1.9	-4.7	139.3	138.5	-0.8	-0.6	0.29	0.27	-0.01	-4.20
Serbia	17.3	15.3	-2.0	-11.8	28.2	28.0	-0.2	-0.7	0.61	0.54	-0.07	-11.20
Seychelles	11.4	11.8	0.4	3.3	16.7	19.2	2.5	15.2	0.68	0.61	-0.07	-10.30
Sierra Leone	53.2	62.5	9.3	17.4	261.7	243.4	-18.3	-7.0	0.20	0.26	0.05	26.20
Singapore	4.0	3.9	-0.1	-2.9	7.7	8.1	0.4	4.7	0.52	0.48	-0.04	-7.30
Slovakia	8.7	8.3	-0.4	-5.1	14.7	14.2	-0.5	-3.5	0.59	0.58	-0.01	-1.60

Slovenia	5.8	5.5	-0.3	-5.7	10.4	9.6	-0.8	-7.6	0.56	0.57	0.01	2.00
Solomon Islands	15.3	19.5	4.2	27.5	38.3	47.7	9.4	24.6	0.40	0.41	0.01	2.30
Somalia	45.0	44.1	-0.9	-2.0	179.9	182.0	2.1	1.2	0.25	0.24	-0.01	-3.10
South Africa	20.9	28.4	7.5	36.0	60.9	77.6	16.7	27.4	0.34	0.37	0.02	6.80
South Sudan	65.8	47.2	-18.6	-28.2	253.6	197.3	-56.3	-22.2	0.26	0.24	-0.02	-7.70
Spain	4.9	5.0	0.1	2.1	9.2	9.2	0.0	0.0	0.53	0.54	0.01	2.10
Sri Lanka	12.9	15.2	2.3	17.5	21.3	28.3	7.0	32.7	0.61	0.54	-0.07	-11.50
State of Palestine	21.9	11.7	-10.2	-46.4	44.6	39.8	-4.8	-10.8	0.49	0.29	-0.20	-39.90
Sudan	42.6	46.4	3.8	8.9	131.5	140.8	9.3	7.0	0.32	0.33	0.01	1.70
Suriname	23.3	26.5	3.2	13.9	47.8	49.4	1.6	3.3	0.49	0.54	0.05	10.30
Sweden	3.5	3.6	0.1	3.9	7.0	6.9	-0.1	-1.7	0.50	0.53	0.03	5.70
Switzerland	3.9	4.2	0.3	8.3	8.2	8.4	0.2	2.8	0.48	0.50	0.03	5.30
Syrian Arab Republic	17.0	15.7	-1.3	-7.4	37.3	42.1	4.8	12.7	0.46	0.37	-0.08	-17.90
Tajikistan	31.5	27.5	-4.0	-12.8	104.9	89.1	-15.8	-15.1	0.30	0.31	0.01	2.60
Thailand	20.4	14.8	-5.6	-27.5	36.9	28.5	-8.4	-22.8	0.55	0.52	-0.03	-6.10
The former Yugoslav Republic of Macedonia	17.4	18.0	0.6	3.3	36.7	36.4	-0.3	-0.9	0.47	0.49	0.02	4.20
Timor-Leste	55.0	32.7	-22.3	-40.5	173.5	124.7	-48.8	-28.1	0.32	0.26	-0.05	-17.30
Togo	43.2	42.6	-0.6	-1.3	146.1	144.1	-2.0	-1.4	0.30	0.30	0.00	0.10
Tonga	10.3	10.3	0.0	0.4	22.1	23.3	1.2	5.4	0.47	0.44	-0.02	-4.80
Trinidad and Tobago	21.0	14.7	-6.3	-30.1	32.7	23.2	-9.5	-29.0	0.64	0.63	-0.01	-1.50
Tunisia	27.4	23.1	-4.3	-15.7	56.3	48.7	-7.6	-13.4	0.49	0.47	-0.01	-2.60
Turkey	33.8	31.4	-2.4	-7.0	74.0	70.8	-3.2	-4.3	0.46	0.44	-0.01	-2.90
Turkmenistan	28.2	29.5	1.3	4.6	84.4	70.4	-14.0	-16.6	0.33	0.42	0.08	25.40
Uganda	40.1	36.6	-3.5	-8.6	181.2	169.9	-11.3	-6.3	0.22	0.22	-0.01	-2.50
Ukraine	11.8	12.2	0.4	3.2	19.4	20.4	1.0	5.1	0.61	0.60	-0.01	-1.80
United Arab Emirates	8.4	9.6	1.2	14.2	16.6	18.2	1.6	9.4	0.51	0.53	0.02	4.40
United Kingdom	4.5	4.8	0.3	6.9	9.3	9.5	0.2	2.1	0.48	0.51	0.02	4.70
United Republic of Tanzania	37.8	37.9	0.1	0.3	165.5	154.8	-10.7	-6.5	0.23	0.24	0.02	7.20
United States of America	5.8	5.7	-0.1	-1.2	11.2	11.0	-0.2	-1.6	0.52	0.52	0.00	0.40
Uruguay	11.8	12.4	0.6	5.1	23.0	23.4	0.4	1.6	0.51	0.53	0.02	3.40
Uzbekistan	31.0	20.0	-11.0	-35.6	72.1	52.3	-19.8	-27.5	0.43	0.38	-0.05	-11.20
Vanuatu	16.3	15.7	-0.6	-3.7	35.6	35.1	-0.5	-1.3	0.46	0.45	-0.01	-2.40
Venezuela (Bolivarian Republic of)	13.0	16.0	3.0	23.0	29.6	32.8	3.2	10.7	0.44	0.49	0.05	11.10
Viet Nam	23.7	22.6	-1.1	-4.8	51.5	47.4	-4.1	-8.0	0.46	0.48	0.02	3.50
Yemen	43.6	41.0	-2.6	-5.9	126.0	128.6	2.6	2.1	0.35	0.32	-0.03	-7.80
Zambia	37.4	34.1	-3.3	-8.9	185.0	169.0	-16.0	-8.7	0.20	0.20	0.00	-0.20
Zimbabwe	25.5	22.3	-3.2	-12.7	77.5	67.2	-10.3	-13.4	0.33	0.33	0.00	0.80

	**2000**											
	**Neonatal mortality rate**				**Under-five mortality rate**				**Ratio**			
**Country or territory**	**UN IGME**	**IHME GBD**	**Difference**	**Difference (%)**	**UN IGME**	**IHME GBD**	**Difference**	**Difference (%)**	**UN IGME**	**IHME GBD**	**Difference**	**Difference (%)**
Afghanistan	60.8	41.6	-19.2	-31.6	128.8	120.5	-8.3	-6.4	0.47	0.34	-0.13	-26.90
Albania	11.0	13.8	2.8	25.5	24.5	28.4	3.9	16.1	0.45	0.49	0.04	8.10

Algeria	21.4	17.6	-3.8	-17.7	39.7	33.7	-6.0	-15.2	0.54	0.52	-0.02	-3.00
Andorra	2.1	1.4	-0.7	-32.7	5.0	3.4	-1.6	-31.1	0.42	0.41	-0.01	-2.20
Angola	50.7	44.7	-6.0	-11.9	206.3	181.6	-24.7	-12.0	0.25	0.25	0.00	0.10
Antigua and Barbuda	8.6	9.4	0.8	9.7	14.9	14.9	0.0	0.0	0.58	0.63	0.06	9.70
Argentina	10.6	10.6	0.0	-0.3	19.8	19.4	-0.4	-2.3	0.54	0.55	0.01	2.00
Armenia	15.7	14.4	-1.3	-8.1	30.0	27.4	-2.6	-8.5	0.52	0.53	0.00	0.40
Australia	3.5	3.5	0.0	1.3	6.2	6.3	0.1	2.1	0.56	0.56	0.00	-0.80
Austria	3.1	3.1	0.0	1.5	5.5	5.6	0.1	2.6	0.56	0.56	-0.01	-1.00
Azerbaijan	34.1	30.6	-3.5	-10.1	74.7	64.6	-10.1	-13.5	0.46	0.47	0.02	3.90
Bahamas	8.3	7.3	-1.0	-12.0	16.0	15.1	-0.9	-5.5	0.52	0.48	-0.04	-6.90
Bahrain	4.7	5.1	0.4	7.8	12.5	11.7	-0.8	-6.1	0.38	0.43	0.06	14.80
Bangladesh	42.4	42.5	0.1	0.2	87.4	85.8	-1.6	-1.9	0.49	0.5	0.01	2.10
Barbados	9.2	9.5	0.3	3.2	15.2	14.9	-0.3	-1.9	0.61	0.64	0.03	5.30
Belarus	6.0	9.1	3.1	51.8	12.8	17.7	4.9	38.3	0.47	0.51	0.05	9.70
Belgium	3.0	3.1	0.1	3.2	5.9	6.0	0.1	1.7	0.51	0.52	0.01	1.50
Belize	11.9	13.4	1.5	12.2	23.7	27.9	4.2	17.6	0.5	0.48	-0.02	-4.60
Benin	40.1	40.0	-0.1	-0.3	143.2	140.2	-3.0	-2.1	0.28	0.29	0.01	1.80
Bhutan	32.1	41.5	9.4	29.2	77.7	83.4	5.7	7.3	0.41	0.5	0.08	20.40
Bolivia (Plurinational State of)	29.8	25.8	-4.0	-13.3	79.6	71.3	-8.3	-10.4	0.37	0.36	-0.01	-3.20
Bosnia and Herzegovina	6.5	7.6	1.1	17.1	9.9	10.6	0.7	7.2	0.66	0.72	0.06	9.20
Botswana	26.3	20.0	-6.3	-24.0	87.0	62.4	-24.6	-28.3	0.3	0.32	0.02	5.90
Brazil	18.3	17.5	-0.8	-4.4	34.7	41.4	6.7	19.4	0.53	0.42	-0.10	-19.90
Brunei Darussalam	5.0	4.9	-0.1	-1.0	11.8	9.7	-2.1	-17.4	0.42	0.51	0.08	19.80
Bulgaria	7.9	7.6	-0.3	-3.5	17.6	16.9	-0.7	-3.8	0.45	0.45	0.00	0.30
Burkina Faso	41.0	37.5	-3.5	-8.5	180.4	179.6	-0.8	-0.4	0.23	0.21	-0.02	-8.10
Burundi	37.1	41.9	4.8	12.9	156.7	168.9	12.2	7.8	0.24	0.25	0.01	4.70
Cabo Verde	17.2	17.3	0.1	0.6	35.5	50.6	15.1	42.6	0.48	0.34	-0.14	-29.40
Cambodia	35.4	32.6	-2.8	-7.9	107.0	93.8	-13.2	-12.4	0.33	0.35	0.02	5.10
Cameroon	32.9	33.0	0.1	0.3	150.3	134.2	-16.1	-10.7	0.22	0.25	0.03	12.30
Canada	3.7	3.5	-0.2	-5.7	6.2	6.0	-0.2	-2.5	0.6	0.58	-0.02	-3.30
Central African Republic	49.3	47.7	-1.6	-3.3	175.2	180.6	5.4	3.1	0.28	0.26	-0.02	-6.20
Chad	44.6	41.5	-3.1	-6.9	186.5	183.6	-2.9	-1.5	0.24	0.23	-0.01	-5.50
Chile	5.5	5.8	0.3	4.7	10.9	11.4	0.5	4.2	0.5	0.51	0.00	0.40
China	21.4	12.9	-8.5	-39.5	36.8	33.9	-2.9	-8.0	0.58	0.38	-0.20	-34.30
Colombia	13.5	13.8	0.3	2.3	25.0	25.6	0.6	2.5	0.54	0.54	0.00	-0.10
Comoros	40.9	42.7	1.8	4.3	101.1	97.7	-3.4	-3.4	0.4	0.44	0.03	7.90
Congo	31.5	28.9	-2.6	-8.2	114.3	108.5	-5.8	-5.1	0.28	0.27	-0.01	-3.30
Costa Rica	7.7	7.4	-0.3	-4.4	12.9	12.4	-0.5	-3.9	0.6	0.59	0.00	-0.50
Côte d’Ivoire	45.4	46.1	0.7	1.5	146.8	137.7	-9.1	-6.2	0.31	0.33	0.03	8.20
Croatia	5.6	5.5	-0.1	-1.5	8.3	8.2	-0.1	-0.6	0.67	0.67	-0.01	-0.90
Cuba	4.1	4.2	0.1	2.8	8.1	8.6	0.5	6.0	0.51	0.49	-0.02	-3.10
Cyprus	3.1	4.2	1.1	36.7	6.6	7.2	0.6	8.7	0.47	0.59	0.12	25.70
Czechia	2.7	2.6	-0.1	-1.9	5.5	5.3	-0.2	-4.5	0.49	0.5	0.01	2.70
Democratic People's Republic of Korea	27.0	12.6	-14.4	-53.2	59.9	120.0	60.1	100.3	0.45	0.11	-0.35	-76.60
Democratic Republic of the Congo	38.8	34.9	-3.9	-10.0	161.3	151.5	-9.8	-6.1	0.24	0.23	-0.01	-4.20
Denmark	3.5	3.2	-0.3	-8.0	5.5	5.6	0.1	1.5	0.64	0.58	-0.06	-9.40
Djibouti	44.2	30.4	-13.8	-31.3	101.5	101.7	0.2	0.2	0.44	0.3	-0.14	-31.40

Dominica	11.3	11.5	0.2	1.8	15.4	19.3	3.9	25.3	0.73	0.6	-0.14	-18.70
Dominican Republic	23.6	23.3	-0.3	-1.1	41.0	41.8	0.8	1.9	0.58	0.56	-0.02	-2.90
Ecuador	14.1	16.3	2.2	15.5	29.1	34.6	5.5	18.8	0.48	0.47	-0.01	-2.80
Egypt	22.5	21.1	-1.4	-6.1	46.8	44.4	-2.4	-5.2	0.48	0.48	0.00	-1.00
El Salvador	14.7	12.1	-2.6	-17.7	32.9	28.4	-4.5	-13.8	0.45	0.43	-0.02	-4.60
Equatorial Guinea	44.6	38.1	-6.5	-14.5	156.4	137.7	-18.7	-12.0	0.29	0.28	-0.01	-2.80
Eritrea	26.0	24.6	-1.4	-5.3	88.4	91.6	3.2	3.6	0.29	0.27	-0.03	-8.70
Estonia	5.3	5.2	-0.1	-2.0	11.0	11.0	0.0	0.1	0.48	0.47	-0.01	-2.10
Eswatini	26.0	20.9	-5.1	-19.7	126.1	91.3	-34.8	-27.6	0.21	0.23	0.02	10.90
Ethiopia	48.7	43.5	-5.2	-10.6	142.6	145.8	3.2	2.2	0.34	0.3	-0.04	-12.60
Fiji	9.0	13.3	4.3	47.4	22.9	27.3	4.4	19.1	0.39	0.49	0.09	23.80
Finland	2.7	2.5	-0.2	-8.0	4.3	4.3	0.0	0.3	0.63	0.58	-0.05	-8.30
France	2.7	2.8	0.1	4.0	5.4	5.5	0.1	1.0	0.5	0.52	0.02	3.00
Gabon	28.8	27.0	-1.8	-6.2	83.1	69.7	-13.4	-16.1	0.35	0.39	0.04	11.70
Gambia	40.7	31.6	-9.1	-22.5	118.4	86.9	-31.5	-26.6	0.34	0.36	0.02	5.70
Georgia	22.1	20.1	-2.0	-8.9	35.4	33.9	-1.5	-4.1	0.62	0.59	-0.03	-5.00
Germany	2.8	2.8	0.0	-1.8	5.4	5.3	-0.1	-1.2	0.52	0.52	0.00	-0.60
Ghana	36.3	35.9	-0.4	-1.1	99.2	100.9	1.7	1.7	0.37	0.36	-0.01	-2.70
Greece	3.9	3.8	-0.1	-1.3	6.4	6.3	-0.1	-1.8	0.61	0.61	0.00	0.50
Grenada	8.2	10.2	2.0	24.9	15.7	17.1	1.4	9.2	0.52	0.6	0.07	14.40
Guatemala	21.2	16.3	-4.9	-23.3	51.9	50.2	-1.7	-3.3	0.41	0.32	-0.08	-20.70
Guinea	46.7	48.3	1.6	3.3	165.4	171.7	6.3	3.8	0.28	0.28	0.00	-0.40
Guinea-Bissau	55.0	50.8	-4.2	-7.6	175.8	161.1	-14.7	-8.4	0.31	0.32	0.00	0.90
Guyana	26.9	23.4	-3.5	-13.1	46.7	38.1	-8.6	-18.5	0.58	0.61	0.04	6.60
Haiti	30.1	31.9	1.8	6.1	104.0	102.9	-1.1	-1.1	0.29	0.31	0.02	7.30
Honduras	17.8	17.3	-0.5	-3.0	37.4	36.3	-1.1	-2.8	0.48	0.48	0.00	-0.10
Hungary	5.7	6.1	0.4	6.2	10.1	10.5	0.4	3.6	0.56	0.58	0.01	2.50
Iceland	2.1	2.0	-0.1	-6.1	4.1	4.0	-0.1	-3.4	0.51	0.5	-0.01	-2.80
India	45.1	38.0	-7.1	-15.7	91.7	83.1	-8.6	-9.4	0.49	0.46	-0.03	-7.00
Indonesia	22.3	24.9	2.6	11.6	52.1	53.9	1.8	3.4	0.43	0.46	0.03	7.90
Iran (Islamic Republic of)	18.9	21.7	2.8	14.8	34.2	39.6	5.4	15.8	0.55	0.55	0.00	-0.90
Iraq	24.4	21.4	-3.0	-12.1	44.9	41.4	-3.5	-7.9	0.54	0.52	-0.02	-4.60
Ireland	4.0	4.0	0.0	-0.4	7.2	7.0	-0.2	-2.1	0.56	0.56	0.01	1.70
Israel	3.6	3.5	-0.1	-1.5	6.9	6.8	-0.1	-1.1	0.52	0.52	0.00	-0.30
Italy	3.5	3.3	-0.2	-4.6	5.6	5.4	-0.2	-3.9	0.63	0.62	0.00	-0.70
Jamaica	17.3	12.9	-4.4	-25.4	22.3	19.9	-2.4	-10.9	0.78	0.65	-0.13	-16.30
Japan	1.8	1.8	0.0	-0.7	4.5	4.4	-0.1	-1.8	0.4	0.4	0.00	1.10
Jordan	16.7	16.6	-0.1	-0.5	27.8	27.6	-0.2	-0.8	0.6	0.6	0.00	0.30
Kazakhstan	21.0	17.5	-3.5	-16.8	43.0	35.3	-7.7	-17.8	0.49	0.49	0.01	1.20
Kenya	30.8	27.0	-3.8	-12.4	104.5	87.6	-16.9	-16.2	0.29	0.31	0.01	4.40
Kiribati	29.0	23.5	-5.5	-18.9	71.2	66.5	-4.7	-6.5	0.41	0.35	-0.05	-13.30
Kuwait	6.5	6.2	-0.3	-3.9	12.7	11.7	-1.0	-7.7	0.51	0.53	0.02	4.10
Kyrgyzstan	21.6	19.9	-1.7	-8.1	49.5	44.4	-5.1	-10.2	0.44	0.45	0.01	2.40
Lao People's Democratic Republic	40.1	50.6	10.5	26.1	112.6	136.6	24.0	21.3	0.36	0.37	0.01	4.00
Latvia	7.2	6.8	-0.4	-5.8	14.3	13.4	-0.9	-6.6	0.5	0.51	0.00	0.80
Lebanon	11.8	9.0	-2.8	-23.5	20.0	17.6	-2.4	-11.8	0.59	0.51	-0.08	-13.20
Lesotho	43.2	38.5	-4.7	-10.9	116.9	97.0	-19.9	-17.1	0.37	0.4	0.03	7.50
Liberia	44.7	41.2	-3.5	-7.7	186.1	169.9	-16.2	-8.7	0.24	0.24	0.00	1.10

Libya	14.9	12.6	-2.3	-15.1	28.1	26.4	-1.7	-5.9	0.53	0.48	-0.05	-9.80
Lithuania	4.6	4.7	0.1	2.5	10.7	10.6	-0.1	-0.9	0.43	0.44	0.01	3.50
Luxembourg	2.5	2.6	0.1	3.2	4.8	4.7	-0.1	-1.1	0.52	0.54	0.02	4.40
Madagascar	31.2	32.0	0.8	2.6	107.1	109.5	2.4	2.3	0.29	0.29	0.00	0.30
Malawi	39.4	36.9	-2.5	-6.3	171.9	156.9	-15.0	-8.7	0.23	0.24	0.01	2.60
Malaysia	5.3	4.7	-0.6	-12.1	10.2	9.3	-0.9	-8.7	0.52	0.5	-0.02	-3.70
Maldives	25.4	17.1	-8.3	-32.5	43.8	29.3	-14.5	-33.1	0.58	0.59	0.01	0.90
Mali	56.8	52.8	-4.0	-7.0	219.5	193.1	-26.4	-12.0	0.26	0.27	0.01	5.70
Malta	5.2	5.1	-0.1	-1.2	7.7	7.5	-0.2	-2.2	0.68	0.68	0.01	1.00
Marshall Islands	18.4	16.3	-2.1	-11.4	40.7	37.0	-3.7	-9.1	0.45	0.44	-0.01	-2.50
Mauritania	42.9	36.8	-6.1	-14.1	113.8	91.4	-22.4	-19.7	0.38	0.4	0.03	6.90
Mauritius	12.2	11.2	-1.0	-8.3	18.5	16.8	-1.7	-9.1	0.66	0.66	0.01	0.80
Mexico	13.0	11.0	-2.0	-15.6	26.8	25.0	-1.8	-6.6	0.49	0.44	-0.05	-9.60
Micronesia (Federated States of)	25.0	14.5	-10.5	-41.9	53.5	34.4	-19.1	-35.7	0.47	0.42	-0.04	-9.50
Mongolia	24.6	22.8	-1.8	-7.1	63.9	61.4	-2.5	-3.9	0.38	0.37	-0.01	-3.40
Montenegro	8.9	8.7	-0.2	-2.4	14.1	14.2	0.1	0.8	0.63	0.61	-0.02	-3.10
Morocco	27.4	24.5	-2.9	-10.4	49.8	45.7	-4.1	-8.3	0.55	0.54	-0.01	-2.30
Mozambique	44.0	41.8	-2.2	-5.0	170.4	159.3	-11.1	-6.5	0.26	0.26	0.00	1.70
Myanmar	37.9	41.9	4.0	10.7	89.8	110.5	20.7	23.1	0.42	0.38	-0.04	-10.10
Namibia	22.9	22.3	-0.6	-2.8	76.2	64.7	-11.5	-15.1	0.3	0.34	0.04	14.50
Nepal	40.6	39.5	-1.1	-2.8	81.5	79.4	-2.1	-2.6	0.5	0.5	0.00	-0.10
Netherlands	3.8	3.8	0.0	0.1	6.2	6.3	0.1	1.7	0.61	0.6	-0.01	-1.60
New Zealand	3.5	3.5	0.0	1.4	7.4	7.7	0.3	3.8	0.47	0.46	-0.01	-2.30
Nicaragua	16.2	13.0	-3.2	-20.0	38.7	35.7	-3.0	-7.7	0.42	0.36	-0.06	-13.30
Niger	43.1	40.9	-2.2	-5.1	223.7	225.7	2.0	0.9	0.19	0.18	-0.01	-5.90
Nigeria	48.1	47.4	-0.7	-1.4	186.2	180.4	-5.8	-3.1	0.26	0.26	0.00	1.70
Norway	2.7	2.6	-0.1	-2.5	4.9	4.9	0.0	-0.4	0.55	0.54	-0.01	-2.10
Oman	7.3	8.1	0.8	10.8	16.5	16.3	-0.2	-1.3	0.44	0.5	0.05	12.30
Pakistan	60.1	46.7	-13.4	-22.2	112.6	90.5	-22.1	-19.6	0.53	0.52	-0.02	-3.30
Panama	14.5	10.4	-4.1	-28.1	26.1	23.6	-2.5	-9.8	0.56	0.44	-0.11	-20.40
Papua New Guinea	29.8	25.6	-4.2	-14.2	77.7	67.0	-10.7	-13.8	0.38	0.38	0.00	-0.50
Paraguay	18.0	15.9	-2.1	-11.7	33.9	28.7	-5.2	-15.2	0.53	0.55	0.02	4.10
Peru	16.3	15.5	-0.8	-4.7	38.7	38.6	-0.1	-0.3	0.42	0.4	-0.02	-4.40
Philippines	16.8	16.4	-0.4	-2.3	38.7	38.6	-0.1	-0.2	0.43	0.42	-0.01	-2.10
Poland	5.7	5.8	0.1	2.0	9.3	9.3	0.0	0.1	0.61	0.62	0.01	1.90
Portugal	3.4	3.6	0.2	5.2	7.2	7.3	0.1	1.3	0.47	0.49	0.02	3.90
Qatar	6.6	7.4	0.8	12.4	12.5	14.3	1.8	14.6	0.53	0.52	-0.01	-1.90
Republic of Korea	3.4	2.7	-0.7	-21.2	7.5	6.6	-0.9	-12.5	0.45	0.41	-0.05	-10.00
Republic of Moldova	20.8	18.6	-2.2	-10.3	31.5	29.1	-2.4	-7.7	0.66	0.64	-0.02	-2.90
Romania	10.5	9.2	-1.3	-12.0	21.9	22.3	0.4	2.0	0.48	0.41	-0.07	-13.70
Russian Federation	9.5	10.6	1.1	12.0	19.4	19.5	0.1	0.3	0.49	0.55	0.06	11.70
Rwanda	38.8	38.1	-0.7	-1.8	181.4	157.9	-23.5	-12.9	0.21	0.24	0.03	12.80
Saint Lucia	11.3	11.7	0.4	3.8	18.1	18.4	0.3	1.8	0.62	0.64	0.01	2.00
Saint Vincent and the Grenadines	13.3	14.5	1.2	8.9	22.5	24.0	1.5	6.5	0.59	0.6	0.01	2.20
Samoa	11.5	7.9	-3.6	-31.7	21.8	16.9	-4.9	-22.3	0.53	0.46	-0.06	-12.00
Sao Tome and Principe	22.8	20.6	-2.2	-9.8	85.0	74.2	-10.8	-12.7	0.27	0.28	0.01	3.30
Saudi Arabia	11.6	13.8	2.2	18.6	22.0	26.5	4.5	20.4	0.53	0.52	-0.01	-1.50

Senegal	38.2	35.3	-2.9	-7.5	131.7	117.3	-14.4	-11.0	0.29	0.3	0.01	3.90
Serbia	7.7	8.7	1.0	13.0	12.6	15.9	3.3	26.3	0.61	0.55	-0.06	-10.50
Seychelles	8.6	7.6	-1.0	-11.9	13.6	13.7	0.1	0.5	0.63	0.55	-0.08	-12.40
Sierra Leone	50.5	54.9	4.4	8.7	233.1	214.1	-19.0	-8.2	0.22	0.26	0.04	18.30
Singapore	1.6	1.6	0.0	-1.1	3.9	3.3	-0.6	-14.7	0.41	0.48	0.07	15.90
Slovakia	5.1	5.2	0.1	1.8	9.8	9.7	-0.1	-1.5	0.52	0.54	0.02	3.30
Slovenia	3.3	3.2	-0.1	-1.7	5.5	5.4	-0.1	-2.7	0.6	0.61	0.01	1.10
Solomon Islands	12.7	16.9	4.2	33.0	30.3	38.1	7.8	25.8	0.42	0.44	0.02	5.80
Somalia	44.4	40.1	-4.3	-9.7	173.3	155.4	-17.9	-10.3	0.26	0.26	0.00	0.70
South Africa	17.8	23.3	5.5	31.0	78.4	77.0	-1.4	-1.8	0.23	0.3	0.08	33.30
South Sudan	57.2	39.7	-17.5	-30.5	182.9	144.5	-38.4	-21.0	0.31	0.28	-0.04	-12.00
Spain	2.8	2.9	0.1	1.9	5.4	5.5	0.1	1.1	0.52	0.52	0.00	0.80
Sri Lanka	9.6	8.7	-0.9	-9.5	16.5	16.3	-0.2	-1.2	0.58	0.53	-0.05	-8.40
State of Palestine	16.2	11.2	-5.0	-31.1	30.2	29.6	-0.6	-2.0	0.54	0.38	-0.16	-29.70
Sudan	37.2	33.9	-3.3	-8.9	104.8	97.4	-7.4	-7.1	0.35	0.35	-0.01	-2.00
Suriname	18.2	24.0	5.8	31.8	34.5	42.4	7.9	22.8	0.53	0.57	0.04	7.30
Sweden	2.3	2.1	-0.2	-7.1	4.1	3.9	-0.2	-4.8	0.56	0.55	-0.01	-2.50
Switzerland	3.5	3.6	0.1	1.5	5.6	5.8	0.2	4.2	0.63	0.61	-0.02	-2.50
Syrian Arab Republic	12.4	7.6	-4.8	-38.8	23.4	20.2	-3.2	-13.7	0.53	0.38	-0.15	-29.10
Tajikistan	28.4	25.6	-2.8	-9.9	87.6	71.9	-15.7	-18.0	0.32	0.36	0.03	9.80
Thailand	12.1	8.5	-3.6	-30.0	21.8	17.5	-4.3	-19.5	0.56	0.48	-0.07	-13.00
The former Yugoslav Republic of Macedonia	9.2	8.9	-0.3	-3.2	16.0	14.9	-1.1	-6.6	0.57	0.6	0.02	3.60
Timor-Leste	36.8	24.7	-12.1	-32.8	108.3	80.7	-27.6	-25.5	0.34	0.31	-0.03	-9.80
Togo	36.1	36.5	0.4	1.2	120.6	122.9	2.3	1.9	0.3	0.3	0.00	-0.70
Tonga	7.6	9.7	2.1	27.0	17.5	21.1	3.6	20.5	0.43	0.46	0.02	5.40
Trinidad and Tobago	21.0	15.4	-5.6	-26.9	32.7	25.9	-6.8	-20.9	0.64	0.59	-0.05	-7.60
Tunisia	18.0	15.8	-2.2	-12.3	31.7	26.8	-4.9	-15.5	0.57	0.59	0.02	3.80
Turkey	19.0	18.4	-0.6	-3.2	39.2	40.1	0.9	2.2	0.48	0.46	-0.03	-5.30
Turkmenistan	29.9	25.7	-4.2	-14.1	80.7	55.9	-24.8	-30.8	0.37	0.46	0.09	24.10
Uganda	29.4	34.4	5.0	17.0	146.0	137.1	-8.9	-6.1	0.2	0.25	0.05	24.60
Ukraine	11.0	8.4	-2.6	-24.0	18.5	14.9	-3.6	-19.4	0.59	0.56	-0.03	-5.60
United Arab Emirates	5.9	5.5	-0.4	-7.5	11.2	10.5	-0.7	-6.2	0.53	0.52	-0.01	-1.40
United Kingdom	3.8	3.7	-0.1	-2.6	6.5	6.5	0.0	0.0	0.58	0.57	-0.02	-2.60
United Republic of Tanzania	32.7	33.5	0.8	2.5	130.4	123.6	-6.8	-5.2	0.25	0.27	0.02	8.10
United States of America	4.6	4.6	0.0	0.8	8.4	8.3	-0.1	-0.9	0.55	0.56	0.01	1.70
Uruguay	8.3	8.2	-0.1	-1.4	17.0	16.4	-0.6	-3.4	0.49	0.5	0.01	2.10
Uzbekistan	28.4	19.8	-8.6	-30.1	62.3	45.2	-17.1	-27.5	0.46	0.44	-0.02	-3.70
Vanuatu	12.3	15.6	3.3	27.2	28.6	33.8	5.2	18.1	0.43	0.46	0.03	7.70
Venezuela (Bolivarian Republic of)	10.9	11.5	0.6	5.4	21.7	22.6	0.9	4.2	0.5	0.51	0.01	1.20
Viet Nam	14.8	13.7	-1.1	-7.3	29.7	27.1	-2.6	-8.7	0.5	0.51	0.01	1.50
Yemen	37.2	34.4	-2.8	-7.6	95.2	88.2	-7.0	-7.3	0.39	0.39	0.00	-0.30
Zambia	37.2	32.8	-4.4	-11.9	165.1	142.4	-22.7	-13.7	0.23	0.23	0.00	2.10
Zimbabwe	27.8	23.9	-3.9	-14.2	101.8	75.9	-25.9	-25.5	0.27	0.31	0.04	15.20

	2017											
	Neonatal mortality rate				Under-five mortality rate				Ratio			
Country or territory	UN IGME	IHME GBD	Difference	Difference (%)	UN IGME	IHME GBD	Difference (%)	Difference	UN IGME	IHME GBD	Difference	Difference (%)
Afghanistan	39.2	22.4	-16.8	-42.7	67.9	54.1	-13.8	-20.3	0.58	0.41	-0.16	-28.10
Albania	6.1	6.9	0.8	12.4	8.8	12.7	3.9	43.9	0.69	0.54	-0.15	-21.90
Algeria	14.9	13.7	-1.2	-8.2	24.0	22.8	-1.2	-5.2	0.62	0.6	-0.02	-3.10
Andorra	1.4	0.8	-0.6	-44.7	3.3	1.9	-1.4	-43.0	0.42	0.41	-0.01	-2.90
Angola	29.1	22.8	-6.3	-21.5	81.1	65.2	-15.9	-19.6	0.36	0.35	-0.01	-2.40
Antigua and Barbuda	3.8	5.6	1.8	48.1	7.4	11.1	3.7	50.2	0.51	0.51	-0.01	-1.40
Argentina	5.9	6.3	0.4	7.5	10.4	11.0	0.6	5.3	0.57	0.58	0.01	2.10
Armenia	6.7	5.5	-1.2	-18.0	12.6	9.6	-3.0	-24.2	0.53	0.58	0.04	8.20
Australia	2.1	2.2	0.1	6.4	3.5	3.9	0.4	10.6	0.6	0.58	-0.02	-3.80
Austria	2.2	2.1	-0.1	-6.3	3.6	3.4	-0.2	-5.0	0.61	0.6	-0.01	-1.40
Azerbaijan	11.8	20.2	8.4	71.4	23.0	35.2	12.2	53.0	0.51	0.57	0.06	12.00
Bahamas	4	6.5	2.5	61.9	7.2	12.5	5.3	74.0	0.56	0.52	-0.04	-7.00
Bahrain	3.1	3.1	0.0	-0.6	7.3	7.3	0.0	0.5	0.42	0.42	0.00	-1.20
Bangladesh	18.4	20.9	2.5	13.8	32.4	33.1	0.7	2.1	0.57	0.63	0.07	11.60
Barbados	8.1	9.1	1.0	11.8	12.4	13.0	0.6	5.1	0.65	0.7	0.04	6.40
Belarus	1.5	3.5	2.0	133.2	3.7	6.5	2.8	74.4	0.41	0.54	0.14	33.70
Belgium	2.1	2	-0.1	-6.9	3.8	3.6	-0.2	-4.3	0.55	0.54	-0.02	-2.70
Belize	8.8	10.3	1.5	16.6	14.2	17.9	3.7	25.9	0.62	0.57	-0.05	-7.40
Benin	32.7	29.2	-3.5	-10.7	98.3	83.0	-15.3	-15.6	0.33	0.35	0.02	5.80
Bhutan	16.9	18.3	1.4	8.5	30.8	29.3	-1.5	-4.9	0.55	0.63	0.08	14.00
Bolivia (Plurinational State of)	18.5	14.1	-4.4	-23.9	34.9	29.6	-5.3	-15.2	0.53	0.48	-0.05	-10.30
Bosnia and Herzegovina	4	5	1.0	24.9	5.7	6.4	0.7	12.6	0.7	0.78	0.08	10.90
Botswana	25.4	11.2	-14.2	-55.7	37.6	22.7	-14.9	-39.7	0.68	0.5	-0.18	-26.60
Brazil	8.5	8.5	0.0	-0.4	14.8	18.4	3.6	24.1	0.57	0.46	-0.11	-19.80
Brunei Darussalam	4.7	4.3	-0.4	-7.9	10.5	9.0	-1.5	-14.1	0.45	0.48	0.03	7.10
Bulgaria	3.7	4	0.3	8.1	7.5	7.7	0.2	2.4	0.49	0.52	0.03	5.50
Burkina Faso	25.4	26.4	1.0	4.1	81.2	106.0	24.8	30.5	0.31	0.25	-0.06	-20.30
Burundi	22.1	26.4	4.3	19.3	61.2	80.7	19.5	31.8	0.36	0.33	-0.03	-9.50
Cabo Verde	10.4	11.8	1.4	13.3	17.4	20.3	2.9	16.5	0.6	0.58	-0.02	-2.70
Cambodia	14.9	16.2	1.3	9.0	29.2	31.5	2.3	8.0	0.51	0.51	0.00	0.90
Cameroon	25.5	23.4	-2.1	-8.1	84.0	73.5	-10.5	-12.5	0.3	0.32	0.02	4.90
Canada	3.5	3.1	-0.4	-12.6	5.1	5.4	0.3	6.4	0.69	0.56	-0.12	-17.80
Central African Republic	41.5	39.1	-2.4	-5.7	121.5	123.9	2.4	2.0	0.34	0.32	-0.03	-7.50
Chad	34.8	31.5	-3.3	-9.4	123.2	117.1	-6.1	-5.0	0.28	0.27	-0.01	-4.60
Chile	5	4.6	-0.4	-7.6	7.4	7.3	-0.1	-1.9	0.68	0.64	-0.04	-5.90
China	4.7	4.8	0.1	2.9	9.3	12.0	2.7	29.3	0.51	0.4	-0.10	-20.40
Colombia	8	7.4	-0.6	-7.0	14.7	14.2	-0.5	-3.4	0.54	0.52	-0.02	-3.80
Comoros	31.7	25.3	-6.4	-20.2	69.0	48.0	-21.0	-30.4	0.46	0.53	0.07	14.70
Congo	19.4	19.8	0.4	2.0	47.5	55.2	7.7	16.2	0.41	0.36	-0.05	-12.20

Costa Rica	5.9	5.3	-0.6	-9.4	9.0	8.1	-0.9	-9.8	0.66	0.66	0.00	0.40
Côte d’Ivoire	33.5	32.1	-1.4	-4.1	88.8	83.2	-5.6	-6.3	0.38	0.39	0.01	2.40
Croatia	2.9	2.8	-0.1	-4.5	4.6	4.3	-0.3	-7.4	0.63	0.65	0.02	3.10
Cuba	2.2	2.3	0.1	5.0	5.4	5.1	-0.3	-4.7	0.41	0.45	0.04	10.20
Cyprus	1.6	1.7	0.1	7.5	2.7	2.9	0.2	8.9	0.59	0.59	-0.01	-1.30
Czechia	1.7	1.4	-0.3	-17.7	3.3	2.9	-0.4	-13.0	0.52	0.49	-0.03	-5.40
Democratic People's Republic of Korea	10	9.7	-0.3	-3.3	19.0	22.7	3.7	19.6	0.53	0.43	-0.10	-19.20
Democratic Republic of the Congo	28.9	25.4	-3.5	-12.1	91.1	81.9	-9.2	-10.1	0.32	0.31	-0.01	-2.20
Denmark	3.1	2.2	-0.9	-29.4	4.3	3.7	-0.6	-12.8	0.72	0.58	-0.14	-19.00
Djibouti	32.4	18.5	-13.9	-43.0	61.7	45.2	-16.5	-26.7	0.53	0.41	-0.12	-22.20
Dominica	27.3	18	-9.3	-33.9	34.0	30.3	-3.7	-10.7	0.8	0.59	-0.21	-25.90
Dominican Republic	20.1	19.3	-0.8	-4.1	29.9	29.3	-0.6	-2.2	0.67	0.66	-0.01	-2.00
Ecuador	7.5	7.9	0.4	4.9	14.5	17.5	3.0	20.5	0.52	0.45	-0.07	-13.00
Egypt	11.6	7.3	-4.3	-37.0	22.1	19.2	-2.9	-13.0	0.52	0.38	-0.14	-27.60
El Salvador	7.1	5.6	-1.5	-21.6	14.5	12.4	-2.1	-14.3	0.49	0.45	-0.04	-8.50
Equatorial Guinea	30.9	20.2	-10.7	-34.7	89.6	54.2	-35.4	-39.5	0.34	0.37	0.03	7.90
Eritrea	17.8	17	-0.8	-4.5	43.1	46.5	3.4	8.0	0.41	0.37	-0.05	-11.50
Estonia	1.2	1.3	0.1	7.8	2.7	3.0	0.3	10.8	0.44	0.43	-0.01	-2.80
Eswatini	17.4	15.5	-1.9	-11.0	53.9	45.2	-8.7	-16.2	0.32	0.34	0.02	6.20
Ethiopia	28.9	23.3	-5.6	-19.4	58.5	56.1	-2.4	-4.1	0.49	0.42	-0.08	-16.00
Fiji	10.6	12.3	1.7	16.4	25.3	25.7	0.4	1.6	0.42	0.48	0.06	14.70
Finland	1.8	1.2	-0.6	-32.4	2.3	2.2	-0.1	-4.2	0.78	0.55	-0.23	-29.40
France	2.4	2.1	-0.3	-12.9	4.2	3.9	-0.3	-7.5	0.57	0.54	-0.03	-5.80
Gabon	21.5	17.4	-4.1	-19.2	48.3	35.2	-13.1	-27.1	0.45	0.49	0.05	10.80
Gambia	27.6	21.8	-5.8	-21.0	63.6	47.8	-15.8	-24.9	0.43	0.46	0.02	5.20
Georgia	6.7	6.5	-0.2	-2.5	10.8	11.1	0.3	2.5	0.62	0.59	-0.03	-4.80
Germany	2.2	2	-0.2	-7.7	3.7	3.6	-0.1	-2.5	0.59	0.56	-0.03	-5.40
Ghana	24.2	26.6	2.4	10.1	49.3	55.6	6.3	12.8	0.49	0.48	-0.01	-2.40
Greece	3	2.5	-0.5	-15.3	5.3	4.5	-0.8	-16.0	0.57	0.57	0.00	0.80
Grenada	10.9	9.5	-1.4	-12.8	16.7	14.4	-2.3	-13.8	0.65	0.66	0.01	1.20
Guatemala	12.9	9.2	-3.7	-28.9	27.6	24.6	-3.0	-10.9	0.47	0.37	-0.09	-20.20
Guinea	24.1	31.2	7.1	29.6	85.7	96.7	11.0	12.9	0.28	0.32	0.04	14.80
Guinea-Bissau	37.3	31	-6.3	-16.8	84.2	73.2	-11.0	-13.0	0.44	0.42	-0.02	-4.40
Guyana	18.9	16	-2.9	-15.2	31.3	24.8	-6.5	-20.8	0.6	0.65	0.04	7.00
Haiti	28.3	24.4	-3.9	-13.6	71.7	59.3	-12.4	-17.3	0.39	0.41	0.02	4.40
Honduras	10	7.6	-2.4	-24.2	18.2	14.6	-3.6	-20.0	0.55	0.52	-0.03	-5.30
Hungary	2.4	2.6	0.2	6.8	4.5	4.8	0.3	5.8	0.53	0.54	0.01	1.00
Iceland	1	0.9	-0.1	-5.7	2.1	2.2	0.1	5.4	0.48	0.43	-0.05	-10.50
India	24	23.5	-0.5	-2.2	39.4	42.4	3.0	7.6	0.61	0.55	-0.06	-9.20
Indonesia	12.4	13.7	1.3	10.5	25.4	26.0	0.6	2.3	0.49	0.53	0.04	8.00
Iran (Islamic Republic of)	9.1	8.3	-0.8	-9.2	14.9	14.4	-0.5	-3.6	0.61	0.58	-0.04	-5.70
Iraq	17.1	12.4	-4.7	-27.4	30.4	25.1	-5.3	-17.5	0.56	0.5	-0.07	-12.00
Ireland	2.2	2.1	-0.1	-4.7	3.5	3.4	-0.1	-2.6	0.63	0.61	-0.01	-2.20
Israel	2	1.9	-0.1	-4.2	3.6	3.6	0.0	-0.3	0.56	0.53	-0.02	-4.00
Italy	2	1.9	-0.1	-4.4	3.4	3.2	-0.2	-5.7	0.59	0.6	0.01	1.30
Jamaica	10.8	10.4	-0.4	-3.4	15.2	14.5	-0.7	-4.4	0.71	0.72	0.01	1.00
Japan	0.9	1.1	0.2	19.1	2.6	2.6	0.0	-0.9	0.35	0.42	0.07	20.10

Jordan	10.1	8.7	-1.4	-14.1	17.0	14.4	-2.6	-15.2	0.59	0.6	0.01	1.40
Kazakhstan	5.3	6.9	1.6	29.6	10.0	14.1	4.1	41.3	0.53	0.49	-0.04	-8.30
Kenya	20.9	18.7	-2.2	-10.5	45.6	44.1	-1.5	-3.4	0.46	0.42	-0.03	-7.40
Kiribati	23	17.6	-5.4	-23.3	54.6	43.0	-11.6	-21.2	0.42	0.41	-0.01	-2.70
Kuwait	4.3	4.7	0.4	9.7	8.1	7.8	-0.3	-3.9	0.53	0.61	0.08	14.20
Kyrgyzstan	10.7	13	2.3	21.8	20.0	20.1	0.1	0.4	0.53	0.65	0.11	21.30
Lao People's Democratic Republic	28.2	25.6	-2.6	-9.3	63.4	57.5	-5.9	-9.3	0.44	0.45	0.00	0.10
Latvia	2.2	2.5	0.3	14.1	4.2	4.9	0.7	15.6	0.52	0.52	-0.01	-1.40
Lebanon	4.5	4.3	-0.2	-4.4	7.8	8.1	0.3	3.7	0.58	0.53	-0.05	-7.80
Lesotho	37.9	28.9	-9.0	-23.7	85.9	69.0	-16.9	-19.6	0.44	0.42	-0.02	-5.10
Liberia	25.1	25.2	0.1	0.3	74.7	74.8	0.1	0.2	0.34	0.34	0.00	0.10
Libya	6.5	5.2	-1.3	-20.5	12.4	14.3	1.9	15.7	0.52	0.36	-0.16	-31.30
Lithuania	2	2.2	0.2	12.3	4.3	4.8	0.5	11.9	0.47	0.47	0.00	0.30
Luxembourg	1.7	1.1	-0.6	-34.2	2.6	2.1	-0.5	-18.3	0.65	0.53	-0.13	-19.50
Madagascar	18.4	25.9	7.5	40.5	44.2	73.9	29.7	67.2	0.42	0.35	-0.07	-15.90
Malawi	22.7	25.7	3.0	13.3	55.4	65.2	9.8	17.6	0.41	0.39	-0.02	-3.70
Malaysia	4.3	3.9	-0.4	-8.9	7.9	7.2	-0.7	-8.8	0.54	0.54	0.00	0.00
Maldives	4.5	4.7	0.2	5.2	7.9	7.7	-0.2	-2.6	0.57	0.62	0.05	8.00
Mali	35.4	37.9	2.5	7.1	106.0	119.1	13.1	12.3	0.33	0.32	-0.02	-4.60
Malta	4.4	4.2	-0.2	-5.1	6.4	6.0	-0.4	-6.2	0.69	0.7	0.01	1.20
Marshall Islands	15.7	11.2	-4.5	-28.7	34.0	22.2	-11.8	-34.7	0.46	0.5	0.04	9.20
Mauritania	33.8	23.5	-10.3	-30.4	79.0	45.0	-34.0	-43.1	0.43	0.52	0.10	22.20
Mauritius	7.9	9	1.1	13.7	13.1	13.8	0.7	5.1	0.6	0.65	0.05	8.20
Mexico	7.6	6.8	-0.8	-10.8	13.4	15.7	2.3	17.1	0.57	0.43	-0.14	-23.80
Micronesia (Federated States of)	16.5	8.7	-7.8	-47.2	32.2	17.5	-14.7	-45.7	0.51	0.5	-0.01	-2.70
Mongolia	9.1	14.2	5.1	55.9	17.2	25.7	8.5	49.7	0.53	0.55	0.02	4.20
Montenegro	2.3	2.5	0.2	9.1	3.5	4.0	0.5	15.0	0.66	0.62	-0.03	-5.10
Morocco	14.4	11.8	-2.6	-17.9	23.3	19.7	-3.6	-15.3	0.62	0.6	-0.02	-3.10
Mozambique	26.9	24.5	-2.4	-8.9	72.4	73.8	1.4	1.9	0.37	0.33	-0.04	-10.60
Myanmar	24.1	21.3	-2.8	-11.8	48.6	44.3	-4.3	-8.8	0.5	0.48	-0.02	-3.30
Namibia	17.5	16.7	-0.8	-4.8	44.2	39.2	-5.0	-11.4	0.4	0.42	0.03	7.30
Nepal	20.7	19.4	-1.3	-6.2	33.7	31.4	-2.3	-6.7	0.61	0.62	0.00	0.50
Netherlands	2.3	2.5	0.2	8.4	3.9	3.9	0.0	0.0	0.59	0.64	0.05	8.40
New Zealand	3	2.4	-0.6	-19.6	5.3	4.5	-0.8	-15.7	0.57	0.54	-0.03	-4.60
Nicaragua	7.1	8.7	1.6	22.5	17.2	15.2	-2.0	-11.7	0.41	0.57	0.16	38.70
Niger	26	24	-2.0	-7.6	84.5	108.1	23.6	27.9	0.31	0.22	-0.09	-27.80
Nigeria	32.9	34.7	1.8	5.5	100.2	103.2	3.0	3.0	0.33	0.34	0.01	2.40
Norway	1.5	1.4	-0.1	-8.9	2.6	2.6	0.0	-0.3	0.58	0.53	-0.05	-8.60
Oman	5.1	5.7	0.6	11.0	11.3	10.6	-0.7	-6.1	0.45	0.53	0.08	18.30
Pakistan	44.2	35.9	-8.3	-18.8	74.9	58.2	-16.7	-22.2	0.59	0.62	0.03	4.50
Panama	8.9	7.7	-1.2	-13.8	16.1	17.2	1.1	6.9	0.55	0.45	-0.11	-19.30
Papua New Guinea	23.7	21.5	-2.2	-9.4	53.4	52.6	-0.8	-1.4	0.44	0.41	-0.04	-8.00
Paraguay	11.1	5.3	-5.8	-52.6	21.0	14.0	-7.0	-33.5	0.53	0.38	-0.15	-28.80
Peru	7.3	6.9	-0.4	-5.3	15.0	15.5	0.5	3.2	0.49	0.45	-0.04	-8.30
Philippines	13.6	12	-1.6	-11.8	28.1	26.6	-1.5	-5.3	0.48	0.45	-0.03	-6.90
Poland	3	2.7	-0.3	-9.7	4.7	4.4	-0.3	-6.3	0.64	0.62	-0.02	-3.60
Portugal	2.1	1.8	-0.3	-13.1	3.7	3.4	-0.3	-7.2	0.57	0.53	-0.04	-6.40

Qatar	3.8	3.8	0.0	0.0	7.6	7.4	-0.2	-3.0	0.5	0.52	0.02	3.10
Republic of Korea	1.5	1.5	0.0	-2.1	3.3	3.3	0.0	-0.3	0.45	0.45	-0.01	-1.80
Republic of Moldova	11.6	10.5	-1.1	-9.1	15.5	14.4	-1.1	-7.1	0.75	0.73	-0.02	-2.20
Romania	3.7	4.2	0.5	14.7	7.8	8.7	0.9	11.6	0.47	0.49	0.01	2.80
Russian Federation	3.3	4.1	0.8	24.2	7.6	7.4	-0.2	-2.4	0.43	0.55	0.12	27.20
Rwanda	16.4	18.3	1.9	11.5	37.9	47.0	9.1	23.9	0.43	0.39	-0.04	-10.00
Saint Lucia	12.4	9.9	-2.5	-20.3	16.6	13.8	-2.8	-17.1	0.75	0.72	-0.03	-3.90
Saint Vincent and the Grenadines	9.6	10.6	1.0	10.0	16.2	16.5	0.3	1.8	0.59	0.64	0.05	8.00
Samoa	8.6	6.4	-2.2	-25.1	16.5	14.1	-2.4	-14.6	0.52	0.46	-0.06	-12.30
Sao Tome and Principe	14.4	13.3	-1.1	-7.8	32.4	29.3	-3.1	-9.6	0.44	0.45	0.01	2.00
Saudi Arabia	3.9	4.3	0.4	9.6	7.4	7.9	0.5	7.3	0.53	0.54	0.01	2.20
Senegal	20.5	20.6	0.1	0.5	45.4	48.0	2.6	5.8	0.45	0.43	-0.02	-5.00
Serbia	3.7	3.1	-0.6	-15.6	5.7	5.0	-0.7	-12.2	0.65	0.62	-0.02	-3.80
Seychelles	8.6	6.9	-1.7	-20.3	14.2	12.5	-1.7	-12.0	0.61	0.55	-0.06	-9.40
Sierra Leone	33.5	32.7	-0.8	-2.5	110.5	110.7	0.2	0.2	0.3	0.29	-0.01	-2.70
Singapore	1.1	0.9	-0.2	-20.1	2.8	1.9	-0.9	-31.6	0.39	0.46	0.07	16.80
Slovakia	2.8	3	0.2	6.5	5.6	5.9	0.3	6.2	0.5	0.5	0.00	0.30
Slovenia	1.2	1.4	0.2	16.7	2.1	2.2	0.1	4.6	0.57	0.64	0.07	11.60
Solomon Islands	8.5	12.7	4.2	48.8	20.6	26.0	5.4	26.3	0.41	0.49	0.07	17.80
Somalia	38.5	30.5	-8.0	-20.9	127.2	102.6	-24.6	-19.4	0.3	0.3	-0.01	-1.90
South Africa	10.7	12.9	2.2	21.0	37.1	33.6	-3.5	-9.5	0.29	0.39	0.10	33.80
South Sudan	39.6	34.2	-5.4	-13.6	96.4	104.8	8.4	8.7	0.41	0.33	-0.08	-20.50
Spain	1.7	1.8	0.1	3.9	3.1	3.2	0.1	3.2	0.55	0.55	0.00	0.70
Sri Lanka	5.8	5.4	-0.4	-6.6	8.8	8.5	-0.3	-3.0	0.66	0.63	-0.02	-3.70
State of Palestine	11.3	6.4	-4.9	-43.4	20.9	13.6	-7.3	-35.0	0.54	0.47	-0.07	-12.90
Sudan	29.5	22.3	-7.2	-24.6	63.2	47.7	-15.5	-24.5	0.47	0.47	0.00	-0.10
Suriname	10.2	17.8	7.6	74.2	19.6	28.7	9.1	46.3	0.52	0.62	0.10	19.10
Sweden	1.7	1.5	-0.2	-12.9	2.8	2.7	-0.1	-2.0	0.61	0.54	-0.07	-11.10
Switzerland	3	2.6	-0.4	-14.3	4.2	3.8	-0.4	-9.6	0.71	0.68	-0.04	-5.30
Syrian Arab Republic	8.7	5.3	-3.4	-39.2	17.0	19.7	2.7	16.2	0.51	0.27	-0.24	-47.70
Tajikistan	14.5	21	6.5	45.0	33.6	46.9	13.3	39.7	0.43	0.45	0.02	3.80
Thailand	5.3	4.3	-1.0	-18.7	9.5	8.7	-0.8	-8.4	0.56	0.5	-0.06	-11.20
The former Yugoslav Republic of Macedonia	10.5	6.3	-4.2	-40.0	13.7	9.6	-4.1	-30.0	0.77	0.66	-0.11	-14.30
Timor-Leste	20.7	16	-4.7	-22.5	47.6	35.7	-11.9	-25.0	0.43	0.45	0.01	3.40
Togo	25.4	24.8	-0.6	-2.2	72.9	66.8	-6.1	-8.4	0.35	0.37	0.02	6.80
Tonga	6.7	7.6	0.9	12.8	16.0	16.6	0.6	3.6	0.42	0.46	0.04	8.90
Trinidad and Tobago	16.6	10.4	-6.2	-37.6	26.1	16.6	-9.5	-36.3	0.64	0.62	-0.01	-2.00
Tunisia	7.5	6.2	-1.3	-17.1	13.0	10.5	-2.5	-19.2	0.58	0.59	0.02	2.60
Turkey	5.9	7.6	1.7	29.3	11.6	14.2	2.6	22.6	0.51	0.54	0.03	5.50
Turkmenistan	21.3	15.7	-5.6	-26.5	47.3	29.1	-18.2	-38.4	0.45	0.54	0.09	19.50
Uganda	20.2	24.4	4.2	21.0	49.0	60.1	11.1	22.6	0.41	0.41	-0.01	-1.30
Ukraine	5.2	4.7	-0.5	-10.1	8.8	9.5	0.7	7.6	0.59	0.49	-0.10	-16.50
United Arab Emirates	4.7	4	-0.7	-14.9	9.1	7.1	-2.0	-21.7	0.52	0.56	0.05	8.80
United Kingdom	2.6	2.6	0.0	1.4	4.3	4.4	0.1	3.4	0.6	0.59	-0.01	-1.90
United Republic of Tanzania	21.1	24	2.9	13.9	54.0	61.5	7.5	14.0	0.39	0.39	0.00	-0.10
United States of America	3.6	3.9	0.3	7.1	6.6	6.7	0.1	1.8	0.55	0.57	0.03	5.20
Uruguay	4.6	5	0.4	8.6	8.2	9.1	0.9	10.4	0.56	0.55	-0.01	-1.60

Uzbekistan	12.1	12.4	0.3	2.8	22.5	23.8	1.3	6.0	0.54	0.52	-0.02	-3.00
Vanuatu	11.6	14.2	2.6	22.0	26.9	29.4	2.5	9.1	0.43	0.48	0.05	11.80
Venezuela (Bolivarian Republic of)	19.8	9.4	-10.4	-52.7	30.9	15.6	-15.3	-49.6	0.64	0.6	-0.04	-6.10
Viet Nam	10.6	7	-3.6	-34.2	20.9	13.1	-7.8	-37.2	0.51	0.53	0.02	4.80
Yemen	27	21.2	-5.8	-21.5	55.4	45.9	-9.5	-17.2	0.49	0.46	-0.03	-5.20
Zambia	22.2	22.4	0.2	0.9	60.0	62.5	2.5	4.1	0.37	0.36	-0.01	-3.10
Zimbabwe	22.4	24.1	1.7	7.7	50.3	56.1	5.8	11.6	0.45	0.43	-0.02	-3.50

5. Supplementary Figures

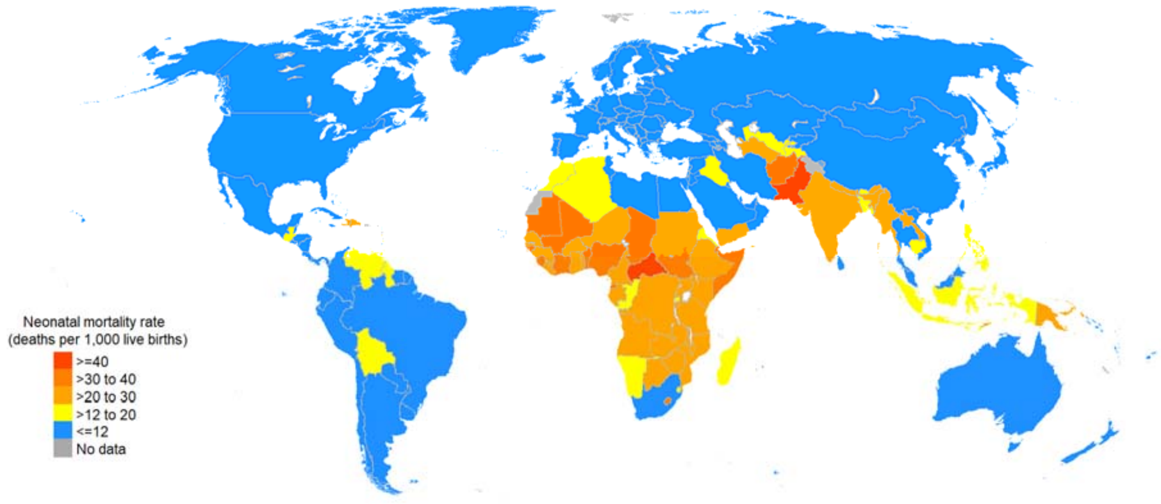

Figure A1: Neonatal mortality rate in 2017 by country

Note: This map does not reflect a position by UN IGME agencies or those of the institutions to which the authors are affiliated on the legal status of any country or territory. The dotted line represents approximately the Line of Control in Jammu and Kashmir agreed upon by India and Pakistan. The final status of Jammu and Kashmir has not yet been agreed upon by the parties. The final boundary between the Sudan and South Sudan has not yet been determined. The final status of the Abyei area has not yet been determined.

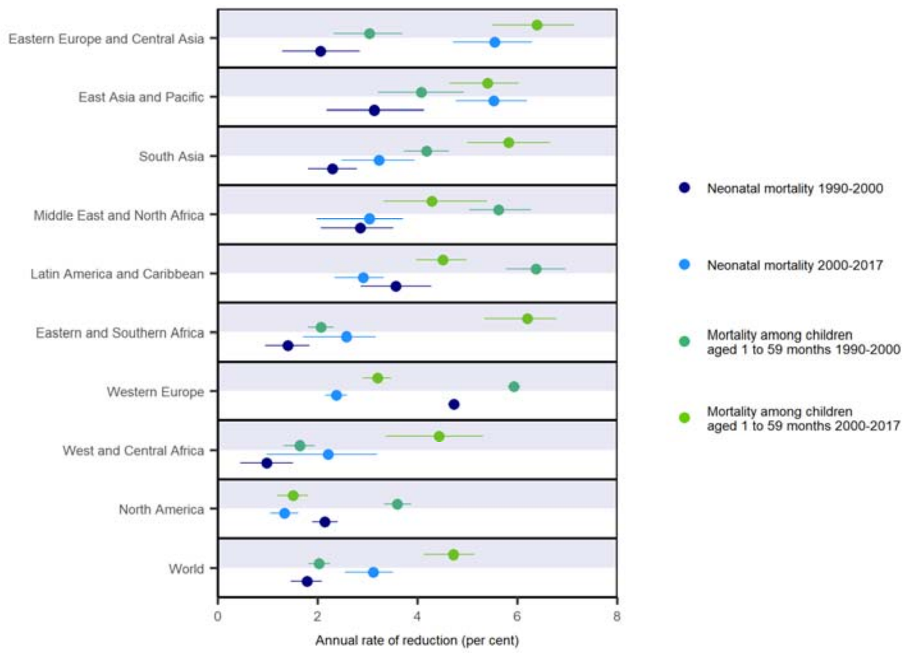

Figure A2: Annual rate of reduction in the neonatal mortality rate and the mortality rate among children aged 1 to 59 months, 1990-2000 and 2000-2017 by region

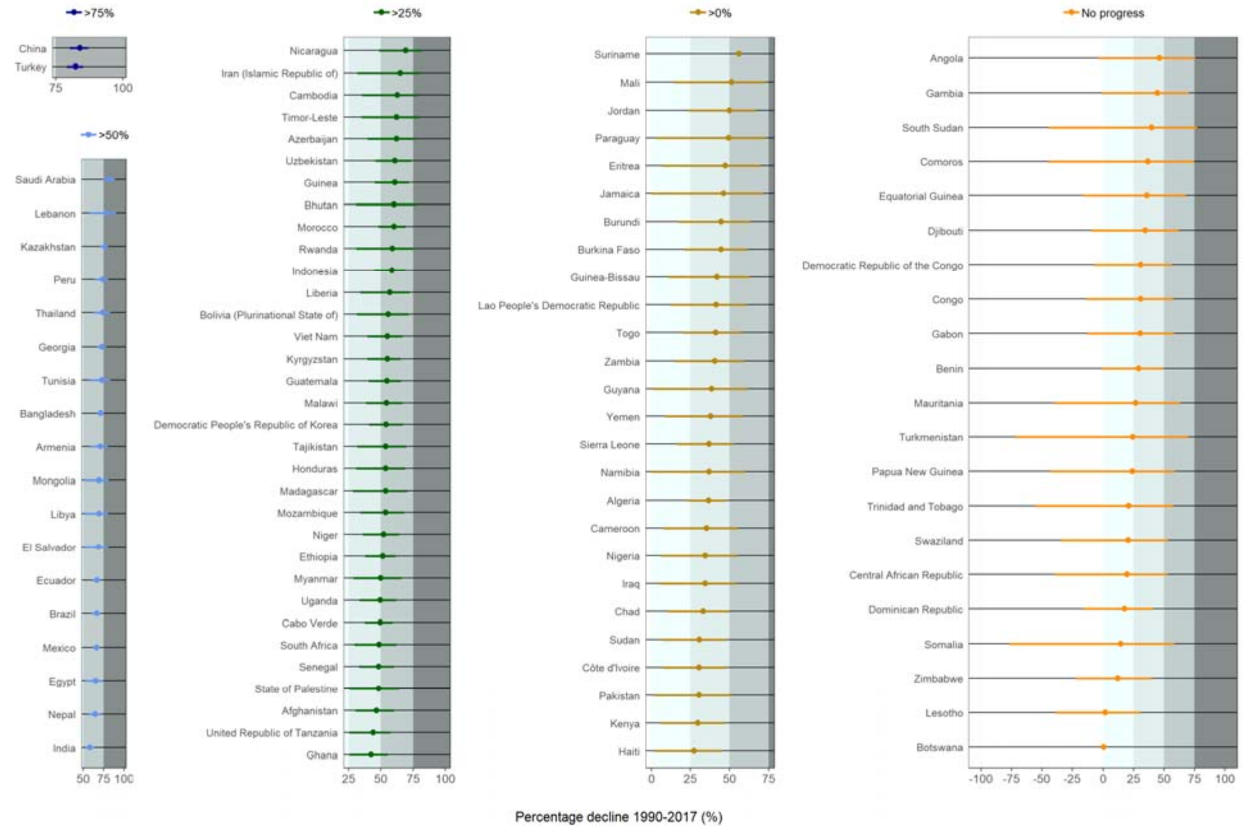

Figure A3: Percentage decline in neonatal mortality rate from 1990-2017 by country for countries with more than 10,000 live births and a neonatal mortality rate of 20 deaths or more per 1,000 live births in 1990.

Note: Point estimate and lower and upper bound of 90% uncertainty interval shown on plots. The lower bounds of 90% UIs provide one-sided probability statements that refer to a 5% or 95% chance. The grey areas indicate the categories the percentage declines of countries are grouped by according to the probabilities to be above or below a category.

Categorization of countries is based on the on the following criteria:

Group 1: Point estimate for percentage decline and 90% UI lower bound >75%. Probability (decline is at least 75%) is at least 95%.

Group 2: Point estimate for percentage decline and 90% UI lower bound >50%. Probability (decline is at least 50%) is at least 95%.

Group 3: Point estimate for percentage decline and 90% UI lower bound >25%. Probability (decline is at least 25%) is at least 95%

Group 4: Point estimate for percentage decline and 90% UI lower bound > 0%. Probability (decline is above 0%) is at least 95%

Group 5: Point estimate for percentage decline and 90% UI lower bound < 0% (Probability (decline is above 0%) is below 95%

Figure A.4: Percentage decline in neonatal mortality rate from 1990-2017 by country

Note: Excludes Dominica and Niue.

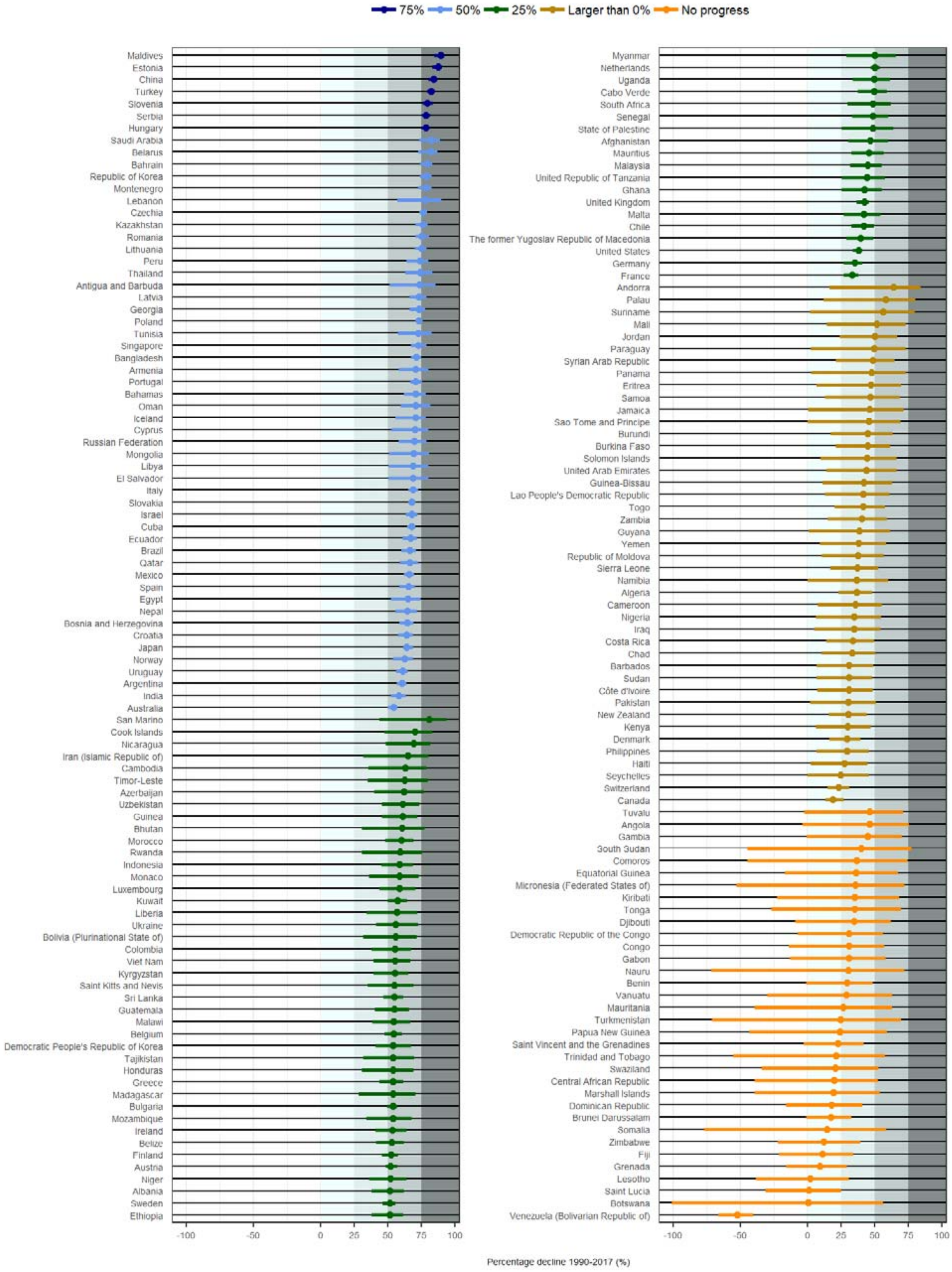
75%
50%
25%
Larger than 0%
No progress
Maldives
Estonia
China
Turkey
Slovenia
Serbia
Hungary
Saudi Arabia
Belarus
Bahrain
Republic of Korea
Montenegro
Lebanon
Czechia
Kazakhstan
Romania
Lithuania
Peru
Thailand
Antigua and Barbuda
Latvia
Georgia
Poland
Tunisia
Singapore
Bangladesh
Armenia
Portugal
Bahamas
Oman
Iceland
Cyprus
Russian Federation
Mongolia
Libya
El Salvador
Italy
Slovakia
Israel
Cuba
Ecuador
Brazil
Qatar
Mexico
Spain
Egypt
Nepal
Bosnia and Herzegovina
Croatia
Japan
Norway
Uruguay
Argentina
India
Australia
San Marino
Cook Islands
Nicaragua
Iran (Islamic Republic of)
Cambodia
Timor-Leste
Azerbaijan
Uzbekistan
Guinea
Bhutan
Morocco
Rwanda
Indonesia
Monaco
Luxembourg
Kuwait
Liberia
Ukraine
Bolivia (Plurinational State of)
Colombia
Viet Nam
Kyrgyzstan
Saint Kitts and Nevis
Sri Lanka
Guatemala
Malawi
Belgium
Democratic People's Republic of Korea
Tajikistan
Honduras
Greece
Madagascar
Bulgaria
Mozambique
Ireland
Belize
Finland
Austria
Niger
Albania
Sweden
Ethiopia
Myanmar
Netherlands
Uganda
Cabo Verde
South Africa
Senegal
State of Palestine
Afghanistan
Mauritius
Malaysia
United Republic of Tanzania
Ghana
United Kingdom
Malta
Chile
The former Yugoslav Republic of Macedonia
United States
Germany
France
Andorra
Palau
Suriname
Mali
Jordan
Paraguay
Syrian Arab Republic
Panama
Eritrea
Samoa
Jamaica
Sao Tome and Principe
Burundi
Burkina Faso
Solomon Islands
United Arab Emirates
Guinea-Bissau
Lao People's Democratic Republic
Togo
Zambia
Guyana
Yemen
Republic of Moldova
Sierra Leone
Namibia
Algeria
Cameroon
Nigeria
Iraq
Costa Rica
Chad
Barbados
Sudan
Côte d'Ivoire
Pakistan
New Zealand
Kenya
Denmark
Philippines
Haiti
Seychelles
Switzerland
Canada
Tuvalu
Angola
Gambia
South Sudan
Comoros
Equatorial Guinea
Micronesia (Federated States of)
Kiribati
Tonga
Djibouti
Democratic Republic of the Congo
Congo
Gabon
Nauru
Benin
Vanuatu
Mauritania
Turkmenistan
Papua New Guinea
Saint Vincent and the Grenadines
Trinidad and Tobago
Swaziland
Central African Republic
Marshall Islands
Dominican Republic
Brunei Darussalam
Somalia
Zimbabwe
Fiji
Grenada
Lesotho
Saint Lucia
Botswana
Venezuela (Bolivarian Republic of)
-100
-50
0
25
50
75
100
Percentage decline 1990-2017 (%)

Figure A.5: Estimated to expected neonatal mortality rate for countries with outlying ratios in 1990, 2000, 2010 and or 2017.

Note: A country is outlying if the estimated NMR in 1990, 2000, 2010 or 2017 was higher or lower than the expected level given U5MR by at least 10%. The dot shows the median estimate, and the lines give the 90% uncertainty interval. Excludes countries with less than 10,000 live births in 2017.
Symbols indicate outlier status for a year: *NMR/U5MR ratio outlying for 1990. ^ NMR/U5MR ratio outlying for 2000, ~NMR/U5MR ratio outlying for 2010, + NMR/U5MR ratio outlying for 2017.

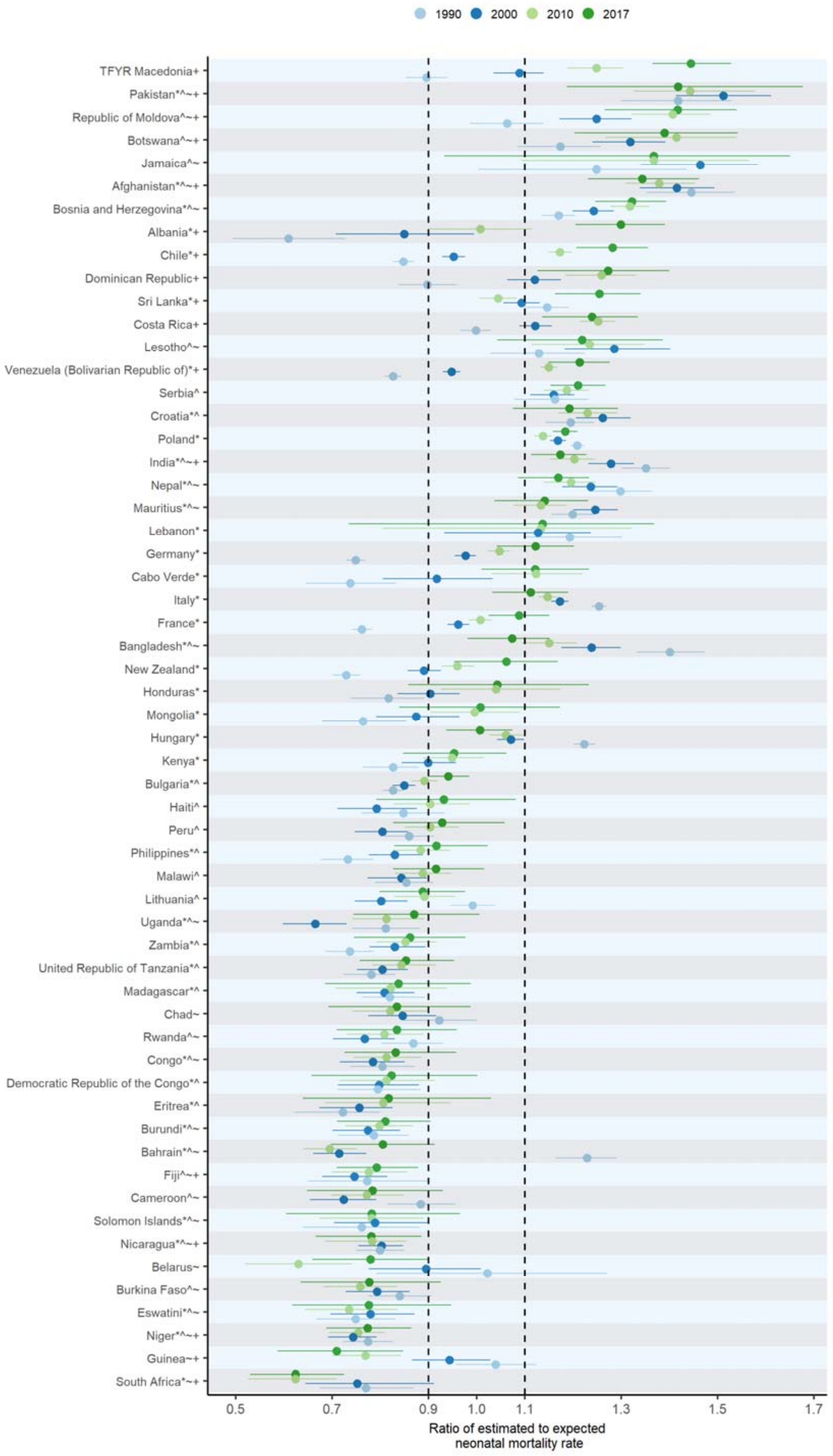
1990 2000 2010 2017
TFYR Macedonia+
Pakistan*^~+
Republic of Moldova^~+
Botswana^~+
Jamaica^~
Afghanistan*^~+
Bosnia and Herzegovina*^~
Albania*+
Chile*+
Dominican Republic+
Sri Lanka*+
Costa Rica+
Lesotho^~
Venezuela (Bolivarian Republic of)*+
Serbia^
Croatia*^
Poland*
India*^~+
Nepal*^~
Mauritius*^~
Lebanon*
Germany*
Cabo Verde*
Italy*
France*
Bangladesh*^~
New Zealand*
Honduras*
Mongolia*
Hungary*
Kenya*
Bulgaria*^
Haiti^
Peru^
Philippines*^
Malawi^
Lithuania^
Uganda*^~
Zambia*^
United Republic of Tanzania*^
Madagascar*^
Chad~
Rwanda^~
Congo*^~
Democratic Republic of the Congo*^
Eritrea*^
Burundi*^~
Bahrain*^~
Fiji^~+
Cameroon^~
Solomon Islands*^~
Nicaragua*^~+
Belarus~
Burkina Faso^~
Eswatini*^~
Niger*^~+
Guinea~+
South Africa*~+
0.5 0.7 0.9 1.0 1.1 1.3 1.5 1.7
Ratio of estimated to expected neonatal mortality rate

Figure A.6: Projected neonatal mortality rate by 2030 if current trends continue in countries that need to accelerate progress to achieve the target.

Note: Excludes countries with less than 10,000 live births in 2017.

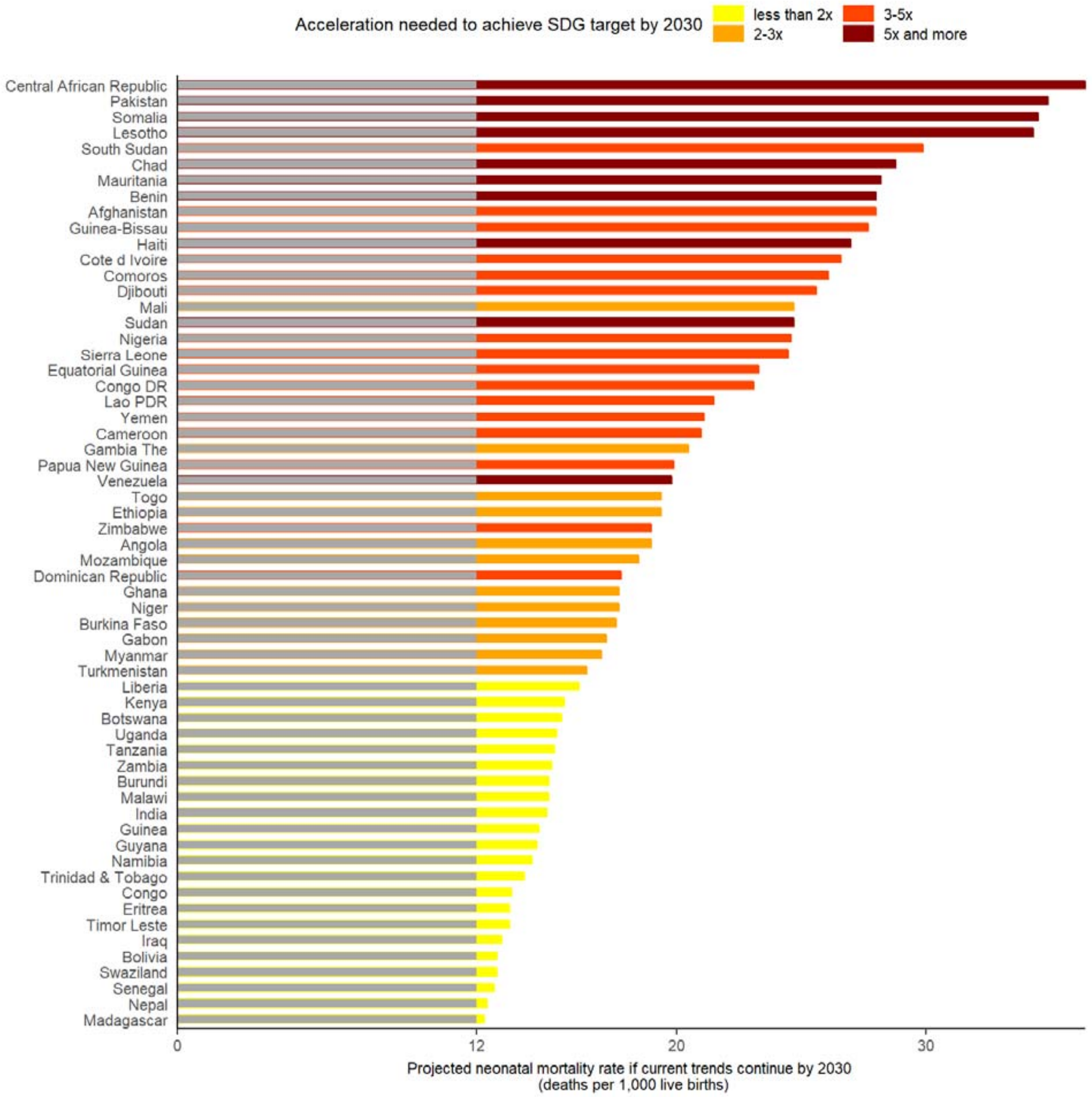

Figure A.7: Required annual rate of reduction in 2017–2030 versus the historical annual rate of reduction in the neonatal mortality rate achieved during 2000–2017, by country

Note: Figure A.4 and A.5 illustrate the mortality rate reductions needed by individual countries to achieve SDG targets by 2030.

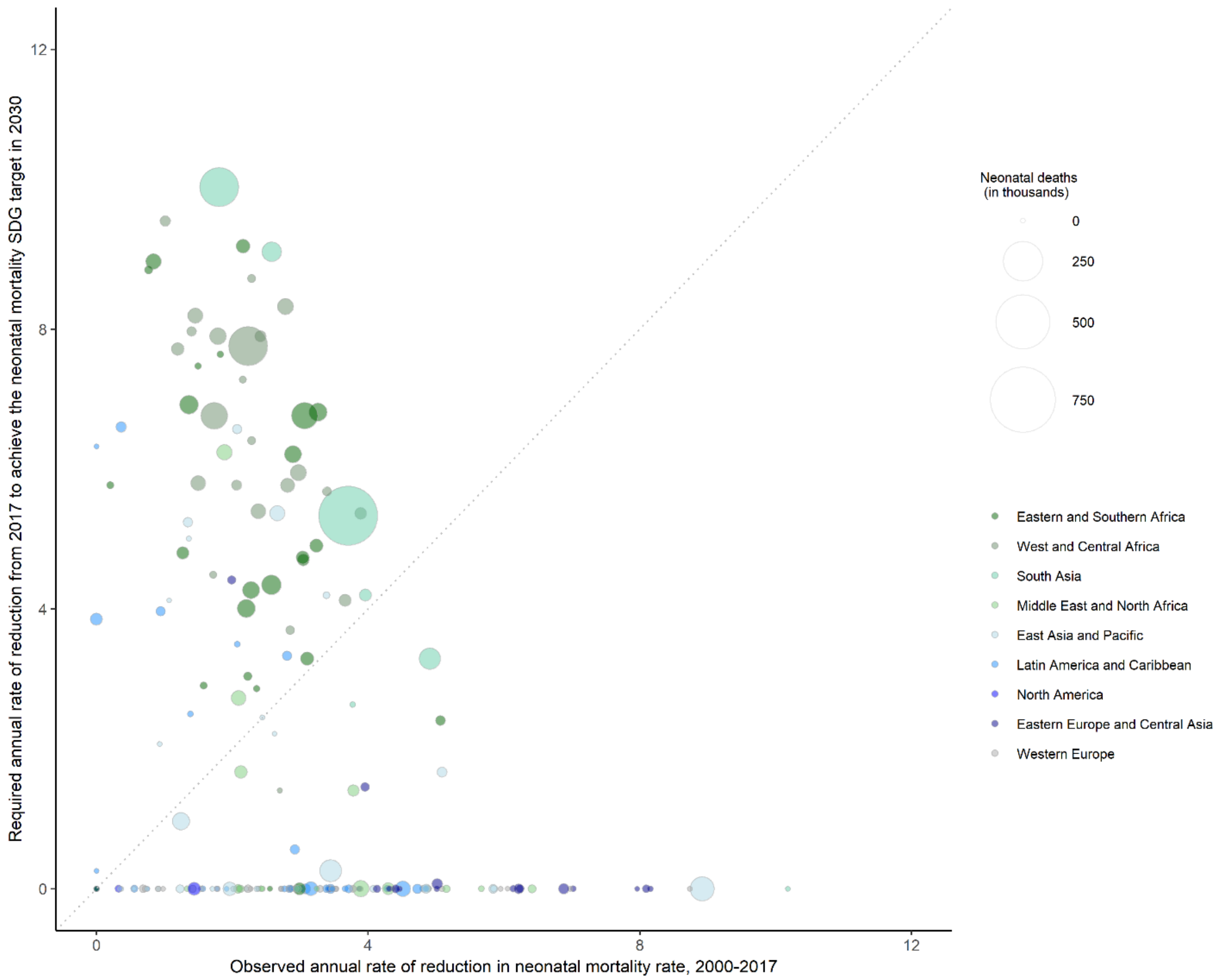

Figure A.8: Required annual rate of reduction in 2017–2030 versus the historical annual rate of reduction in the under-five mortality rate achieved during 2000–2017, by country

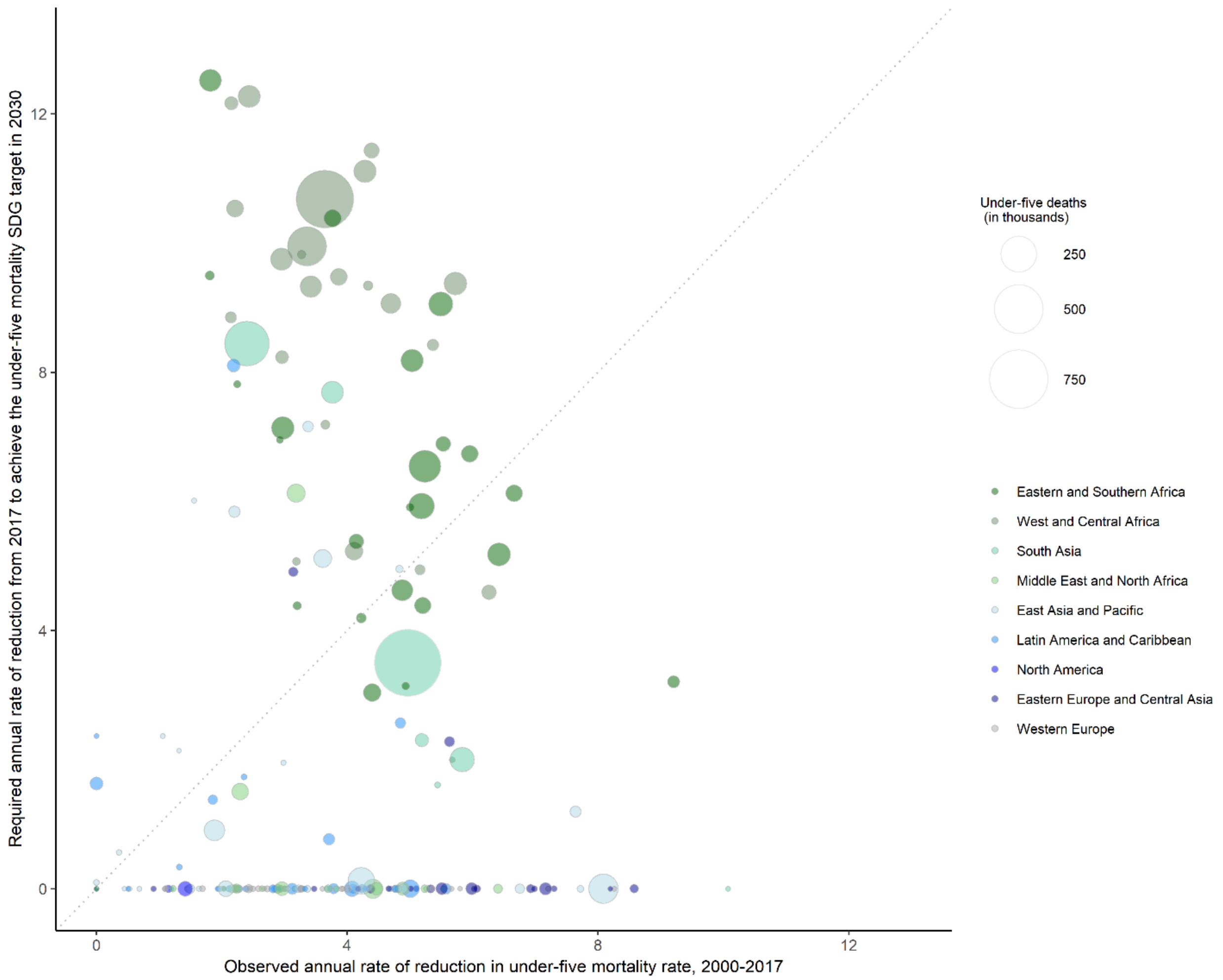

Figure A.9: Neonatal mortality estimates with underlying data, by country

Figure A.10: Ratio of neonatal mortality to under-five mortality rate estimates with underlying data, by country

References

1 Pedersen J, Liu J. Child Mortality Estimation: Appropriate Time Periods for Child Mortality Estimates from Full Birth Histories. Plos Medicine, 2012; 9(8).

2 Walker N, Hill K, Zhao FM. Child Mortality Estimation: Methods Used to Adjust for Bias due to AIDS in Estimating Trends in Under-Five Mortality. Plos Medicine, 2012; 9(8).

3 Alexander, M. and Alkema, L. Global Estimation of Neonatal Mortality using a Bayesian Hierarchical Splines Regression Model Demographic Research, 2018, 38, 335-372.

4 Kim H-Y, Kasonde P, Mwiya M, et al. Pregnancy loss and role of infant HIV status on perinatal 29 mortality among HIV-infected women. BMC Pediatr 2012; 12: 138.

[5] CRED. EM-DAT: The CRED International Disaster Database. Belgium: Université Catholique de Louvain. (available from: http://www.emdat.be/).

[6] World Health Organization. WHO methods and data sources for life tables 1990-2015. Global Health Estimates Technical Paper WHO/HIS/IER/GHE/2016.2. WHO, Geneva, May 2016. https://www.who.int/healthinfo/global_burden_disease/LT_method_1990_2015.pdf

[7] United Nations Inter-agency Group for Child Mortality Estimation (UN IGME), Levels & Trends in Child Mortality: Report 2018, Estimates Developed by the UN Inter-agency Group for Child Mortality Estimation. New York: United Nations Children’s Fund, 2018.

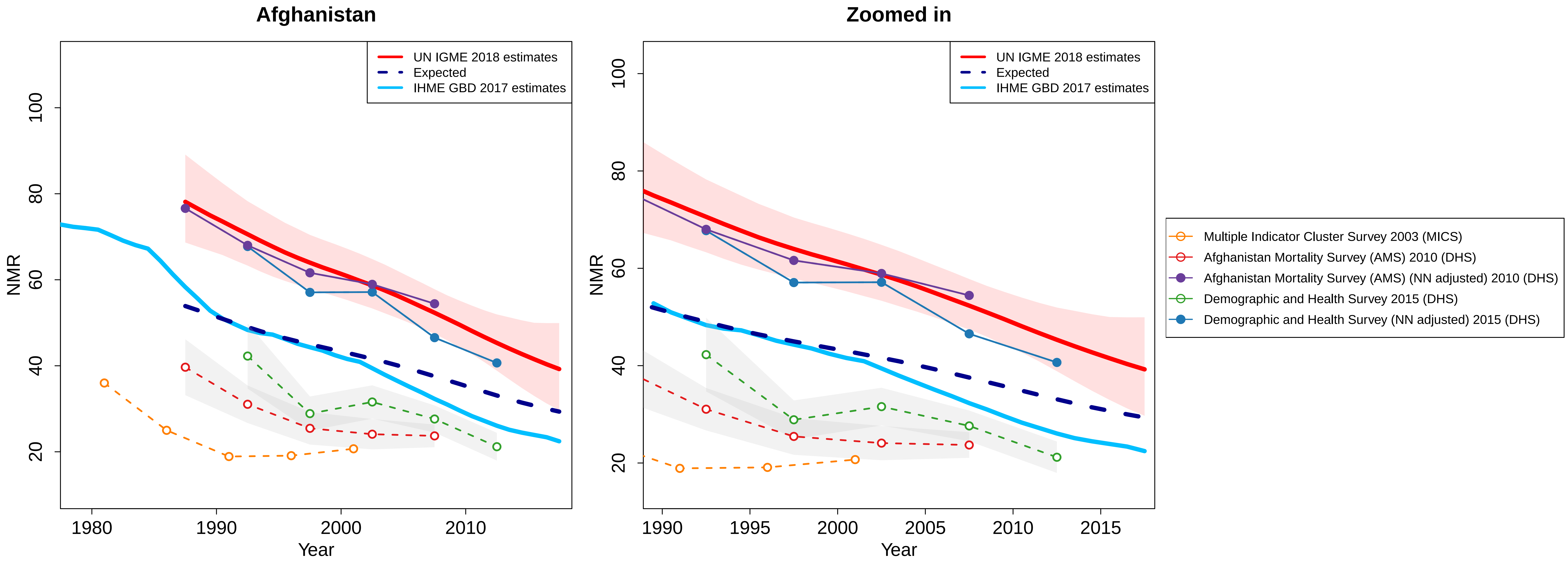
Afghanistan
Zoomed in
UN IGME 2018 estimates
Expected
IHME GBD 2017 estimates
NMR
Year
Multiple Indicator Cluster Survey 2003 (MICS)
Afghanistan Mortality Survey (AMS) 2010 (DHS)
Afghanistan Mortality Survey (AMS) (NN adjusted) 2010 (DHS)
Demographic and Health Survey 2015 (DHS)
Demographic and Health Survey (NN adjusted) 2015 (DHS)

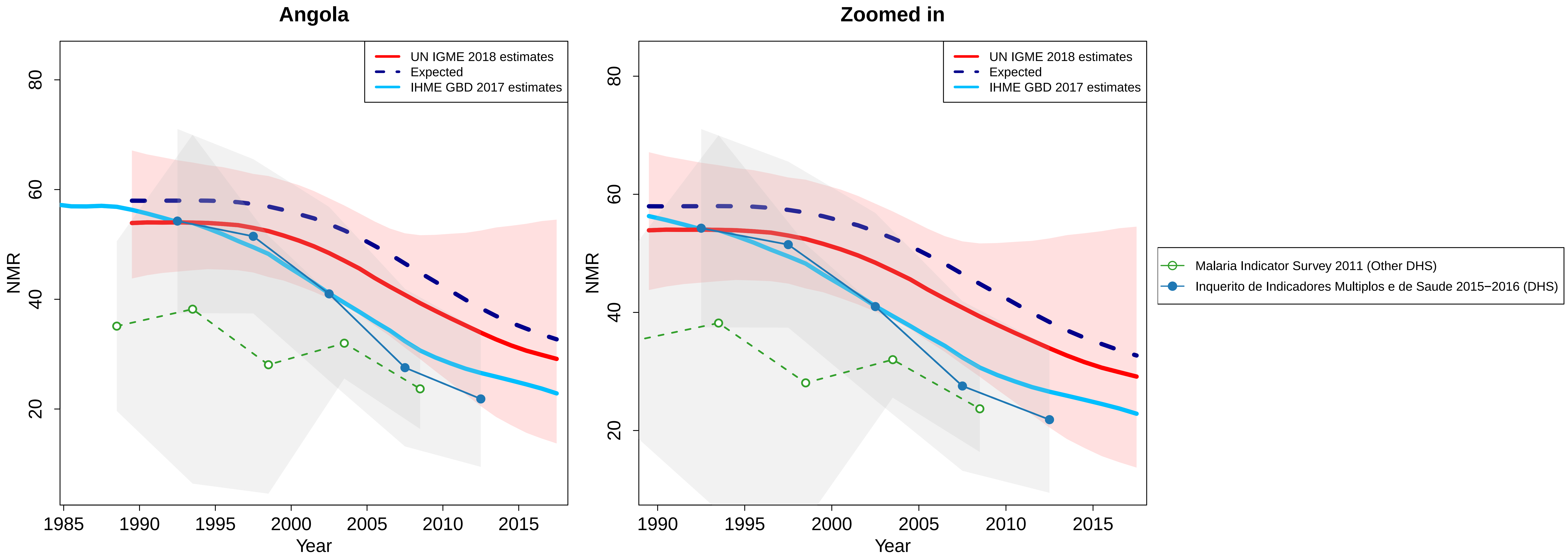
Angola
Zoomed in
UN IGME 2018 estimates
Expected
IHME GBD 2017 estimates
NMR
Year
80
60
40
20
1985
1990
1995
2000
2005
2010
2015
Malaria Indicator Survey 2011 (Other DHS)
Inquerito de Indicadores Multiplos e de Saude 2015−2016 (DHS)

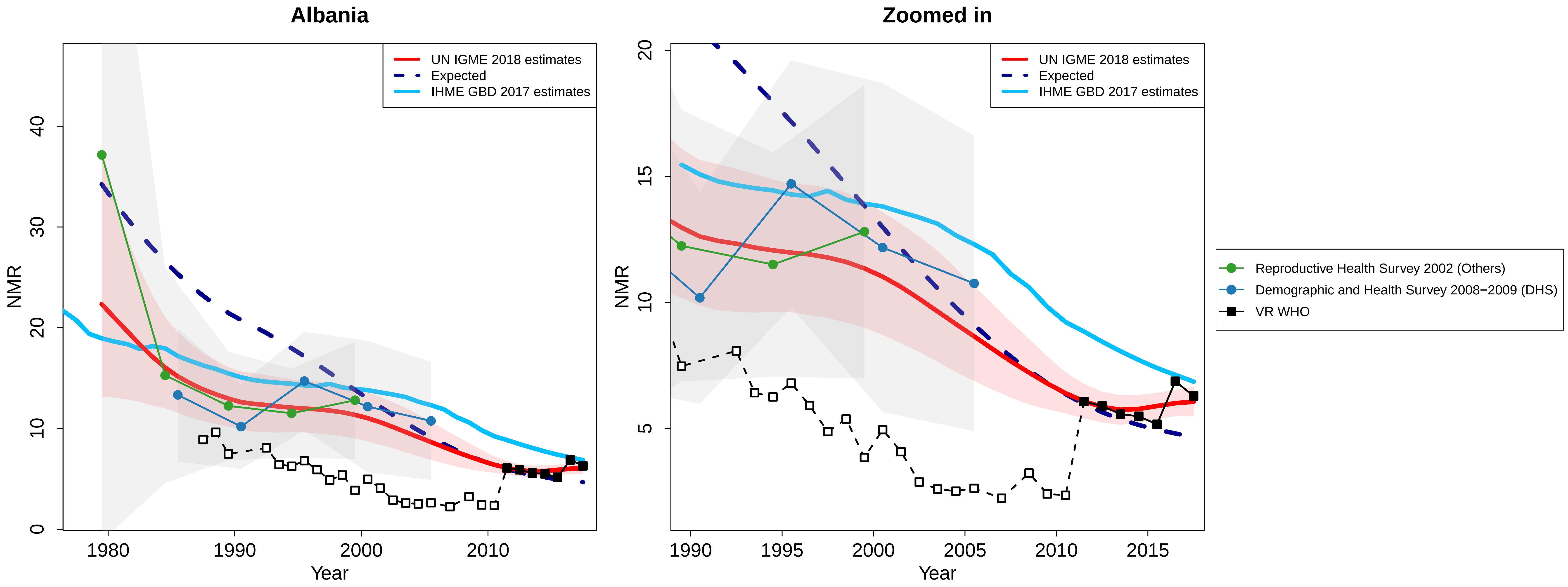

Albania
Zoomed in
UN IGME 2018 estimates
Expected
IHME GBD 2017 estimates
Reproductive Health Survey 2002 (Others)
Demographic and Health Survey 2008−2009 (DHS)
VR WHO
NMR
Year

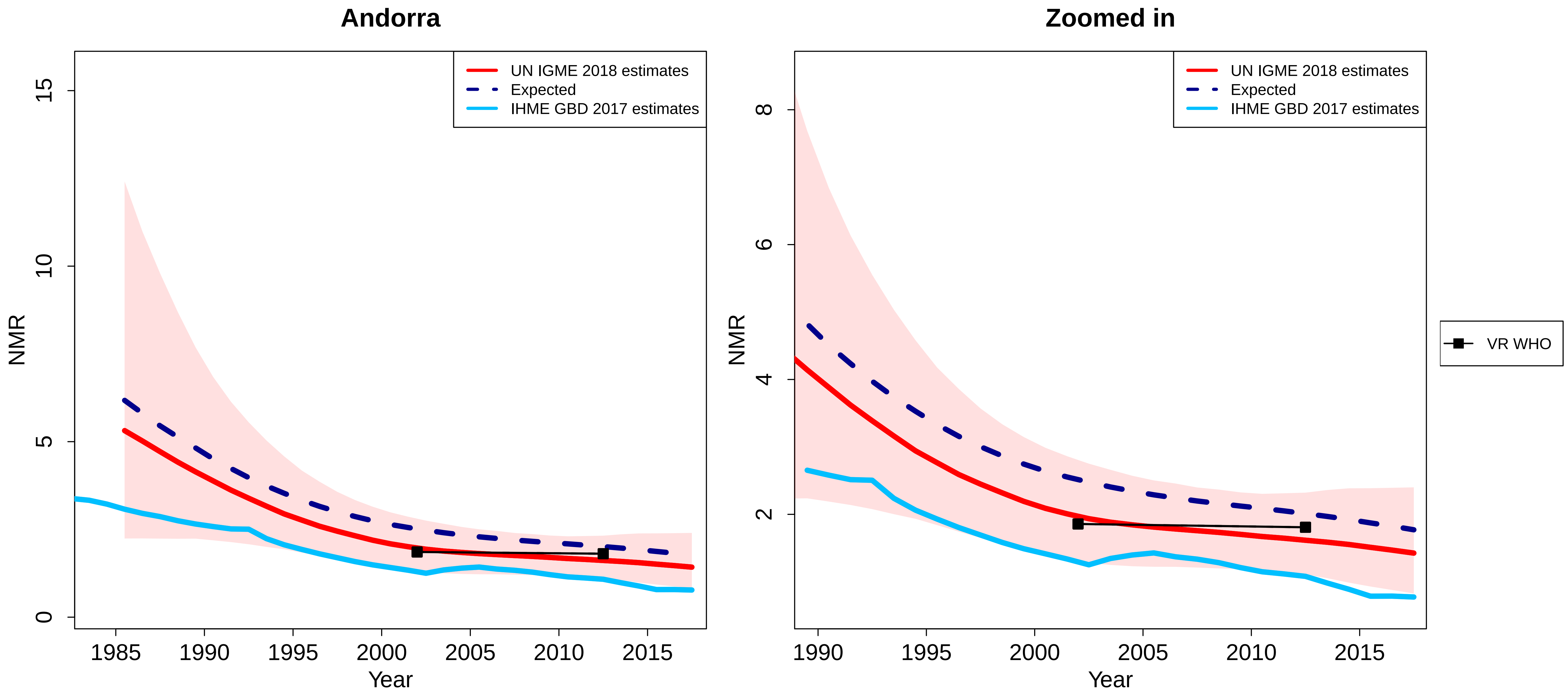
Andorra
UN IGME 2018 estimates
Expected
IHME GBD 2017 estimates
NMR
Year
0
5
10
15
1985
1990
1995
2000
2005
2010
2015
Zoomed in
UN IGME 2018 estimates
Expected
IHME GBD 2017 estimates
NMR
Year
2
4
6
8
1990
1995
2000
2005
2010
2015
VR WHO

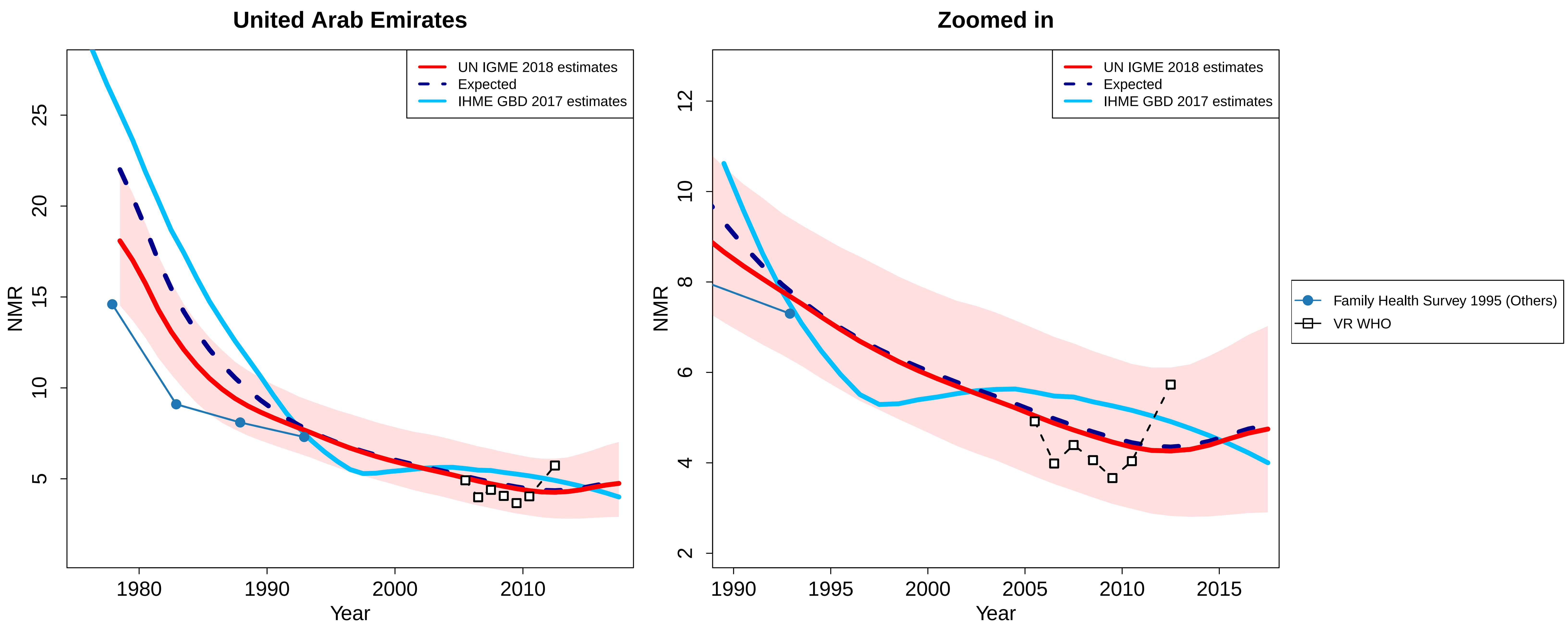
United Arab Emirates
Zoomed in
UN IGME 2018 estimates
Expected
IHME GBD 2017 estimates
Family Health Survey 1995 (Others)
VR WHO
NMR
Year
1980
1990
2000
2010
5
10
15
20
25
1995
2005
2015
2
4
6
8
10
12

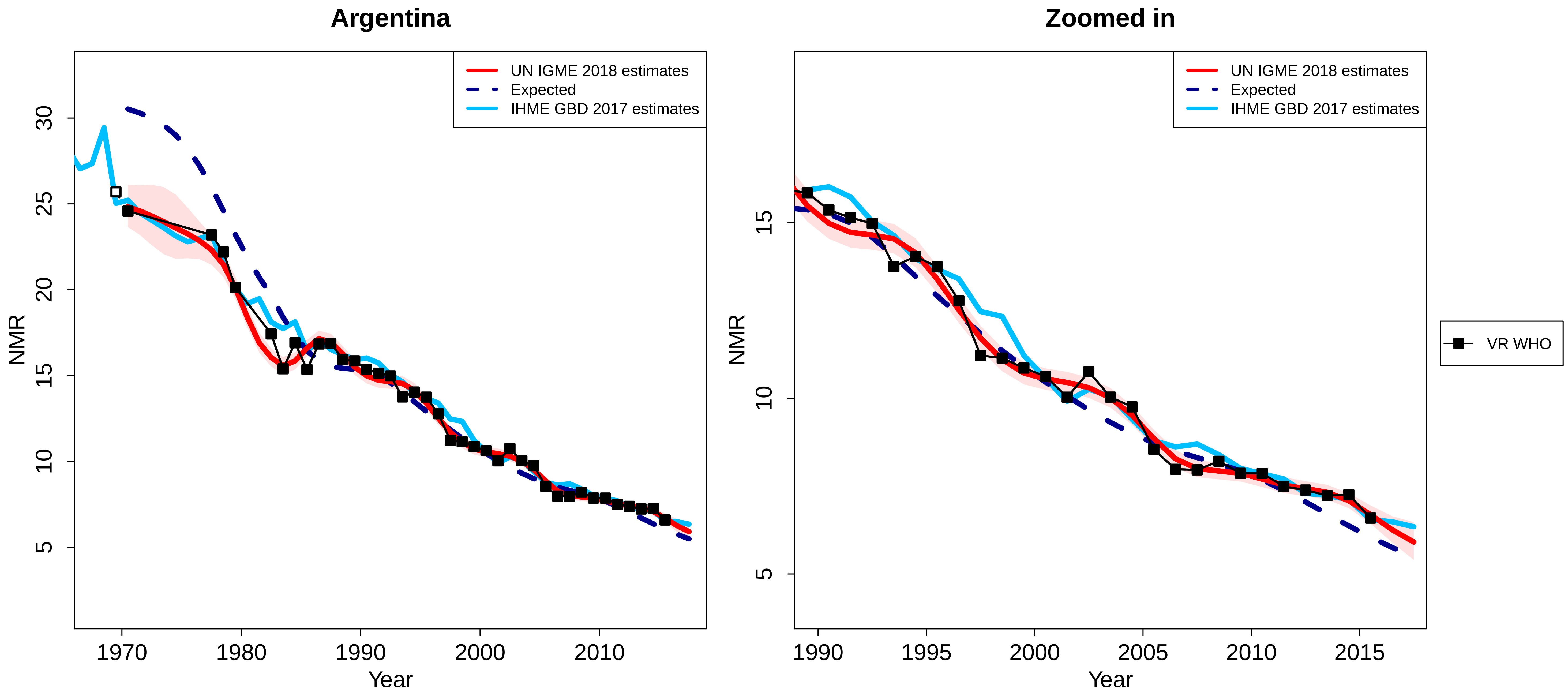

Argentina
Zoomed in
UN IGME 2018 estimates
Expected
IHME GBD 2017 estimates
VR WHO
NMR
Year
5
10
15
20
25
30
1970
1980
1990
2000
2010
1995
2005
2015

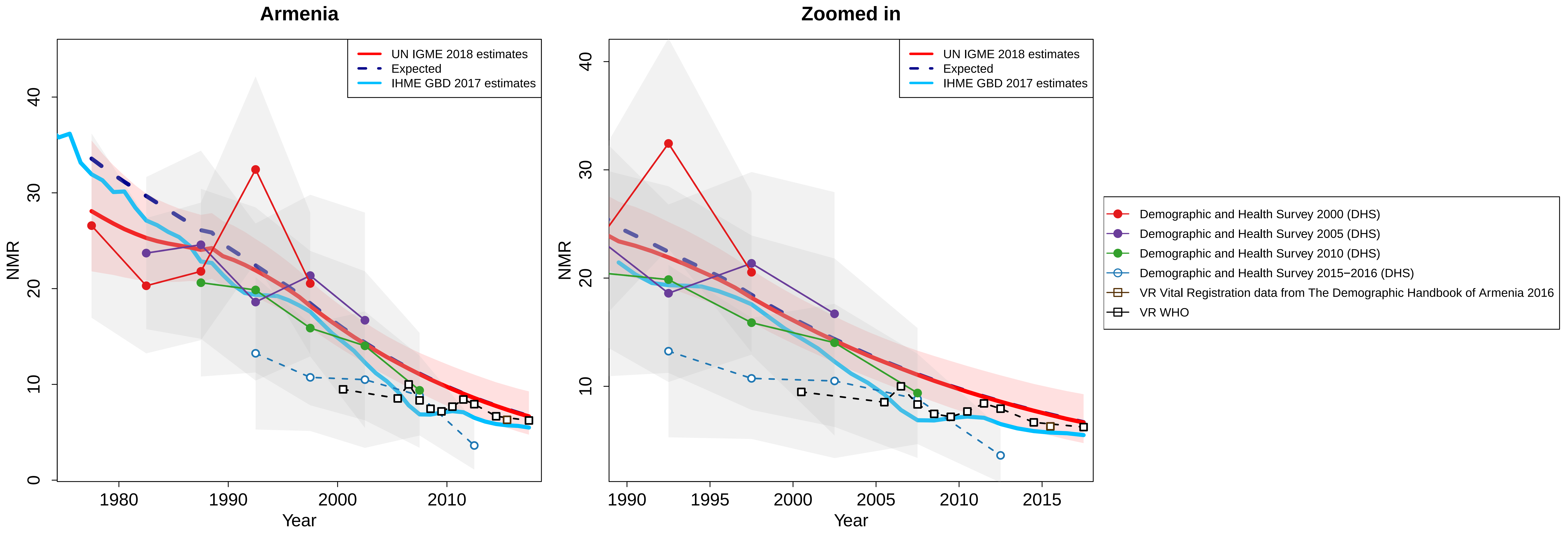
Armenia
Zoomed in
UN IGME 2018 estimates
Expected
IHME GBD 2017 estimates
NMR
Year
0
10
20
30
40
1980
1990
2000
2010
1995
2005
2015
Demographic and Health Survey 2000 (DHS)
Demographic and Health Survey 2005 (DHS)
Demographic and Health Survey 2010 (DHS)
Demographic and Health Survey 2015−2016 (DHS)
VR Vital Registration data from The Demographic Handbook of Armenia 2016
VR WHO

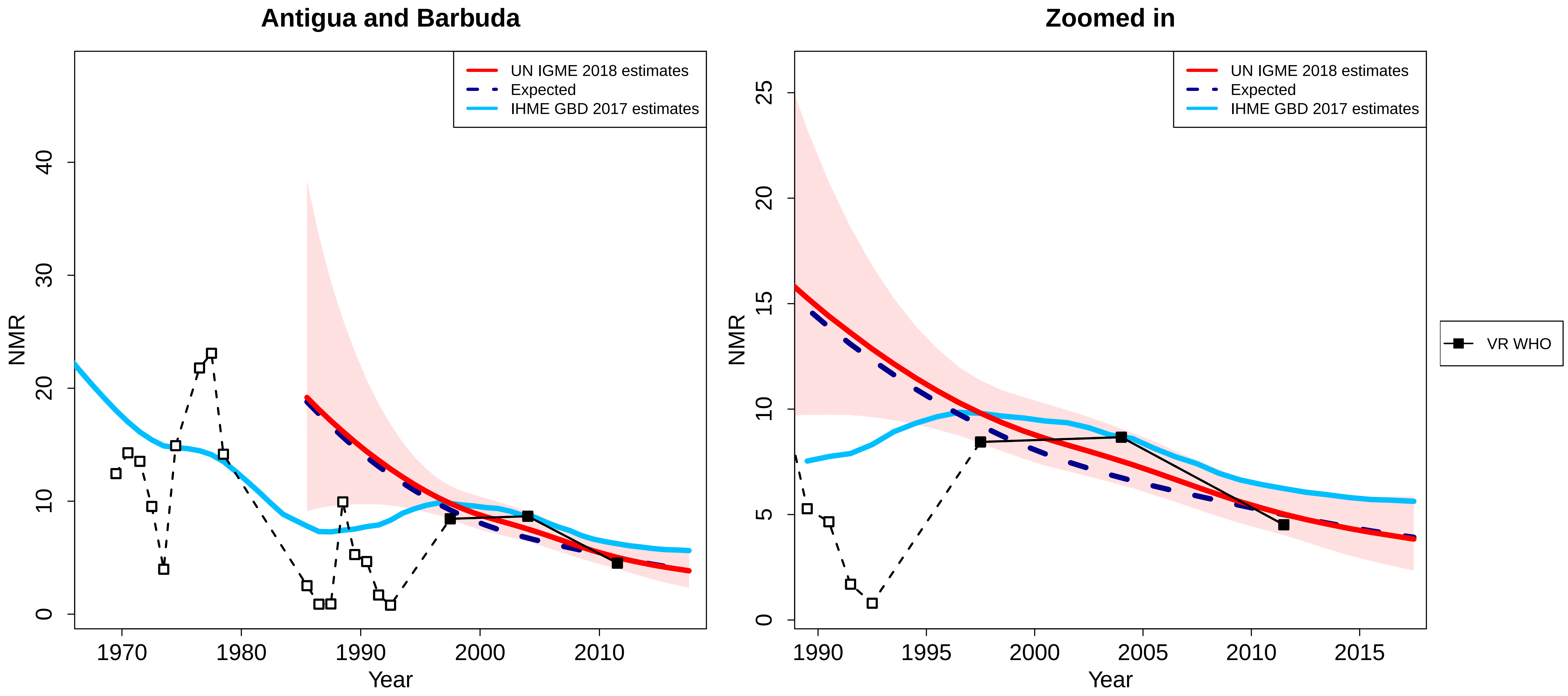

Antigua and Barbuda
Zoomed in
UN IGME 2018 estimates
Expected
IHME GBD 2017 estimates
VR WHO
NMR
Year
0
10
20
30
40
1970
1980
1990
2000
2010
5
15
25
1995
2005
2015

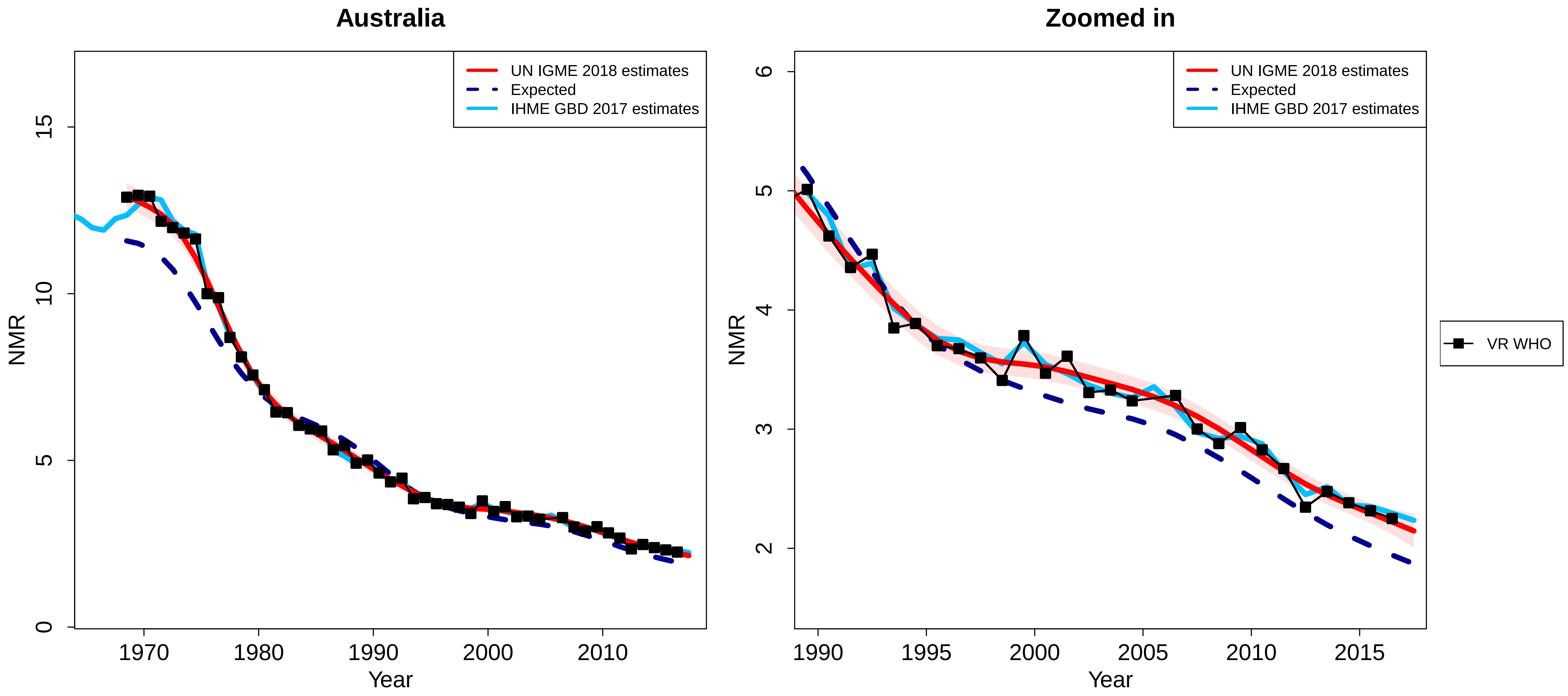
Australia
Zoomed in
UN IGME 2018 estimates
Expected
IHME GBD 2017 estimates
VR WHO
NMR
Year
0
5
10
15
1970
1980
1990
2000
2010
2
3
4
5
6
1995
2005
2015

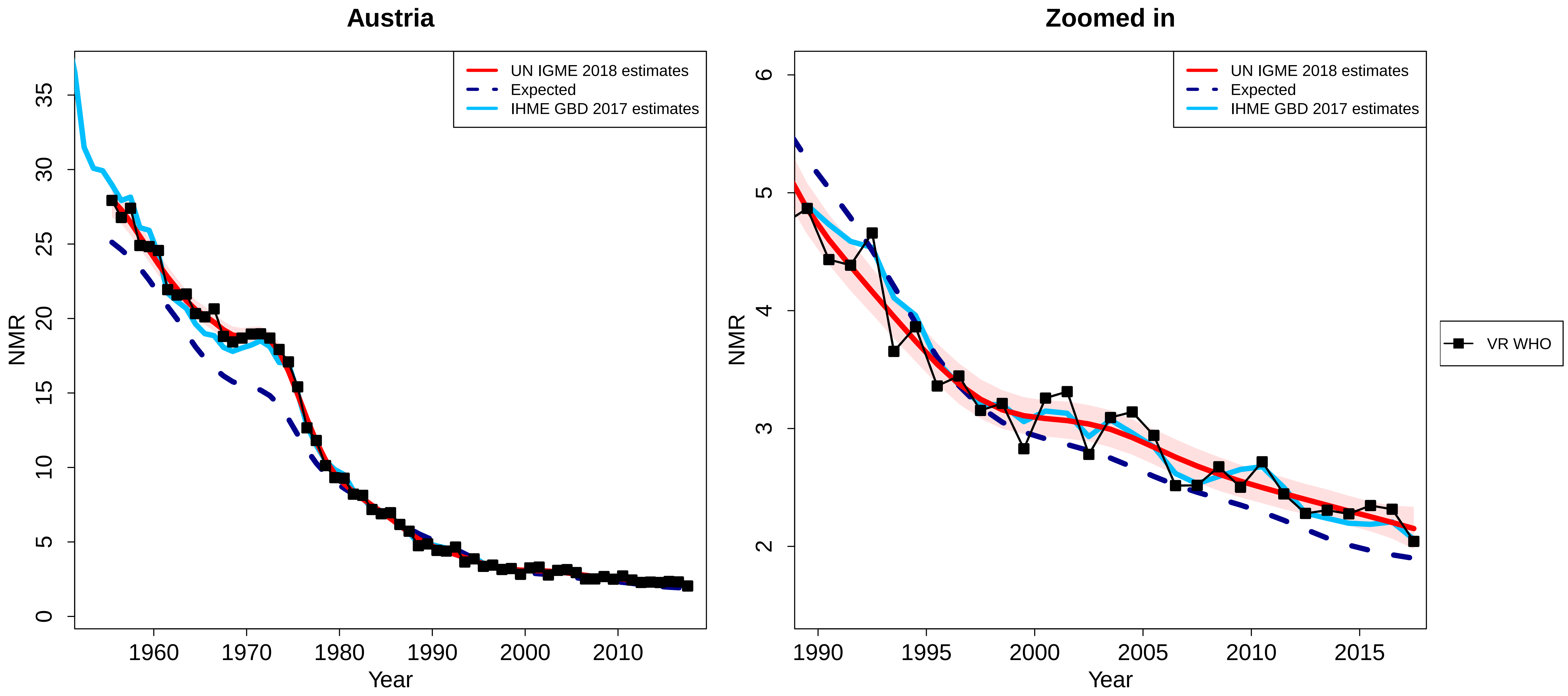
Austria
Zoomed in
UN IGME 2018 estimates
Expected
IHME GBD 2017 estimates
VR WHO
NMR
Year
0
5
10
15
20
25
30
35
1960
1970
1980
1990
2000
2010
2
3
4
5
6
1990
1995
2000
2005
2010
2015

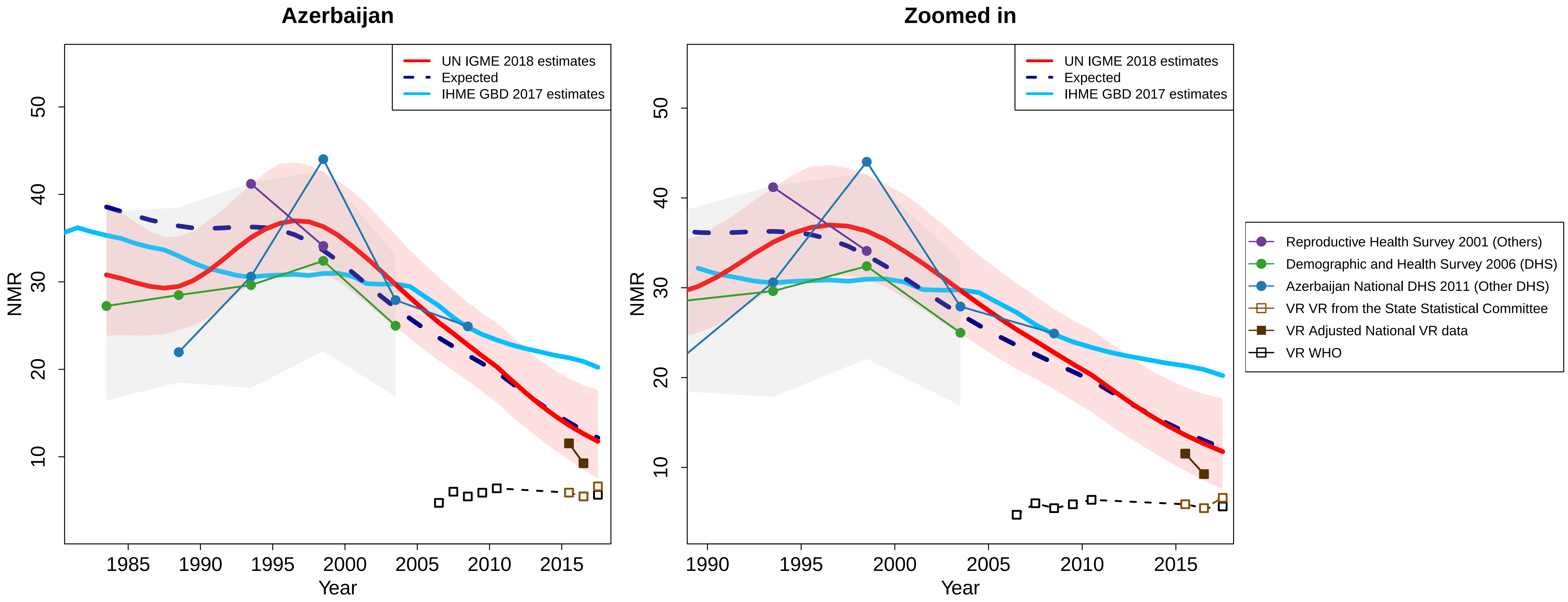

Azerbaijan
Zoomed in
UN IGME 2018 estimates
Expected
IHME GBD 2017 estimates
NMR
Year
Reproductive Health Survey 2001 (Others)
Demographic and Health Survey 2006 (DHS)
Azerbaijan National DHS 2011 (Other DHS)
VR VR from the State Statistical Committee
VR Adjusted National VR data
VR WHO

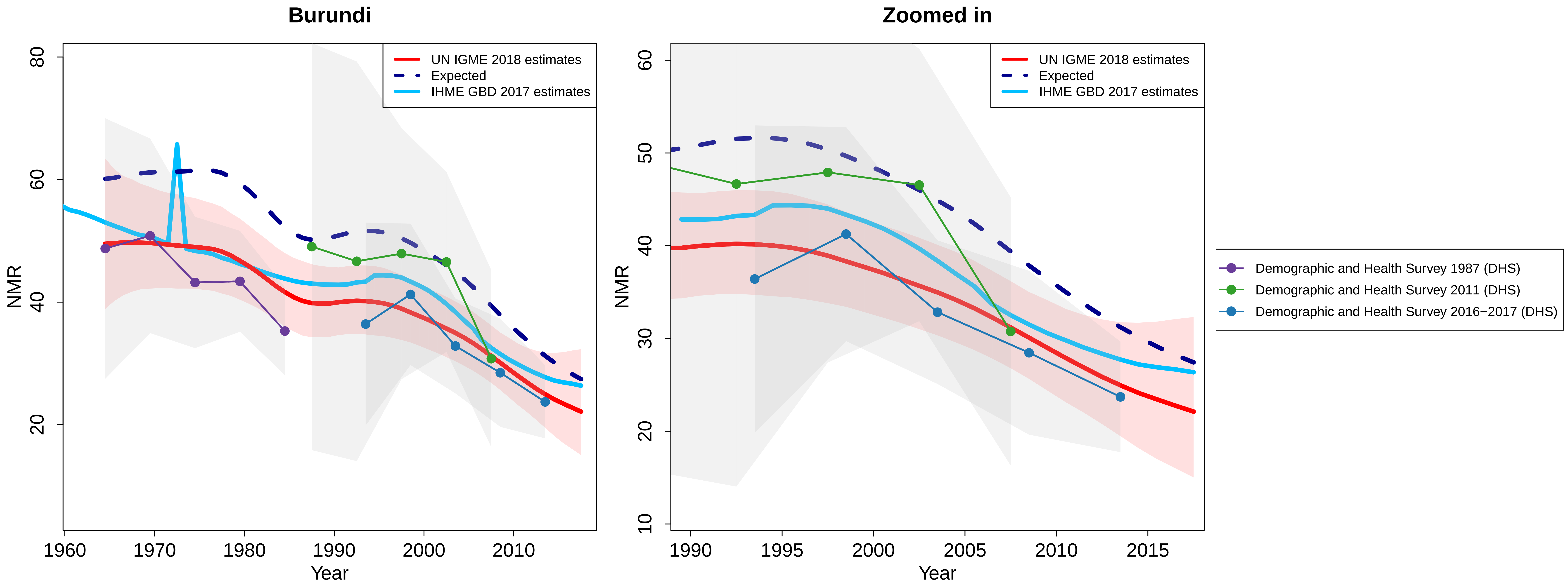

Burundi
Zoomed in
UN IGME 2018 estimates
Expected
IHME GBD 2017 estimates
Demographic and Health Survey 1987 (DHS)
Demographic and Health Survey 2011 (DHS)
Demographic and Health Survey 2016−2017 (DHS)
NMR
Year
1960
1970
1980
1990
2000
2010
20
40
60
80
1990
1995
2000
2005
2010
2015
10
20
30
40
50
60

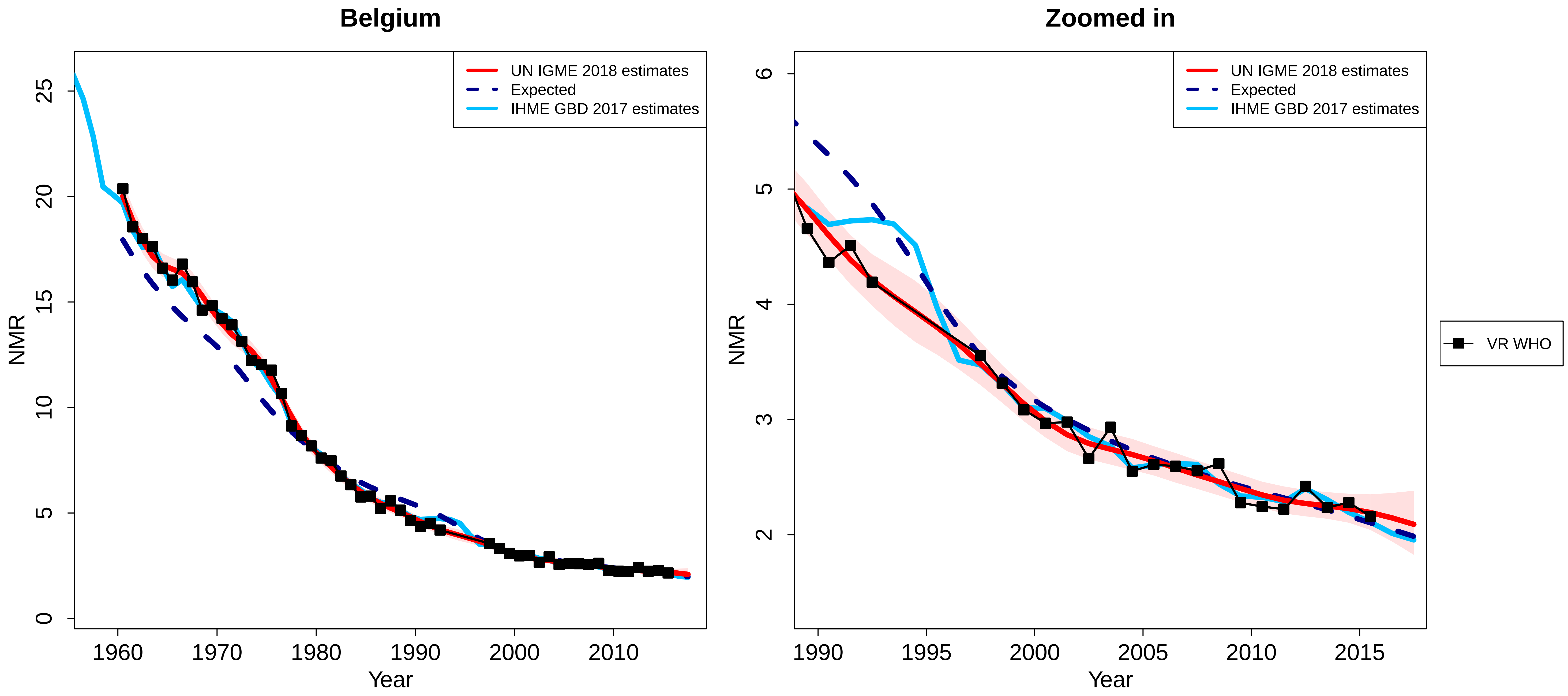

Belgium
UN IGME 2018 estimates
Expected
IHME GBD 2017 estimates
NMR
Year
0
5
10
15
20
25
1960
1970
1980
1990
2000
2010
Zoomed in
UN IGME 2018 estimates
Expected
IHME GBD 2017 estimates
NMR
Year
2
3
4
5
6
1990
1995
2000
2005
2010
2015
VR WHO

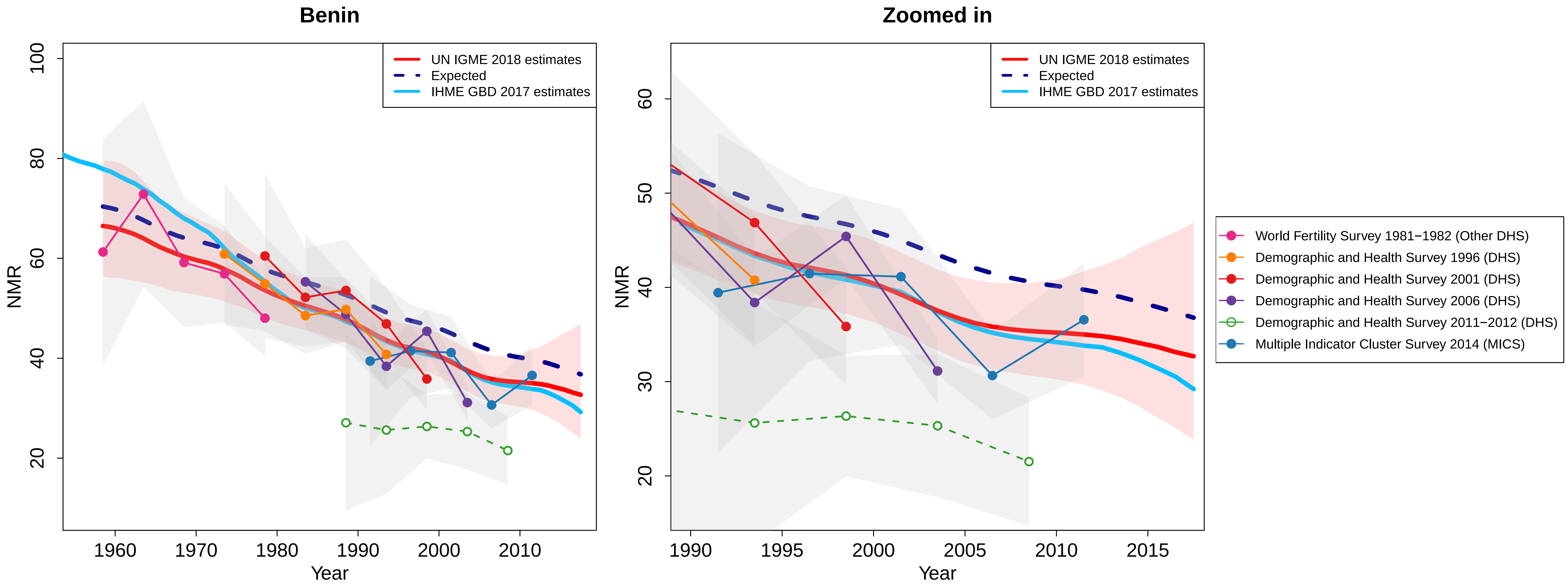

Benin
Zoomed in
UN IGME 2018 estimates
Expected
IHME GBD 2017 estimates
NMR
Year
World Fertility Survey 1981−1982 (Other DHS)
Demographic and Health Survey 1996 (DHS)
Demographic and Health Survey 2001 (DHS)
Demographic and Health Survey 2006 (DHS)
Demographic and Health Survey 2011−2012 (DHS)
Multiple Indicator Cluster Survey 2014 (MICS)

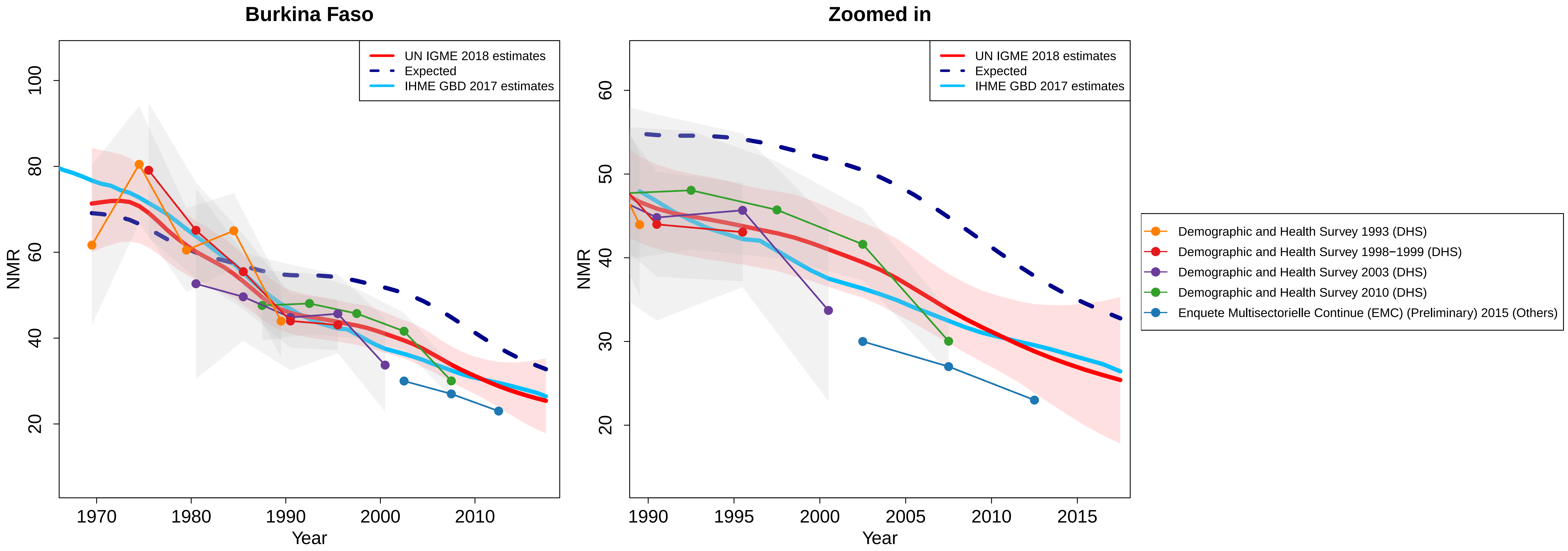

Burkina Faso
Zoomed in
UN IGME 2018 estimates
Expected
IHME GBD 2017 estimates
NMR
Year
Demographic and Health Survey 1993 (DHS)
Demographic and Health Survey 1998−1999 (DHS)
Demographic and Health Survey 2003 (DHS)
Demographic and Health Survey 2010 (DHS)
Enquete Multisectorielle Continue (EMC) (Preliminary) 2015 (Others)

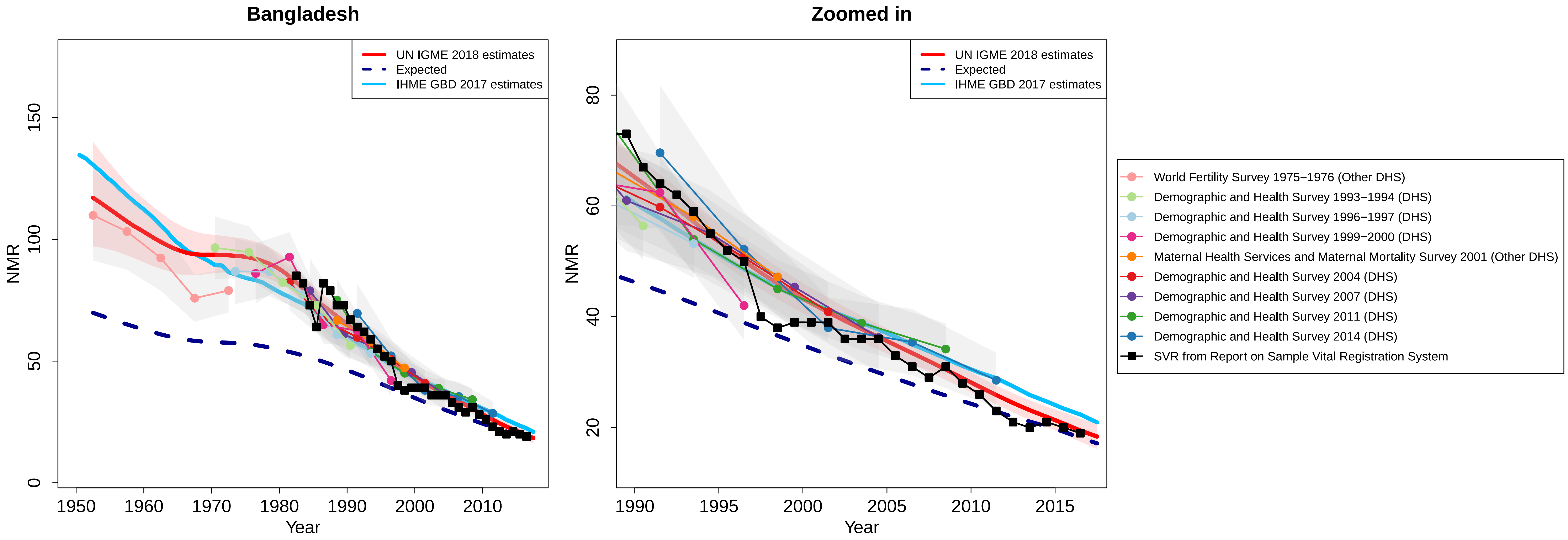
Bangladesh
Zoomed in
UN IGME 2018 estimates
Expected
IHME GBD 2017 estimates
NMR
Year
World Fertility Survey 1975−1976 (Other DHS)
Demographic and Health Survey 1993−1994 (DHS)
Demographic and Health Survey 1996−1997 (DHS)
Demographic and Health Survey 1999−2000 (DHS)
Maternal Health Services and Maternal Mortality Survey 2001 (Other DHS)
Demographic and Health Survey 2004 (DHS)
Demographic and Health Survey 2007 (DHS)
Demographic and Health Survey 2011 (DHS)
Demographic and Health Survey 2014 (DHS)
SVR from Report on Sample Vital Registration System

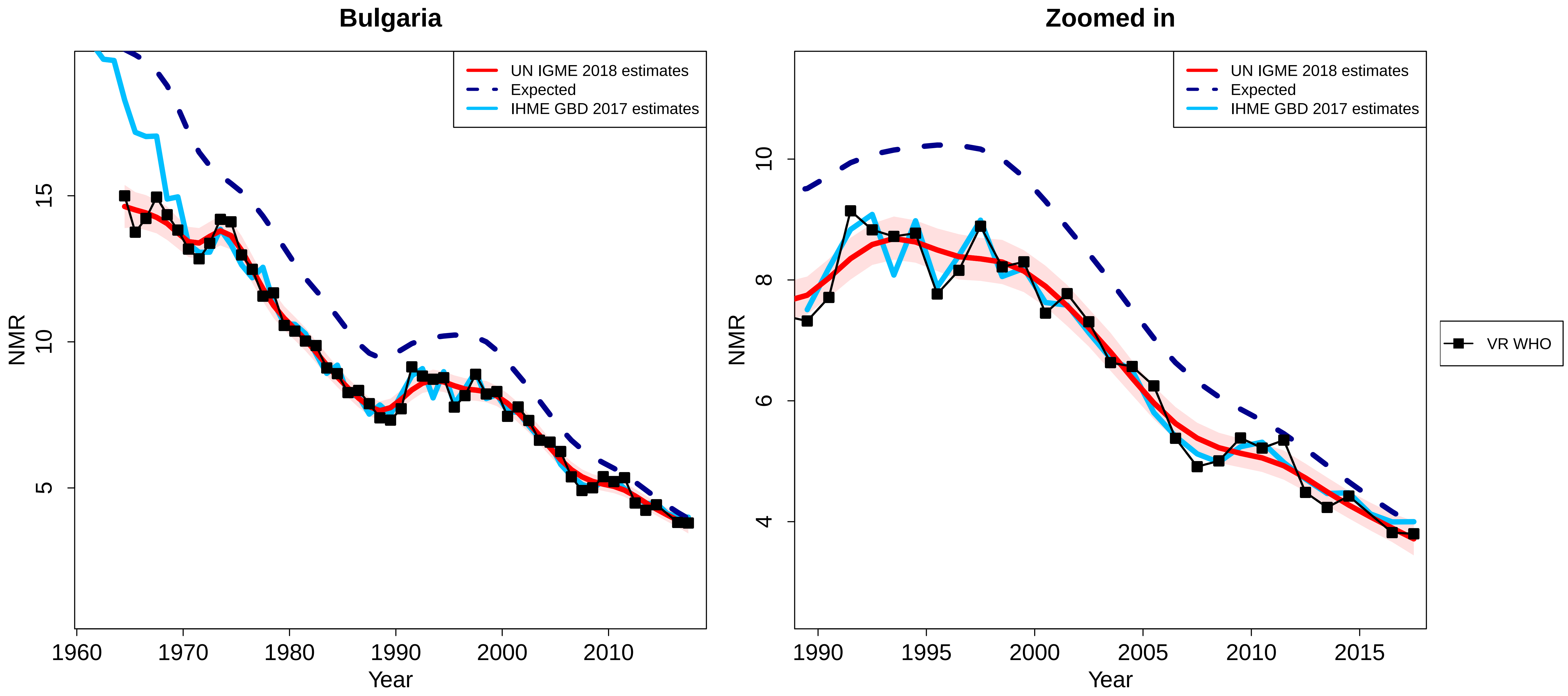
Bulgaria
Zoomed in
UN IGME 2018 estimates
Expected
IHME GBD 2017 estimates
VR WHO
NMR
Year
1960
1970
1980
1990
2000
2010
5
10
15
1995
2005
2015
4
6
8
10

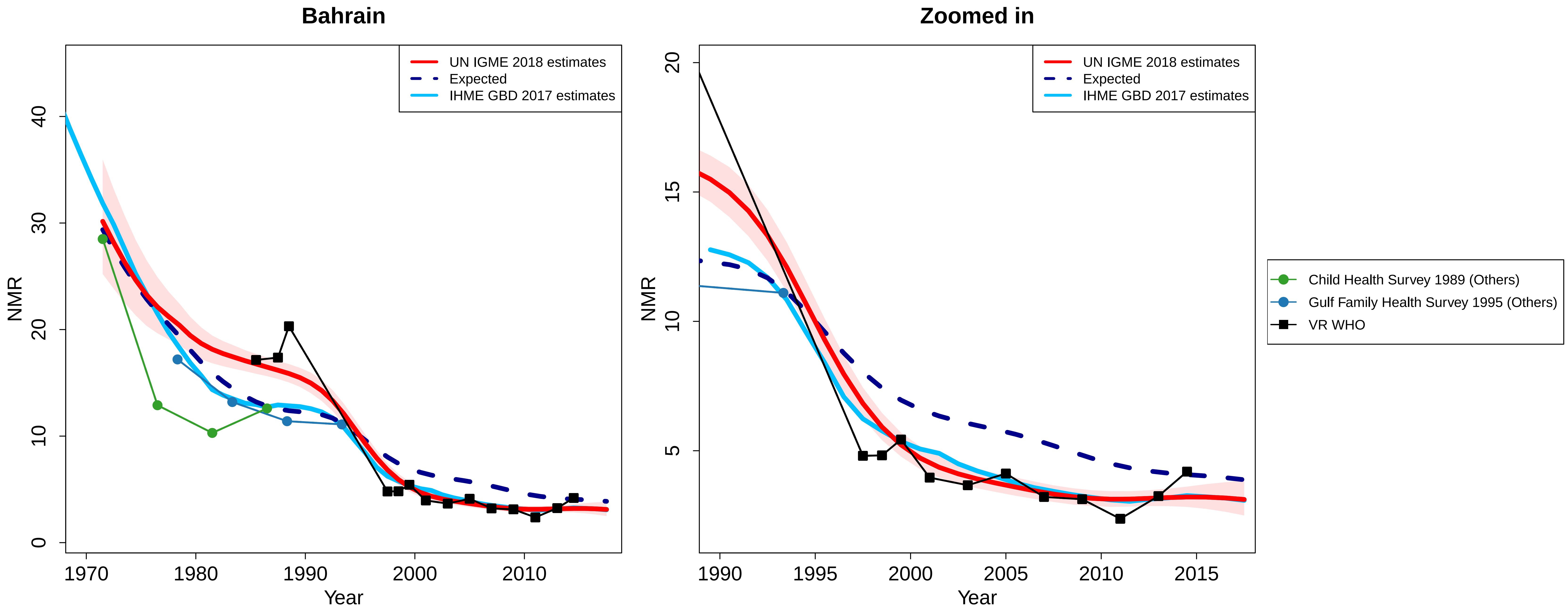
Bahrain
Zoomed in
UN IGME 2018 estimates
Expected
IHME GBD 2017 estimates
NMR
Year
0
10
20
30
40
1970
1980
1990
2000
2010
5
10
15
20
1990
1995
2000
2005
2010
2015
Child Health Survey 1989 (Others)
Gulf Family Health Survey 1995 (Others)
VR WHO

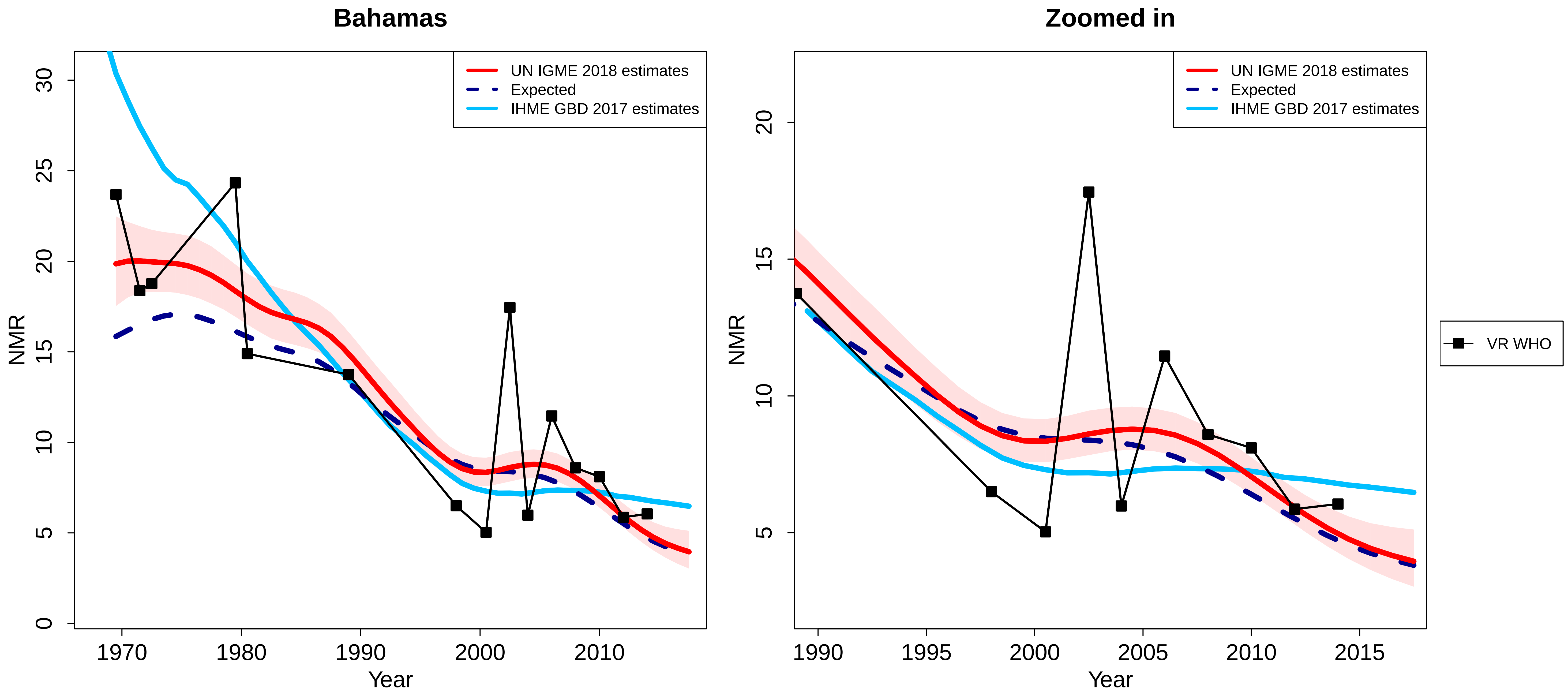

Bahamas
Zoomed in
UN IGME 2018 estimates
Expected
IHME GBD 2017 estimates
NMR
Year
VR WHO

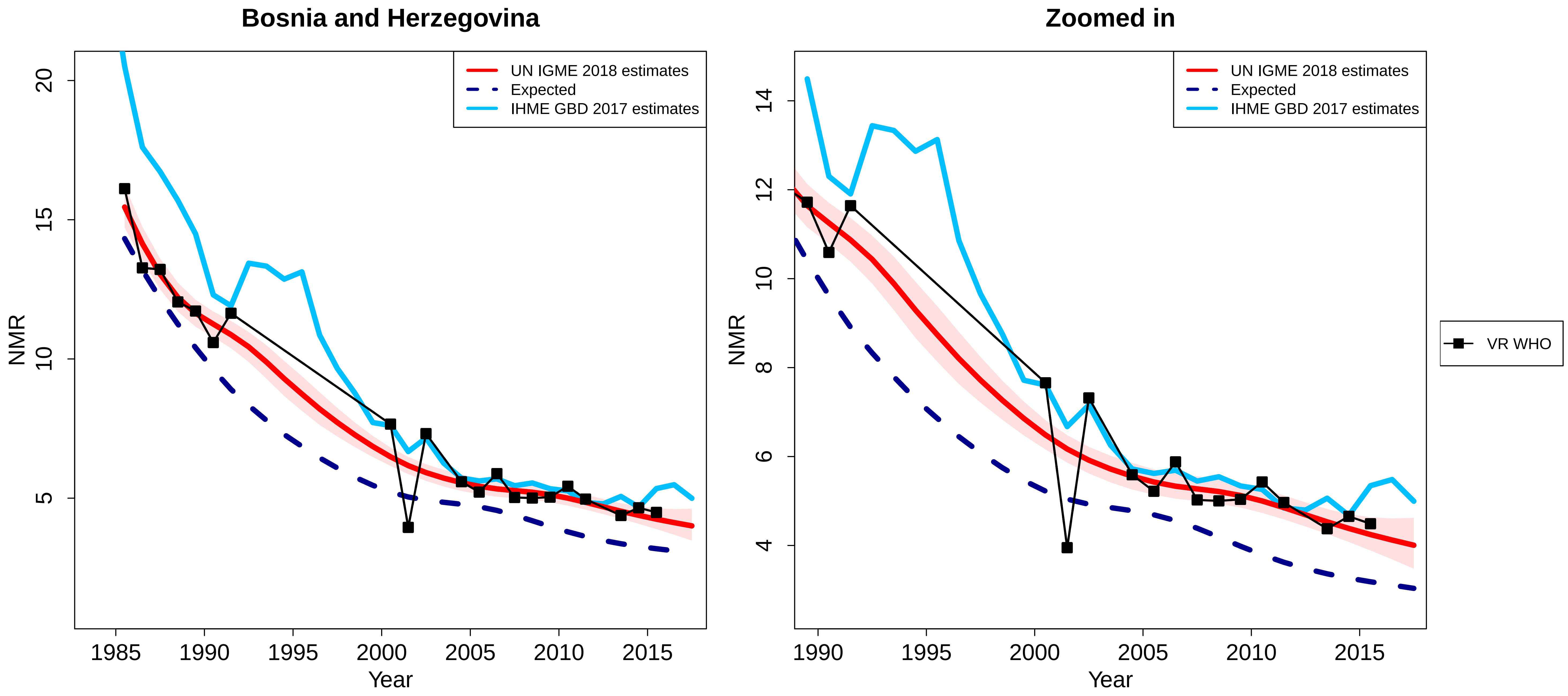
Bosnia and Herzegovina
Zoomed in
UN IGME 2018 estimates
Expected
IHME GBD 2017 estimates
VR WHO
NMR
Year
1985
1990
1995
2000
2005
2010
2015
5
10
15
20
4
6
8
10
12
14

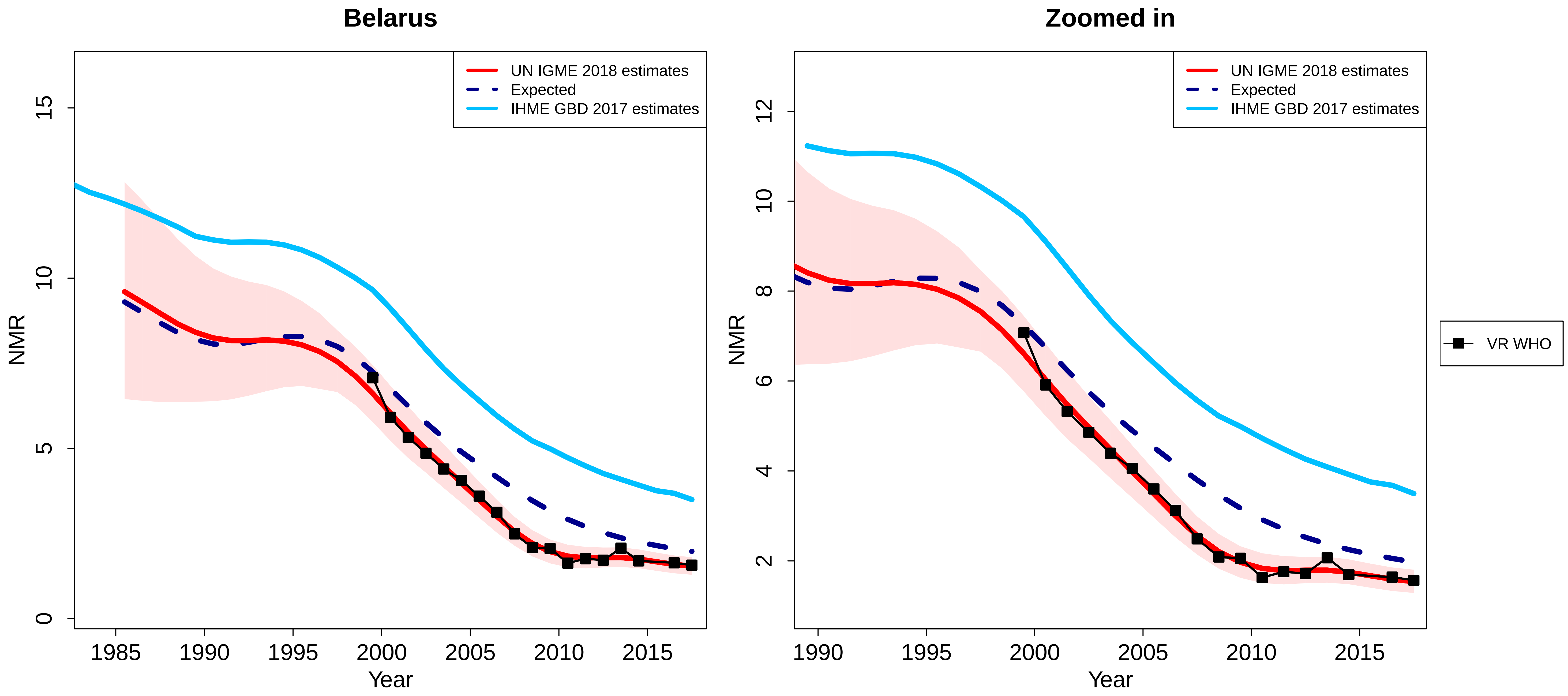

Belarus
Zoomed in
UN IGME 2018 estimates
Expected
IHME GBD 2017 estimates
VR WHO
NMR
Year
0
5
10
15
2
4
6
8
10
12
1985
1990
1995
2000
2005
2010
2015

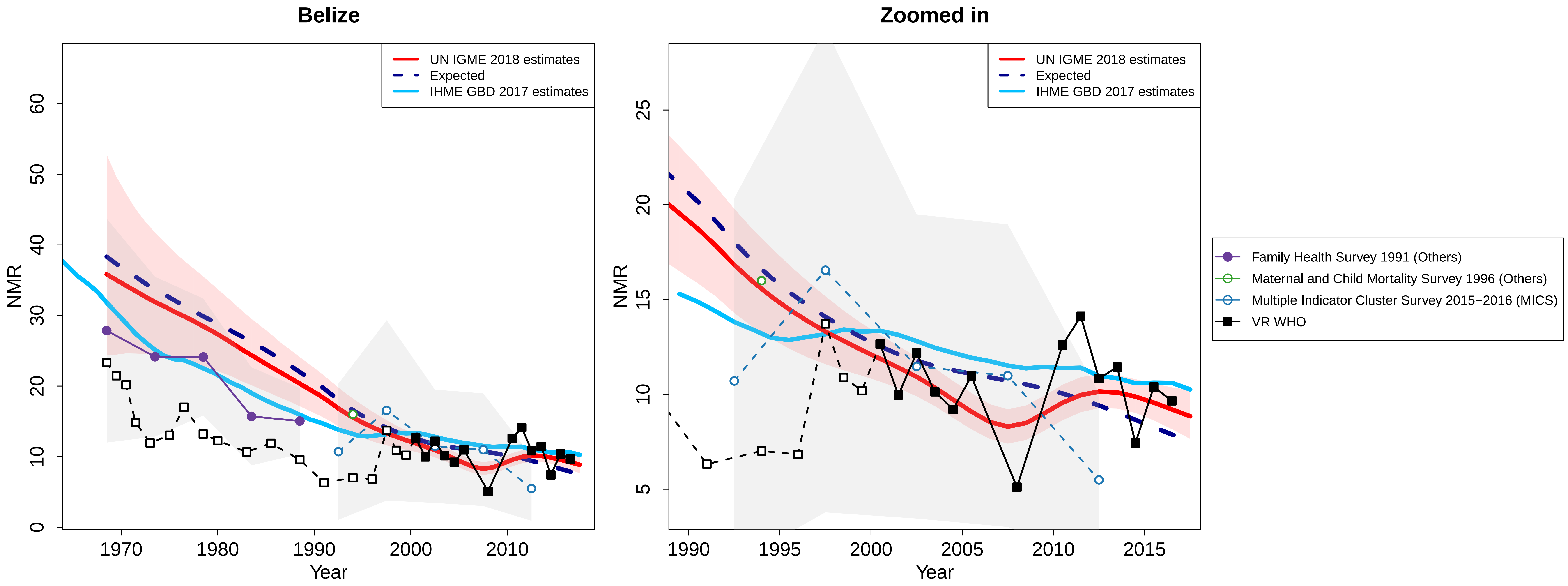
Belize
Zoomed in
UN IGME 2018 estimates
Expected
IHME GBD 2017 estimates
NMR
Year
0
10
20
30
40
50
60
1970
1980
1990
2000
2010
5
10
15
20
25
1995
2005
2015
Family Health Survey 1991 (Others)
Maternal and Child Mortality Survey 1996 (Others)
Multiple Indicator Cluster Survey 2015−2016 (MICS)
VR WHO

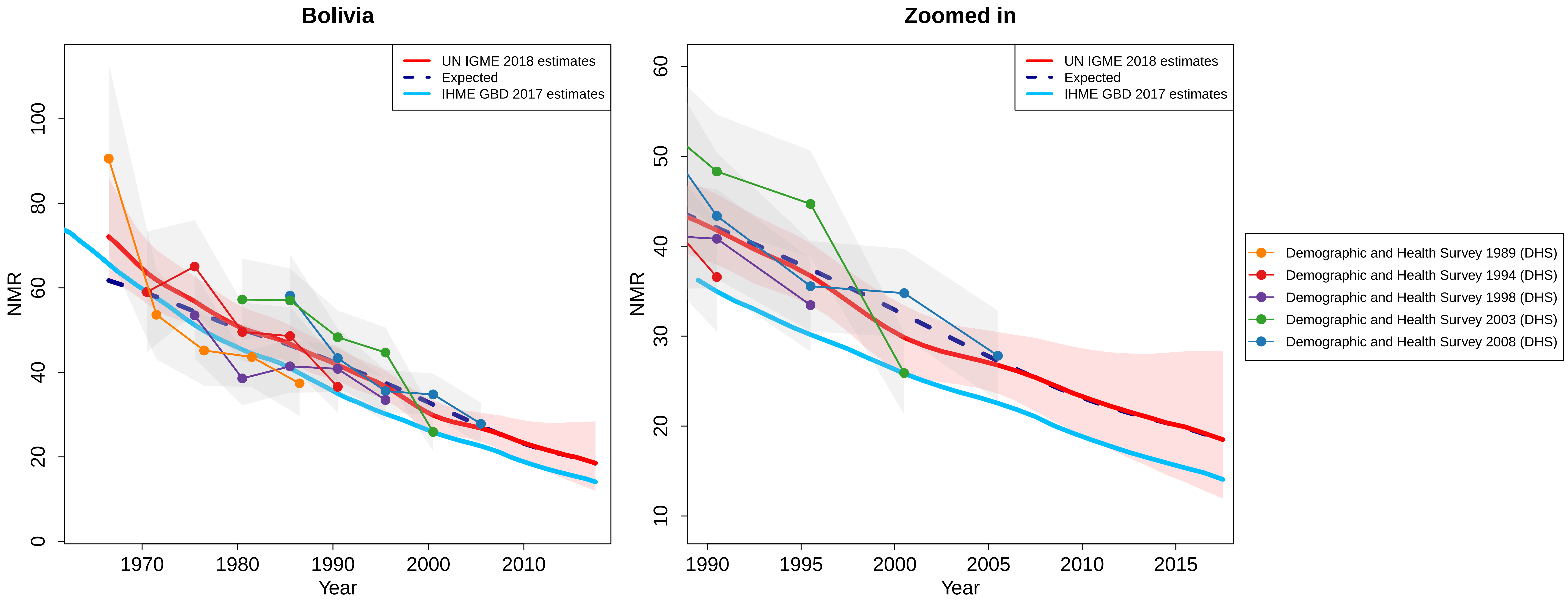

Bolivia
Zoomed in
UN IGME 2018 estimates
Expected
IHME GBD 2017 estimates
NMR
Year
0
20
40
60
80
100
1970
1980
1990
2000
2010
10
20
30
40
50
60
1990
1995
2000
2005
2010
2015
Demographic and Health Survey 1989 (DHS)
Demographic and Health Survey 1994 (DHS)
Demographic and Health Survey 1998 (DHS)
Demographic and Health Survey 2003 (DHS)
Demographic and Health Survey 2008 (DHS)

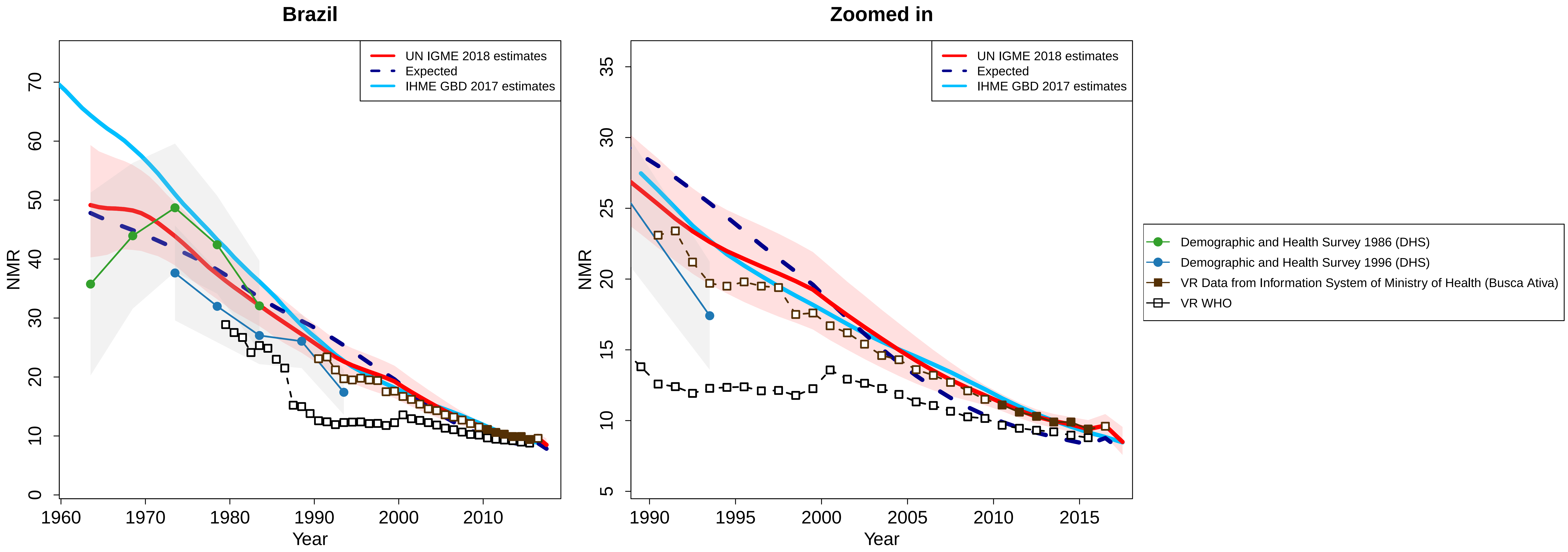

Brazil
Zoomed in
UN IGME 2018 estimates
Expected
IHME GBD 2017 estimates
NMR
Year
Demographic and Health Survey 1986 (DHS)
Demographic and Health Survey 1996 (DHS)
VR Data from Information System of Ministry of Health (Busca Ativa)
VR WHO

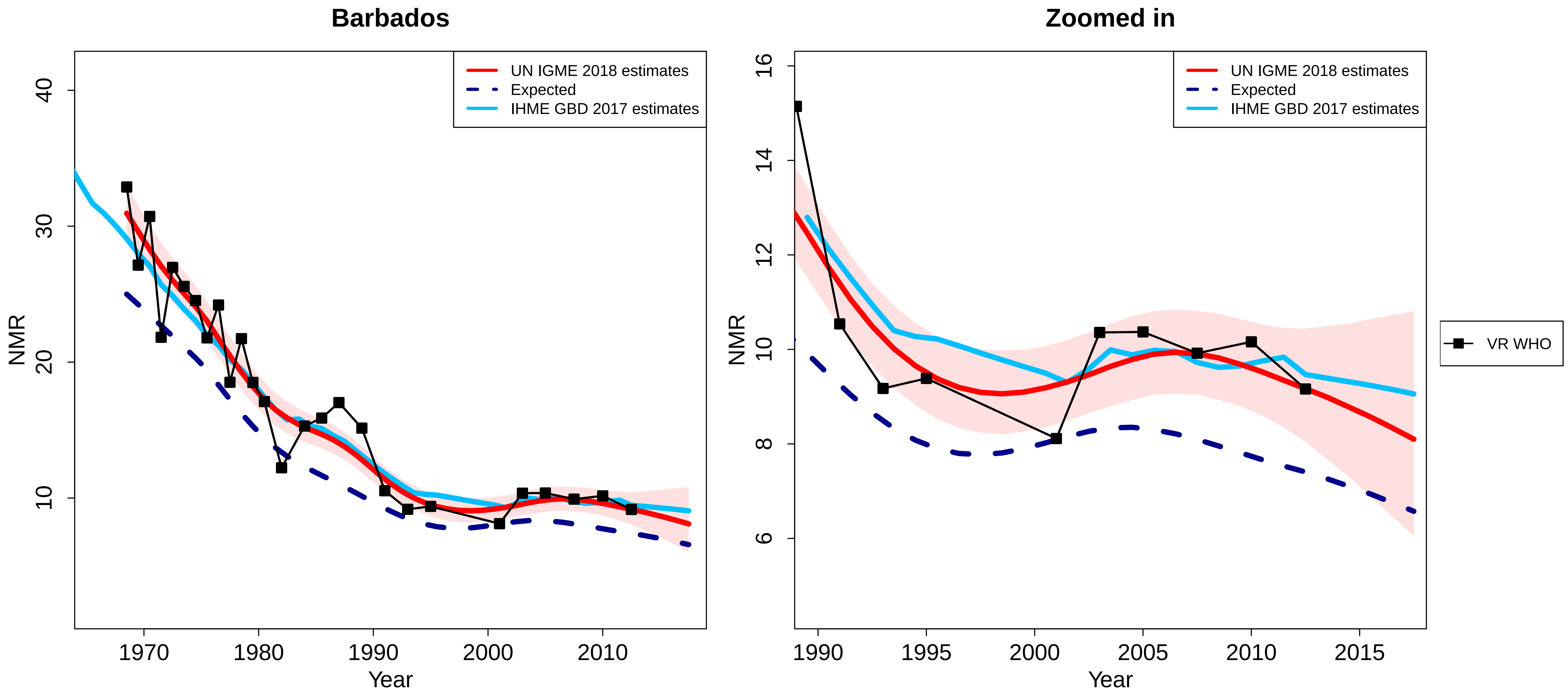

Barbados
Zoomed in
UN IGME 2018 estimates
Expected
IHME GBD 2017 estimates
VR WHO
NMR
Year

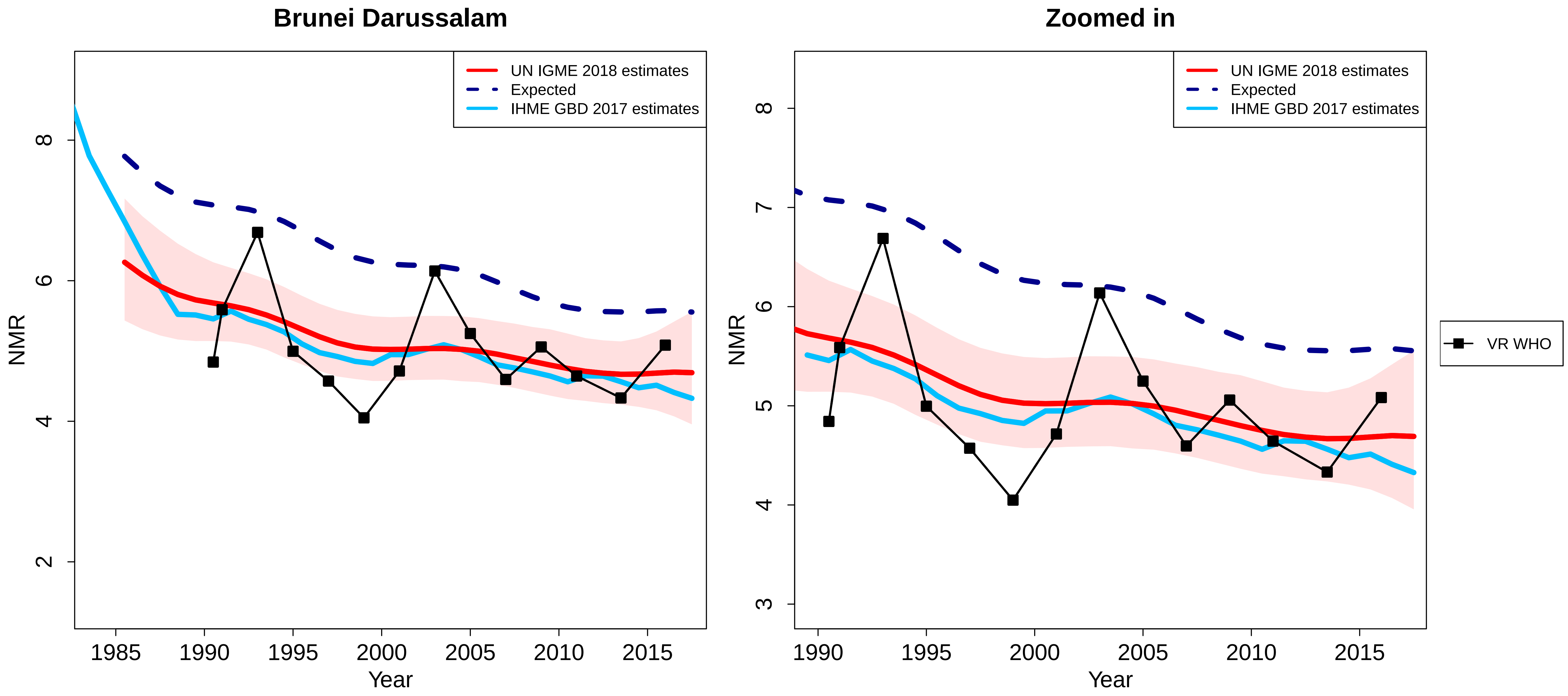
Brunei Darussalam
Zoomed in
UN IGME 2018 estimates
Expected
IHME GBD 2017 estimates
VR WHO
NMR
Year
1985
1990
1995
2000
2005
2010
2015
2
4
6
8
3
5
7

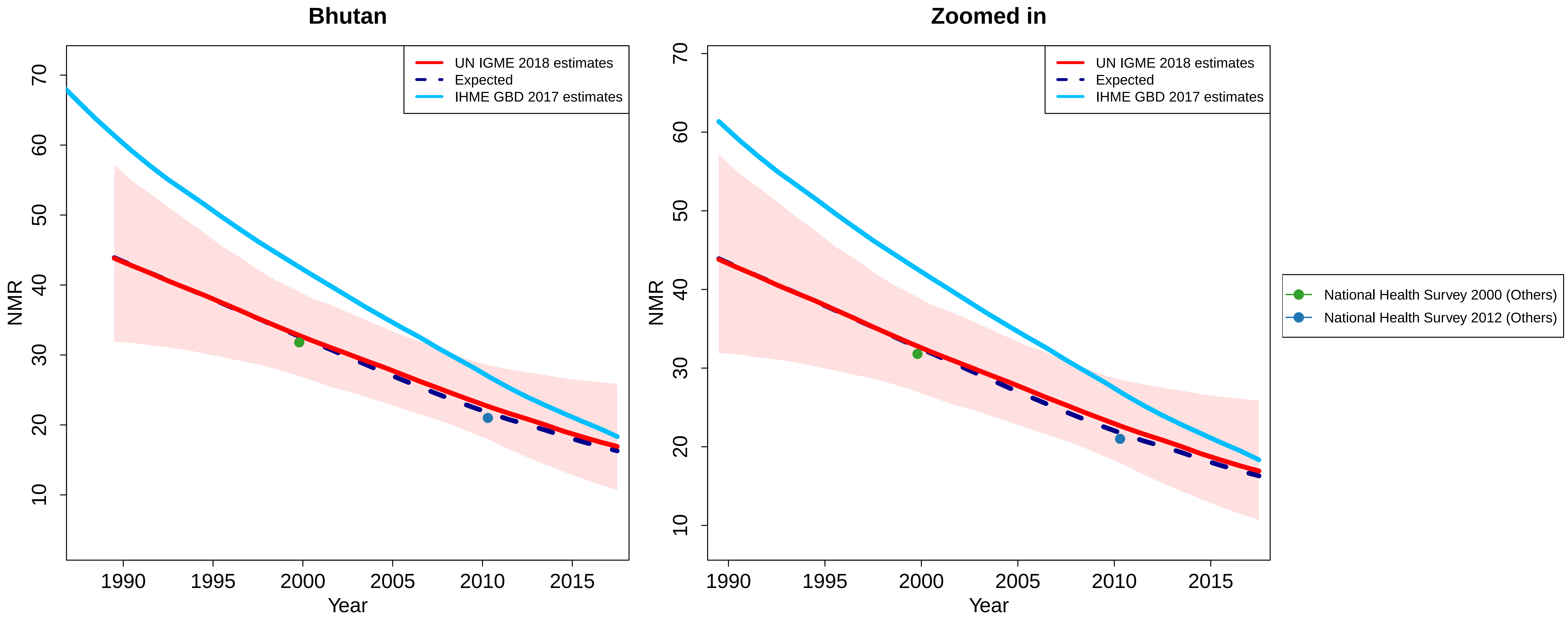
Bhutan
UN IGME 2018 estimates
Expected
IHME GBD 2017 estimates
NMR
Year
10
20
30
40
50
60
70
1990
1995
2000
2005
2010
2015
Zoomed in
UN IGME 2018 estimates
Expected
IHME GBD 2017 estimates
NMR
Year
National Health Survey 2000 (Others)
National Health Survey 2012 (Others)

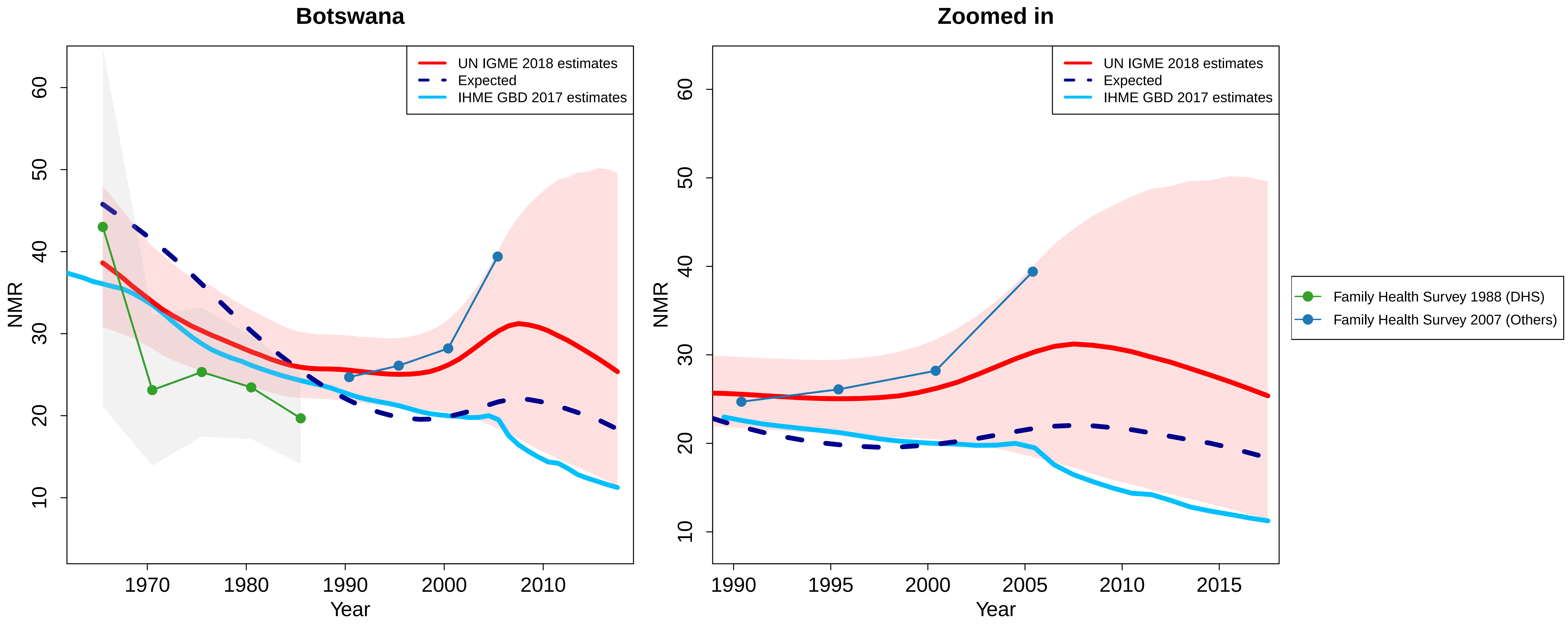
Botswana
Zoomed in
NMR
Year
UN IGME 2018 estimates
Expected
IHME GBD 2017 estimates
Family Health Survey 1988 (DHS)
Family Health Survey 2007 (Others)
10
20
30
40
50
60
1970
1980
1990
2000
2010
1995
2005
2015

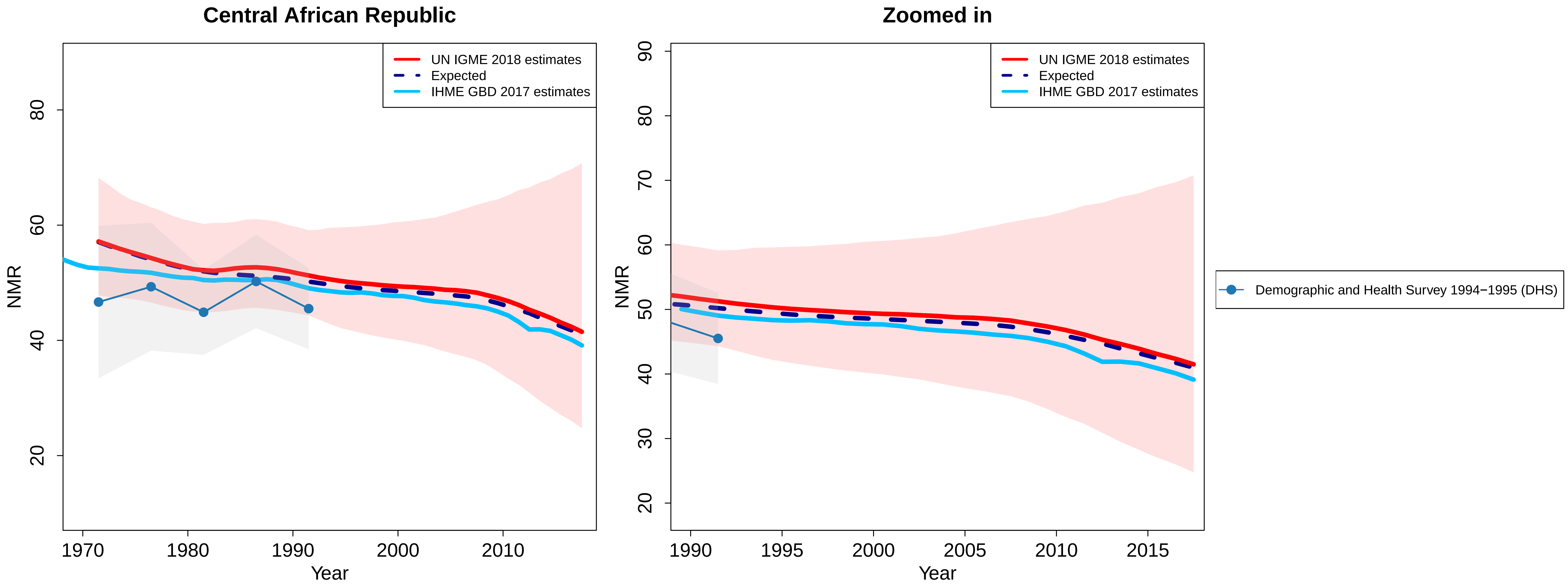
Central African Republic
Zoomed in
UN IGME 2018 estimates
Expected
IHME GBD 2017 estimates
NMR
Year
Demographic and Health Survey 1994−1995 (DHS)

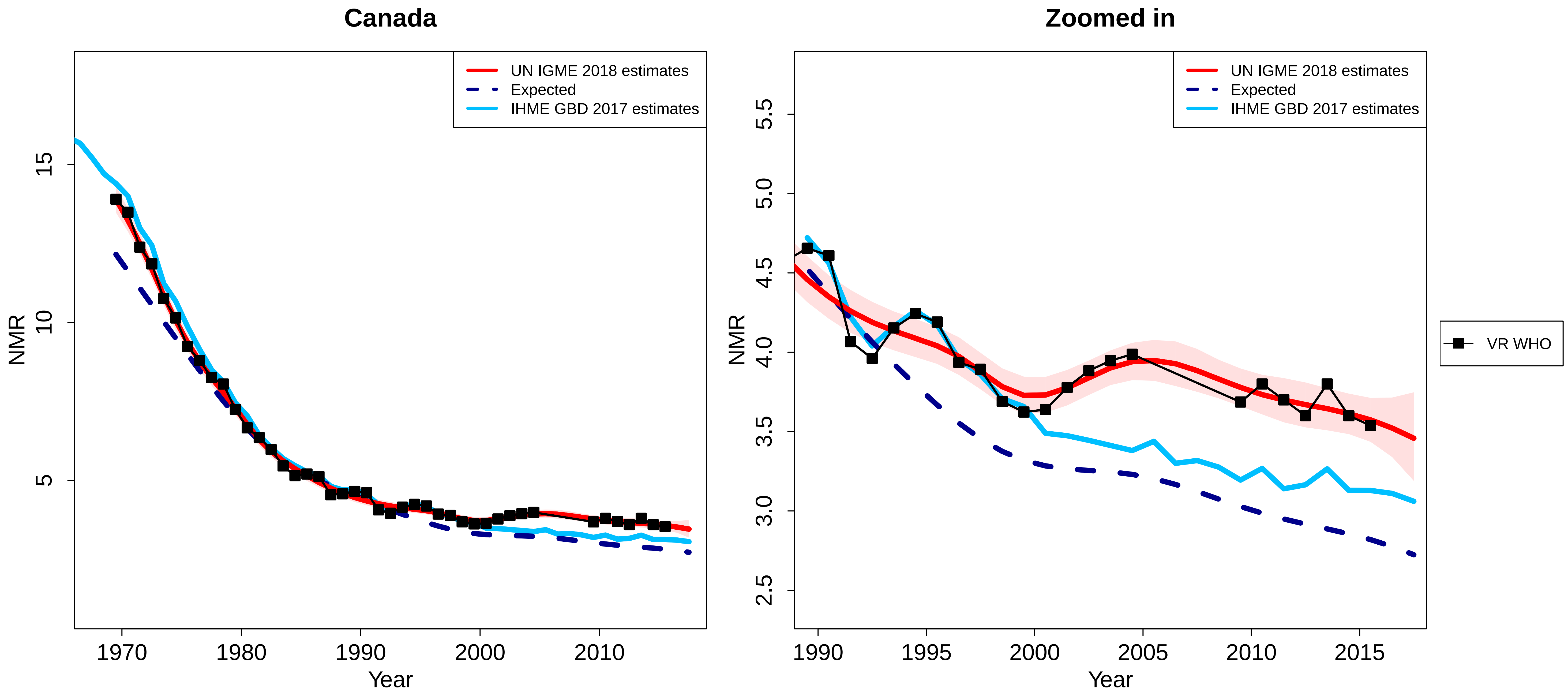

Canada
Zoomed in
UN IGME 2018 estimates
Expected
IHME GBD 2017 estimates
VR WHO
NMR
Year
5
10
15
1970
1980
1990
2000
2010
2.5
3.0
3.5
4.0
4.5
5.0
5.5
1990
1995
2000
2005
2010
2015

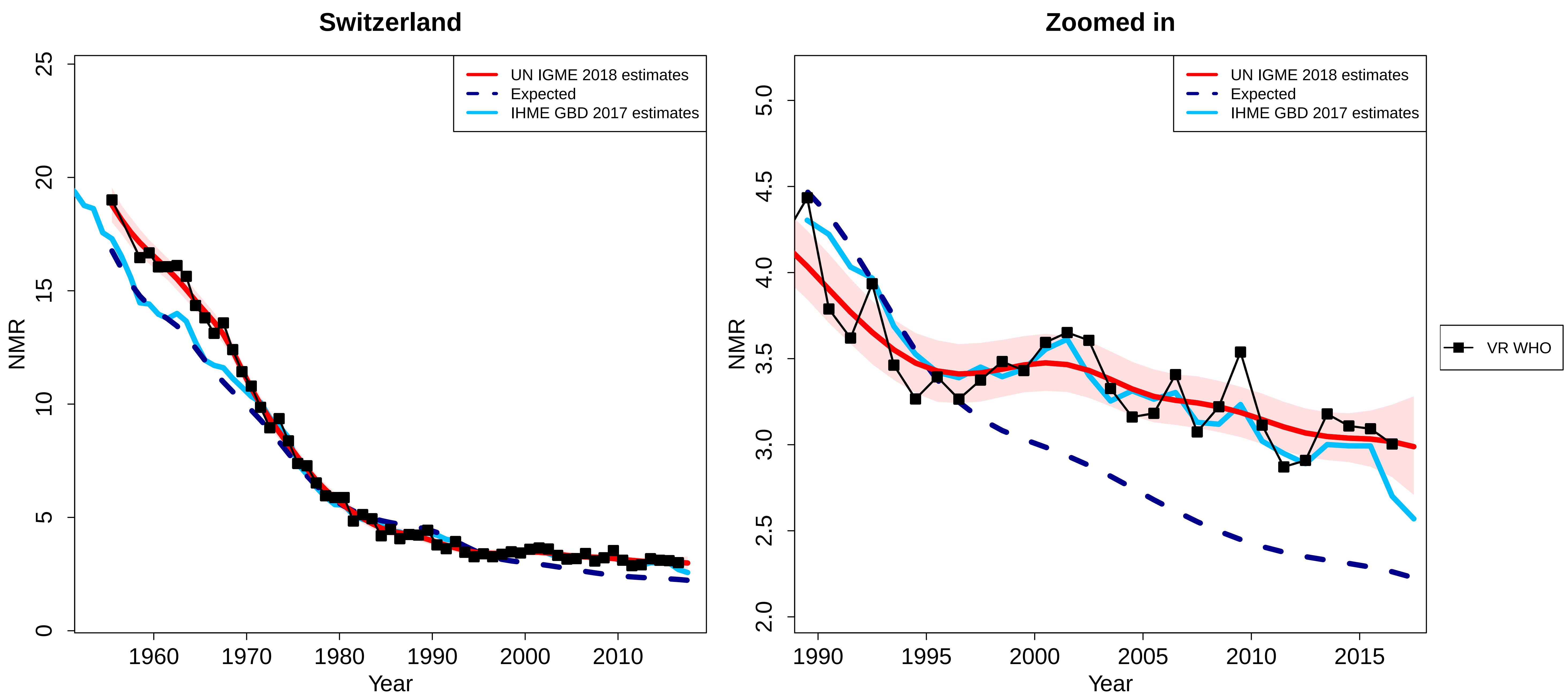

Switzerland
Zoomed in
UN IGME 2018 estimates
Expected
IHME GBD 2017 estimates
VR WHO
NMR
Year

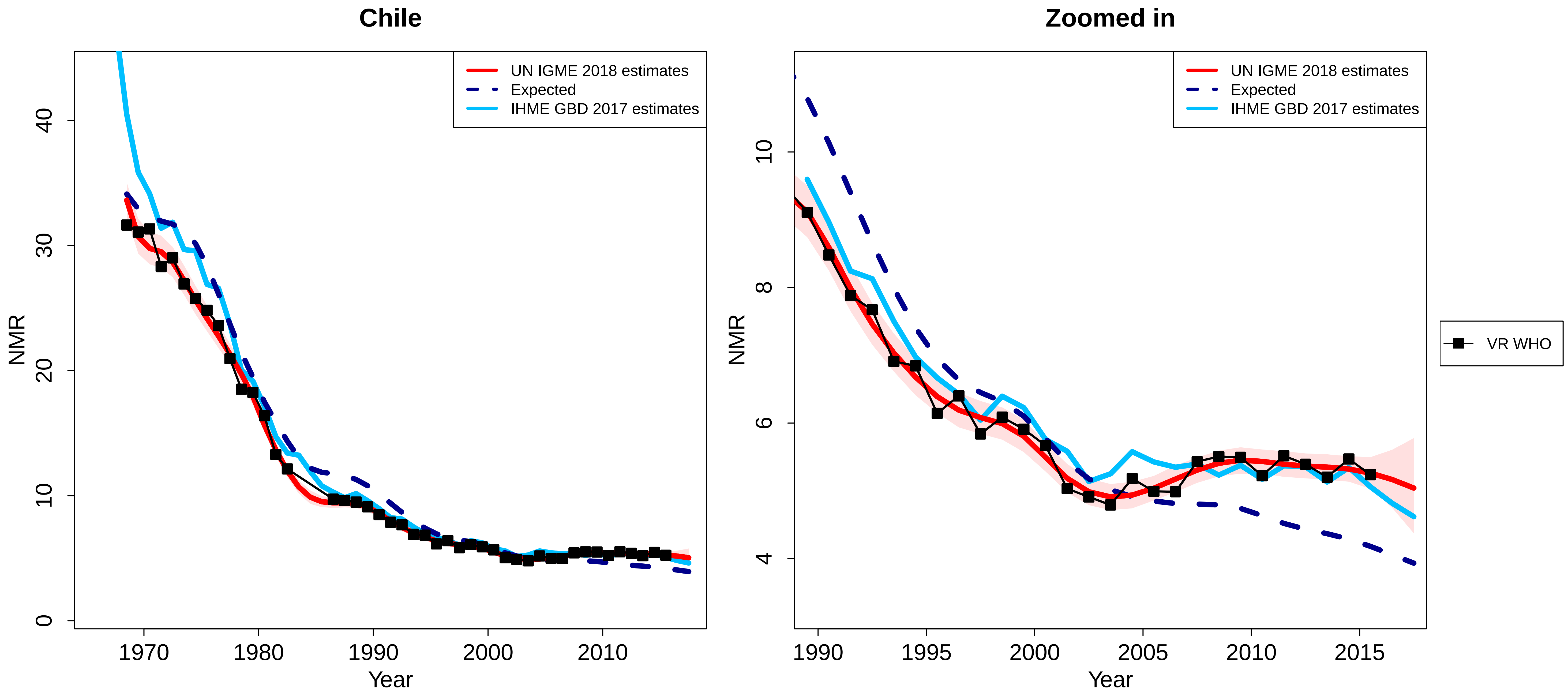

Chile
Zoomed in
UN IGME 2018 estimates
Expected
IHME GBD 2017 estimates
VR WHO
NMR
Year

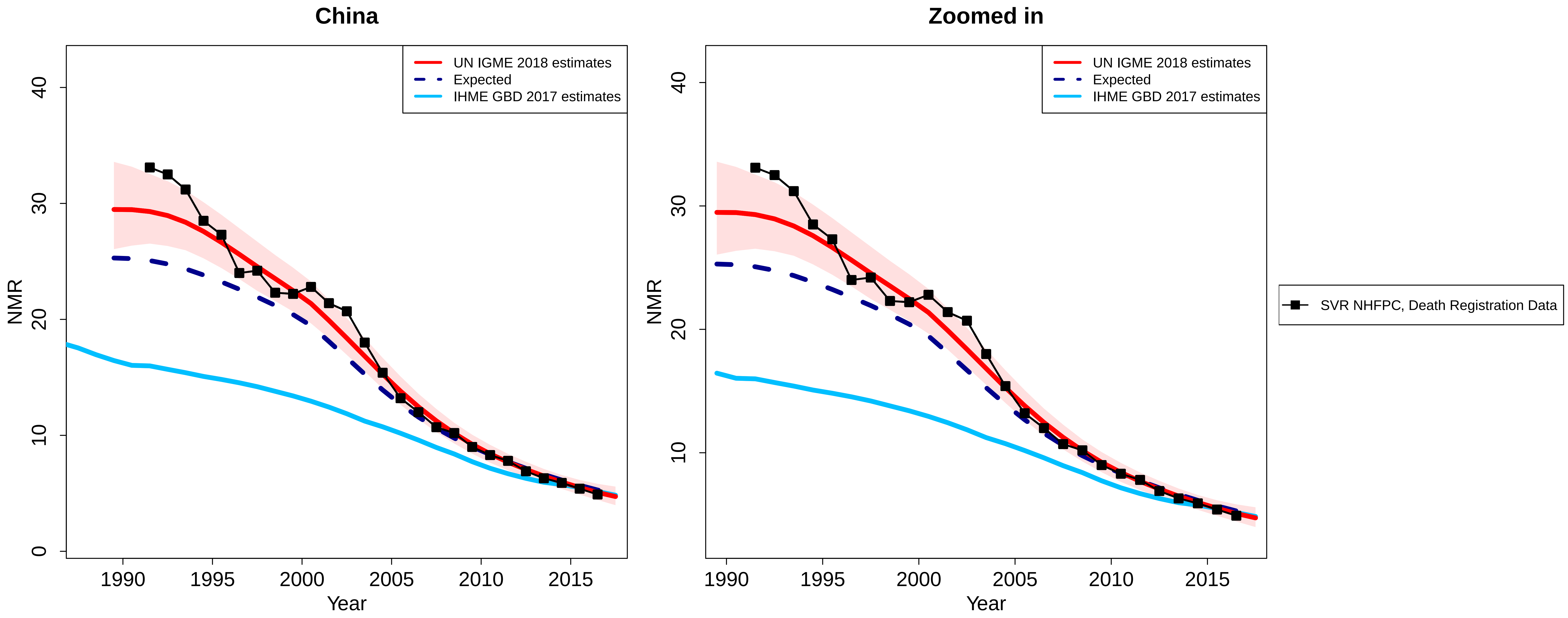
China
Zoomed in
UN IGME 2018 estimates
Expected
IHME GBD 2017 estimates
SVR NHFPC, Death Registration Data
NMR
Year
0
10
20
30
40
1990
1995
2000
2005
2010
2015

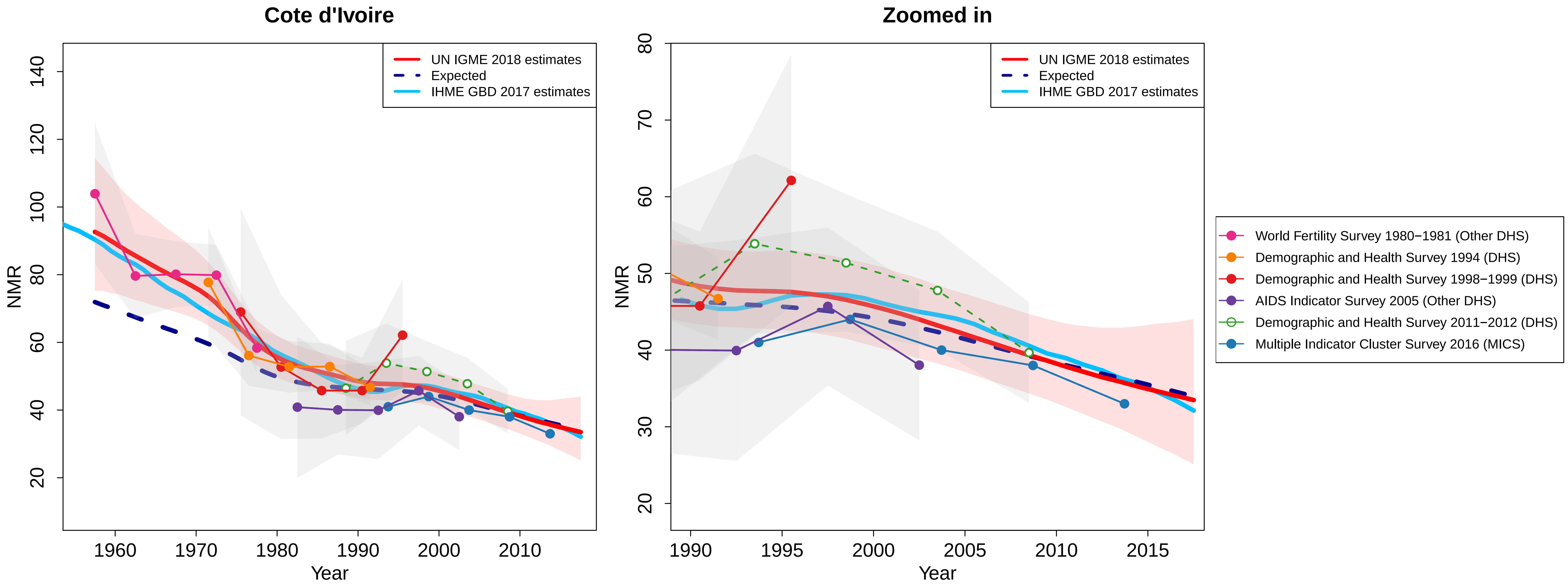

Cote d'Ivoire
Zoomed in
UN IGME 2018 estimates
Expected
IHME GBD 2017 estimates
World Fertility Survey 1980−1981 (Other DHS)
Demographic and Health Survey 1994 (DHS)
Demographic and Health Survey 1998−1999 (DHS)
AIDS Indicator Survey 2005 (Other DHS)
Demographic and Health Survey 2011−2012 (DHS)
Multiple Indicator Cluster Survey 2016 (MICS)
NMR
Year

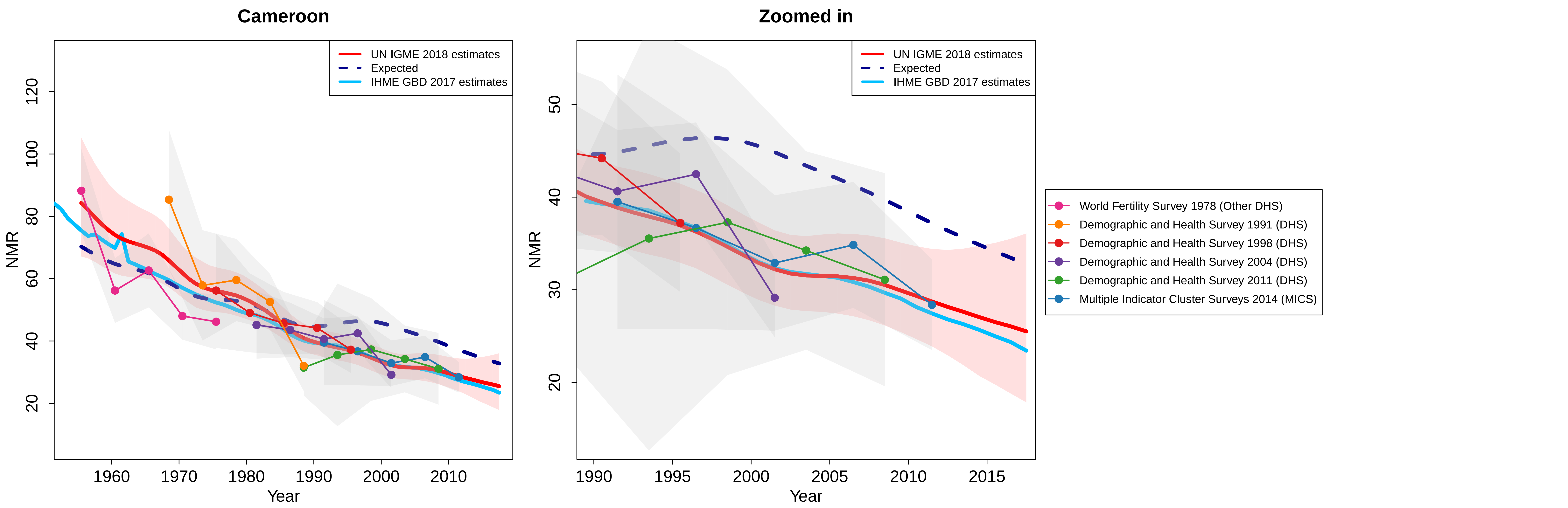
Cameroon
Zoomed in
UN IGME 2018 estimates
Expected
IHME GBD 2017 estimates
NMR
Year
World Fertility Survey 1978 (Other DHS)
Demographic and Health Survey 1991 (DHS)
Demographic and Health Survey 1998 (DHS)
Demographic and Health Survey 2004 (DHS)
Demographic and Health Survey 2011 (DHS)
Multiple Indicator Cluster Surveys 2014 (MICS)

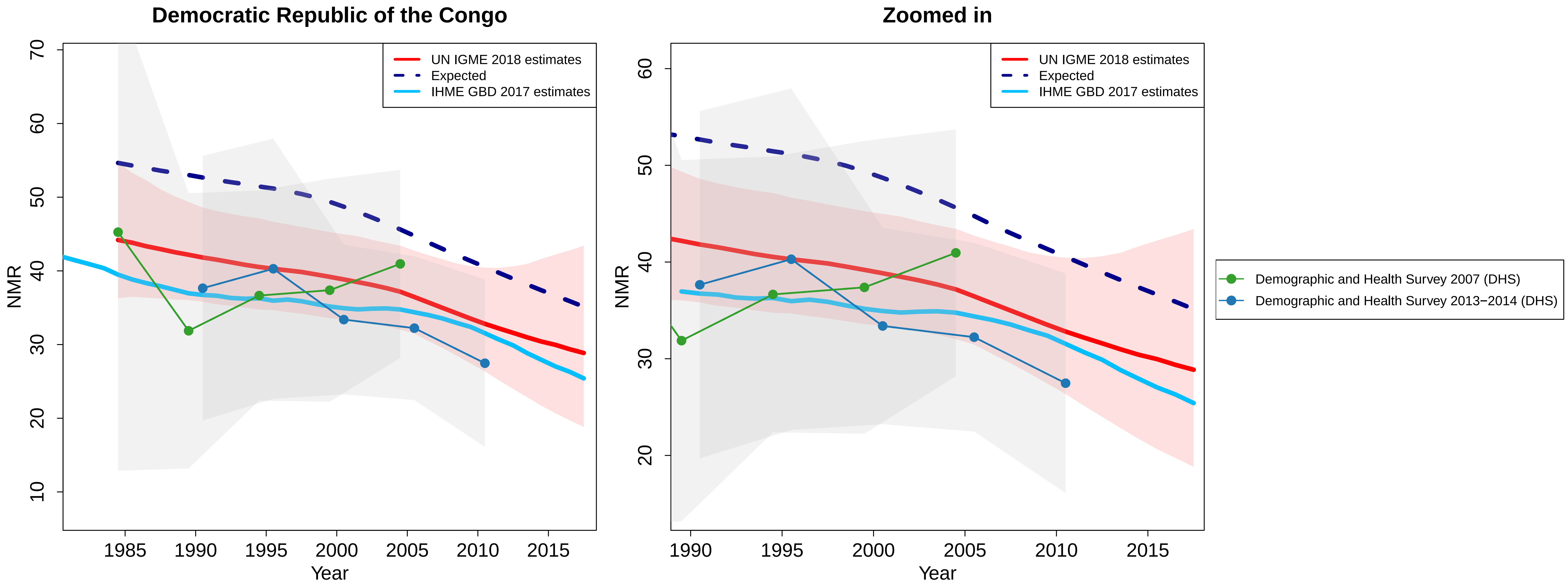

Democratic Republic of the Congo
Zoomed in
UN IGME 2018 estimates
Expected
IHME GBD 2017 estimates
Demographic and Health Survey 2007 (DHS)
Demographic and Health Survey 2013−2014 (DHS)
NMR
Year

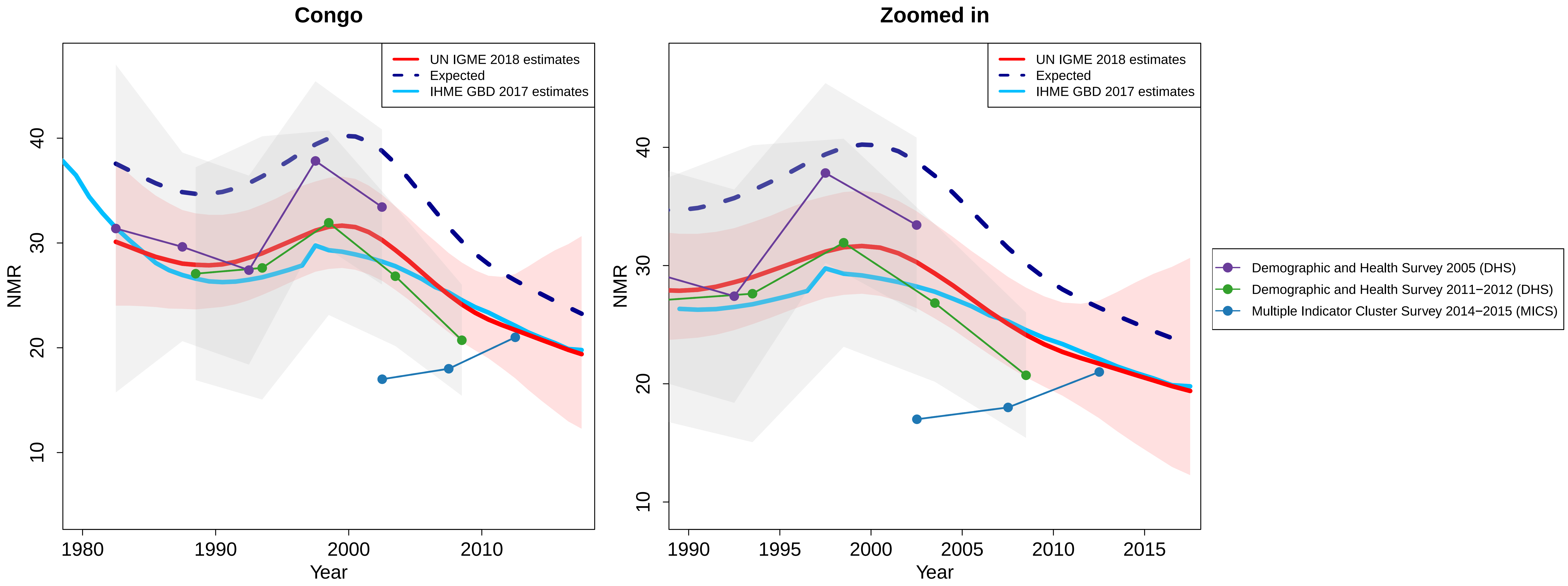

Congo
Zoomed in
UN IGME 2018 estimates
Expected
IHME GBD 2017 estimates
NMR
Year
10
20
30
40
1980
1990
2000
2010
1995
2005
2015
Demographic and Health Survey 2005 (DHS)
Demographic and Health Survey 2011−2012 (DHS)
Multiple Indicator Cluster Survey 2014−2015 (MICS)

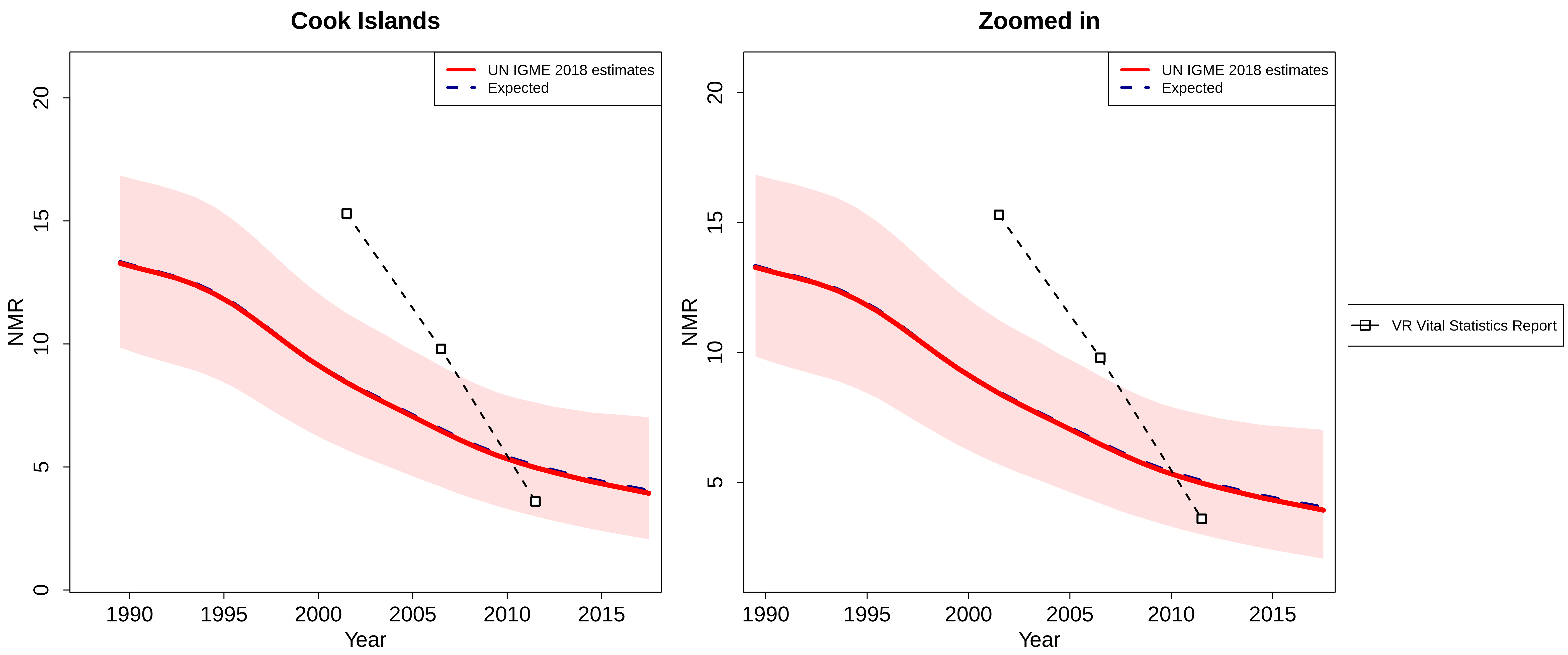

Cook Islands
Zoomed in
UN IGME 2018 estimates
Expected
VR Vital Statistics Report
NMR
Year
0
5
10
15
20
1990
1995
2000
2005
2010
2015

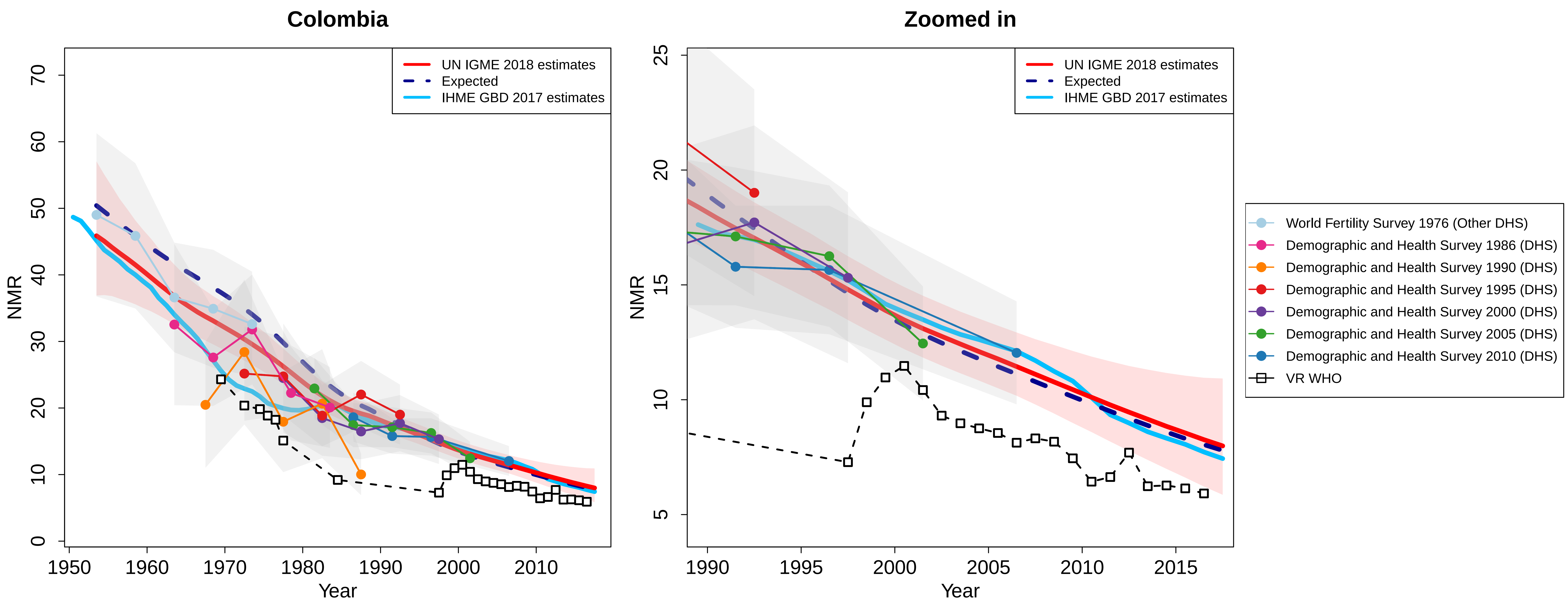

Colombia
Zoomed in
UN IGME 2018 estimates
Expected
IHME GBD 2017 estimates
NMR
Year
World Fertility Survey 1976 (Other DHS)
Demographic and Health Survey 1986 (DHS)
Demographic and Health Survey 1990 (DHS)
Demographic and Health Survey 1995 (DHS)
Demographic and Health Survey 2000 (DHS)
Demographic and Health Survey 2005 (DHS)
Demographic and Health Survey 2010 (DHS)
VR WHO

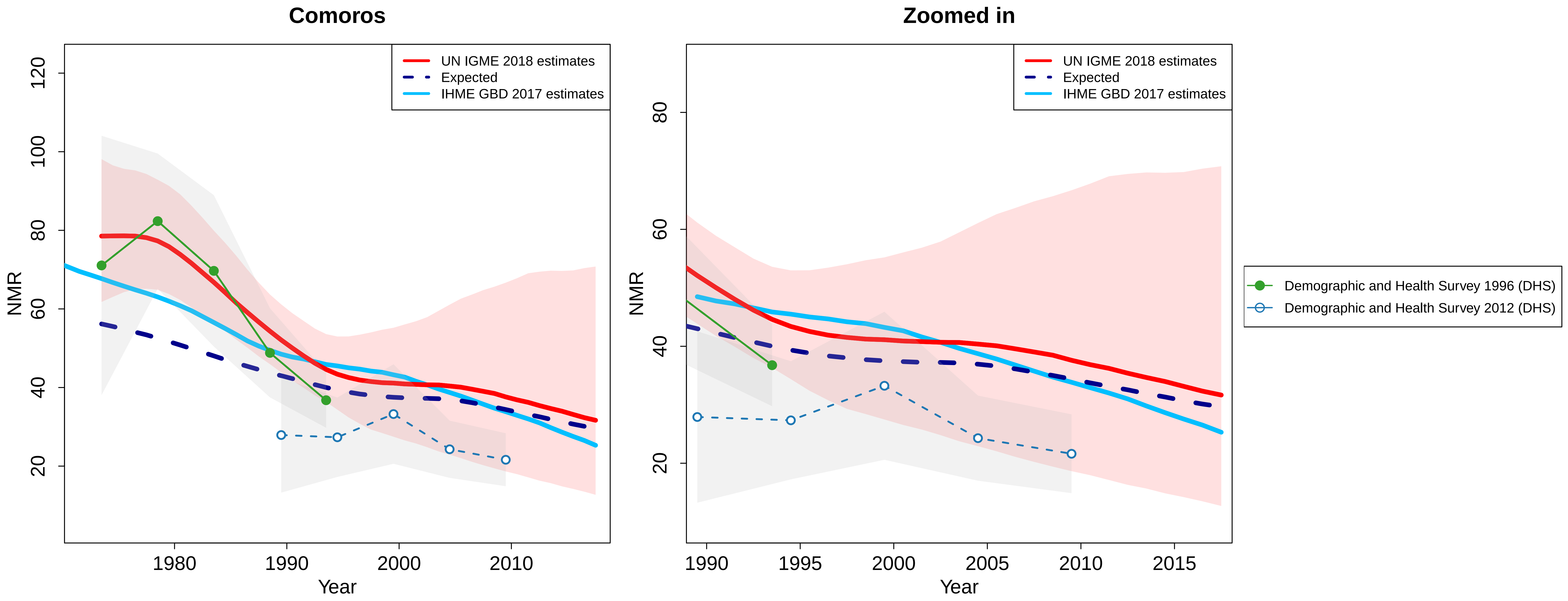

Comoros
Zoomed in
UN IGME 2018 estimates
Expected
IHME GBD 2017 estimates
NMR
Year
120
100
80
60
40
20
1980
1990
2000
2010
1995
2005
2015
Demographic and Health Survey 1996 (DHS)
Demographic and Health Survey 2012 (DHS)

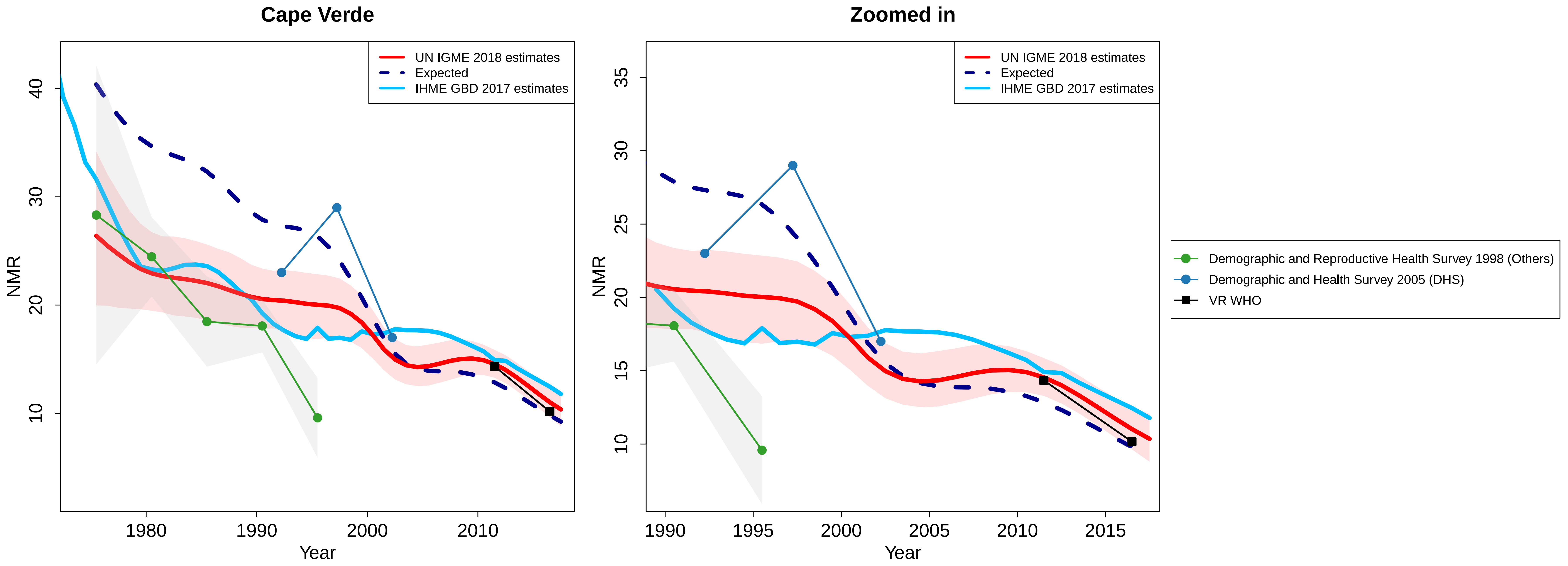

Cape Verde
Zoomed in
UN IGME 2018 estimates
Expected
IHME GBD 2017 estimates
NMR
Year
Demographic and Reproductive Health Survey 1998 (Others)
Demographic and Health Survey 2005 (DHS)
VR WHO

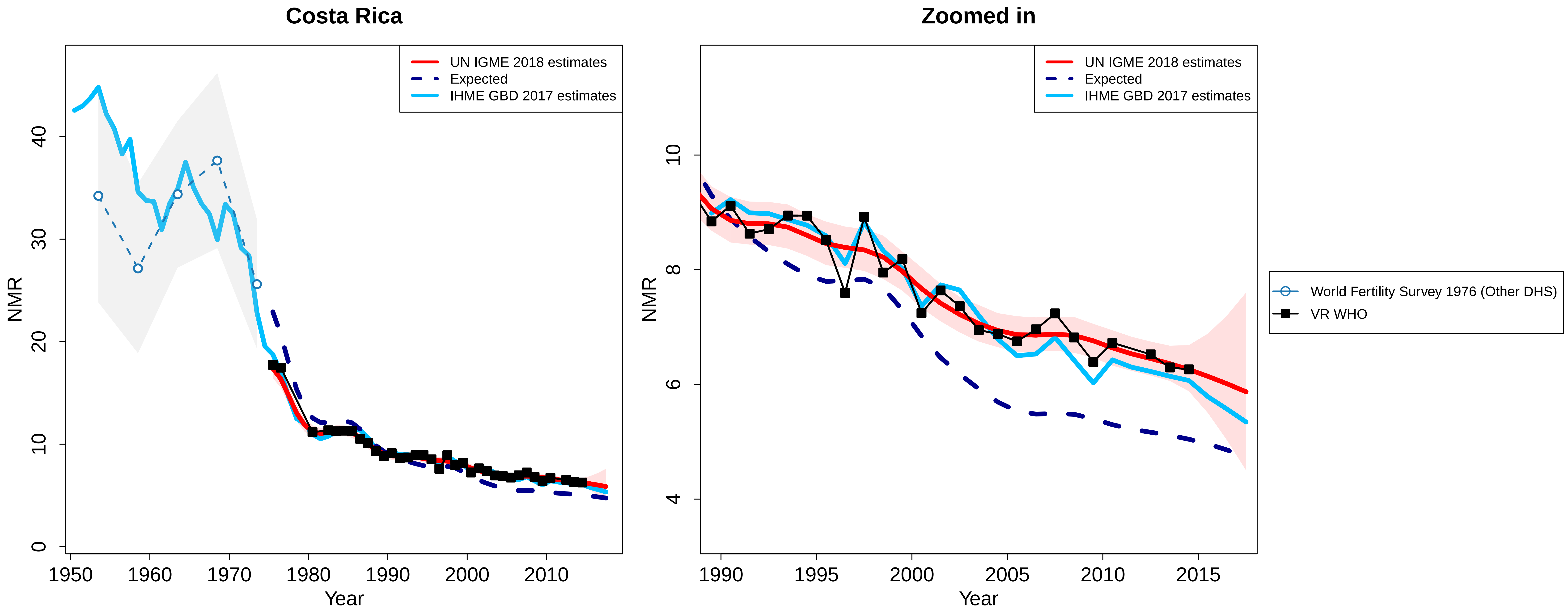
Costa Rica
UN IGME 2018 estimates
Expected
IHME GBD 2017 estimates
NMR
Year
Zoomed in
UN IGME 2018 estimates
Expected
IHME GBD 2017 estimates
NMR
Year
World Fertility Survey 1976 (Other DHS)
VR WHO

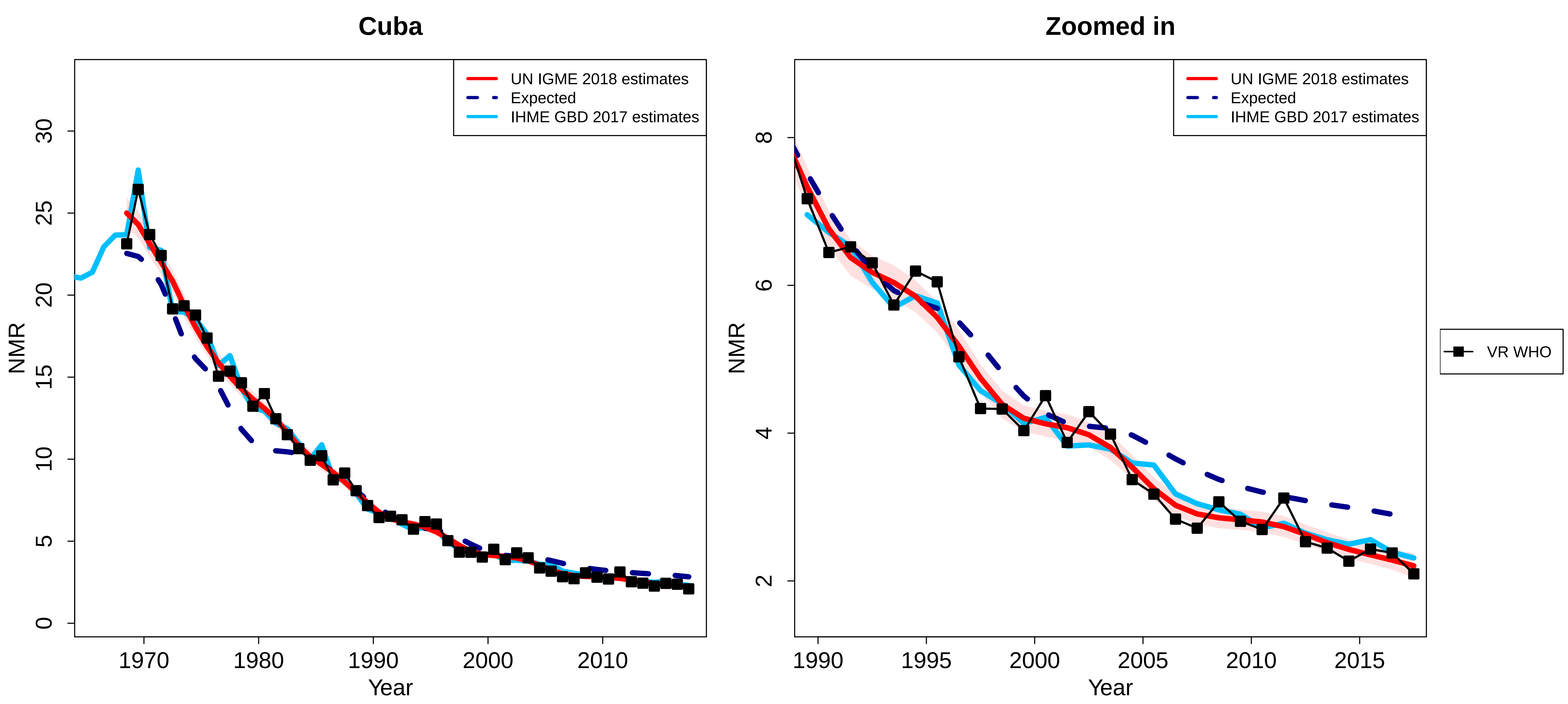

Cuba
UN IGME 2018 estimates
Expected
IHME GBD 2017 estimates
NMR
Year
0
5
10
15
20
25
30
1970
1980
1990
2000
2010
Zoomed in
UN IGME 2018 estimates
Expected
IHME GBD 2017 estimates
NMR
Year
2
4
6
8
1990
1995
2000
2005
2010
2015
VR WHO

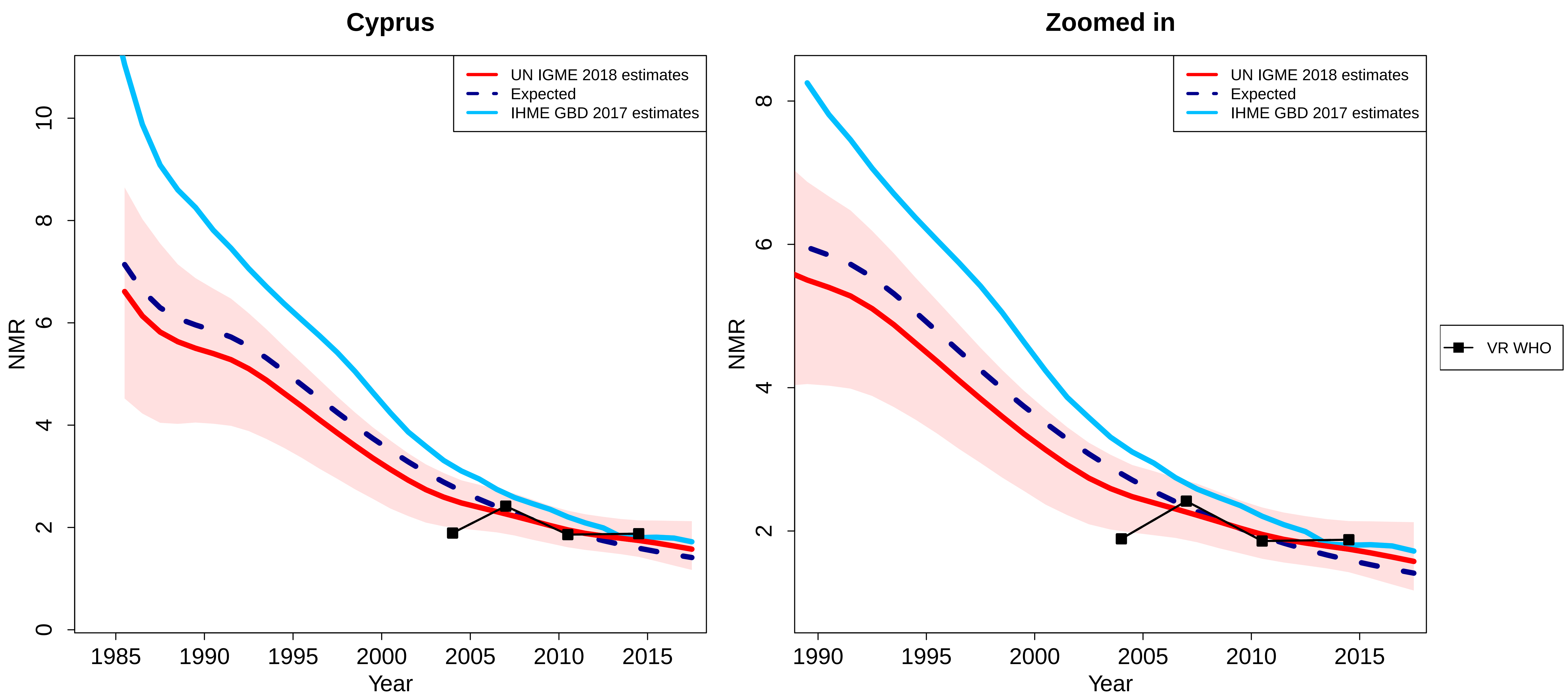
Cyprus
Zoomed in
UN IGME 2018 estimates
Expected
IHME GBD 2017 estimates
VR WHO
NMR
Year
0
2
4
6
8
10
1985
1990
1995
2000
2005
2010
2015

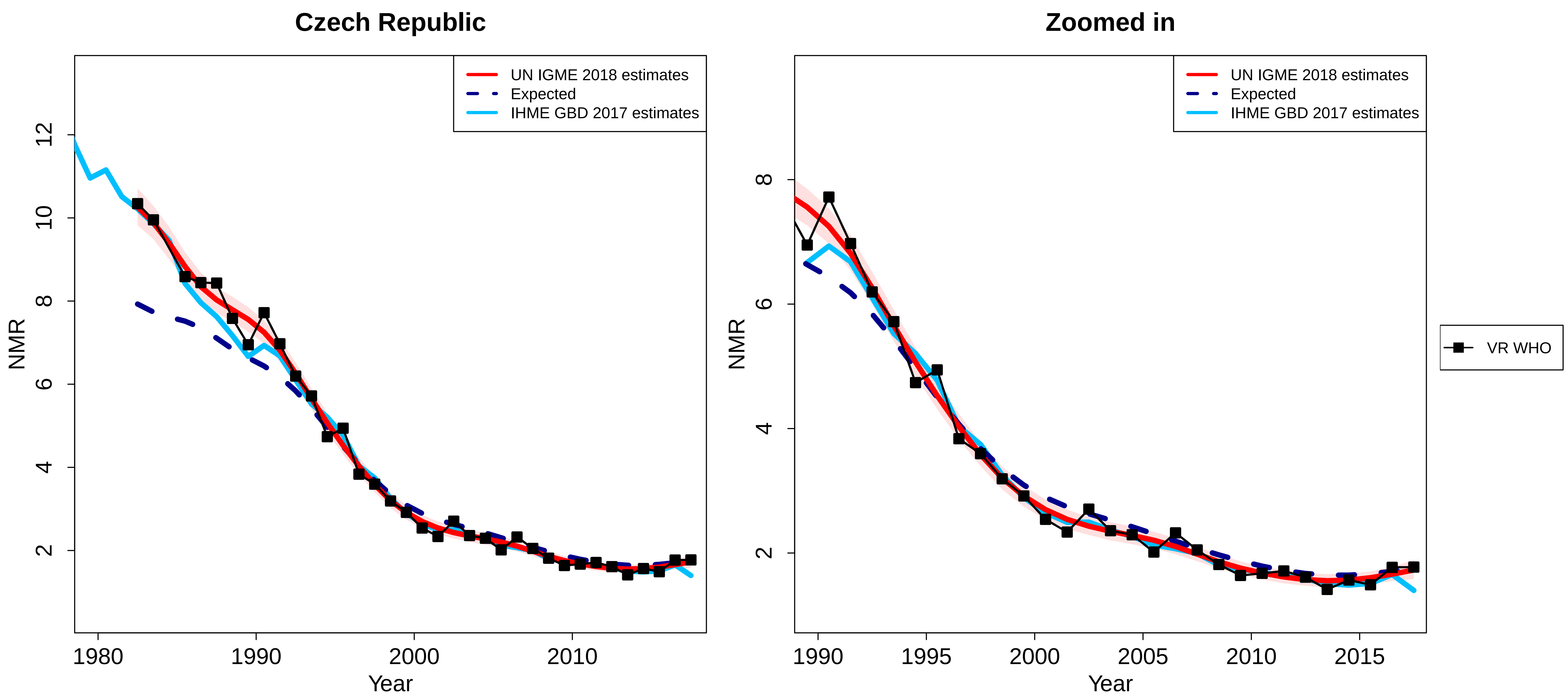
Czech Republic
Zoomed in
UN IGME 2018 estimates
Expected
IHME GBD 2017 estimates
VR WHO
NMR
Year
1980
1990
2000
2010
2
4
6
8
10
12
1995
2005
2015

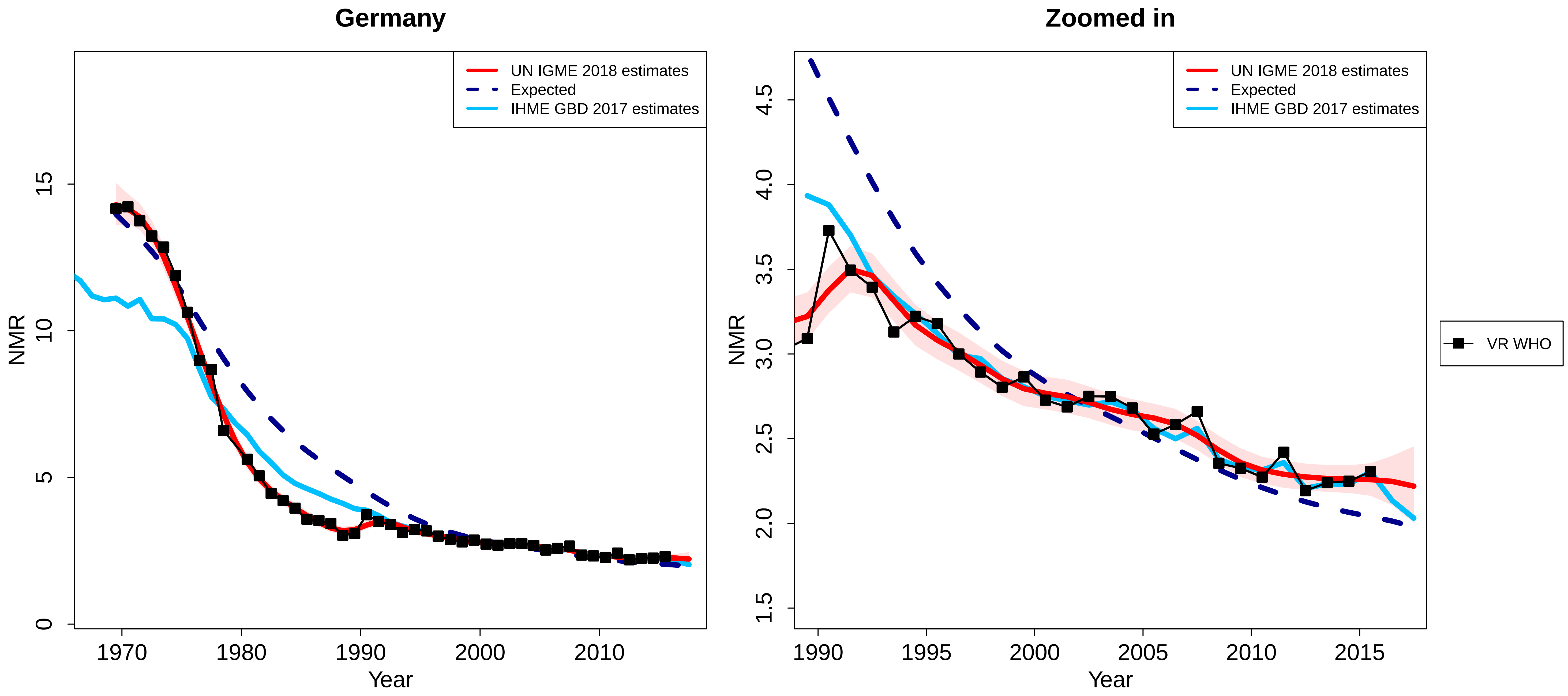
Germany
Zoomed in
UN IGME 2018 estimates
Expected
IHME GBD 2017 estimates
VR WHO
NMR
Year
0
5
10
15
1970
1980
1990
2000
2010
1.5
2.0
2.5
3.0
3.5
4.0
4.5
1995
2005
2015

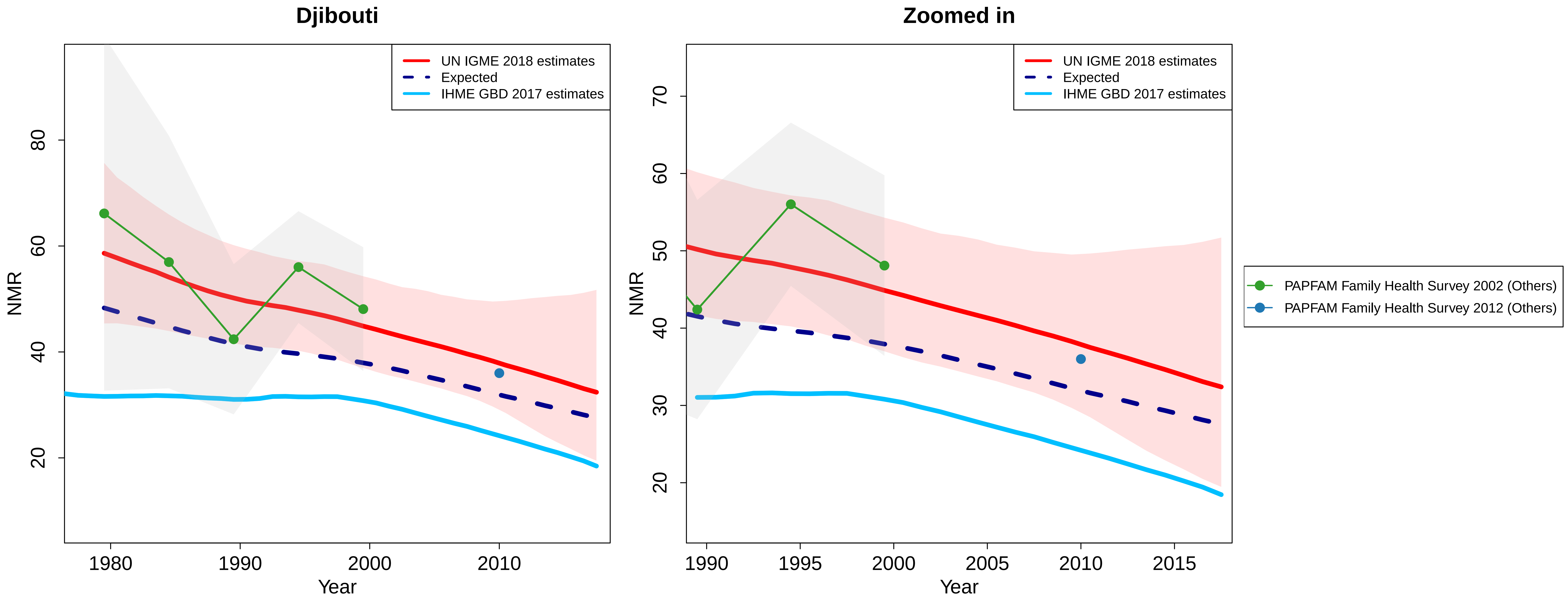
Djibouti
Zoomed in
UN IGME 2018 estimates
Expected
IHME GBD 2017 estimates
NMR
Year
PAPFAM Family Health Survey 2002 (Others)
PAPFAM Family Health Survey 2012 (Others)

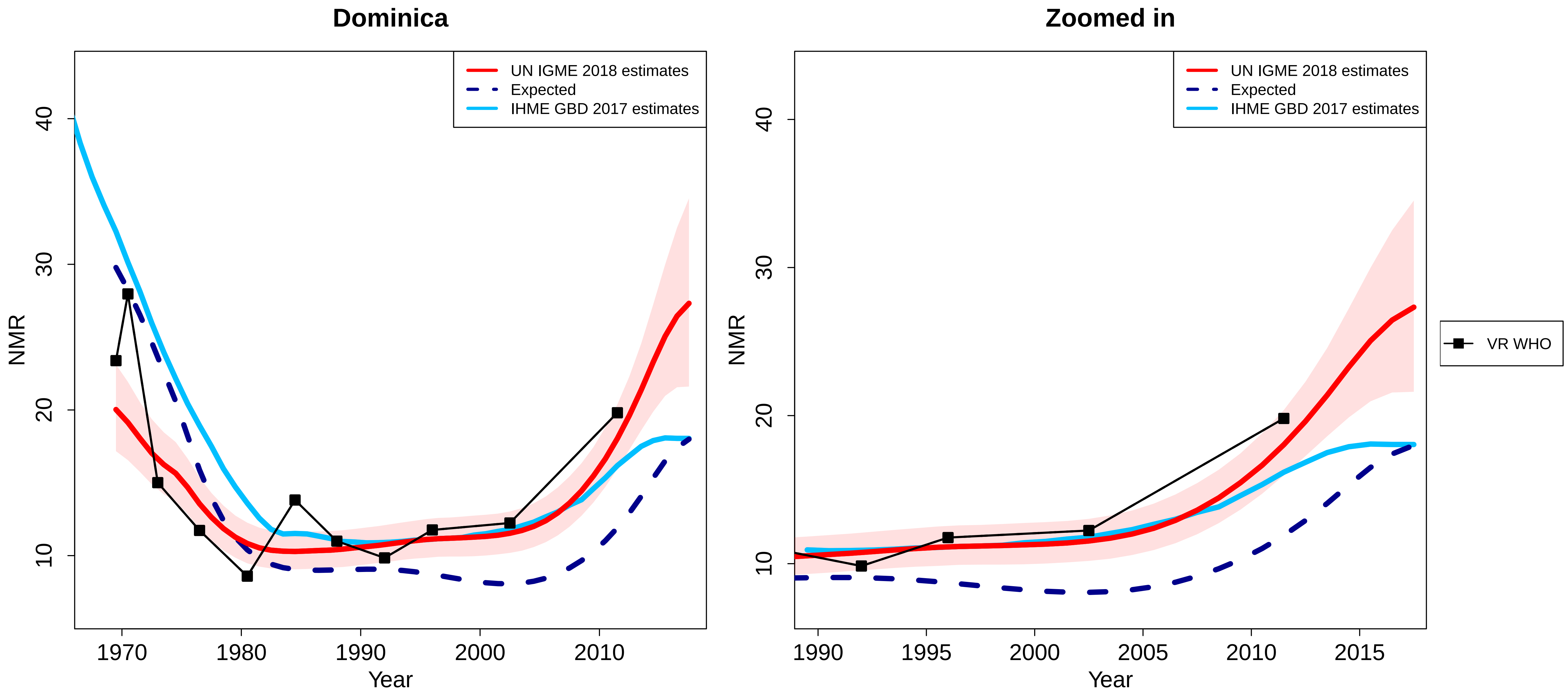
Dominica
Zoomed in
UN IGME 2018 estimates
Expected
IHME GBD 2017 estimates
VR WHO
NMR
Year
10
20
30
40
1970
1980
1990
2000
2010
1995
2005
2015

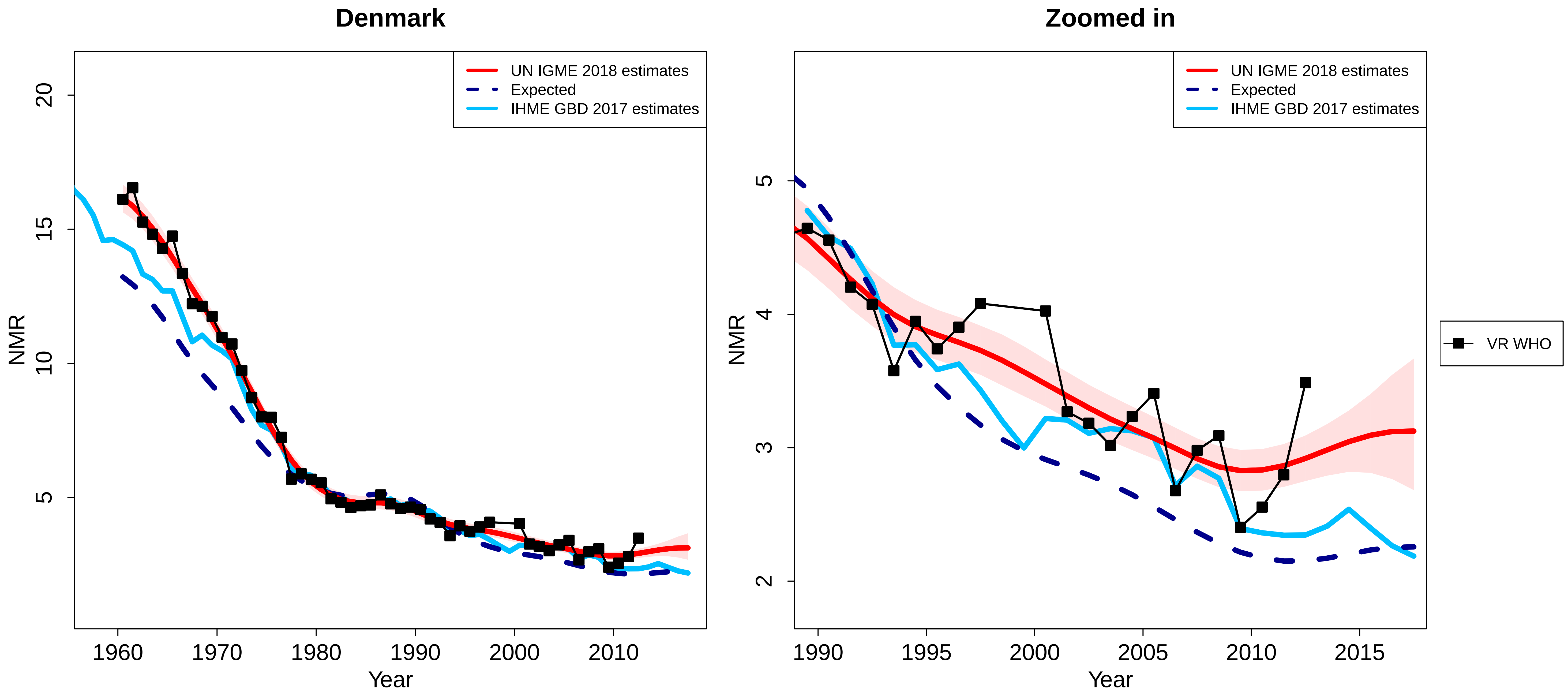
Denmark
NMR
Year
UN IGME 2018 estimates
Expected
IHME GBD 2017 estimates
Zoomed in
VR WHO

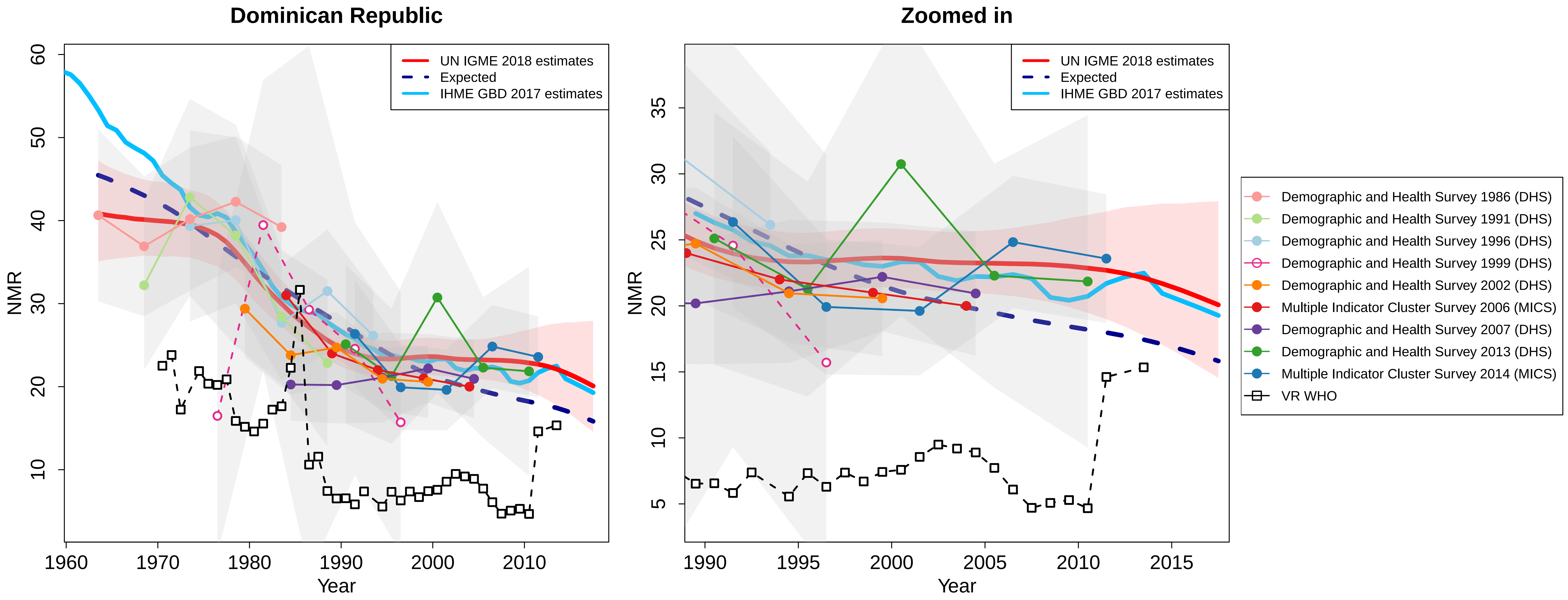
Dominican Republic
Zoomed in
UN IGME 2018 estimates
Expected
IHME GBD 2017 estimates
NMR
Year
Demographic and Health Survey 1986 (DHS)
Demographic and Health Survey 1991 (DHS)
Demographic and Health Survey 1996 (DHS)
Demographic and Health Survey 1999 (DHS)
Demographic and Health Survey 2002 (DHS)
Multiple Indicator Cluster Survey 2006 (MICS)
Demographic and Health Survey 2007 (DHS)
Demographic and Health Survey 2013 (DHS)
Multiple Indicator Cluster Survey 2014 (MICS)
VR WHO

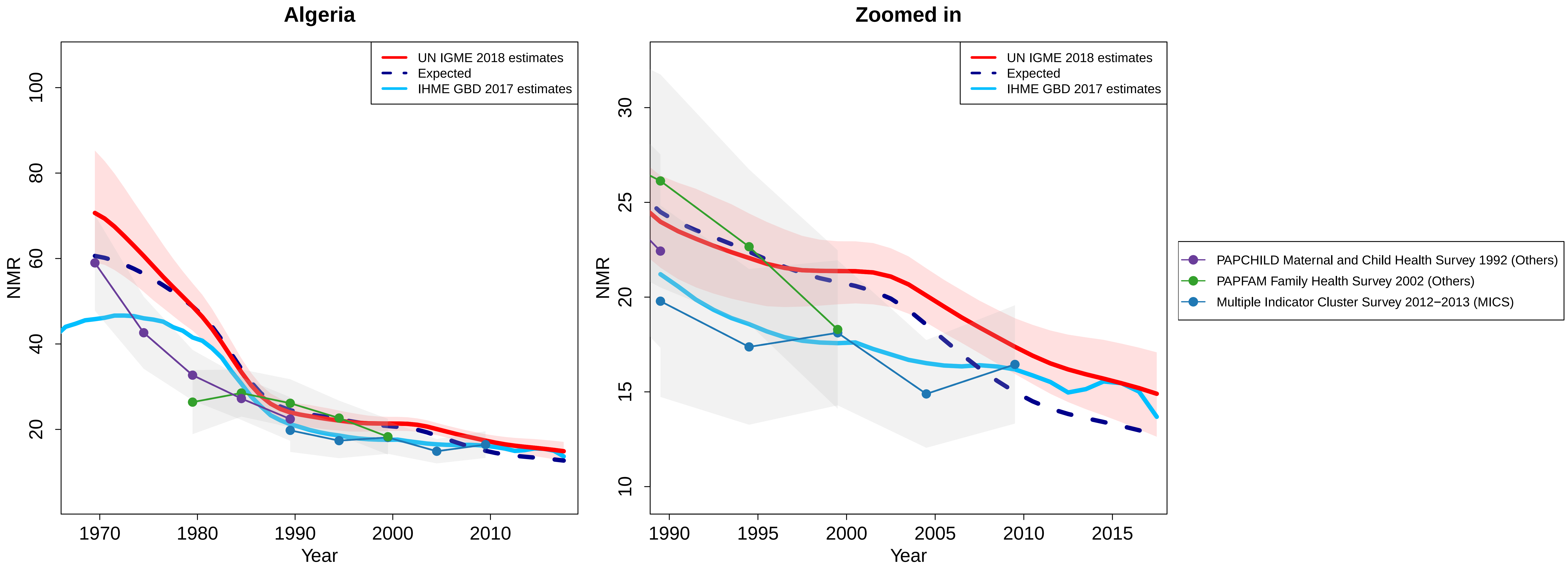
Algeria
Zoomed in
UN IGME 2018 estimates
Expected
IHME GBD 2017 estimates
NMR
Year
PAPCHILD Maternal and Child Health Survey 1992 (Others)
PAPFAM Family Health Survey 2002 (Others)
Multiple Indicator Cluster Survey 2012−2013 (MICS)

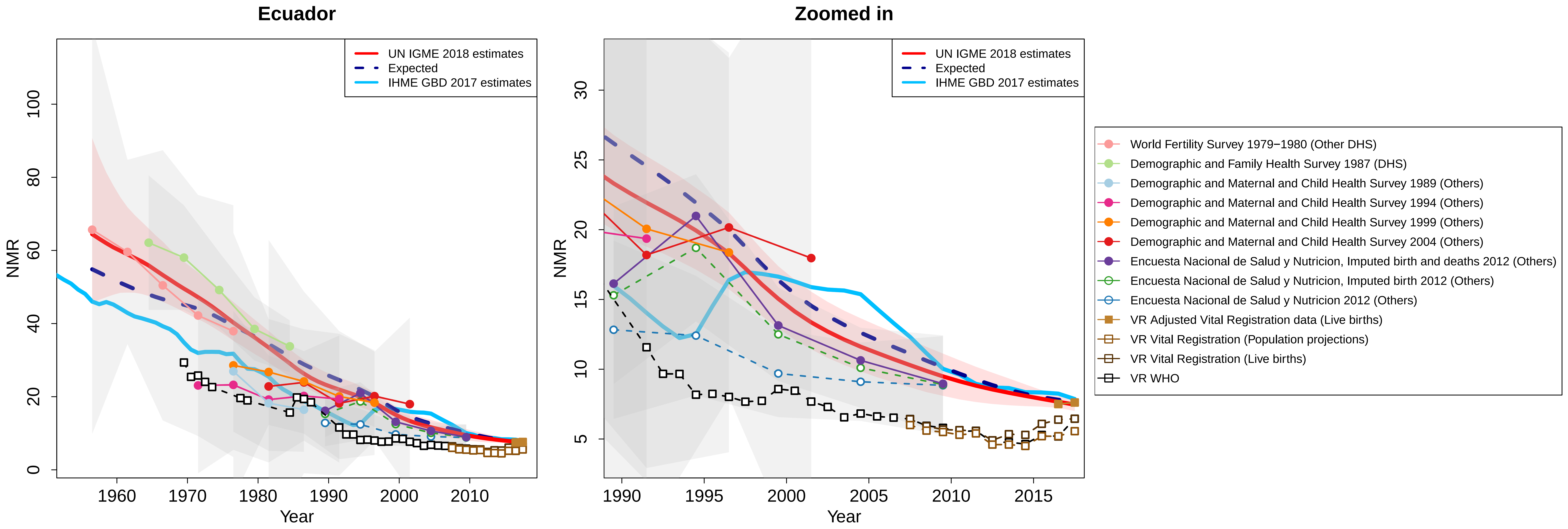

Ecuador
Zoomed in
UN IGME 2018 estimates
Expected
IHME GBD 2017 estimates
NMR
Year
World Fertility Survey 1979−1980 (Other DHS)
Demographic and Family Health Survey 1987 (DHS)
Demographic and Maternal and Child Health Survey 1989 (Others)
Demographic and Maternal and Child Health Survey 1994 (Others)
Demographic and Maternal and Child Health Survey 1999 (Others)
Demographic and Maternal and Child Health Survey 2004 (Others)
Encuesta Nacional de Salud y Nutricion, Imputed birth and deaths 2012 (Others)
Encuesta Nacional de Salud y Nutricion, Imputed birth 2012 (Others)
Encuesta Nacional de Salud y Nutricion 2012 (Others)
VR Adjusted Vital Registration data (Live births)
VR Vital Registration (Population projections)
VR Vital Registration (Live births)
VR WHO

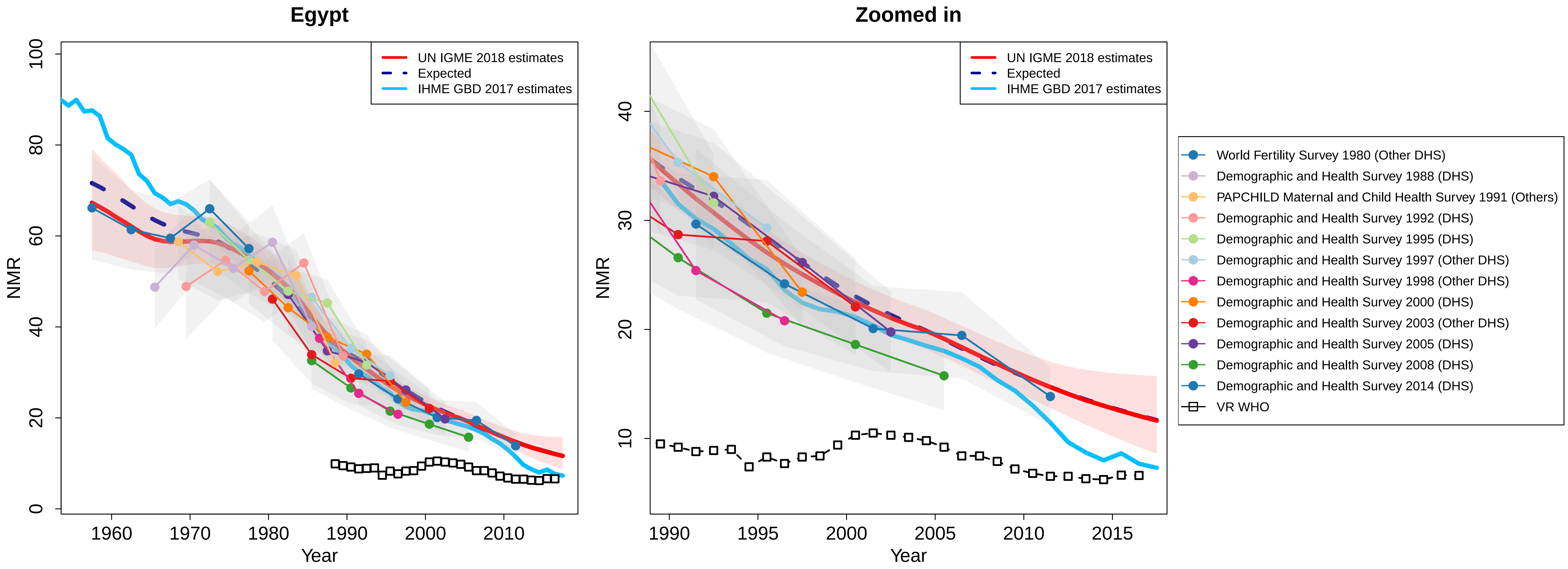

Egypt
Zoomed in
UN IGME 2018 estimates
Expected
IHME GBD 2017 estimates
NMR
Year
World Fertility Survey 1980 (Other DHS)
Demographic and Health Survey 1988 (DHS)
PAPCHILD Maternal and Child Health Survey 1991 (Others)
Demographic and Health Survey 1992 (DHS)
Demographic and Health Survey 1995 (DHS)
Demographic and Health Survey 1997 (Other DHS)
Demographic and Health Survey 1998 (Other DHS)
Demographic and Health Survey 2000 (DHS)
Demographic and Health Survey 2003 (Other DHS)
Demographic and Health Survey 2005 (DHS)
Demographic and Health Survey 2008 (DHS)
Demographic and Health Survey 2014 (DHS)
VR WHO

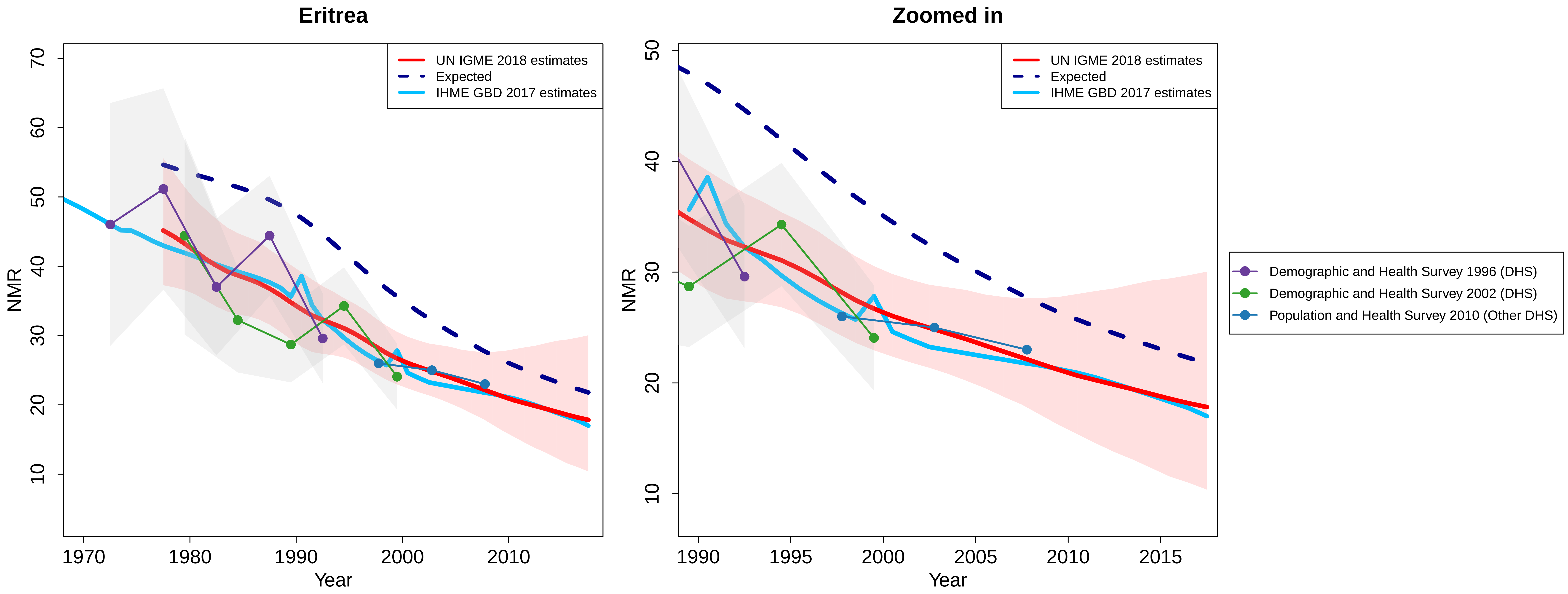

Eritrea
Zoomed in
UN IGME 2018 estimates
Expected
IHME GBD 2017 estimates
NMR
Year
1970
1980
1990
2000
2010
10
20
30
40
50
60
70
1995
2005
2015
Demographic and Health Survey 1996 (DHS)
Demographic and Health Survey 2002 (DHS)
Population and Health Survey 2010 (Other DHS)

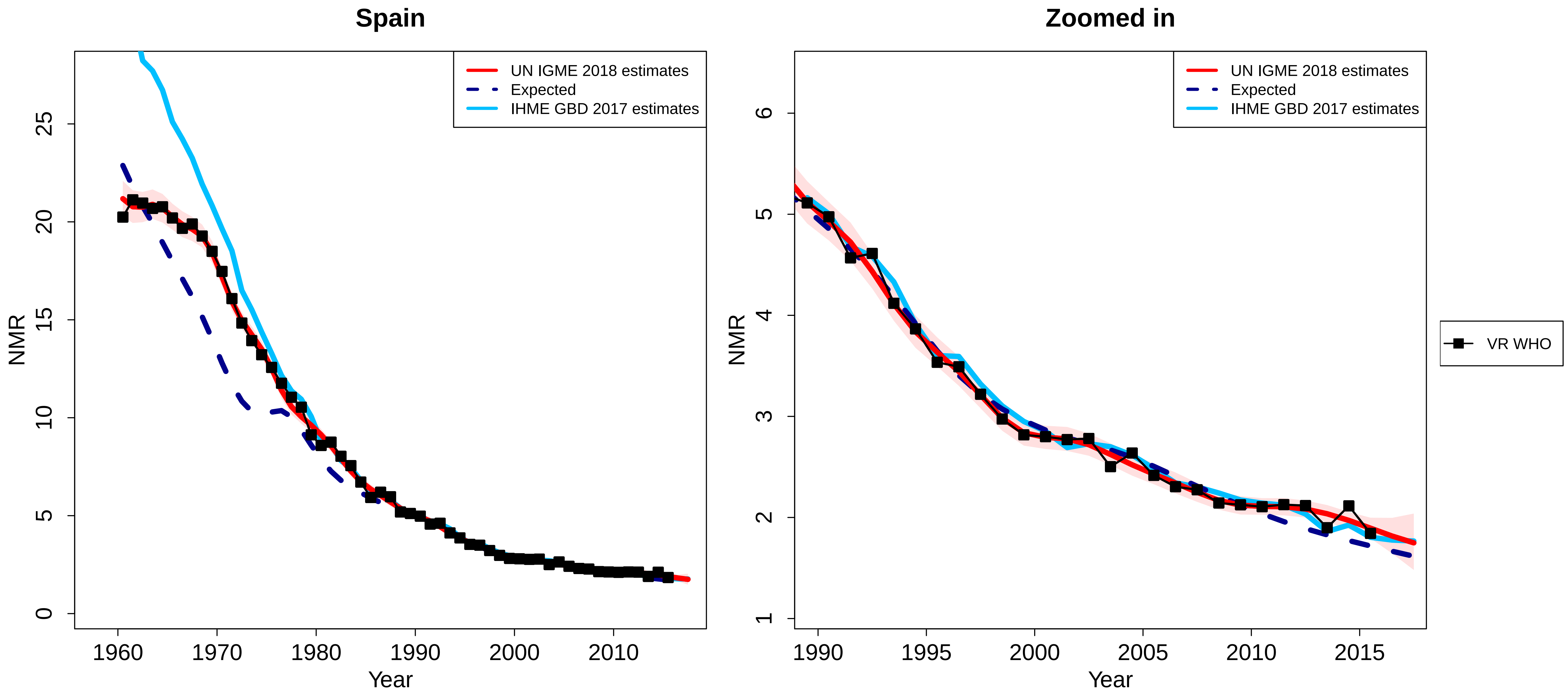
Spain
Zoomed in
UN IGME 2018 estimates
Expected
IHME GBD 2017 estimates
VR WHO
NMR
Year
0
5
10
15
20
25
1960
1970
1980
1990
2000
2010
1
2
3
4
5
6
1990
1995
2000
2005
2010
2015

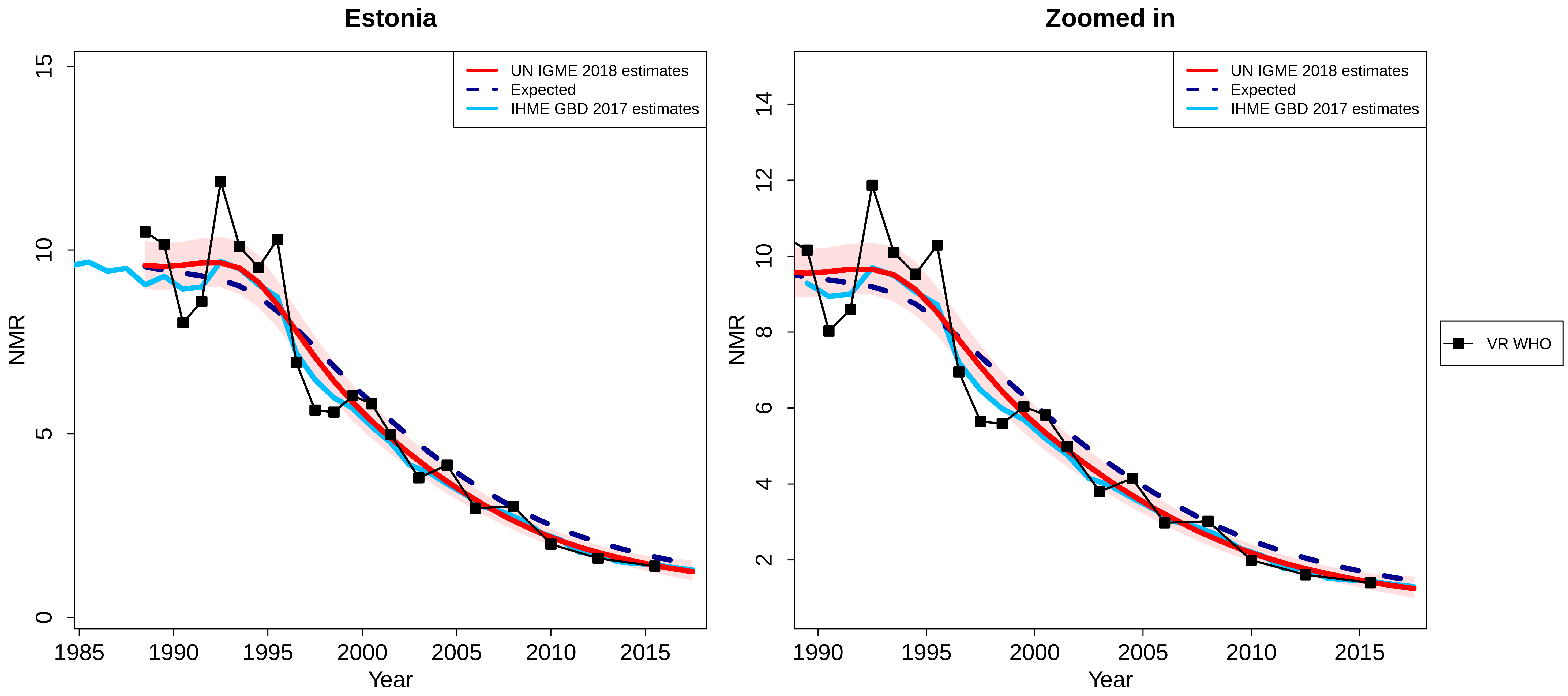

Estonia
Zoomed in
UN IGME 2018 estimates
Expected
IHME GBD 2017 estimates
VR WHO
NMR
Year
0
5
10
15
2
4
6
8
10
12
14
1985
1990
1995
2000
2005
2010
2015

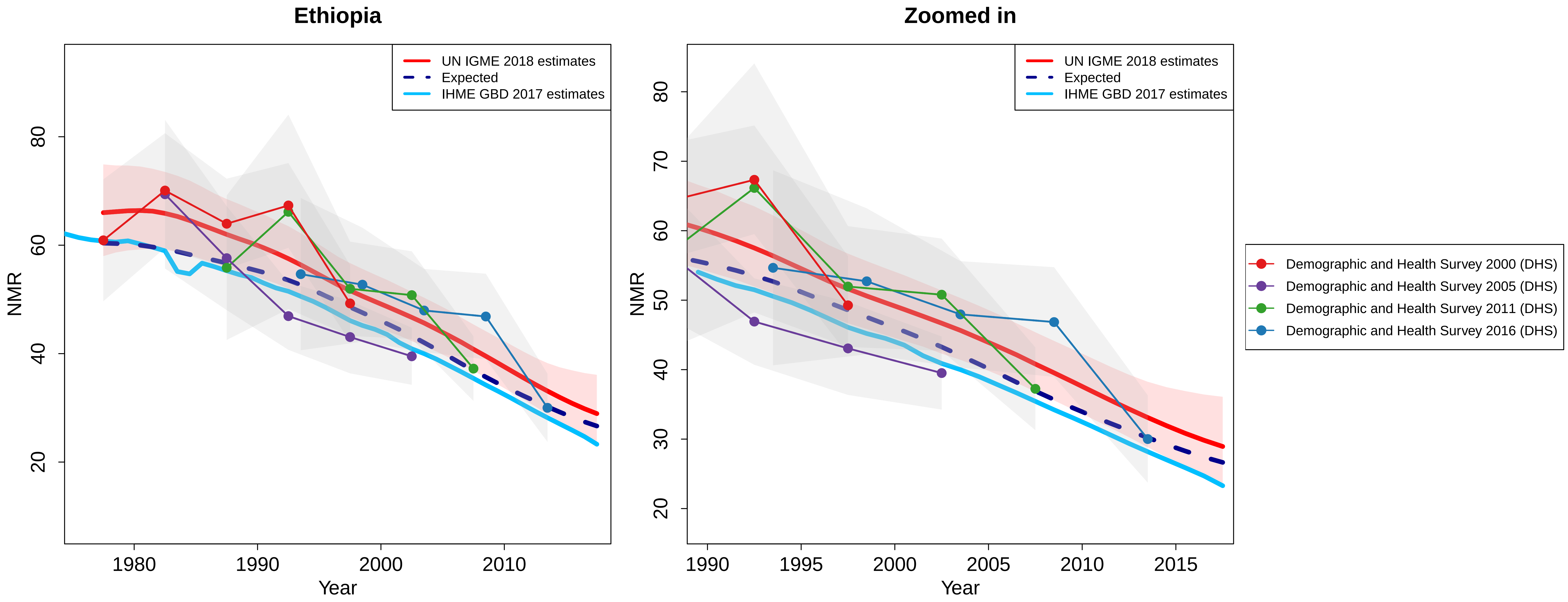

Ethiopia
Zoomed in
UN IGME 2018 estimates
Expected
IHME GBD 2017 estimates
NMR
Year
Demographic and Health Survey 2000 (DHS)
Demographic and Health Survey 2005 (DHS)
Demographic and Health Survey 2011 (DHS)
Demographic and Health Survey 2016 (DHS)

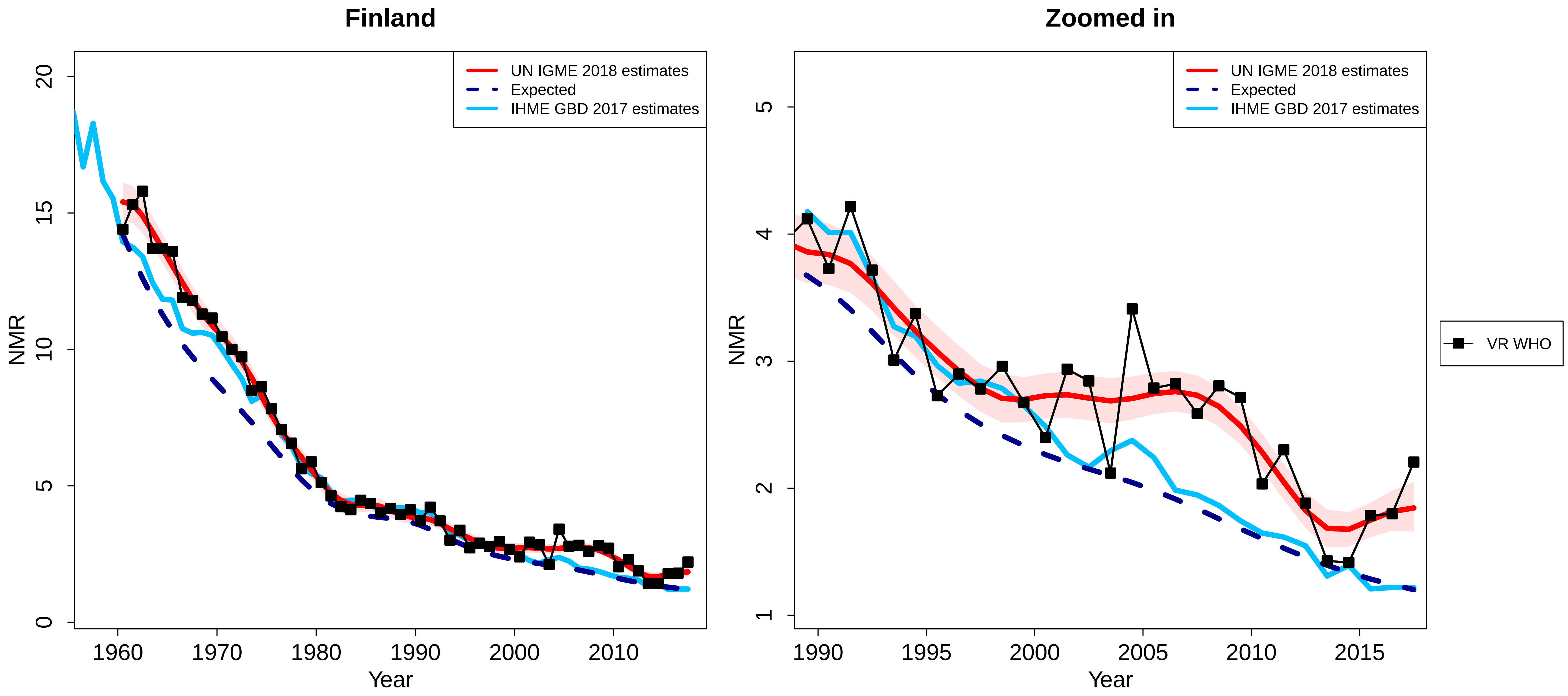
Finland
Zoomed in
UN IGME 2018 estimates
Expected
IHME GBD 2017 estimates
VR WHO
NMR
Year
0
5
10
15
20
1960
1970
1980
1990
2000
2010
1
2
3
4
5
1990
1995
2000
2005
2010
2015

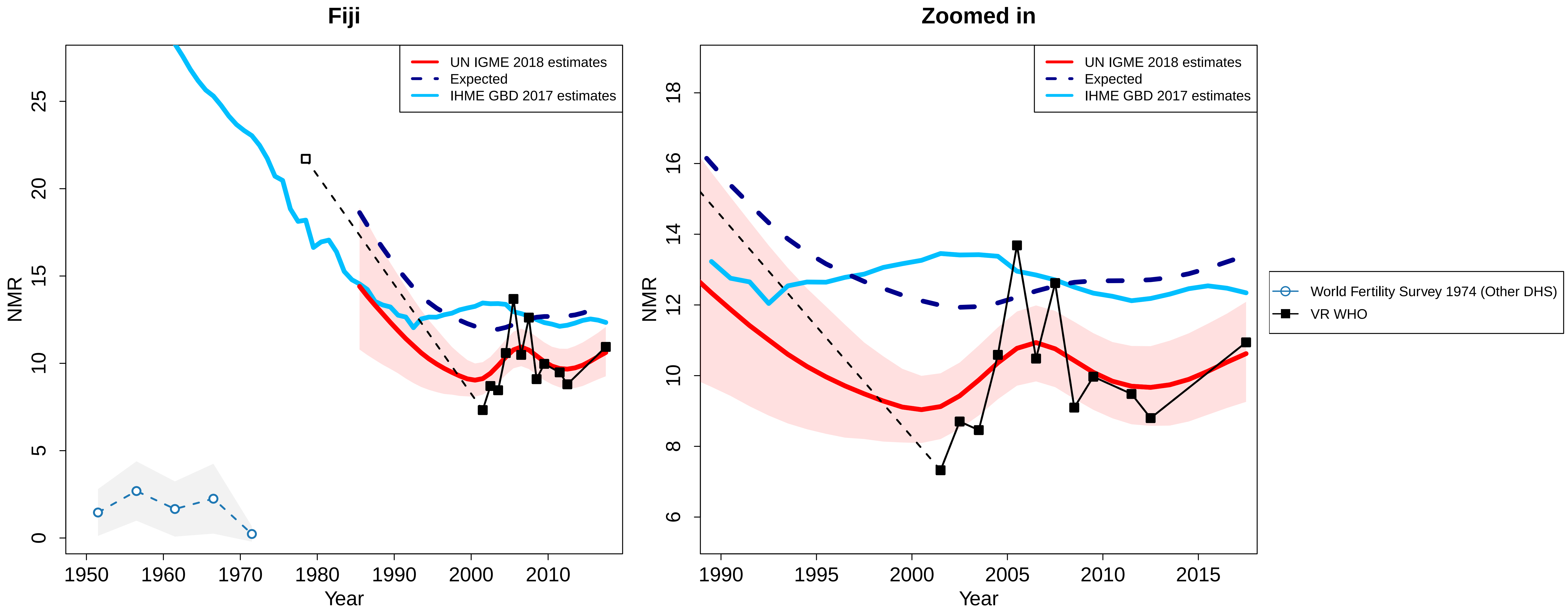

Fiji
Zoomed in
UN IGME 2018 estimates
Expected
IHME GBD 2017 estimates
World Fertility Survey 1974 (Other DHS)
VR WHO
NMR
Year

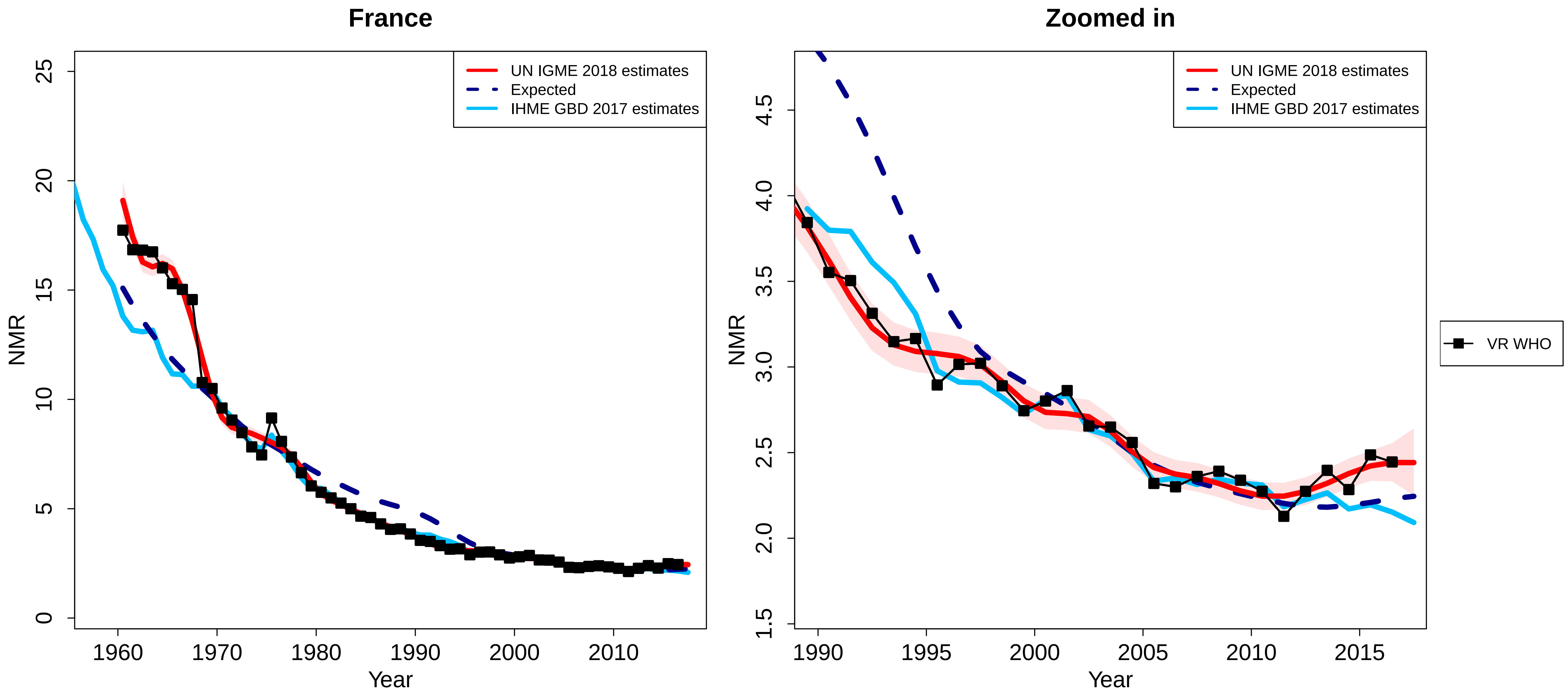

France
UN IGME 2018 estimates
Expected
IHME GBD 2017 estimates
NMR
0
5
10
15
20
25
1960
1970
1980
1990
2000
2010
Year
Zoomed in
UN IGME 2018 estimates
Expected
IHME GBD 2017 estimates
NMR
1.5
2.0
2.5
3.0
3.5
4.0
4.5
1990
1995
2000
2005
2010
2015
Year
VR WHO

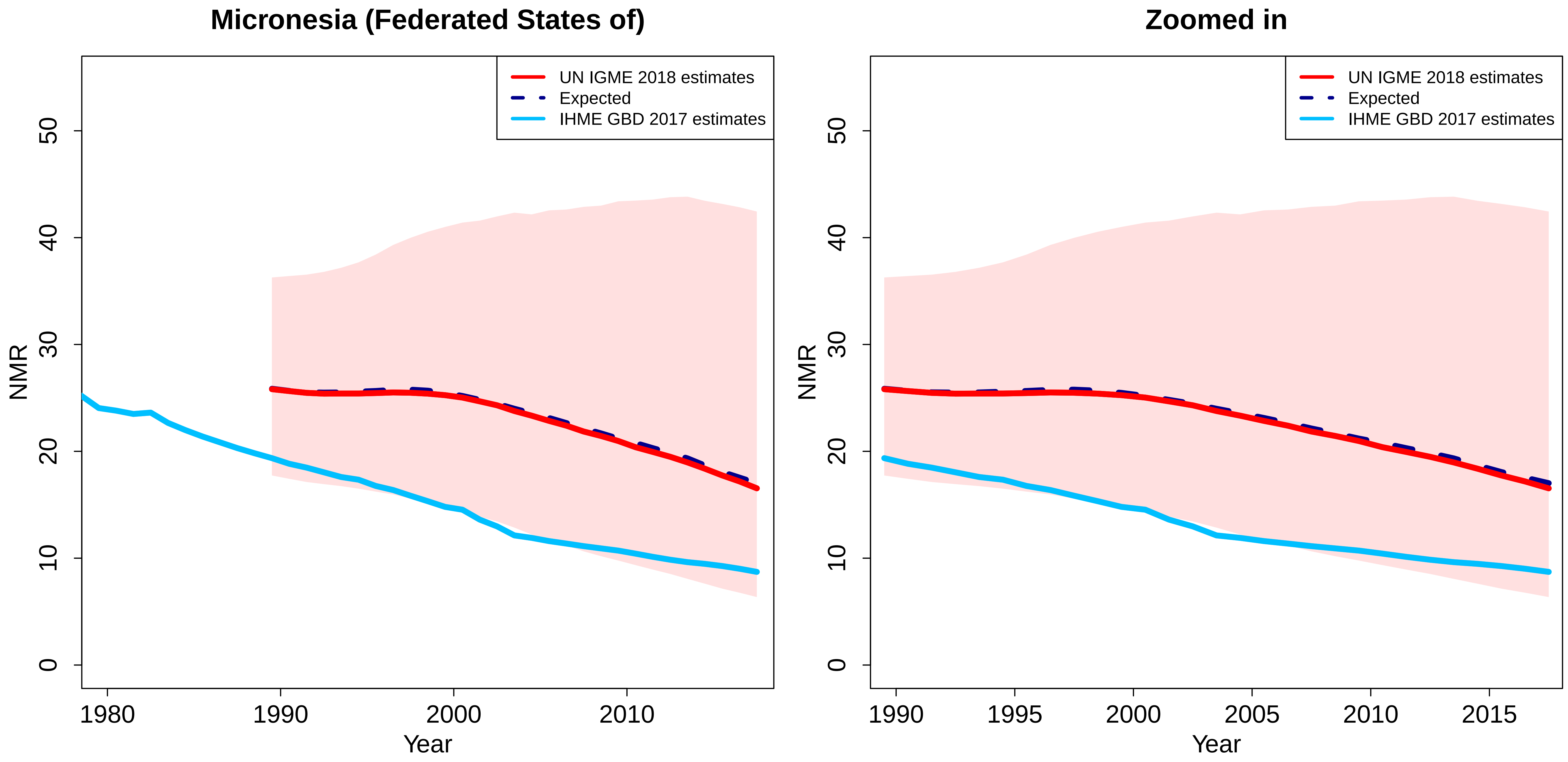
Micronesia (Federated States of)
Zoomed in
UN IGME 2018 estimates
Expected
IHME GBD 2017 estimates
NMR
Year
0
10
20
30
40
50
1980
1990
2000
2010
1995
2005
2015

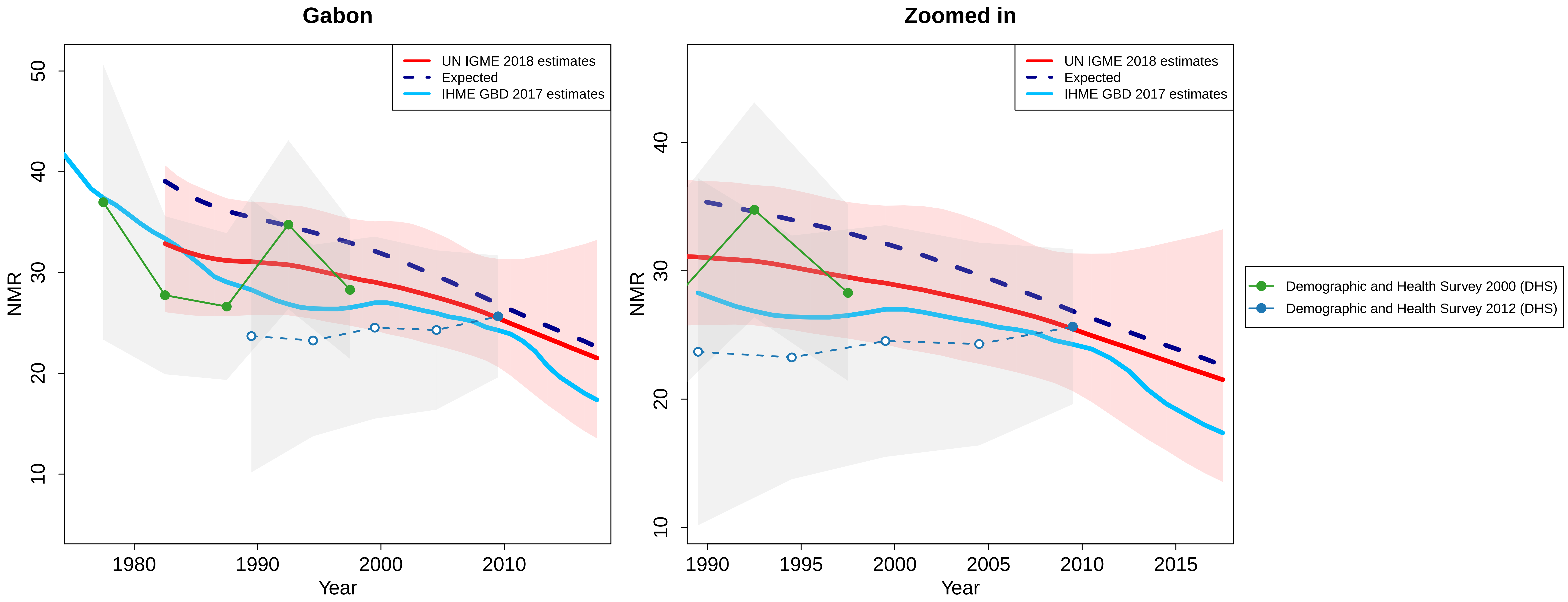
Gabon
Zoomed in
UN IGME 2018 estimates
Expected
IHME GBD 2017 estimates
Demographic and Health Survey 2000 (DHS)
Demographic and Health Survey 2012 (DHS)
NMR
Year
10
20
30
40
50
1980
1990
2000
2010
1995
2005
2015

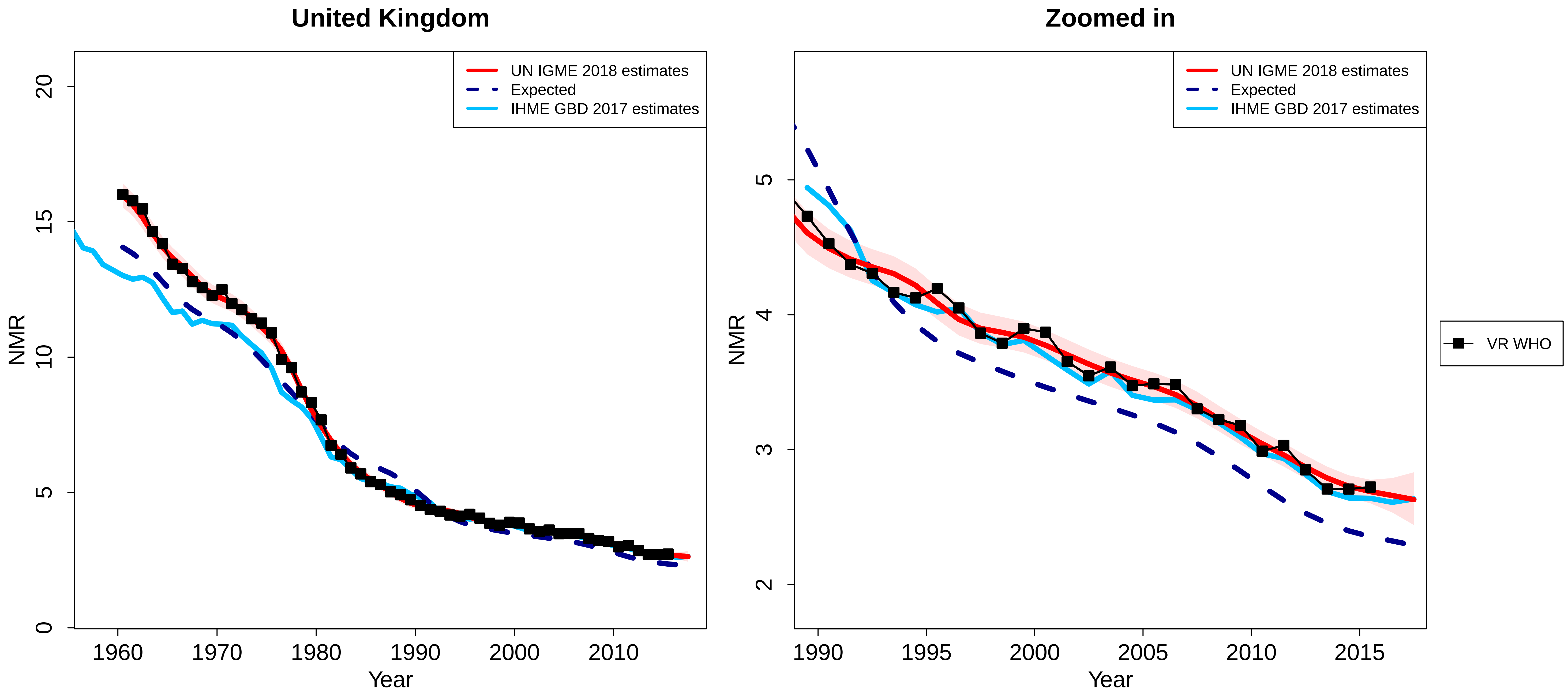
United Kingdom
Zoomed in
UN IGME 2018 estimates
Expected
IHME GBD 2017 estimates
VR WHO
NMR
Year
0
5
10
15
20
1960
1970
1980
1990
2000
2010
2
3
4
5
1990
1995
2000
2005
2010
2015

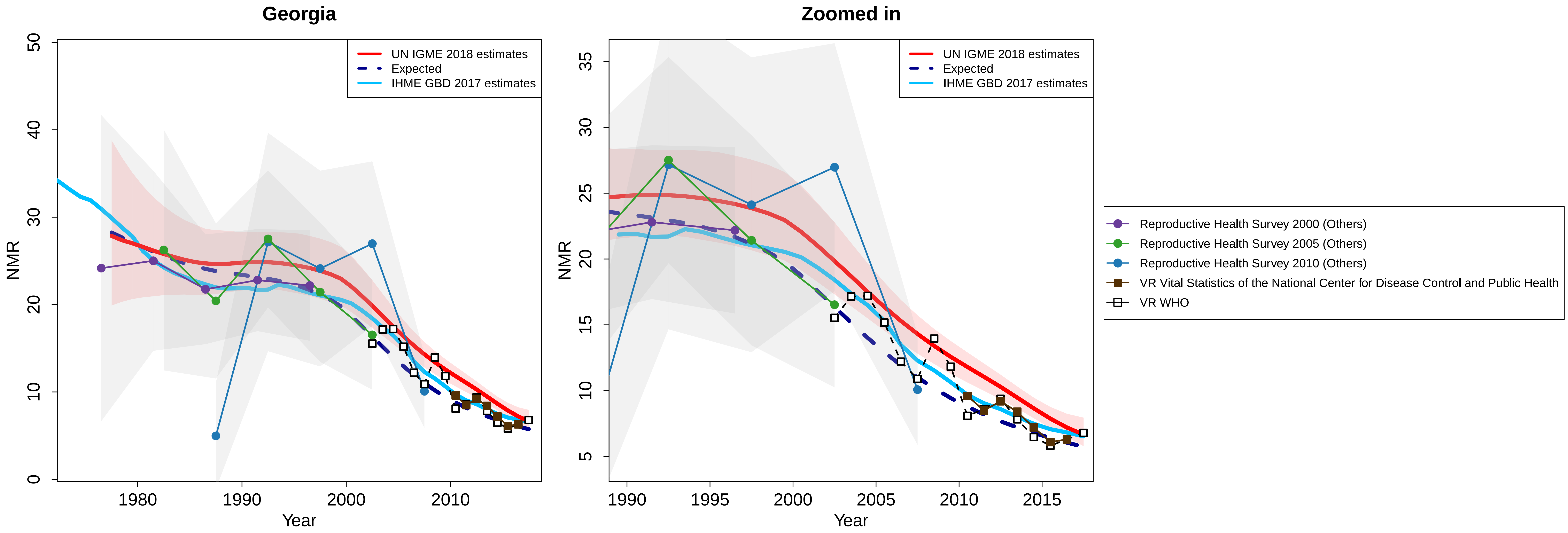
Georgia
Zoomed in
NMR
Year
UN IGME 2018 estimates
Expected
IHME GBD 2017 estimates
Reproductive Health Survey 2000 (Others)
Reproductive Health Survey 2005 (Others)
Reproductive Health Survey 2010 (Others)
VR Vital Statistics of the National Center for Disease Control and Public Health
VR WHO
0
10
20
30
40
50
1980
1990
2000
2010
5
15
25
35
1995
2005
2015

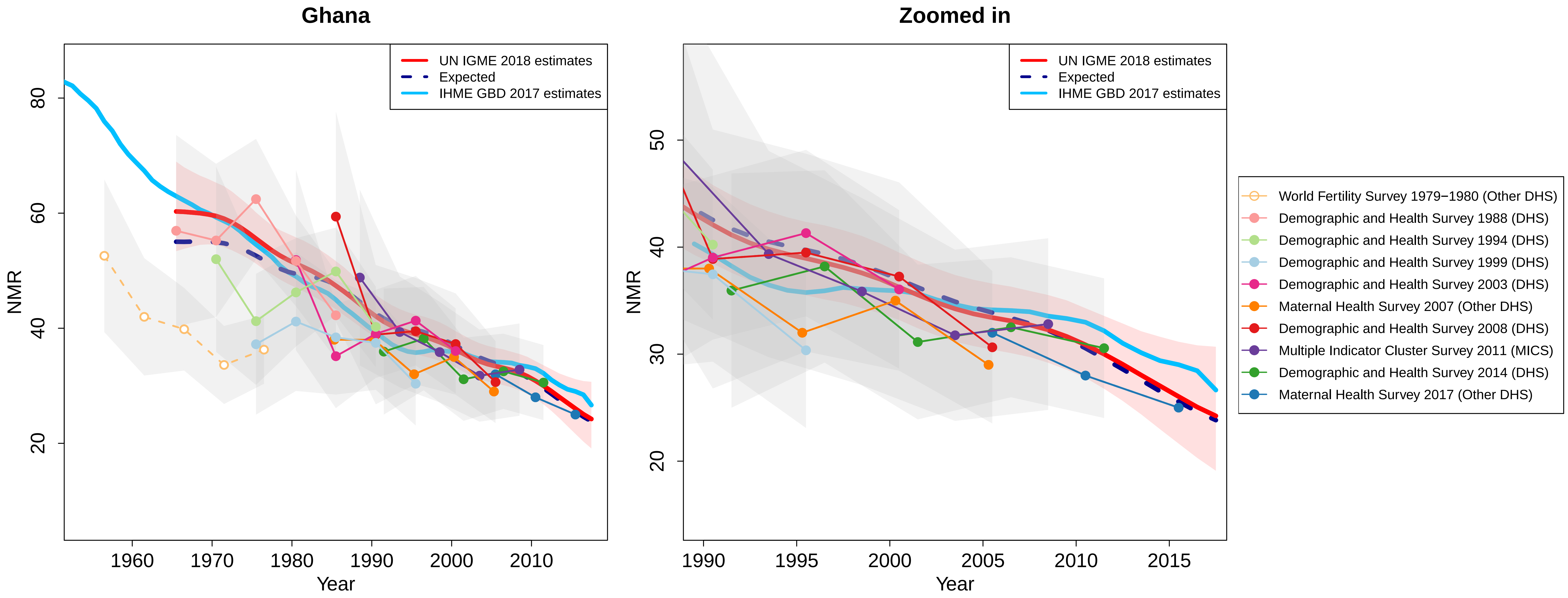

Ghana
Zoomed in
UN IGME 2018 estimates
Expected
IHME GBD 2017 estimates
NMR
Year
World Fertility Survey 1979−1980 (Other DHS)
Demographic and Health Survey 1988 (DHS)
Demographic and Health Survey 1994 (DHS)
Demographic and Health Survey 1999 (DHS)
Demographic and Health Survey 2003 (DHS)
Maternal Health Survey 2007 (Other DHS)
Demographic and Health Survey 2008 (DHS)
Multiple Indicator Cluster Survey 2011 (MICS)
Demographic and Health Survey 2014 (DHS)
Maternal Health Survey 2017 (Other DHS)

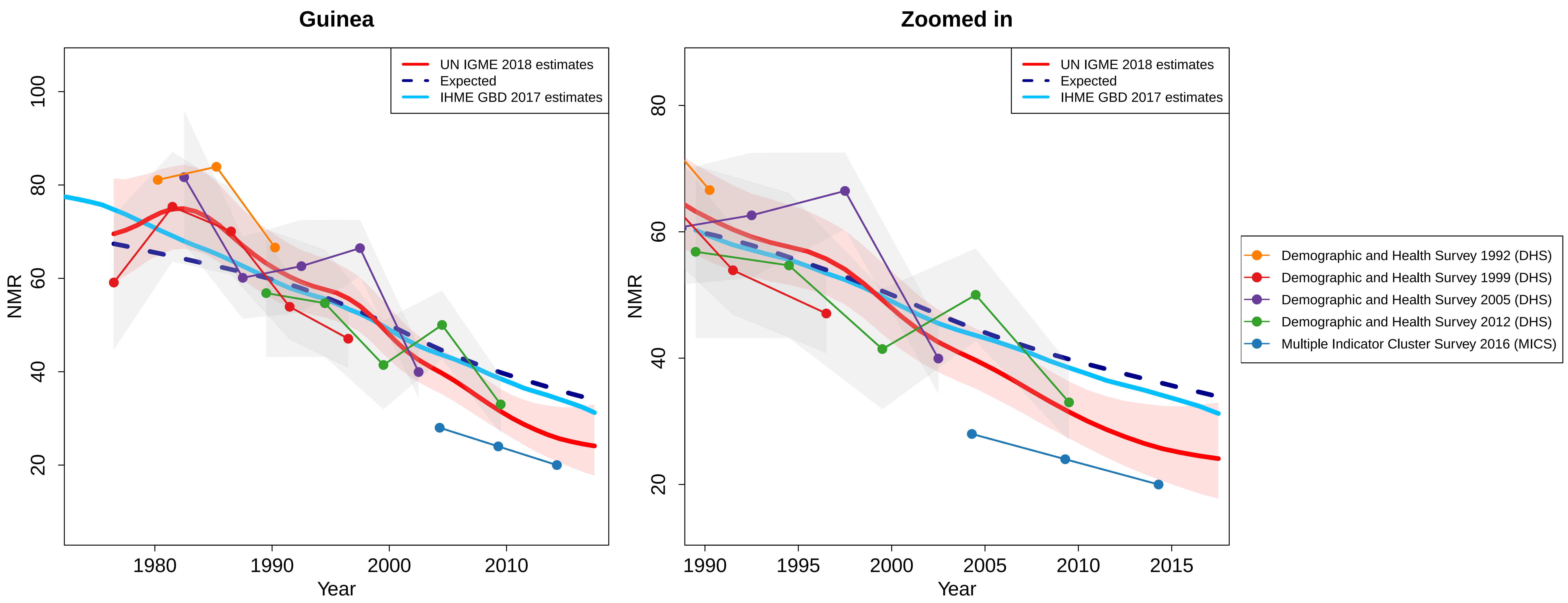

Guinea
Zoomed in
UN IGME 2018 estimates
Expected
IHME GBD 2017 estimates
NMR
Year
Demographic and Health Survey 1992 (DHS)
Demographic and Health Survey 1999 (DHS)
Demographic and Health Survey 2005 (DHS)
Demographic and Health Survey 2012 (DHS)
Multiple Indicator Cluster Survey 2016 (MICS)

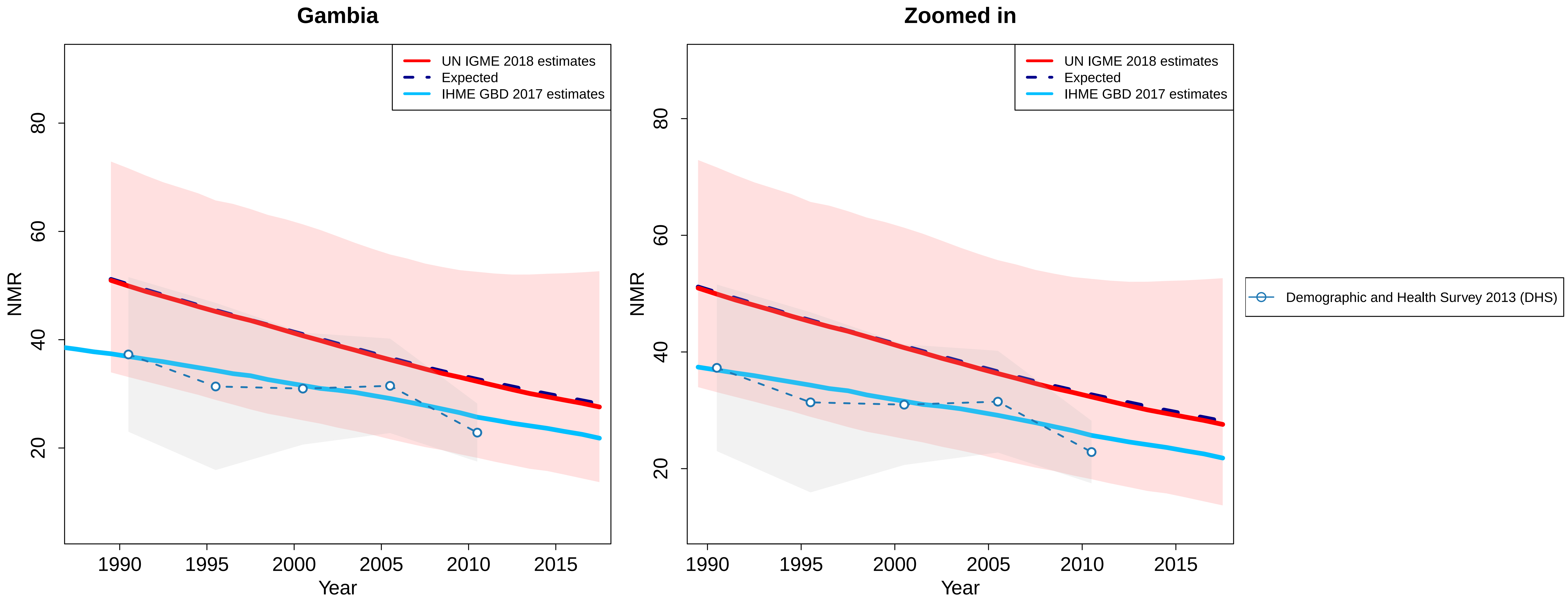

Gambia
UN IGME 2018 estimates
Expected
IHME GBD 2017 estimates
NMR
80
60
40
20
1990
1995
2000
2005
2010
2015
Year
Zoomed in
UN IGME 2018 estimates
Expected
IHME GBD 2017 estimates
NMR
80
60
40
20
1990
1995
2000
2005
2010
2015
Year
Demographic and Health Survey 2013 (DHS)

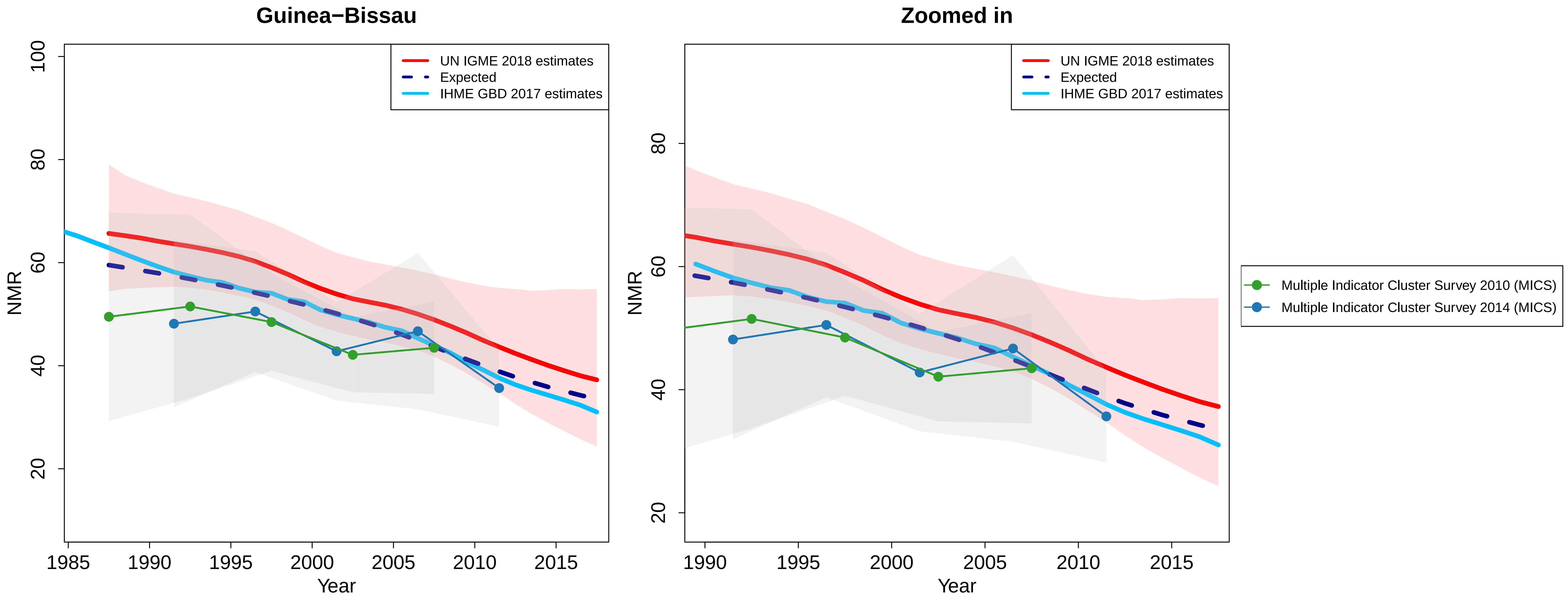
Guinea−Bissau
Zoomed in
UN IGME 2018 estimates
Expected
IHME GBD 2017 estimates
Multiple Indicator Cluster Survey 2010 (MICS)
Multiple Indicator Cluster Survey 2014 (MICS)
NMR
Year
1985
1990
1995
2000
2005
2010
2015
20
40
60
80
100

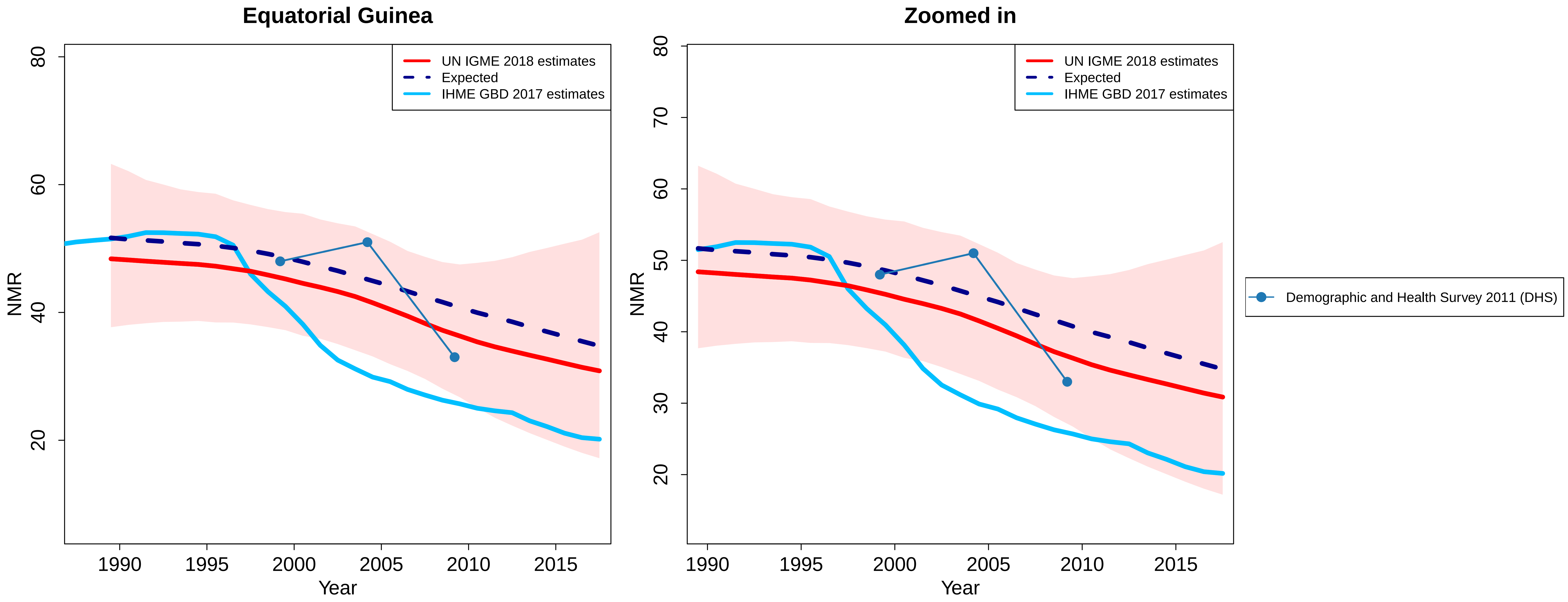

Equatorial Guinea
Zoomed in
UN IGME 2018 estimates
Expected
IHME GBD 2017 estimates
Demographic and Health Survey 2011 (DHS)
NMR
Year

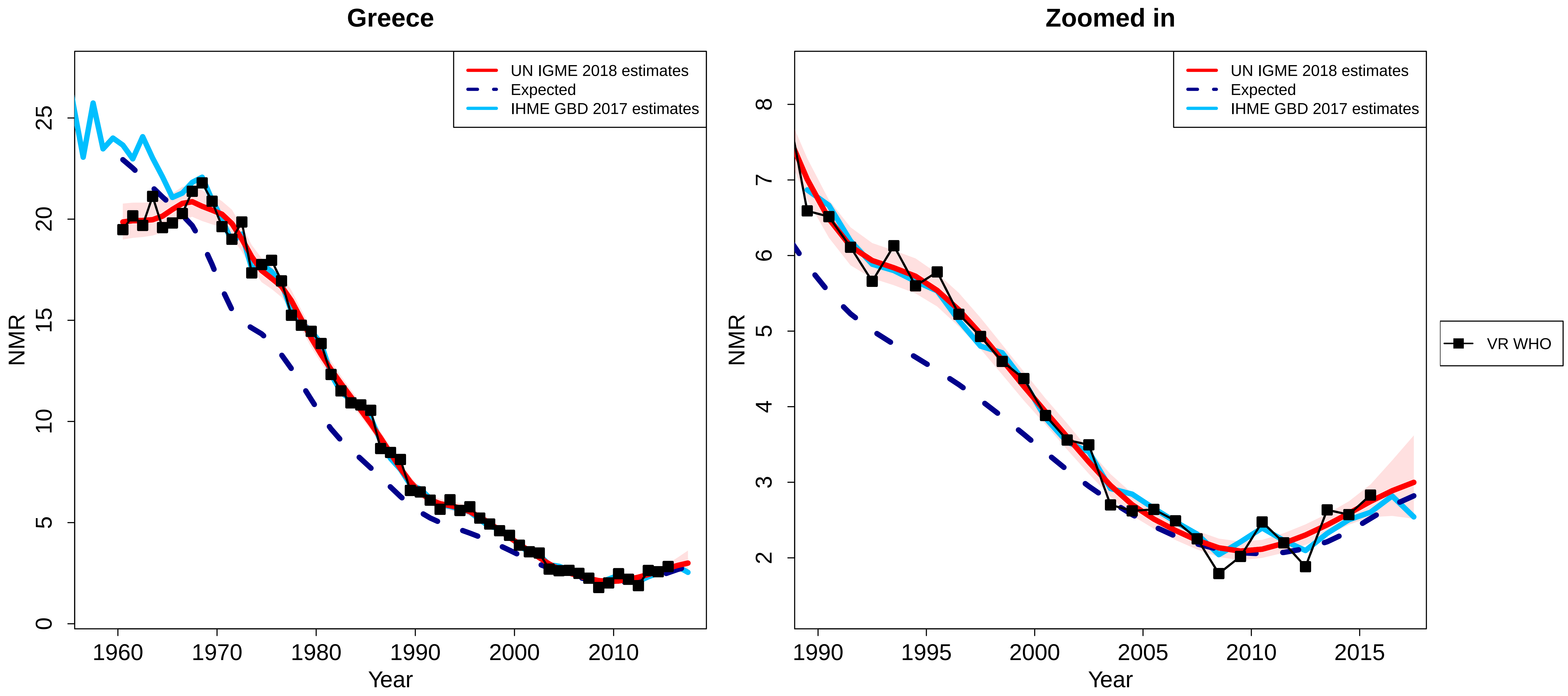
Greece
Zoomed in
UN IGME 2018 estimates
Expected
IHME GBD 2017 estimates
VR WHO
NMR
Year
0
5
10
15
20
25
1960
1970
1980
1990
2000
2010
2
3
4
5
6
7
8
1995
2005
2015

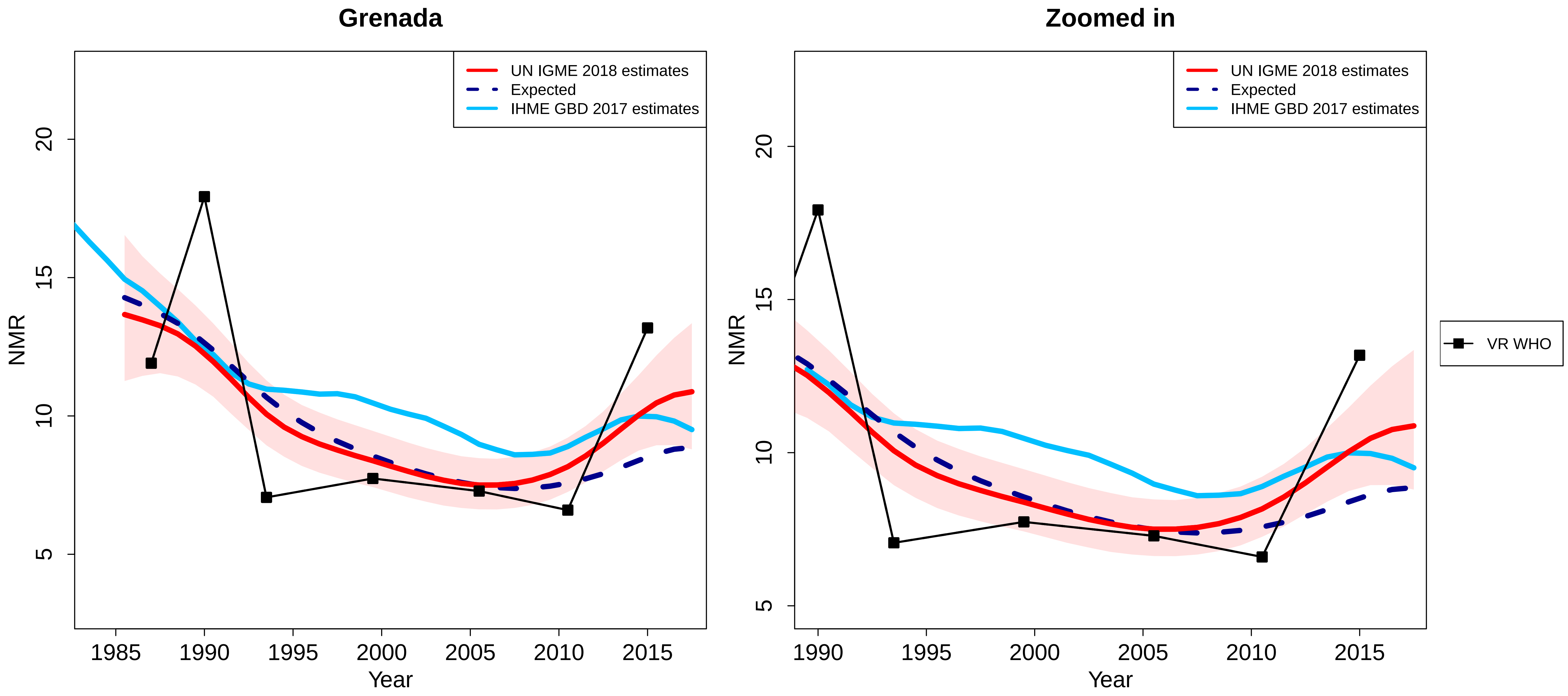
Grenada
Zoomed in
UN IGME 2018 estimates
Expected
IHME GBD 2017 estimates
VR WHO
NMR
Year
1985
1990
1995
2000
2005
2010
2015
5
10
15
20

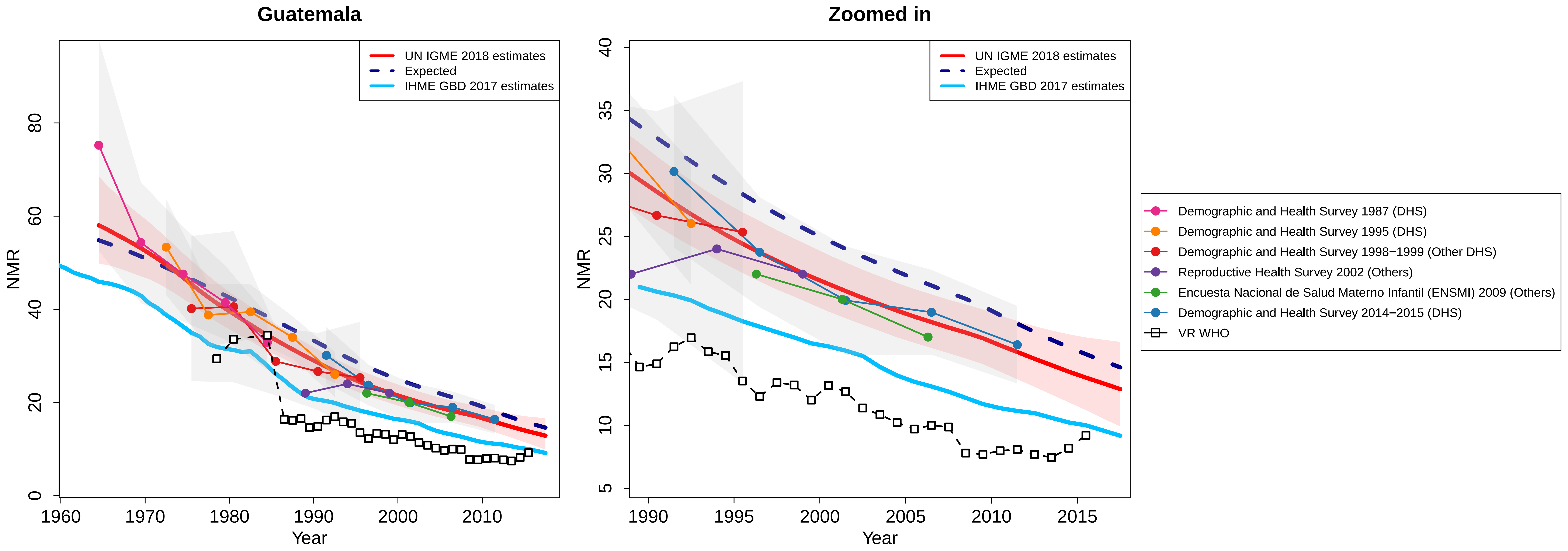
Guatemala
Zoomed in
UN IGME 2018 estimates
Expected
IHME GBD 2017 estimates
NMR
Year
1960
1970
1980
1990
2000
2010
0
20
40
60
80
1990
1995
2000
2005
2010
2015
5
10
15
20
25
30
35
40
Demographic and Health Survey 1987 (DHS)
Demographic and Health Survey 1995 (DHS)
Demographic and Health Survey 1998−1999 (Other DHS)
Reproductive Health Survey 2002 (Others)
Encuesta Nacional de Salud Materno Infantil (ENSMI) 2009 (Others)
Demographic and Health Survey 2014−2015 (DHS)
VR WHO

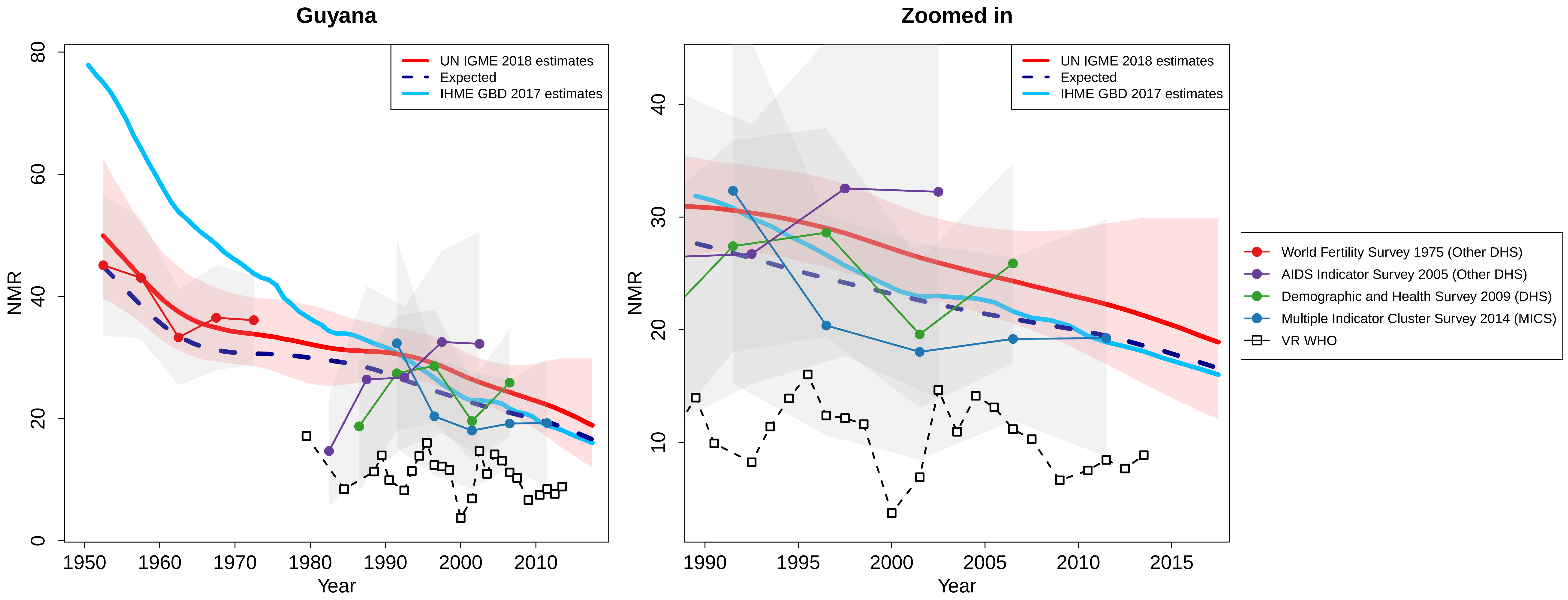
Guyana
Zoomed in
UN IGME 2018 estimates
Expected
IHME GBD 2017 estimates
World Fertility Survey 1975 (Other DHS)
AIDS Indicator Survey 2005 (Other DHS)
Demographic and Health Survey 2009 (DHS)
Multiple Indicator Cluster Survey 2014 (MICS)
VR WHO
NMR
Year
0
20
40
60
80
1950
1960
1970
1980
1990
2000
2010
10
30
1995
2005
2015

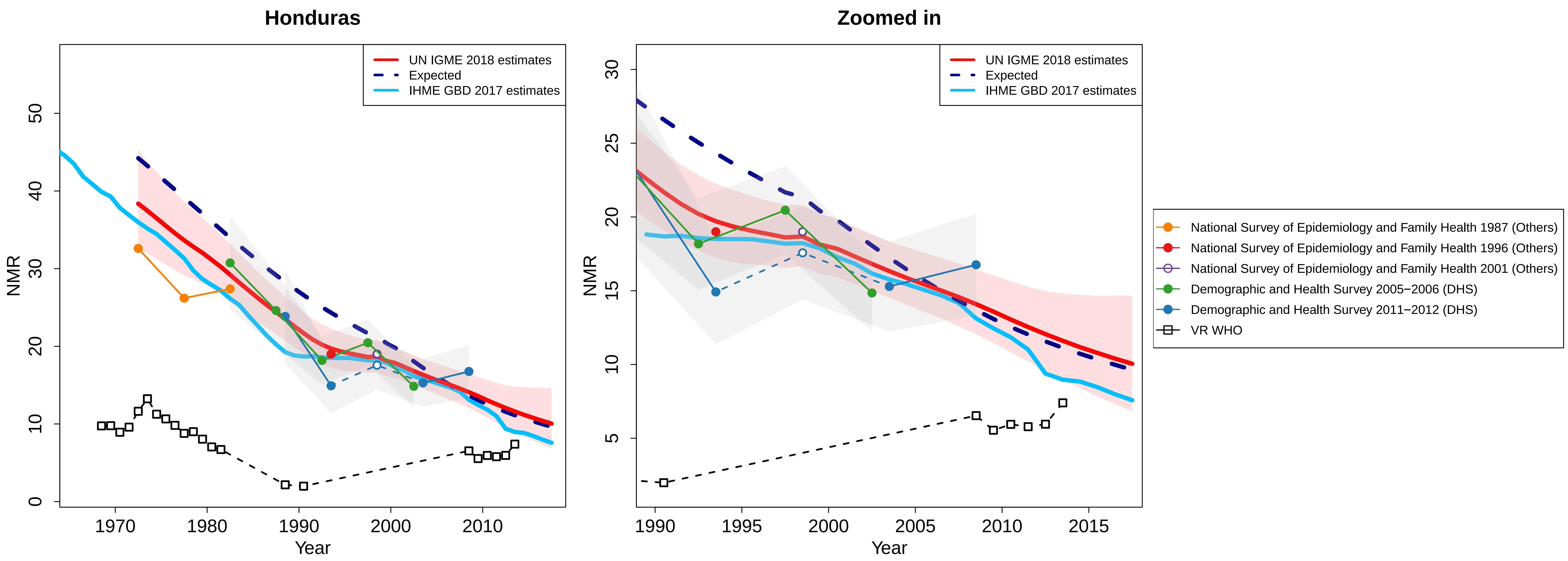
Honduras
Zoomed in
UN IGME 2018 estimates
Expected
IHME GBD 2017 estimates
NMR
Year
National Survey of Epidemiology and Family Health 1987 (Others)
National Survey of Epidemiology and Family Health 1996 (Others)
National Survey of Epidemiology and Family Health 2001 (Others)
Demographic and Health Survey 2005–2006 (DHS)
Demographic and Health Survey 2011–2012 (DHS)
VR WHO

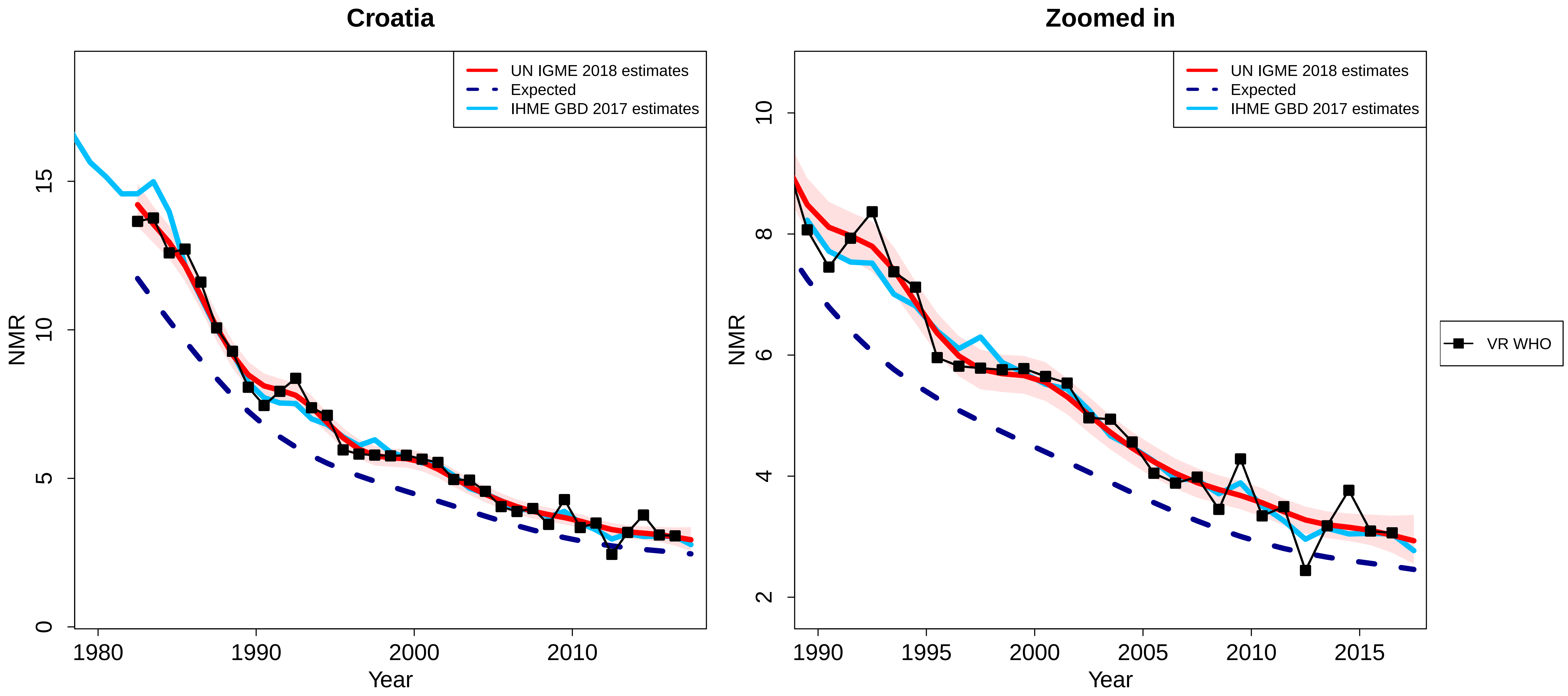
Croatia
UN IGME 2018 estimates
Expected
IHME GBD 2017 estimates
NMR
0
5
10
15
1980
1990
2000
2010
Year
Zoomed in
UN IGME 2018 estimates
Expected
IHME GBD 2017 estimates
NMR
2
4
6
8
10
1990
1995
2000
2005
2010
2015
Year
VR WHO

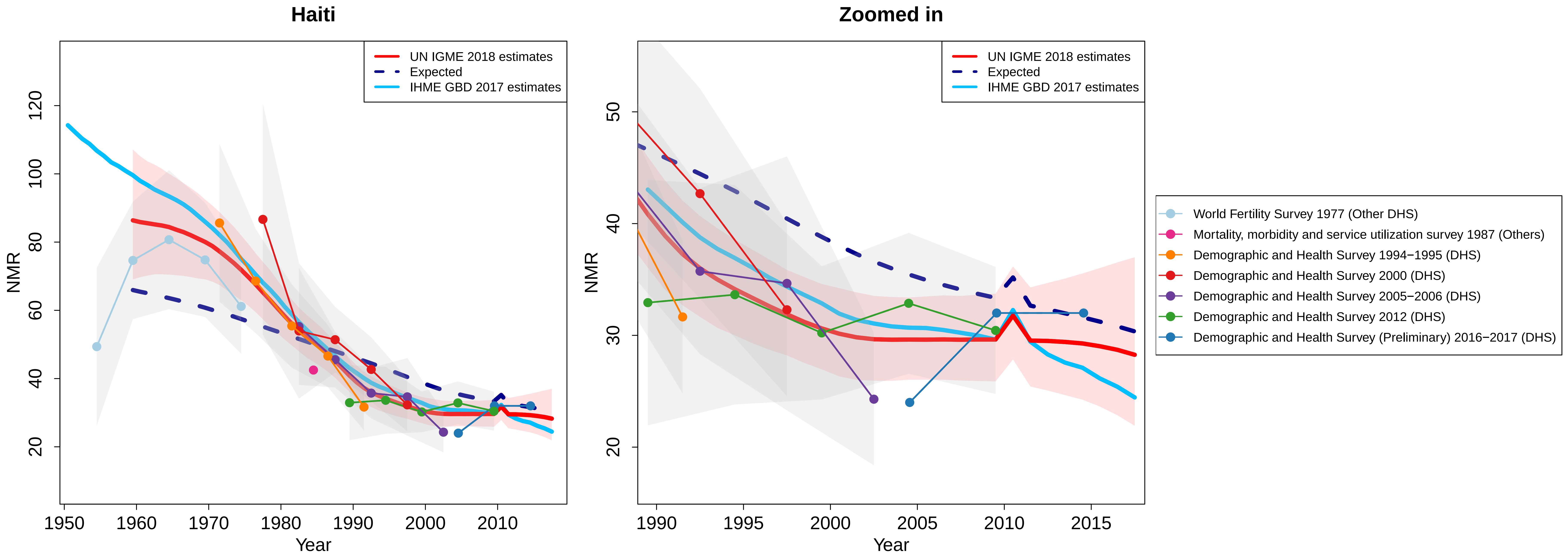

Haiti
Zoomed in
UN IGME 2018 estimates
Expected
IHME GBD 2017 estimates
NMR
Year
World Fertility Survey 1977 (Other DHS)
Mortality, morbidity and service utilization survey 1987 (Others)
Demographic and Health Survey 1994−1995 (DHS)
Demographic and Health Survey 2000 (DHS)
Demographic and Health Survey 2005−2006 (DHS)
Demographic and Health Survey 2012 (DHS)
Demographic and Health Survey (Preliminary) 2016−2017 (DHS)

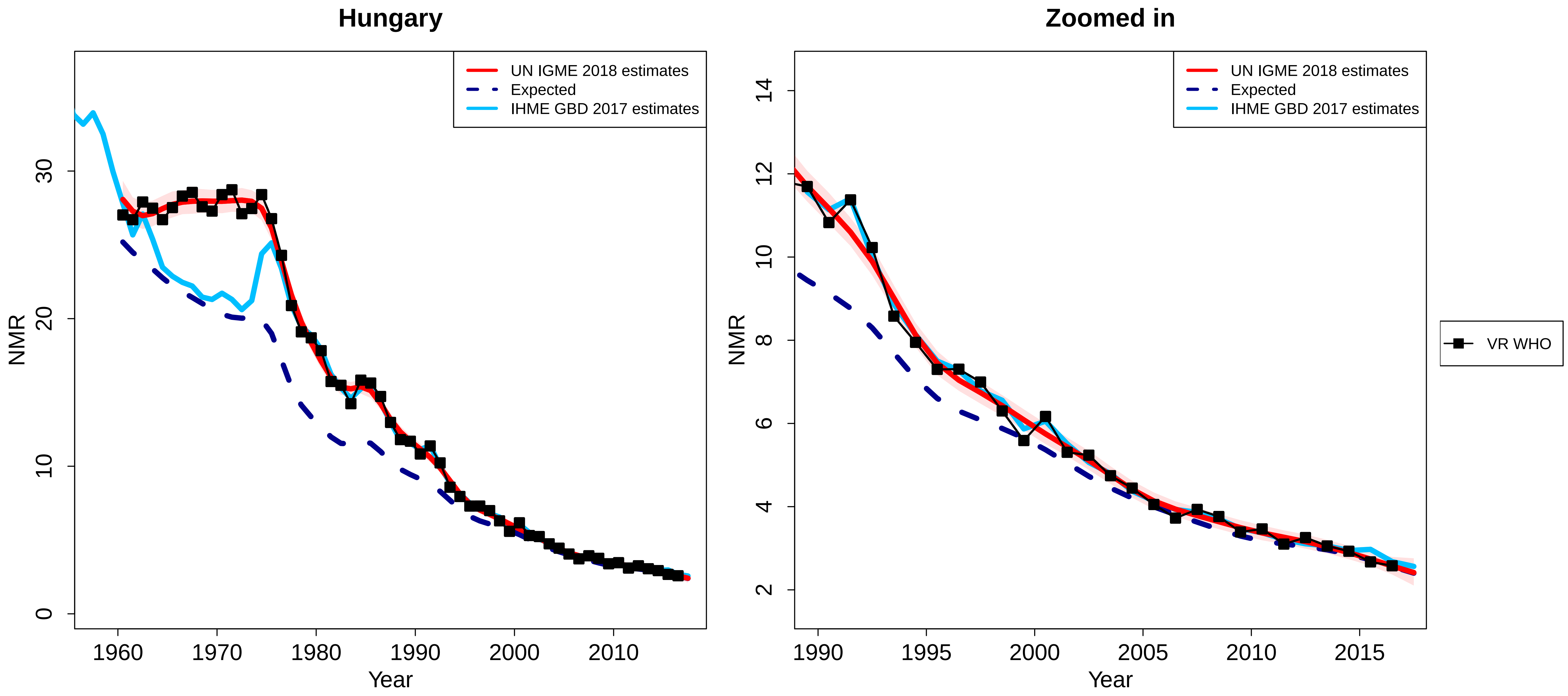
Hungary
Zoomed in
UN IGME 2018 estimates
Expected
IHME GBD 2017 estimates
UN IGME 2018 estimates
Expected
IHME GBD 2017 estimates
VR WHO
NMR
NMR
Year
Year
0
10
20
30
2
4
6
8
10
12
14
1960
1970
1980
1990
2000
2010
1990
1995
2000
2005
2010
2015

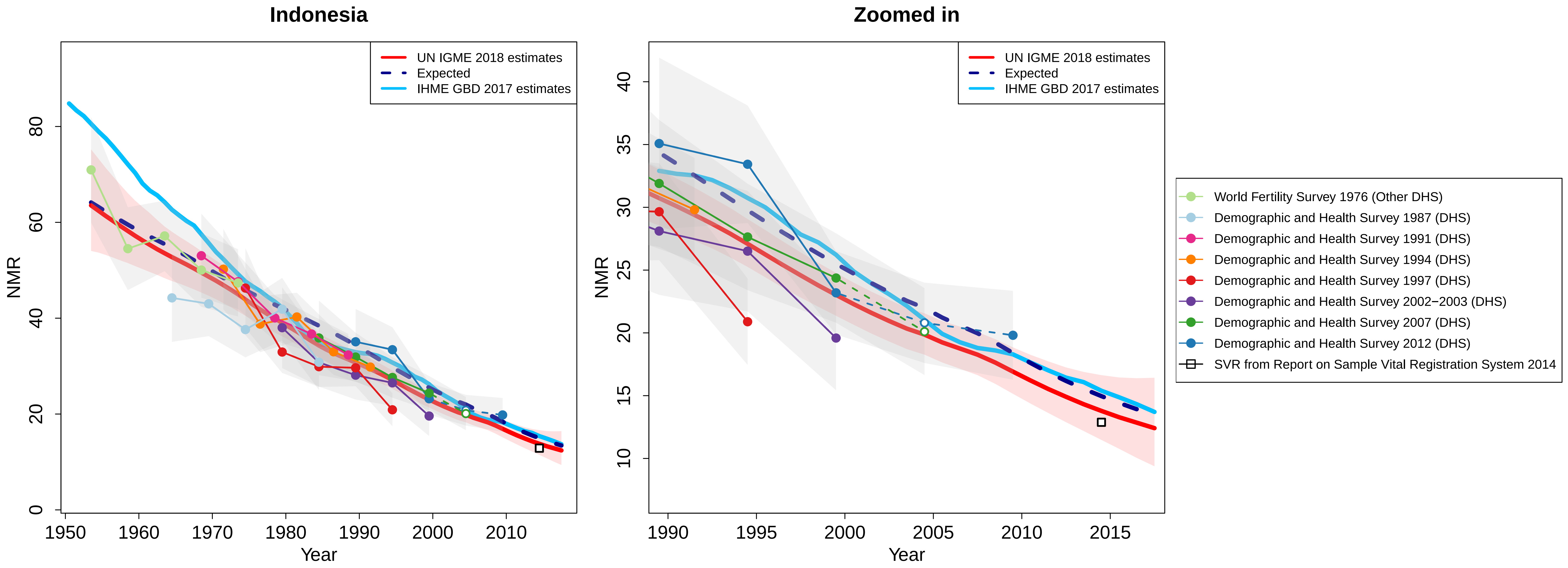

Indonesia
Zoomed in
NMR
Year
UN IGME 2018 estimates
Expected
IHME GBD 2017 estimates
World Fertility Survey 1976 (Other DHS)
Demographic and Health Survey 1987 (DHS)
Demographic and Health Survey 1991 (DHS)
Demographic and Health Survey 1994 (DHS)
Demographic and Health Survey 1997 (DHS)
Demographic and Health Survey 2002−2003 (DHS)
Demographic and Health Survey 2007 (DHS)
Demographic and Health Survey 2012 (DHS)
SVR from Report on Sample Vital Registration System 2014

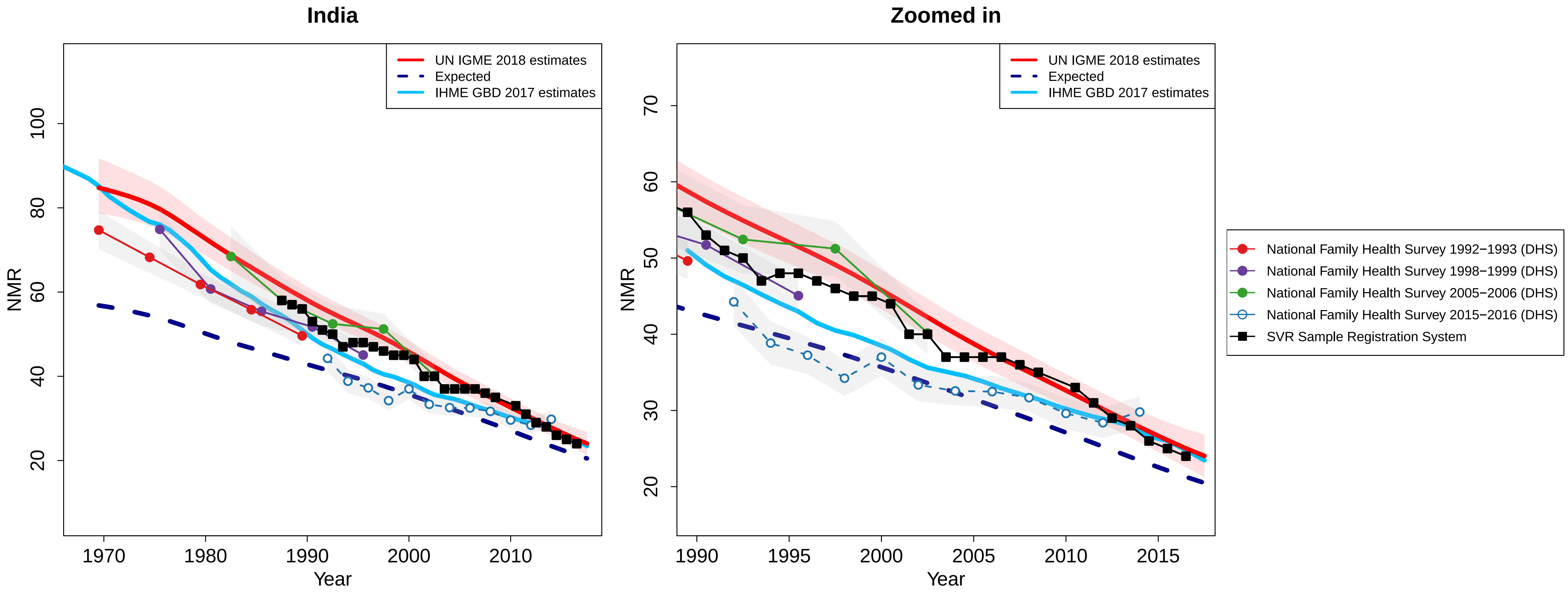
India
Zoomed in
UN IGME 2018 estimates
Expected
IHME GBD 2017 estimates
NMR
Year
National Family Health Survey 1992−1993 (DHS)
National Family Health Survey 1998−1999 (DHS)
National Family Health Survey 2005−2006 (DHS)
National Family Health Survey 2015−2016 (DHS)
SVR Sample Registration System

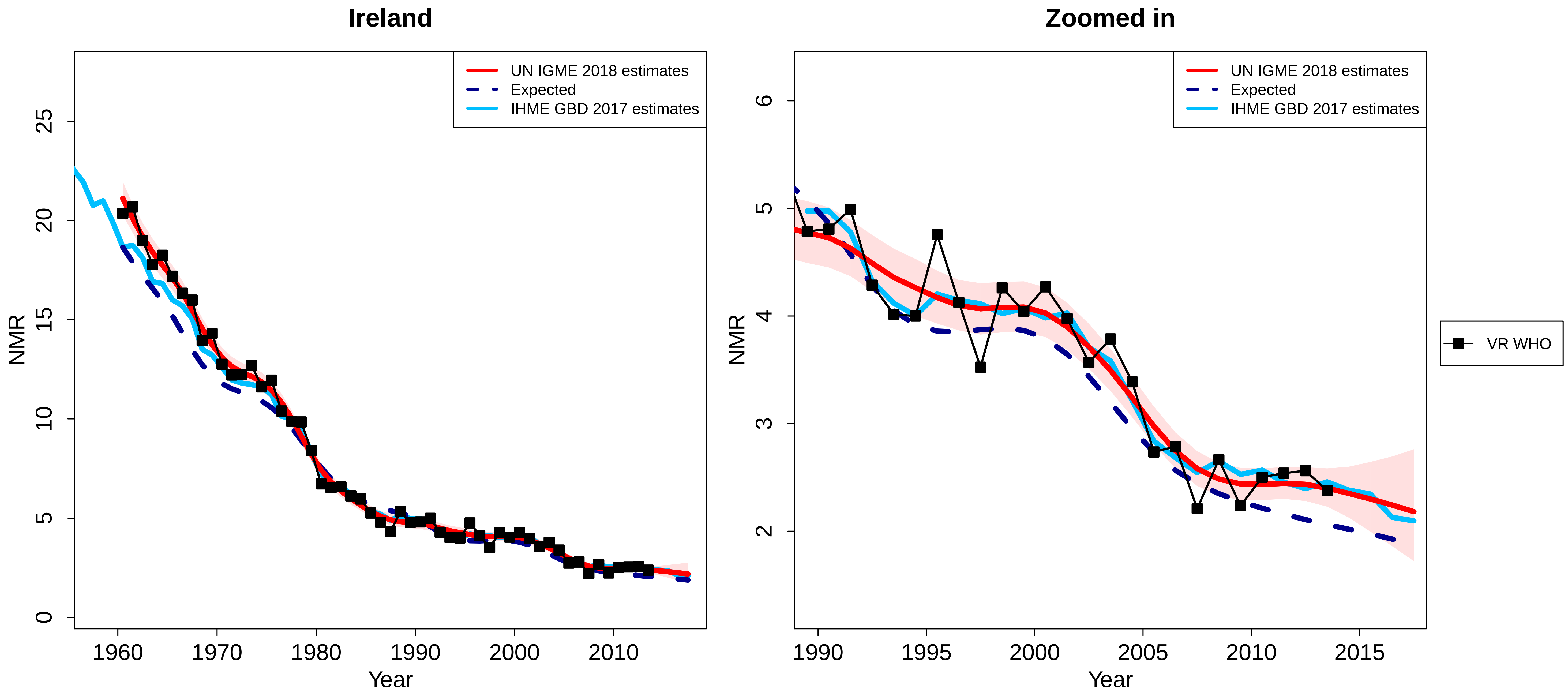
Ireland
UN IGME 2018 estimates
Expected
IHME GBD 2017 estimates
NMR
Year
Zoomed in
UN IGME 2018 estimates
Expected
IHME GBD 2017 estimates
NMR
Year
VR WHO

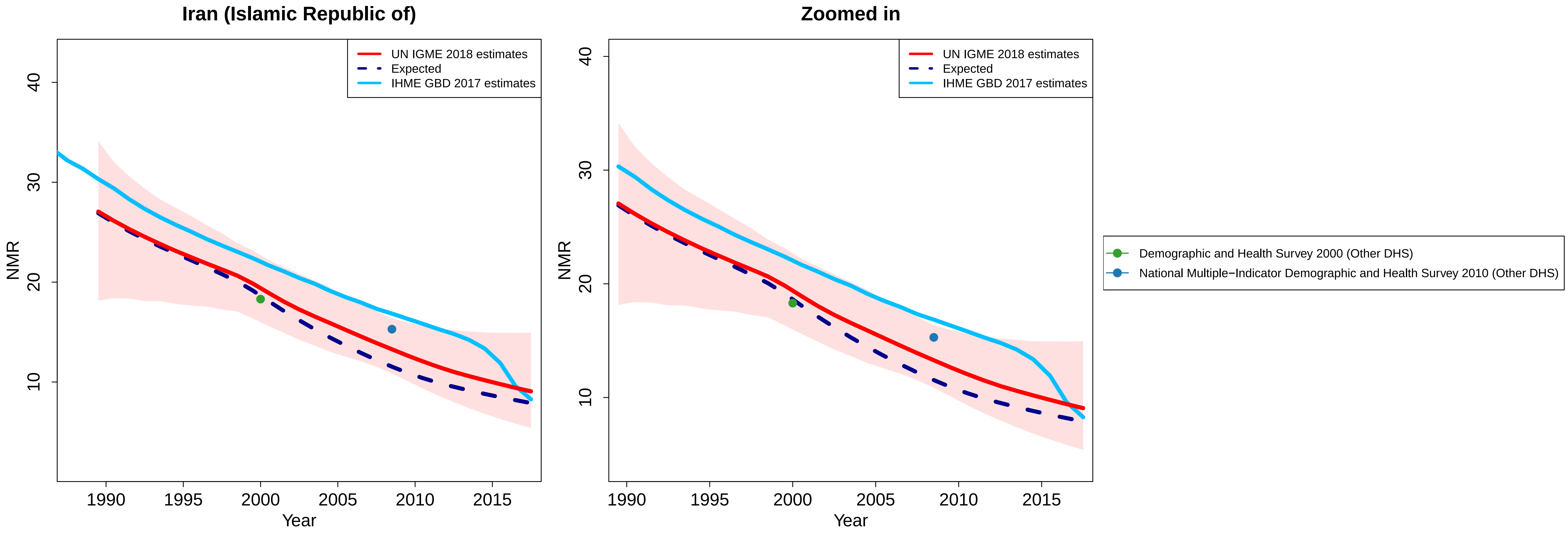

Iran (Islamic Republic of)
Zoomed in
UN IGME 2018 estimates
Expected
IHME GBD 2017 estimates
NMR
Year
Demographic and Health Survey 2000 (Other DHS)
National Multiple−Indicator Demographic and Health Survey 2010 (Other DHS)

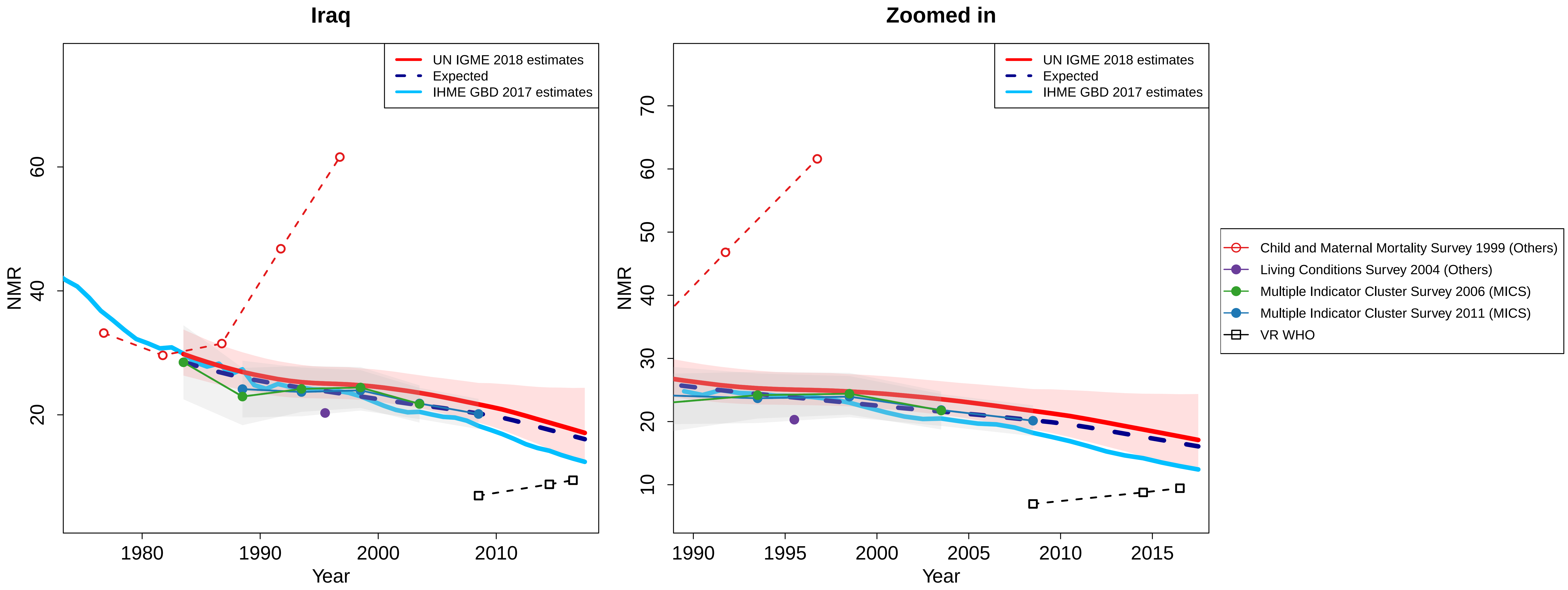
Iraq
Zoomed in
UN IGME 2018 estimates
Expected
IHME GBD 2017 estimates
NMR
Year
1980
1990
2000
2010
20
40
60
1995
2005
2015
10
30
50
70
Child and Maternal Mortality Survey 1999 (Others)
Living Conditions Survey 2004 (Others)
Multiple Indicator Cluster Survey 2006 (MICS)
Multiple Indicator Cluster Survey 2011 (MICS)
VR WHO

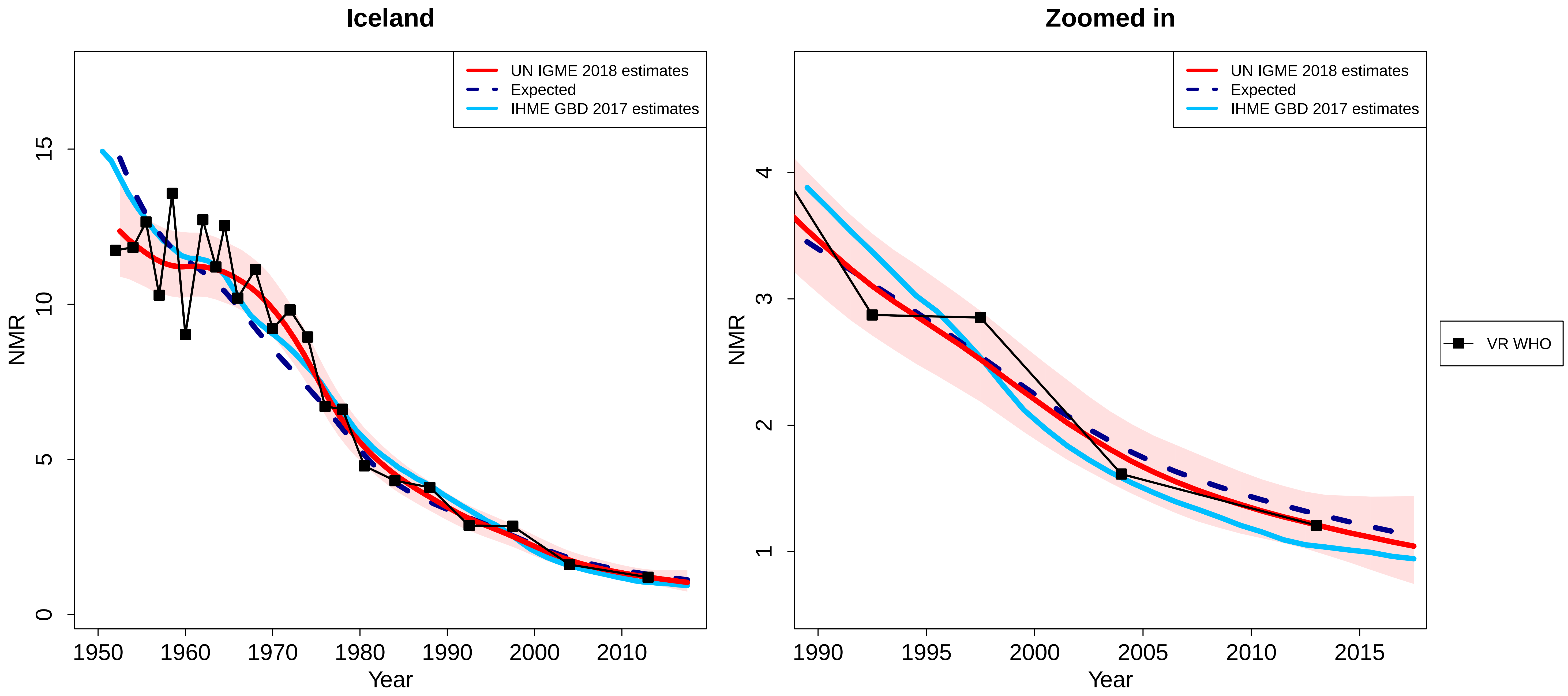

Iceland
Zoomed in
UN IGME 2018 estimates
Expected
IHME GBD 2017 estimates
VR WHO
NMR
Year
0
5
10
15
1950
1960
1970
1980
1990
2000
2010
1
2
3
4
1990
1995
2000
2005
2010
2015

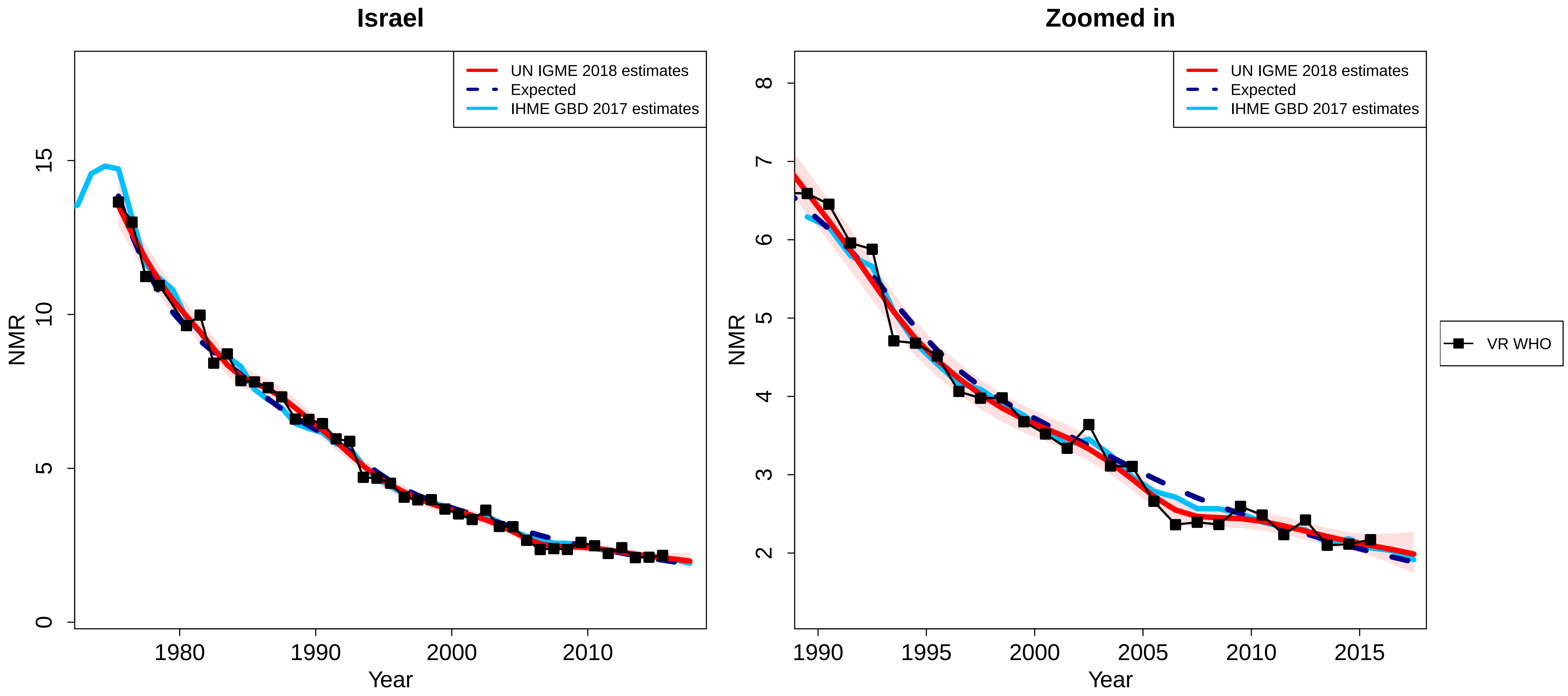
Israel
Zoomed in
UN IGME 2018 estimates
Expected
IHME GBD 2017 estimates
VR WHO
NMR
Year
0
5
10
15
1980
1990
2000
2010
2
3
4
5
6
7
8
1990
1995
2000
2005
2010
2015

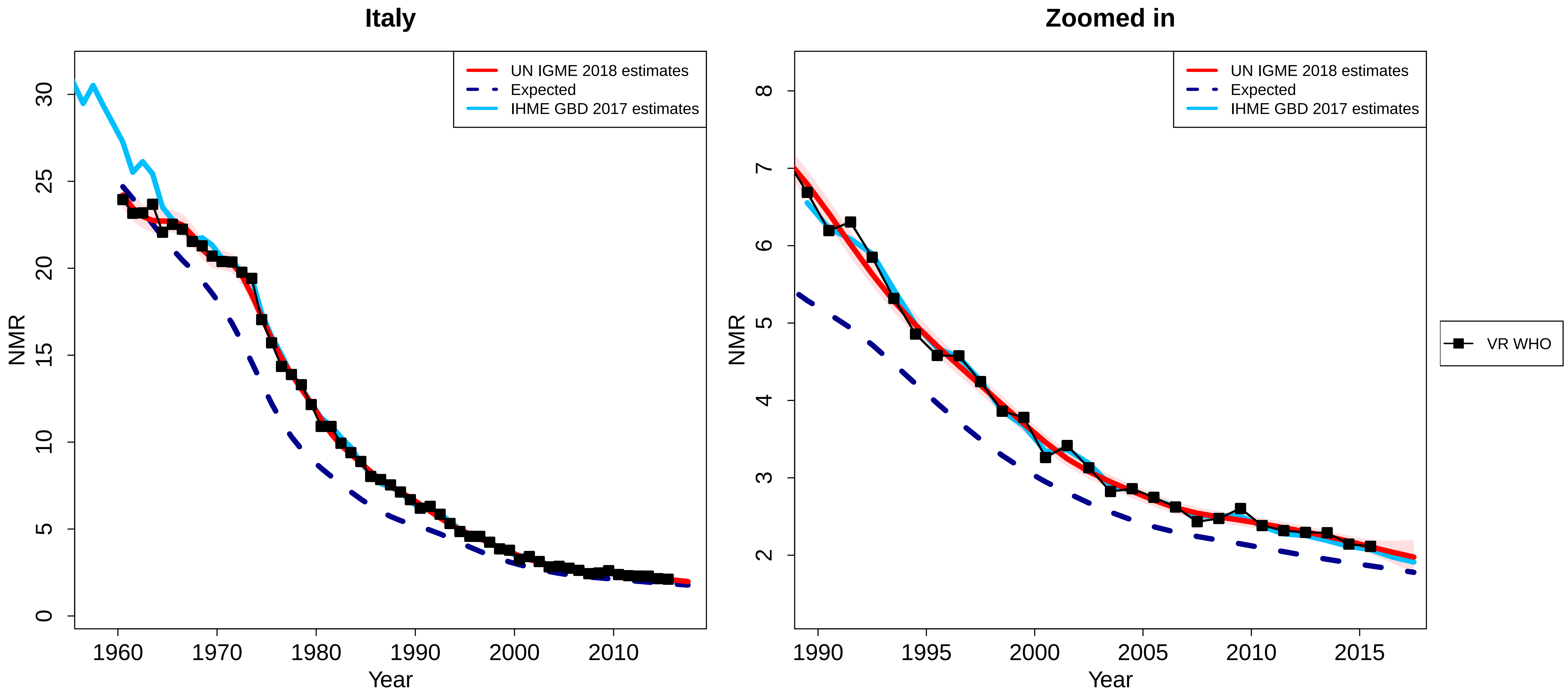

Italy
Zoomed in
UN IGME 2018 estimates
Expected
IHME GBD 2017 estimates
VR WHO
NMR
Year

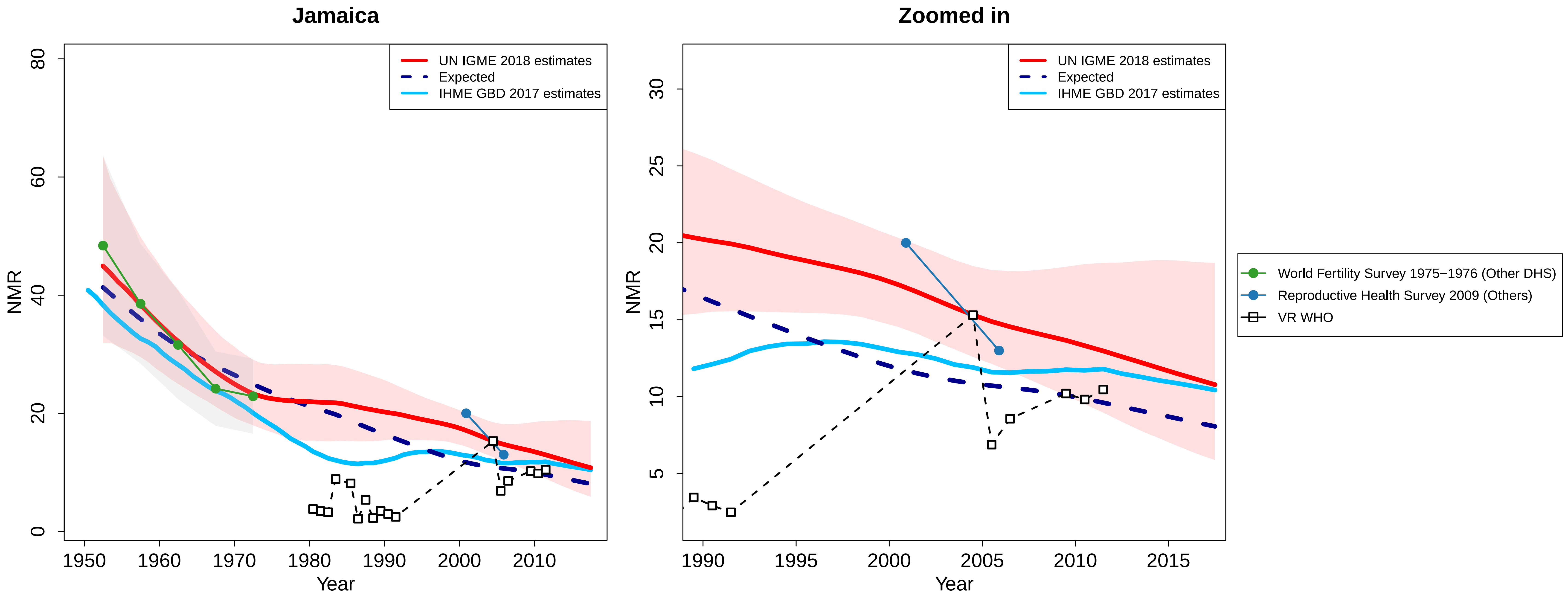
Jamaica
Zoomed in
UN IGME 2018 estimates
Expected
IHME GBD 2017 estimates
World Fertility Survey 1975−1976 (Other DHS)
Reproductive Health Survey 2009 (Others)
VR WHO
NMR
Year
0
20
40
60
80
1950
1960
1970
1980
1990
2000
2010
5
10
15
20
25
30
1995
2005
2015

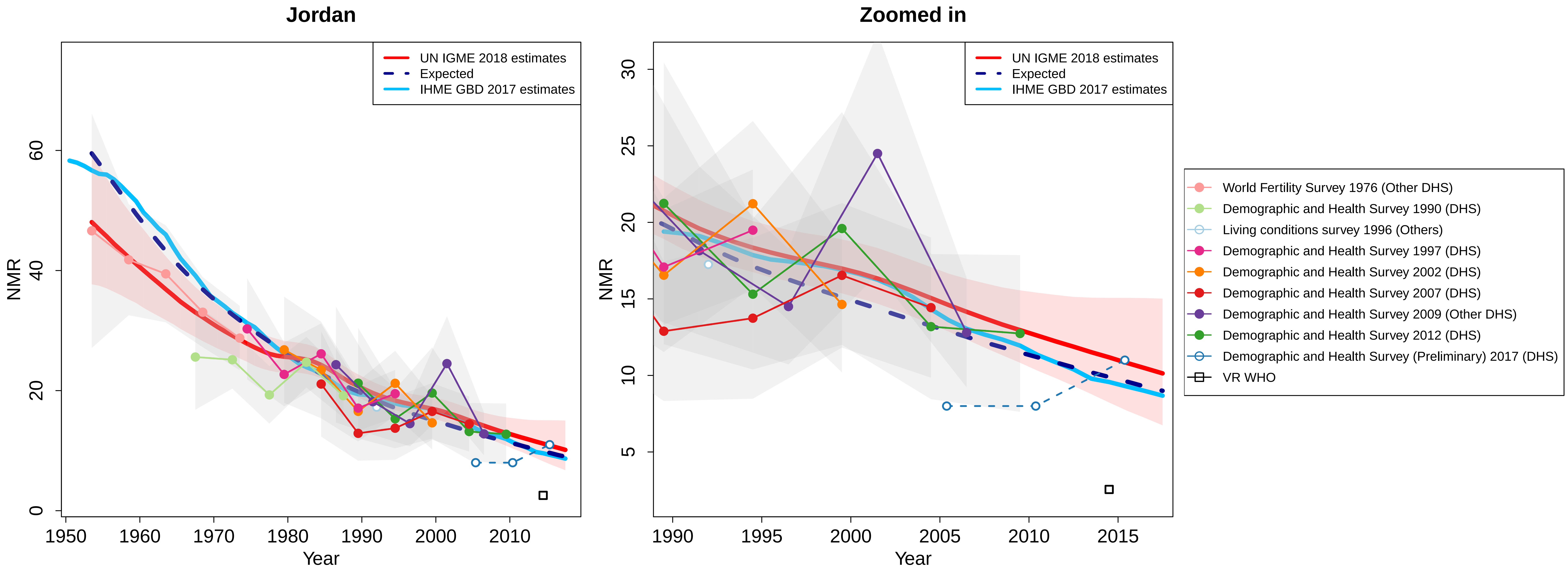
Jordan
Zoomed in
UN IGME 2018 estimates
Expected
IHME GBD 2017 estimates
NMR
Year
0
20
40
60
1950
1960
1970
1980
1990
2000
2010
5
10
15
20
25
30
1995
2005
2015
World Fertility Survey 1976 (Other DHS)
Demographic and Health Survey 1990 (DHS)
Living conditions survey 1996 (Others)
Demographic and Health Survey 1997 (DHS)
Demographic and Health Survey 2002 (DHS)
Demographic and Health Survey 2007 (DHS)
Demographic and Health Survey 2009 (Other DHS)
Demographic and Health Survey 2012 (DHS)
Demographic and Health Survey (Preliminary) 2017 (DHS)
VR WHO

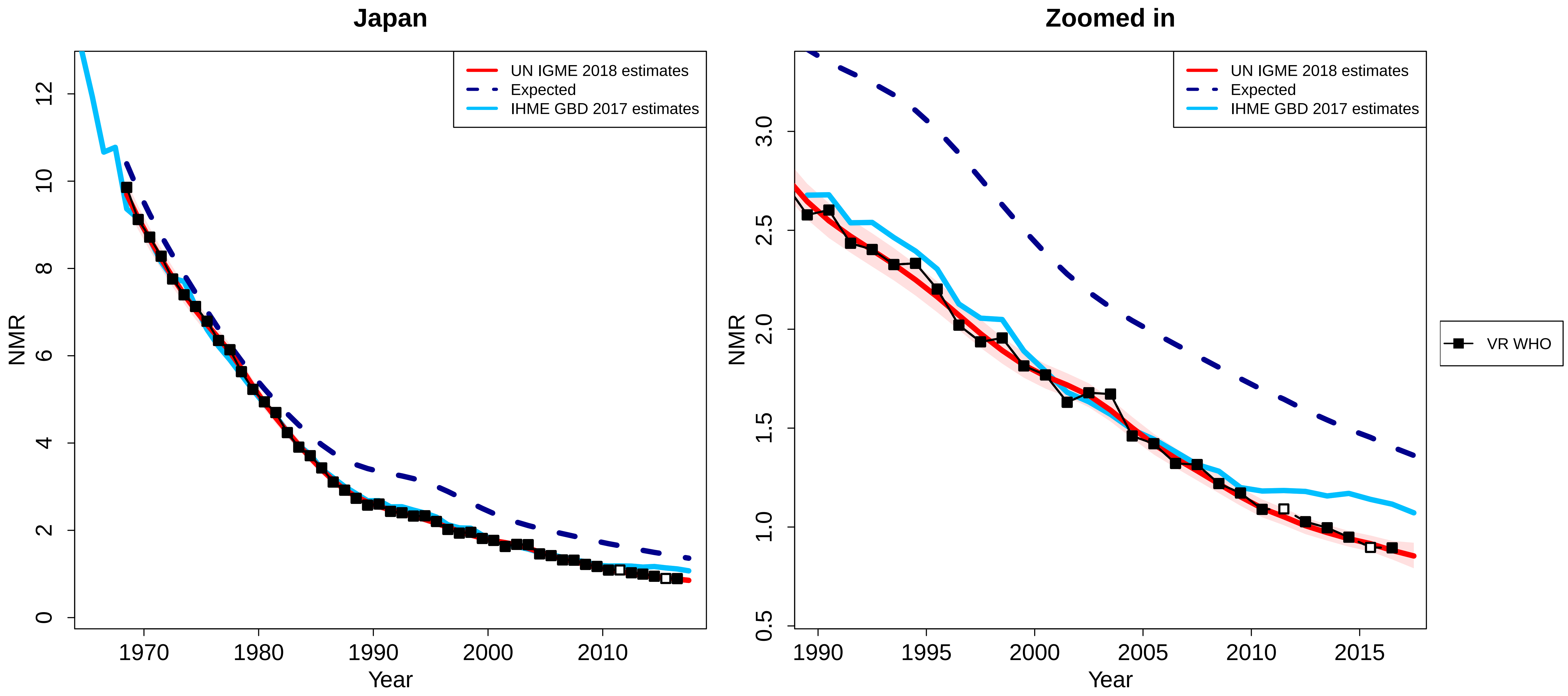

Japan
Zoomed in
UN IGME 2018 estimates
Expected
IHME GBD 2017 estimates
VR WHO
NMR
Year

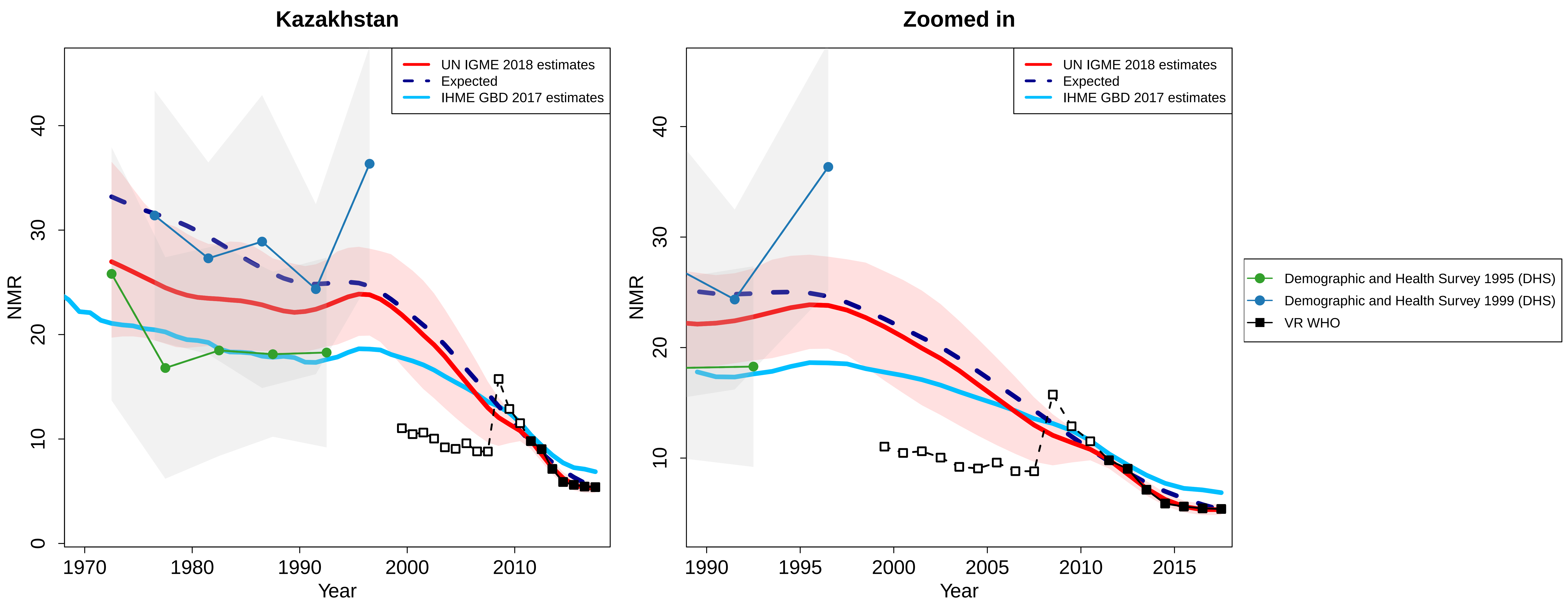

Kazakhstan
Zoomed in
UN IGME 2018 estimates
Expected
IHME GBD 2017 estimates
Demographic and Health Survey 1995 (DHS)
Demographic and Health Survey 1999 (DHS)
VR WHO
NMR
Year
0
10
20
30
40
1970
1980
1990
2000
2010
1990
1995
2000
2005
2010
2015

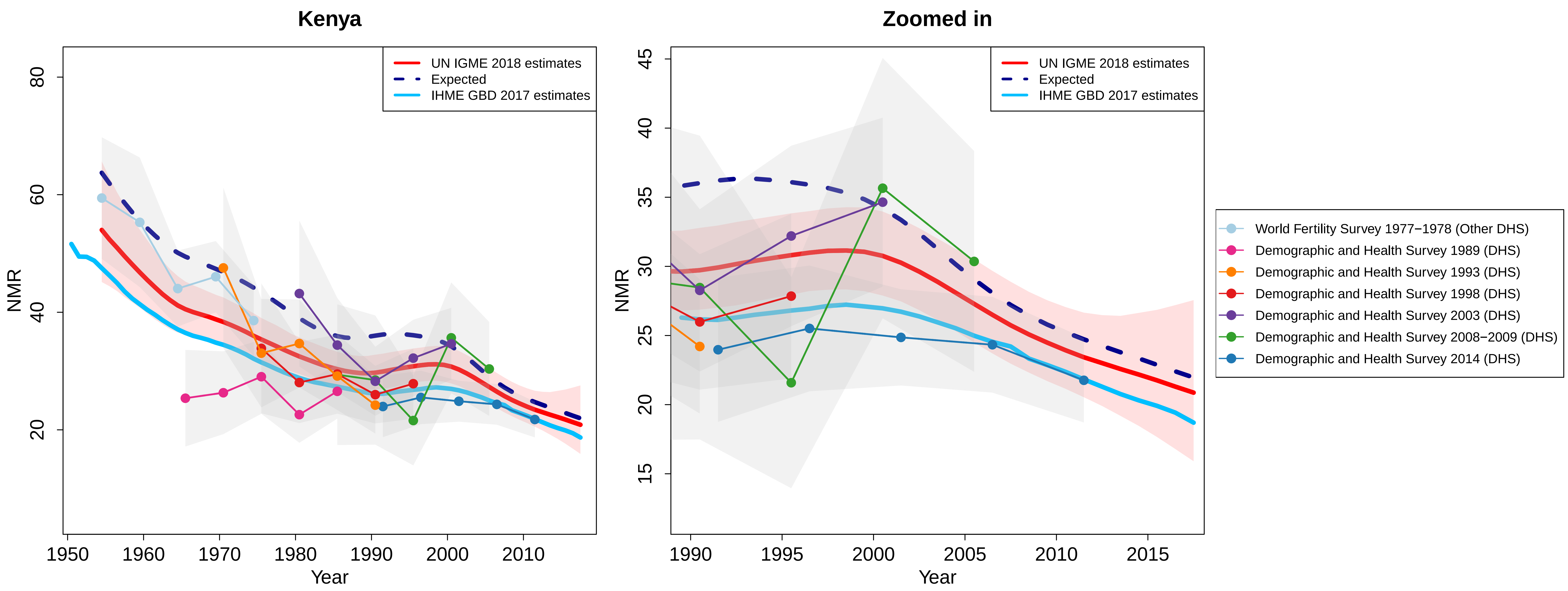
Kenya
Zoomed in
UN IGME 2018 estimates
Expected
IHME GBD 2017 estimates
NMR
Year
World Fertility Survey 1977−1978 (Other DHS)
Demographic and Health Survey 1989 (DHS)
Demographic and Health Survey 1993 (DHS)
Demographic and Health Survey 1998 (DHS)
Demographic and Health Survey 2003 (DHS)
Demographic and Health Survey 2008−2009 (DHS)
Demographic and Health Survey 2014 (DHS)

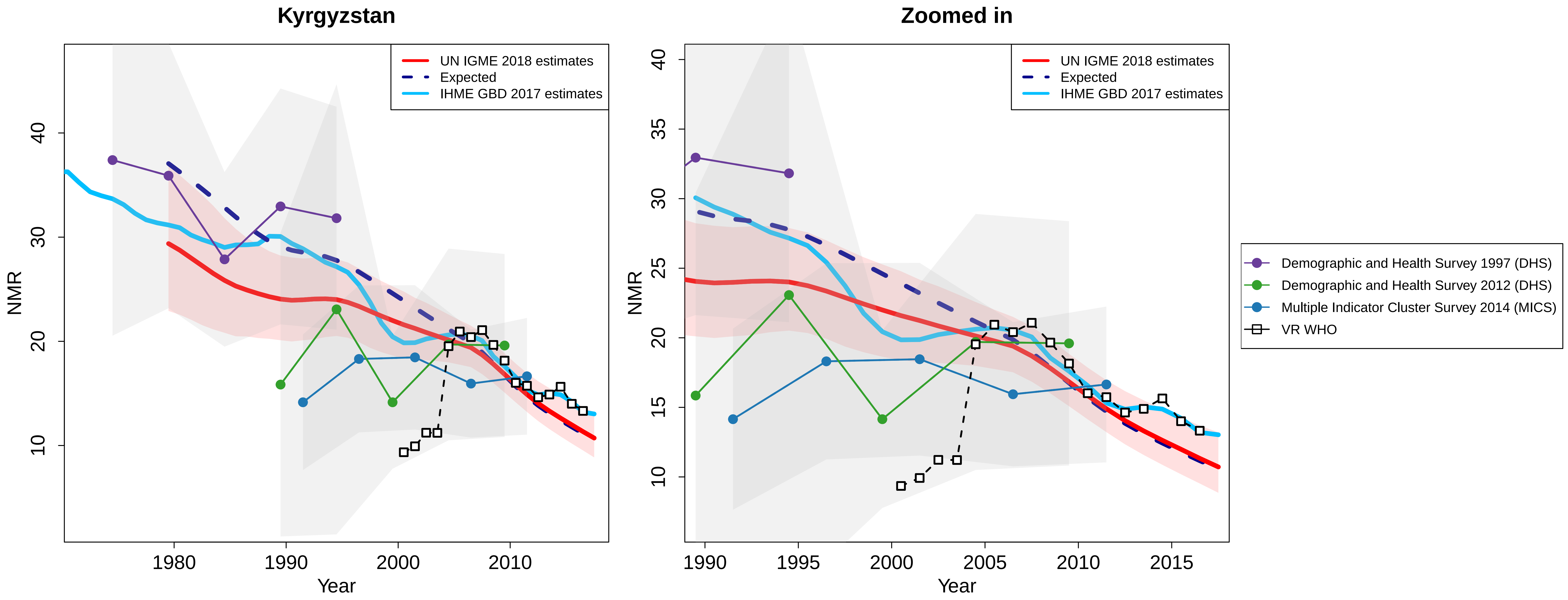
Kyrgyzstan
Zoomed in
UN IGME 2018 estimates
Expected
IHME GBD 2017 estimates
NMR
Year
Demographic and Health Survey 1997 (DHS)
Demographic and Health Survey 2012 (DHS)
Multiple Indicator Cluster Survey 2014 (MICS)
VR WHO

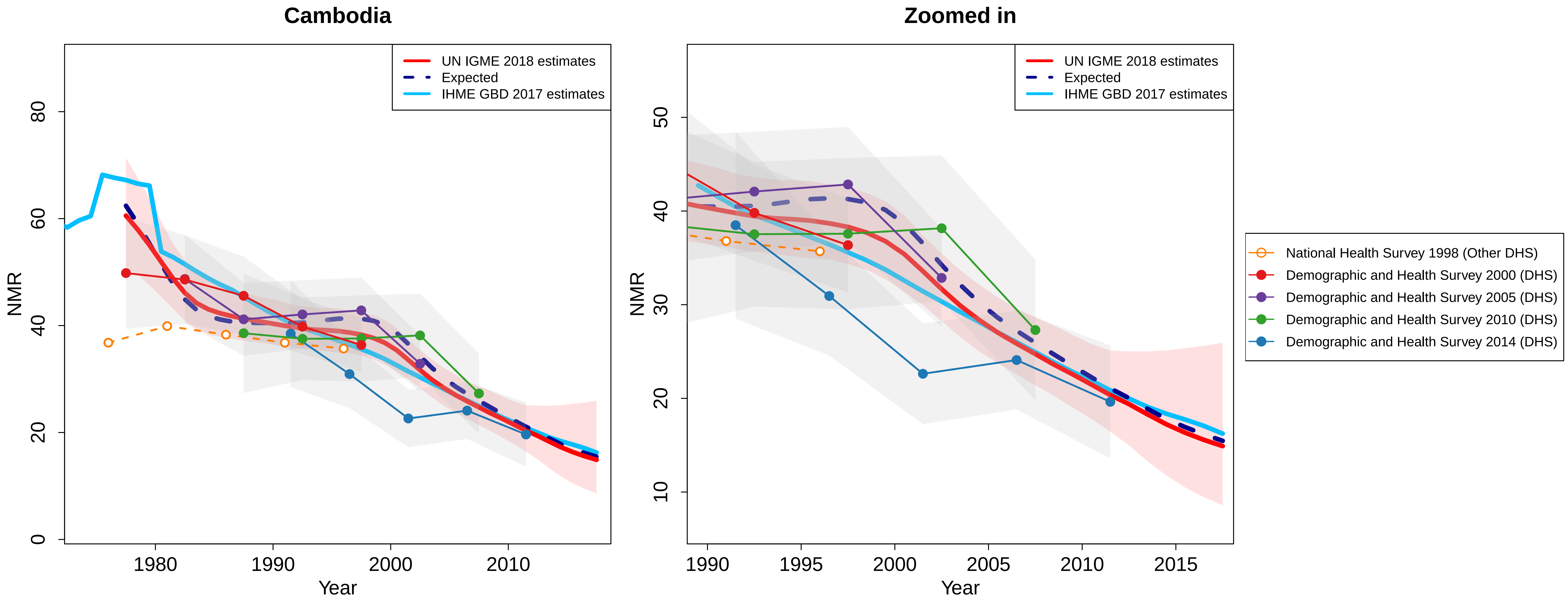

Cambodia
Zoomed in
UN IGME 2018 estimates
Expected
IHME GBD 2017 estimates
NMR
Year
National Health Survey 1998 (Other DHS)
Demographic and Health Survey 2000 (DHS)
Demographic and Health Survey 2005 (DHS)
Demographic and Health Survey 2010 (DHS)
Demographic and Health Survey 2014 (DHS)

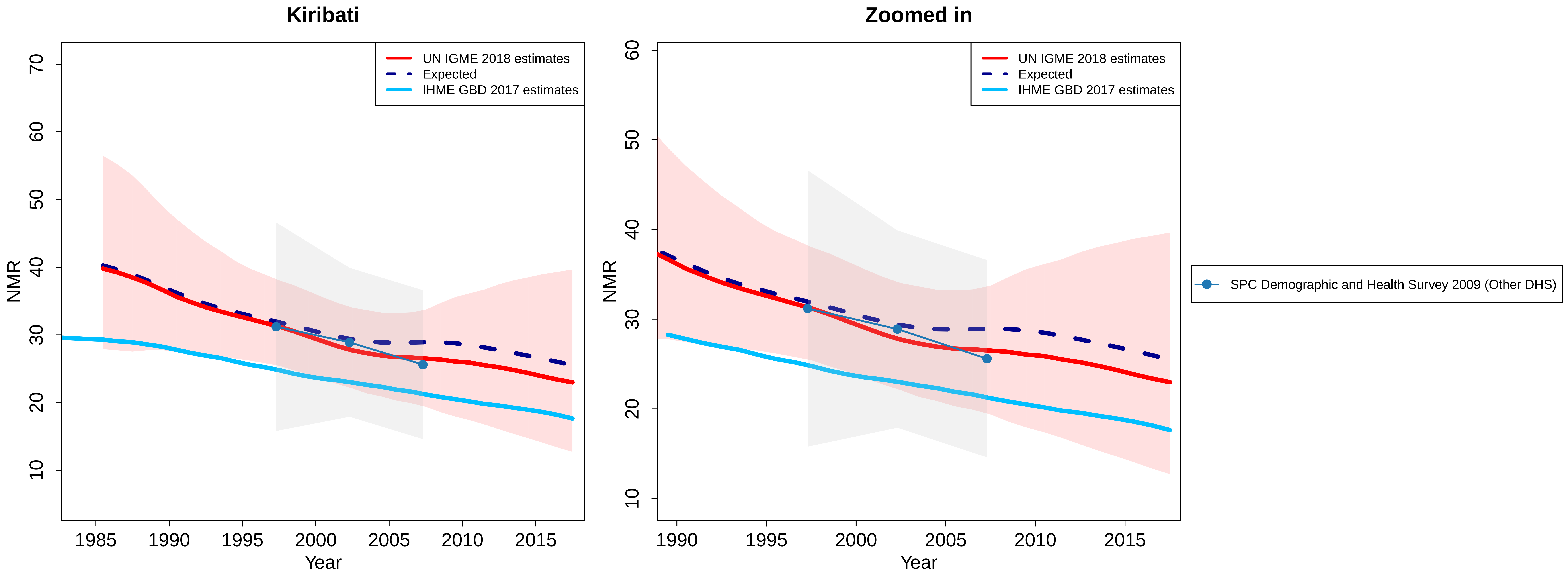
Kiribati
Zoomed in
UN IGME 2018 estimates
Expected
IHME GBD 2017 estimates
NMR
Year
10
20
30
40
50
60
70
1985
1990
1995
2000
2005
2010
2015
SPC Demographic and Health Survey 2009 (Other DHS)

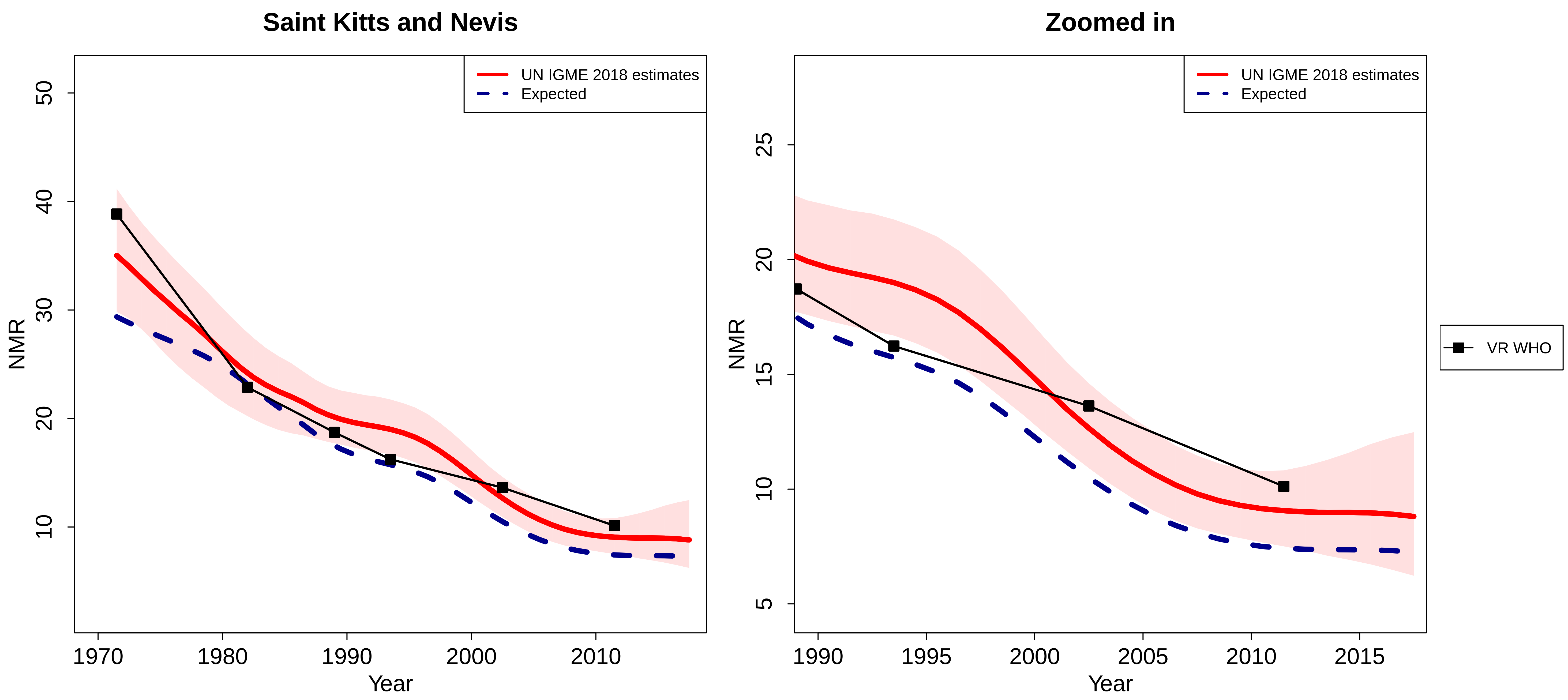

Saint Kitts and Nevis
Zoomed in
UN IGME 2018 estimates
Expected
VR WHO
NMR
Year
10
20
30
40
50
1970
1980
1990
2000
2010
5
15
25
1995
2005
2015

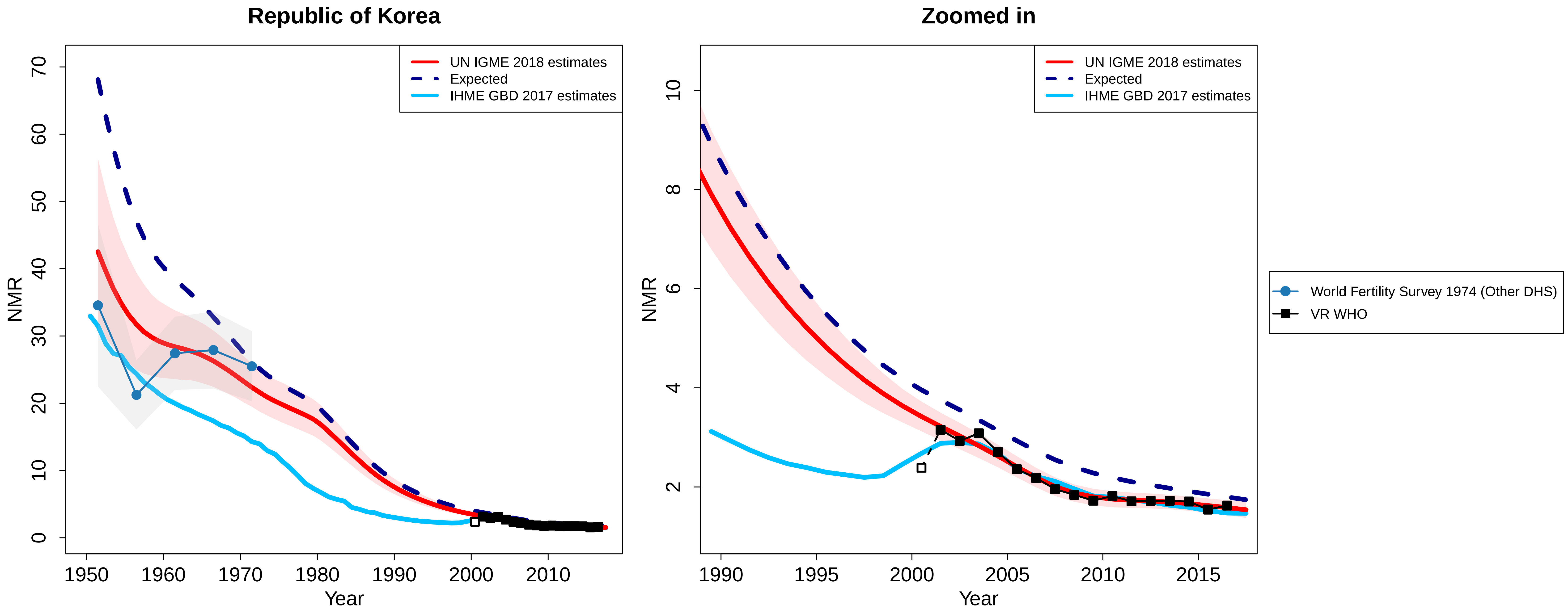
Republic of Korea
Zoomed in
UN IGME 2018 estimates
Expected
IHME GBD 2017 estimates
World Fertility Survey 1974 (Other DHS)
VR WHO
NMR
Year
0
10
20
30
40
50
60
70
1950
1960
1970
1980
1990
2000
2010
2
4
6
8
10
1995
2005
2015

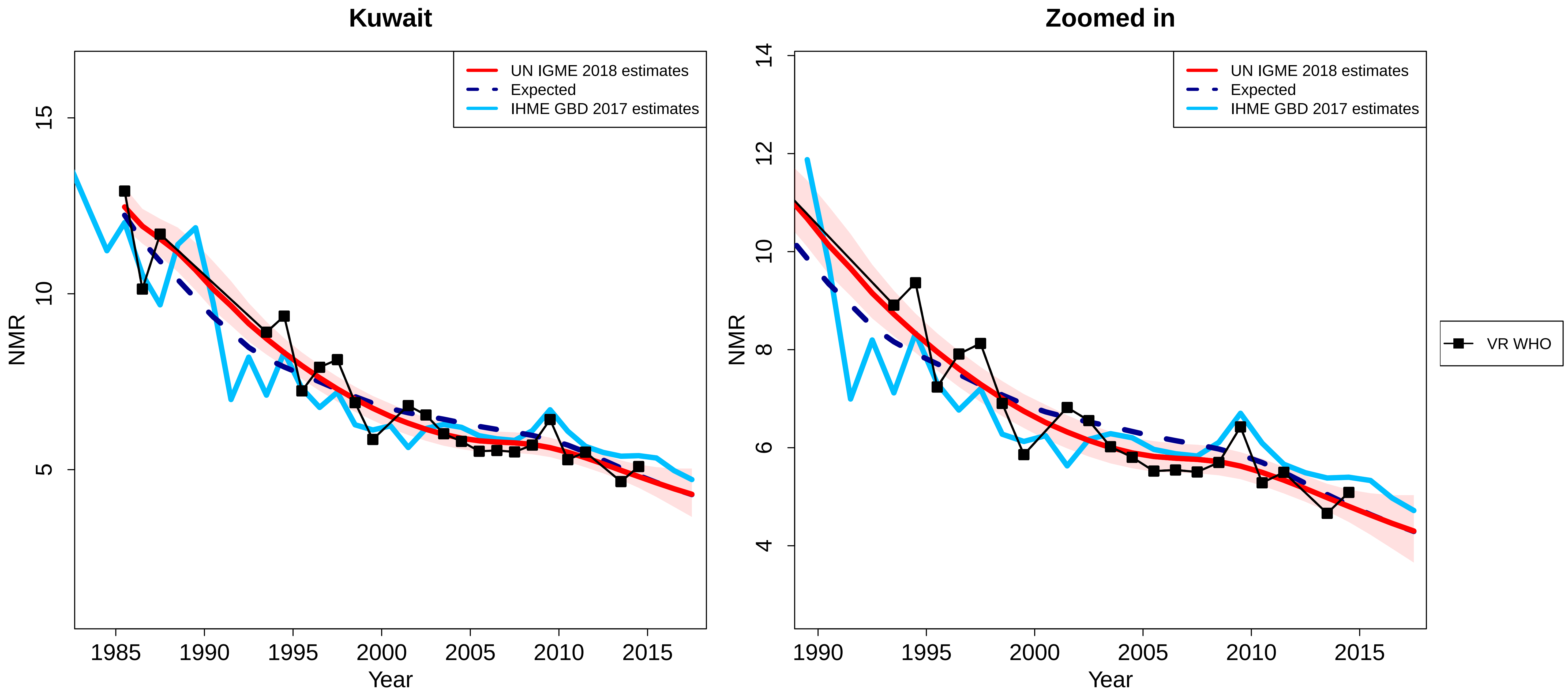
Kuwait
UN IGME 2018 estimates
Expected
IHME GBD 2017 estimates
NMR
Year
5
10
15
1985
1990
1995
2000
2005
2010
2015
Zoomed in
4
6
8
10
12
14
VR WHO

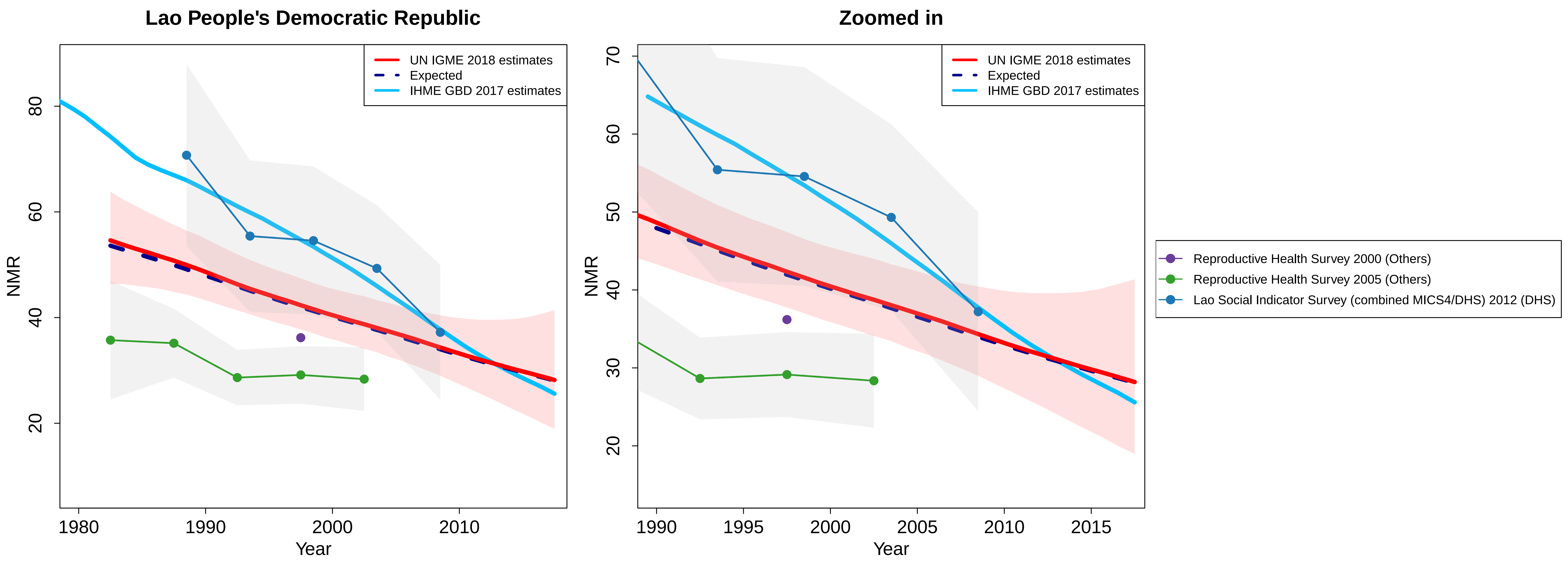
Lao People's Democratic Republic
Zoomed in
UN IGME 2018 estimates
Expected
IHME GBD 2017 estimates
Reproductive Health Survey 2000 (Others)
Reproductive Health Survey 2005 (Others)
Lao Social Indicator Survey (combined MICS4/DHS) 2012 (DHS)
NMR
Year
1980
1990
2000
2010
1995
2005
2015
20
30
40
50
60
70
80

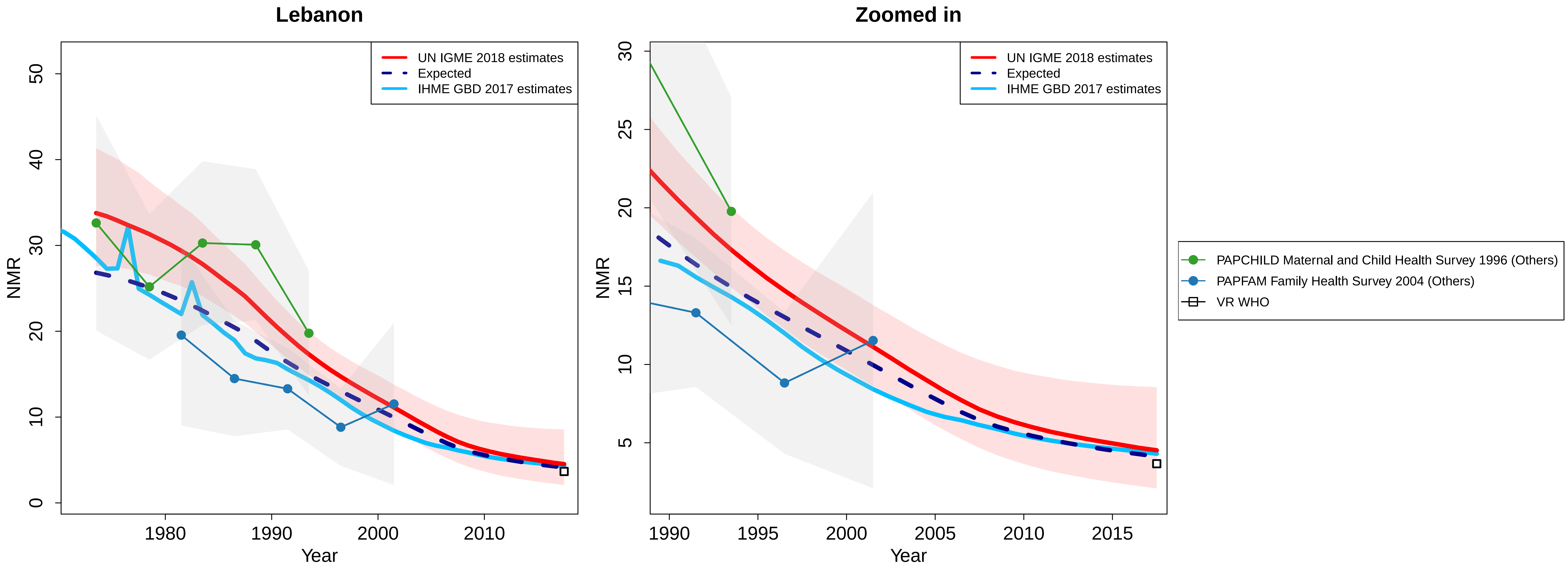

Lebanon
Zoomed in
UN IGME 2018 estimates
Expected
IHME GBD 2017 estimates
NMR
Year
PAPCHILD Maternal and Child Health Survey 1996 (Others)
PAPFAM Family Health Survey 2004 (Others)
VR WHO

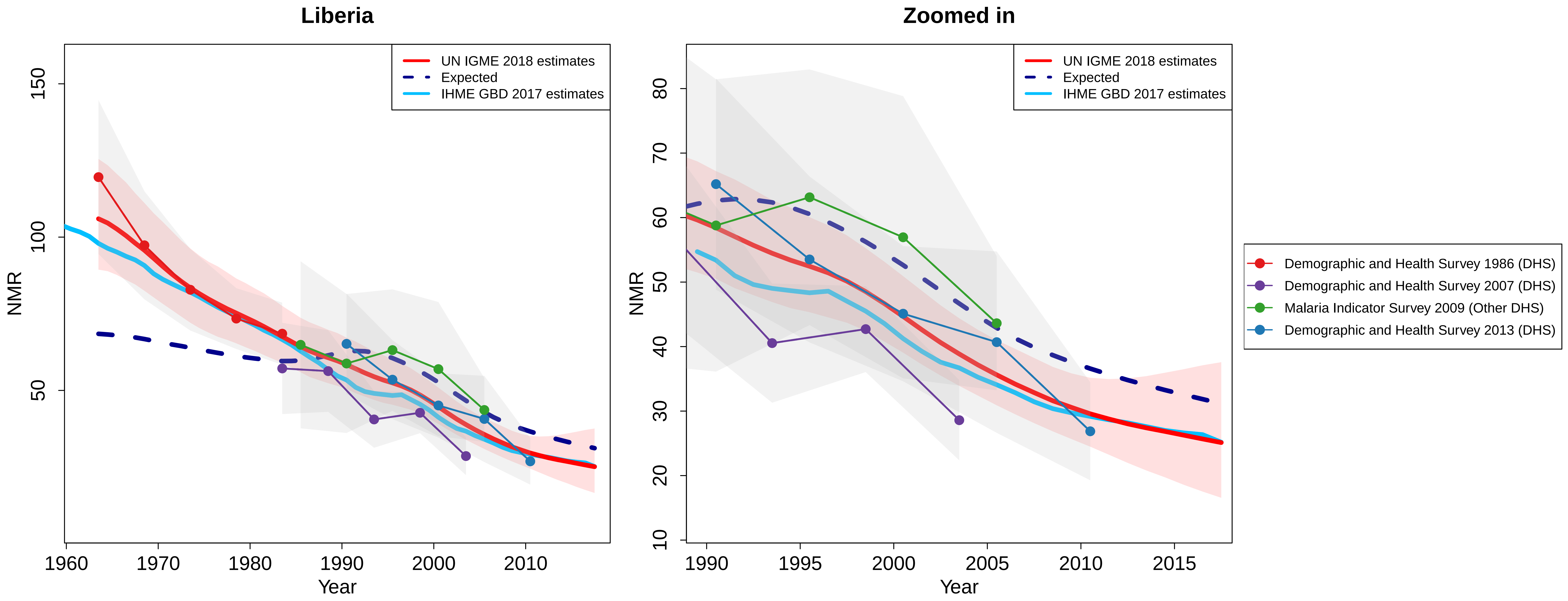
Liberia
UN IGME 2018 estimates
Expected
IHME GBD 2017 estimates
NMR
Year
Zoomed in
Demographic and Health Survey 1986 (DHS)
Demographic and Health Survey 2007 (DHS)
Malaria Indicator Survey 2009 (Other DHS)
Demographic and Health Survey 2013 (DHS)

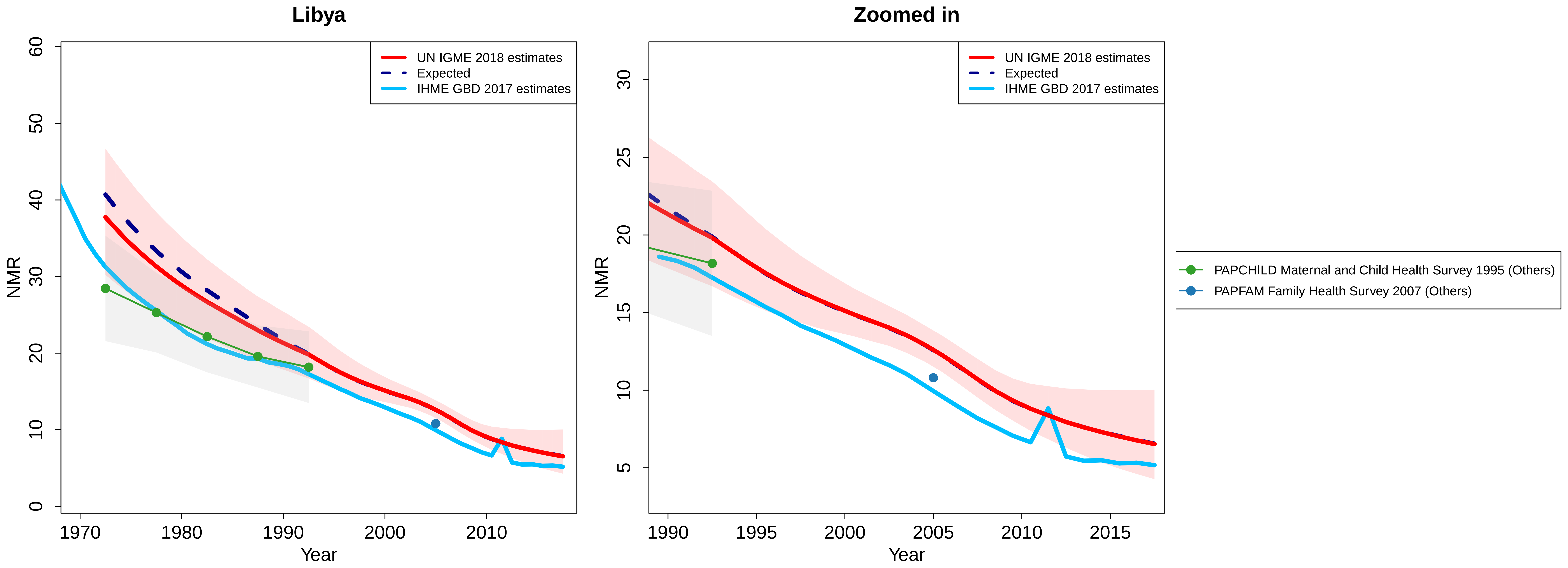

Libya
Zoomed in
UN IGME 2018 estimates
Expected
IHME GBD 2017 estimates
NMR
Year
PAPCHILD Maternal and Child Health Survey 1995 (Others)
PAPFAM Family Health Survey 2007 (Others)

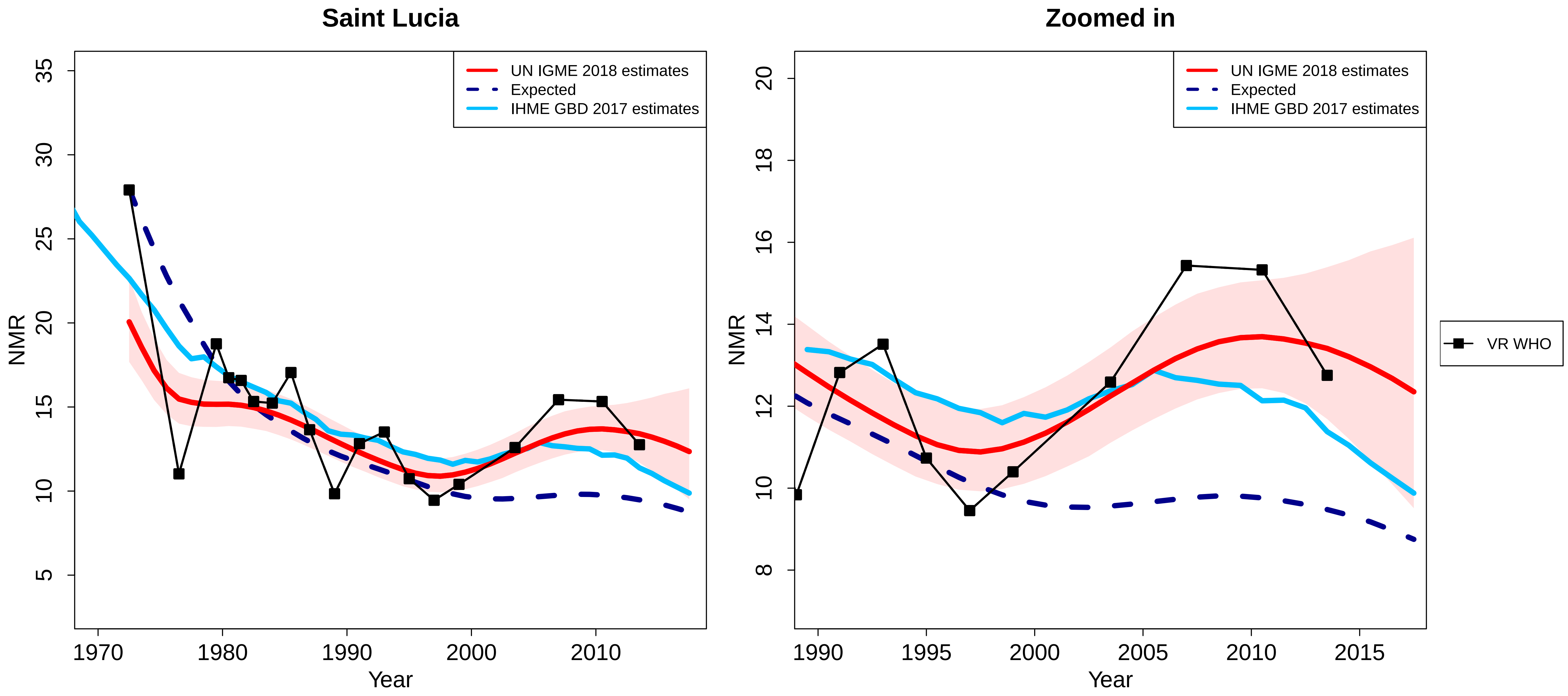
Saint Lucia
Zoomed in
UN IGME 2018 estimates
Expected
IHME GBD 2017 estimates
VR WHO
NMR
Year

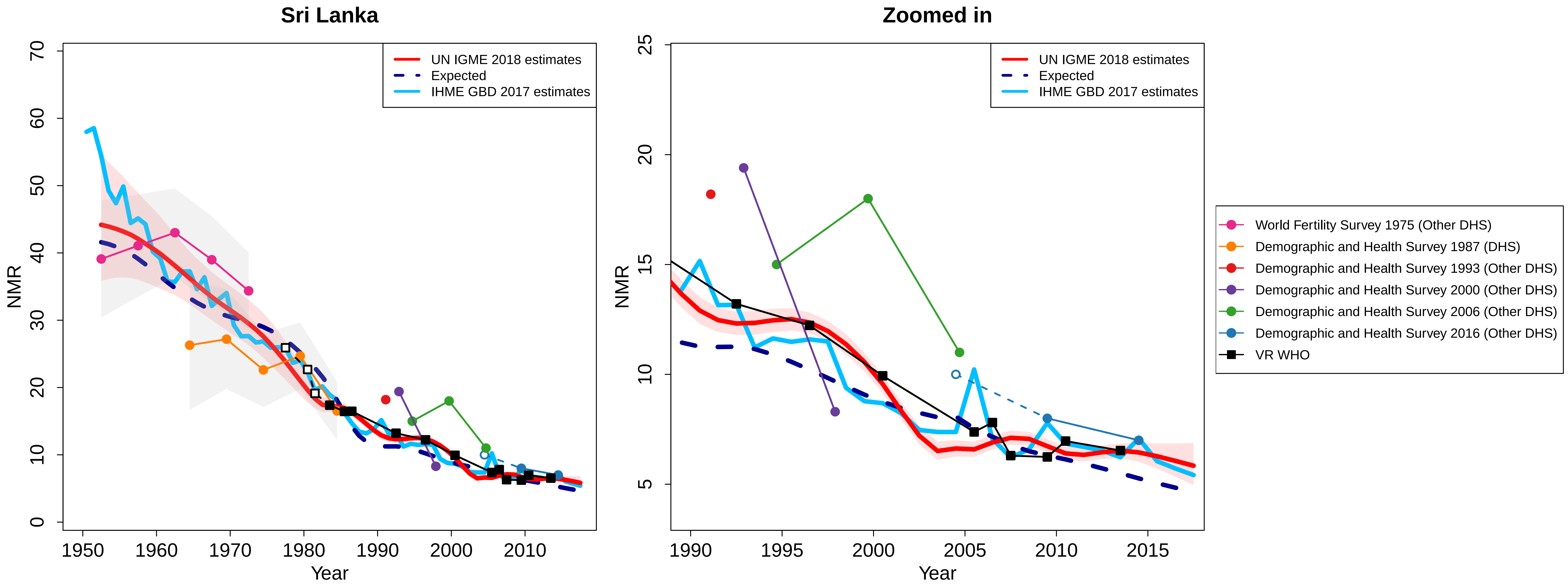

Sri Lanka
Zoomed in
UN IGME 2018 estimates
Expected
IHME GBD 2017 estimates
NMR
Year
World Fertility Survey 1975 (Other DHS)
Demographic and Health Survey 1987 (DHS)
Demographic and Health Survey 1993 (Other DHS)
Demographic and Health Survey 2000 (Other DHS)
Demographic and Health Survey 2006 (Other DHS)
Demographic and Health Survey 2016 (Other DHS)
VR WHO

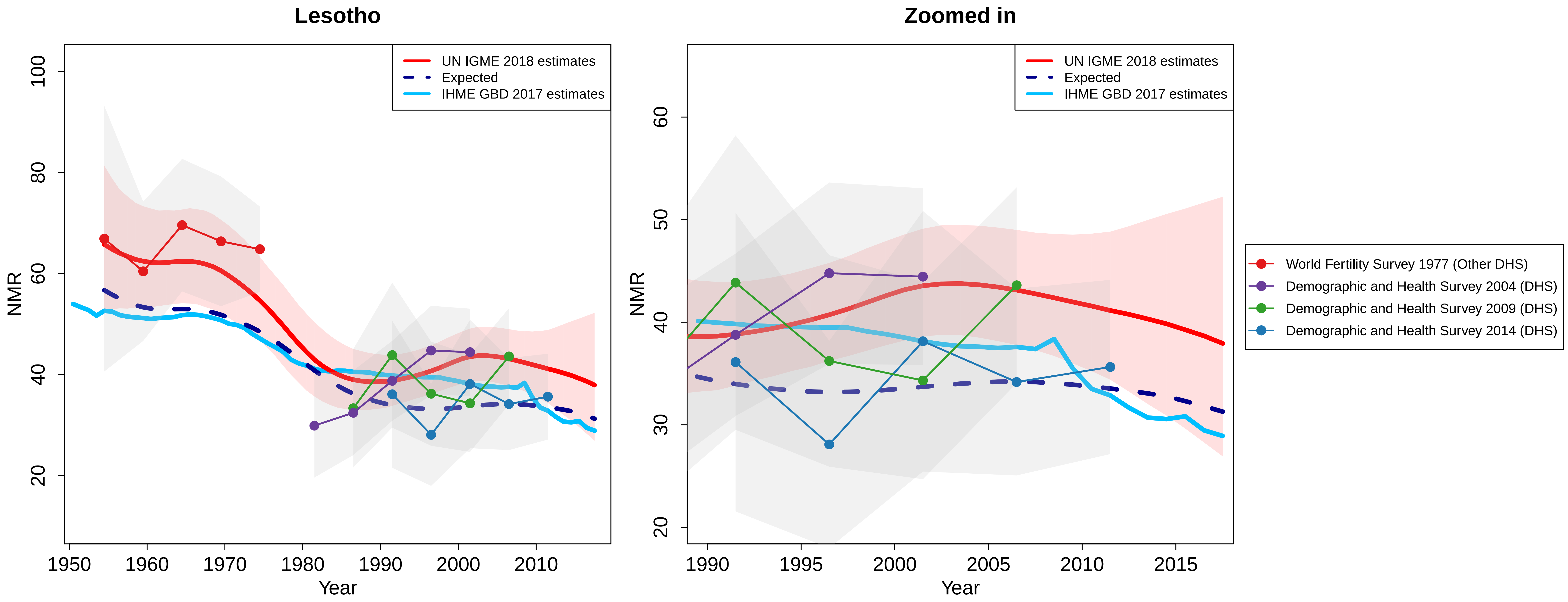
Lesotho
Zoomed in
UN IGME 2018 estimates
Expected
IHME GBD 2017 estimates
UN IGME 2018 estimates
Expected
IHME GBD 2017 estimates
NMR
Year
NMR
Year
World Fertility Survey 1977 (Other DHS)
Demographic and Health Survey 2004 (DHS)
Demographic and Health Survey 2009 (DHS)
Demographic and Health Survey 2014 (DHS)

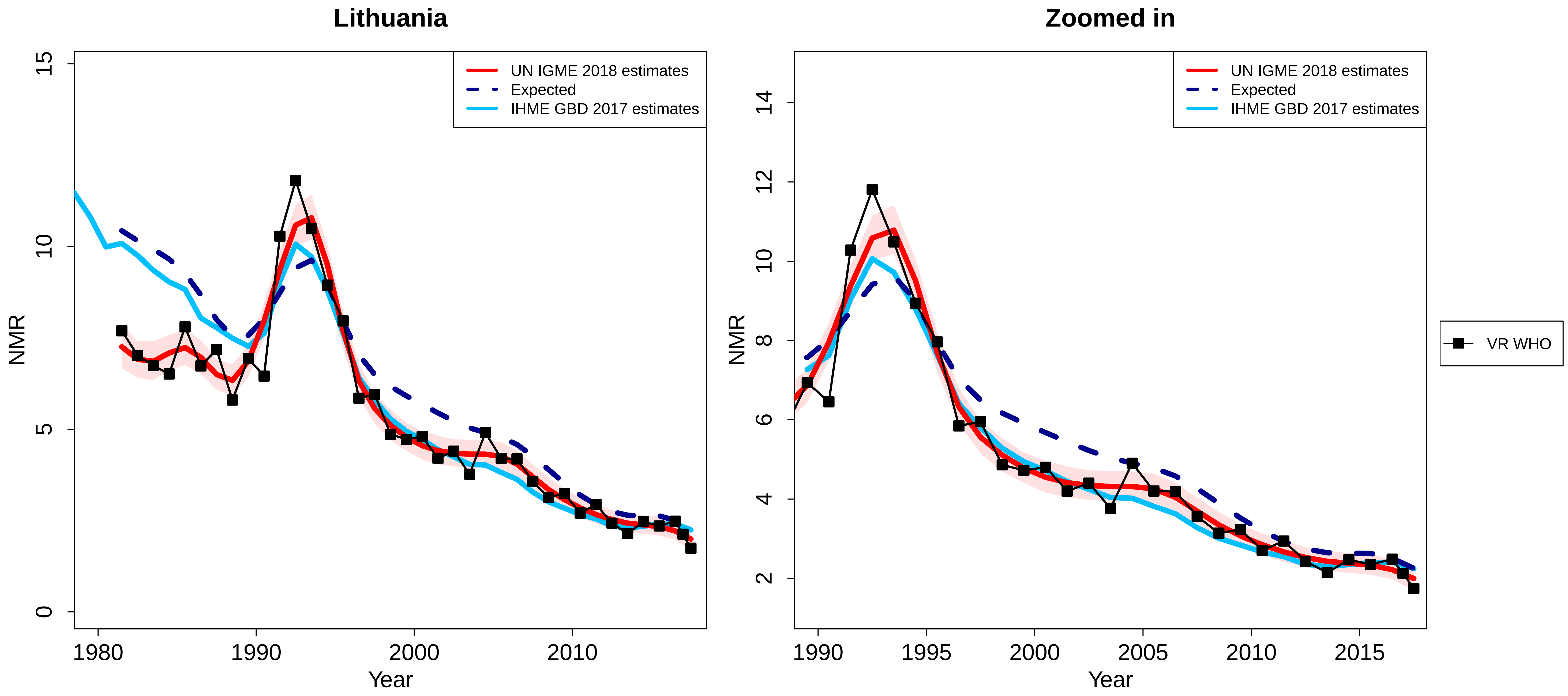
Lithuania
Zoomed in
UN IGME 2018 estimates
Expected
IHME GBD 2017 estimates
VR WHO
NMR
Year
0
5
10
15
1980
1990
2000
2010
2
4
6
8
10
12
14
1990
1995
2000
2005
2010
2015

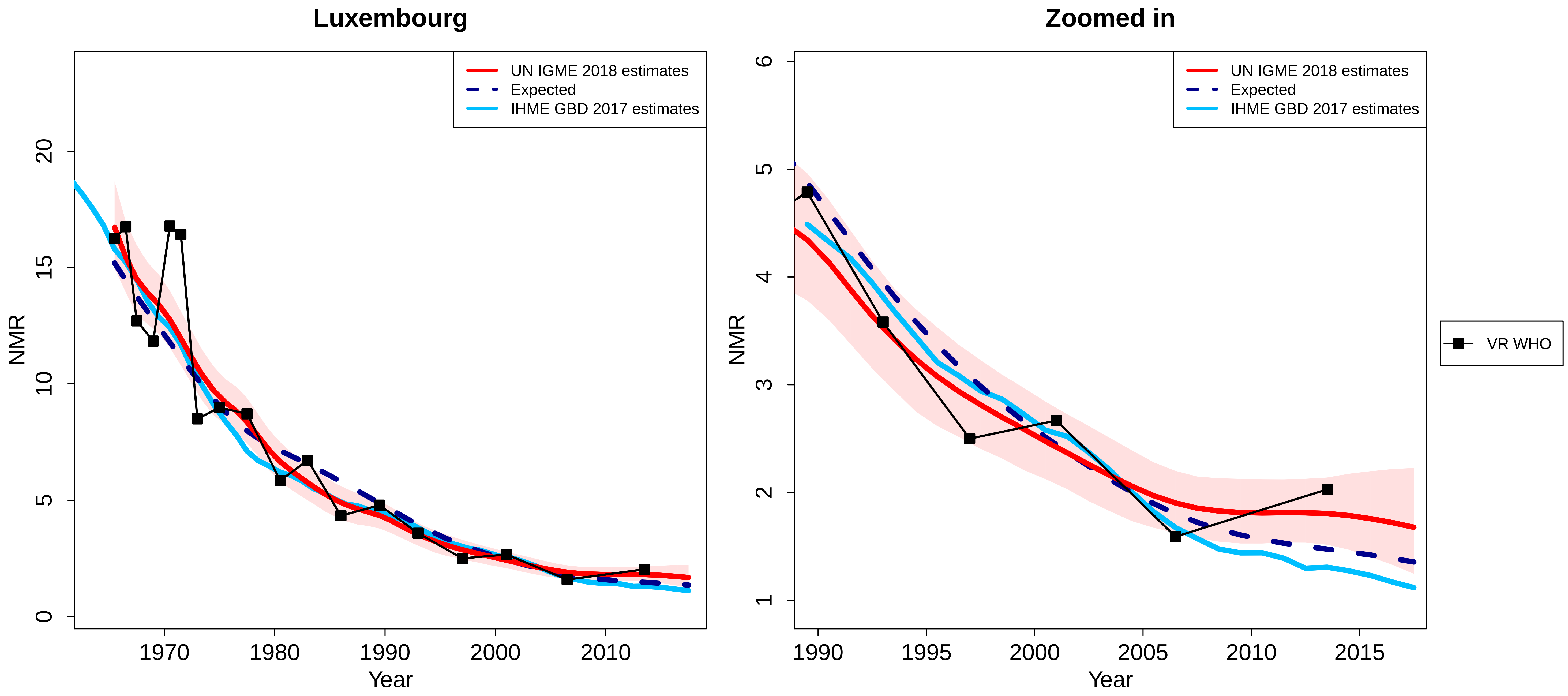
Luxembourg
Zoomed in
UN IGME 2018 estimates
Expected
IHME GBD 2017 estimates
VR WHO
NMR
Year
0
5
10
15
20
1970
1980
1990
2000
2010
1
2
3
4
5
6
1990
1995
2000
2005
2010
2015

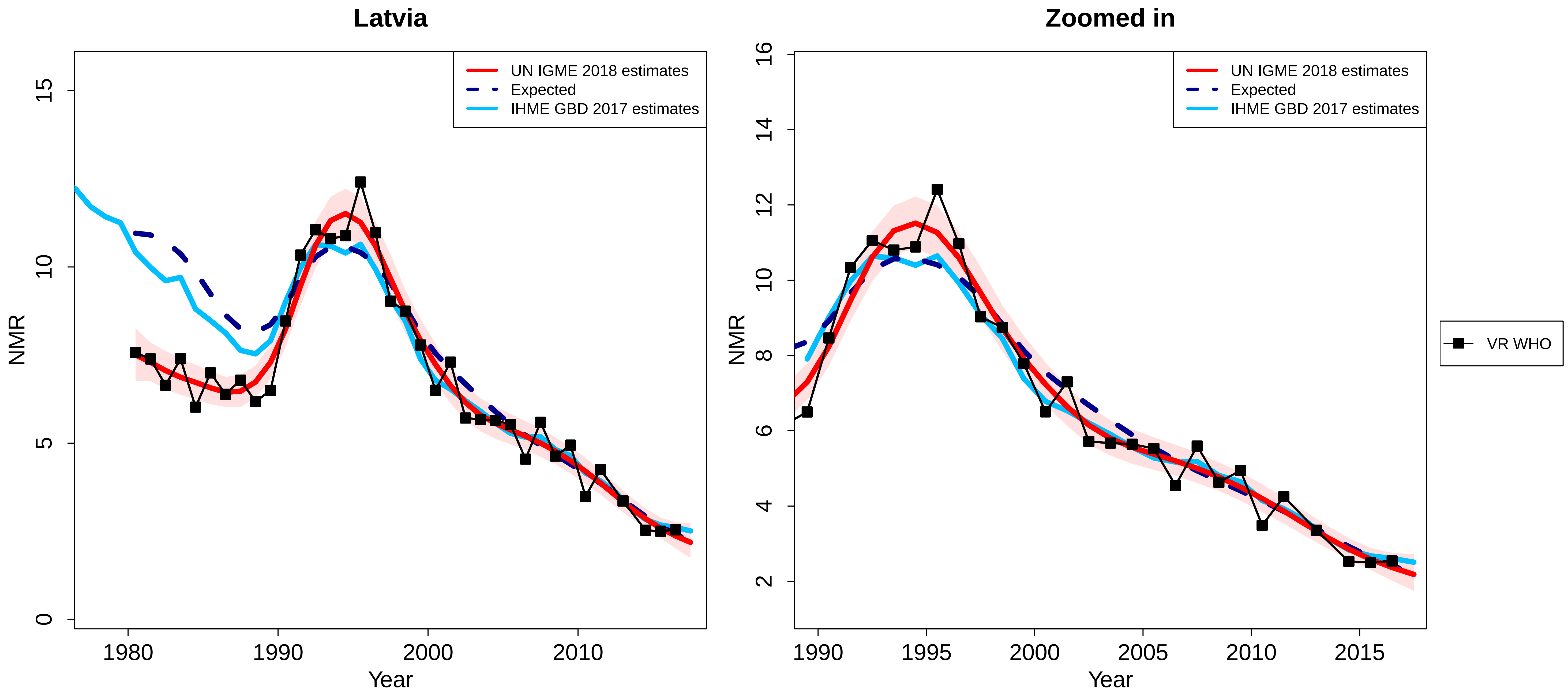
Latvia
UN IGME 2018 estimates
Expected
IHME GBD 2017 estimates
NMR
Year
0
5
10
15
1980
1990
2000
2010
Zoomed in
2
4
6
8
10
12
14
16
1990
1995
2000
2005
2010
2015
VR WHO

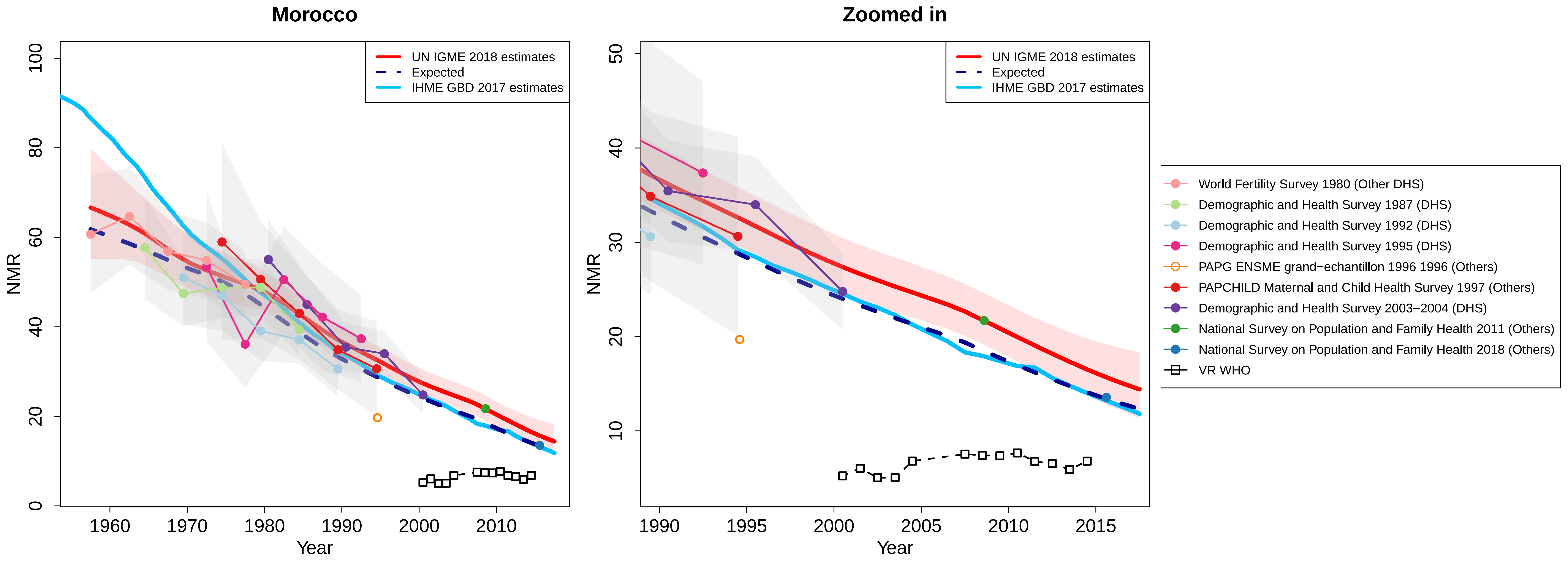

Morocco
Zoomed in
UN IGME 2018 estimates
Expected
IHME GBD 2017 estimates
NMR
Year
World Fertility Survey 1980 (Other DHS)
Demographic and Health Survey 1987 (DHS)
Demographic and Health Survey 1992 (DHS)
Demographic and Health Survey 1995 (DHS)
PAPG ENSME grand−echantillon 1996 1996 (Others)
PAPCHILD Maternal and Child Health Survey 1997 (Others)
Demographic and Health Survey 2003−2004 (DHS)
National Survey on Population and Family Health 2011 (Others)
National Survey on Population and Family Health 2018 (Others)
VR WHO

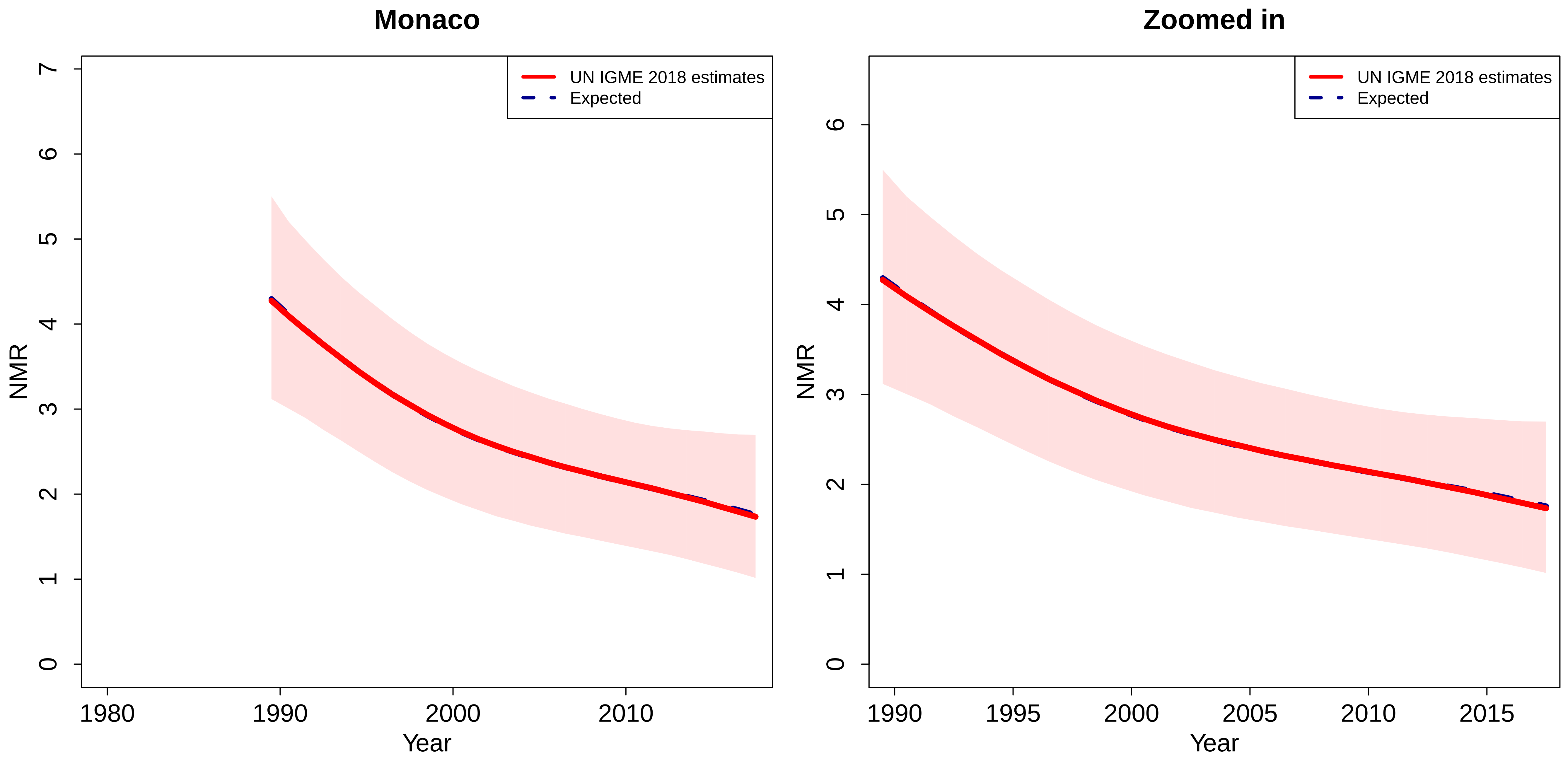
Monaco
UN IGME 2018 estimates
Expected
NMR
Year
0
1
2
3
4
5
6
7
1980
1990
2000
2010
Zoomed in
UN IGME 2018 estimates
Expected
NMR
Year
0
1
2
3
4
5
6
1990
1995
2000
2005
2010
2015

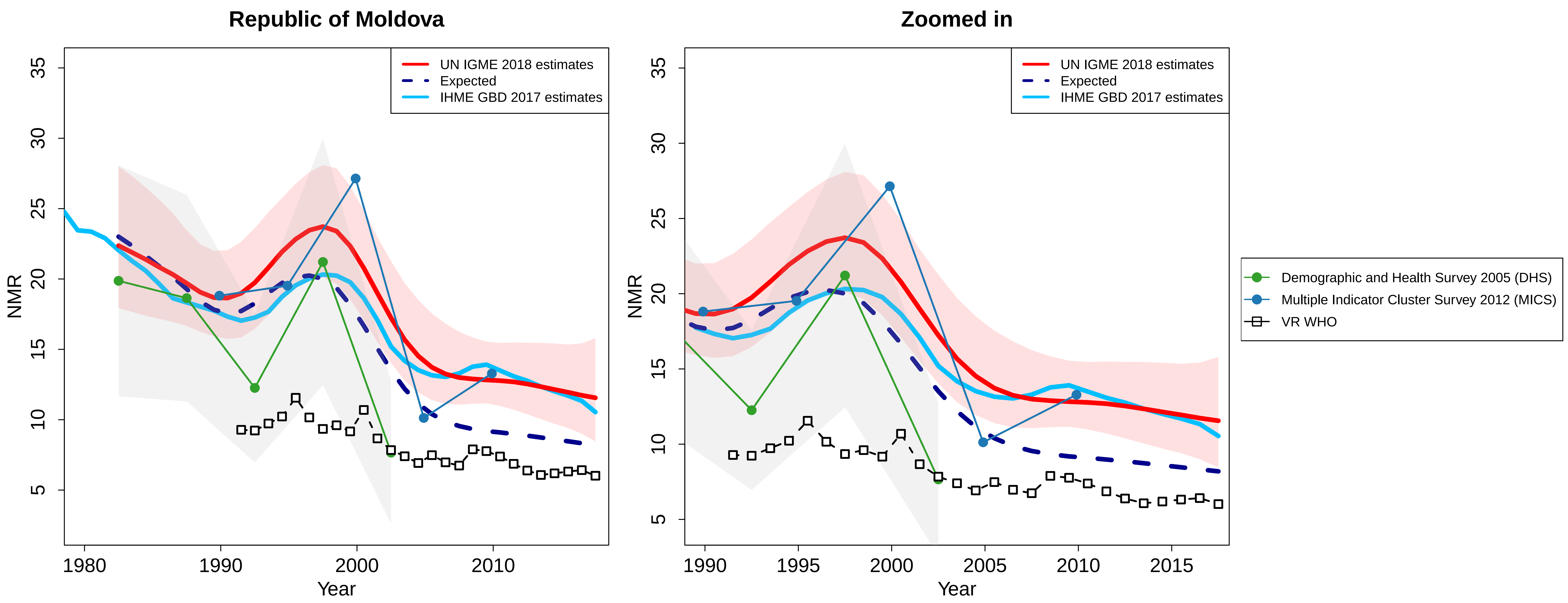

Republic of Moldova
Zoomed in
UN IGME 2018 estimates
Expected
IHME GBD 2017 estimates
NMR
Year
Demographic and Health Survey 2005 (DHS)
Multiple Indicator Cluster Survey 2012 (MICS)
VR WHO

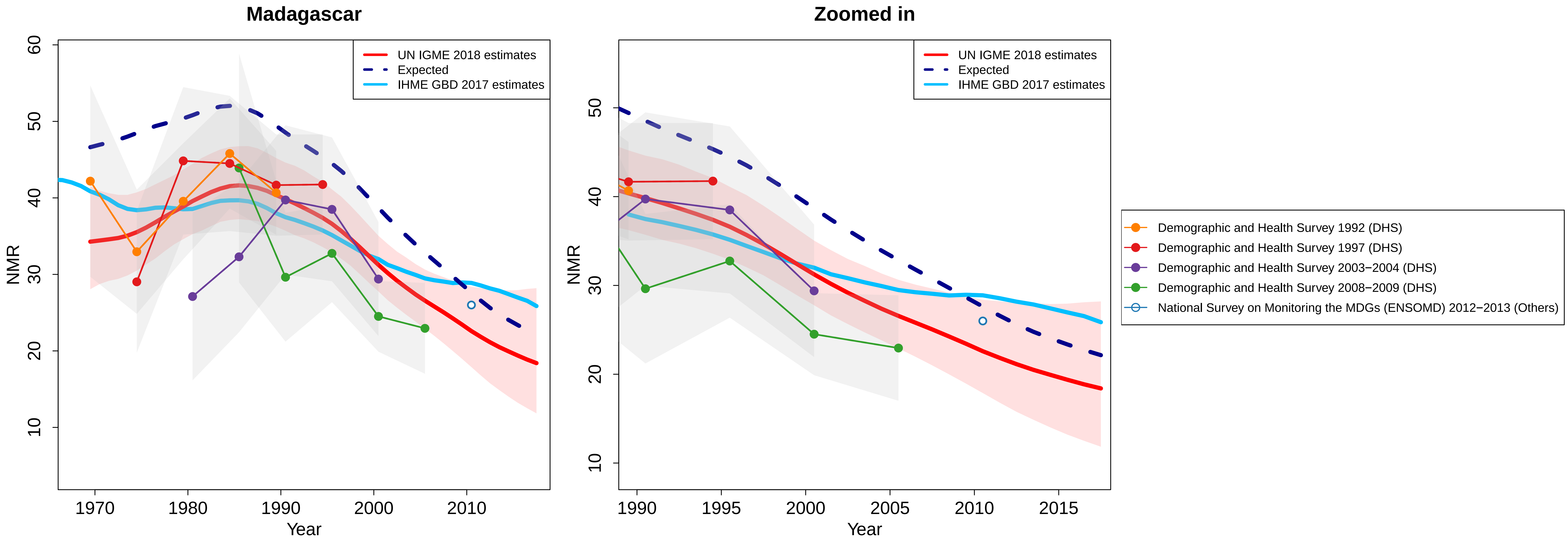
Madagascar
Zoomed in
UN IGME 2018 estimates
Expected
IHME GBD 2017 estimates
NMR
Year
Demographic and Health Survey 1992 (DHS)
Demographic and Health Survey 1997 (DHS)
Demographic and Health Survey 2003−2004 (DHS)
Demographic and Health Survey 2008−2009 (DHS)
National Survey on Monitoring the MDGs (ENSOMD) 2012−2013 (Others)

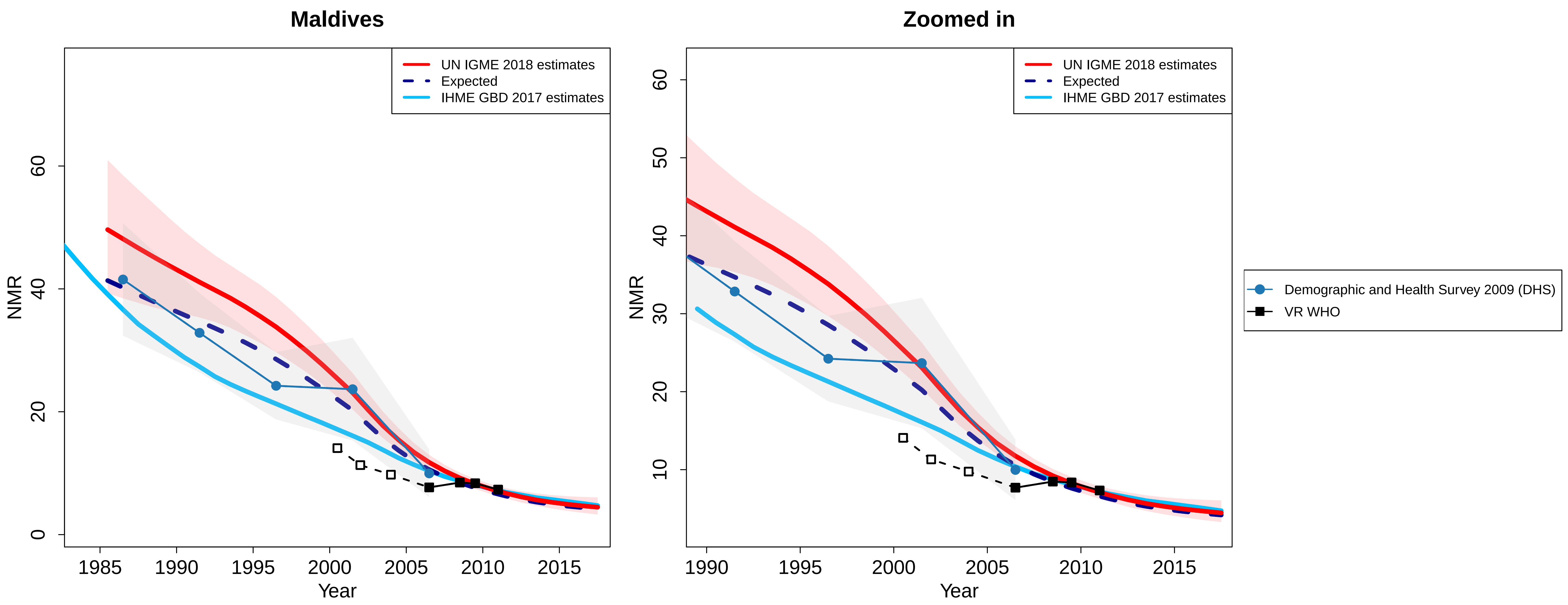
Maldives
Zoomed in
UN IGME 2018 estimates
Expected
IHME GBD 2017 estimates
Demographic and Health Survey 2009 (DHS)
VR WHO
NMR
Year
0
20
40
60
10
30
50
1985
1990
1995
2000
2005
2010
2015

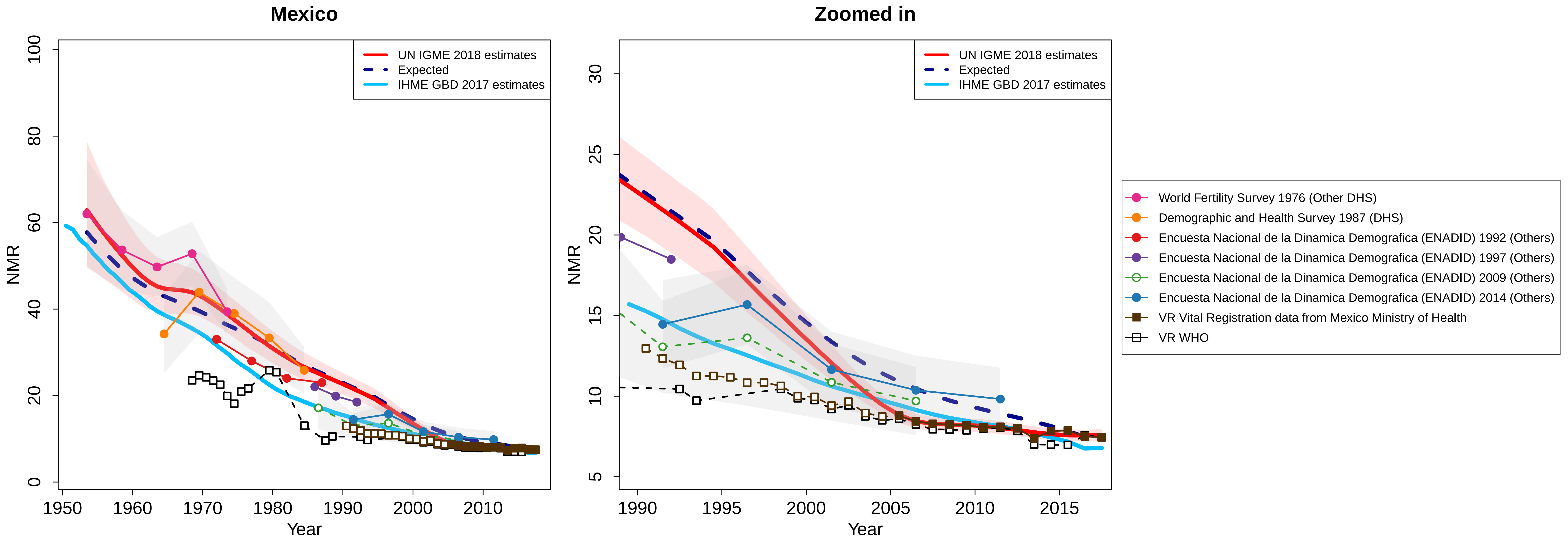
Mexico
Zoomed in
NMR
Year
UN IGME 2018 estimates
Expected
IHME GBD 2017 estimates
World Fertility Survey 1976 (Other DHS)
Demographic and Health Survey 1987 (DHS)
Encuesta Nacional de la Dinamica Demografica (ENADID) 1992 (Others)
Encuesta Nacional de la Dinamica Demografica (ENADID) 1997 (Others)
Encuesta Nacional de la Dinamica Demografica (ENADID) 2009 (Others)
Encuesta Nacional de la Dinamica Demografica (ENADID) 2014 (Others)
VR Vital Registration data from Mexico Ministry of Health
VR WHO

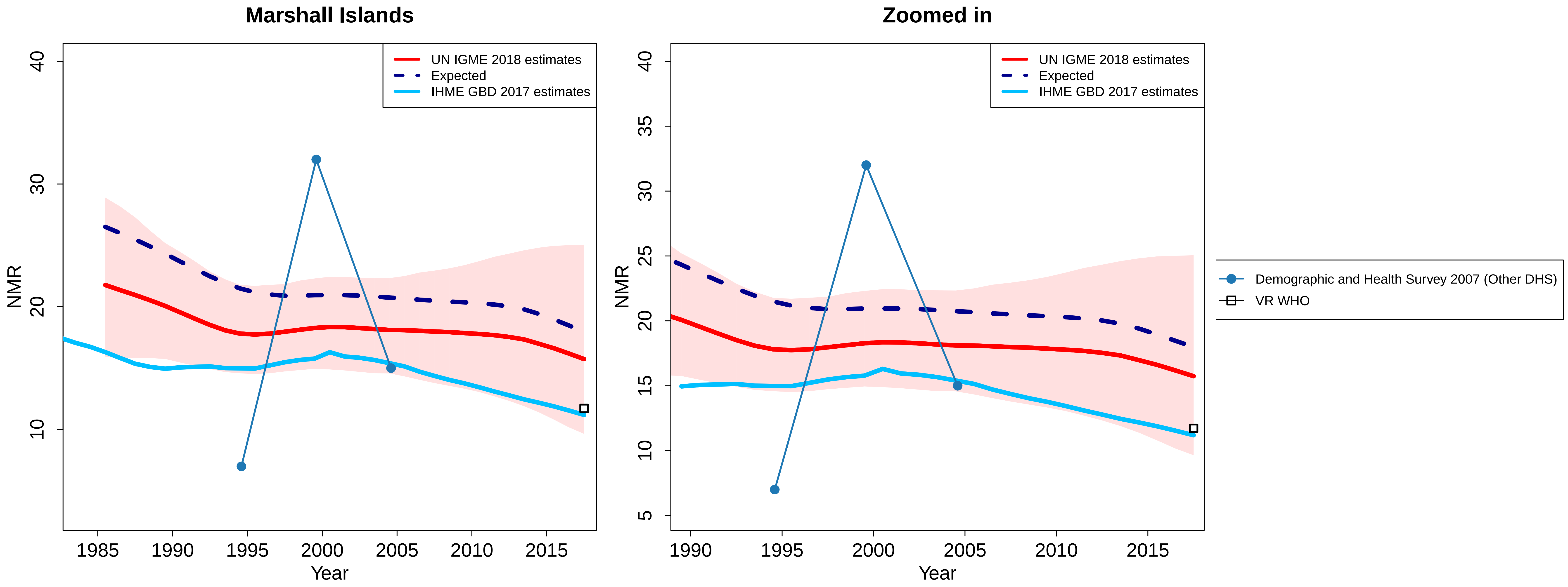

Marshall Islands
Zoomed in
UN IGME 2018 estimates
Expected
IHME GBD 2017 estimates
Demographic and Health Survey 2007 (Other DHS)
VR WHO
NMR
Year

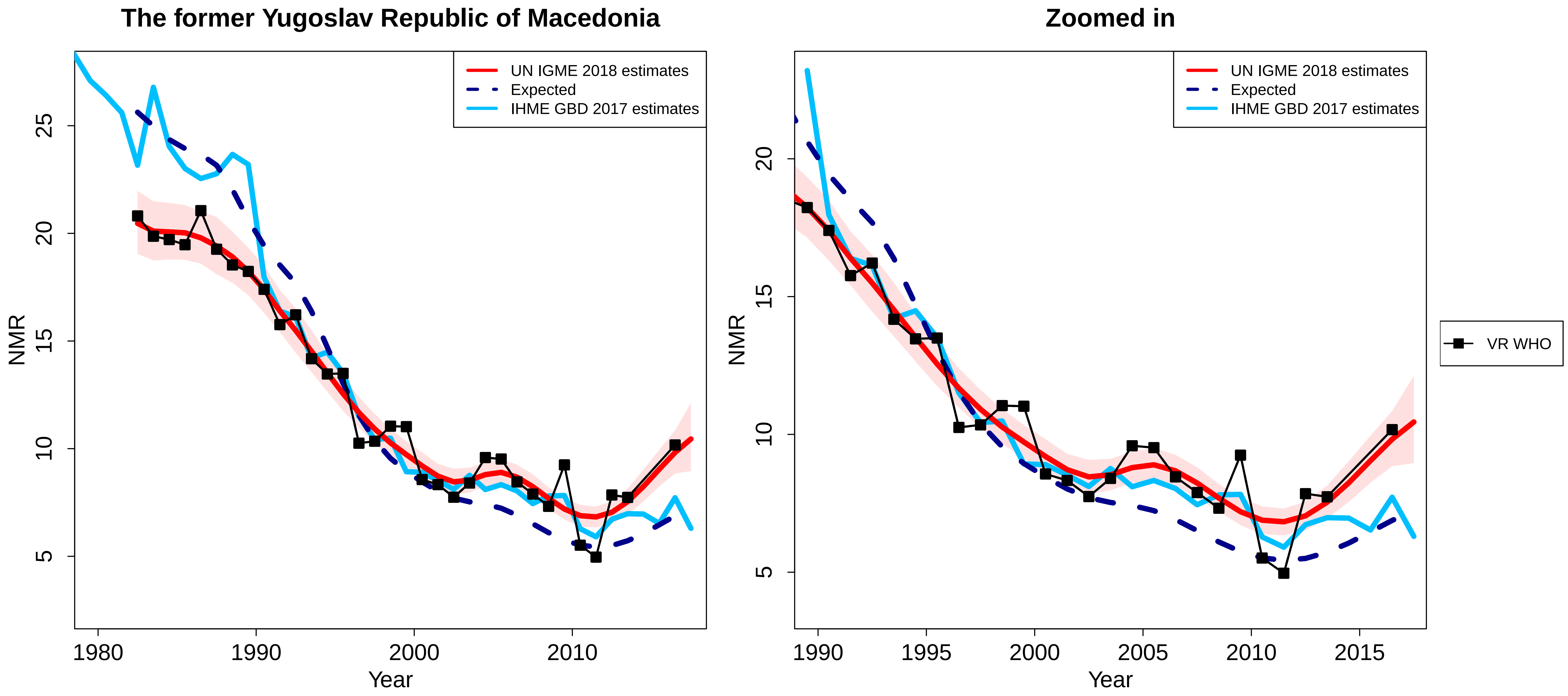
The former Yugoslav Republic of Macedonia
Zoomed in
UN IGME 2018 estimates
Expected
IHME GBD 2017 estimates
VR WHO
NMR
Year
1980
1990
2000
2010
1995
2005
2015
5
10
15
20
25

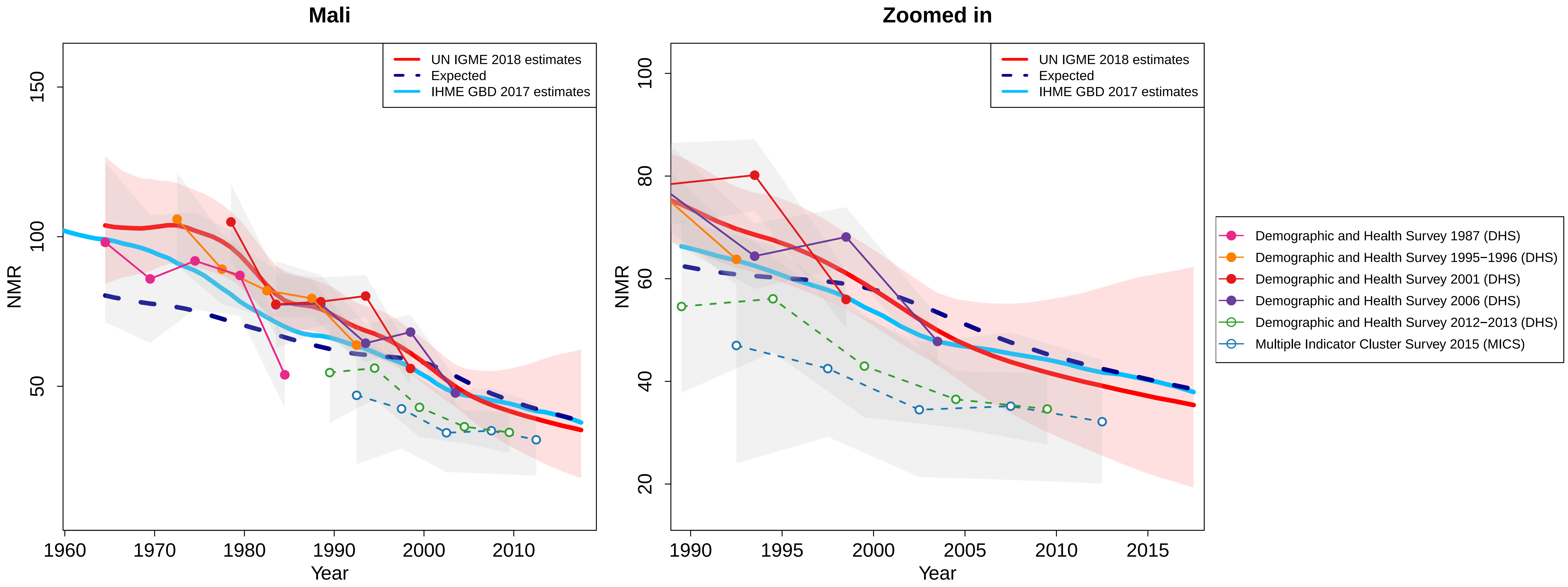
Mali
Zoomed in
UN IGME 2018 estimates
Expected
IHME GBD 2017 estimates
NMR
Year
Demographic and Health Survey 1987 (DHS)
Demographic and Health Survey 1995−1996 (DHS)
Demographic and Health Survey 2001 (DHS)
Demographic and Health Survey 2006 (DHS)
Demographic and Health Survey 2012−2013 (DHS)
Multiple Indicator Cluster Survey 2015 (MICS)

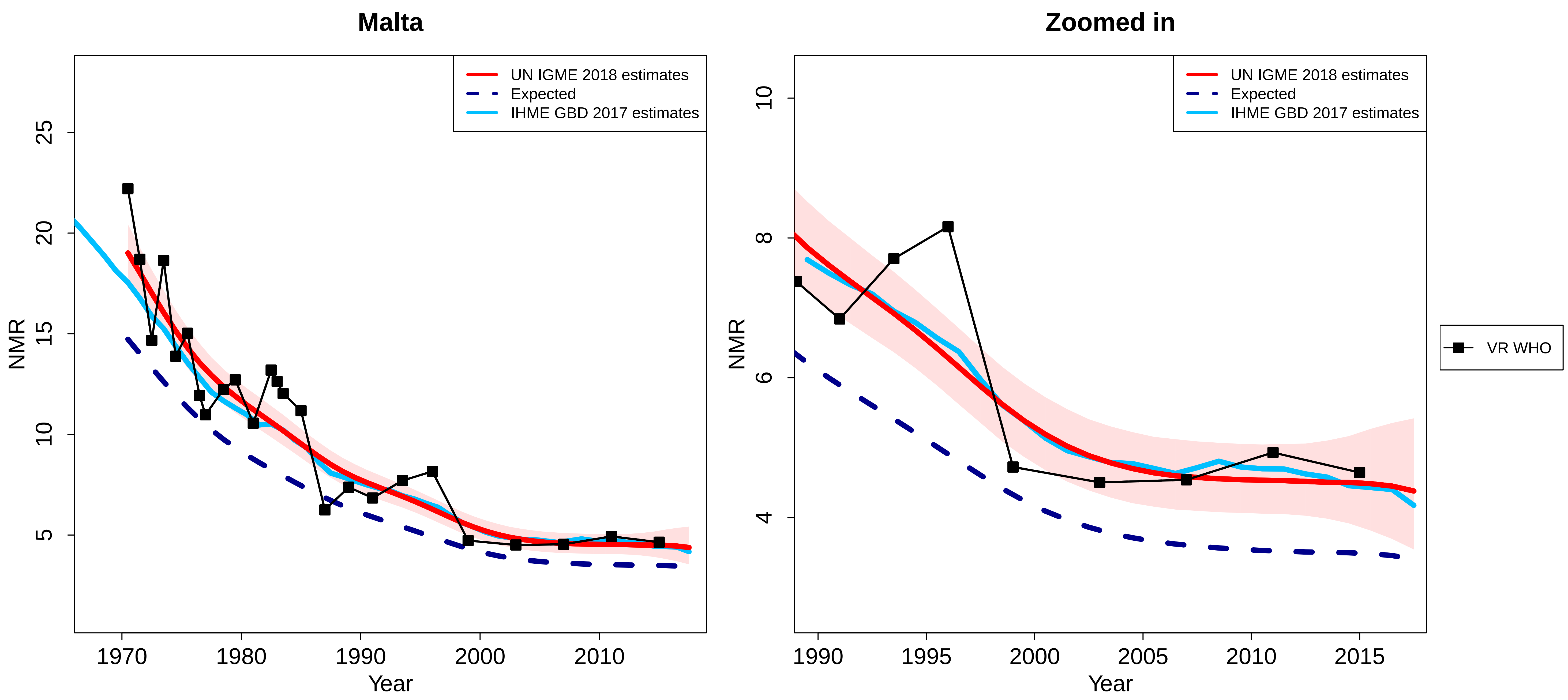

Malta
Zoomed in
UN IGME 2018 estimates
Expected
IHME GBD 2017 estimates
VR WHO
NMR
Year

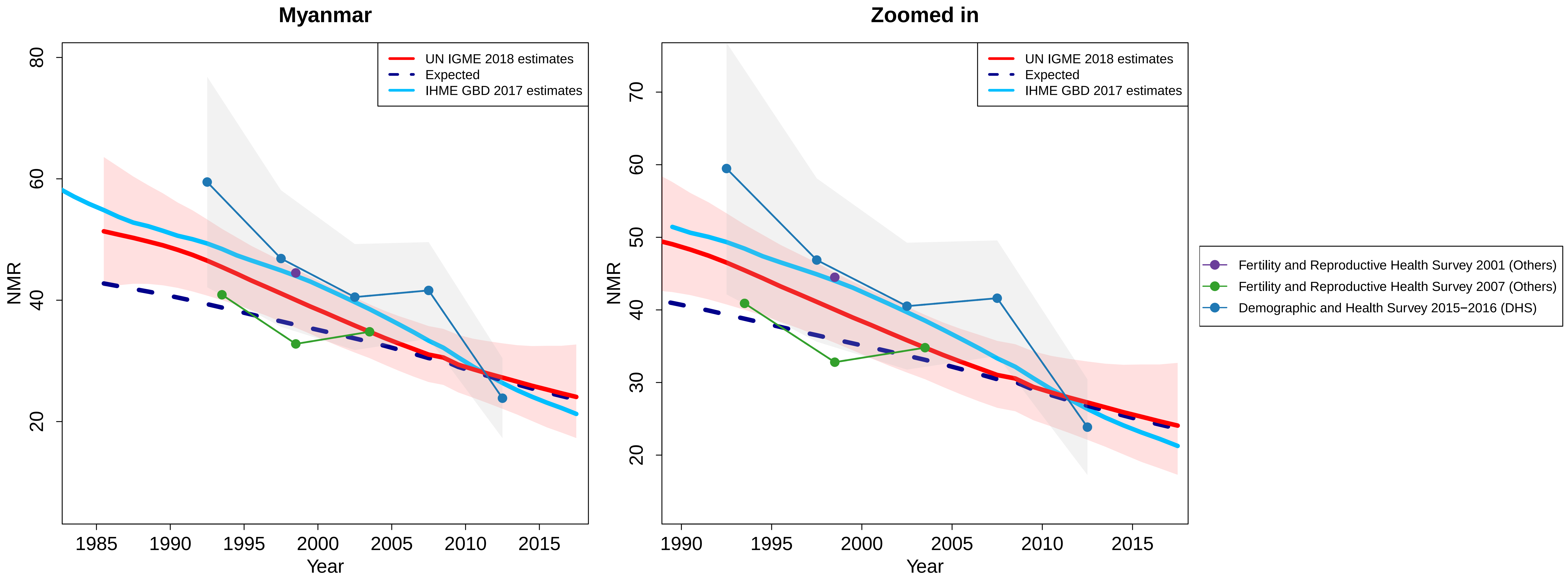

Myanmar
Zoomed in
UN IGME 2018 estimates
Expected
IHME GBD 2017 estimates
NMR
Year
Fertility and Reproductive Health Survey 2001 (Others)
Fertility and Reproductive Health Survey 2007 (Others)
Demographic and Health Survey 2015−2016 (DHS)

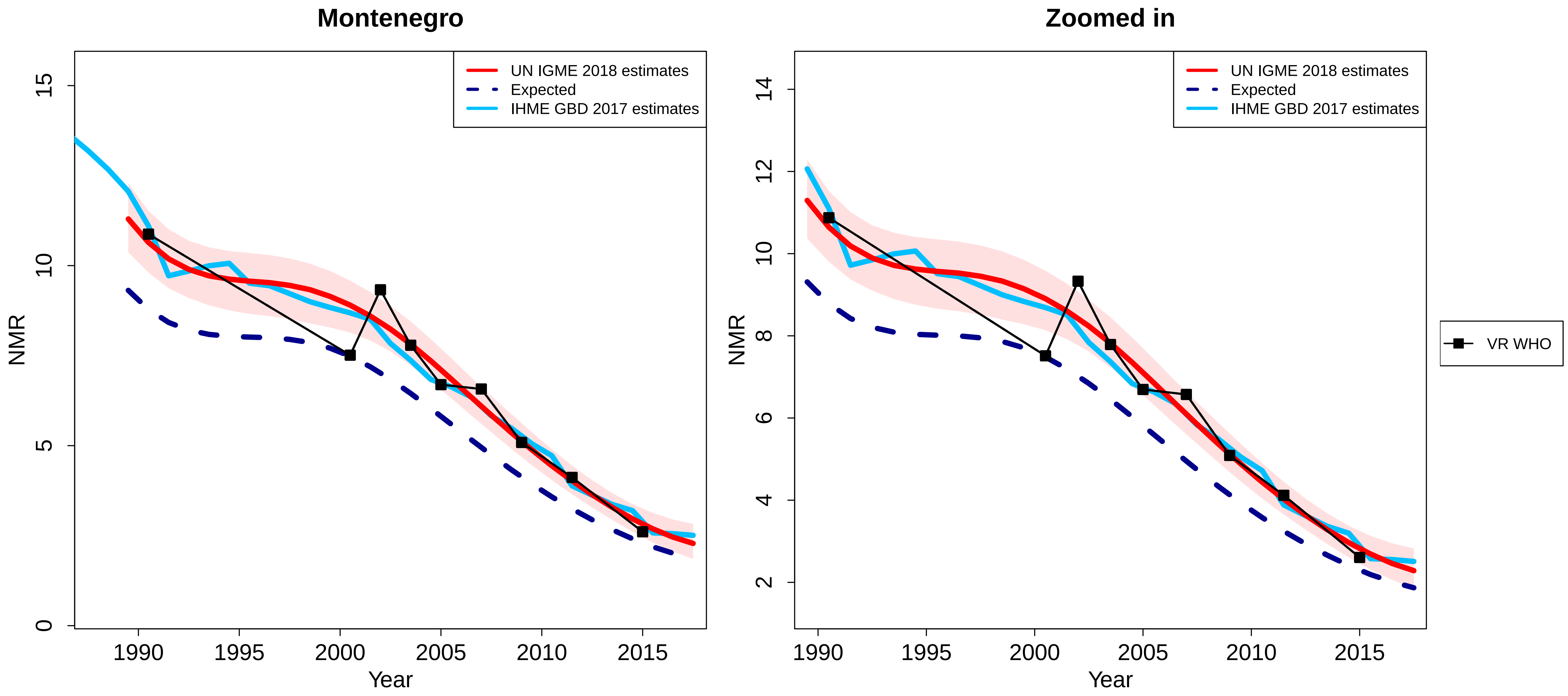
Montenegro
Zoomed in
UN IGME 2018 estimates
Expected
IHME GBD 2017 estimates
VR WHO
NMR
Year
0
5
10
15
2
4
6
8
10
12
14
1990
1995
2000
2005
2010
2015

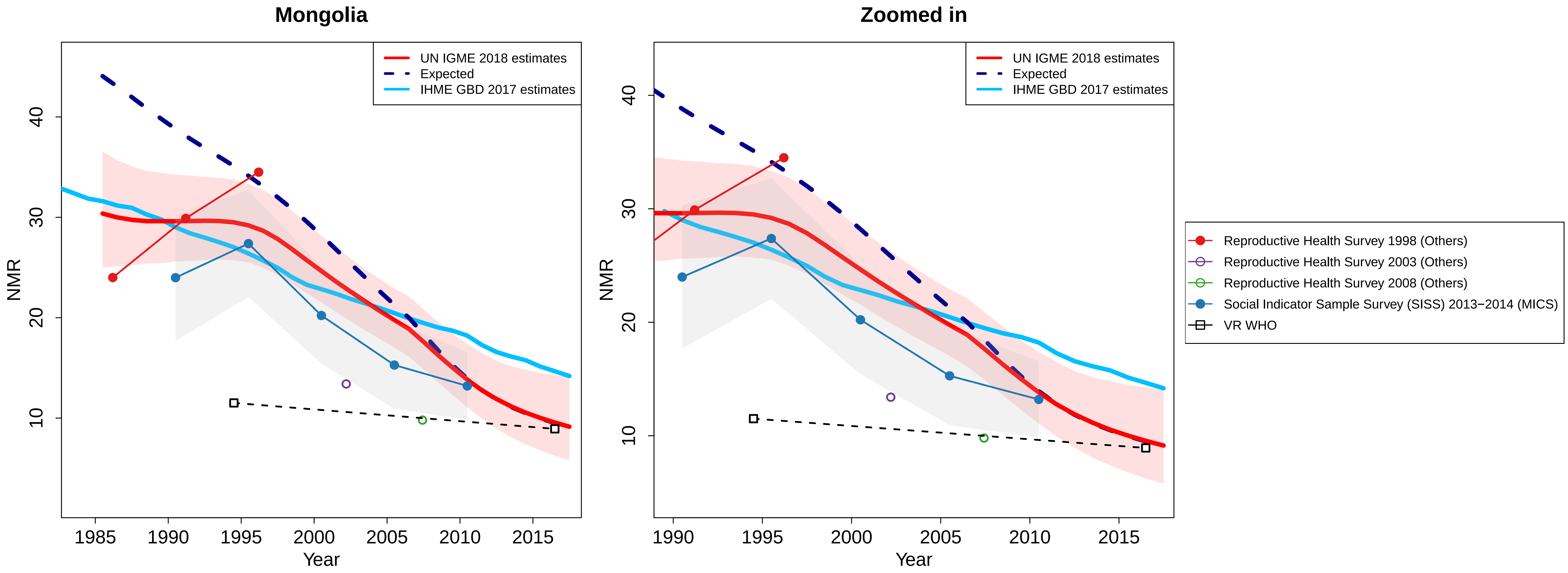
Mongolia
Zoomed in
UN IGME 2018 estimates
Expected
IHME GBD 2017 estimates
NMR
Year
Reproductive Health Survey 1998 (Others)
Reproductive Health Survey 2003 (Others)
Reproductive Health Survey 2008 (Others)
Social Indicator Sample Survey (SISS) 2013−2014 (MICS)
VR WHO

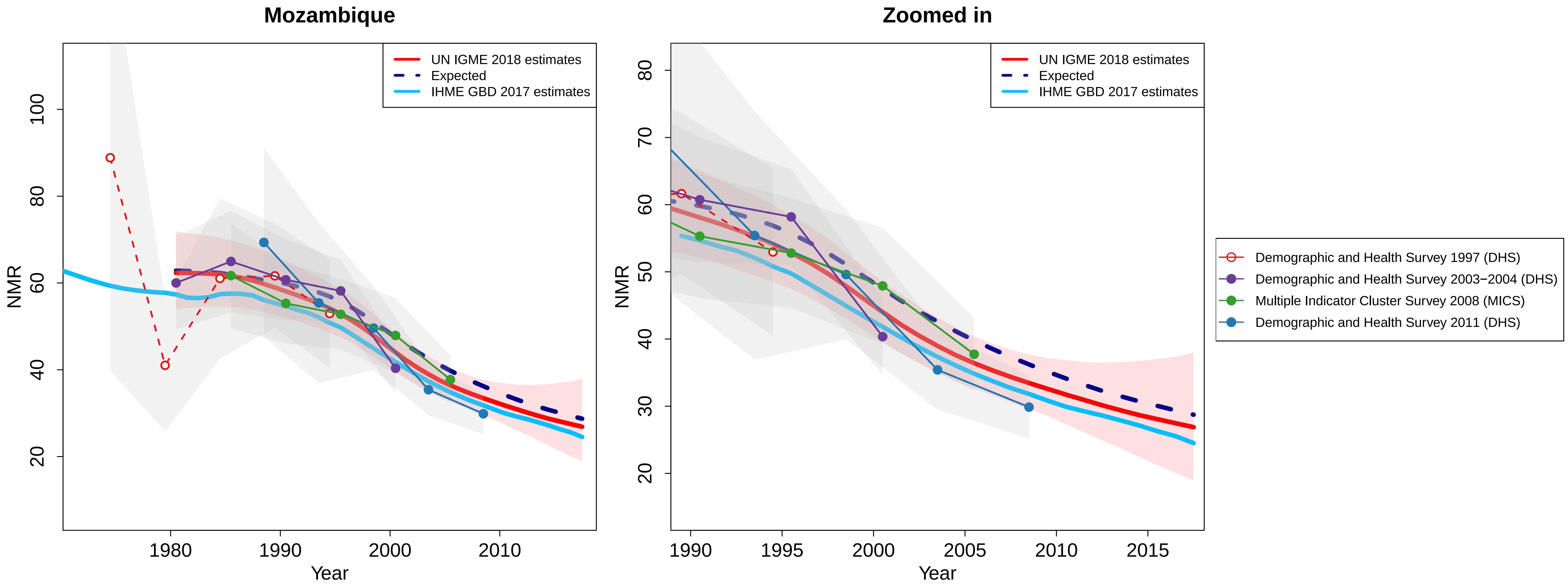

Mozambique
Zoomed in
UN IGME 2018 estimates
Expected
IHME GBD 2017 estimates
NMR
Year
Demographic and Health Survey 1997 (DHS)
Demographic and Health Survey 2003−2004 (DHS)
Multiple Indicator Cluster Survey 2008 (MICS)
Demographic and Health Survey 2011 (DHS)

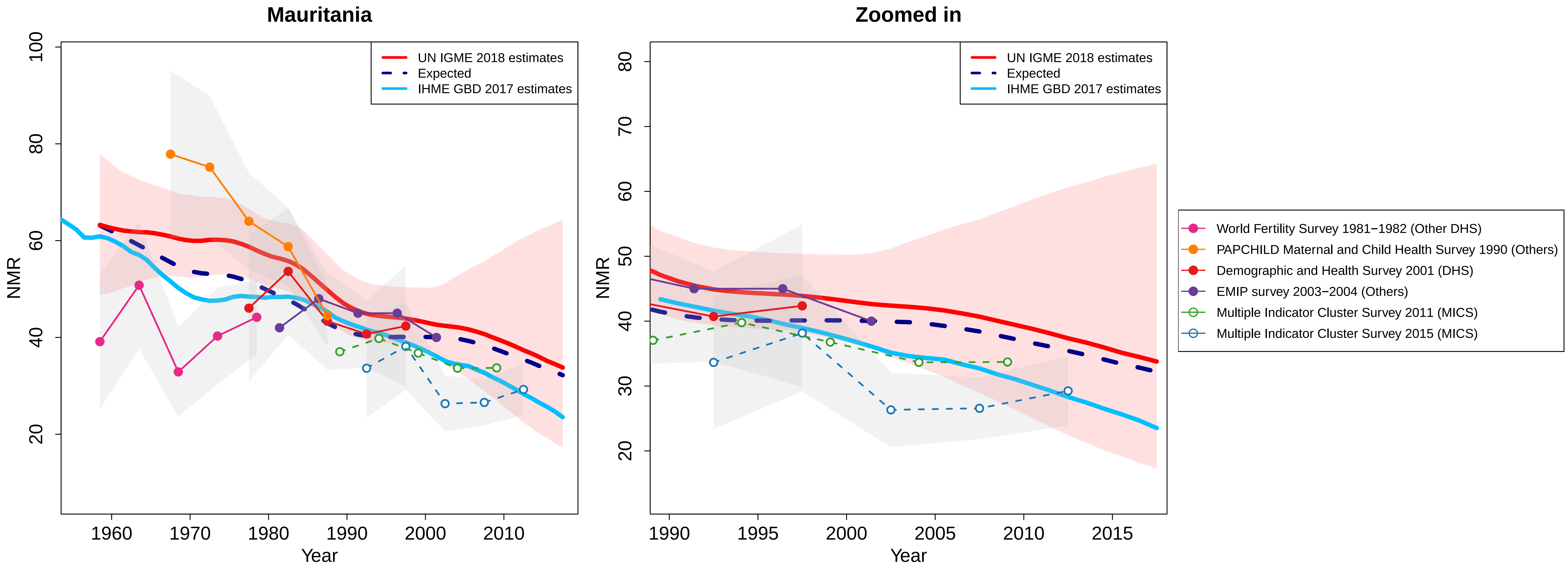

Mauritania
Zoomed in
NMR
Year
UN IGME 2018 estimates
Expected
IHME GBD 2017 estimates
World Fertility Survey 1981−1982 (Other DHS)
PAPCHILD Maternal and Child Health Survey 1990 (Others)
Demographic and Health Survey 2001 (DHS)
EMIP survey 2003−2004 (Others)
Multiple Indicator Cluster Survey 2011 (MICS)
Multiple Indicator Cluster Survey 2015 (MICS)

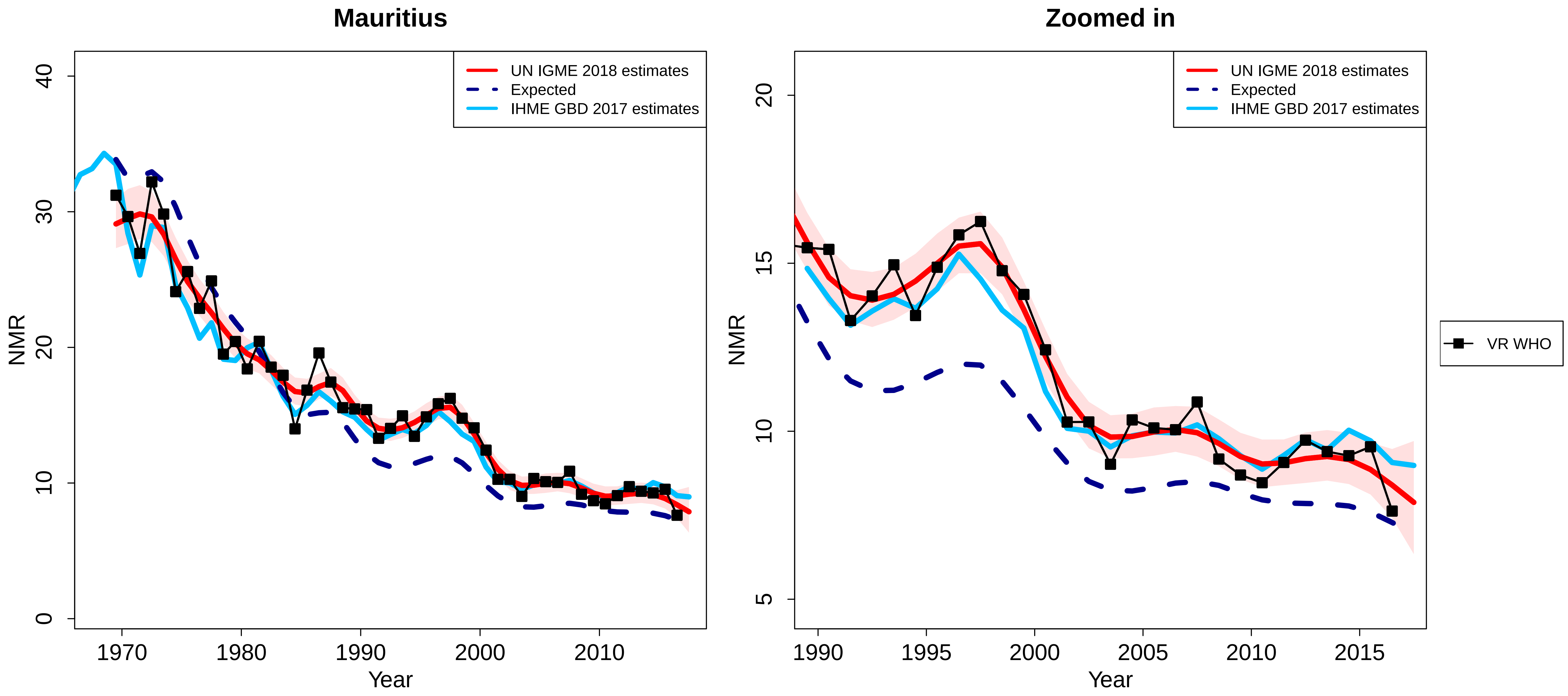

Mauritius
Zoomed in
UN IGME 2018 estimates
Expected
IHME GBD 2017 estimates
NMR
Year
VR WHO

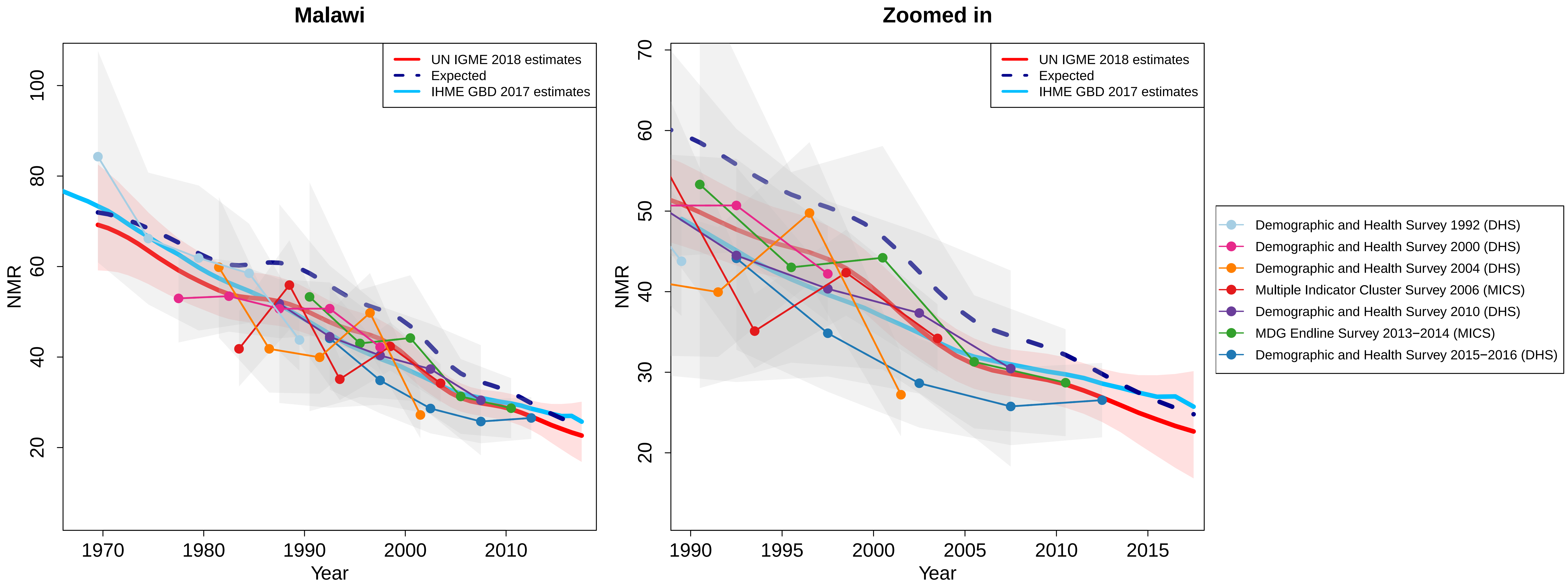

Malawi
Zoomed in
UN IGME 2018 estimates
Expected
IHME GBD 2017 estimates
NMR
Year
Demographic and Health Survey 1992 (DHS)
Demographic and Health Survey 2000 (DHS)
Demographic and Health Survey 2004 (DHS)
Multiple Indicator Cluster Survey 2006 (MICS)
Demographic and Health Survey 2010 (DHS)
MDG Endline Survey 2013−2014 (MICS)
Demographic and Health Survey 2015−2016 (DHS)

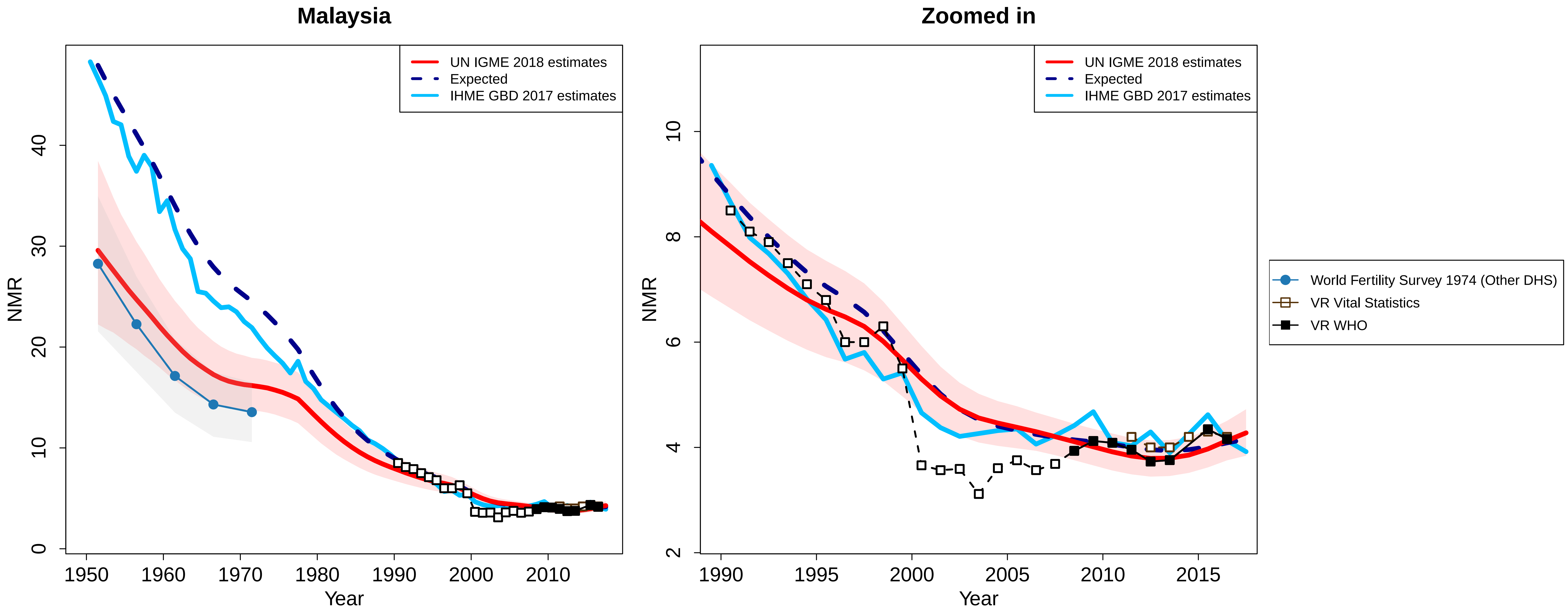
Malaysia
Zoomed in
UN IGME 2018 estimates
Expected
IHME GBD 2017 estimates
World Fertility Survey 1974 (Other DHS)
VR Vital Statistics
VR WHO
NMR
Year
0
10
20
30
40
1950
1960
1970
1980
1990
2000
2010
2
4
6
8
10
1995
2005
2015

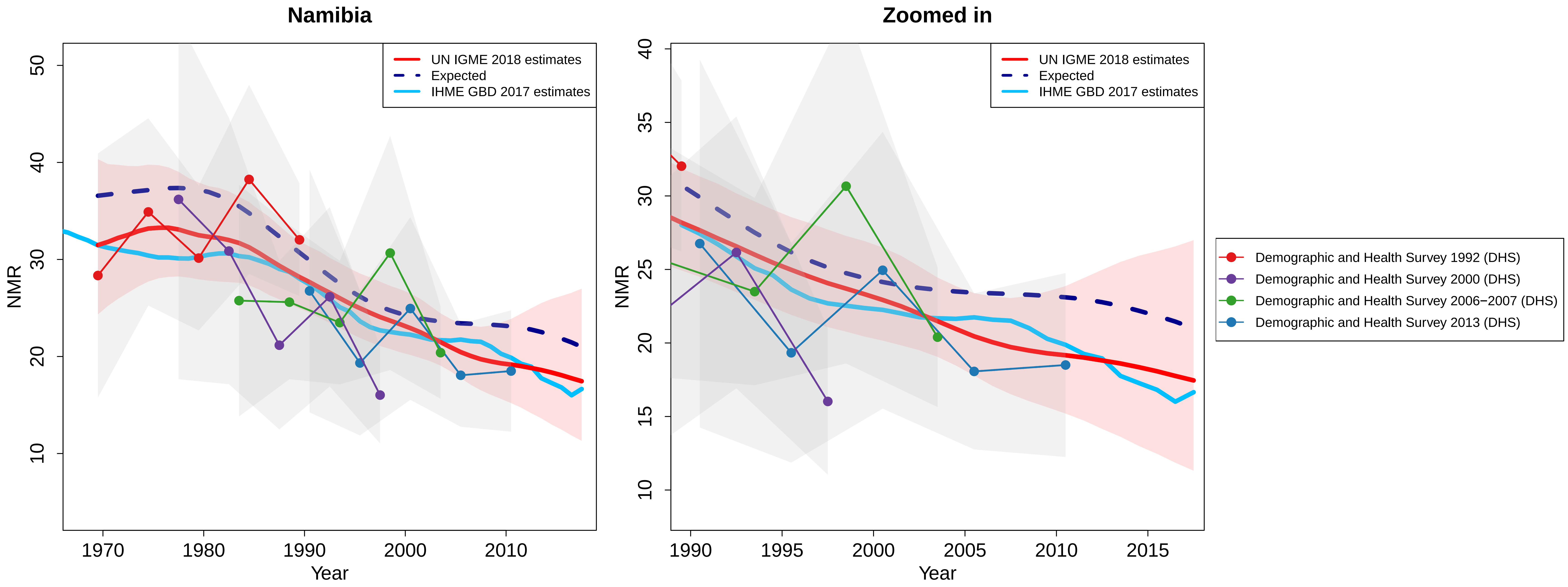
Namibia
Zoomed in
UN IGME 2018 estimates
Expected
IHME GBD 2017 estimates
NMR
Year
Demographic and Health Survey 1992 (DHS)
Demographic and Health Survey 2000 (DHS)
Demographic and Health Survey 2006−2007 (DHS)
Demographic and Health Survey 2013 (DHS)

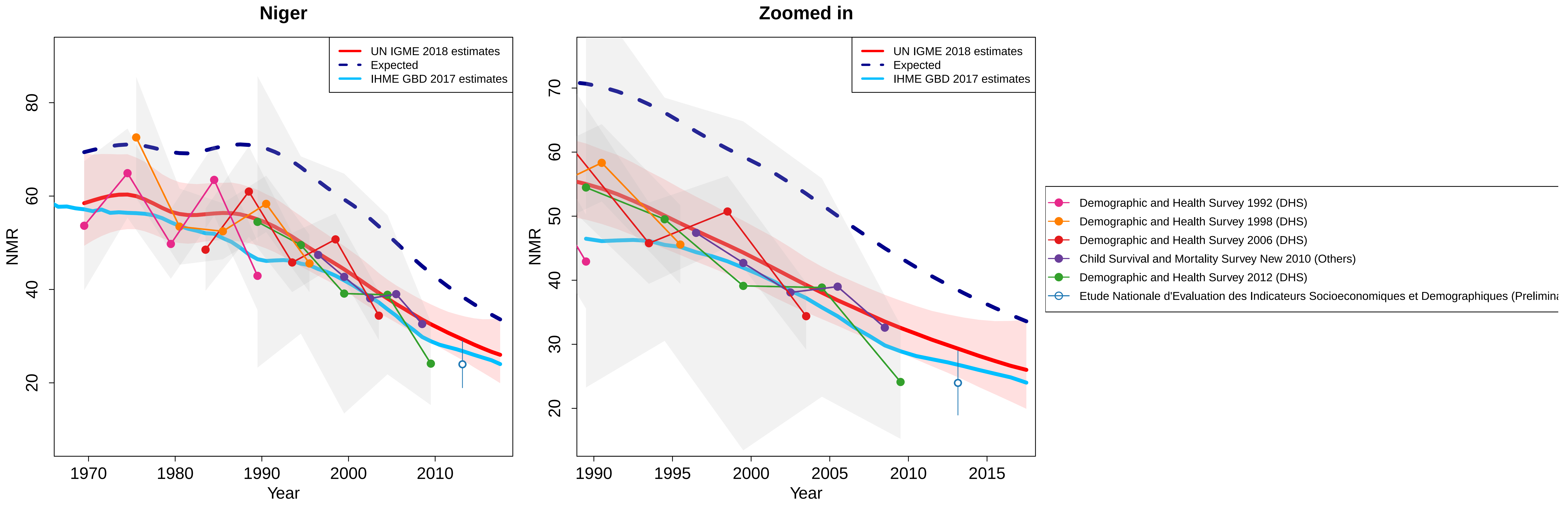

Niger
Zoomed in
UN IGME 2018 estimates
Expected
IHME GBD 2017 estimates
NMR
Year
20
40
60
80
1970
1980
1990
2000
2010
20
30
40
50
60
70
1990
1995
2000
2005
2010
2015
Demographic and Health Survey 1992 (DHS)
Demographic and Health Survey 1998 (DHS)
Demographic and Health Survey 2006 (DHS)
Child Survival and Mortality Survey New 2010 (Others)
Demographic and Health Survey 2012 (DHS)
Etude Nationale d'Evaluation des Indicateurs Socioeconomiques et Demographiques (Prelimina

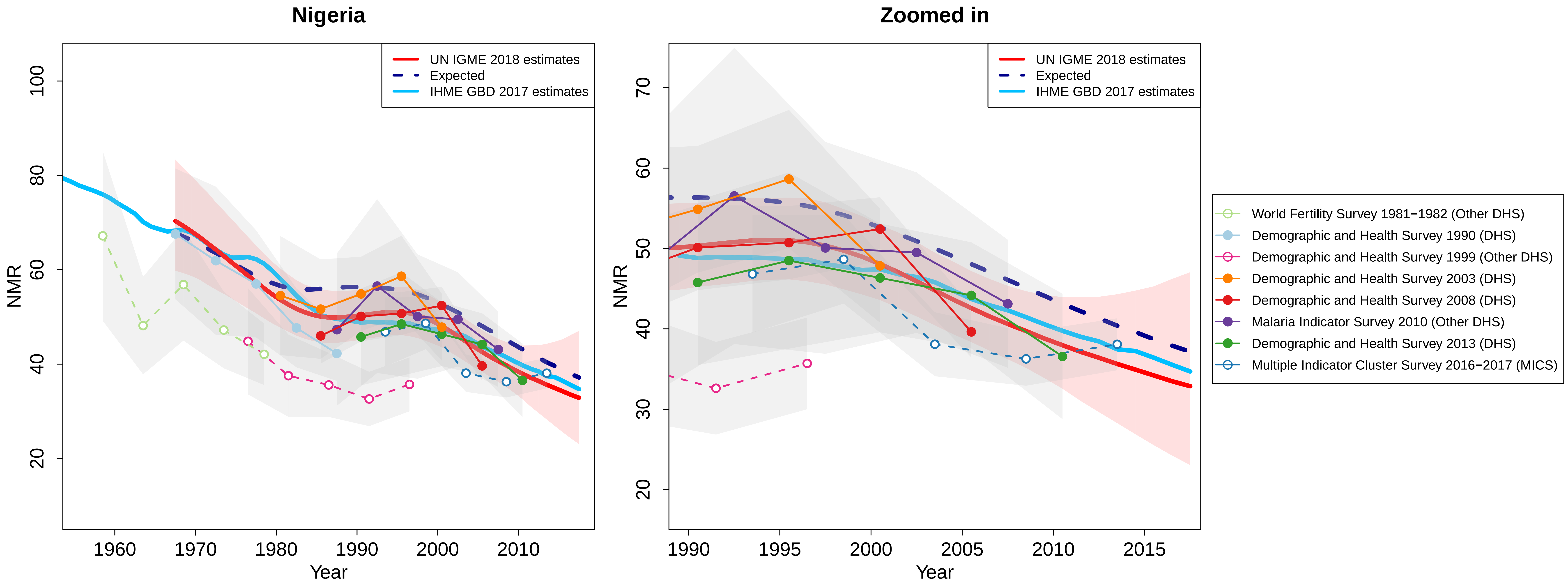
Nigeria
Zoomed in
UN IGME 2018 estimates
Expected
IHME GBD 2017 estimates
NMR
Year
World Fertility Survey 1981−1982 (Other DHS)
Demographic and Health Survey 1990 (DHS)
Demographic and Health Survey 1999 (Other DHS)
Demographic and Health Survey 2003 (DHS)
Demographic and Health Survey 2008 (DHS)
Malaria Indicator Survey 2010 (Other DHS)
Demographic and Health Survey 2013 (DHS)
Multiple Indicator Cluster Survey 2016−2017 (MICS)

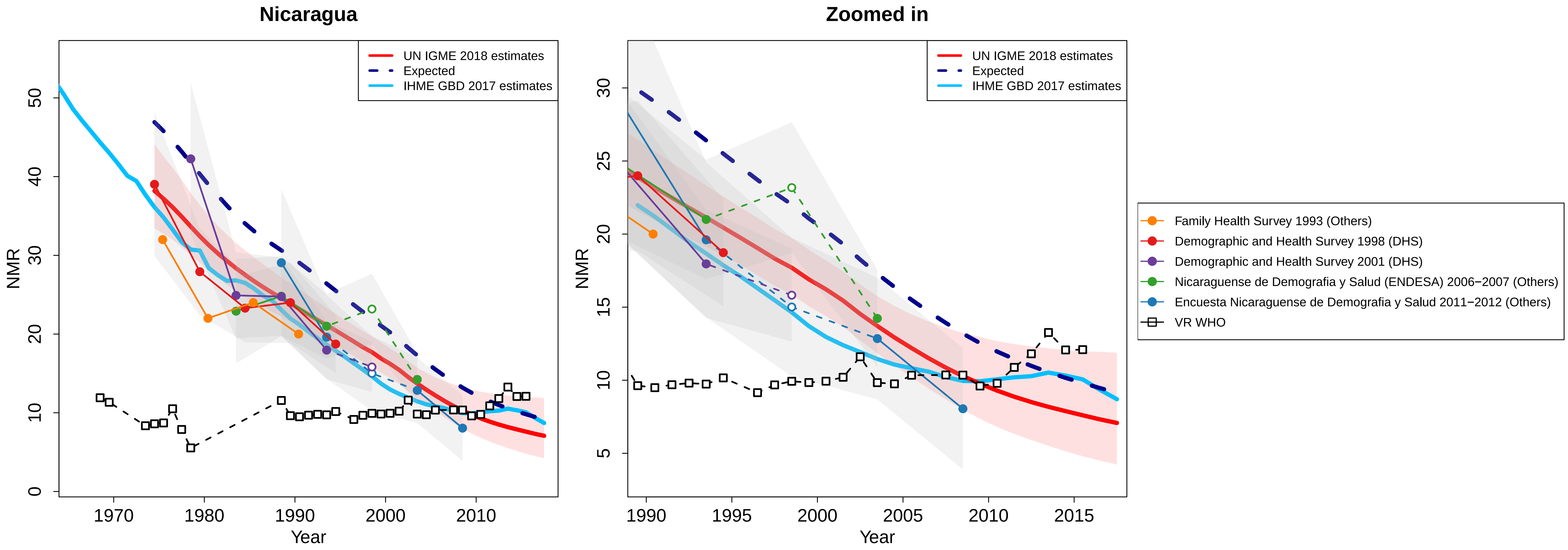
Nicaragua
Zoomed in
UN IGME 2018 estimates
Expected
IHME GBD 2017 estimates
NMR
Year
Family Health Survey 1993 (Others)
Demographic and Health Survey 1998 (DHS)
Demographic and Health Survey 2001 (DHS)
Nicaraguense de Demografia y Salud (ENDESA) 2006−2007 (Others)
Encuesta Nicaraguense de Demografia y Salud 2011−2012 (Others)
VR WHO

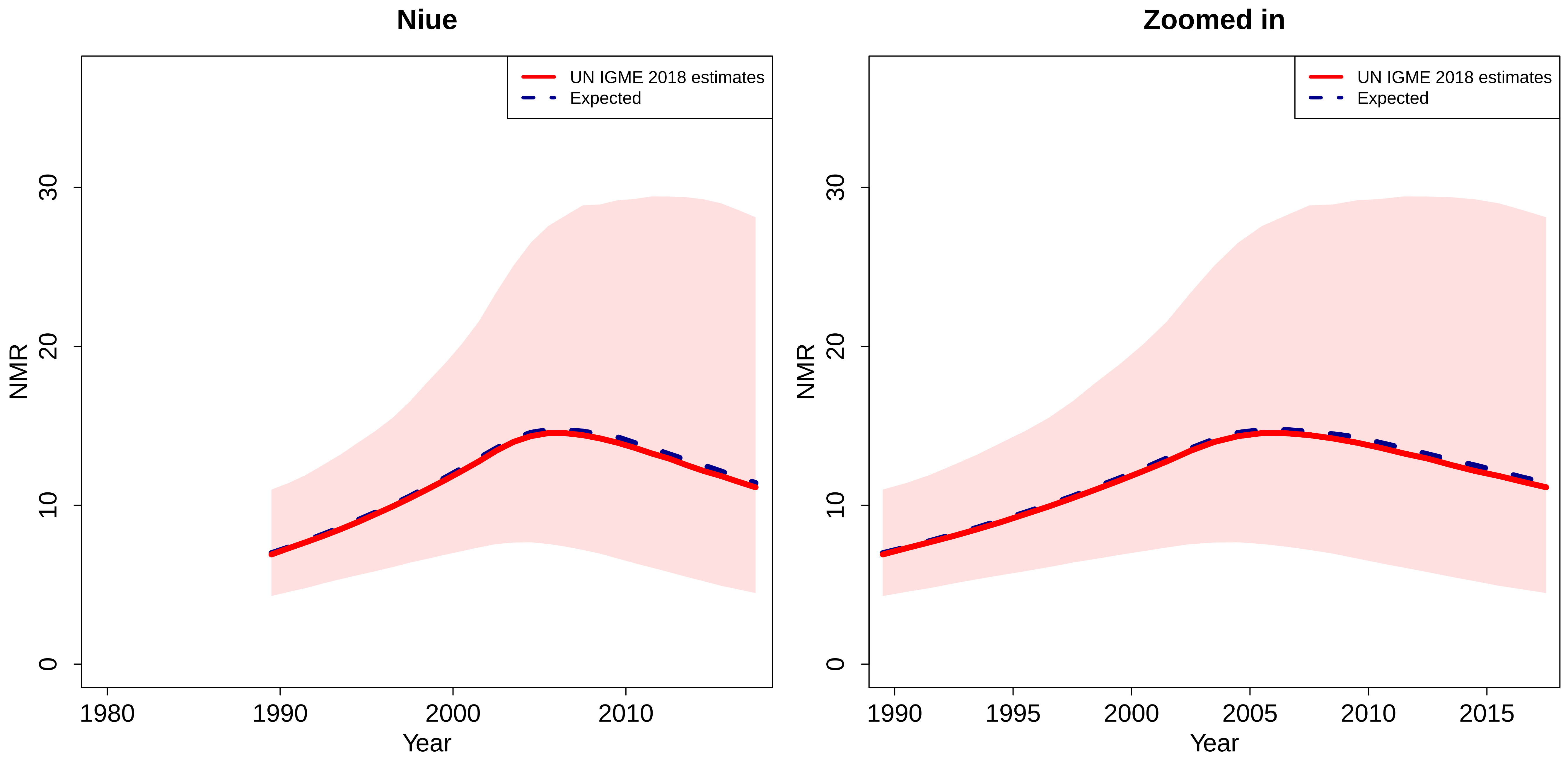
Niue
Zoomed in
UN IGME 2018 estimates
Expected
NMR
Year
0
10
20
30
1980
1990
2000
2010
1995
2005
2015

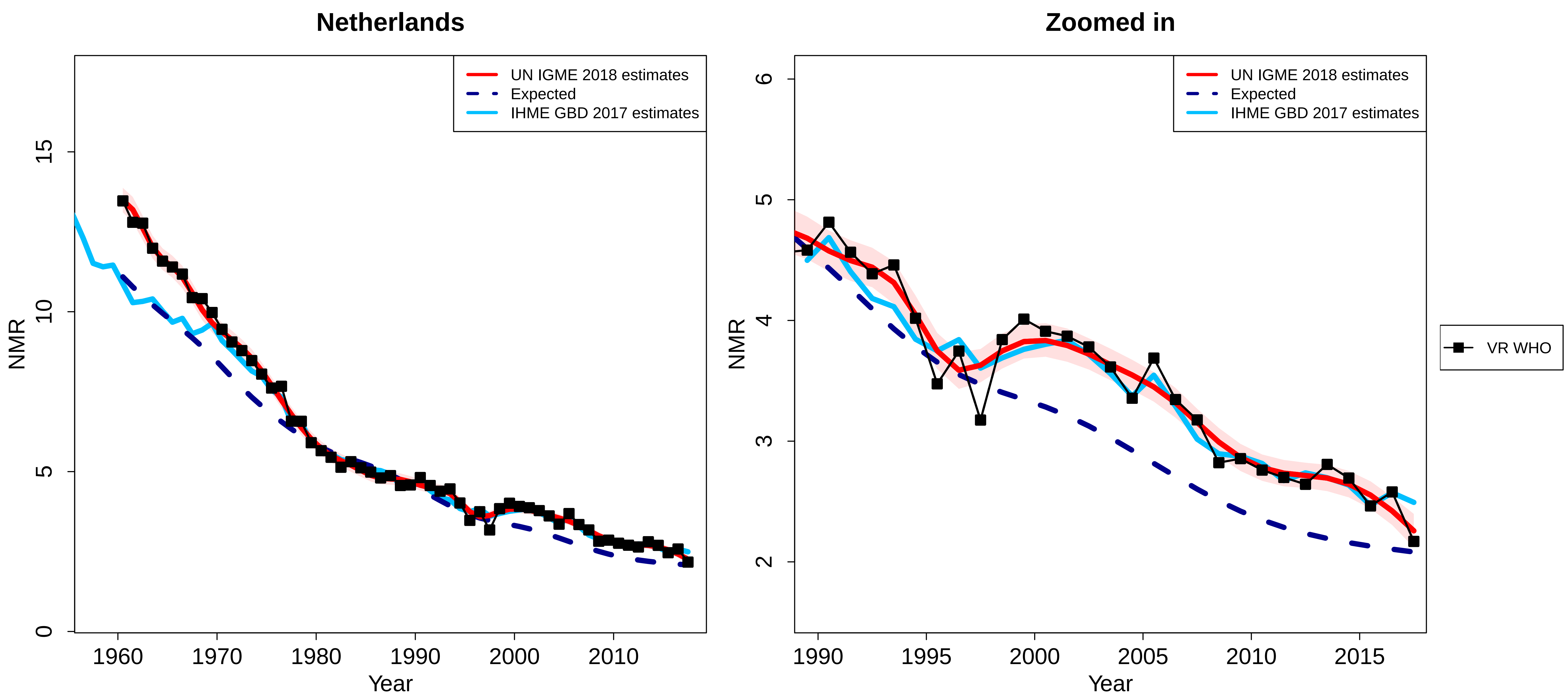
Netherlands
Zoomed in
UN IGME 2018 estimates
Expected
IHME GBD 2017 estimates
VR WHO
NMR
Year
0
5
10
15
1960
1970
1980
1990
2000
2010
2
3
4
5
6
1990
1995
2000
2005
2010
2015

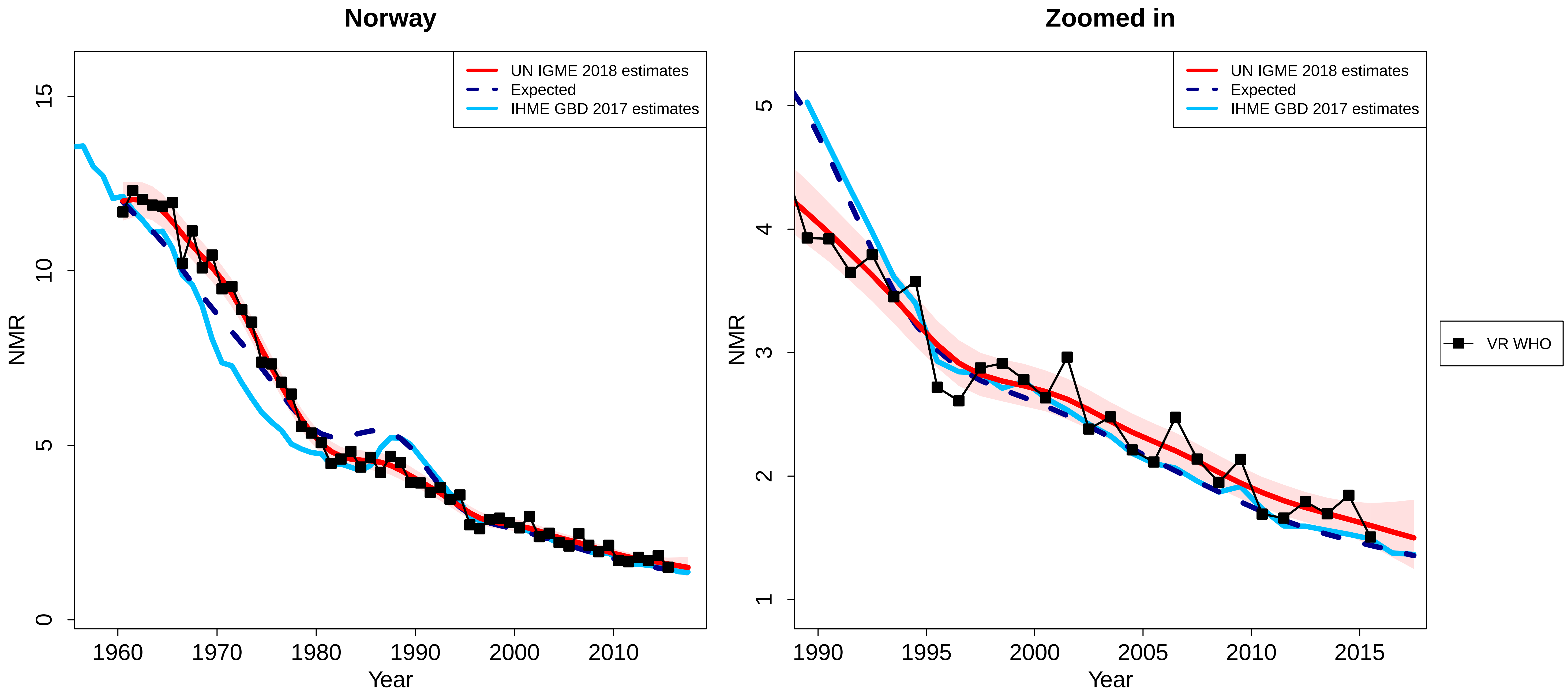
Norway
Zoomed in
UN IGME 2018 estimates
Expected
IHME GBD 2017 estimates
VR WHO
NMR
Year
0
5
10
15
1960
1970
1980
1990
2000
2010
1
2
3
4
5
1990
1995
2000
2005
2010
2015

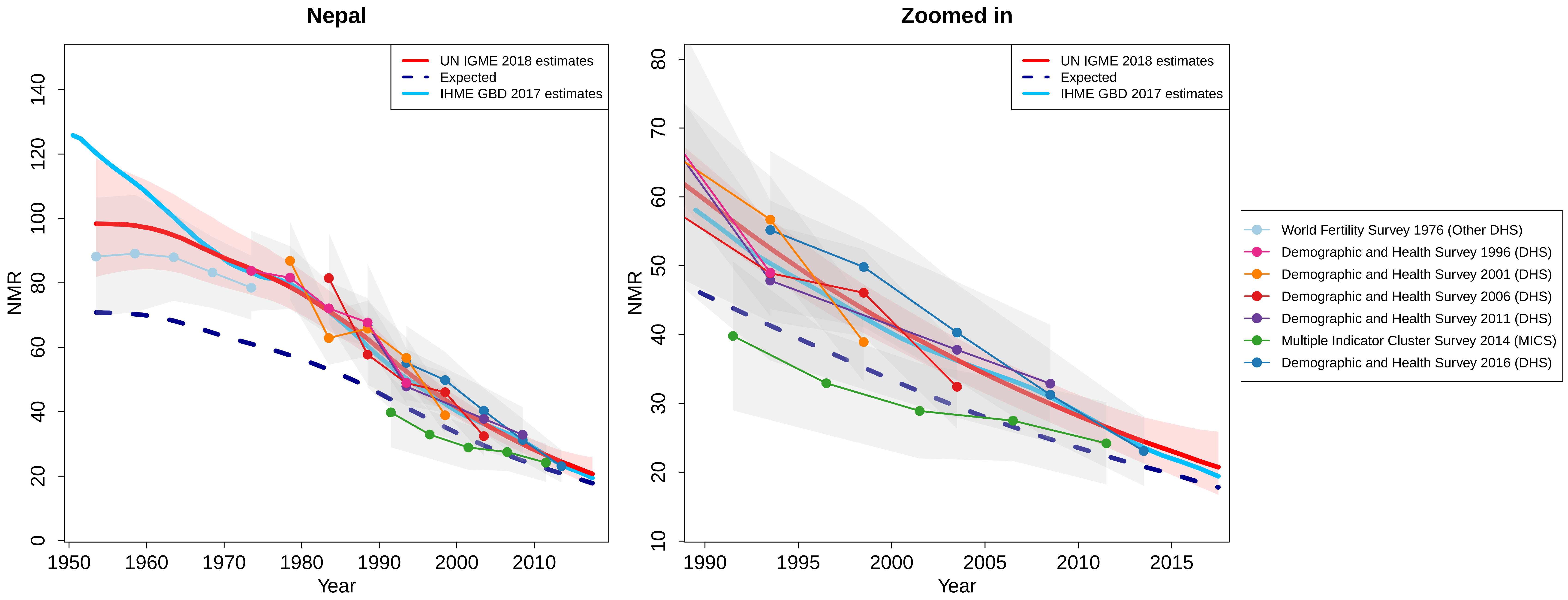

Nepal
Zoomed in
NMR
Year
UN IGME 2018 estimates
Expected
IHME GBD 2017 estimates
World Fertility Survey 1976 (Other DHS)
Demographic and Health Survey 1996 (DHS)
Demographic and Health Survey 2001 (DHS)
Demographic and Health Survey 2006 (DHS)
Demographic and Health Survey 2011 (DHS)
Multiple Indicator Cluster Survey 2014 (MICS)
Demographic and Health Survey 2016 (DHS)

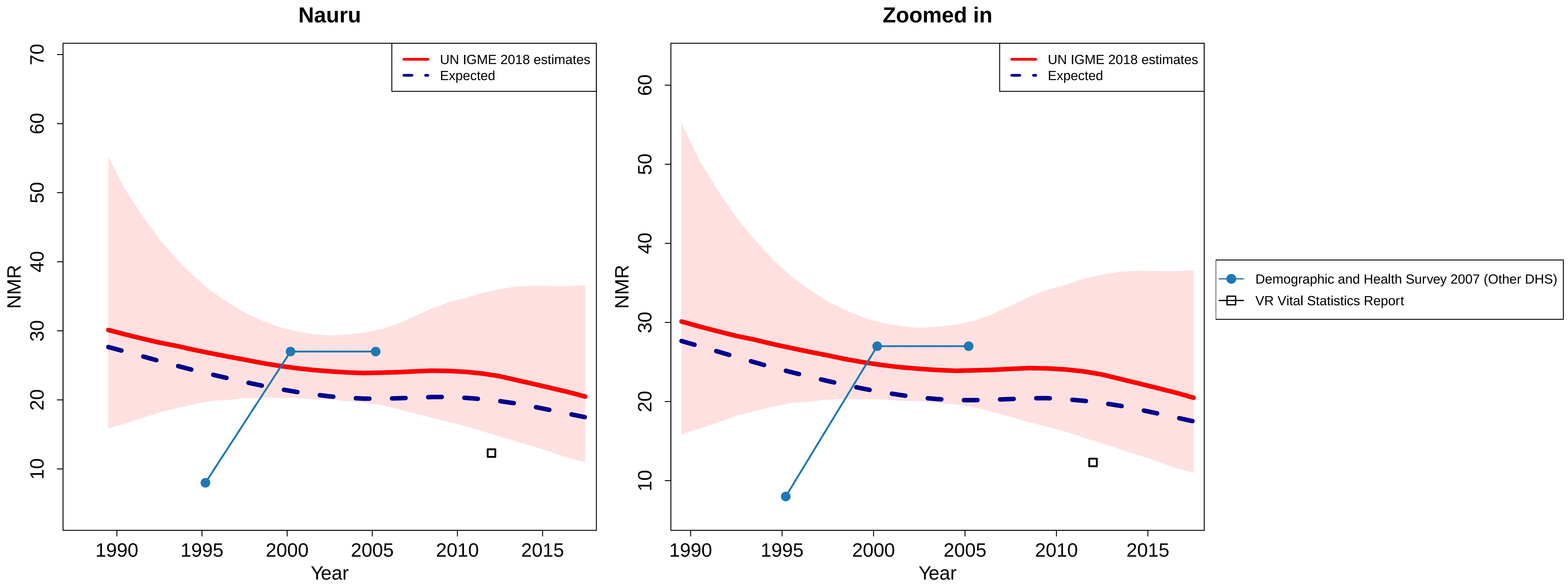

Nauru
Zoomed in
UN IGME 2018 estimates
Expected
NMR
Year
10
20
30
40
50
60
70
1990
1995
2000
2005
2010
2015
Demographic and Health Survey 2007 (Other DHS)
VR Vital Statistics Report

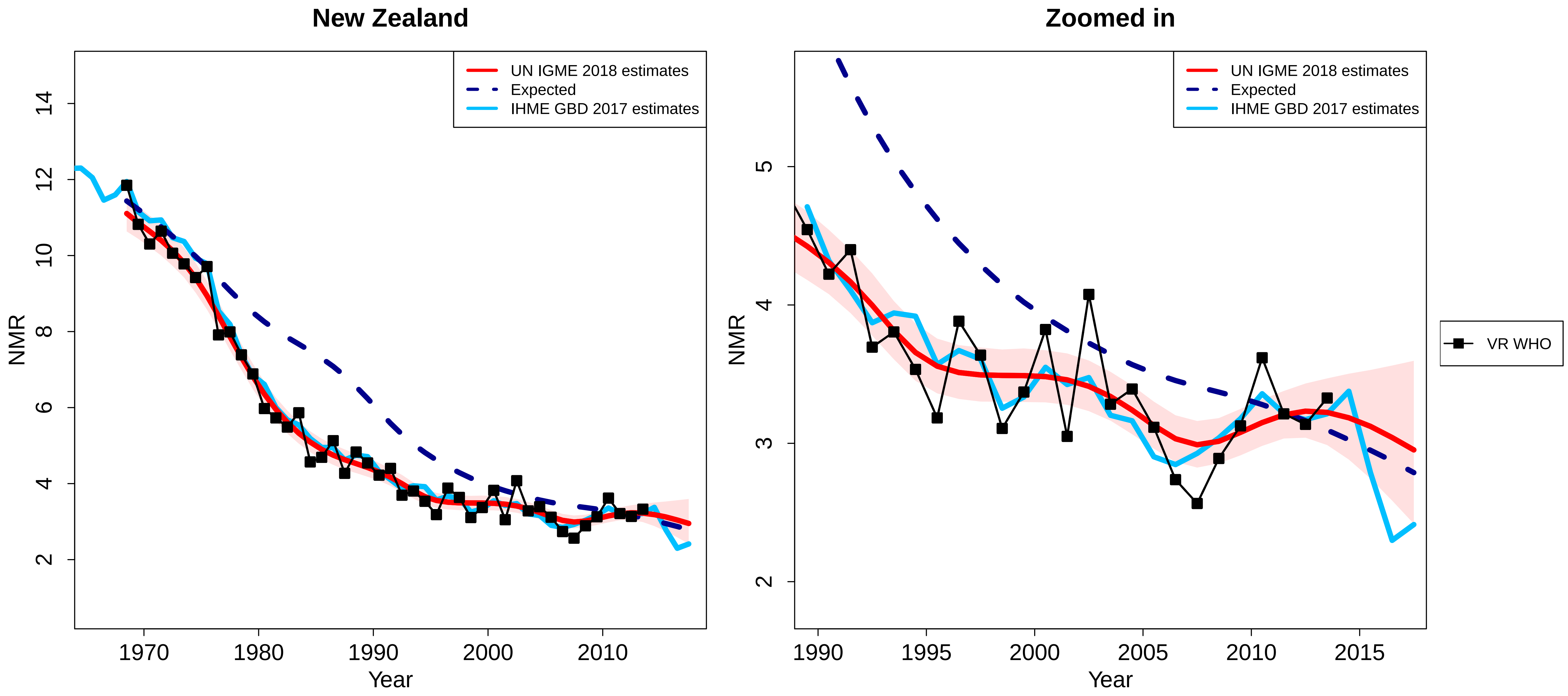
New Zealand
Zoomed in
UN IGME 2018 estimates
Expected
IHME GBD 2017 estimates
VR WHO
NMR
Year
1970
1980
1990
2000
2010
1995
2005
2015
2
3
4
5
6
8
10
12
14

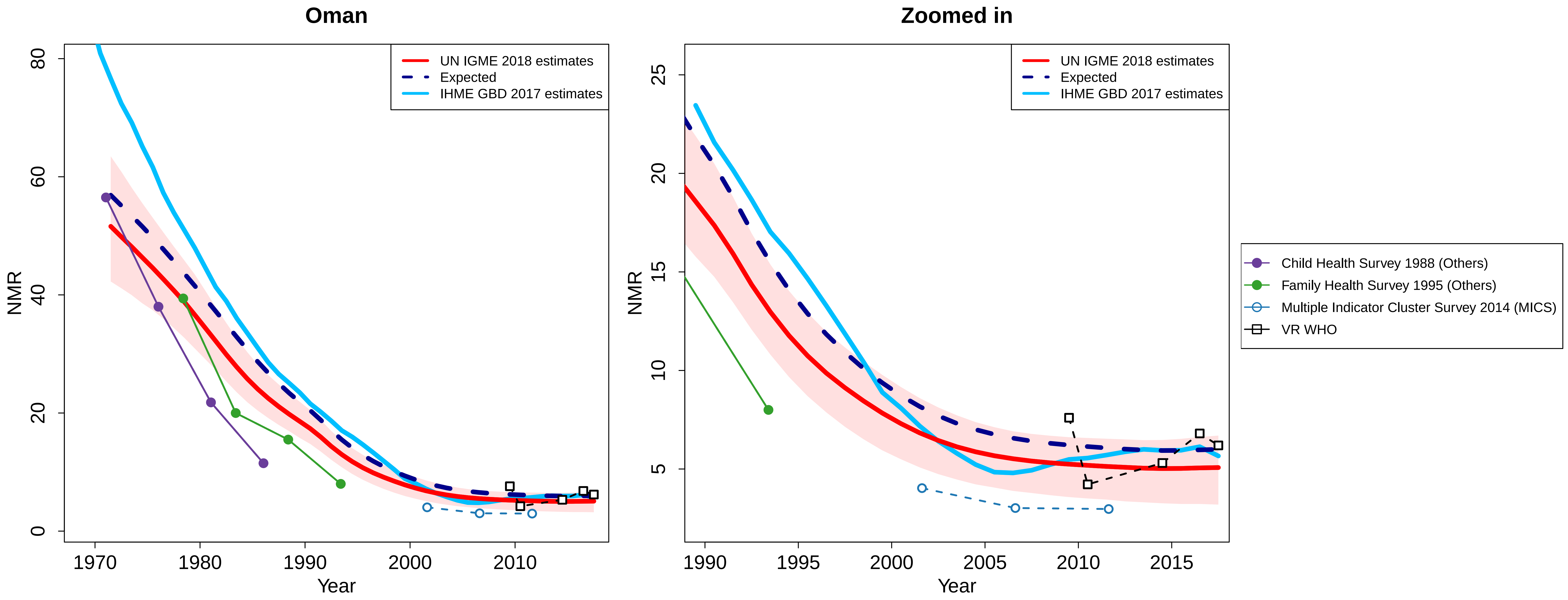
Oman
Zoomed in
UN IGME 2018 estimates
Expected
IHME GBD 2017 estimates
NMR
Year
Child Health Survey 1988 (Others)
Family Health Survey 1995 (Others)
Multiple Indicator Cluster Survey 2014 (MICS)
VR WHO

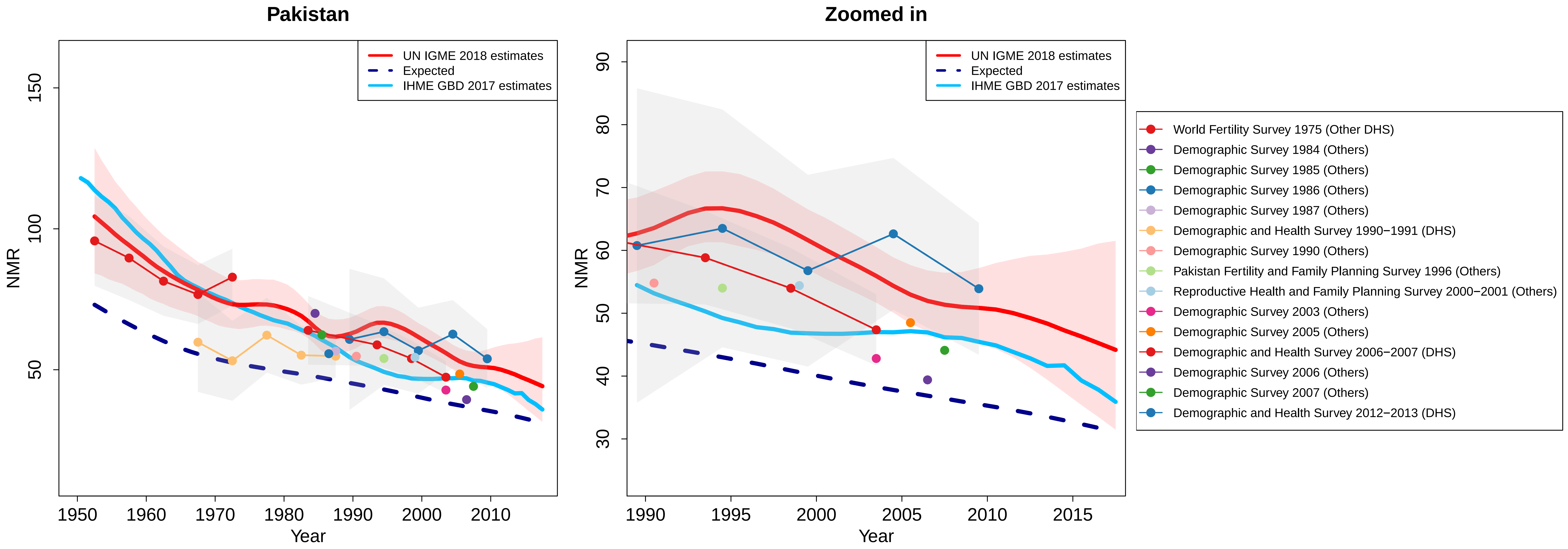

Pakistan
Zoomed in
UN IGME 2018 estimates
Expected
IHME GBD 2017 estimates
NMR
Year
150
100
50
1950
1960
1970
1980
1990
2000
2010
90
80
70
60
50
40
30
1990
1995
2000
2005
2010
2015
World Fertility Survey 1975 (Other DHS)
Demographic Survey 1984 (Others)
Demographic Survey 1985 (Others)
Demographic Survey 1986 (Others)
Demographic Survey 1987 (Others)
Demographic and Health Survey 1990−1991 (DHS)
Demographic Survey 1990 (Others)
Pakistan Fertility and Family Planning Survey 1996 (Others)
Reproductive Health and Family Planning Survey 2000−2001 (Others)
Demographic Survey 2003 (Others)
Demographic Survey 2005 (Others)
Demographic and Health Survey 2006−2007 (DHS)
Demographic Survey 2006 (Others)
Demographic Survey 2007 (Others)
Demographic and Health Survey 2012−2013 (DHS)

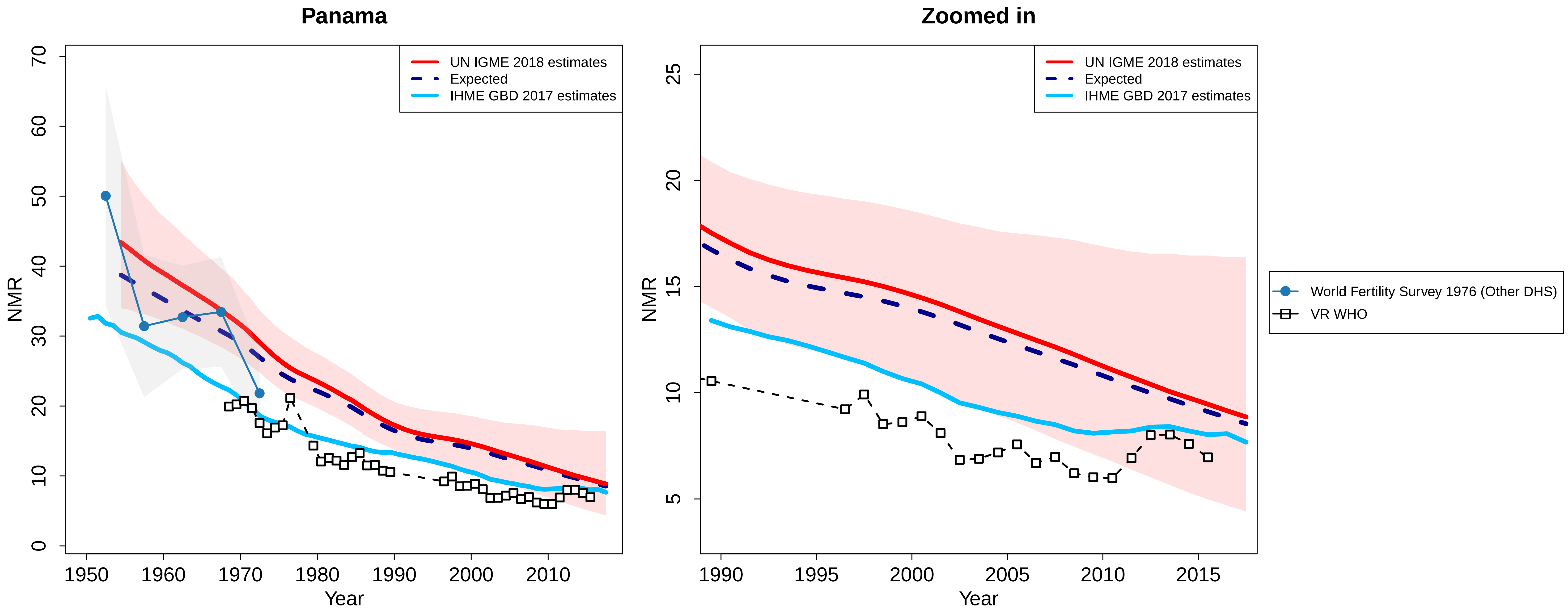

Panama
Zoomed in
UN IGME 2018 estimates
Expected
IHME GBD 2017 estimates
World Fertility Survey 1976 (Other DHS)
VR WHO
NMR
Year

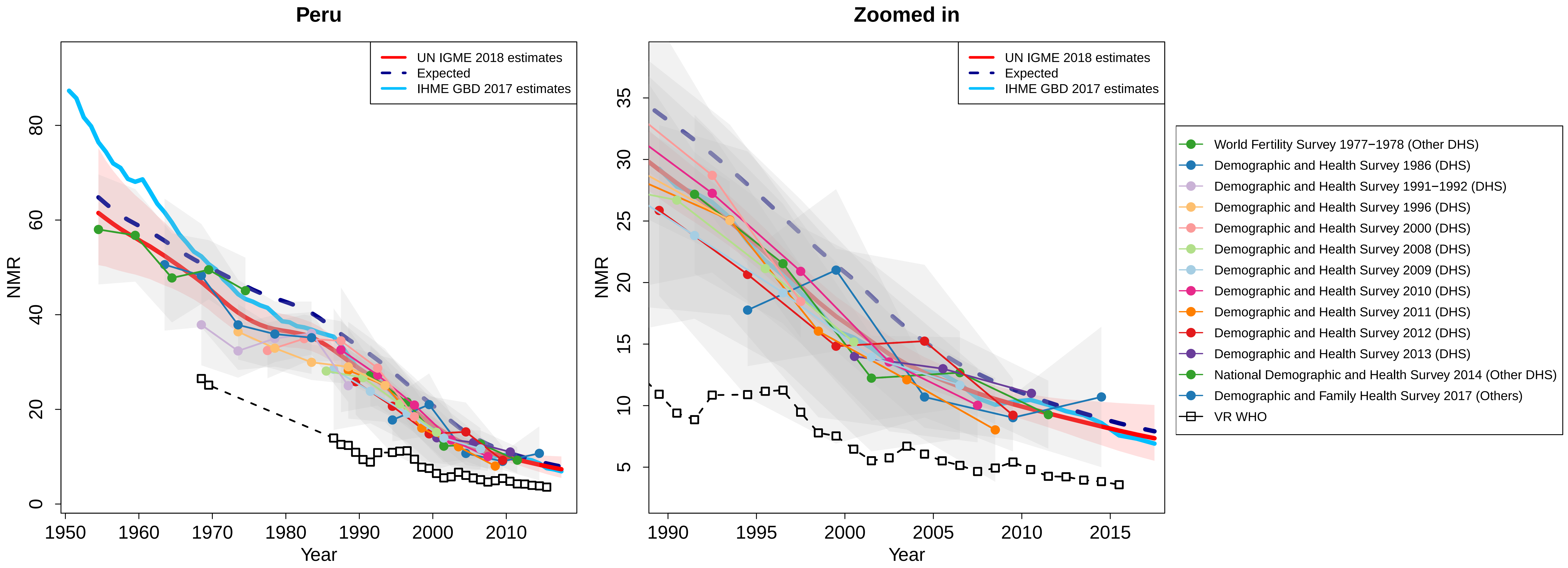
Peru
Zoomed in
UN IGME 2018 estimates
Expected
IHME GBD 2017 estimates
NMR
Year
World Fertility Survey 1977−1978 (Other DHS)
Demographic and Health Survey 1986 (DHS)
Demographic and Health Survey 1991−1992 (DHS)
Demographic and Health Survey 1996 (DHS)
Demographic and Health Survey 2000 (DHS)
Demographic and Health Survey 2008 (DHS)
Demographic and Health Survey 2009 (DHS)
Demographic and Health Survey 2010 (DHS)
Demographic and Health Survey 2011 (DHS)
Demographic and Health Survey 2012 (DHS)
Demographic and Health Survey 2013 (DHS)
National Demographic and Health Survey 2014 (Other DHS)
Demographic and Family Health Survey 2017 (Others)
VR WHO

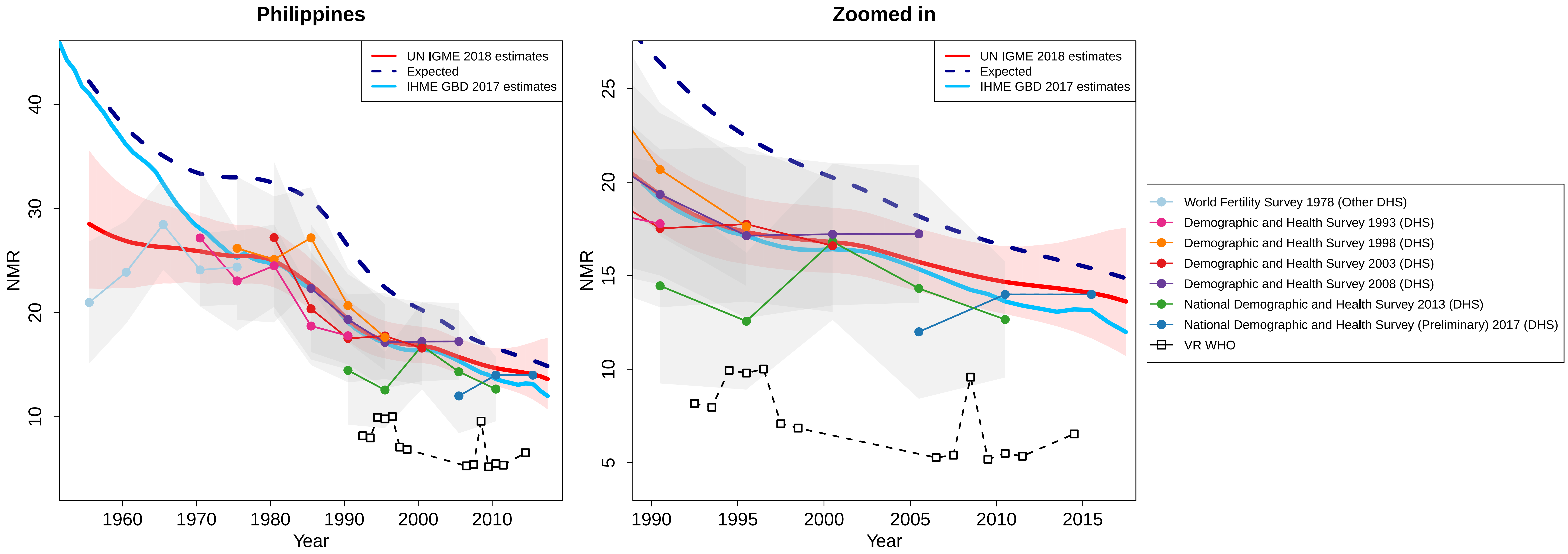

Philippines
Zoomed in
UN IGME 2018 estimates
Expected
IHME GBD 2017 estimates
NMR
Year
1960
1970
1980
1990
2000
2010
10
20
30
40
1990
1995
2000
2005
2010
2015
5
10
15
20
25
World Fertility Survey 1978 (Other DHS)
Demographic and Health Survey 1993 (DHS)
Demographic and Health Survey 1998 (DHS)
Demographic and Health Survey 2003 (DHS)
Demographic and Health Survey 2008 (DHS)
National Demographic and Health Survey 2013 (DHS)
National Demographic and Health Survey (Preliminary) 2017 (DHS)
VR WHO

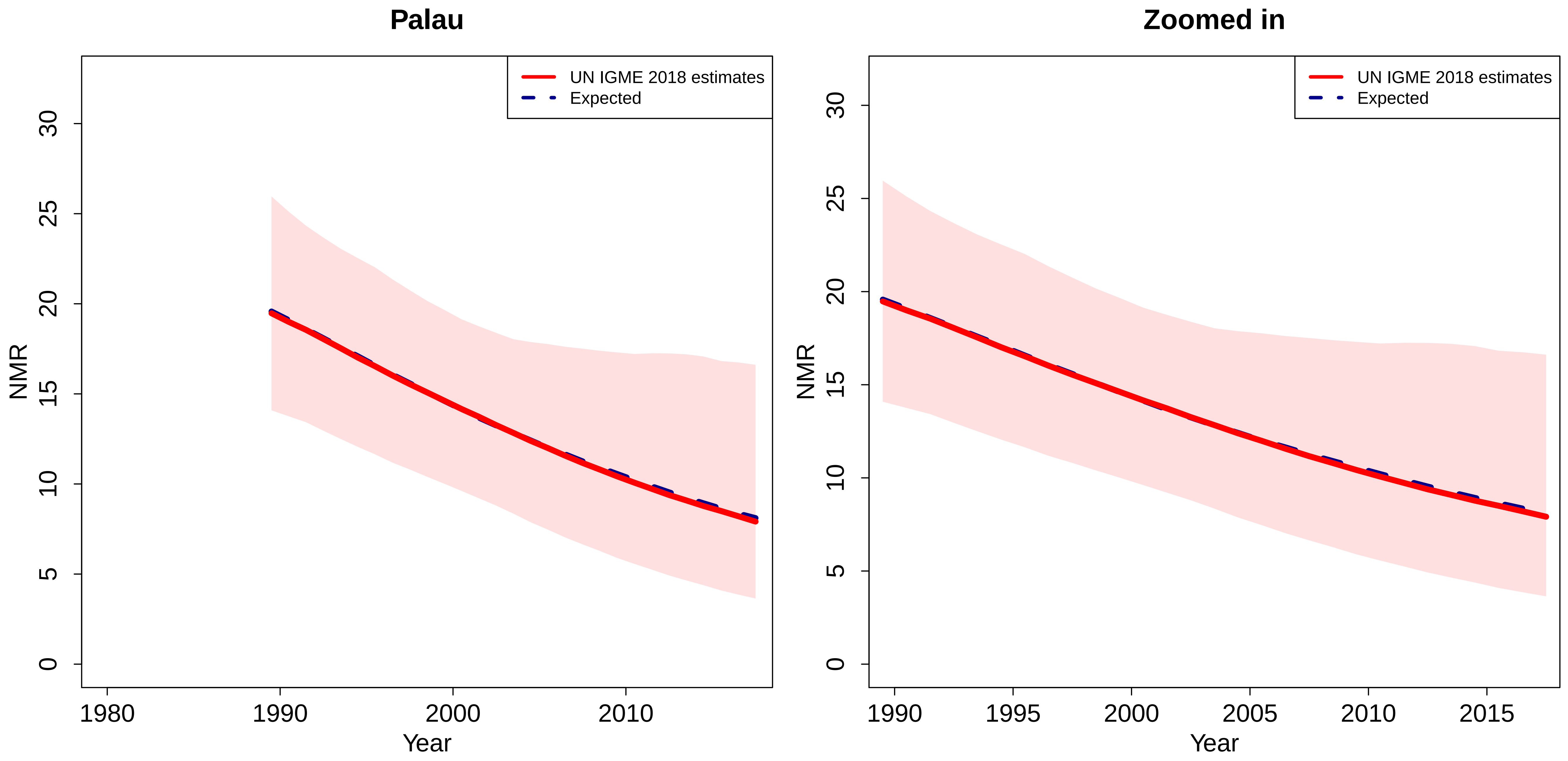

Palau
UN IGME 2018 estimates
Expected
NMR
0
5
10
15
20
25
30
1980
1990
2000
2010
Year
Zoomed in
UN IGME 2018 estimates
Expected
NMR
0
5
10
15
20
25
30
1990
1995
2000
2005
2010
2015
Year

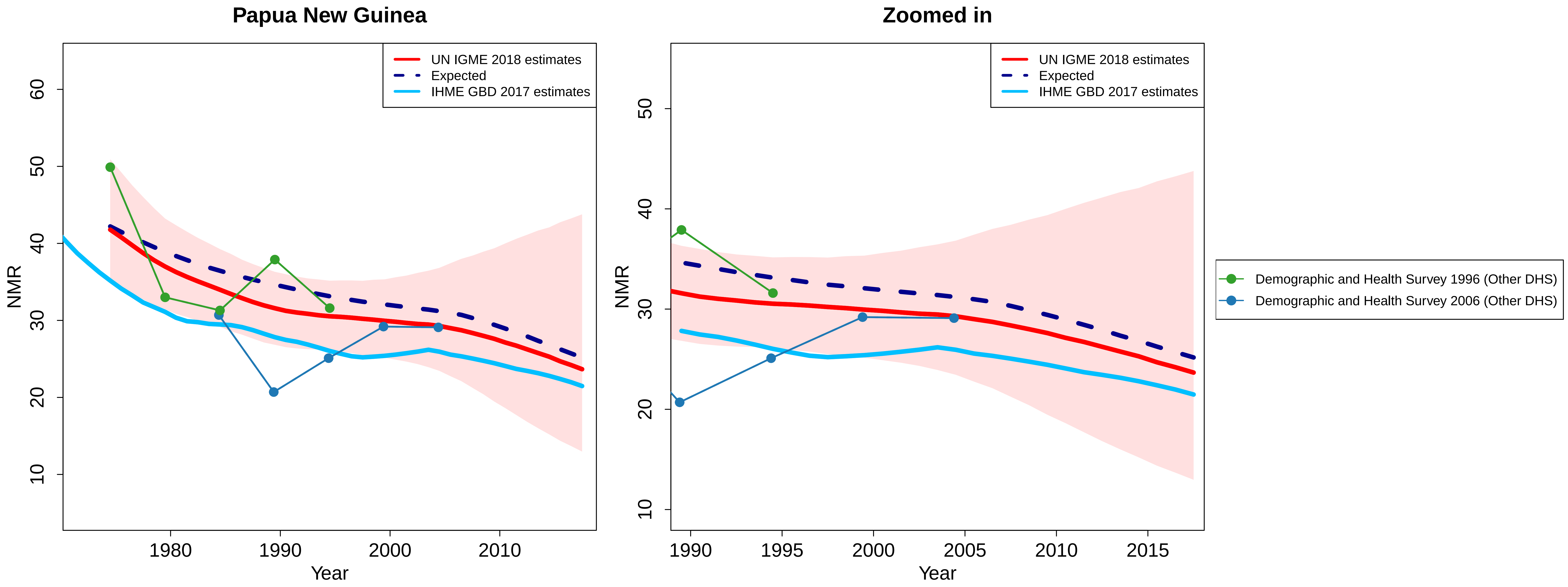

Papua New Guinea
Zoomed in
UN IGME 2018 estimates
Expected
IHME GBD 2017 estimates
Demographic and Health Survey 1996 (Other DHS)
Demographic and Health Survey 2006 (Other DHS)
NMR
Year

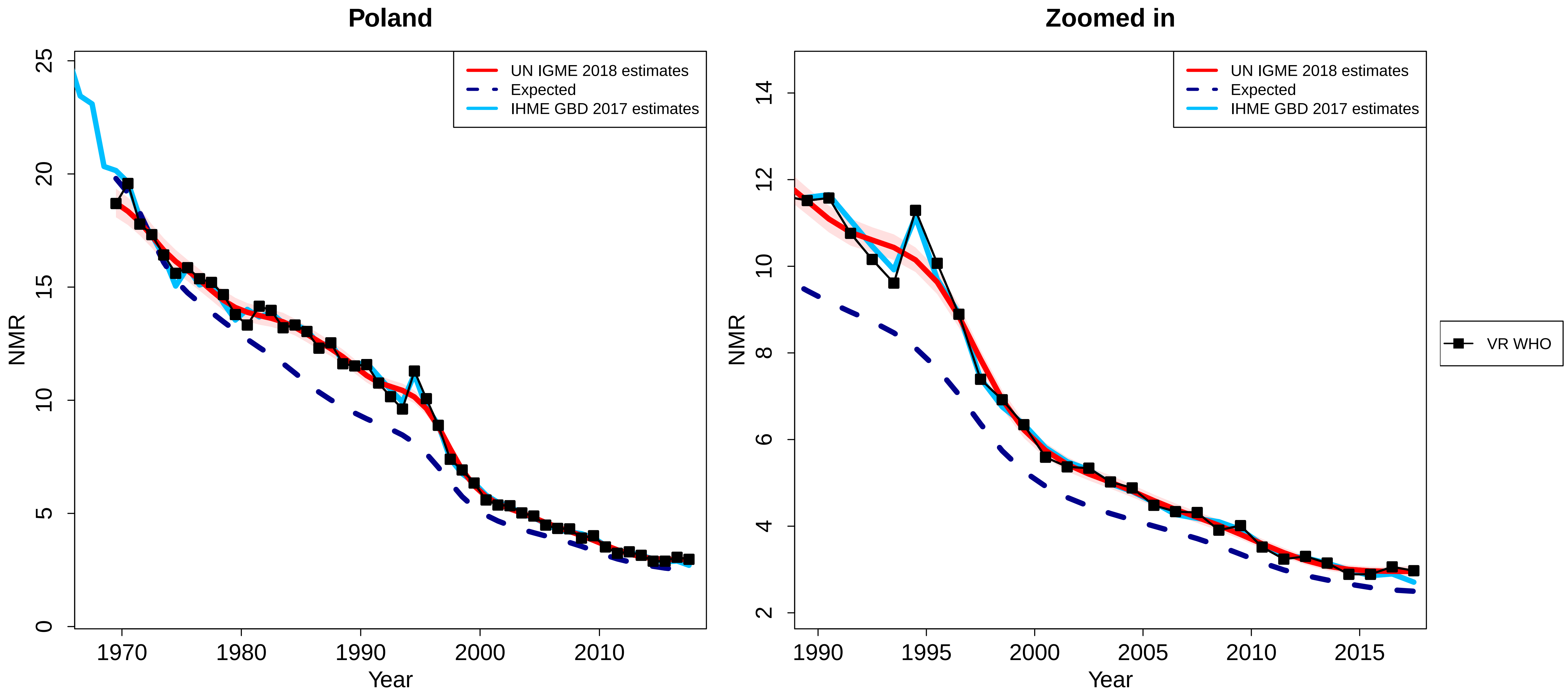
Poland
Zoomed in
UN IGME 2018 estimates
Expected
IHME GBD 2017 estimates
VR WHO
NMR
Year
0
5
10
15
20
25
1970
1980
1990
2000
2010
2
4
6
8
10
12
14
1990
1995
2000
2005
2010
2015

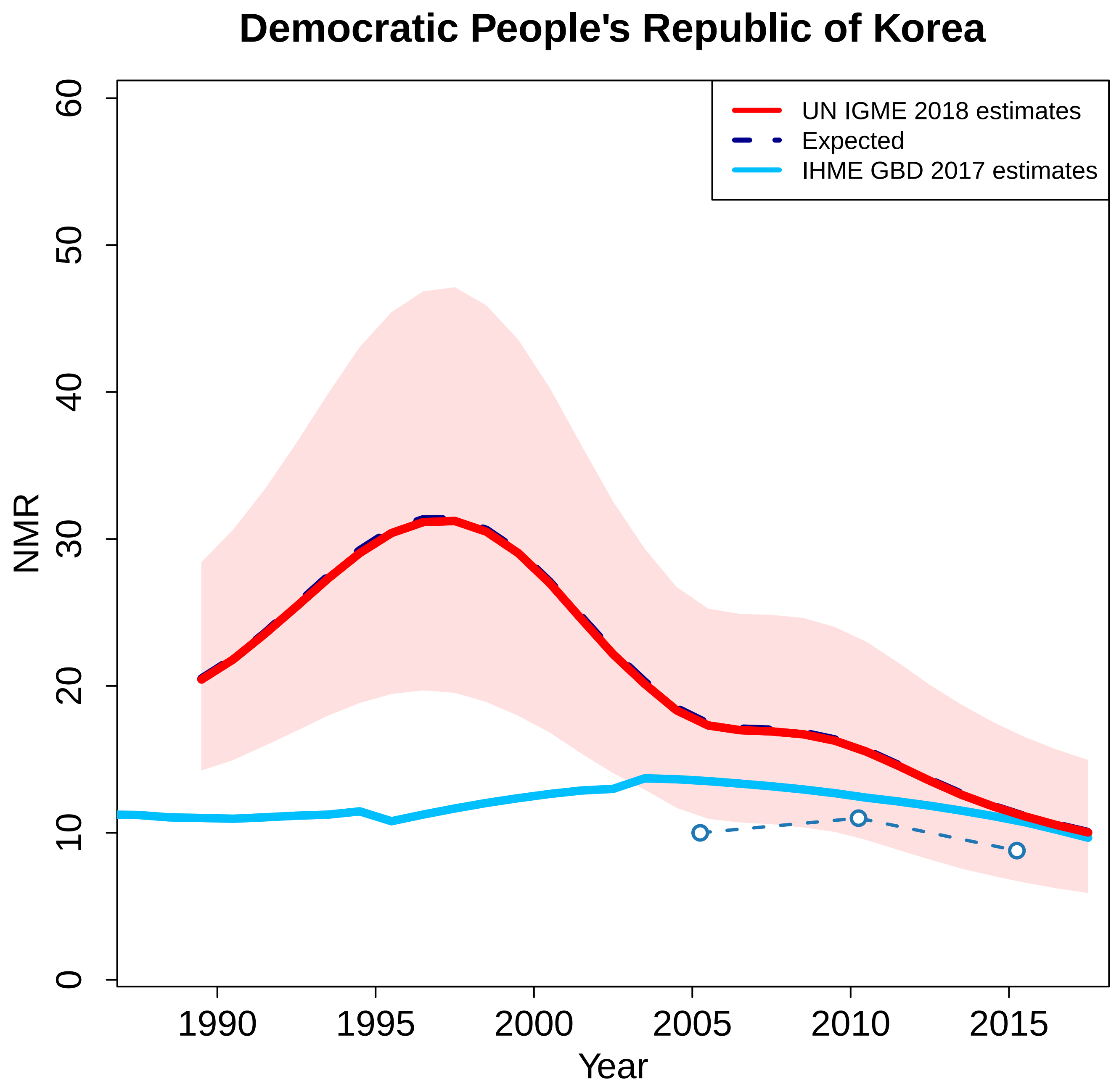

Democratic People's Republic of Korea
NMR
Year
UN IGME 2018 estimates
Expected
IHME GBD 2017 estimates

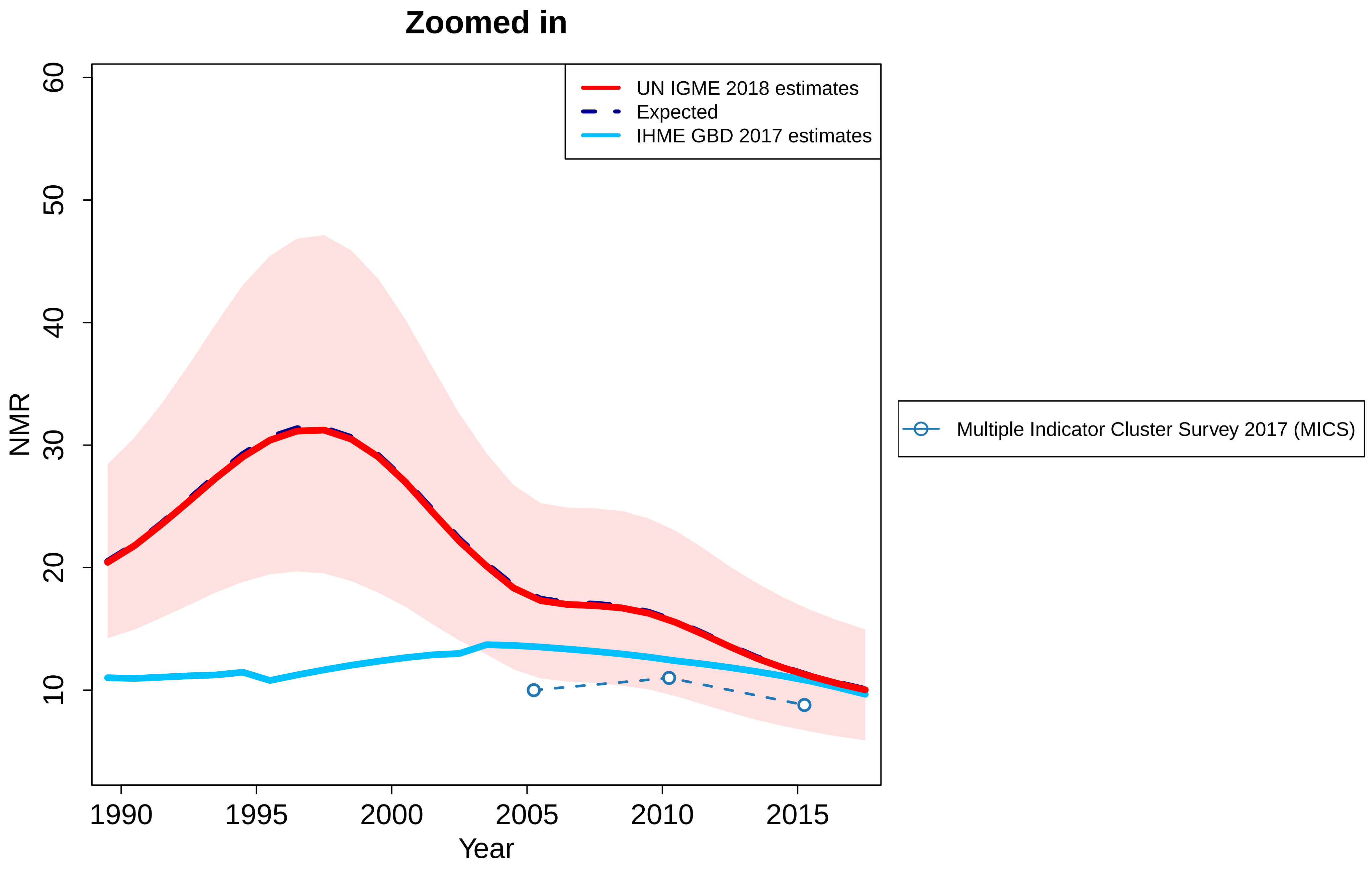

Zoomed in
NMR
Year
UN IGME 2018 estimates
Expected
IHME GBD 2017 estimates
Multiple Indicator Cluster Survey 2017 (MICS)

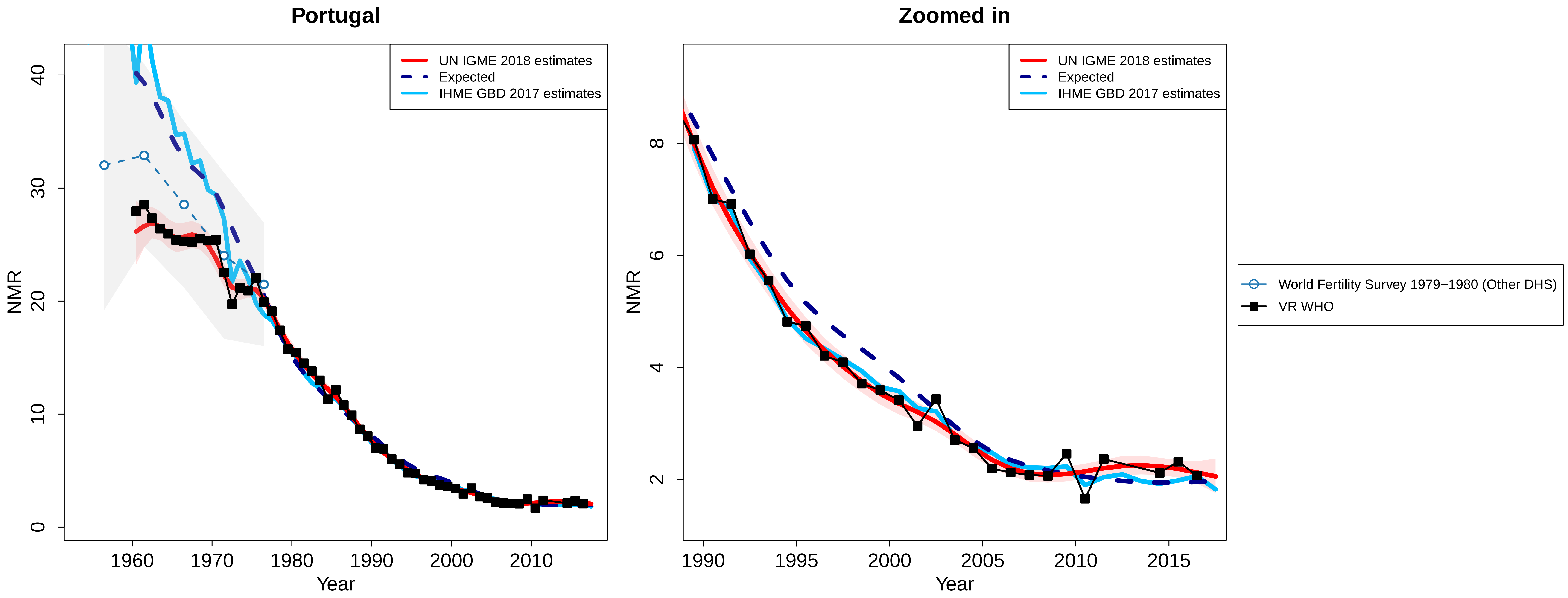
Portugal
Zoomed in
UN IGME 2018 estimates
Expected
IHME GBD 2017 estimates
World Fertility Survey 1979−1980 (Other DHS)
VR WHO
NMR
Year
0
10
20
30
40
1960
1970
1980
1990
2000
2010
2
4
6
8
1995
2005
2015

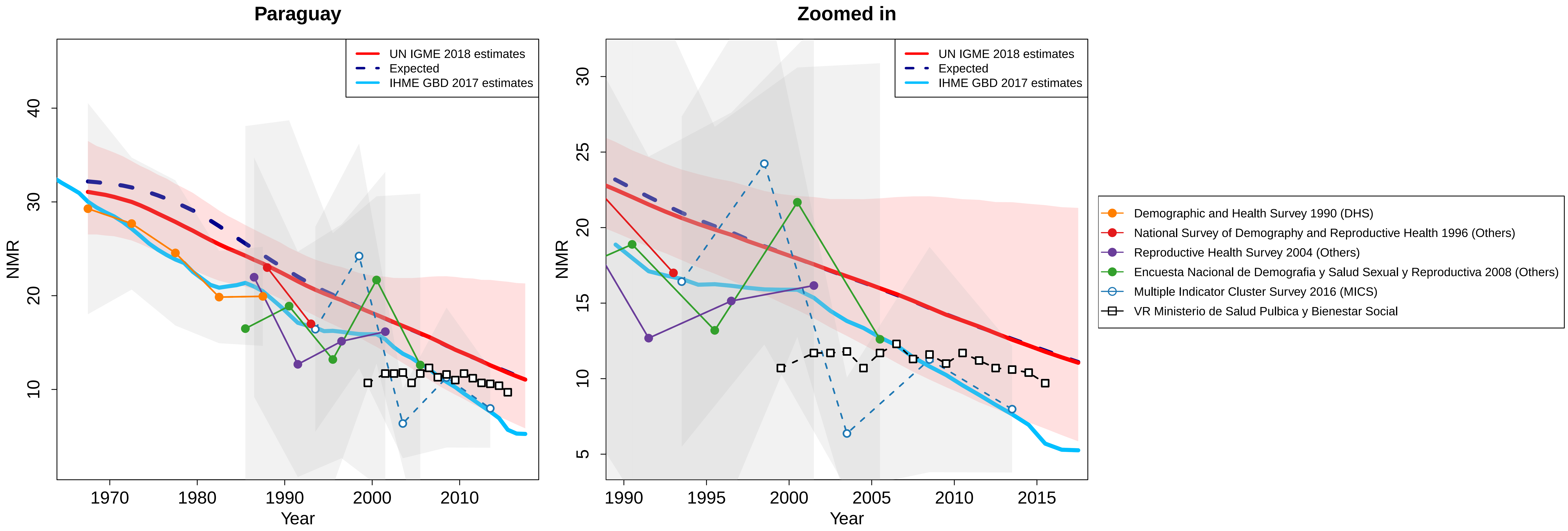
Paraguay
Zoomed in
UN IGME 2018 estimates
Expected
IHME GBD 2017 estimates
NMR
Year
Demographic and Health Survey 1990 (DHS)
National Survey of Demography and Reproductive Health 1996 (Others)
Reproductive Health Survey 2004 (Others)
Encuesta Nacional de Demografia y Salud Sexual y Reproductiva 2008 (Others)
Multiple Indicator Cluster Survey 2016 (MICS)
VR Ministerio de Salud Pulbica y Bienestar Social

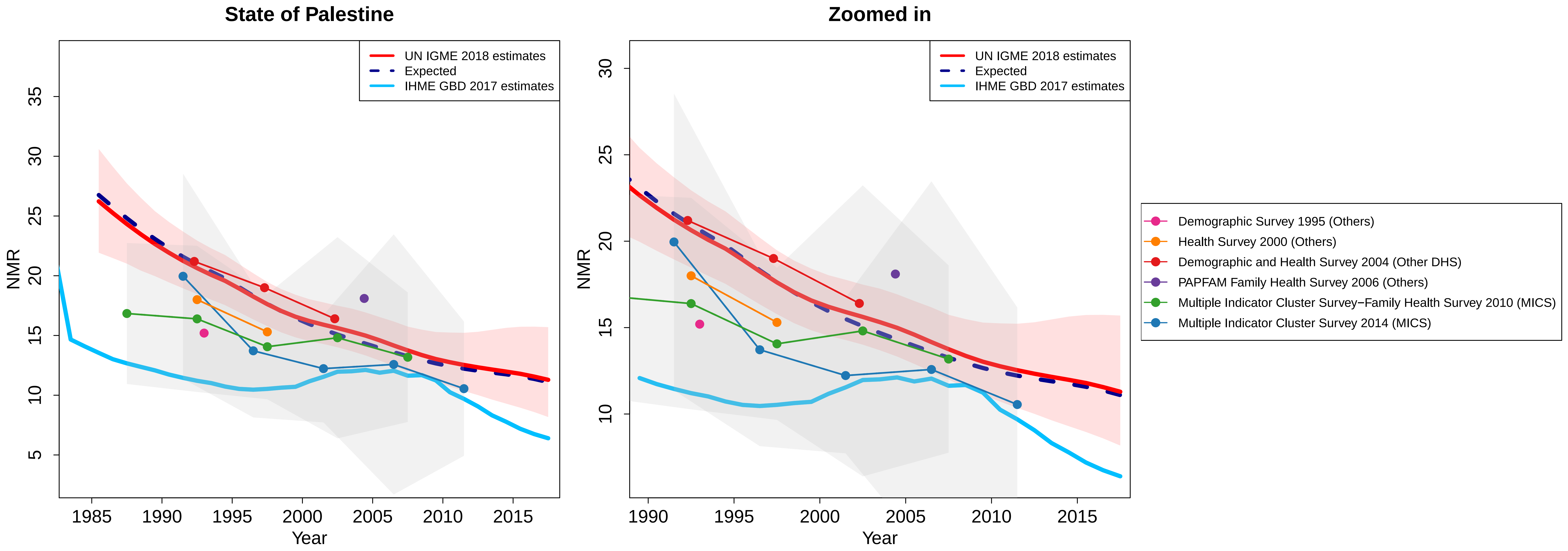
State of Palestine
Zoomed in
UN IGME 2018 estimates
Expected
IHME GBD 2017 estimates
NMR
Year
Demographic Survey 1995 (Others)
Health Survey 2000 (Others)
Demographic and Health Survey 2004 (Other DHS)
PAPFAM Family Health Survey 2006 (Others)
Multiple Indicator Cluster Survey−Family Health Survey 2010 (MICS)
Multiple Indicator Cluster Survey 2014 (MICS)

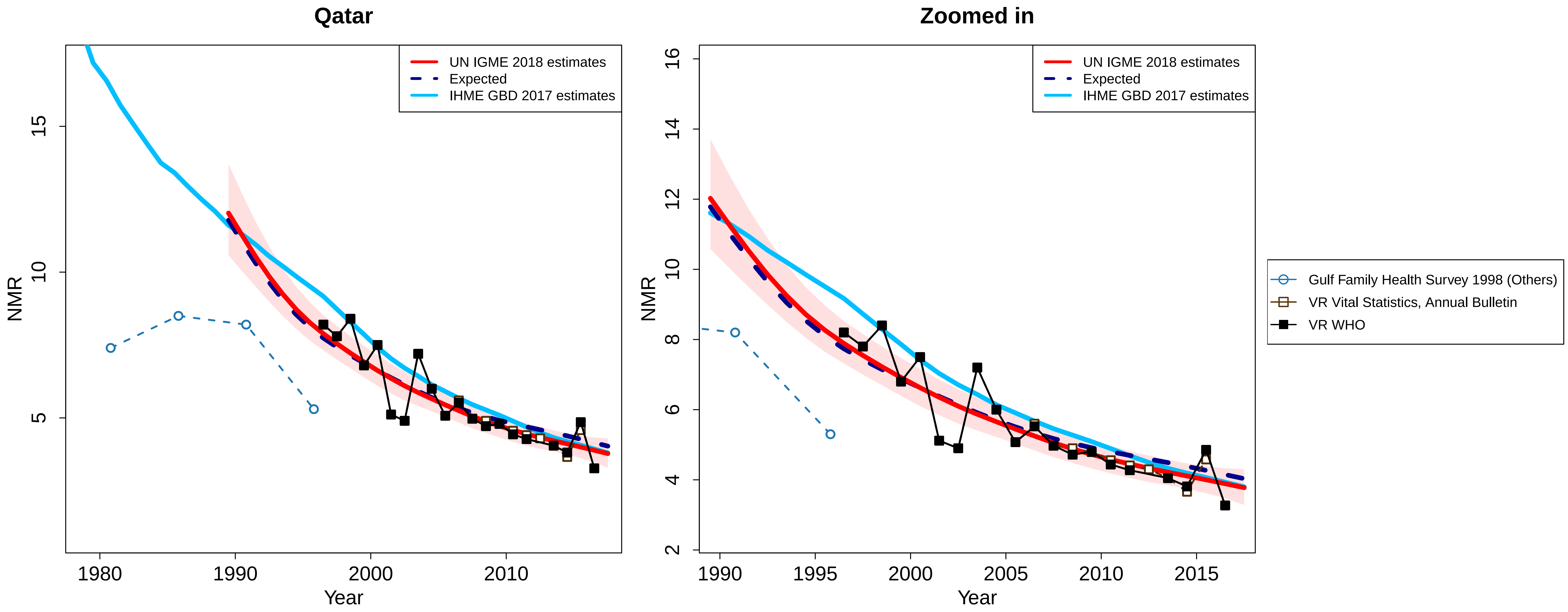
Qatar
Zoomed in
UN IGME 2018 estimates
Expected
IHME GBD 2017 estimates
Gulf Family Health Survey 1998 (Others)
VR Vital Statistics, Annual Bulletin
VR WHO
NMR
Year
1980
1990
2000
2010
1995
2005
2015
5
10
15
2
4
6
8
12
14
16

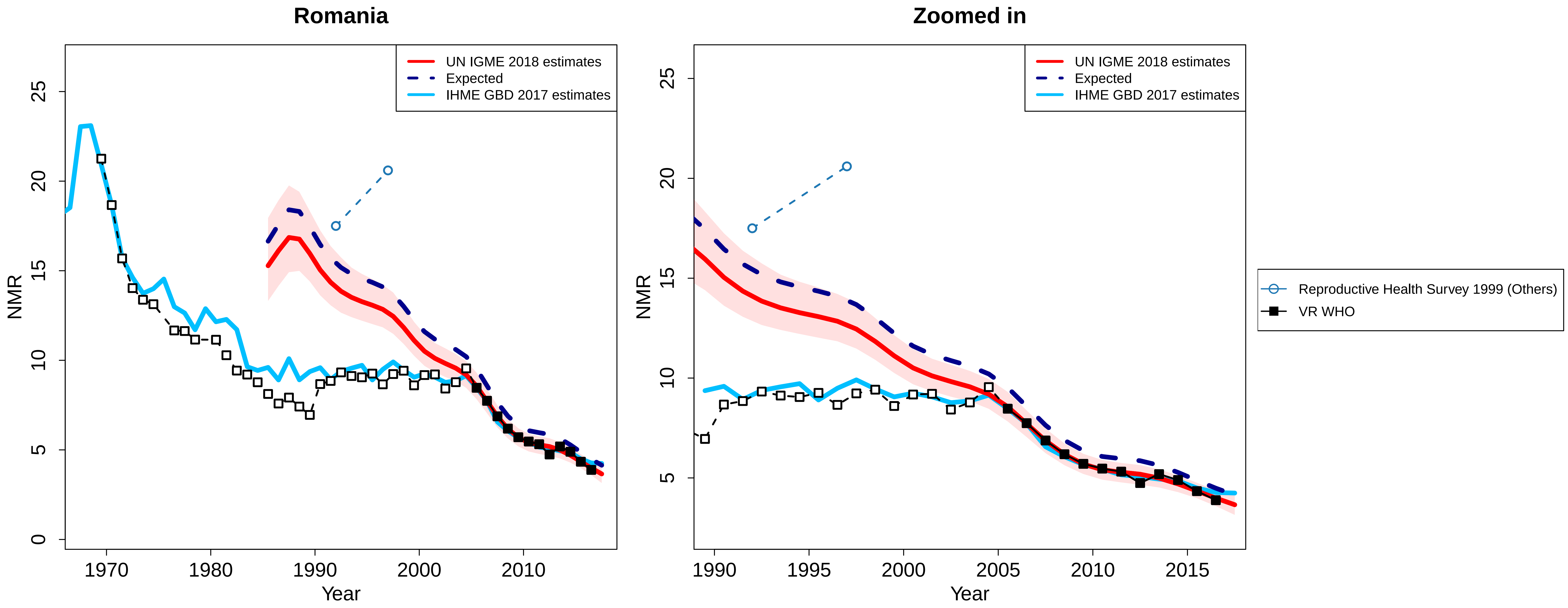
Romania
Zoomed in
UN IGME 2018 estimates
Expected
IHME GBD 2017 estimates
NMR
Year
Reproductive Health Survey 1999 (Others)
VR WHO

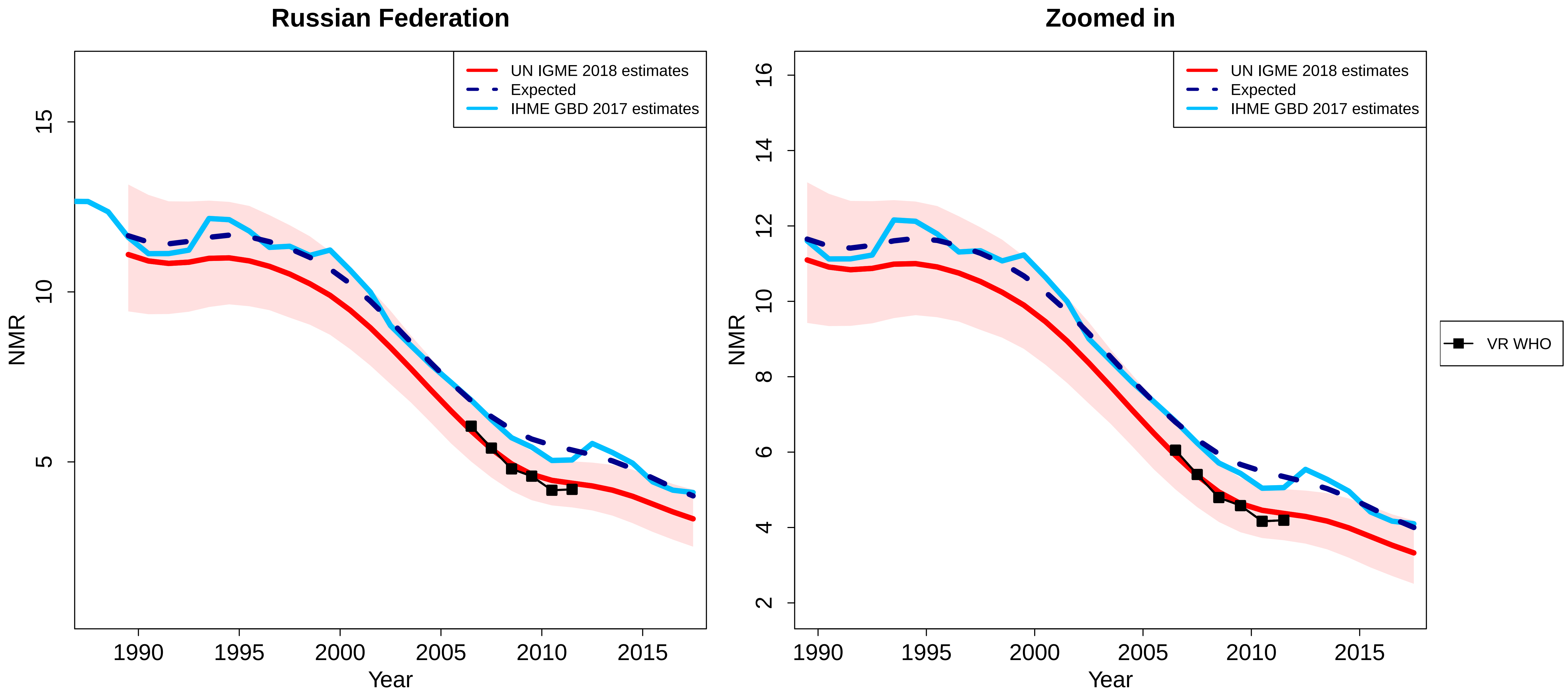
Russian Federation
Zoomed in
UN IGME 2018 estimates
Expected
IHME GBD 2017 estimates
VR WHO
NMR
Year
1990
1995
2000
2005
2010
2015
5
10
15
2
4
6
8
10
12
14
16

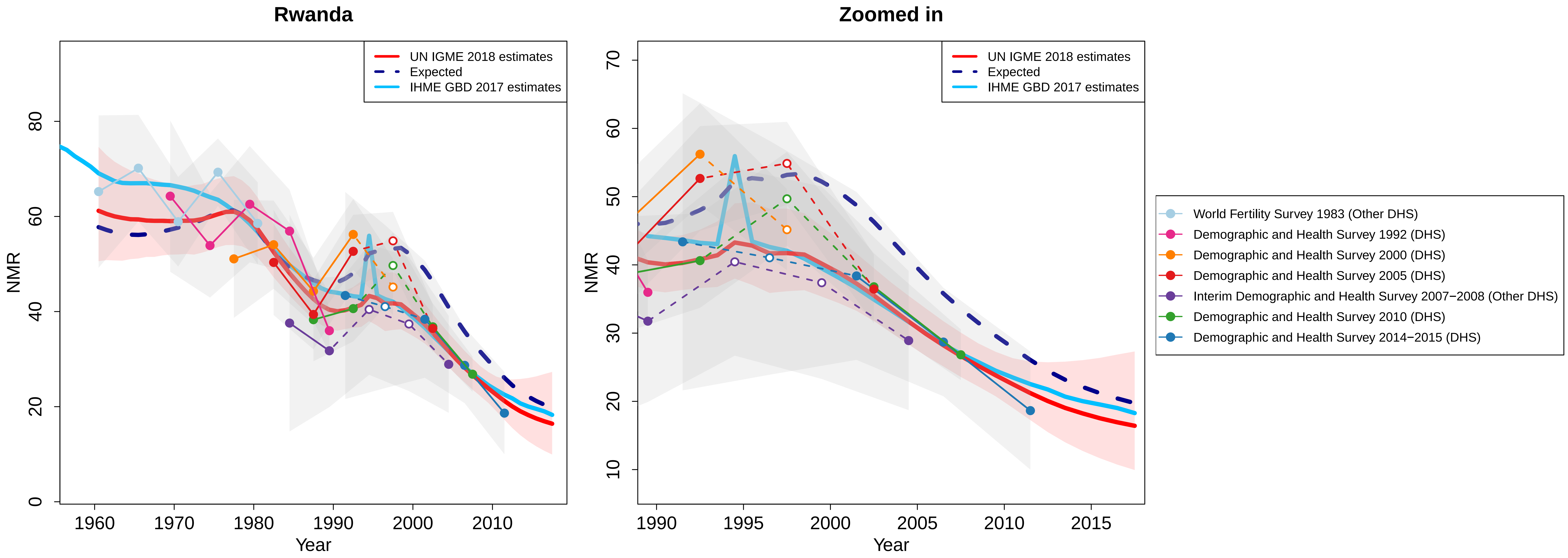
Rwanda
Zoomed in
UN IGME 2018 estimates
Expected
IHME GBD 2017 estimates
NMR
Year
World Fertility Survey 1983 (Other DHS)
Demographic and Health Survey 1992 (DHS)
Demographic and Health Survey 2000 (DHS)
Demographic and Health Survey 2005 (DHS)
Interim Demographic and Health Survey 2007–2008 (Other DHS)
Demographic and Health Survey 2010 (DHS)
Demographic and Health Survey 2014–2015 (DHS)

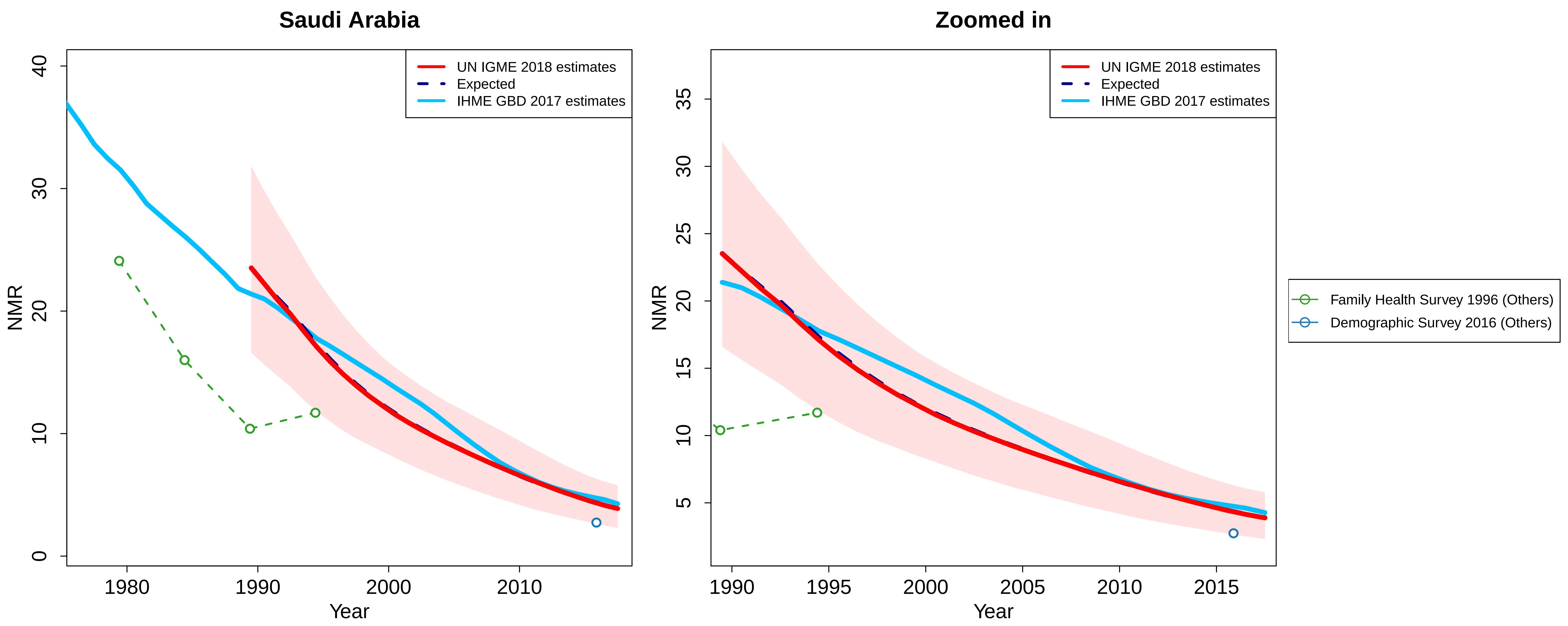

Saudi Arabia
Zoomed in
UN IGME 2018 estimates
Expected
IHME GBD 2017 estimates
Family Health Survey 1996 (Others)
Demographic Survey 2016 (Others)
NMR
Year

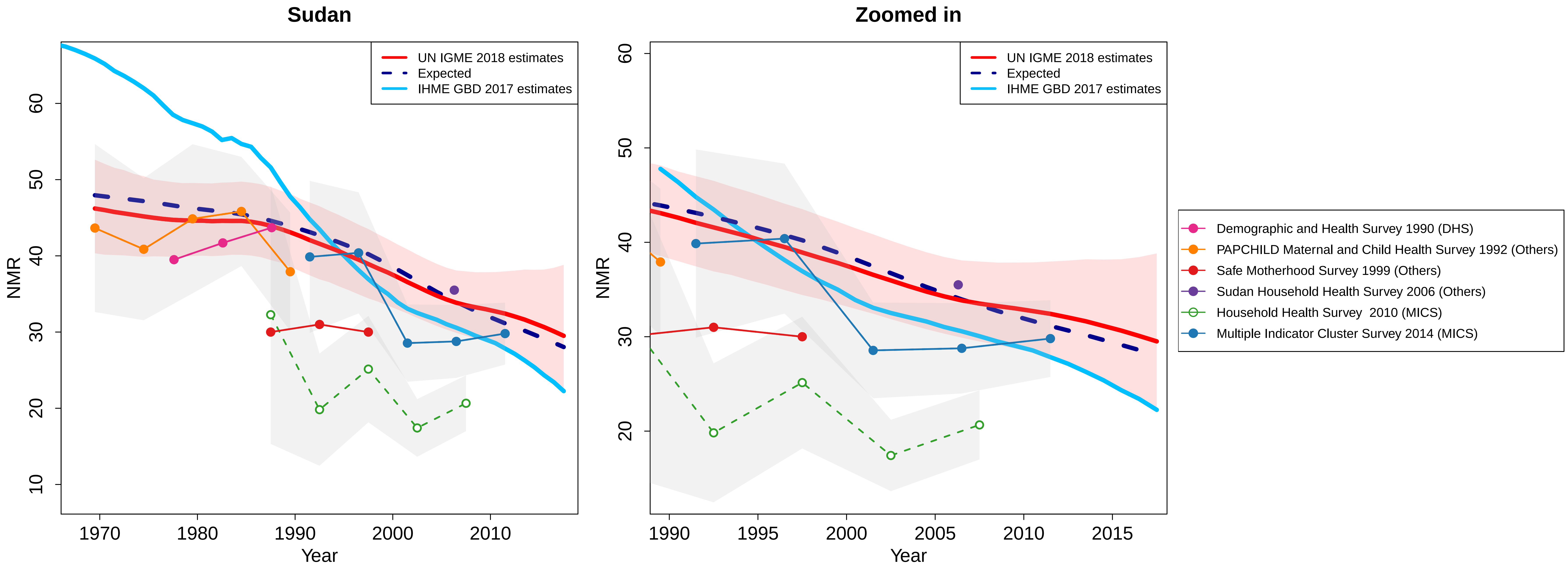

Sudan
Zoomed in
NMR
Year
UN IGME 2018 estimates
Expected
IHME GBD 2017 estimates
Demographic and Health Survey 1990 (DHS)
PAPCHILD Maternal and Child Health Survey 1992 (Others)
Safe Motherhood Survey 1999 (Others)
Sudan Household Health Survey 2006 (Others)
Household Health Survey 2010 (MICS)
Multiple Indicator Cluster Survey 2014 (MICS)

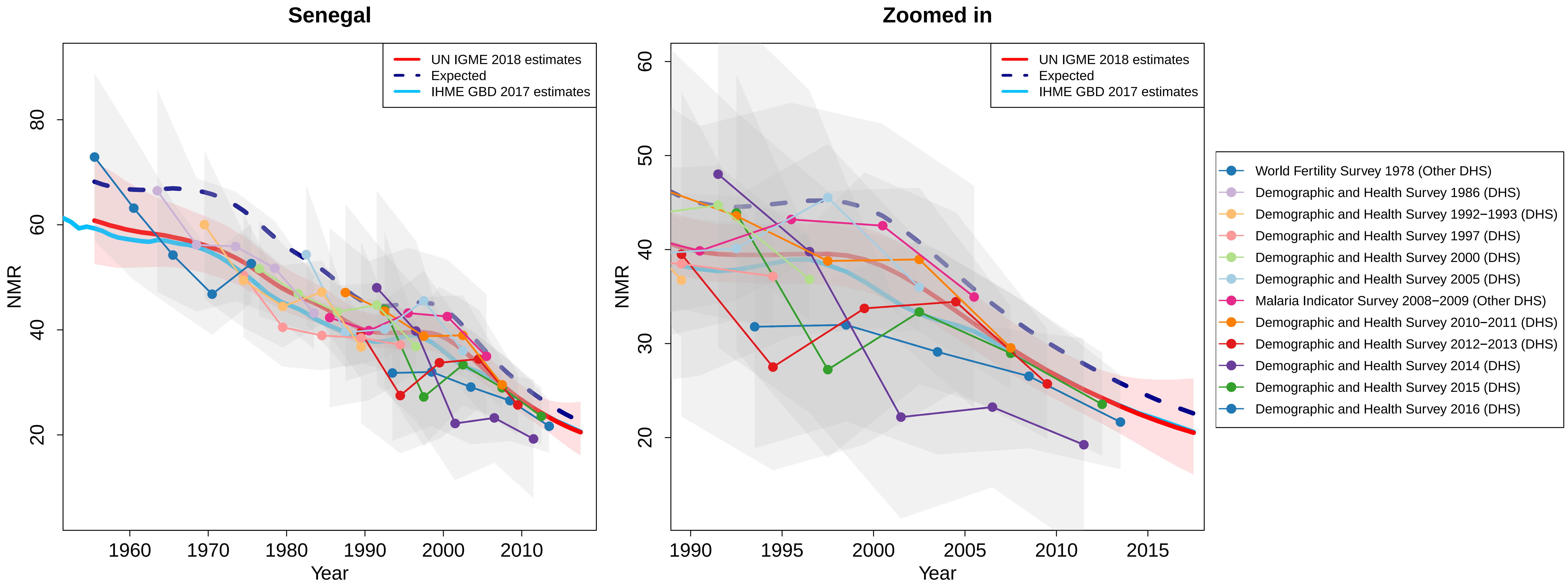

Senegal
Zoomed in
UN IGME 2018 estimates
Expected
IHME GBD 2017 estimates
NMR
Year
World Fertility Survey 1978 (Other DHS)
Demographic and Health Survey 1986 (DHS)
Demographic and Health Survey 1992−1993 (DHS)
Demographic and Health Survey 1997 (DHS)
Demographic and Health Survey 2000 (DHS)
Demographic and Health Survey 2005 (DHS)
Malaria Indicator Survey 2008−2009 (Other DHS)
Demographic and Health Survey 2010−2011 (DHS)
Demographic and Health Survey 2012−2013 (DHS)
Demographic and Health Survey 2014 (DHS)
Demographic and Health Survey 2015 (DHS)
Demographic and Health Survey 2016 (DHS)

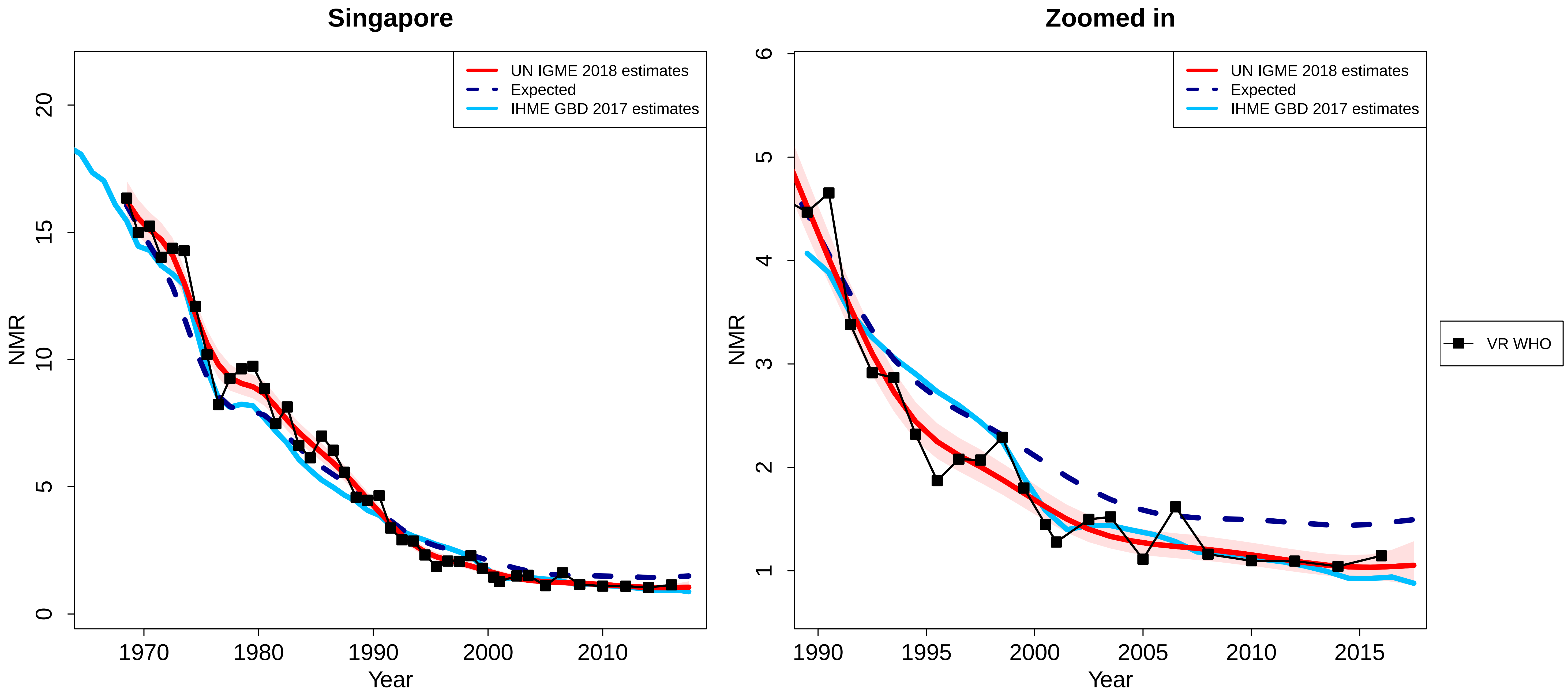

Singapore
Zoomed in
UN IGME 2018 estimates
Expected
IHME GBD 2017 estimates
VR WHO
NMR
Year
0
5
10
15
20
1970
1980
1990
2000
2010
1
2
3
4
5
6
1990
1995
2000
2005
2010
2015

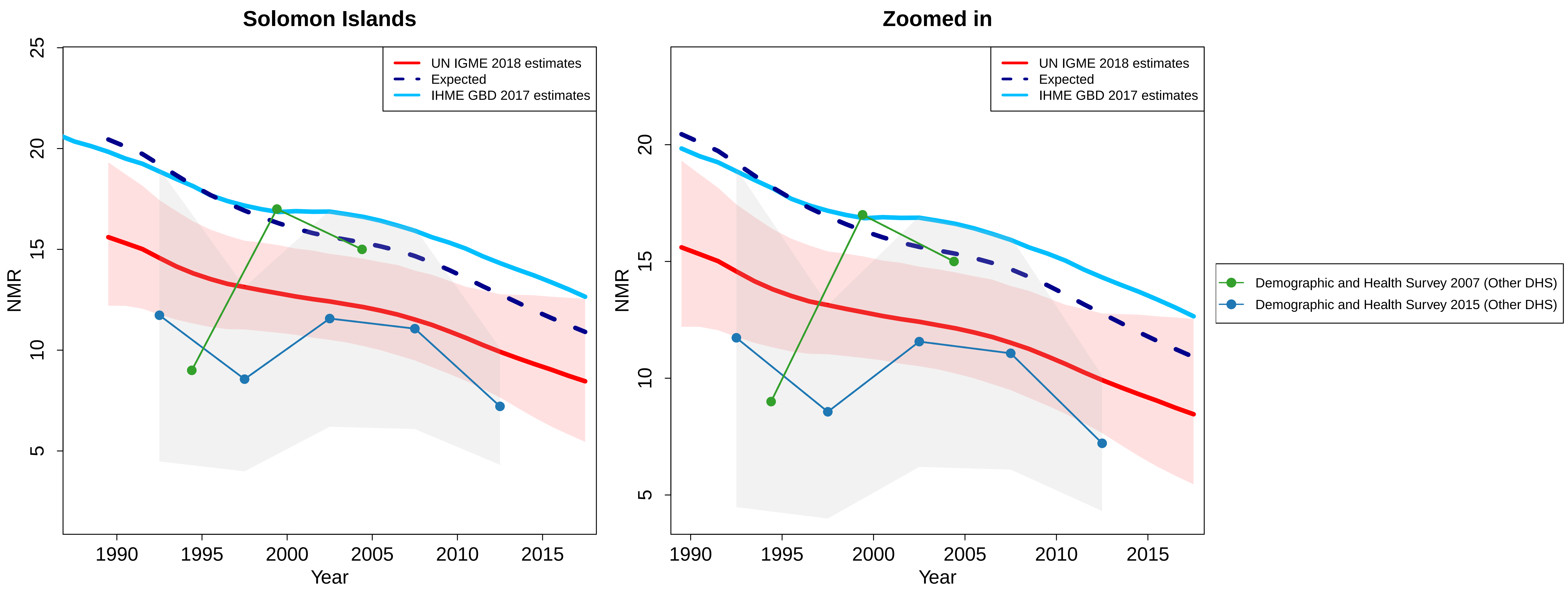

Solomon Islands
Zoomed in
UN IGME 2018 estimates
Expected
IHME GBD 2017 estimates
Demographic and Health Survey 2007 (Other DHS)
Demographic and Health Survey 2015 (Other DHS)
NMR
Year
1990
1995
2000
2005
2010
2015
5
10
15
20
25

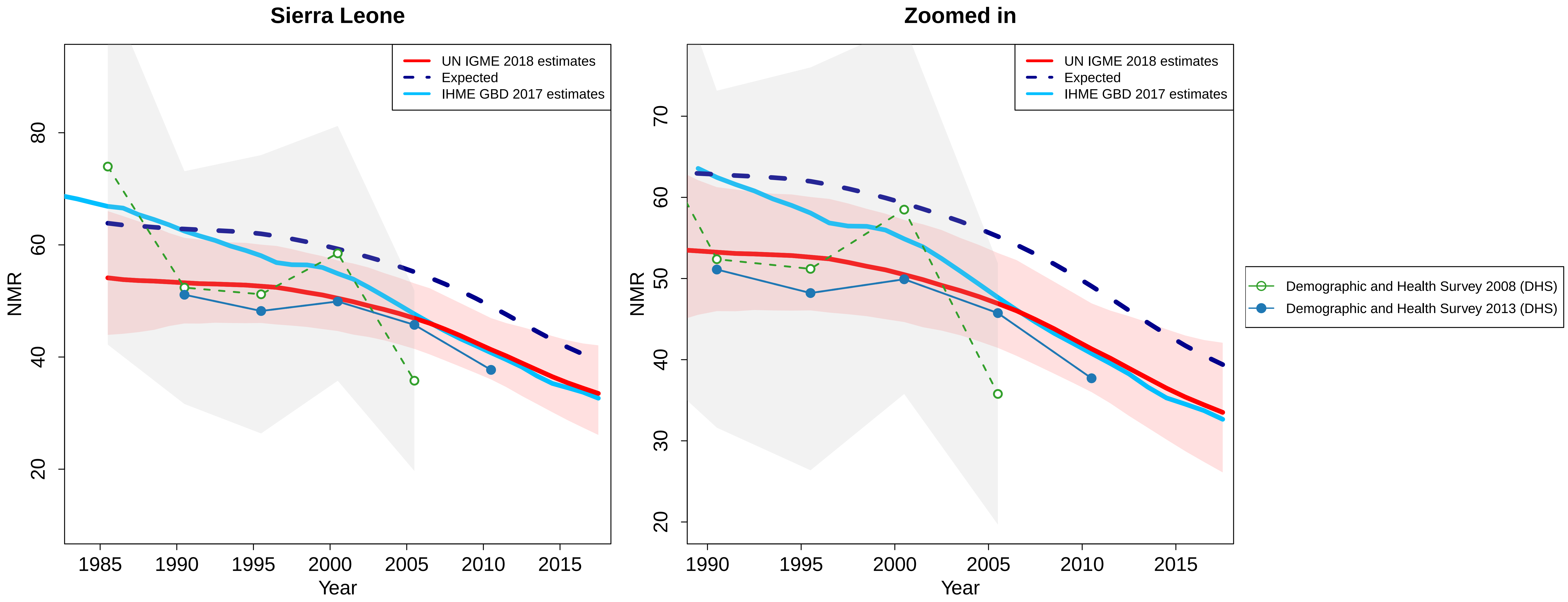

Sierra Leone
Zoomed in
UN IGME 2018 estimates
Expected
IHME GBD 2017 estimates
Demographic and Health Survey 2008 (DHS)
Demographic and Health Survey 2013 (DHS)
NMR
Year
20
40
60
80
1985
1990
1995
2000
2005
2010
2015
30
50
70

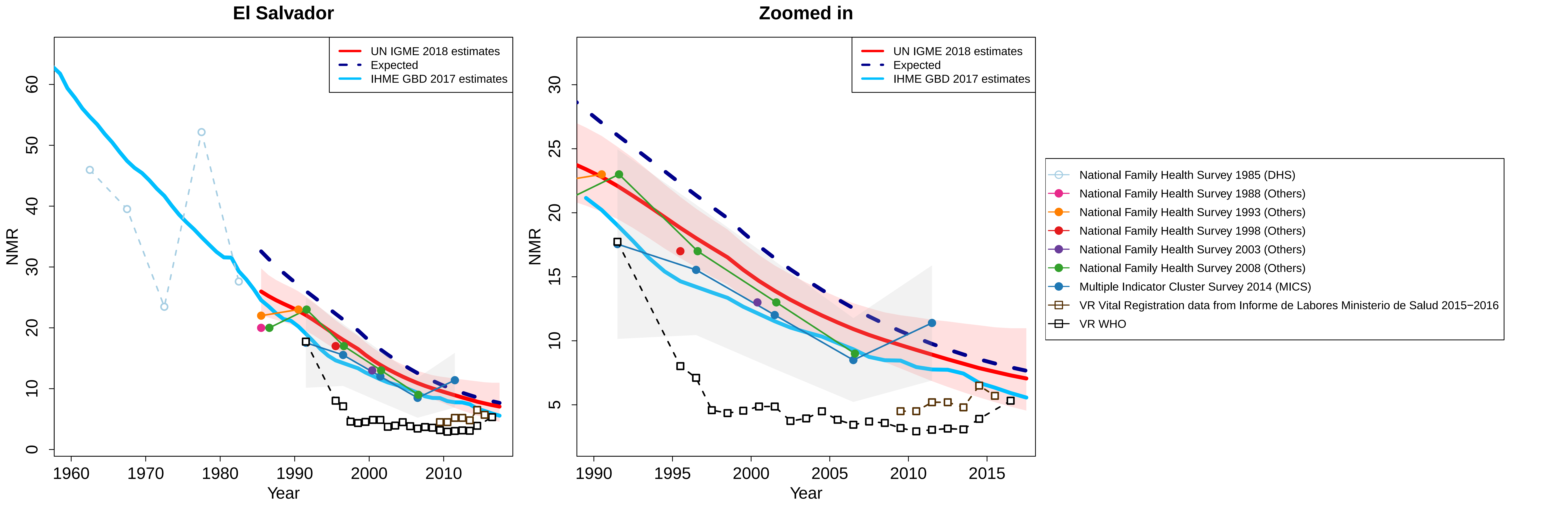

El Salvador
Zoomed in
UN IGME 2018 estimates
Expected
IHME GBD 2017 estimates
NMR
Year
National Family Health Survey 1985 (DHS)
National Family Health Survey 1988 (Others)
National Family Health Survey 1993 (Others)
National Family Health Survey 1998 (Others)
National Family Health Survey 2003 (Others)
National Family Health Survey 2008 (Others)
Multiple Indicator Cluster Survey 2014 (MICS)
VR Vital Registration data from Informe de Labores Ministerio de Salud 2015−2016
VR WHO

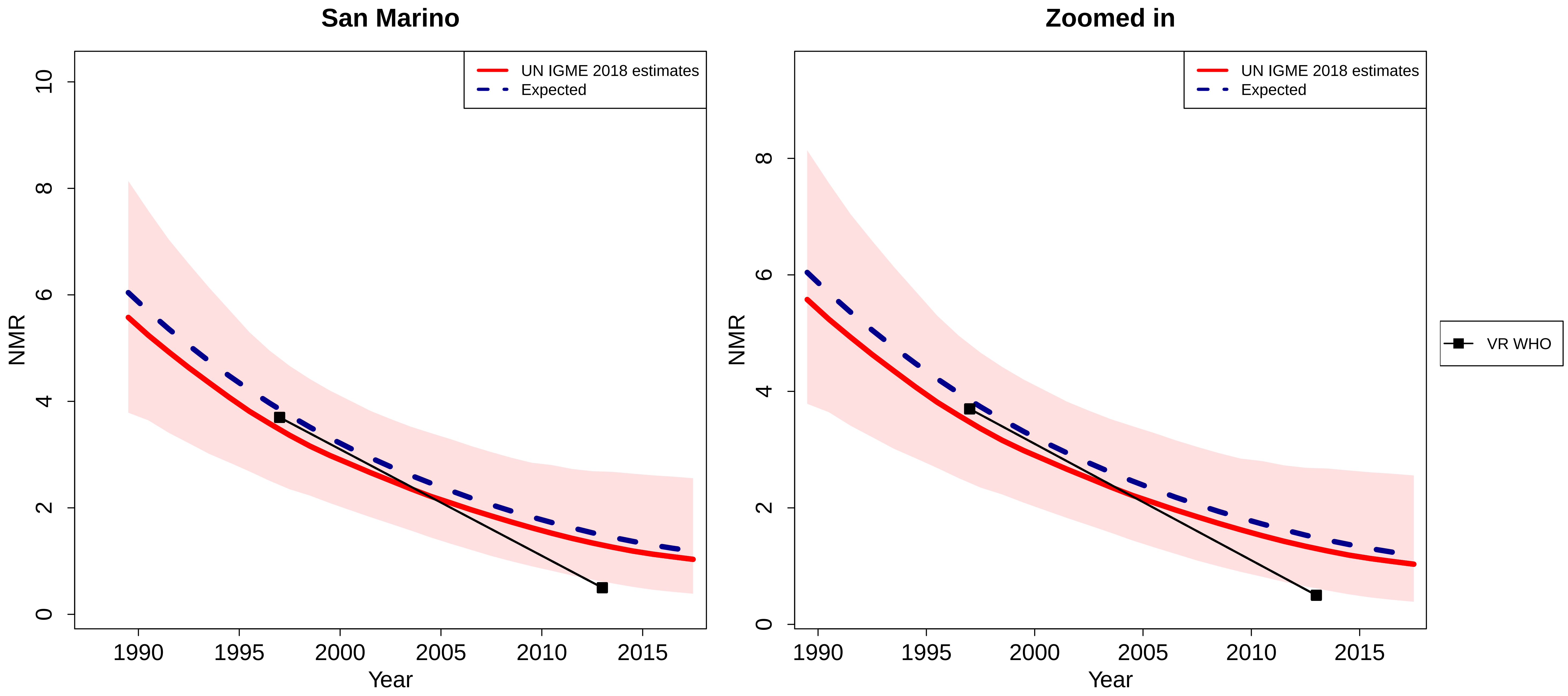

San Marino
Zoomed in
UN IGME 2018 estimates
Expected
VR WHO
NMR
Year
0
2
4
6
8
10
1990
1995
2000
2005
2010
2015

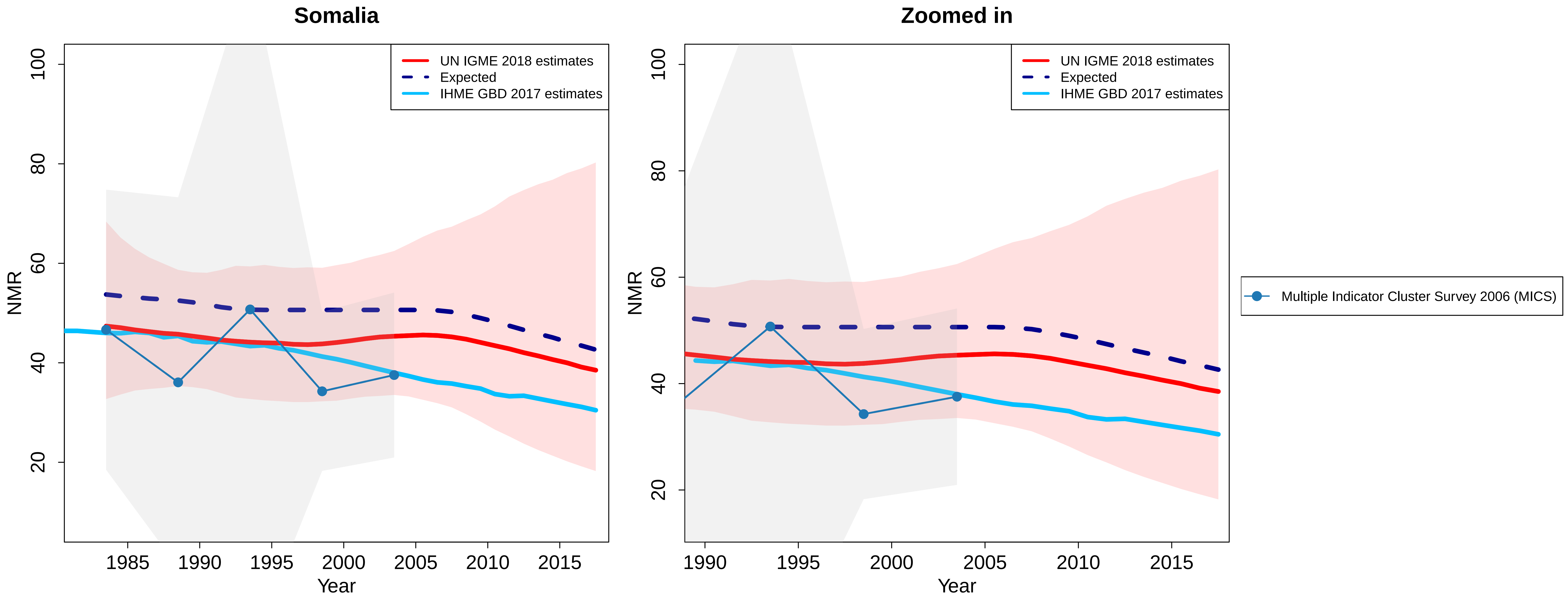
Somalia
Zoomed in
NMR
Year
UN IGME 2018 estimates
Expected
IHME GBD 2017 estimates
Multiple Indicator Cluster Survey 2006 (MICS)
1985
1990
1995
2000
2005
2010
2015
20
40
60
80
100

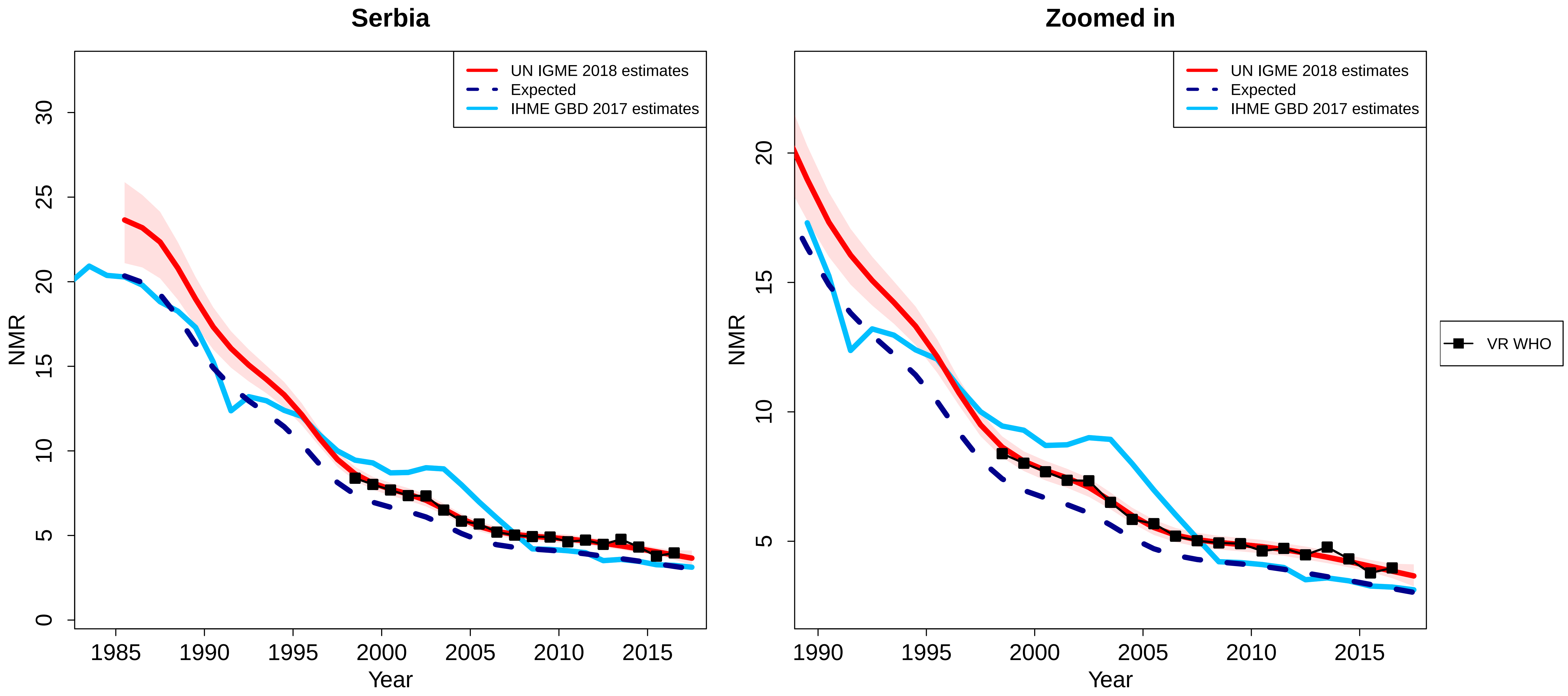
Serbia
Zoomed in
UN IGME 2018 estimates
Expected
IHME GBD 2017 estimates
VR WHO
NMR
Year
0
5
10
15
20
25
30
1985
1990
1995
2000
2005
2010
2015

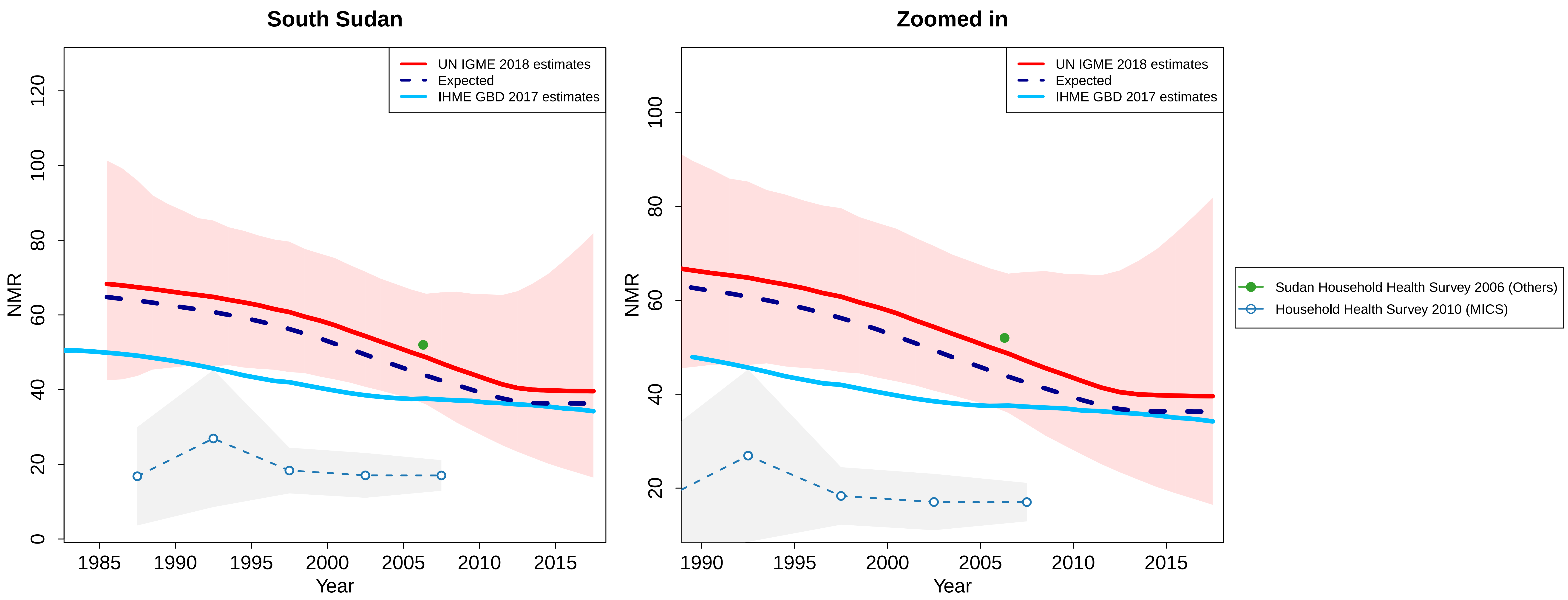
South Sudan
Zoomed in
UN IGME 2018 estimates
Expected
IHME GBD 2017 estimates
NMR
Year
0
20
40
60
80
100
120
1985
1990
1995
2000
2005
2010
2015
Sudan Household Health Survey 2006 (Others)
Household Health Survey 2010 (MICS)

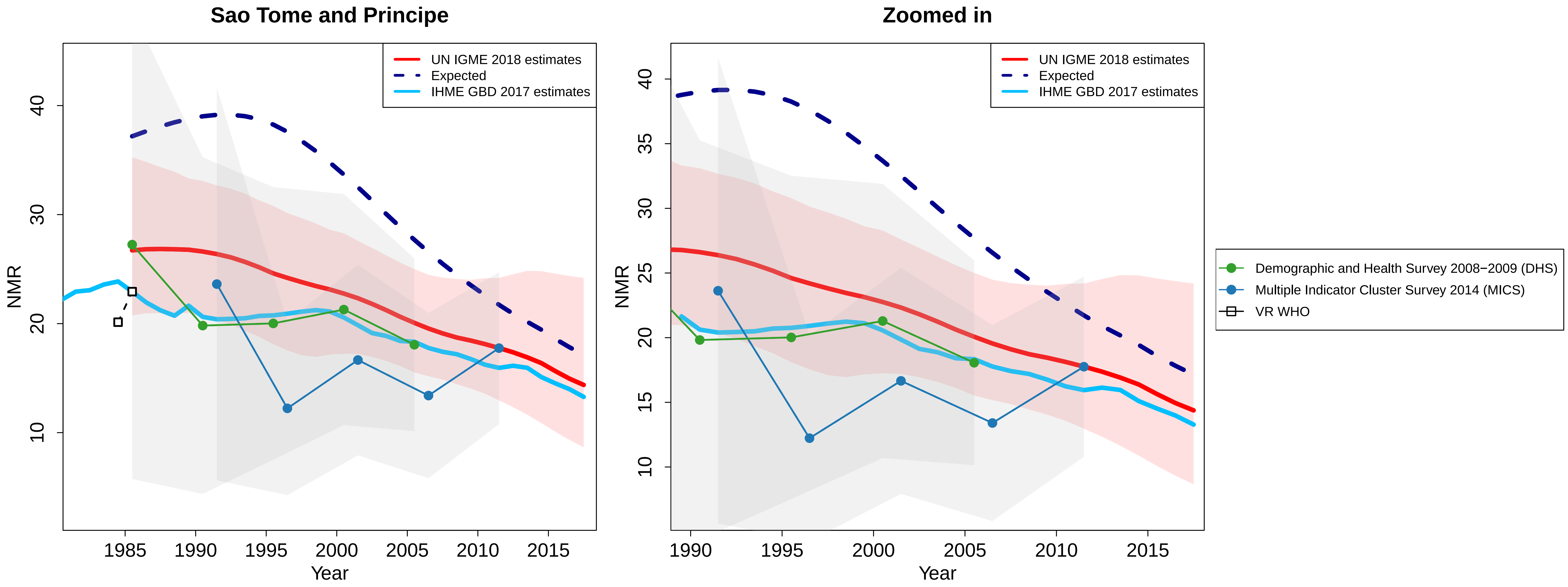
Sao Tome and Principe
Zoomed in
UN IGME 2018 estimates
Expected
IHME GBD 2017 estimates
NMR
Year
Demographic and Health Survey 2008−2009 (DHS)
Multiple Indicator Cluster Survey 2014 (MICS)
VR WHO

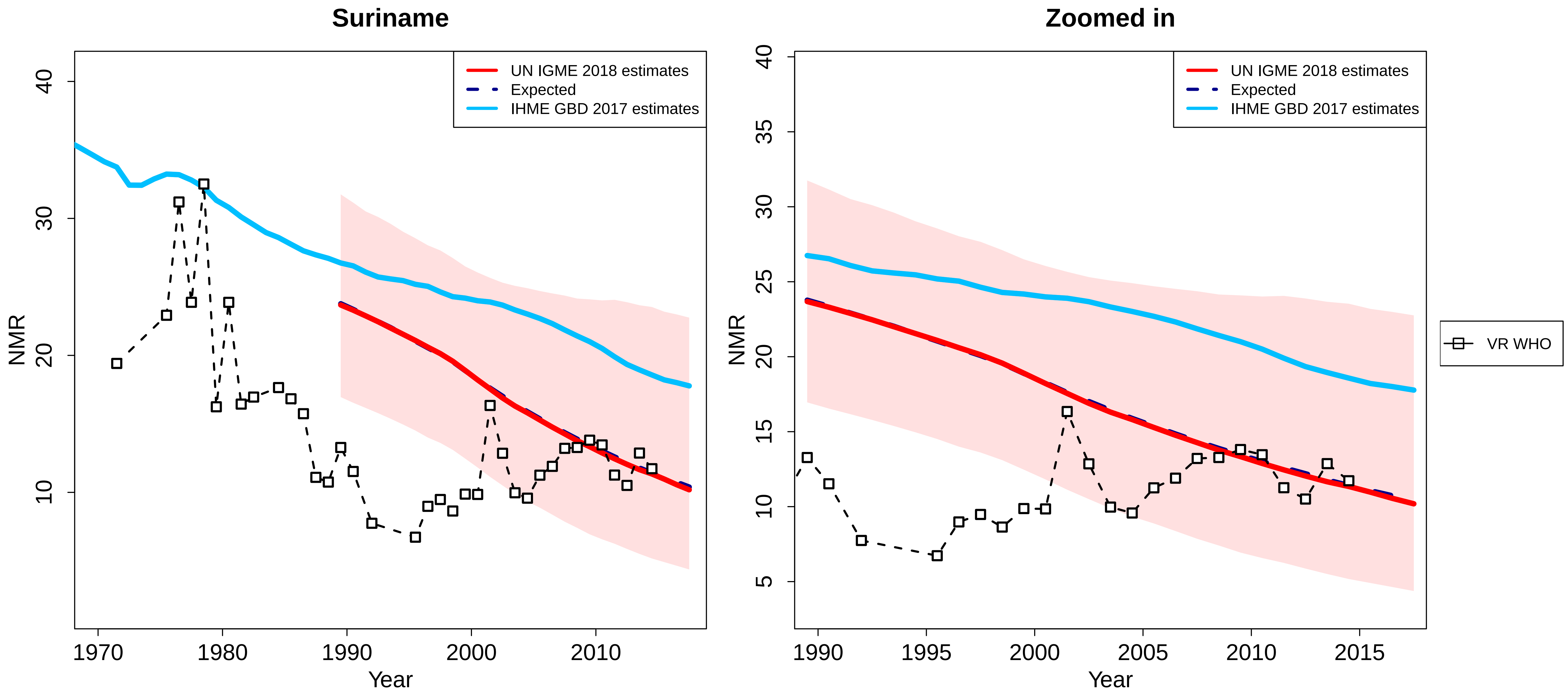

Suriname
Zoomed in
UN IGME 2018 estimates
Expected
IHME GBD 2017 estimates
VR WHO
NMR
Year

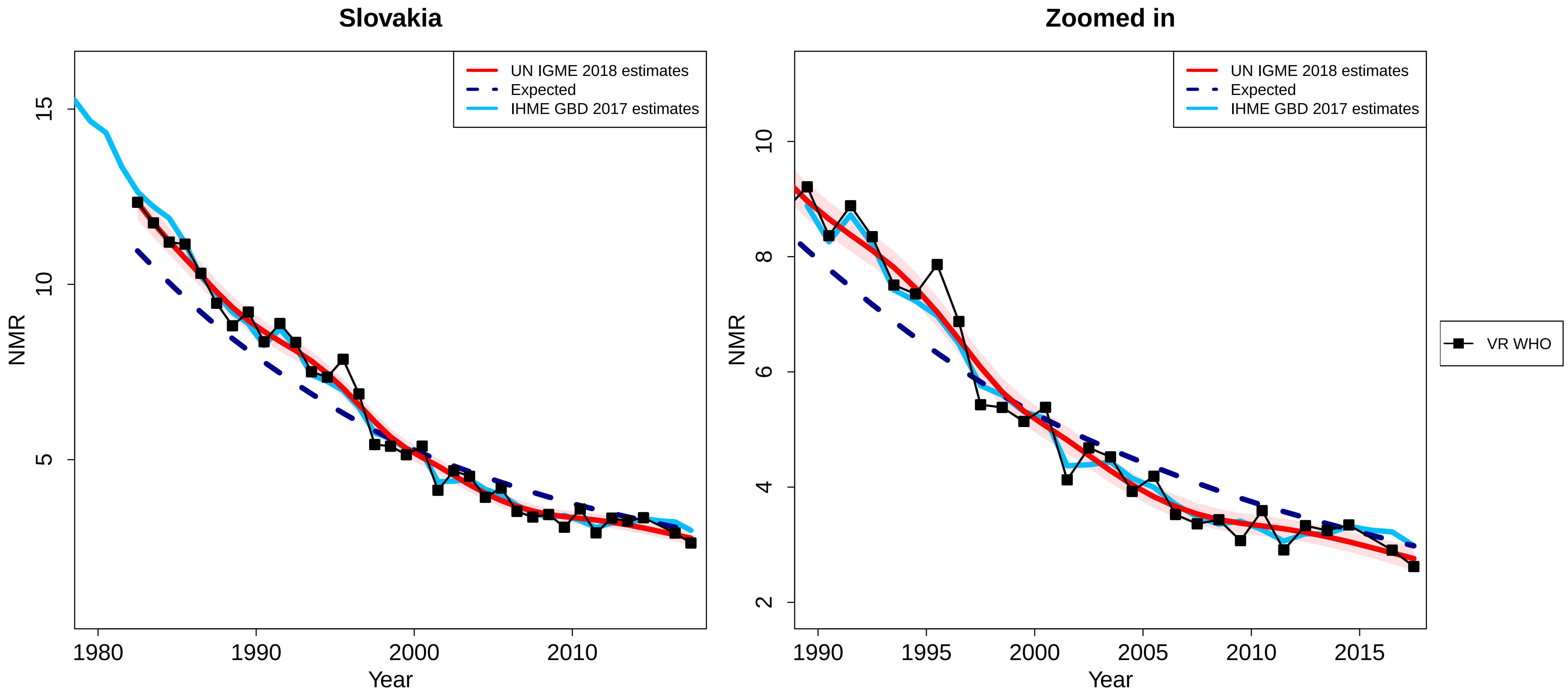
Slovakia
Zoomed in
UN IGME 2018 estimates
Expected
IHME GBD 2017 estimates
VR WHO
NMR
Year
1980
1990
2000
2010
1995
2005
2015
5
10
15
2
4
6
8

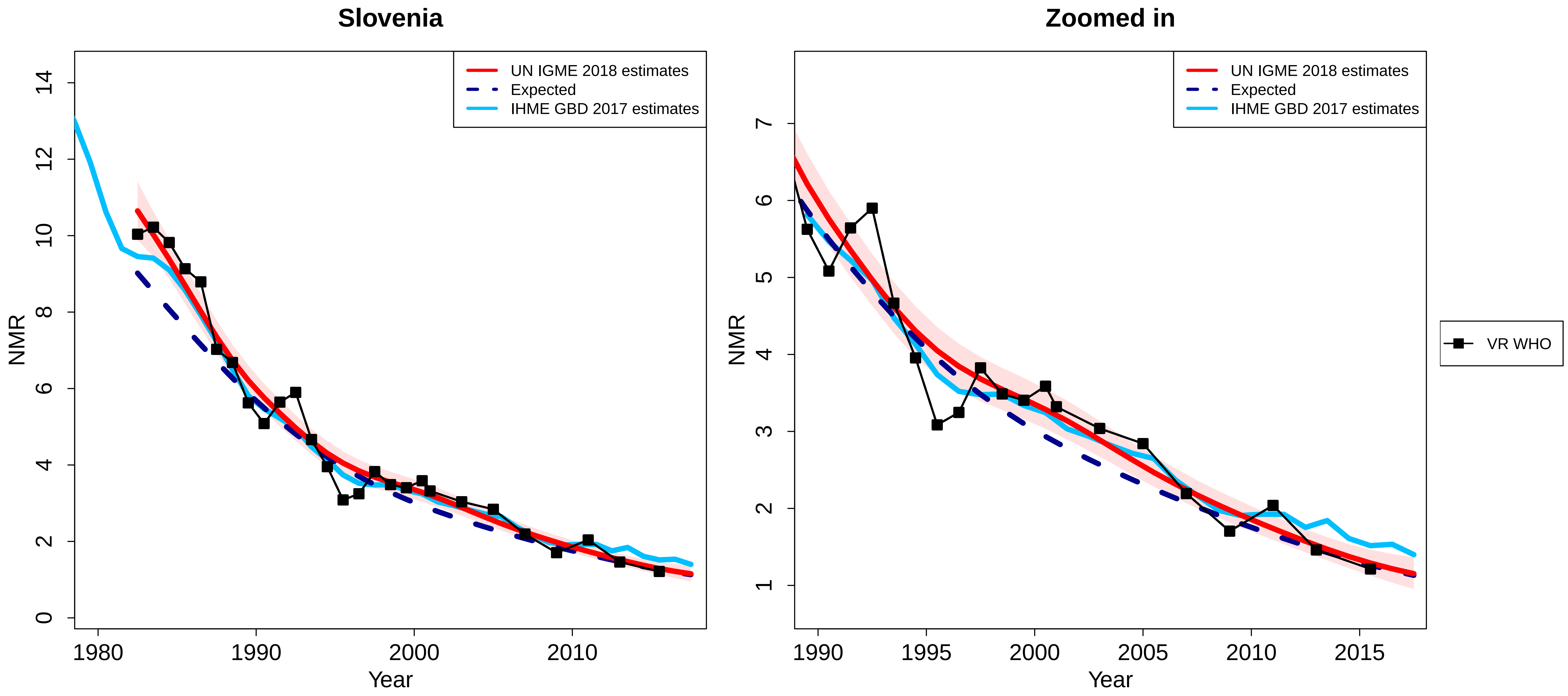

Slovenia
Zoomed in
UN IGME 2018 estimates
Expected
IHME GBD 2017 estimates
VR WHO
NMR
Year
0
2
4
6
8
10
12
14
1
2
3
4
5
6
7
1980
1990
2000
2010
1995
2005
2015

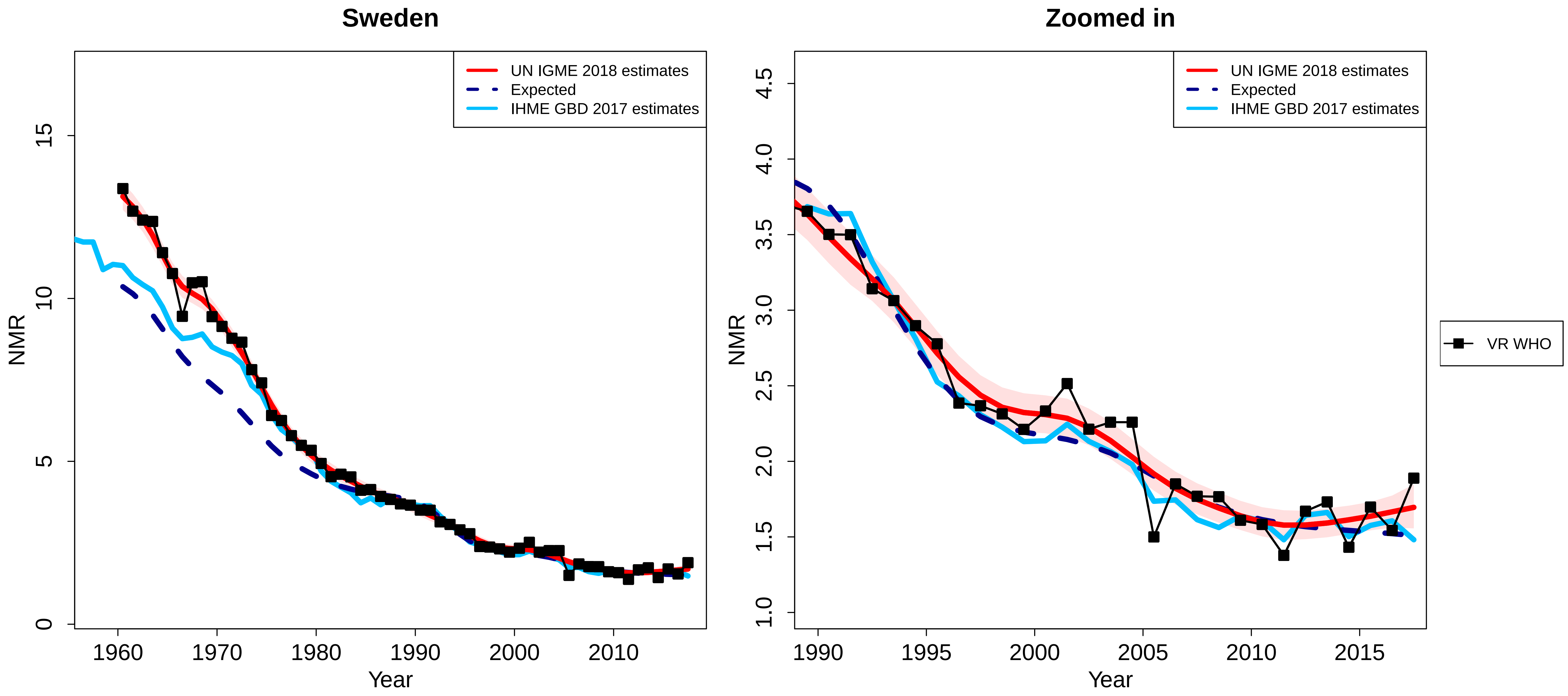

Sweden
Zoomed in
UN IGME 2018 estimates
Expected
IHME GBD 2017 estimates
VR WHO
NMR
Year
0
5
10
15
1.0
1.5
2.0
2.5
3.0
3.5
4.0
4.5
1960
1970
1980
1990
2000
2010
1990
1995
2000
2005
2010
2015

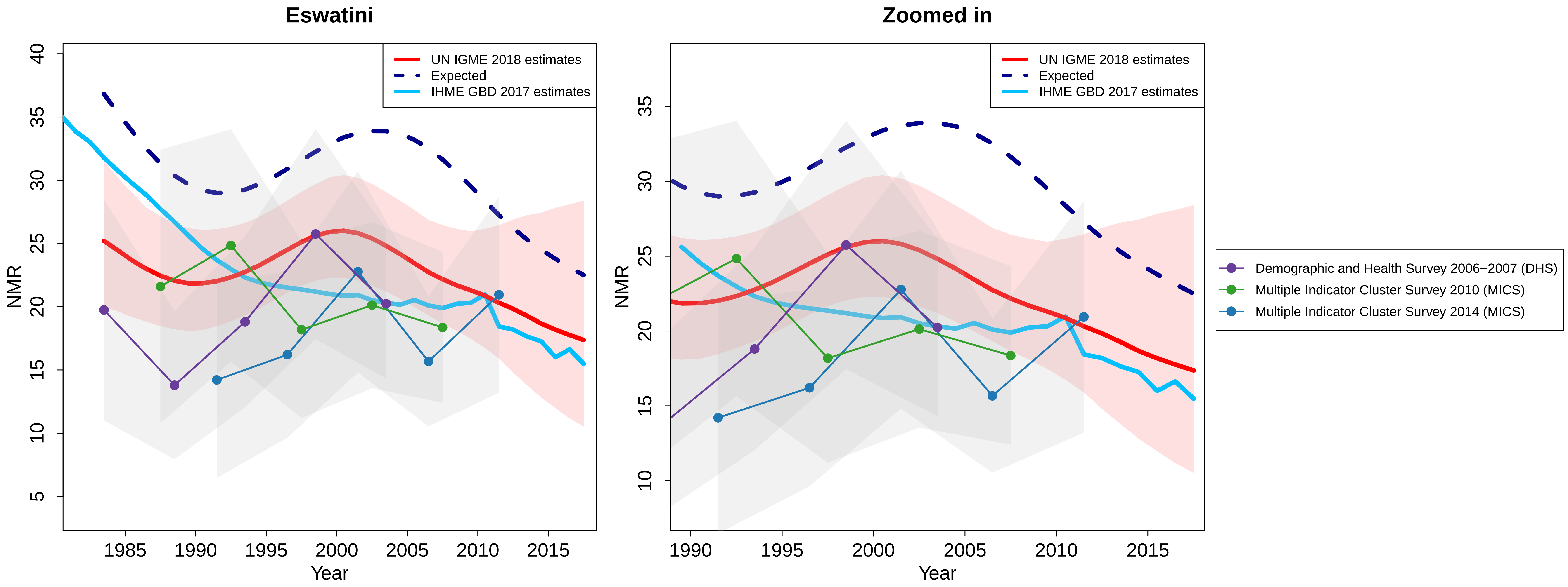

Eswatini
Zoomed in
UN IGME 2018 estimates
Expected
IHME GBD 2017 estimates
NMR
Year
Demographic and Health Survey 2006−2007 (DHS)
Multiple Indicator Cluster Survey 2010 (MICS)
Multiple Indicator Cluster Survey 2014 (MICS)

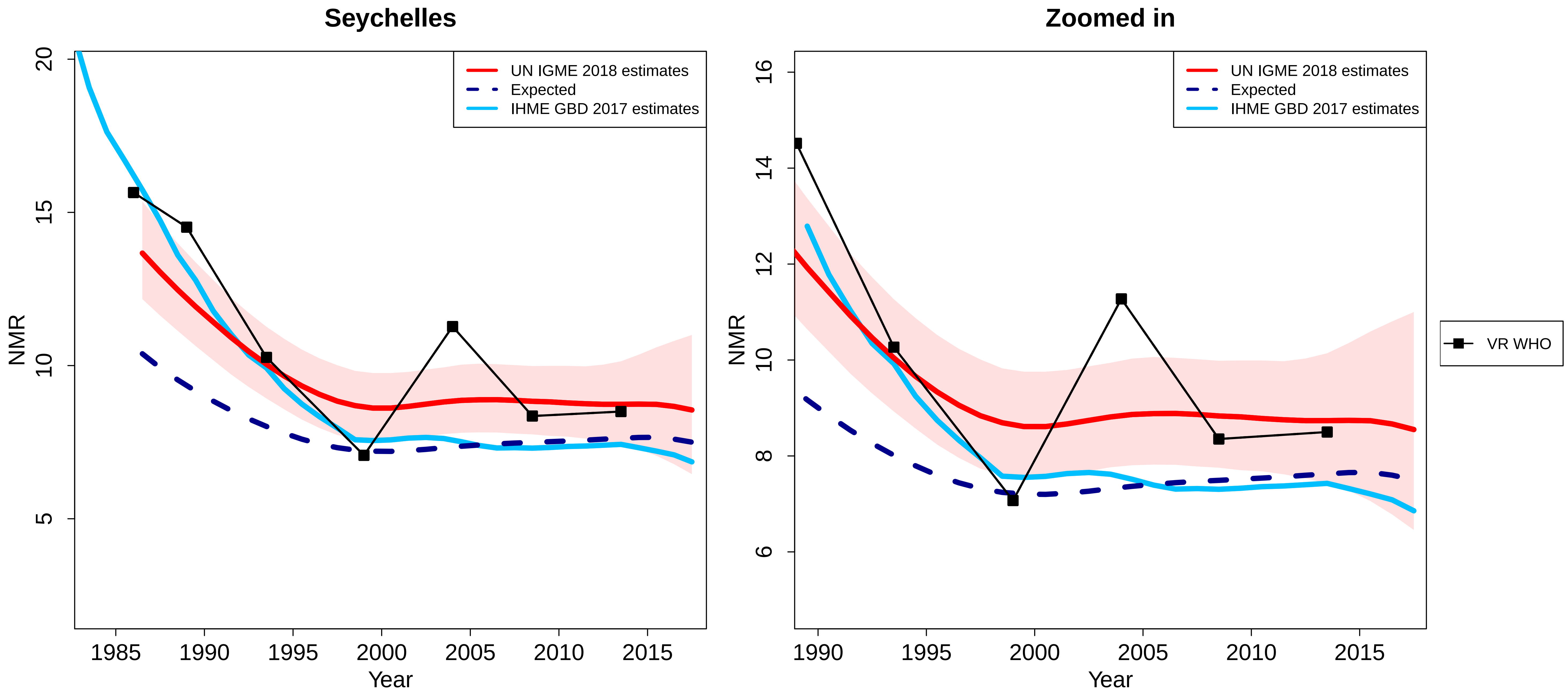
Seychelles
Zoomed in
UN IGME 2018 estimates
Expected
IHME GBD 2017 estimates
VR WHO
NMR
Year
1985
1990
1995
2000
2005
2010
2015
5
10
15
20
6
8
10
12
14
16

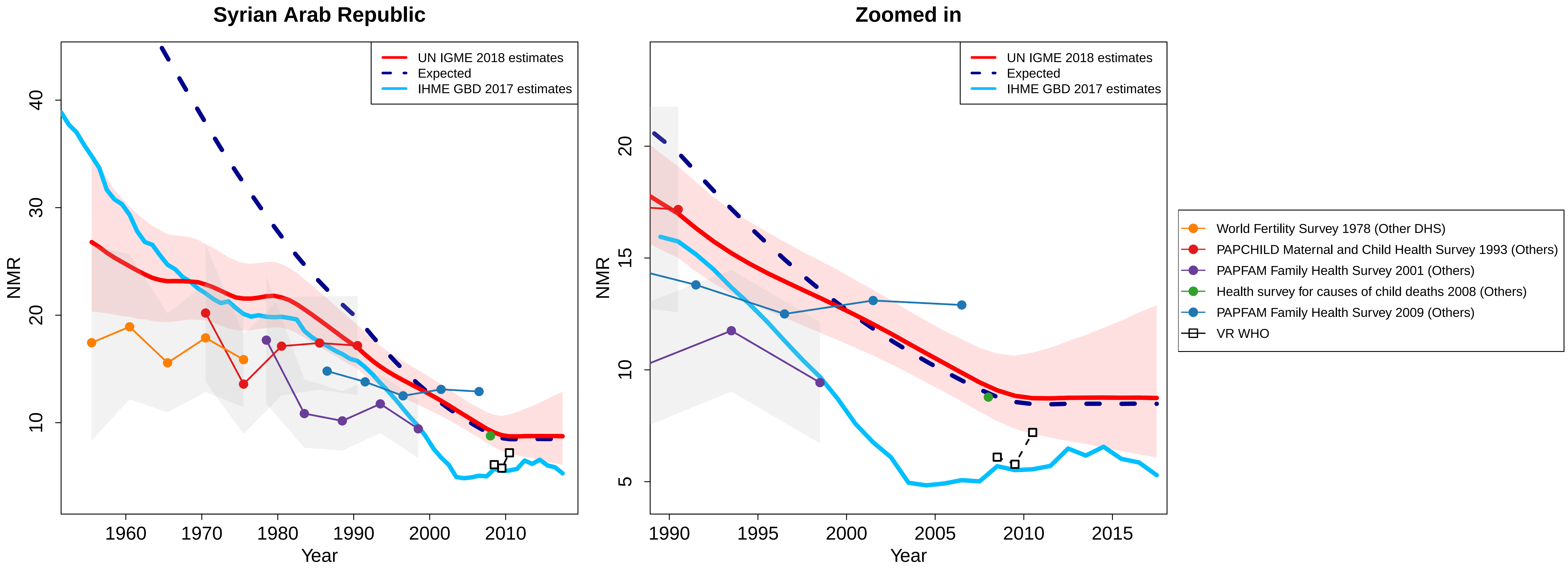

Syrian Arab Republic
Zoomed in
UN IGME 2018 estimates
Expected
IHME GBD 2017 estimates
NMR
Year
World Fertility Survey 1978 (Other DHS)
PAPCHILD Maternal and Child Health Survey 1993 (Others)
PAPFAM Family Health Survey 2001 (Others)
Health survey for causes of child deaths 2008 (Others)
PAPFAM Family Health Survey 2009 (Others)
VR WHO

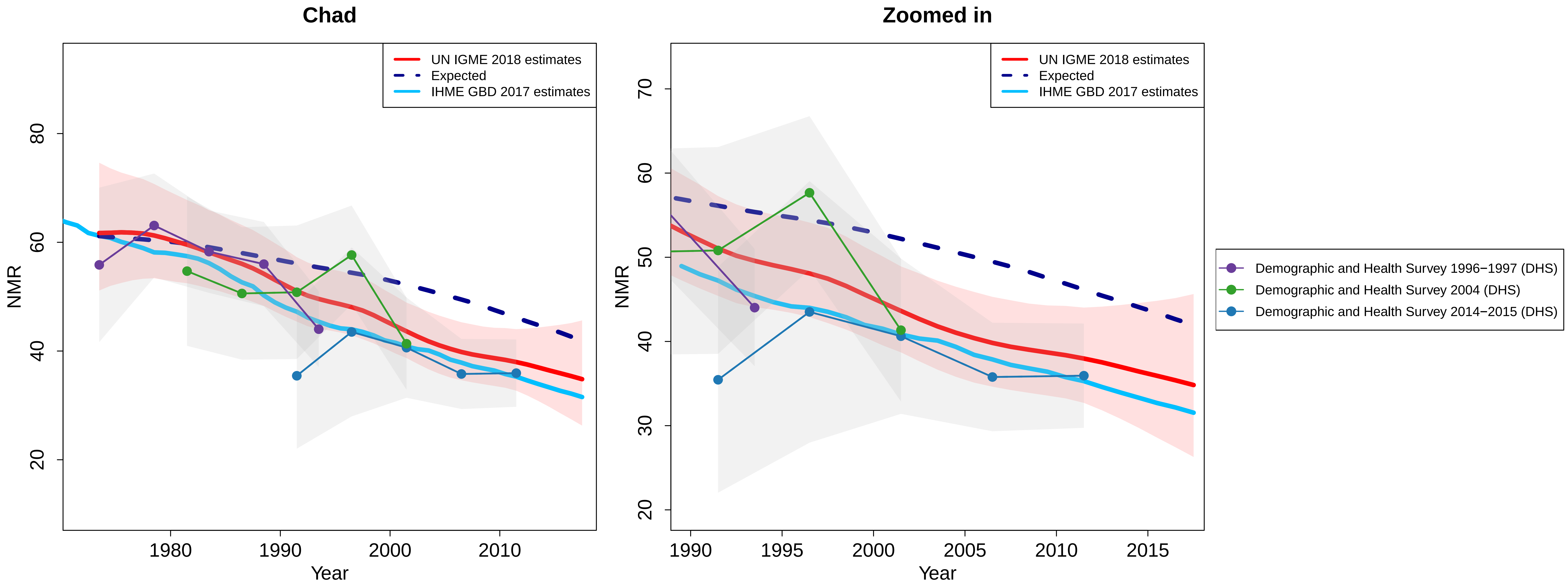

Chad
Zoomed in
UN IGME 2018 estimates
Expected
IHME GBD 2017 estimates
Demographic and Health Survey 1996−1997 (DHS)
Demographic and Health Survey 2004 (DHS)
Demographic and Health Survey 2014−2015 (DHS)
NMR
Year
20
40
60
80
1980
1990
2000
2010
30
50
70
1995
2005
2015

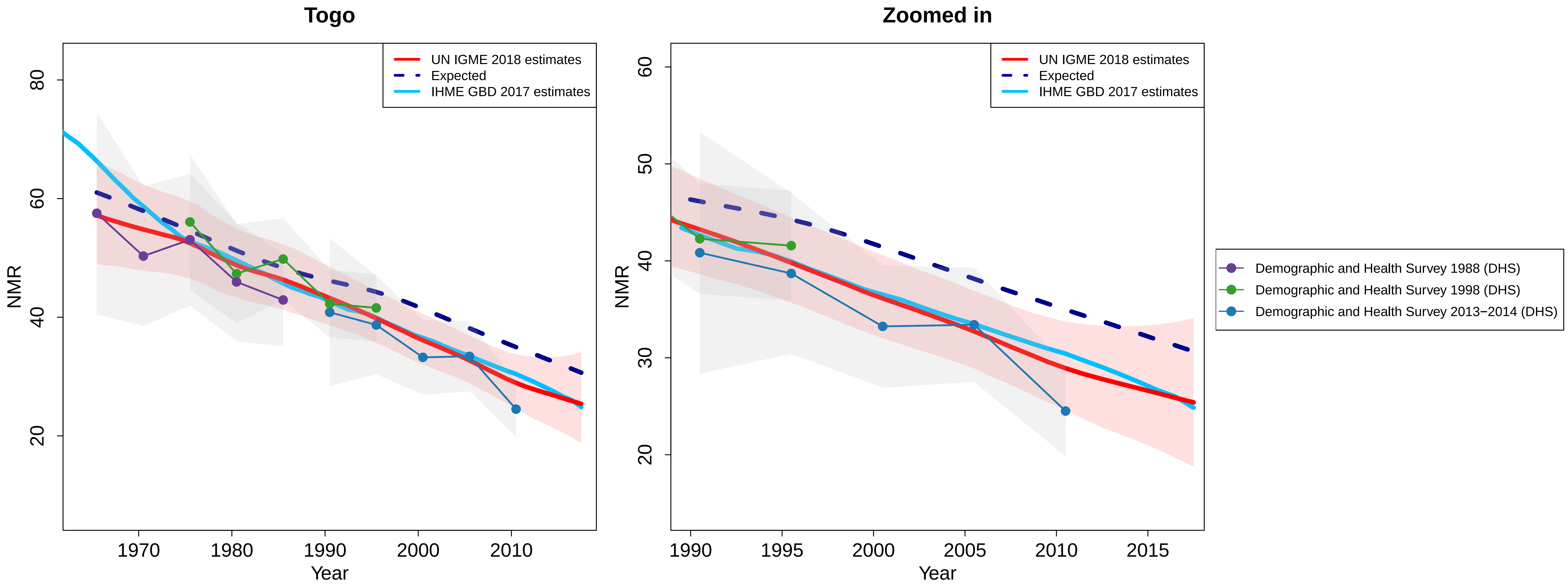

Togo
Zoomed in
UN IGME 2018 estimates
Expected
IHME GBD 2017 estimates
Demographic and Health Survey 1988 (DHS)
Demographic and Health Survey 1998 (DHS)
Demographic and Health Survey 2013−2014 (DHS)
NMR
Year

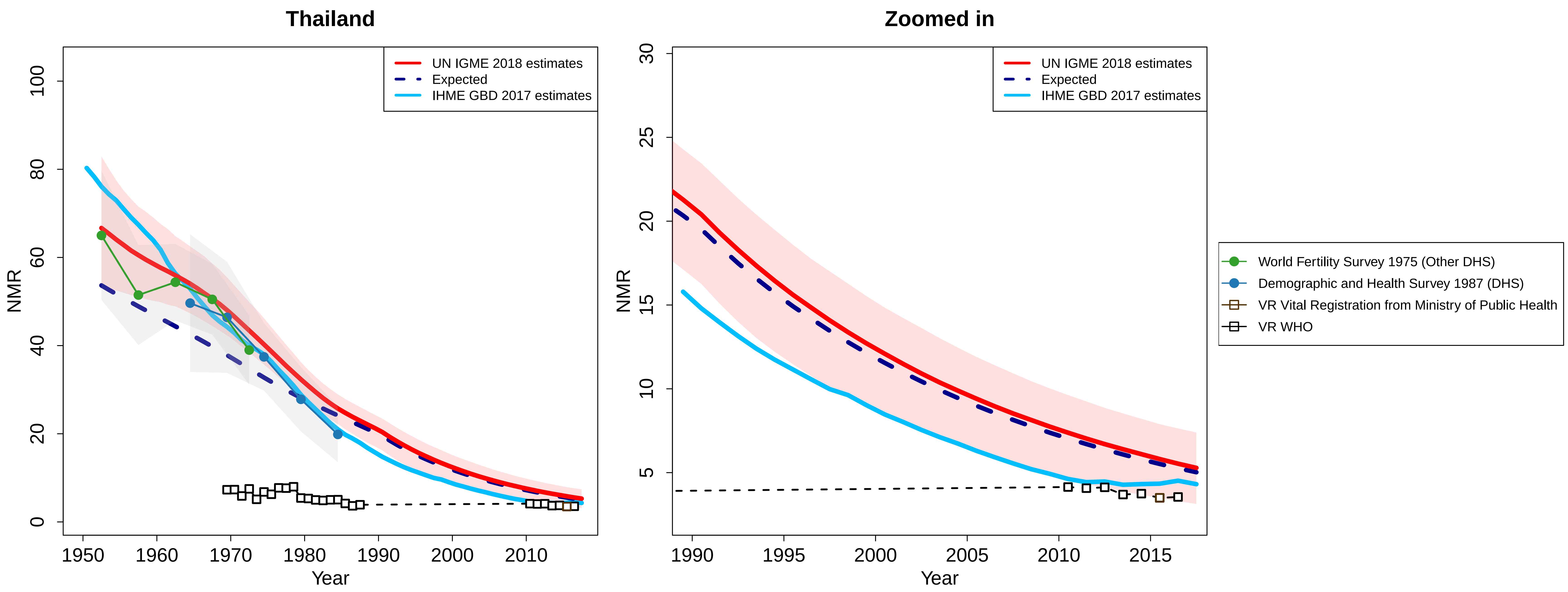
Thailand
Zoomed in
UN IGME 2018 estimates
Expected
IHME GBD 2017 estimates
NMR
Year
0
20
40
60
80
100
1950
1960
1970
1980
1990
2000
2010
5
10
15
20
25
30
1995
2005
2015
World Fertility Survey 1975 (Other DHS)
Demographic and Health Survey 1987 (DHS)
VR Vital Registration from Ministry of Public Health
VR WHO

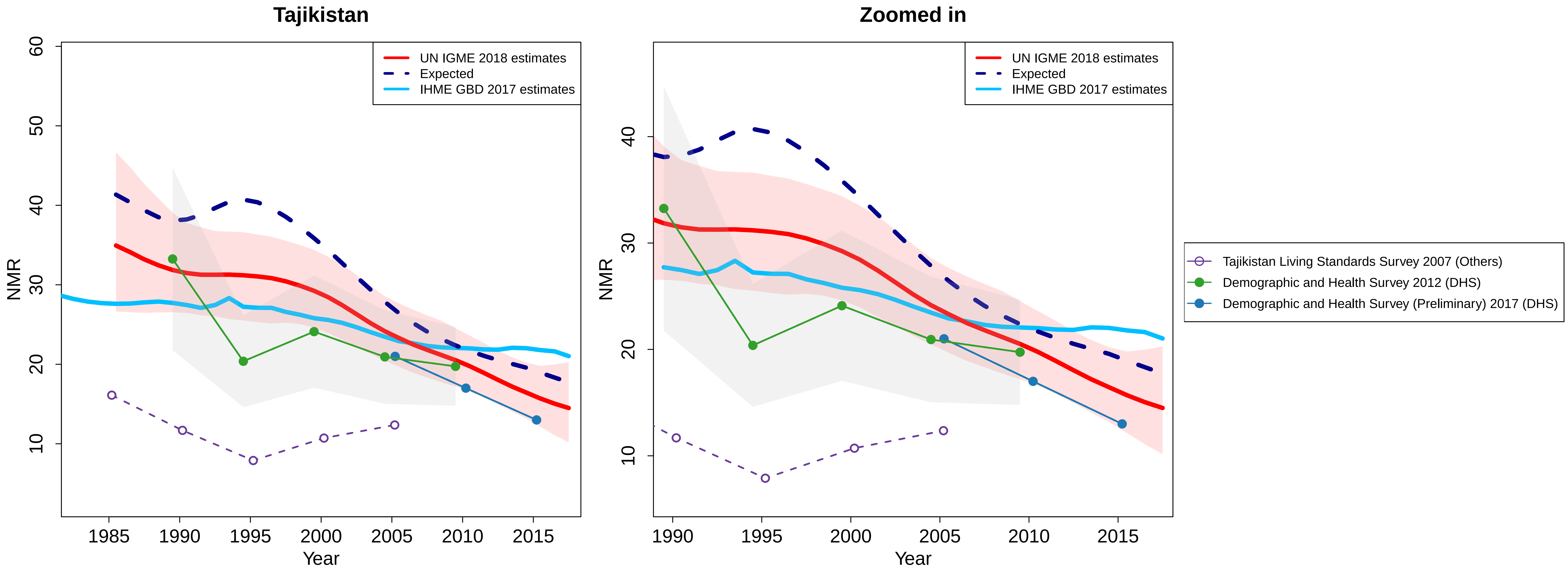
Tajikistan
Zoomed in
UN IGME 2018 estimates
Expected
IHME GBD 2017 estimates
Tajikistan Living Standards Survey 2007 (Others)
Demographic and Health Survey 2012 (DHS)
Demographic and Health Survey (Preliminary) 2017 (DHS)
NMR
Year
10
20
30
40
50
60
1985
1990
1995
2000
2005
2010
2015

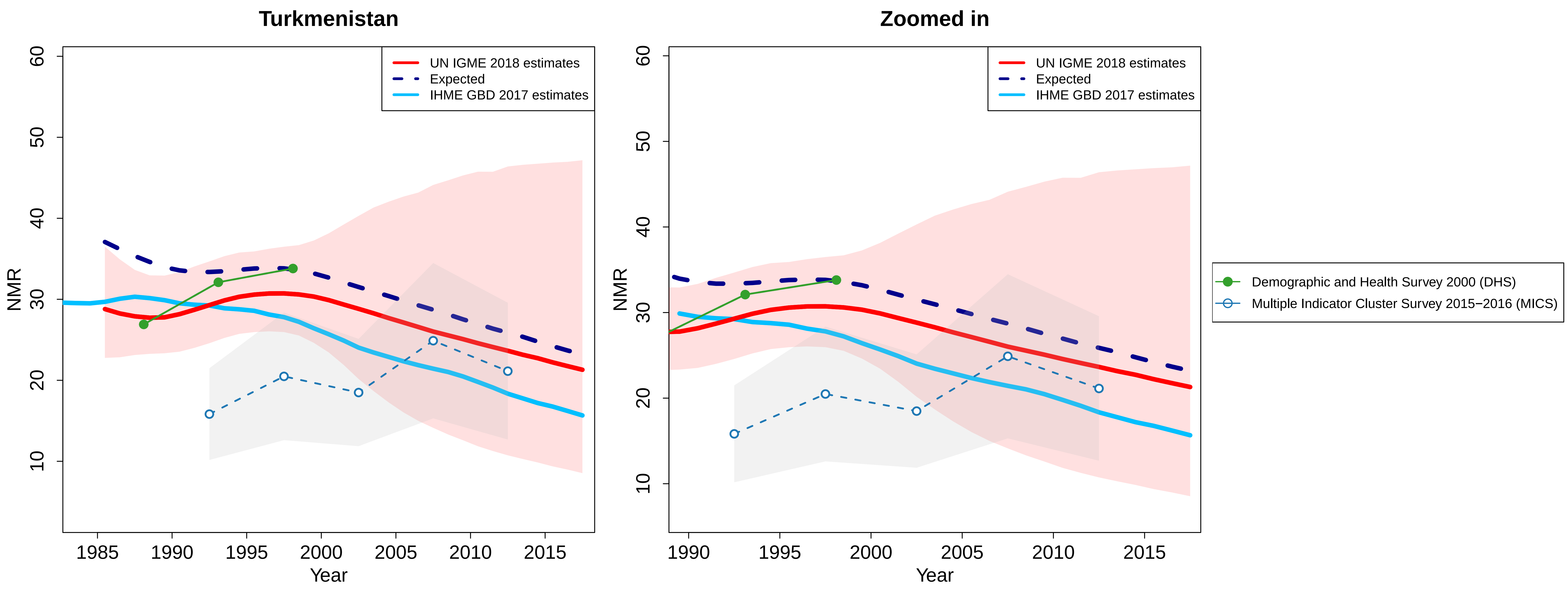
Turkmenistan
Zoomed in
UN IGME 2018 estimates
Expected
IHME GBD 2017 estimates
NMR
Year
10
20
30
40
50
60
1985
1990
1995
2000
2005
2010
2015
Demographic and Health Survey 2000 (DHS)
Multiple Indicator Cluster Survey 2015−2016 (MICS)

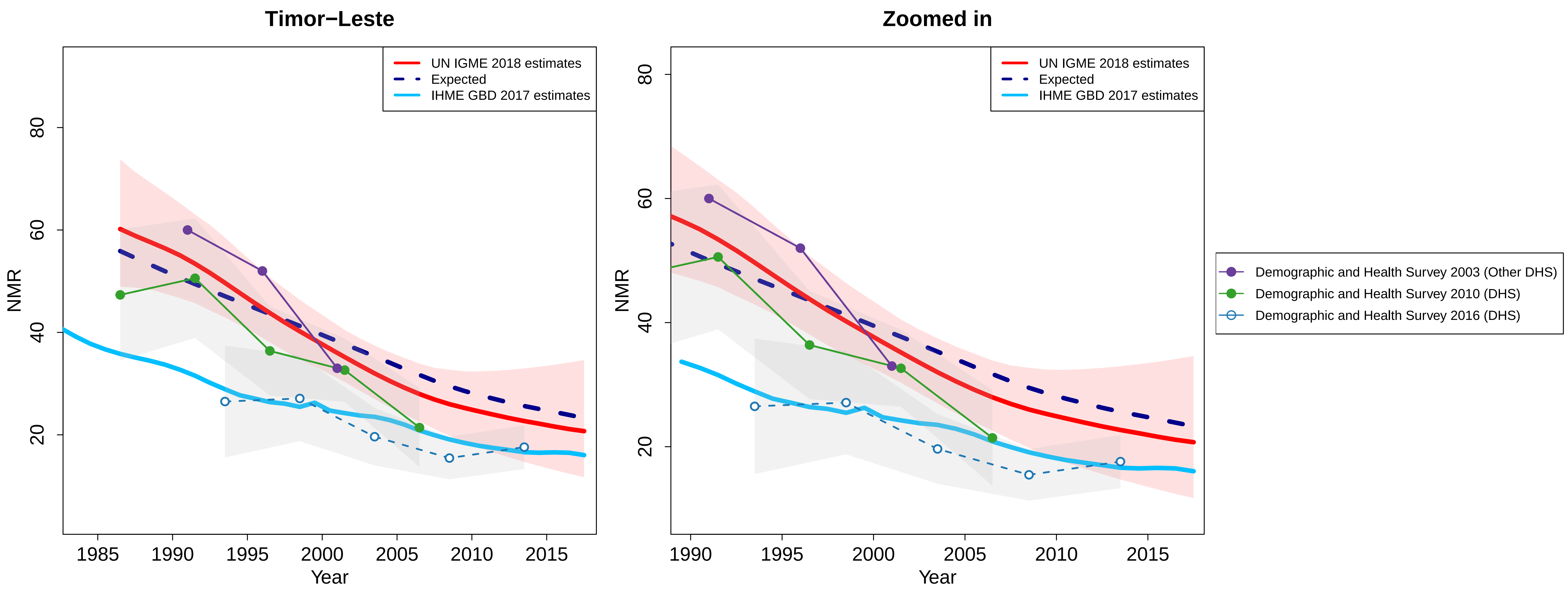
Timor−Leste
Zoomed in
UN IGME 2018 estimates
Expected
IHME GBD 2017 estimates
Demographic and Health Survey 2003 (Other DHS)
Demographic and Health Survey 2010 (DHS)
Demographic and Health Survey 2016 (DHS)
NMR
Year
1985
1990
1995
2000
2005
2010
2015
20
40
60
80

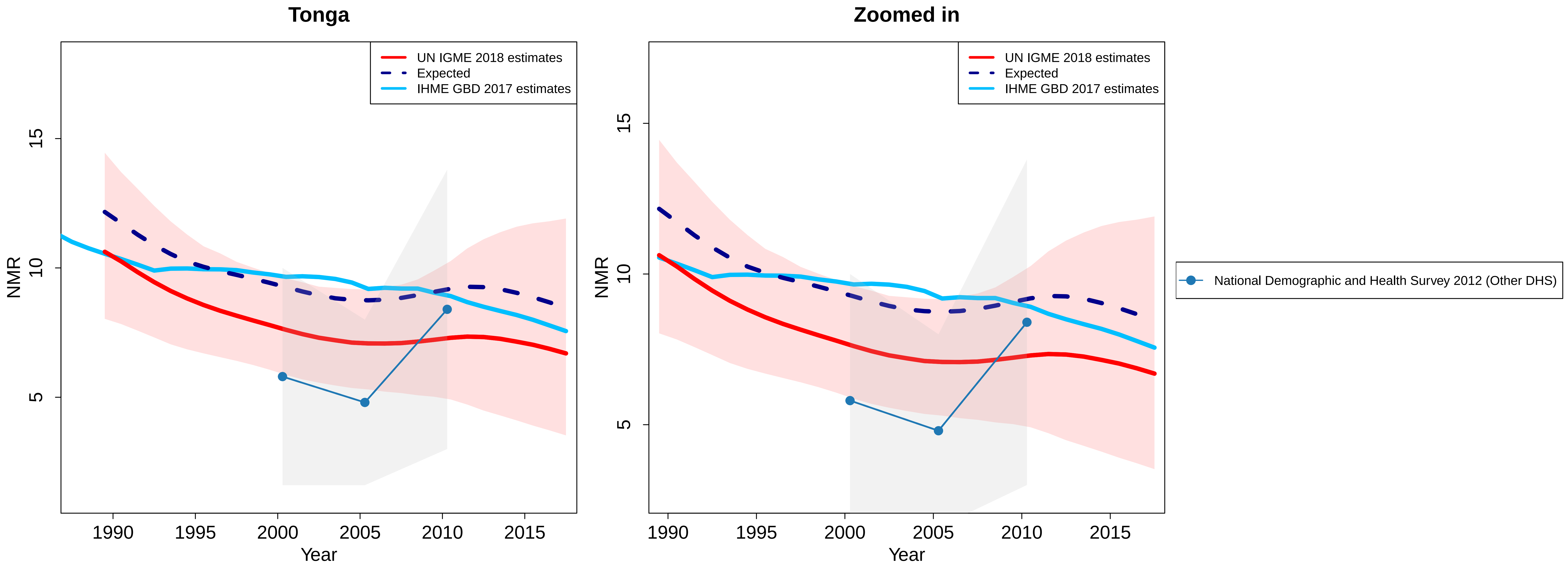

Tonga
Zoomed in
UN IGME 2018 estimates
Expected
IHME GBD 2017 estimates
National Demographic and Health Survey 2012 (Other DHS)
NMR
Year
5
10
15
1990
1995
2000
2005
2010
2015

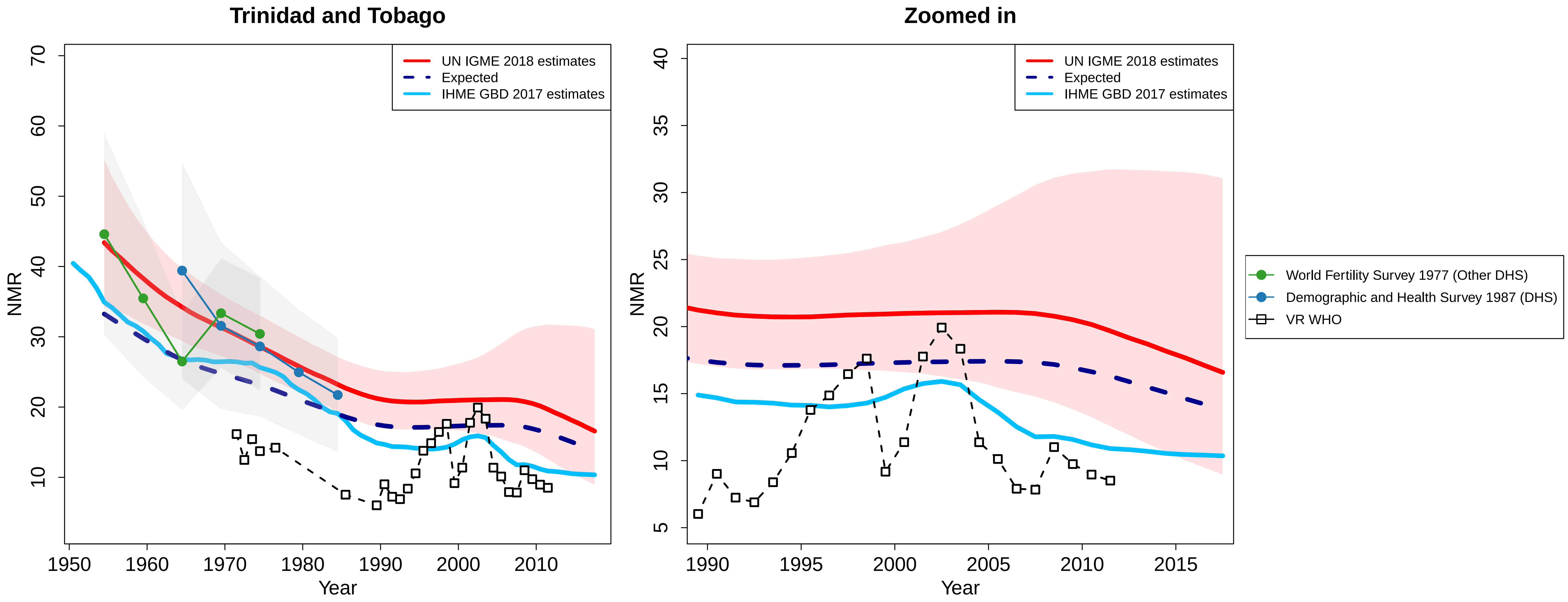

Trinidad and Tobago
Zoomed in
UN IGME 2018 estimates
Expected
IHME GBD 2017 estimates
NMR
Year
World Fertility Survey 1977 (Other DHS)
Demographic and Health Survey 1987 (DHS)
VR WHO

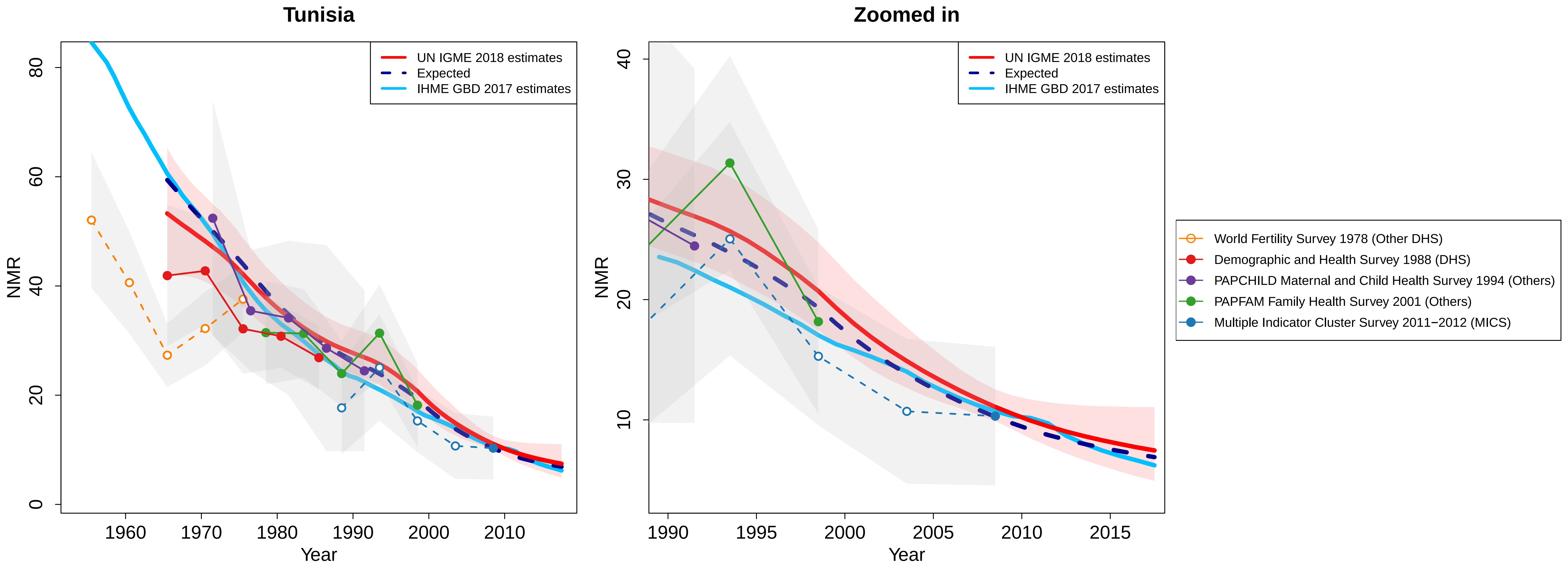
Tunisia
Zoomed in
UN IGME 2018 estimates
Expected
IHME GBD 2017 estimates
NMR
Year
World Fertility Survey 1978 (Other DHS)
Demographic and Health Survey 1988 (DHS)
PAPCHILD Maternal and Child Health Survey 1994 (Others)
PAPFAM Family Health Survey 2001 (Others)
Multiple Indicator Cluster Survey 2011−2012 (MICS)

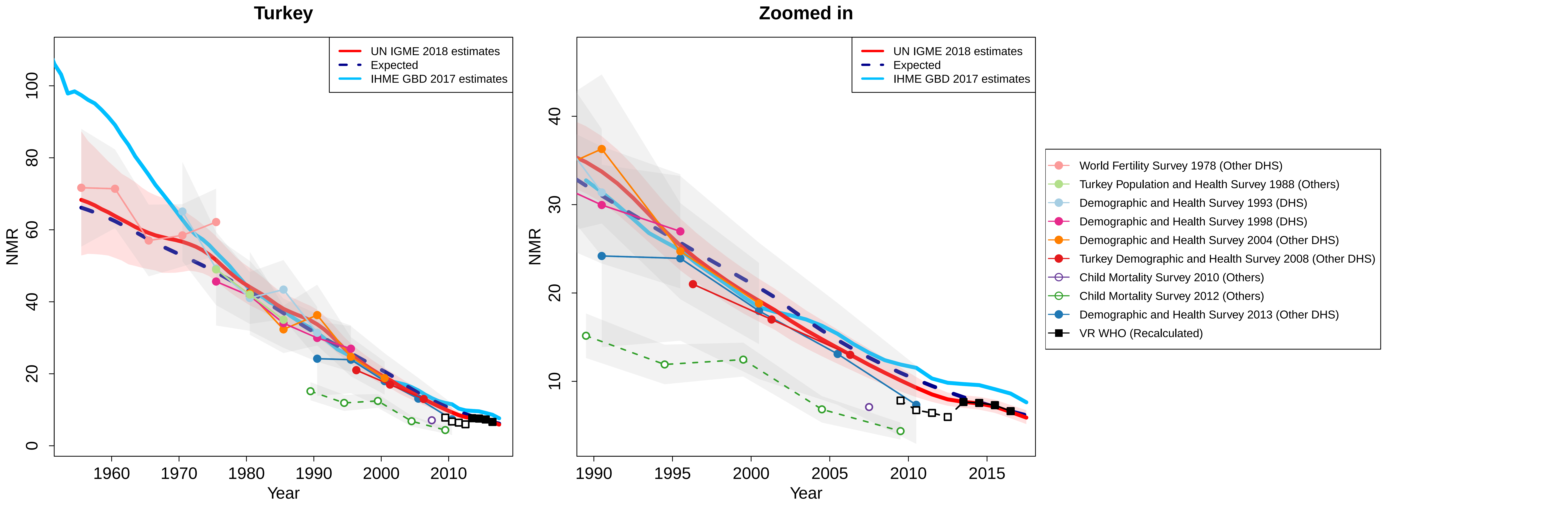

Turkey
Zoomed in
UN IGME 2018 estimates
Expected
IHME GBD 2017 estimates
NMR
Year
World Fertility Survey 1978 (Other DHS)
Turkey Population and Health Survey 1988 (Others)
Demographic and Health Survey 1993 (DHS)
Demographic and Health Survey 1998 (DHS)
Demographic and Health Survey 2004 (Other DHS)
Turkey Demographic and Health Survey 2008 (Other DHS)
Child Mortality Survey 2010 (Others)
Child Mortality Survey 2012 (Others)
Demographic and Health Survey 2013 (Other DHS)
VR WHO (Recalculated)

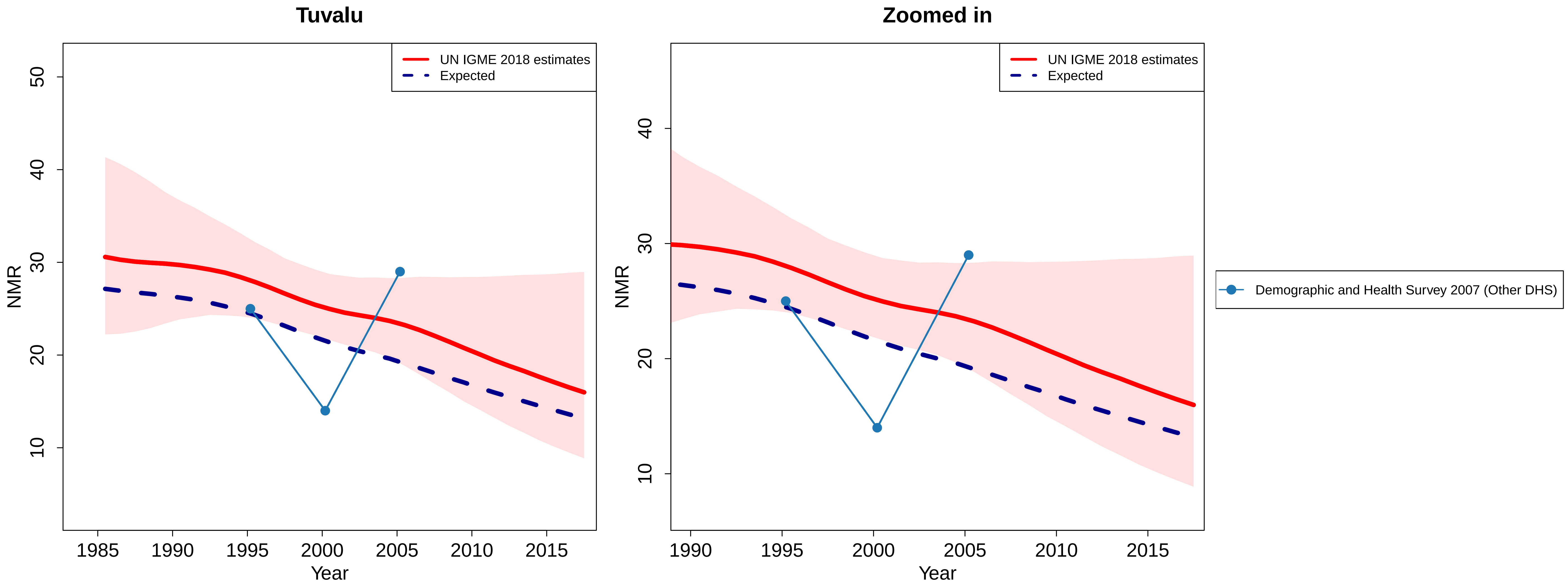

Tuvalu
Zoomed in
UN IGME 2018 estimates
Expected
NMR
Year
1985
1990
1995
2000
2005
2010
2015
10
20
30
40
50
Demographic and Health Survey 2007 (Other DHS)

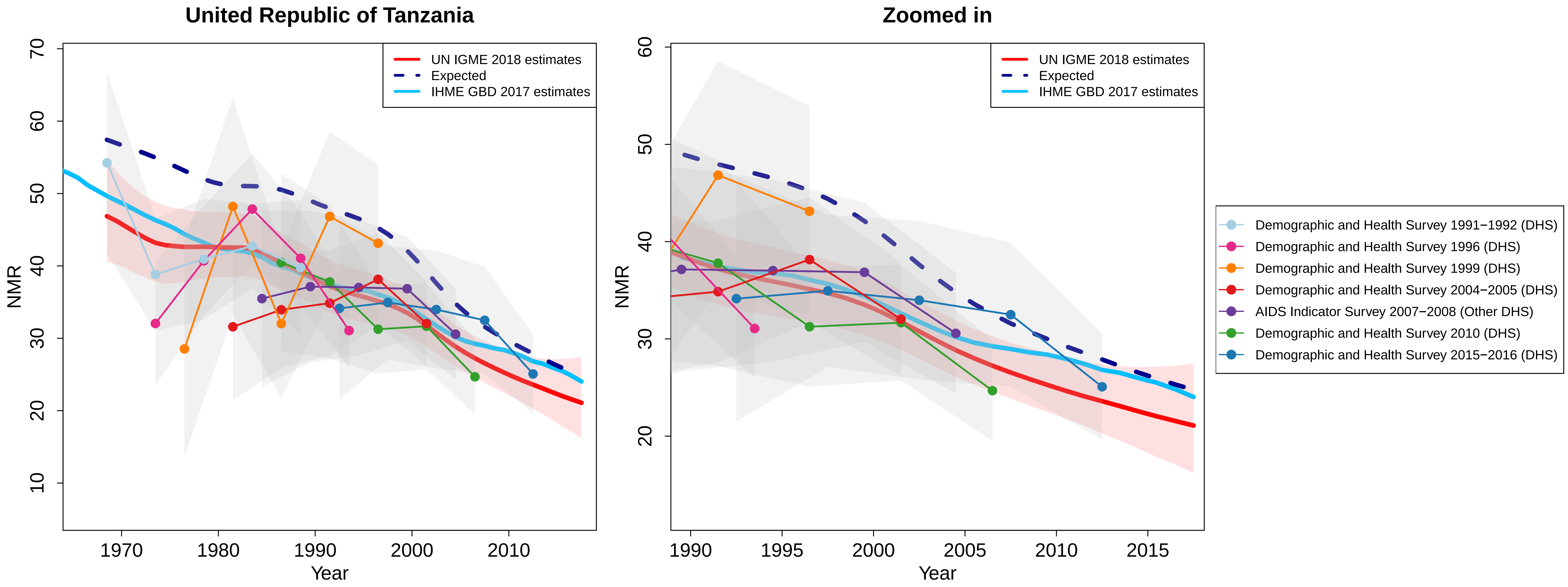
United Republic of Tanzania
Zoomed in
UN IGME 2018 estimates
Expected
IHME GBD 2017 estimates
NMR
Year
Demographic and Health Survey 1991−1992 (DHS)
Demographic and Health Survey 1996 (DHS)
Demographic and Health Survey 1999 (DHS)
Demographic and Health Survey 2004−2005 (DHS)
AIDS Indicator Survey 2007−2008 (Other DHS)
Demographic and Health Survey 2010 (DHS)
Demographic and Health Survey 2015−2016 (DHS)

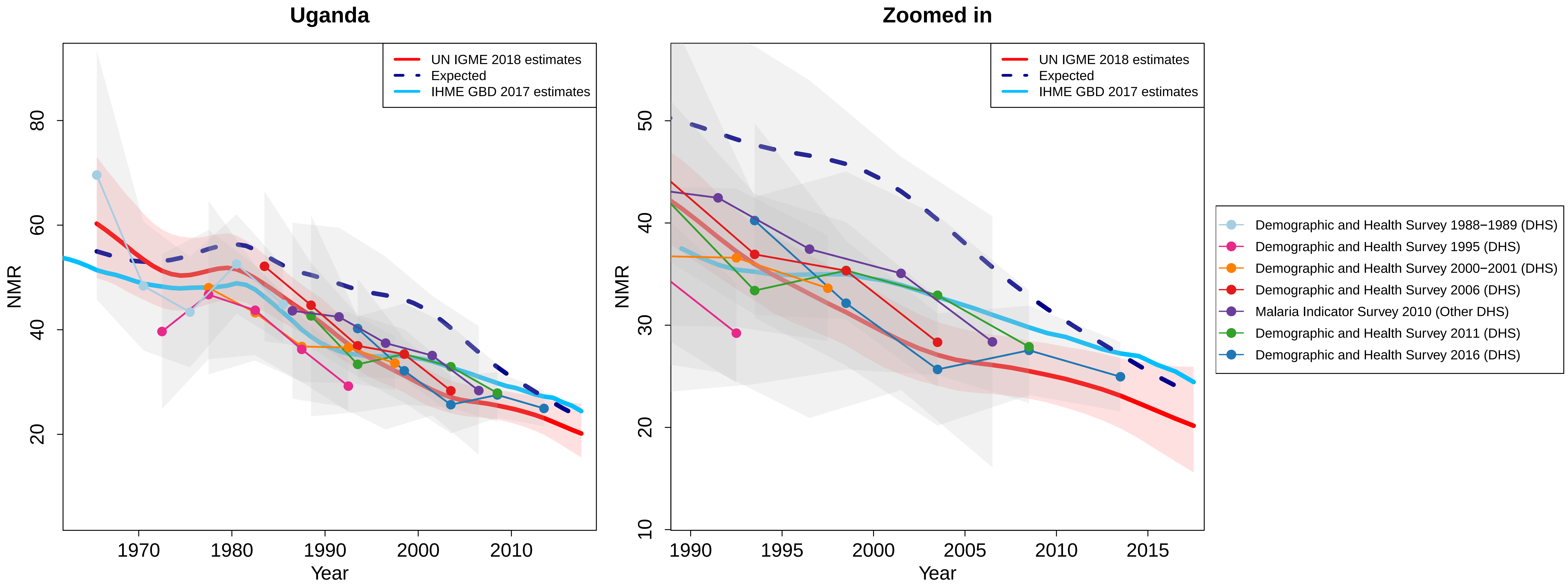
Uganda
Zoomed in
UN IGME 2018 estimates
Expected
IHME GBD 2017 estimates
NMR
Year
Demographic and Health Survey 1988−1989 (DHS)
Demographic and Health Survey 1995 (DHS)
Demographic and Health Survey 2000−2001 (DHS)
Demographic and Health Survey 2006 (DHS)
Malaria Indicator Survey 2010 (Other DHS)
Demographic and Health Survey 2011 (DHS)
Demographic and Health Survey 2016 (DHS)

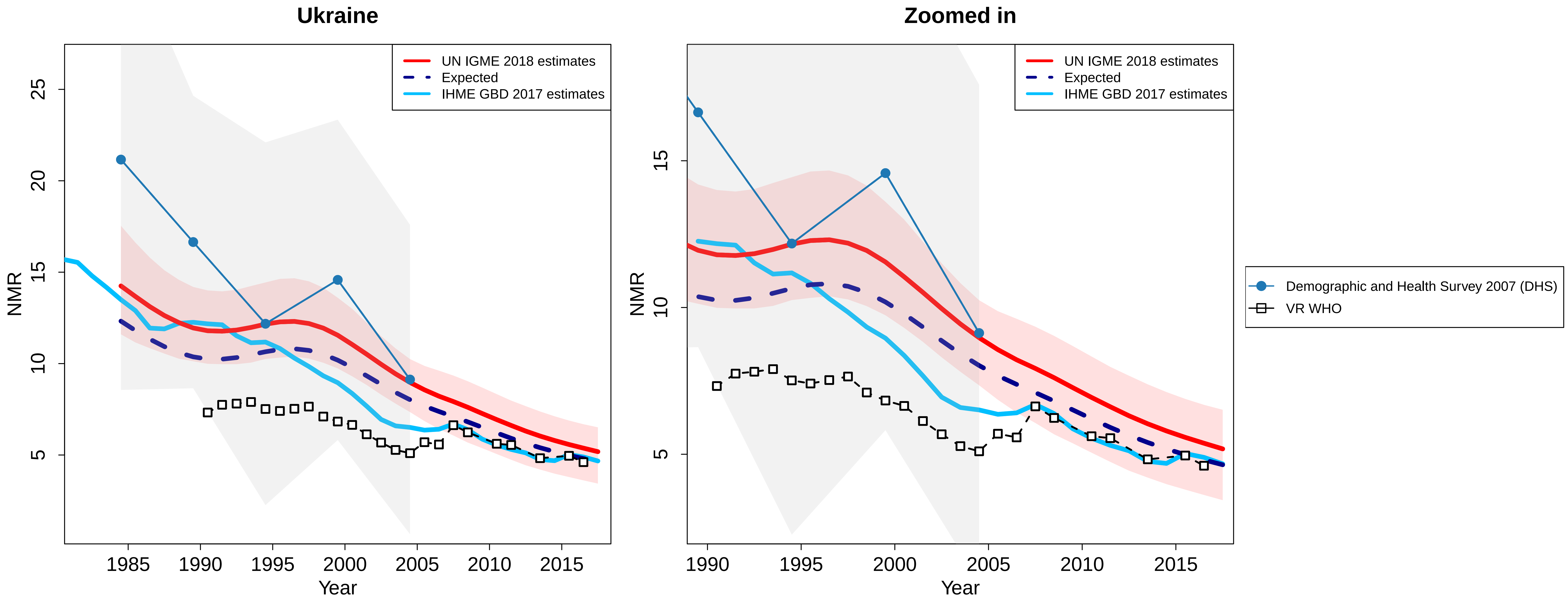
Ukraine
Zoomed in
UN IGME 2018 estimates
Expected
IHME GBD 2017 estimates
Demographic and Health Survey 2007 (DHS)
VR WHO
NMR
Year
1985
1990
1995
2000
2005
2010
2015
5
10
15
20
25

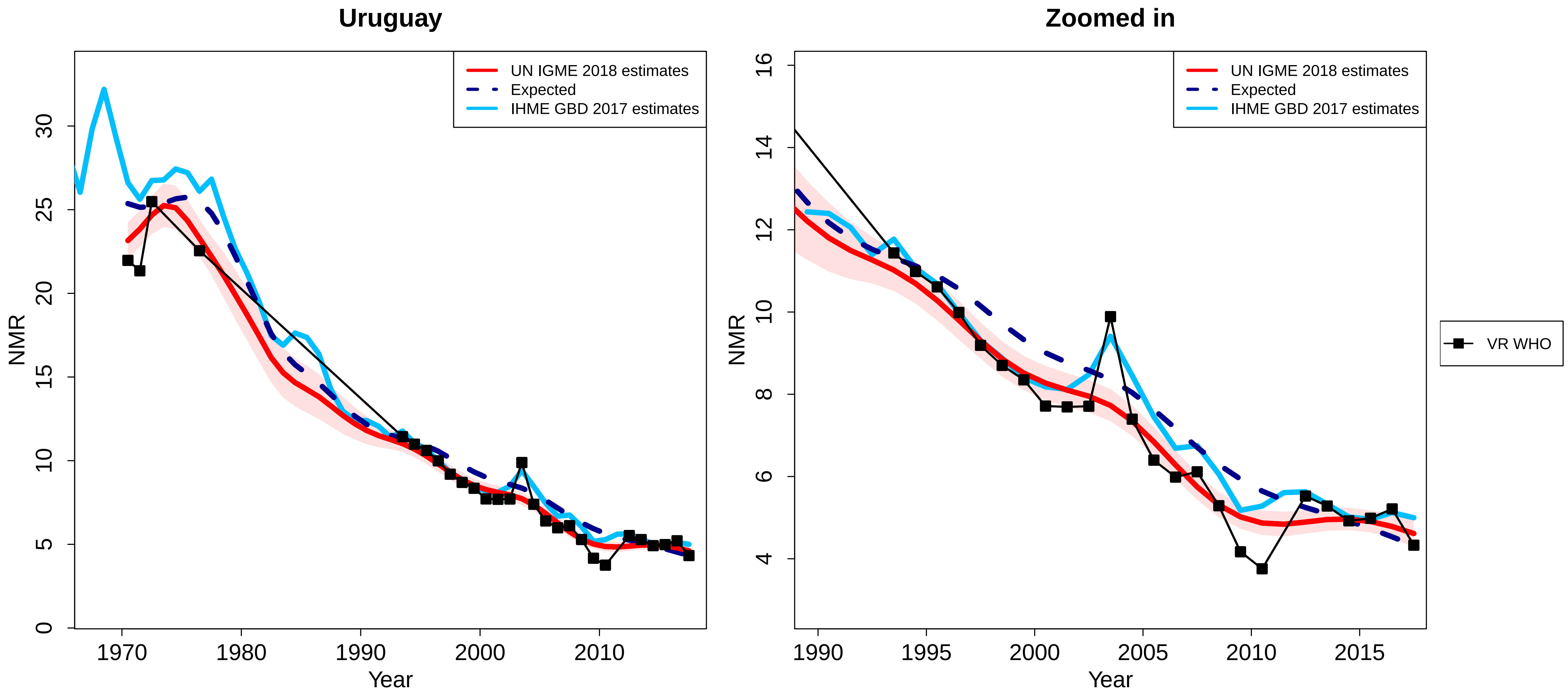
Uruguay
Zoomed in
UN IGME 2018 estimates
Expected
IHME GBD 2017 estimates
VR WHO
NMR
Year
0
5
10
15
20
25
30
1970
1980
1990
2000
2010
4
6
8
10
12
14
16
1990
1995
2000
2005
2010
2015

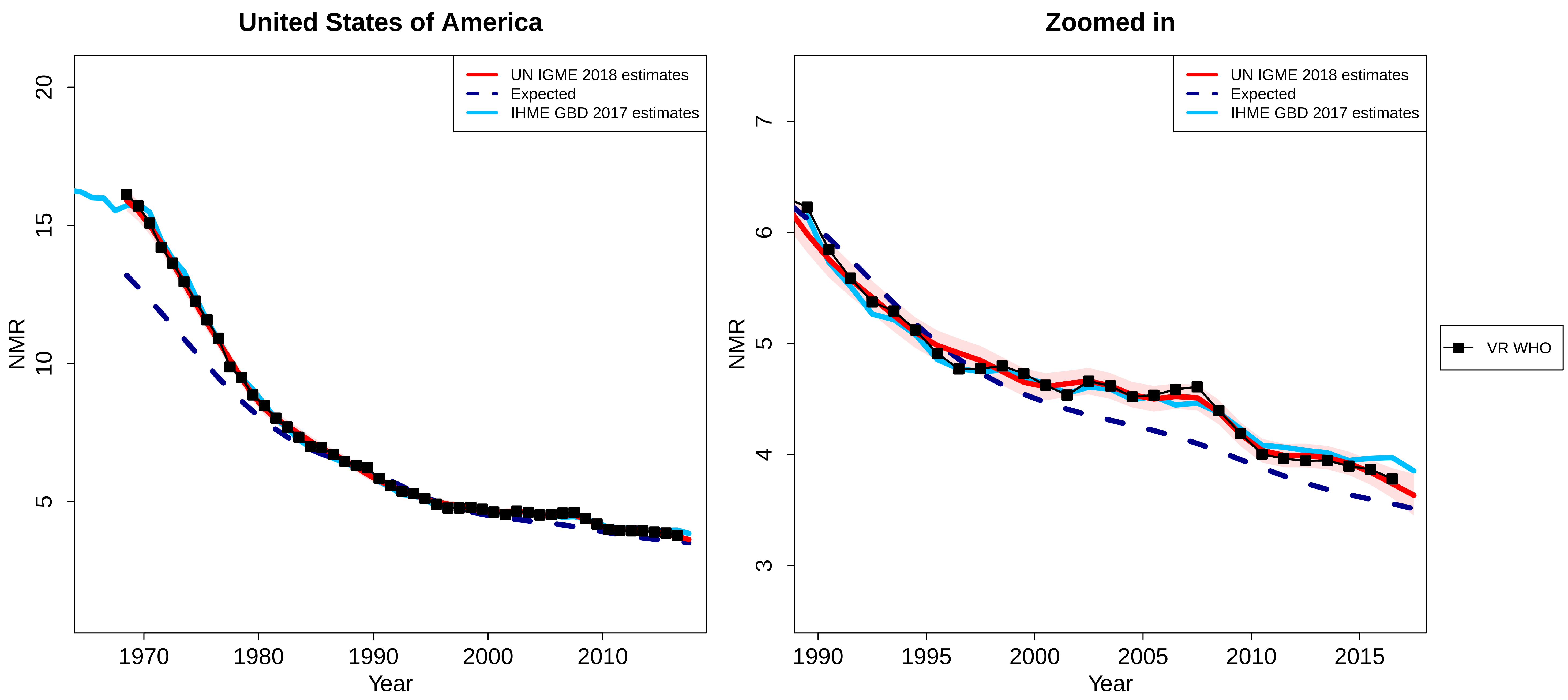
United States of America
Zoomed in
UN IGME 2018 estimates
Expected
IHME GBD 2017 estimates
VR WHO
NMR
Year
1970
1980
1990
2000
2010
5
10
15
20
1995
2005
2015
3
4
5
6
7

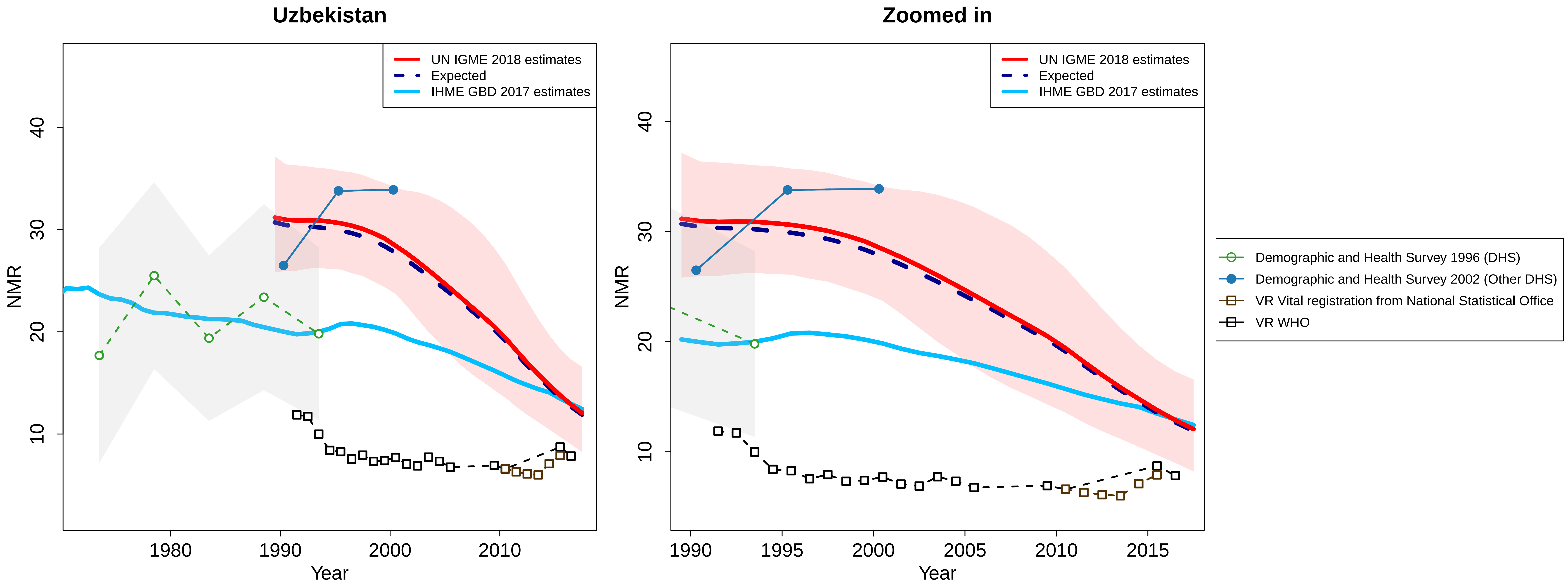

Uzbekistan
Zoomed in
UN IGME 2018 estimates
Expected
IHME GBD 2017 estimates
NMR
Year
10
20
30
40
1980
1990
2000
2010
1995
2005
2015
Demographic and Health Survey 1996 (DHS)
Demographic and Health Survey 2002 (Other DHS)
VR Vital registration from National Statistical Office
VR WHO

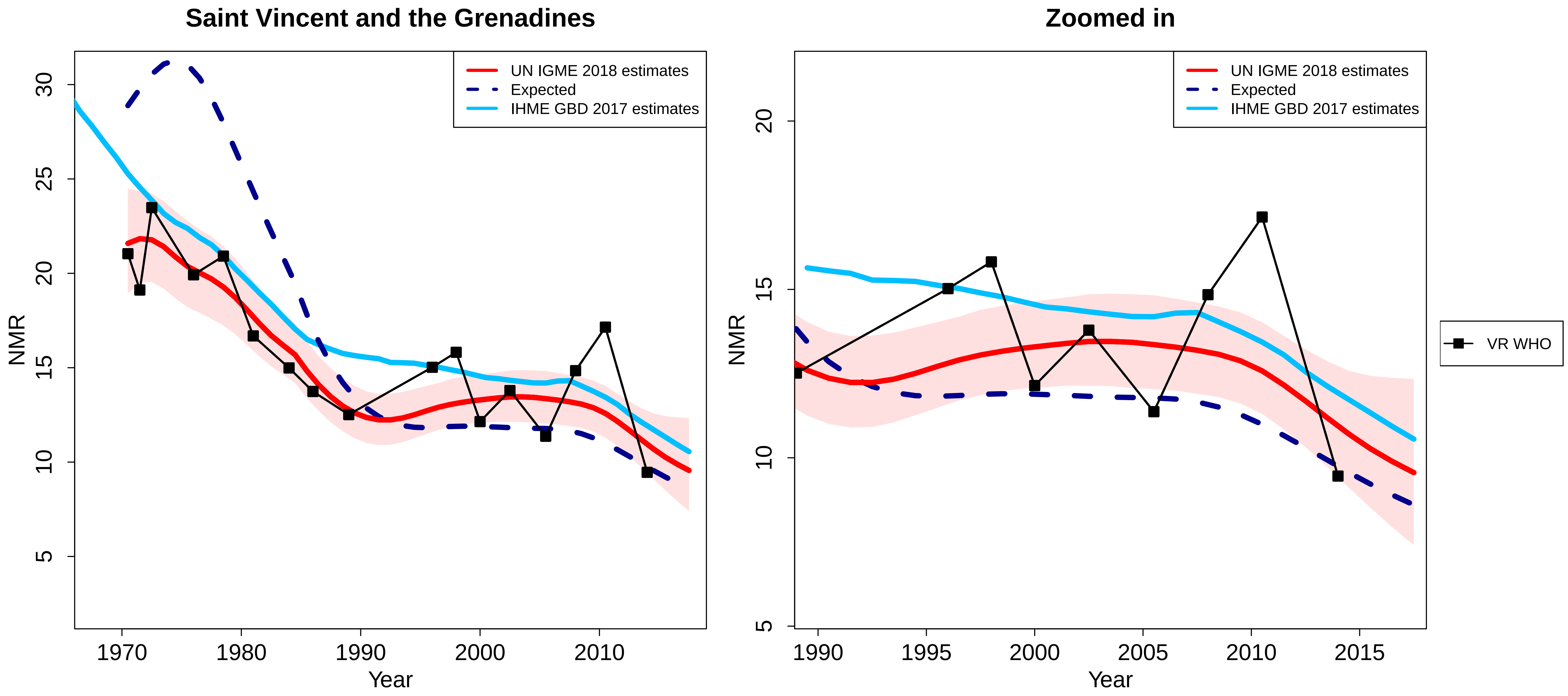

Saint Vincent and the Grenadines
Zoomed in
UN IGME 2018 estimates
Expected
IHME GBD 2017 estimates
NMR
Year
VR WHO

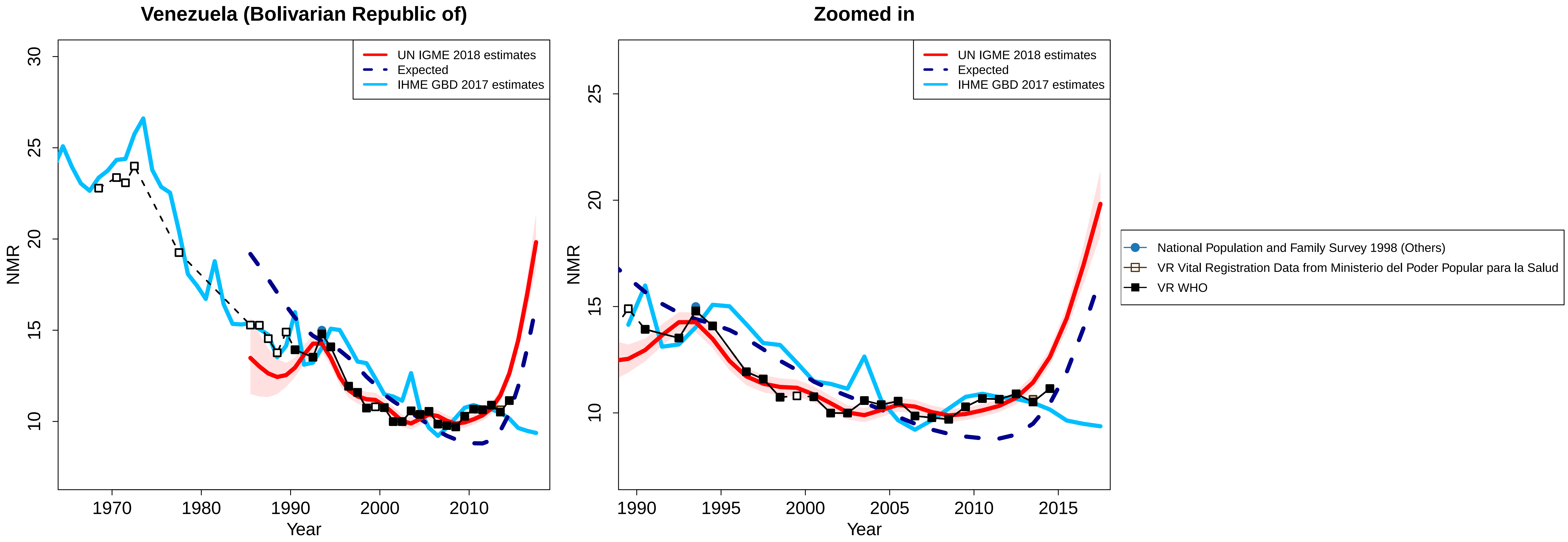
Venezuela (Bolivarian Republic of)
Zoomed in
UN IGME 2018 estimates
Expected
IHME GBD 2017 estimates
NMR
Year
National Population and Family Survey 1998 (Others)
VR Vital Registration Data from Ministerio del Poder Popular para la Salud
VR WHO

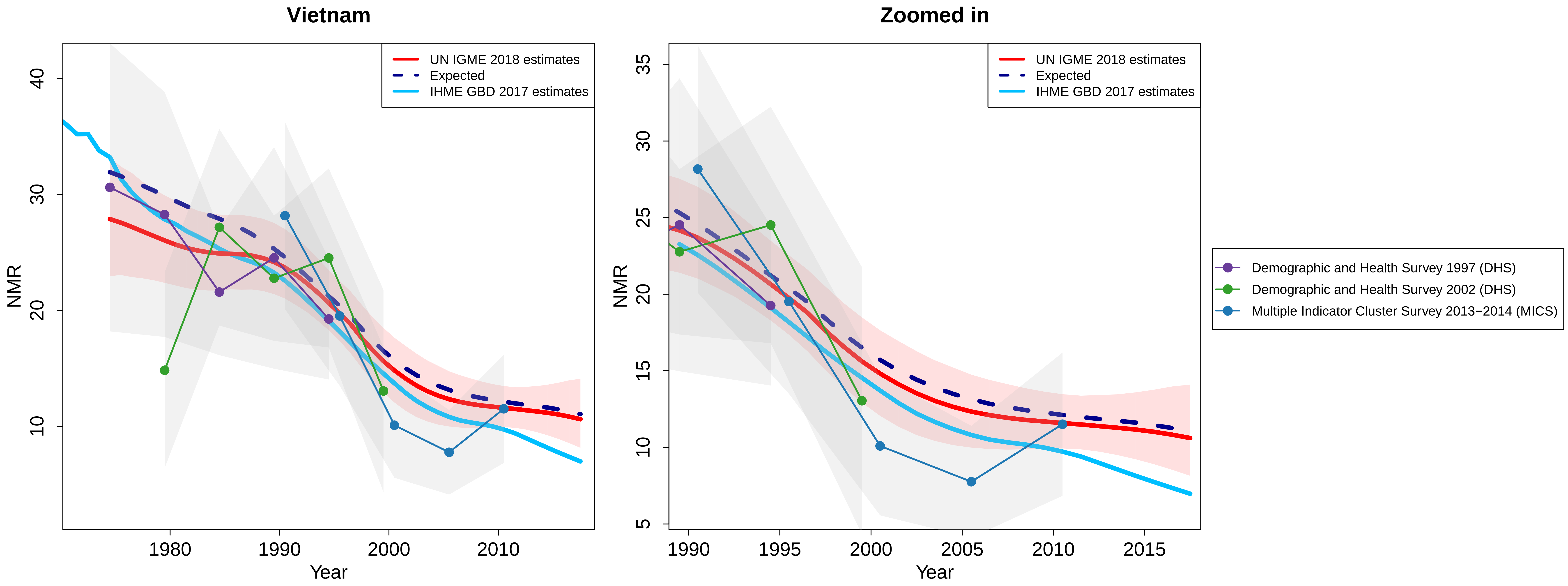

Vietnam
Zoomed in
UN IGME 2018 estimates
Expected
IHME GBD 2017 estimates
NMR
Year
Demographic and Health Survey 1997 (DHS)
Demographic and Health Survey 2002 (DHS)
Multiple Indicator Cluster Survey 2013−2014 (MICS)

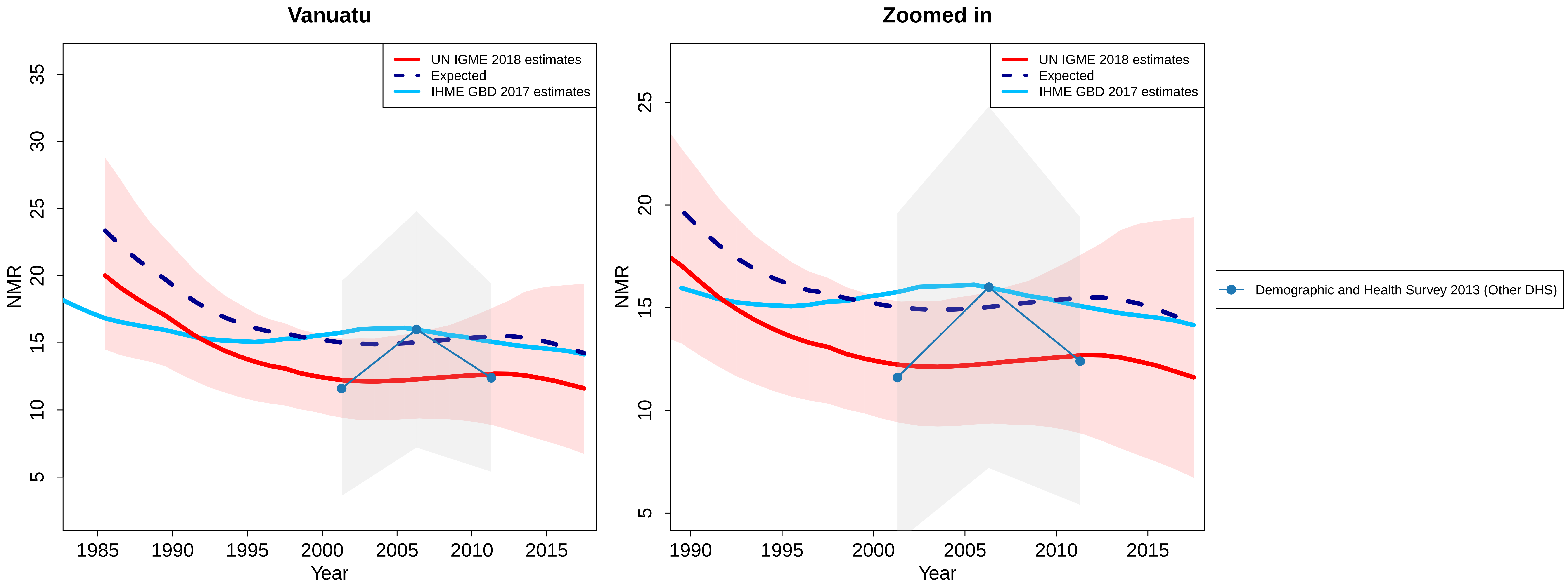

Vanuatu
Zoomed in
UN IGME 2018 estimates
Expected
IHME GBD 2017 estimates
Demographic and Health Survey 2013 (Other DHS)
NMR
Year

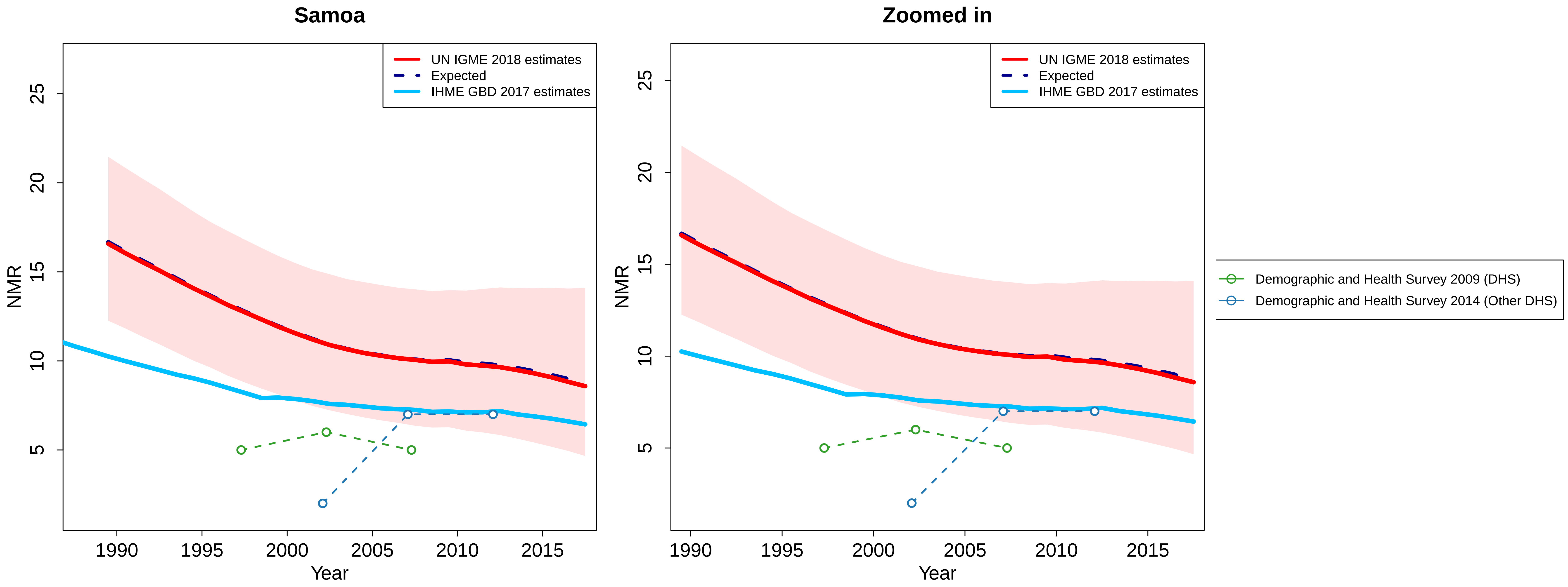

Samoa
Zoomed in
UN IGME 2018 estimates
Expected
IHME GBD 2017 estimates
NMR
Year
Demographic and Health Survey 2009 (DHS)
Demographic and Health Survey 2014 (Other DHS)

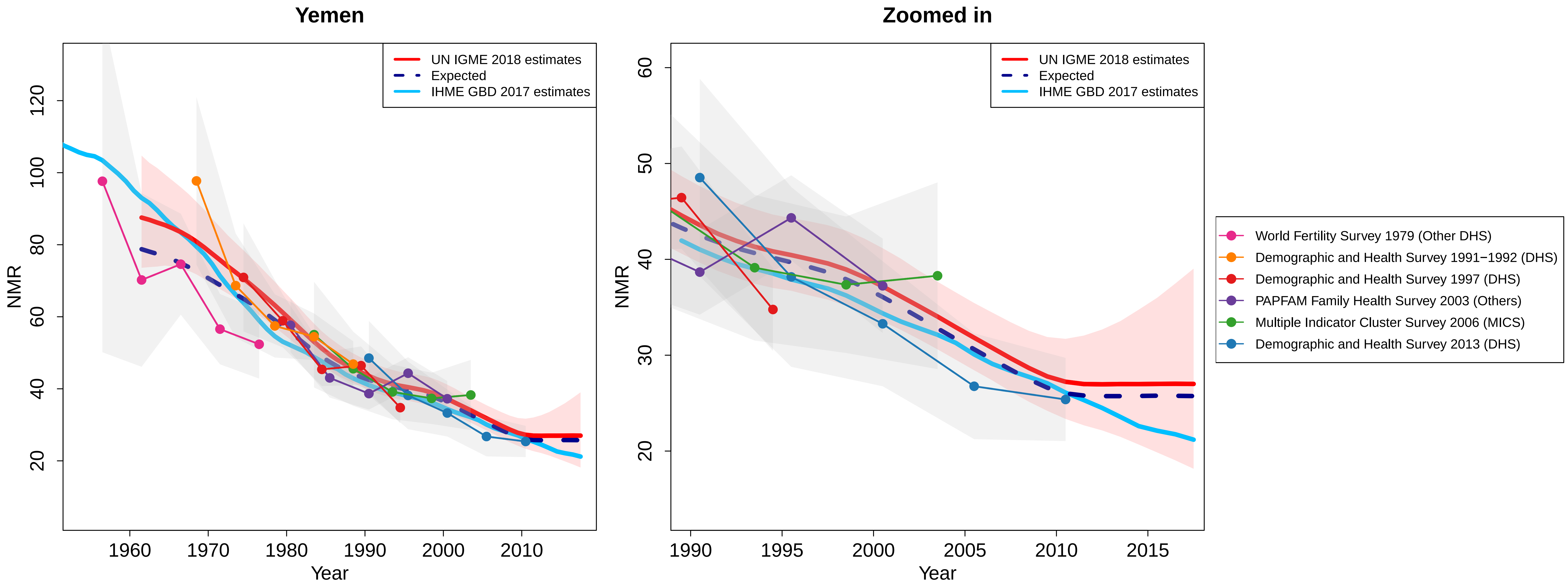
Yemen
Zoomed in
UN IGME 2018 estimates
Expected
IHME GBD 2017 estimates
NMR
Year
World Fertility Survey 1979 (Other DHS)
Demographic and Health Survey 1991−1992 (DHS)
Demographic and Health Survey 1997 (DHS)
PAPFAM Family Health Survey 2003 (Others)
Multiple Indicator Cluster Survey 2006 (MICS)
Demographic and Health Survey 2013 (DHS)

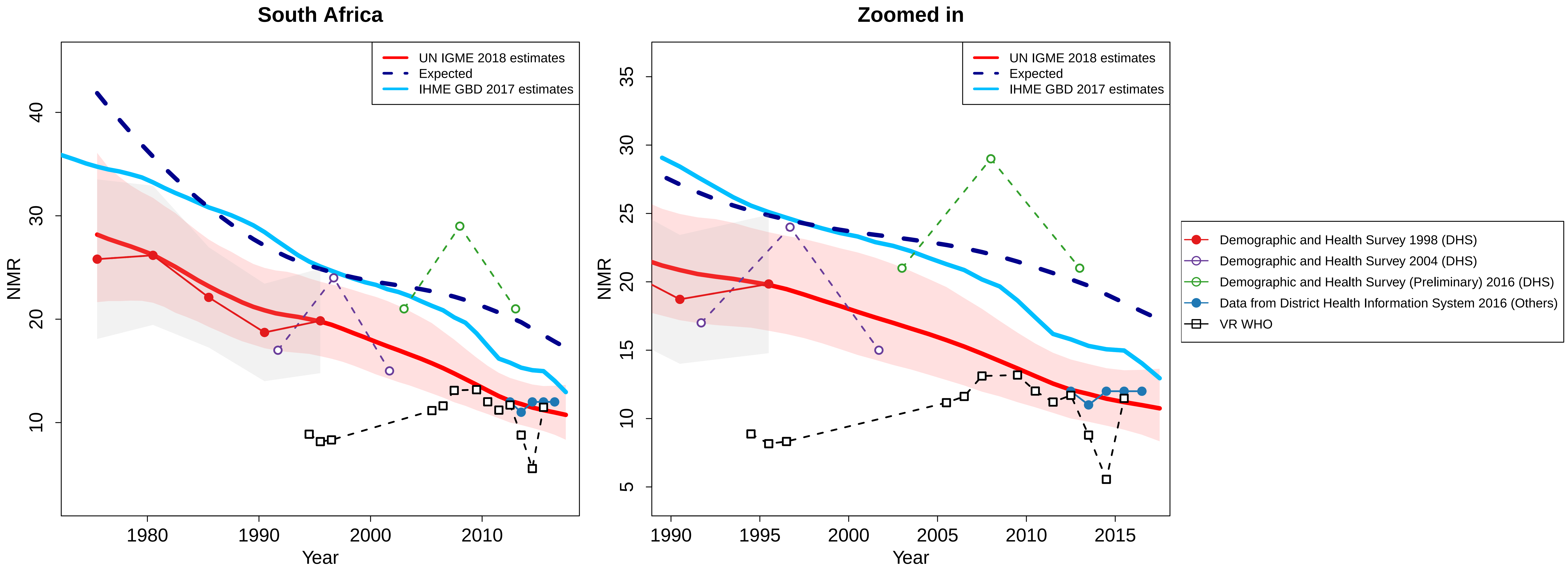

South Africa
Zoomed in
UN IGME 2018 estimates
Expected
IHME GBD 2017 estimates
NMR
Year
Demographic and Health Survey 1998 (DHS)
Demographic and Health Survey 2004 (DHS)
Demographic and Health Survey (Preliminary) 2016 (DHS)
Data from District Health Information System 2016 (Others)
VR WHO

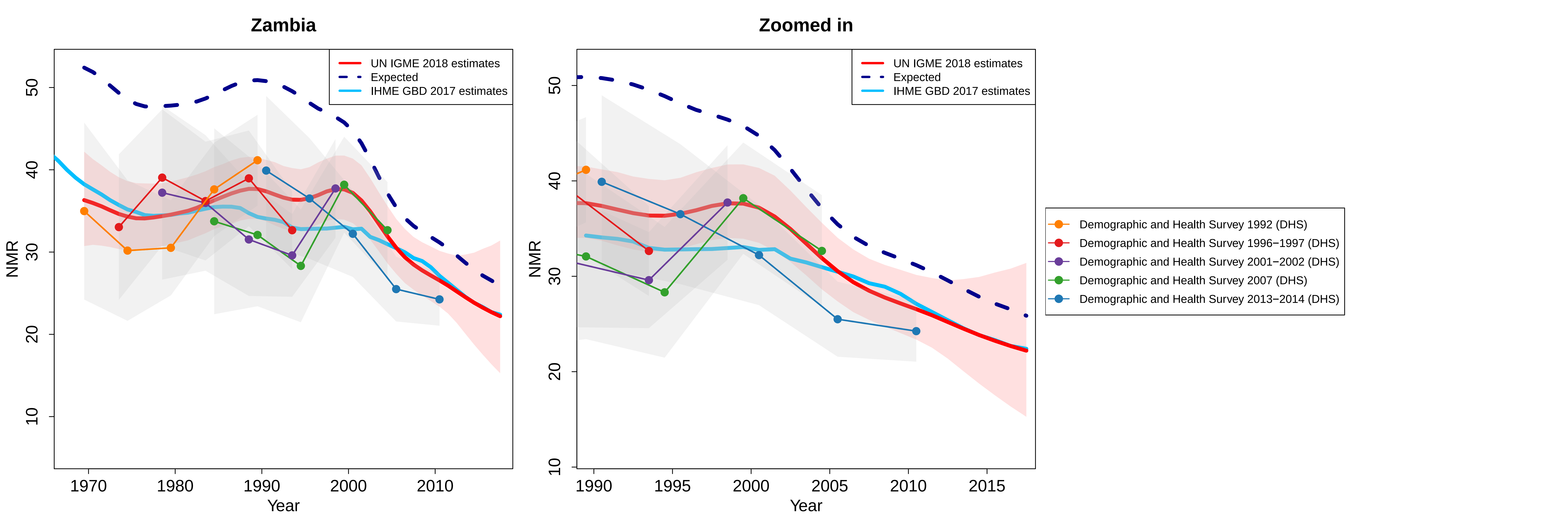
Zambia
Zoomed in
UN IGME 2018 estimates
Expected
IHME GBD 2017 estimates
NMR
Year
Demographic and Health Survey 1992 (DHS)
Demographic and Health Survey 1996−1997 (DHS)
Demographic and Health Survey 2001−2002 (DHS)
Demographic and Health Survey 2007 (DHS)
Demographic and Health Survey 2013−2014 (DHS)

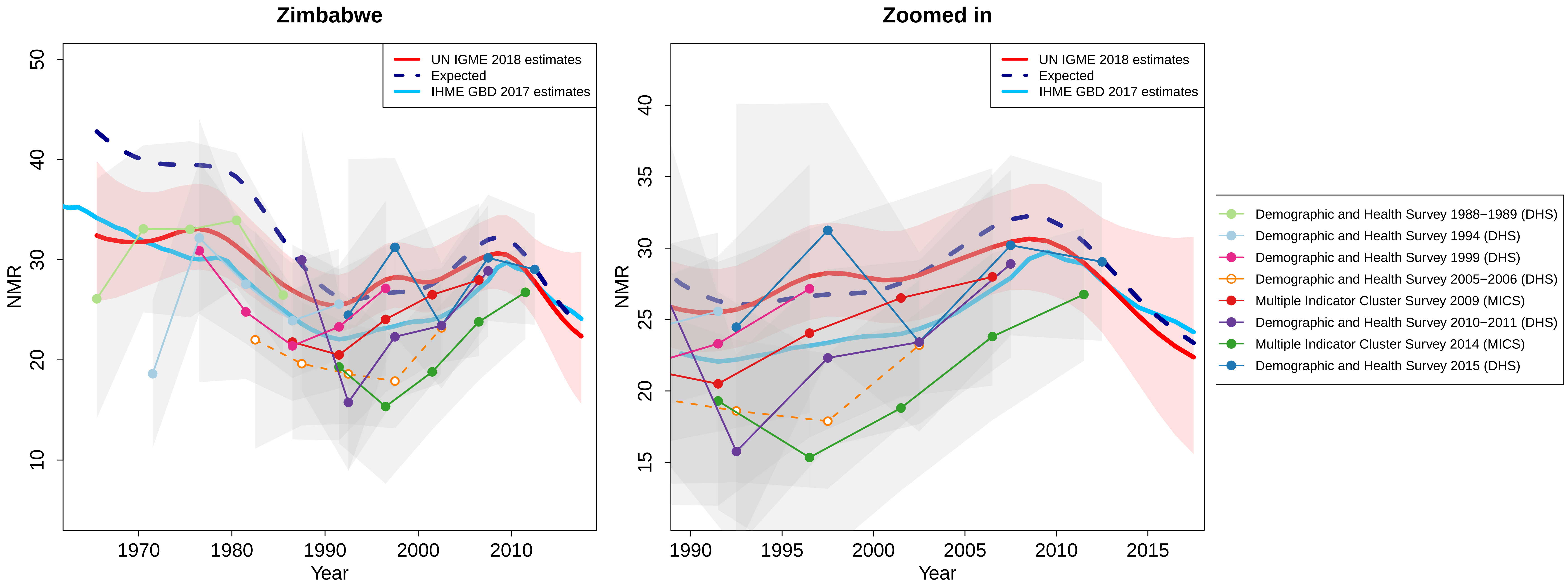
Zimbabwe
Zoomed in
NMR
Year
UN IGME 2018 estimates
Expected
IHME GBD 2017 estimates
Demographic and Health Survey 1988−1989 (DHS)
Demographic and Health Survey 1994 (DHS)
Demographic and Health Survey 1999 (DHS)
Demographic and Health Survey 2005−2006 (DHS)
Multiple Indicator Cluster Survey 2009 (MICS)
Demographic and Health Survey 2010−2011 (DHS)
Multiple Indicator Cluster Survey 2014 (MICS)
Demographic and Health Survey 2015 (DHS)

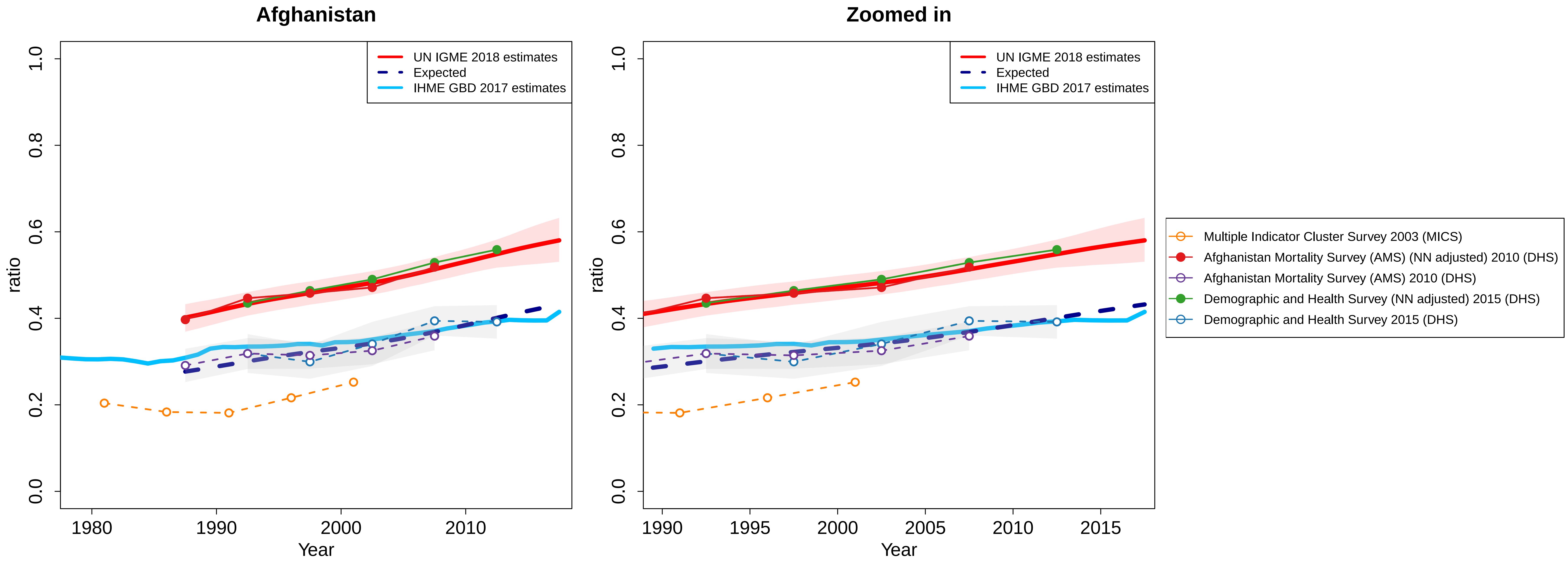

Afghanistan
Zoomed in
UN IGME 2018 estimates
Expected
IHME GBD 2017 estimates
ratio
Year
0.0
0.2
0.4
0.6
0.8
1.0
1980
1990
2000
2010
1995
2005
2015
Multiple Indicator Cluster Survey 2003 (MICS)
Afghanistan Mortality Survey (AMS) (NN adjusted) 2010 (DHS)
Afghanistan Mortality Survey (AMS) 2010 (DHS)
Demographic and Health Survey (NN adjusted) 2015 (DHS)
Demographic and Health Survey 2015 (DHS)

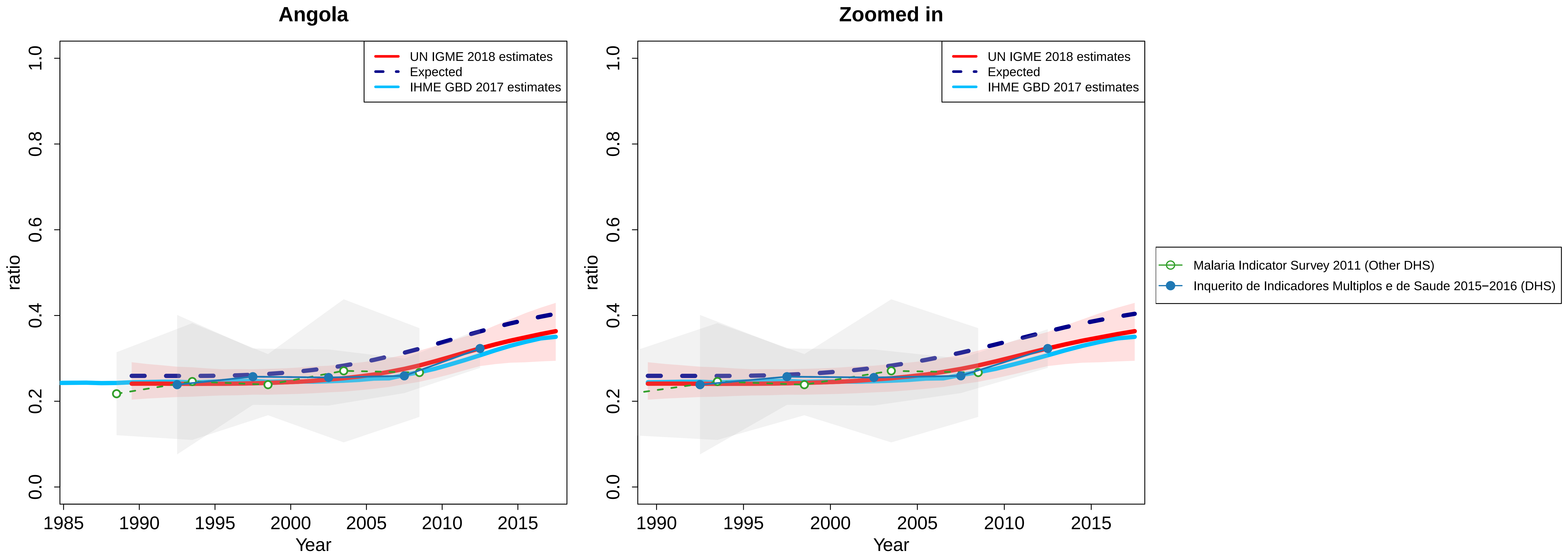
Angola
Zoomed in
UN IGME 2018 estimates
Expected
IHME GBD 2017 estimates
ratio
Year
0.0
0.2
0.4
0.6
0.8
1.0
1985
1990
1995
2000
2005
2010
2015
Malaria Indicator Survey 2011 (Other DHS)
Inquerito de Indicadores Multiplos e de Saude 2015−2016 (DHS)

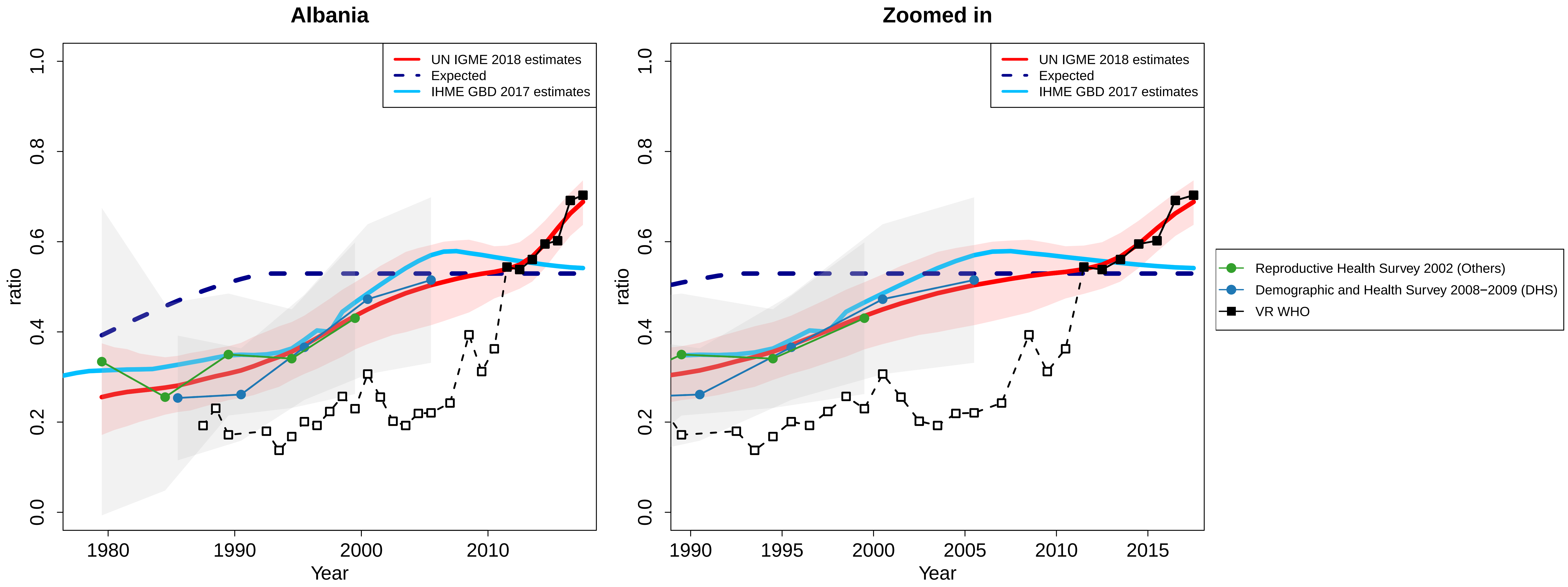
Albania
Zoomed in
UN IGME 2018 estimates
Expected
IHME GBD 2017 estimates
Reproductive Health Survey 2002 (Others)
Demographic and Health Survey 2008−2009 (DHS)
VR WHO
ratio
Year
0.0
0.2
0.4
0.6
0.8
1.0
1980
1990
2000
2010
1995
2005
2015

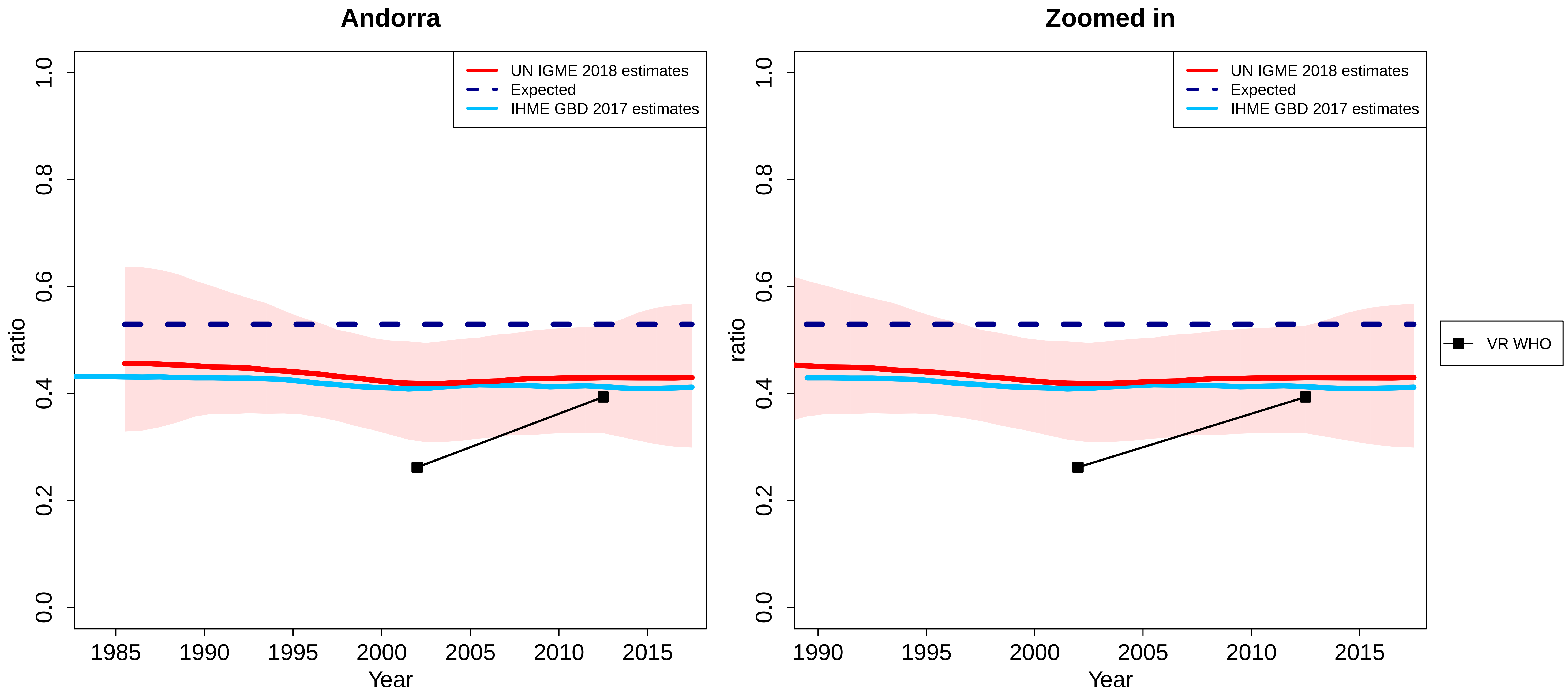
Andorra
Zoomed in
UN IGME 2018 estimates
Expected
IHME GBD 2017 estimates
VR WHO
ratio
Year
0.0
0.2
0.4
0.6
0.8
1.0
1985
1990
1995
2000
2005
2010
2015

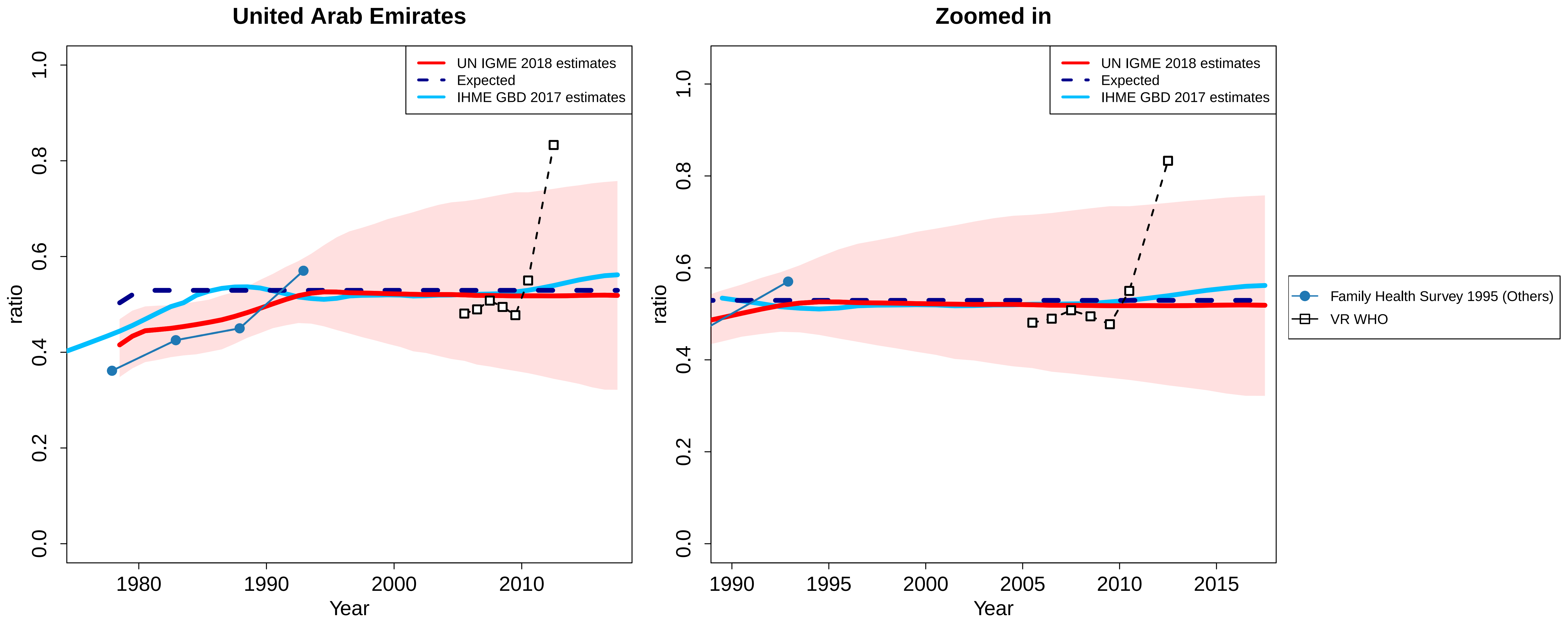
United Arab Emirates
Zoomed in
UN IGME 2018 estimates
Expected
IHME GBD 2017 estimates
Family Health Survey 1995 (Others)
VR WHO
ratio
Year
0.0
0.2
0.4
0.6
0.8
1.0
1980
1990
2000
2010
1995
2005
2015

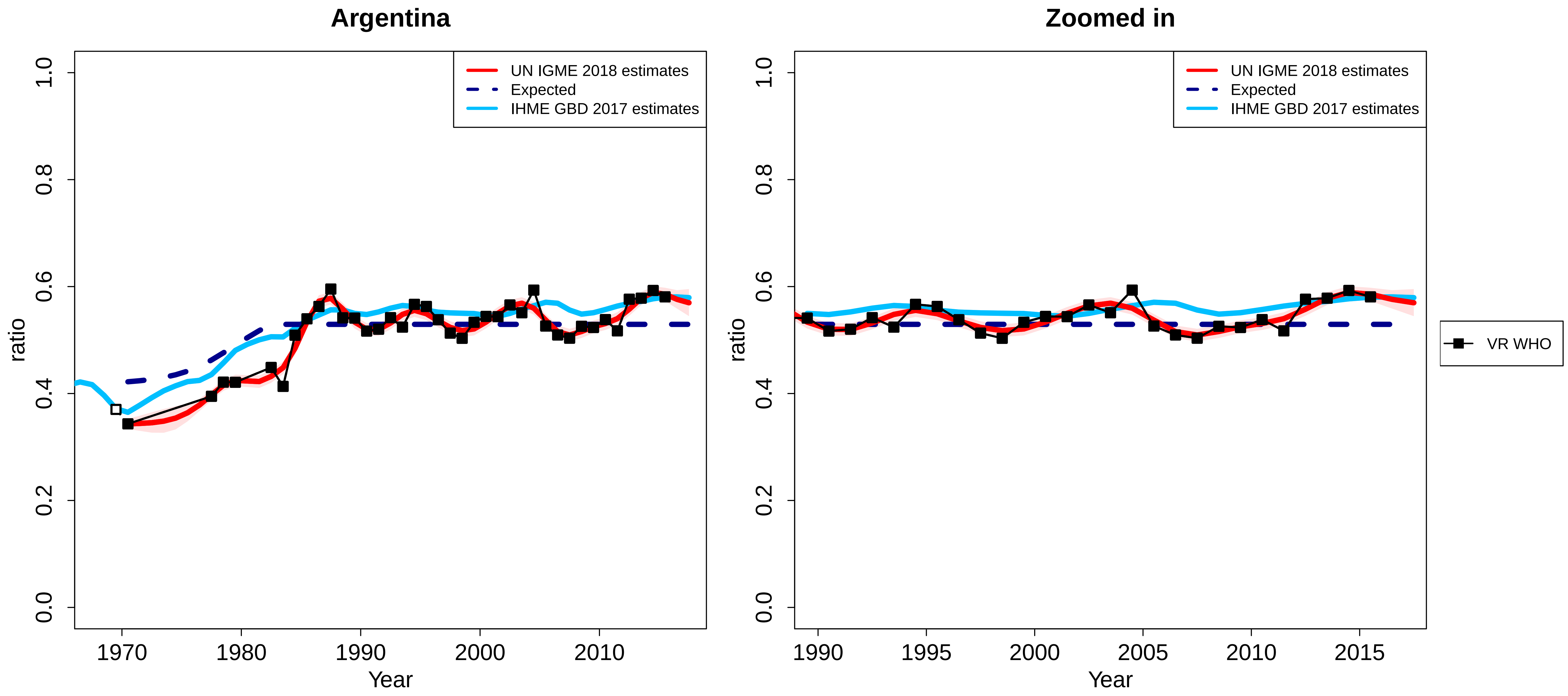

Argentina
Zoomed in
UN IGME 2018 estimates
Expected
IHME GBD 2017 estimates
VR WHO
ratio
Year
0.0
0.2
0.4
0.6
0.8
1.0
1970
1980
1990
2000
2010
1995
2005
2015

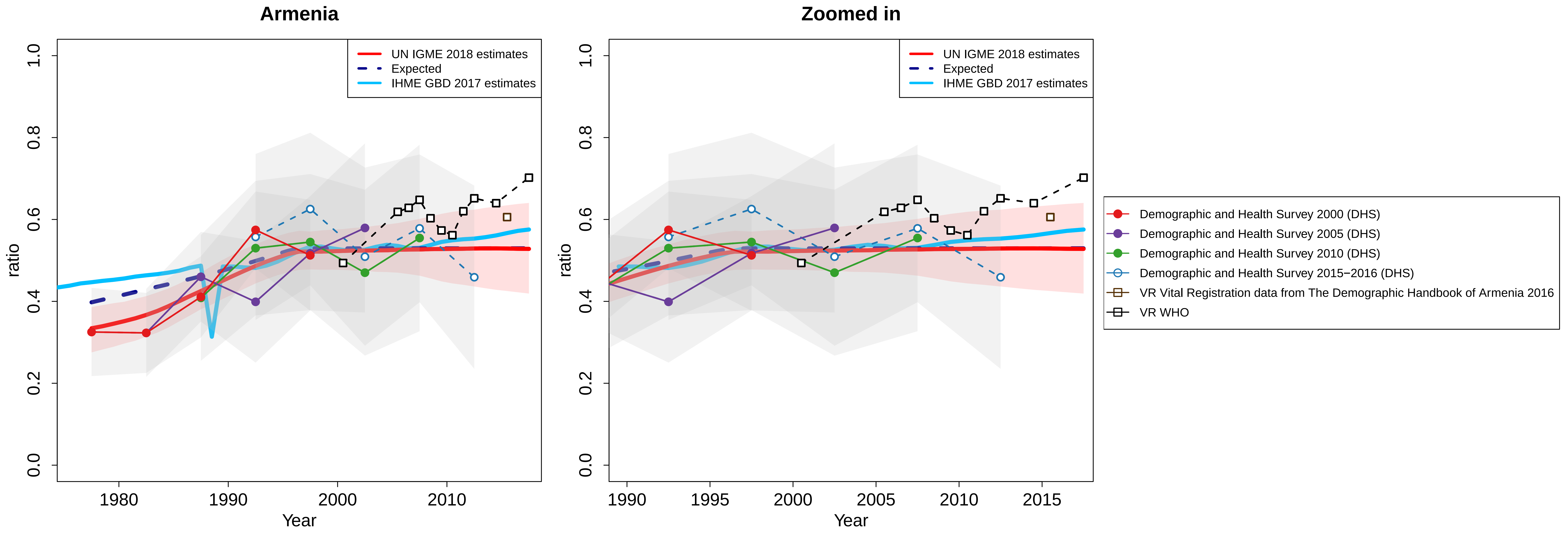
Armenia
Zoomed in
UN IGME 2018 estimates
Expected
IHME GBD 2017 estimates
ratio
Year
0.0
0.2
0.4
0.6
0.8
1.0
1980
1990
2000
2010
1995
2005
2015
Demographic and Health Survey 2000 (DHS)
Demographic and Health Survey 2005 (DHS)
Demographic and Health Survey 2010 (DHS)
Demographic and Health Survey 2015–2016 (DHS)
VR Vital Registration data from The Demographic Handbook of Armenia 2016
VR WHO

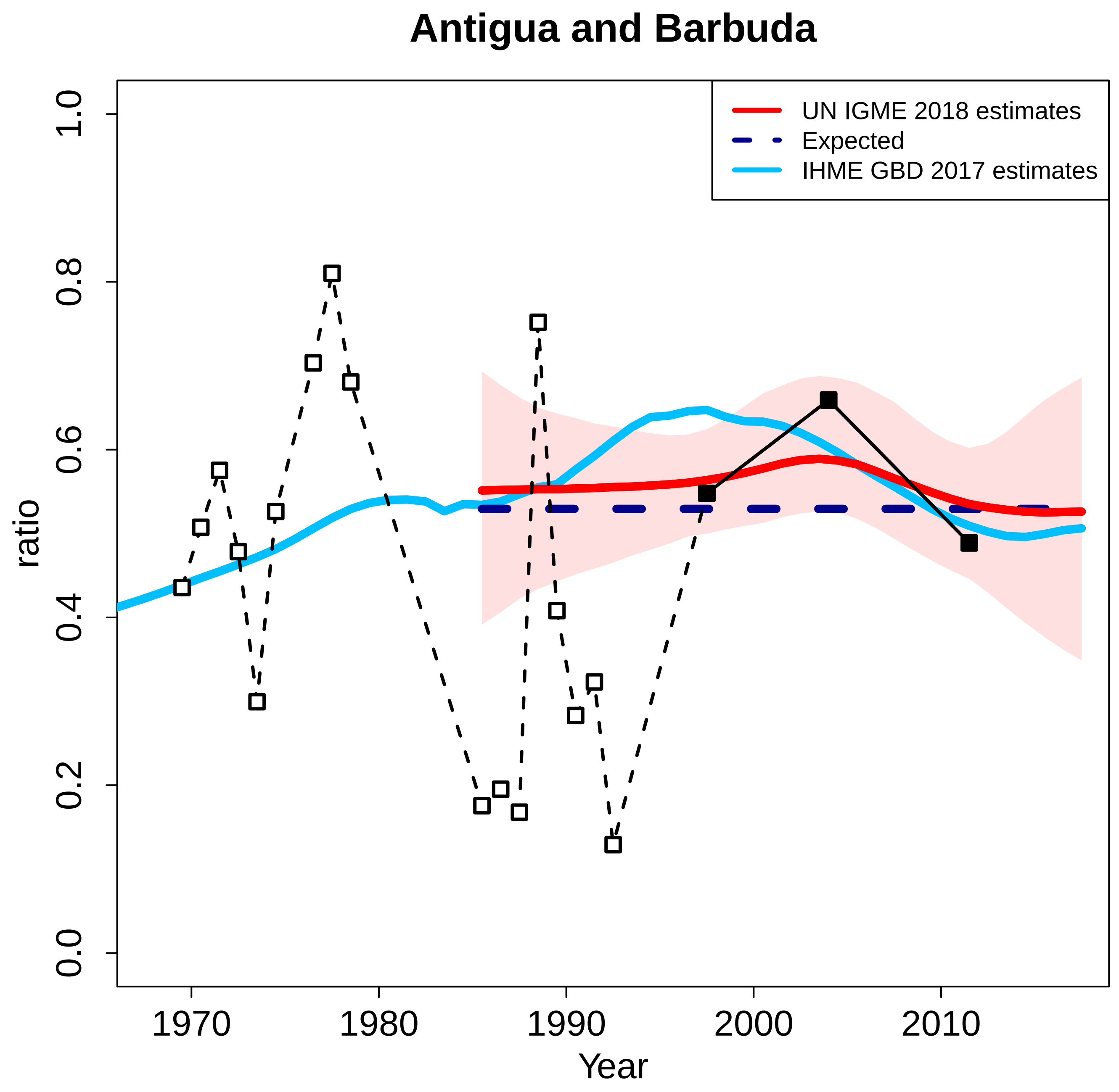

Antigua and Barbuda
ratio
Year
UN IGME 2018 estimates
Expected
IHME GBD 2017 estimates
0.0
0.2
0.4
0.6
0.8
1.0
1970
1980
1990
2000
2010

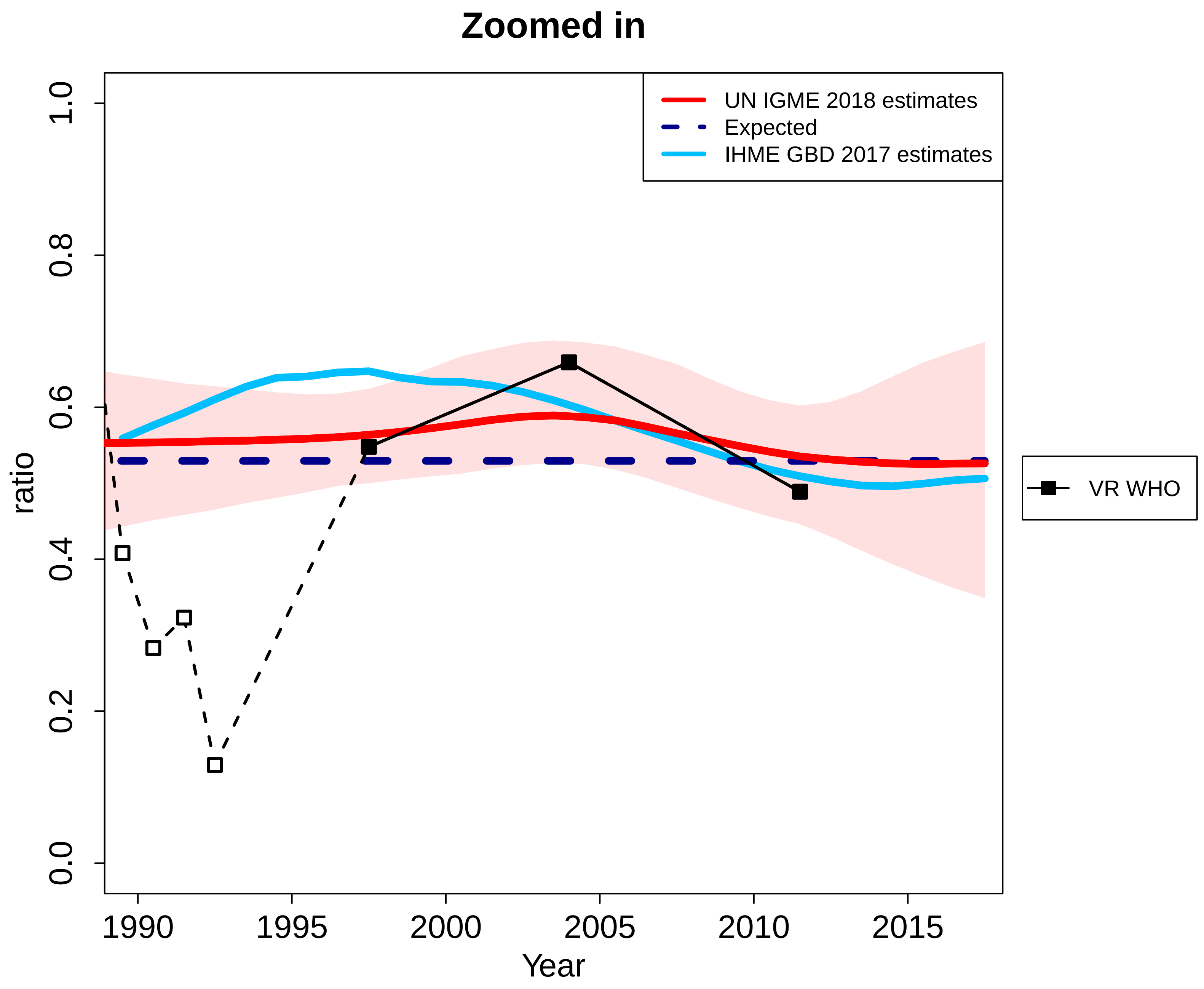

Zoomed in
ratio
Year
UN IGME 2018 estimates
Expected
IHME GBD 2017 estimates
VR WHO
0.0
0.2
0.4
0.6
0.8
1.0
1990
1995
2000
2005
2010
2015

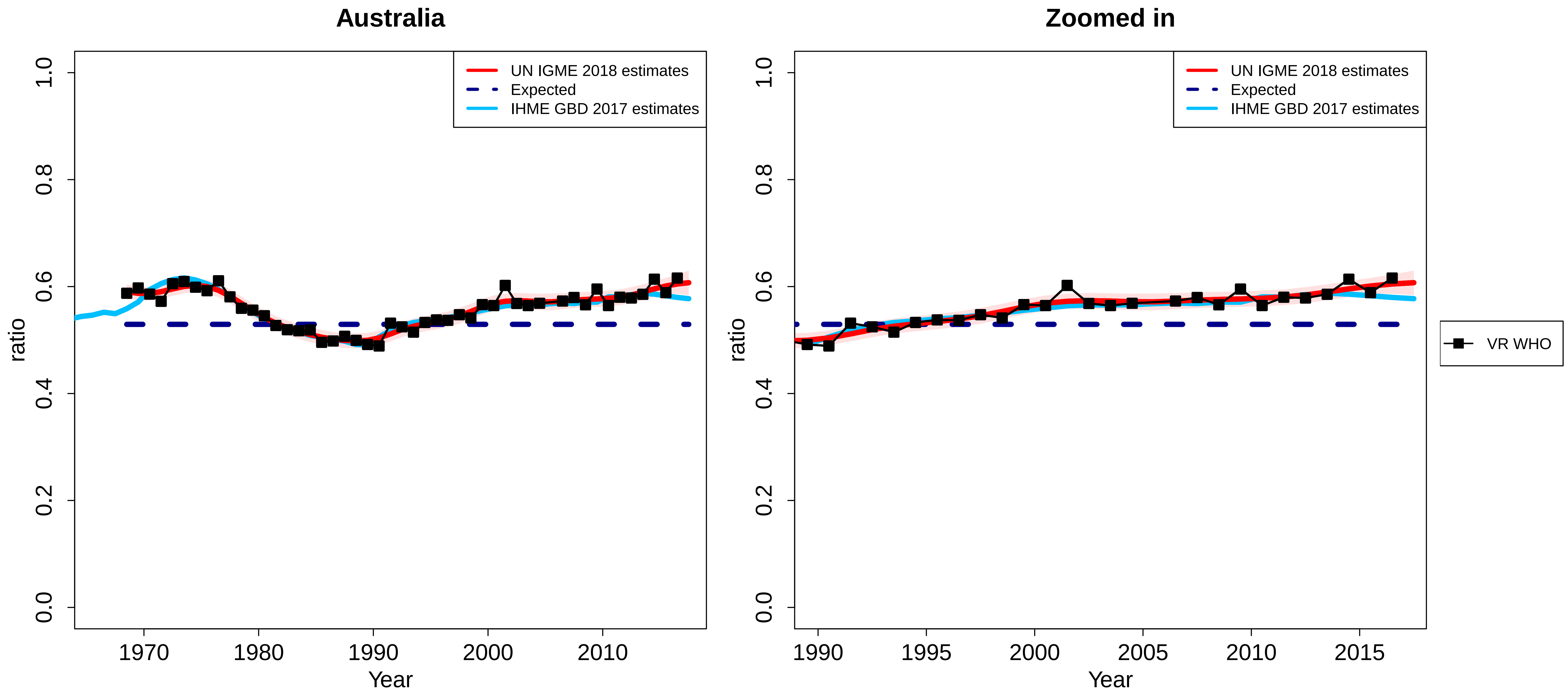
Australia
Zoomed in
UN IGME 2018 estimates
Expected
IHME GBD 2017 estimates
ratio
0.0
0.2
0.4
0.6
0.8
1.0
1970
1980
1990
2000
2010
Year
1990
1995
2000
2005
2010
2015
VR WHO

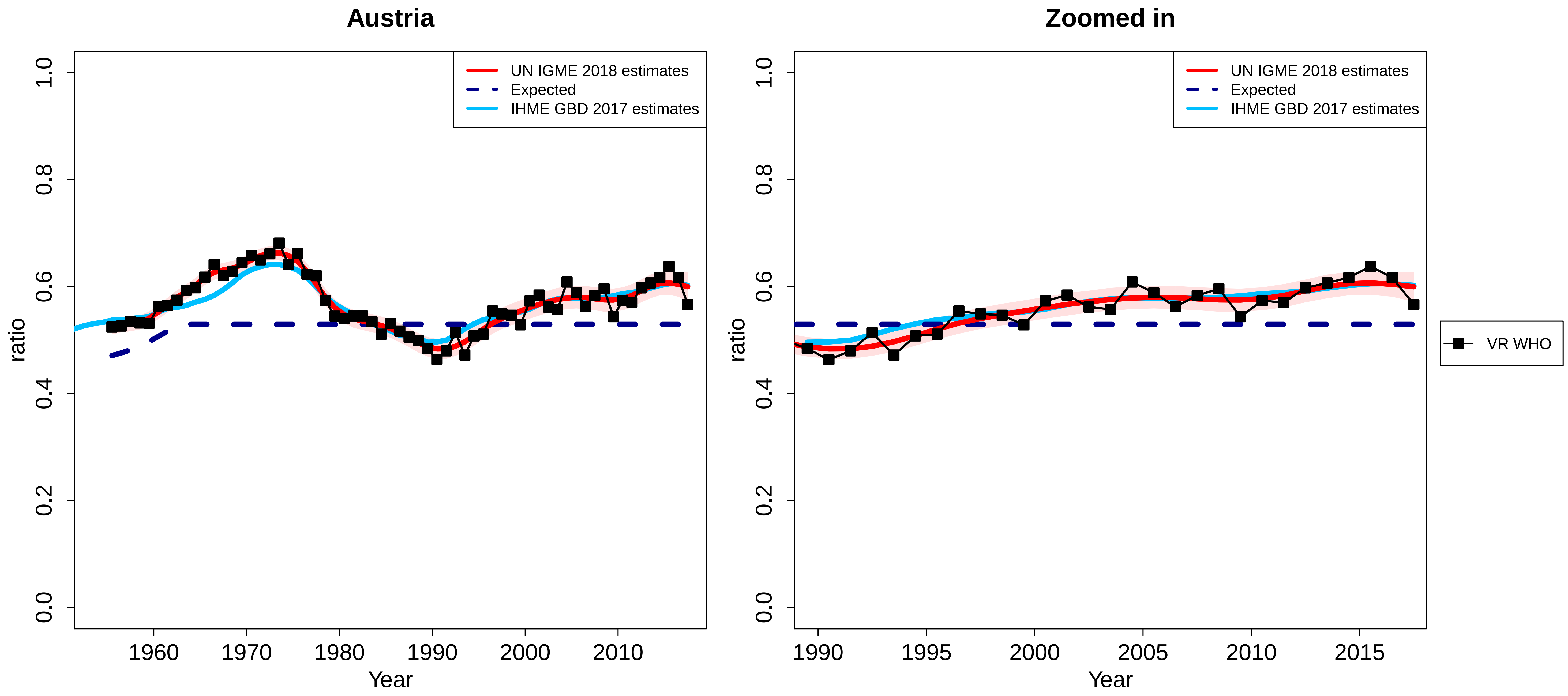

Austria
Zoomed in
UN IGME 2018 estimates
Expected
IHME GBD 2017 estimates
ratio
Year
0.0
0.2
0.4
0.6
0.8
1.0
1960
1970
1980
1990
2000
2010
1995
2005
2015
VR WHO

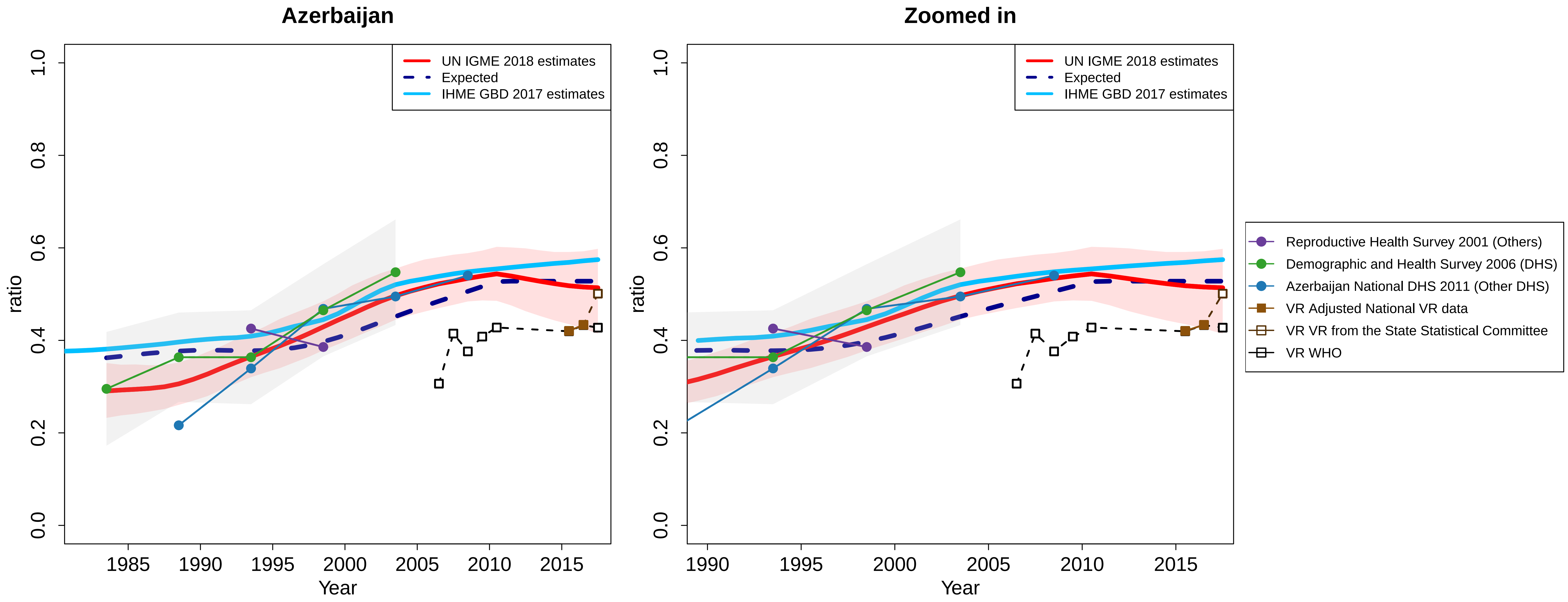

Azerbaijan
Zoomed in
UN IGME 2018 estimates
Expected
IHME GBD 2017 estimates
ratio
Year
Reproductive Health Survey 2001 (Others)
Demographic and Health Survey 2006 (DHS)
Azerbaijan National DHS 2011 (Other DHS)
VR Adjusted National VR data
VR VR from the State Statistical Committee
VR WHO

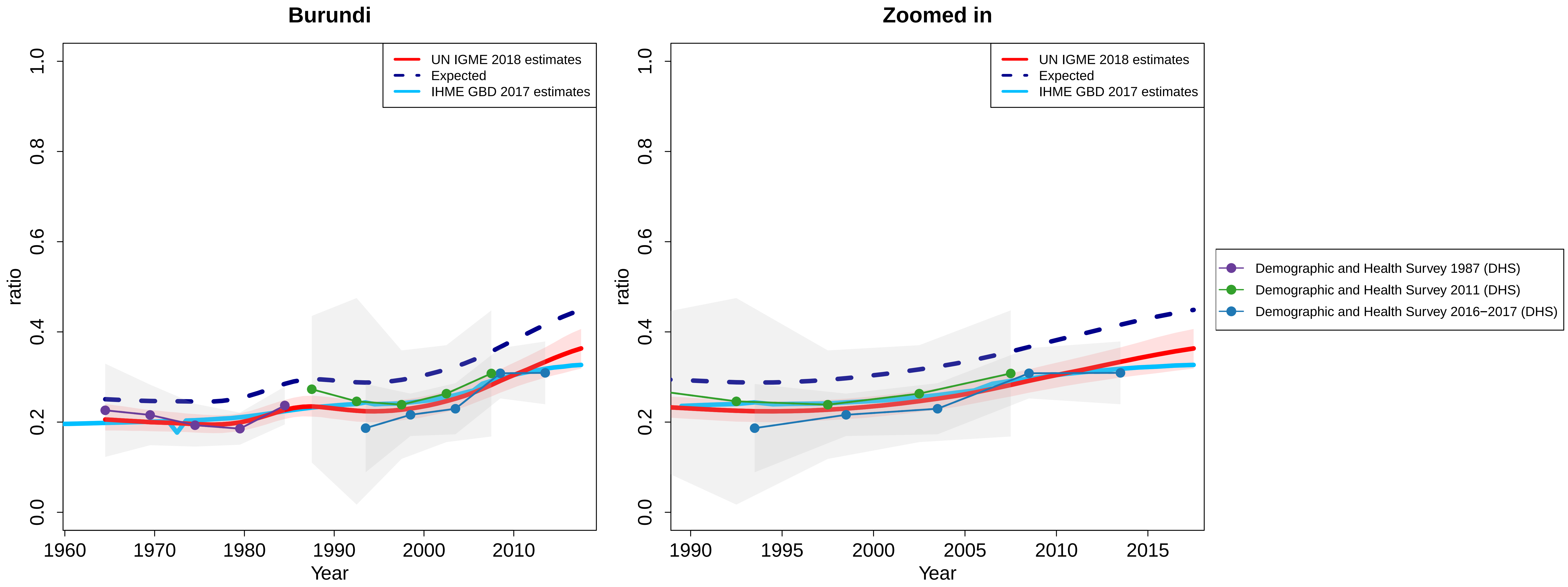

Burundi
Zoomed in
UN IGME 2018 estimates
Expected
IHME GBD 2017 estimates
ratio
Year
1960
1970
1980
1990
2000
2010
1995
2005
2015
0.0
0.2
0.4
0.6
0.8
1.0
Demographic and Health Survey 1987 (DHS)
Demographic and Health Survey 2011 (DHS)
Demographic and Health Survey 2016–2017 (DHS)

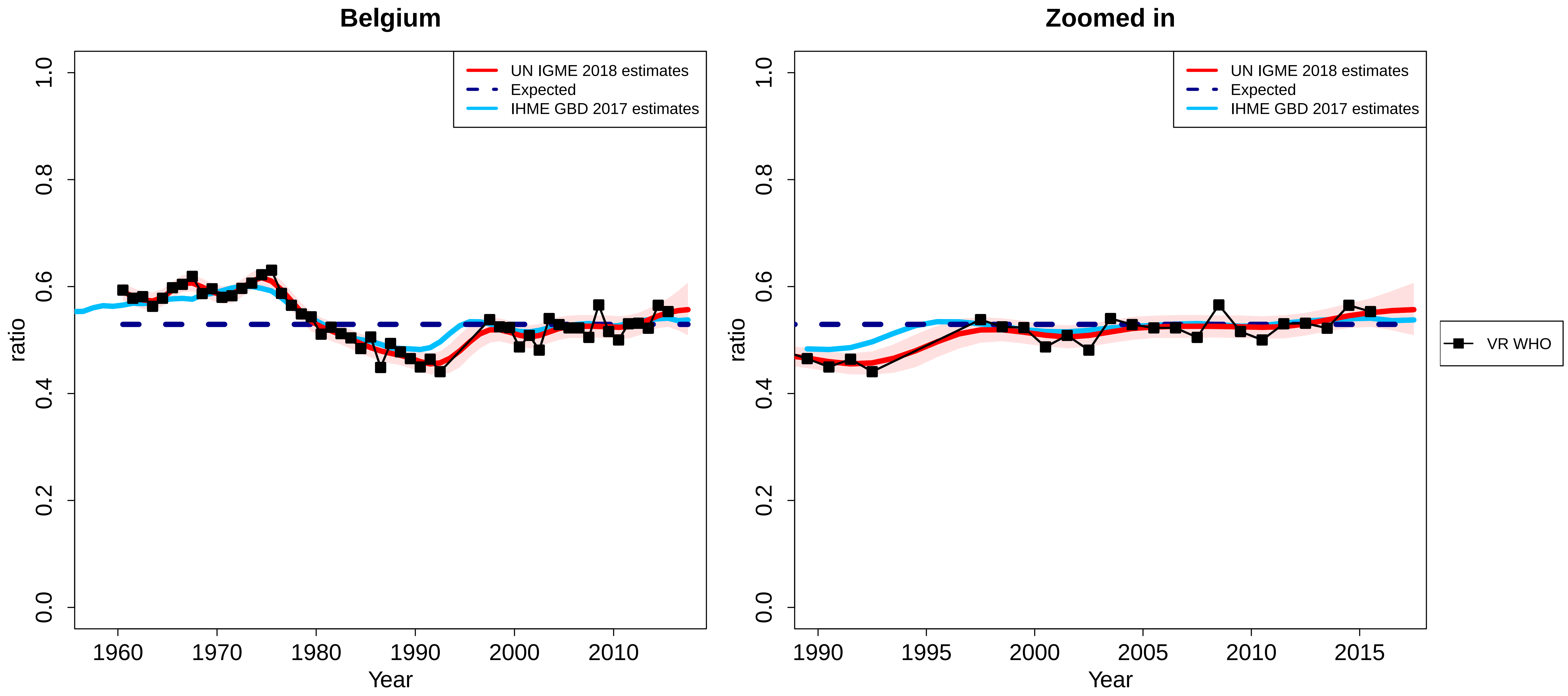

Belgium
Zoomed in
UN IGME 2018 estimates
Expected
IHME GBD 2017 estimates
VR WHO
ratio
Year
0.0
0.2
0.4
0.6
0.8
1.0
1960
1970
1980
1990
2000
2010
1990
1995
2000
2005
2010
2015

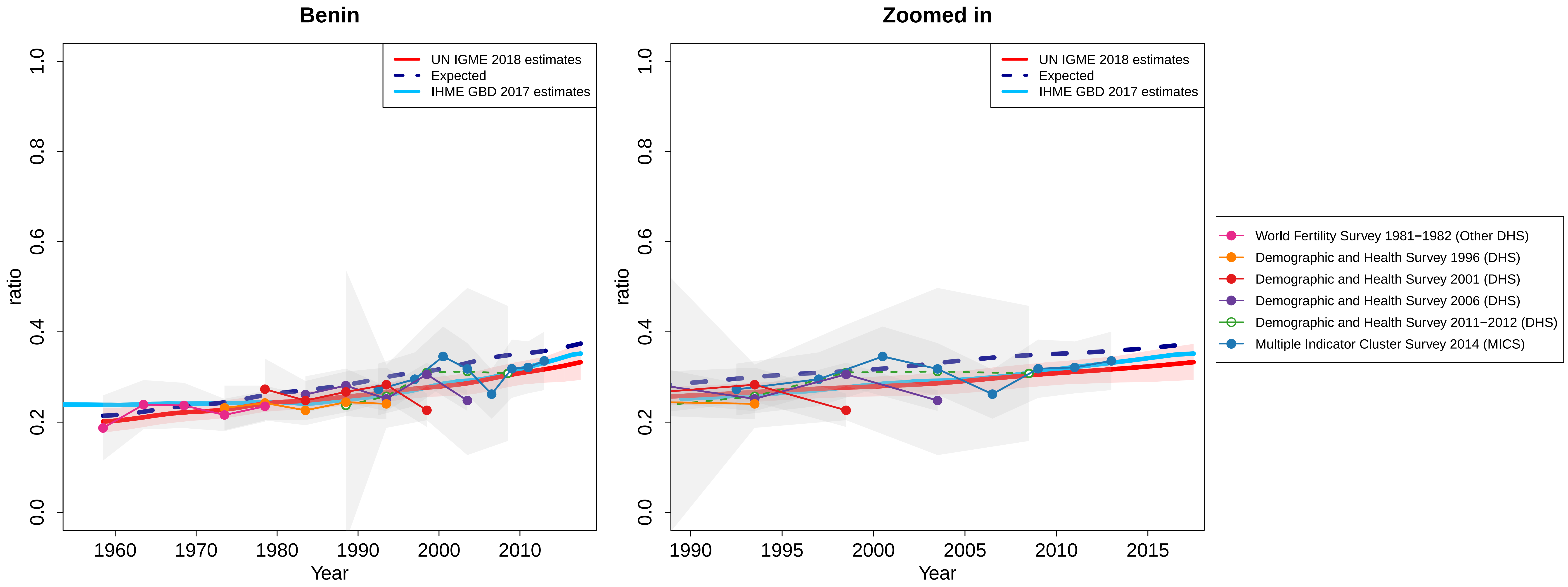

Benin
Zoomed in
ratio
Year
UN IGME 2018 estimates
Expected
IHME GBD 2017 estimates
World Fertility Survey 1981−1982 (Other DHS)
Demographic and Health Survey 1996 (DHS)
Demographic and Health Survey 2001 (DHS)
Demographic and Health Survey 2006 (DHS)
Demographic and Health Survey 2011−2012 (DHS)
Multiple Indicator Cluster Survey 2014 (MICS)

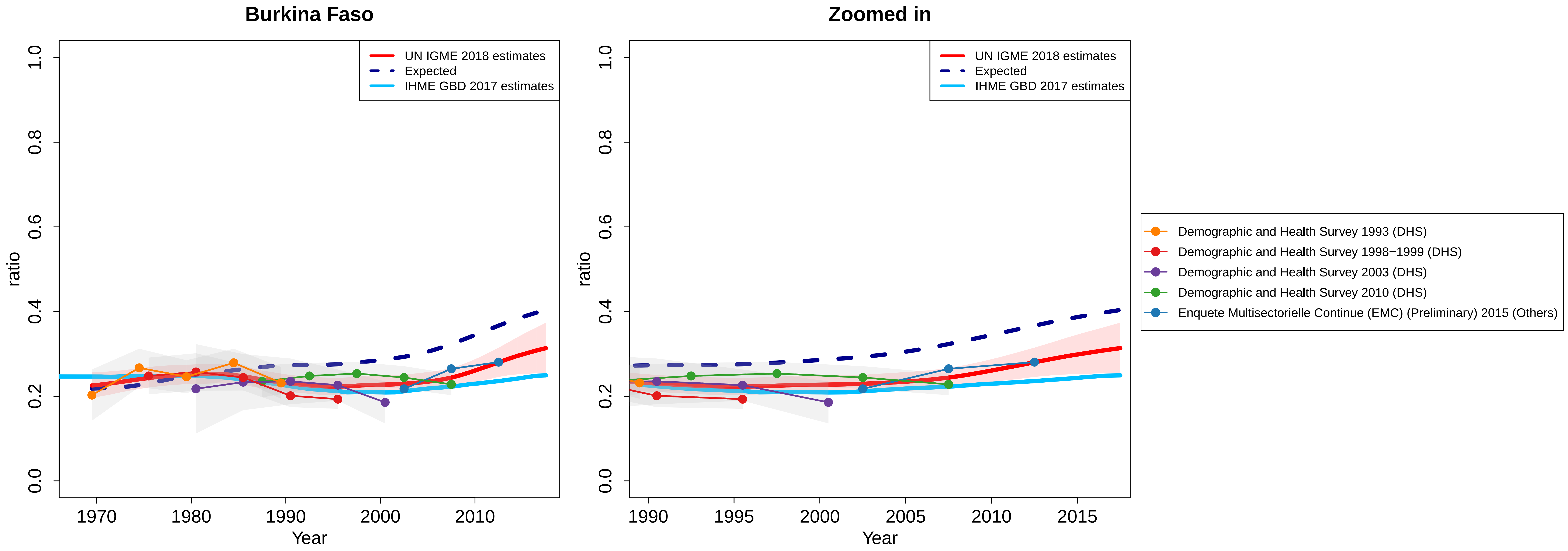
Burkina Faso
Zoomed in
ratio
Year
UN IGME 2018 estimates
Expected
IHME GBD 2017 estimates
0.0
0.2
0.4
0.6
0.8
1.0
1970
1980
1990
2000
2010
1995
2005
2015
Demographic and Health Survey 1993 (DHS)
Demographic and Health Survey 1998−1999 (DHS)
Demographic and Health Survey 2003 (DHS)
Demographic and Health Survey 2010 (DHS)
Enquete Multisectorielle Continue (EMC) (Preliminary) 2015 (Others)

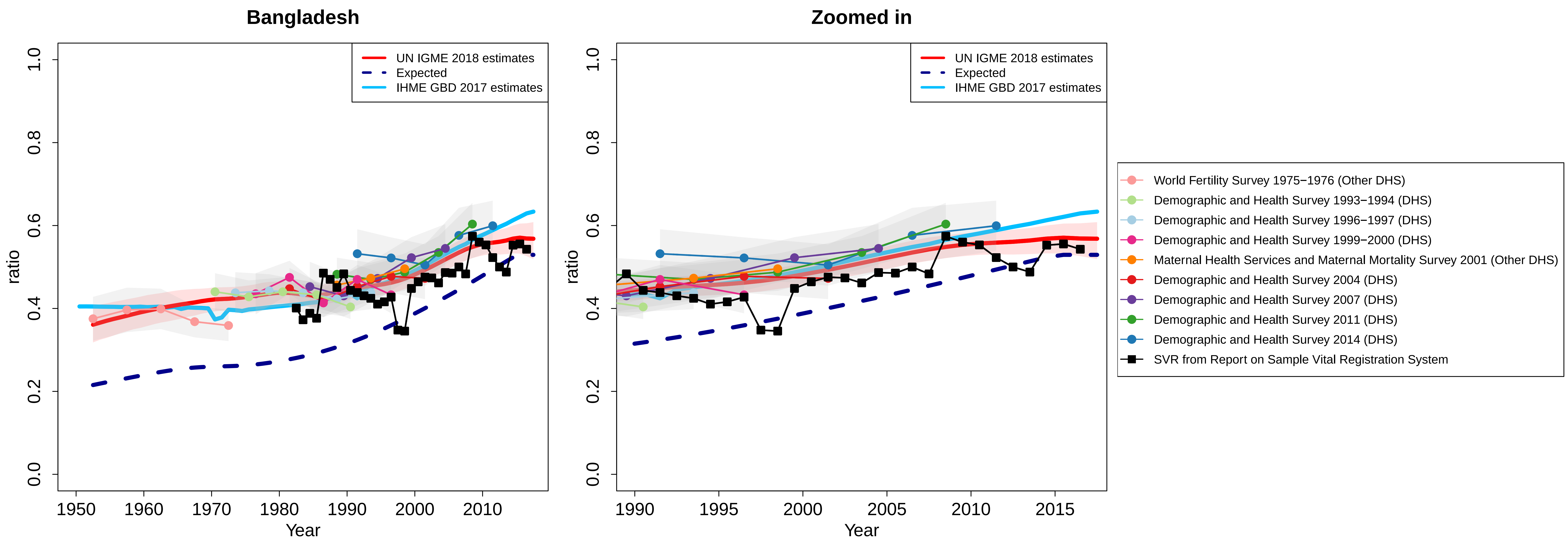

Bangladesh
Zoomed in
UN IGME 2018 estimates
Expected
IHME GBD 2017 estimates
ratio
Year
0.0
0.2
0.4
0.6
0.8
1.0
1950
1960
1970
1980
1990
2000
2010
1995
2005
2015
World Fertility Survey 1975−1976 (Other DHS)
Demographic and Health Survey 1993−1994 (DHS)
Demographic and Health Survey 1996−1997 (DHS)
Demographic and Health Survey 1999−2000 (DHS)
Maternal Health Services and Maternal Mortality Survey 2001 (Other DHS)
Demographic and Health Survey 2004 (DHS)
Demographic and Health Survey 2007 (DHS)
Demographic and Health Survey 2011 (DHS)
Demographic and Health Survey 2014 (DHS)
SVR from Report on Sample Vital Registration System

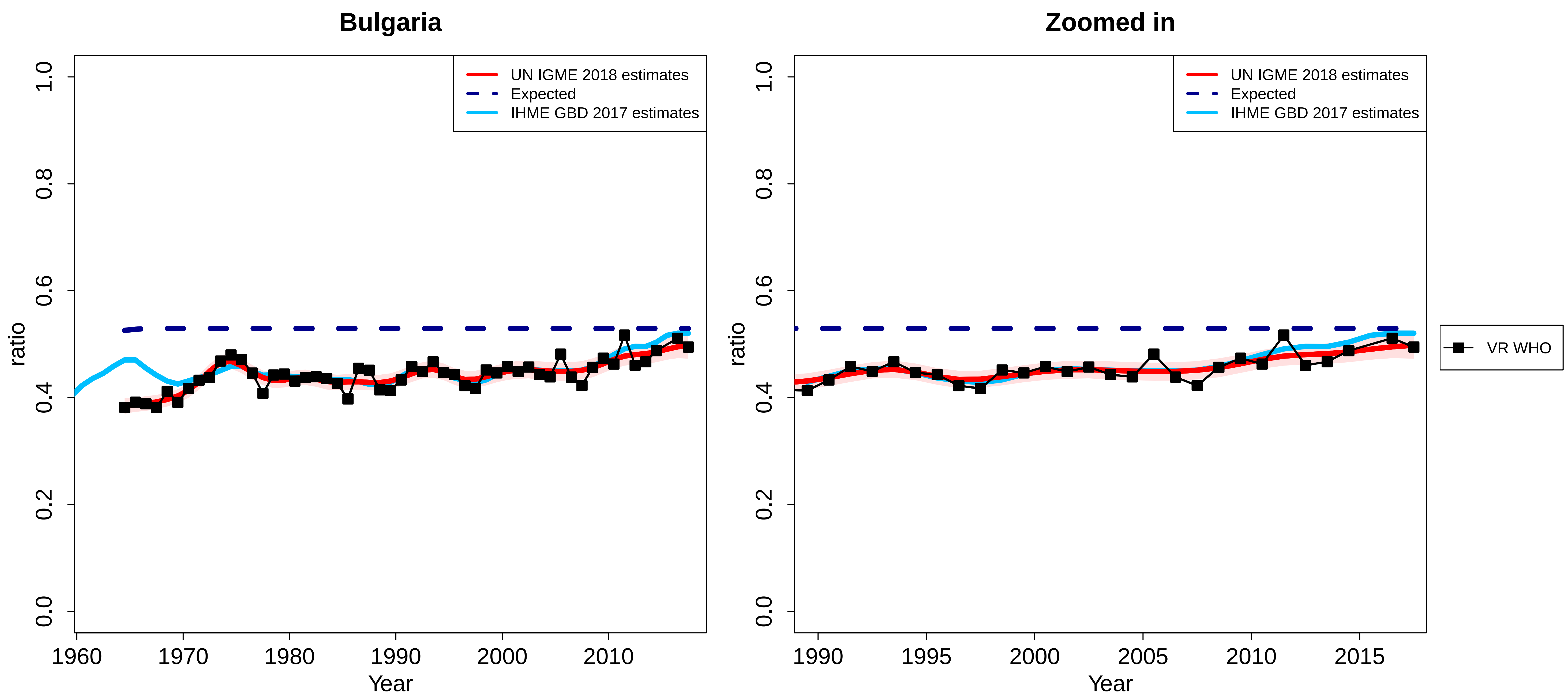

Bulgaria
Zoomed in
UN IGME 2018 estimates
Expected
IHME GBD 2017 estimates
VR WHO
ratio
Year
0.0
0.2
0.4
0.6
0.8
1.0
1960
1970
1980
1990
2000
2010
1995
2005
2015

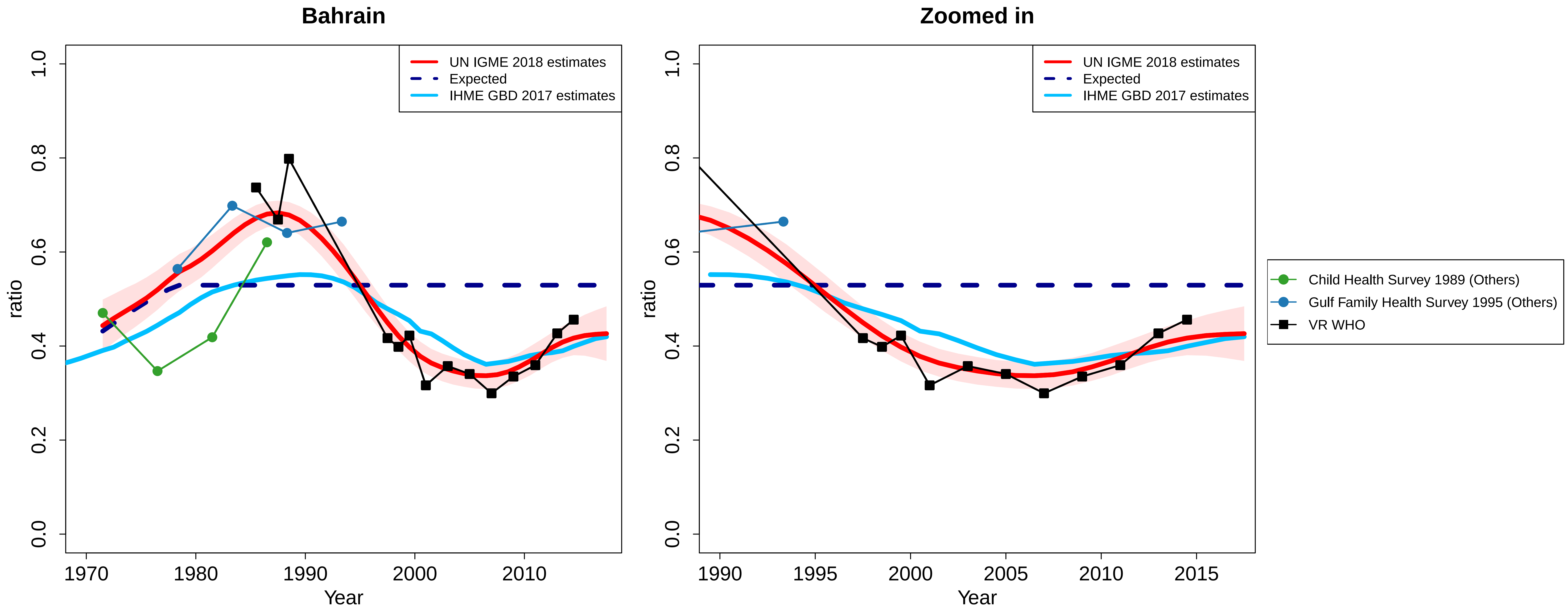

Bahrain
Zoomed in
UN IGME 2018 estimates
Expected
IHME GBD 2017 estimates
Child Health Survey 1989 (Others)
Gulf Family Health Survey 1995 (Others)
VR WHO
ratio
Year
0.0
0.2
0.4
0.6
0.8
1.0
1970
1980
1990
2000
2010
1995
2005
2015

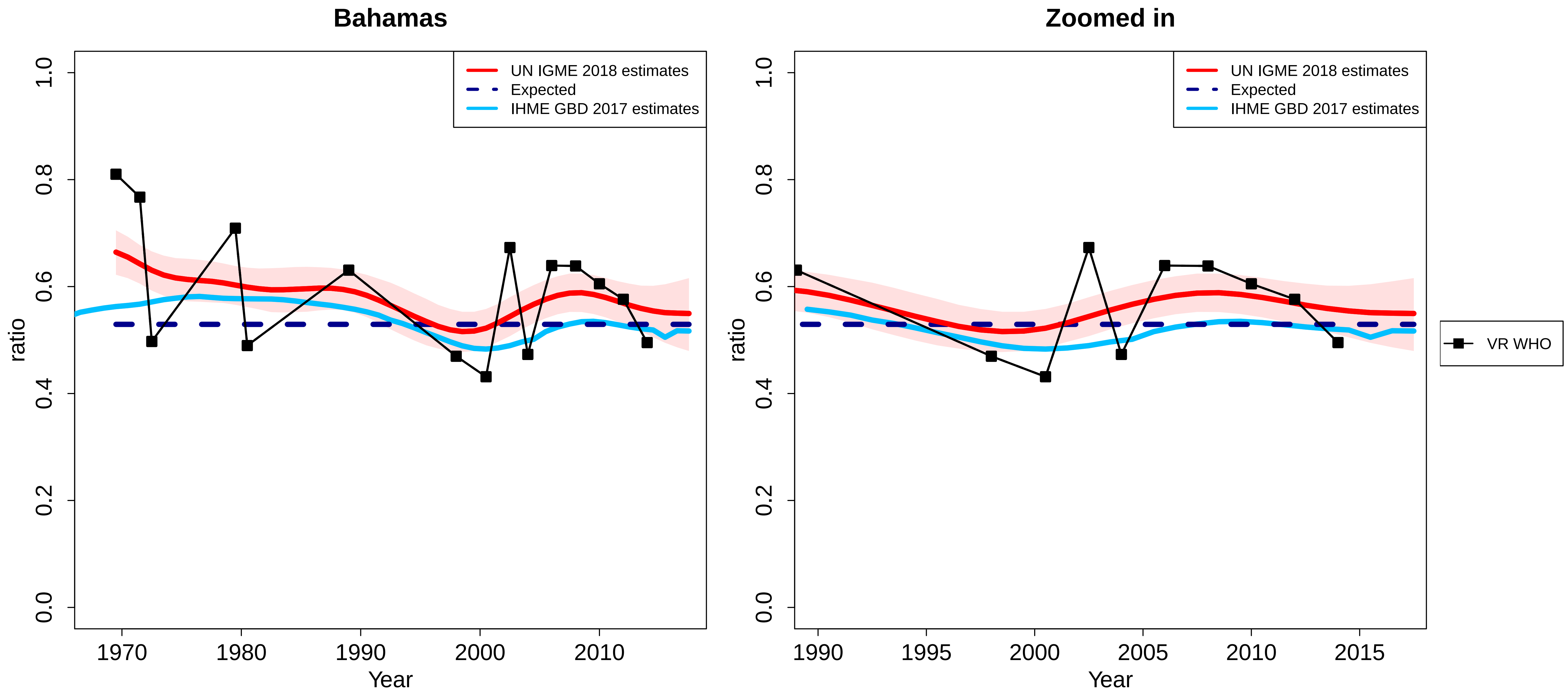
Bahamas
Zoomed in
UN IGME 2018 estimates
Expected
IHME GBD 2017 estimates
VR WHO
ratio
Year
0.0
0.2
0.4
0.6
0.8
1.0
1970
1980
1990
2000
2010
1995
2005
2015

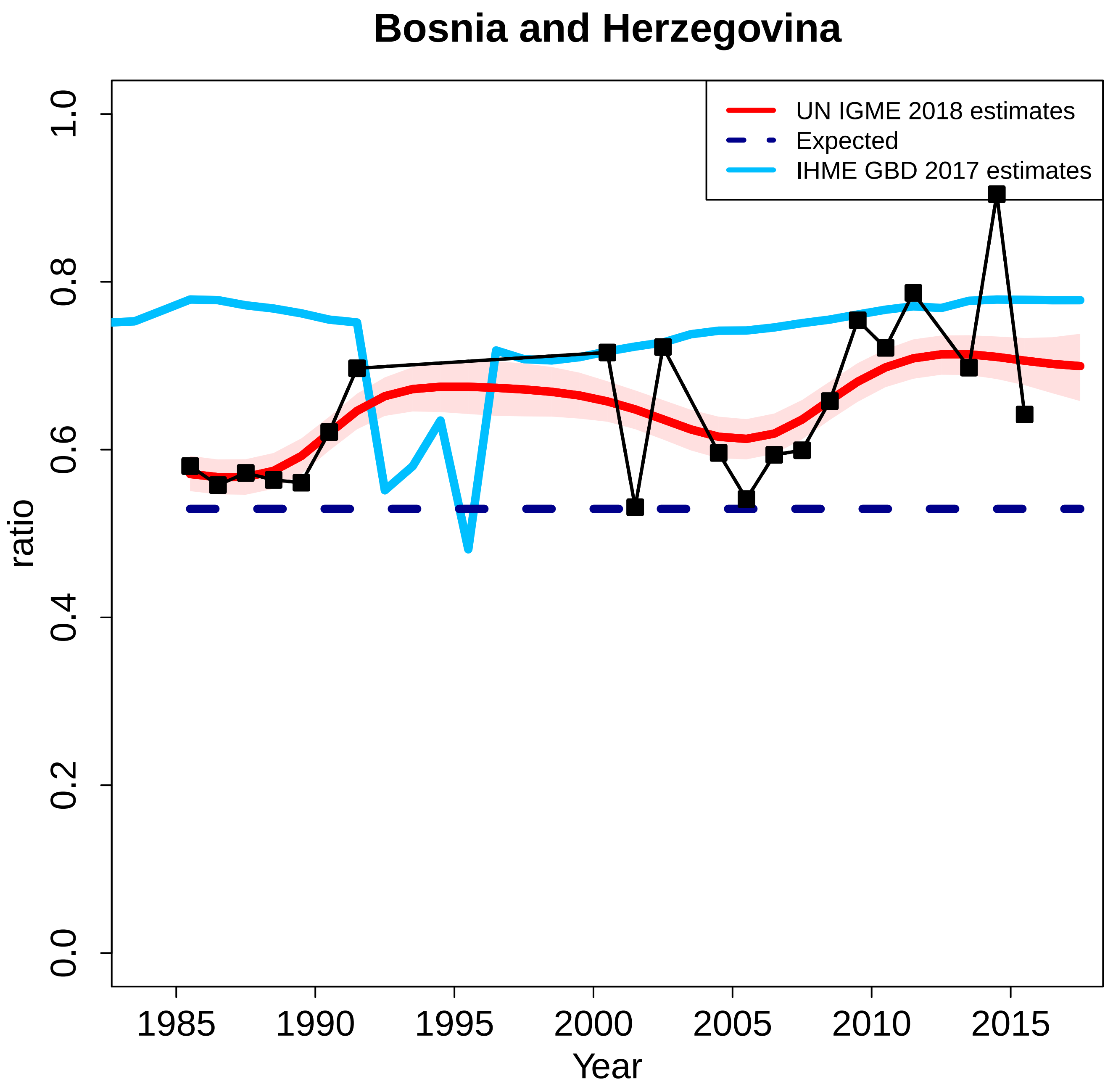

Bosnia and Herzegovina
UN IGME 2018 estimates
Expected
IHME GBD 2017 estimates
ratio
0.0
0.2
0.4
0.6
0.8
1.0
1985
1990
1995
2000
2005
2010
2015
Year

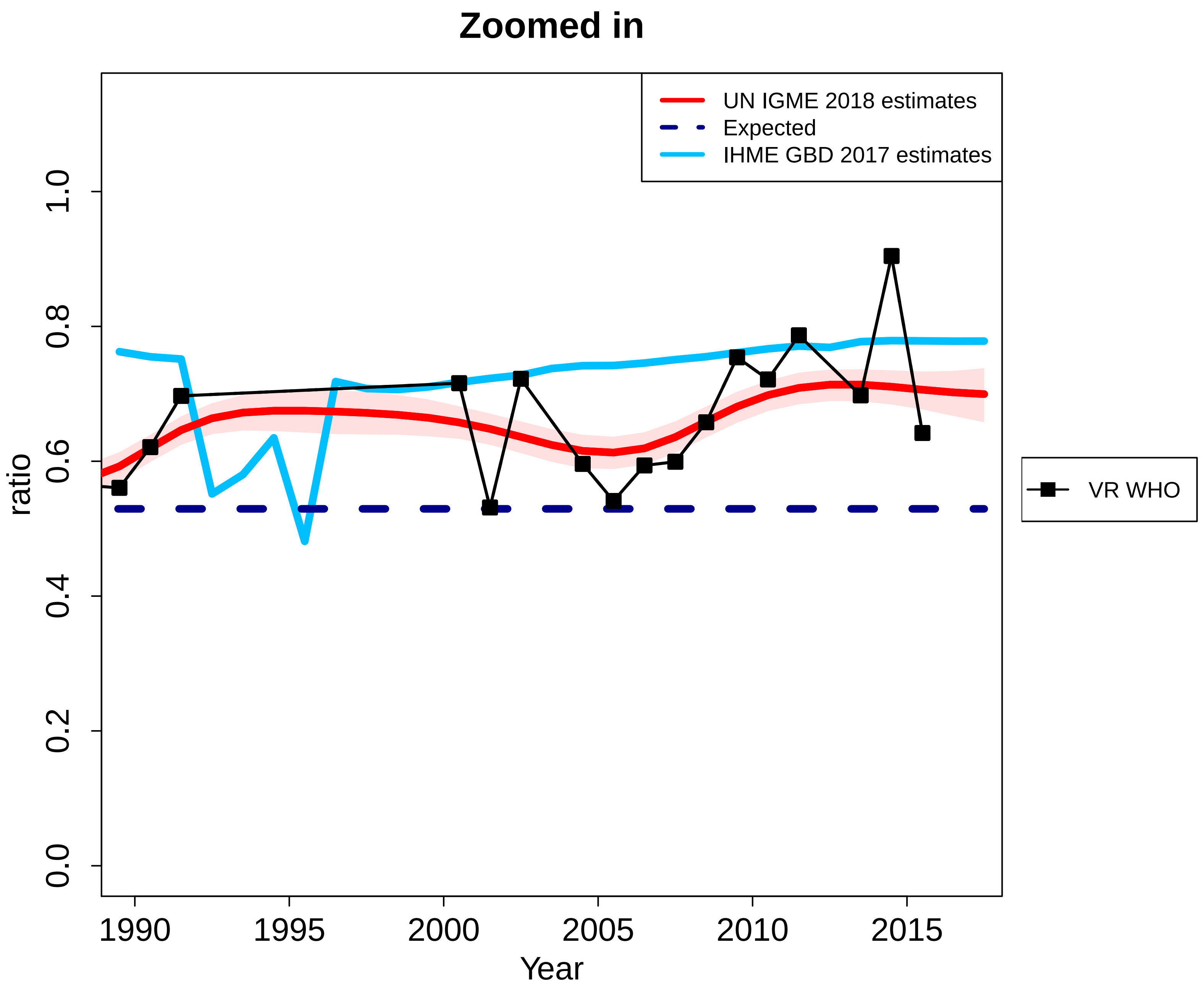

Zoomed in
UN IGME 2018 estimates
Expected
IHME GBD 2017 estimates
ratio
0.0
0.2
0.4
0.6
0.8
1.0
1990
1995
2000
2005
2010
2015
Year
VR WHO

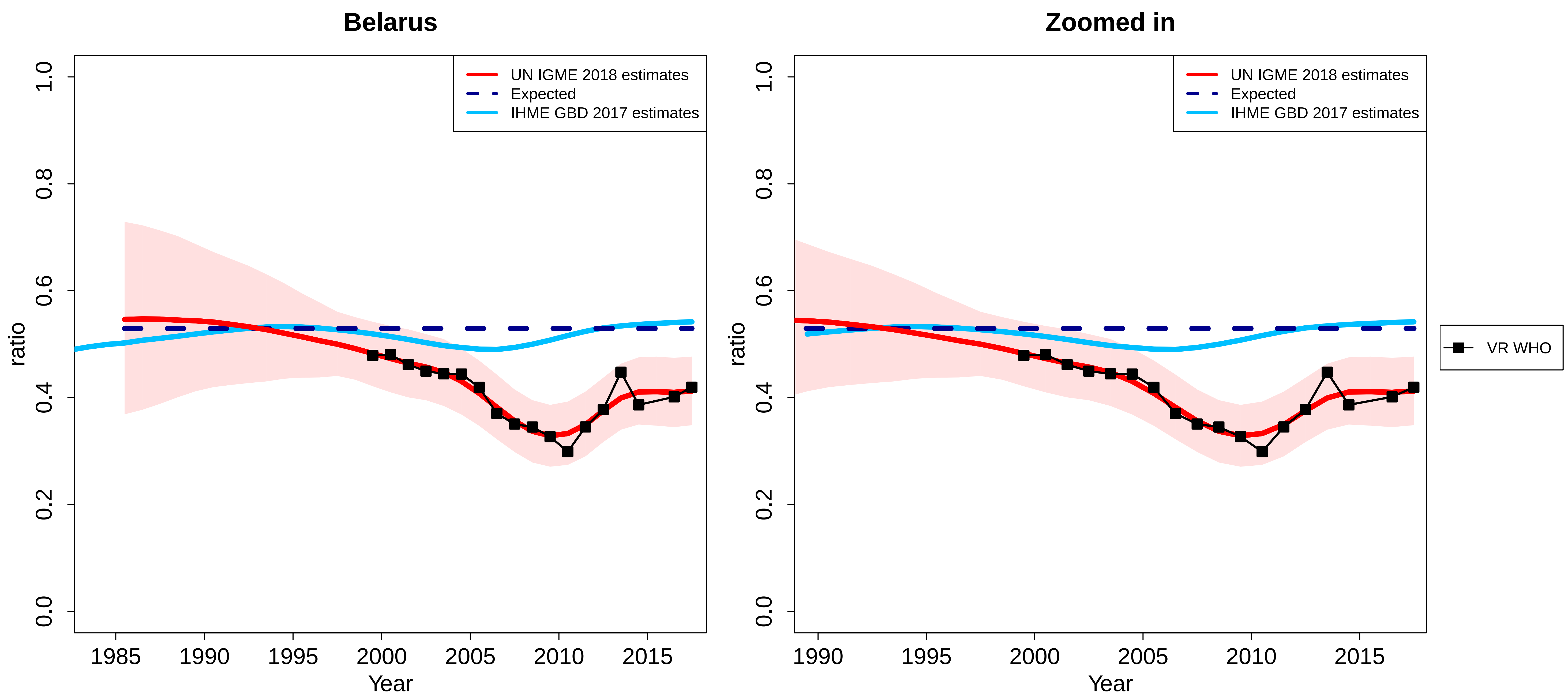

Belarus
Zoomed in
UN IGME 2018 estimates
Expected
IHME GBD 2017 estimates
VR WHO
ratio
Year
0.0
0.2
0.4
0.6
0.8
1.0
1985
1990
1995
2000
2005
2010
2015

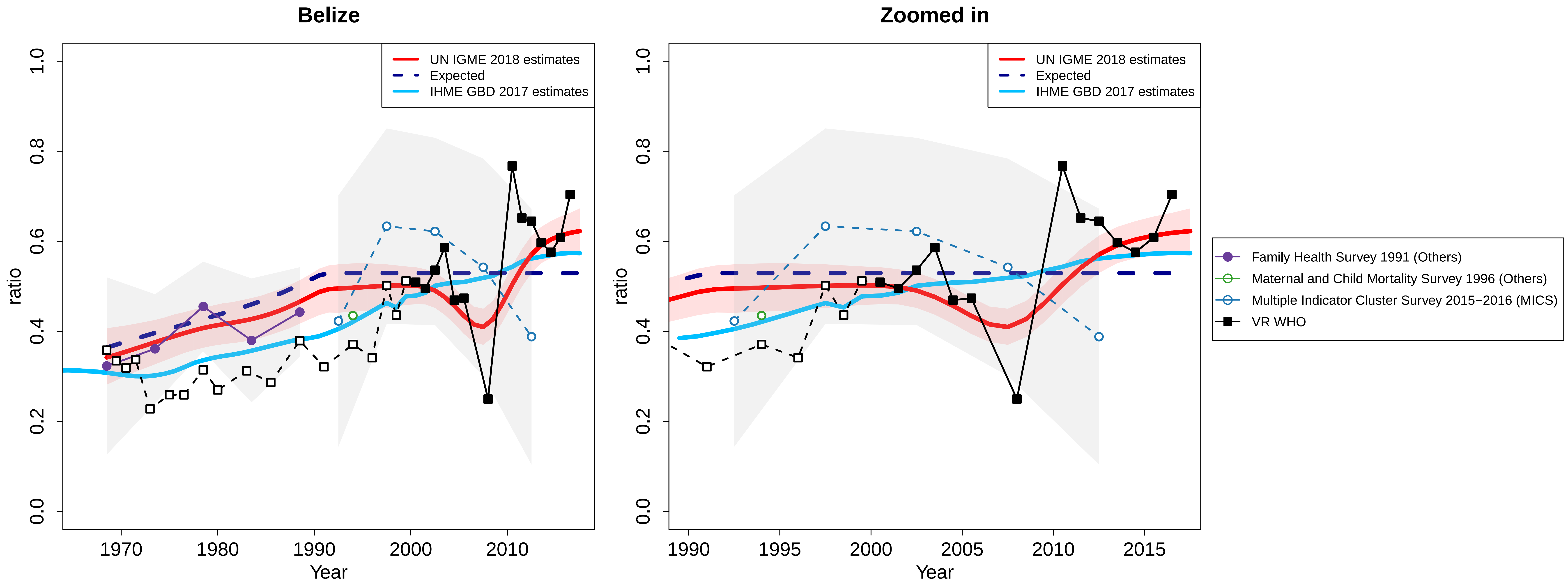
Belize
Zoomed in
UN IGME 2018 estimates
Expected
IHME GBD 2017 estimates
ratio
Year
1970
1980
1990
2000
2010
1995
2005
2015
0.0
0.2
0.4
0.6
0.8
1.0
Family Health Survey 1991 (Others)
Maternal and Child Mortality Survey 1996 (Others)
Multiple Indicator Cluster Survey 2015−2016 (MICS)
VR WHO

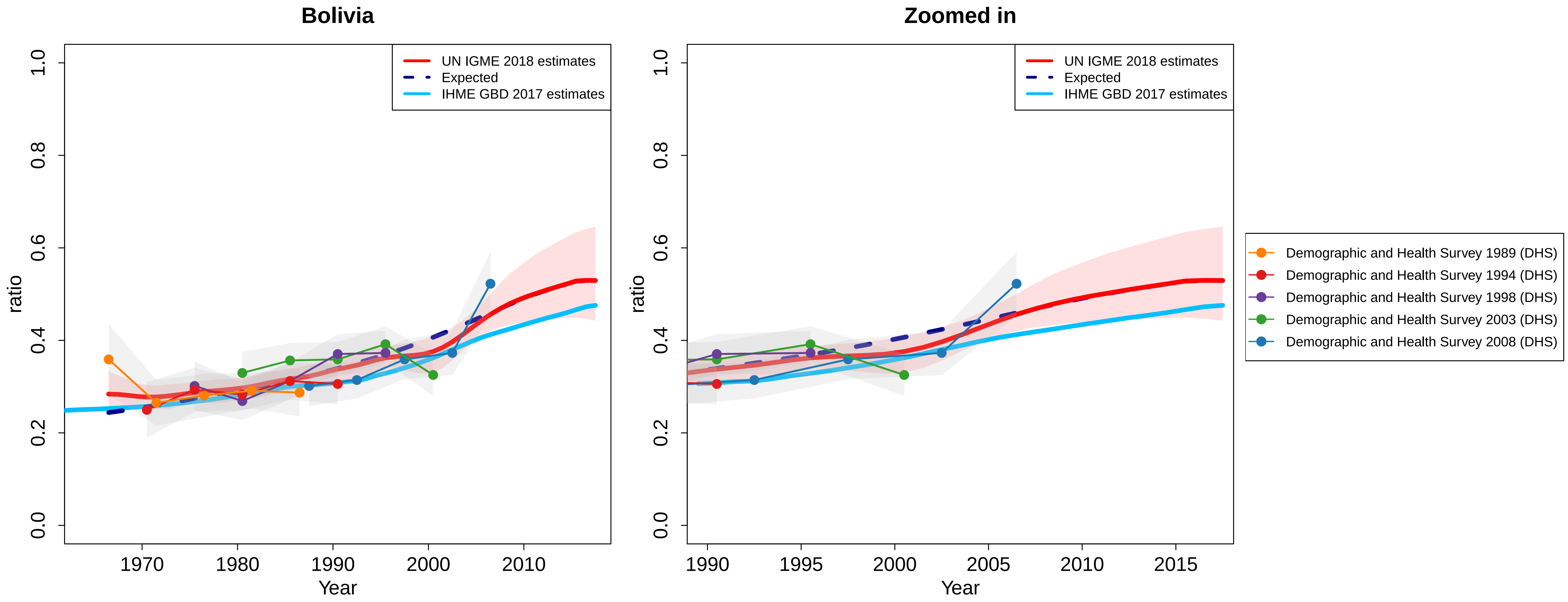

Bolivia
Zoomed in
UN IGME 2018 estimates
Expected
IHME GBD 2017 estimates
ratio
Year
1970
1980
1990
2000
2010
1995
2005
2015
0.0
0.2
0.4
0.6
0.8
1.0
Demographic and Health Survey 1989 (DHS)
Demographic and Health Survey 1994 (DHS)
Demographic and Health Survey 1998 (DHS)
Demographic and Health Survey 2003 (DHS)
Demographic and Health Survey 2008 (DHS)

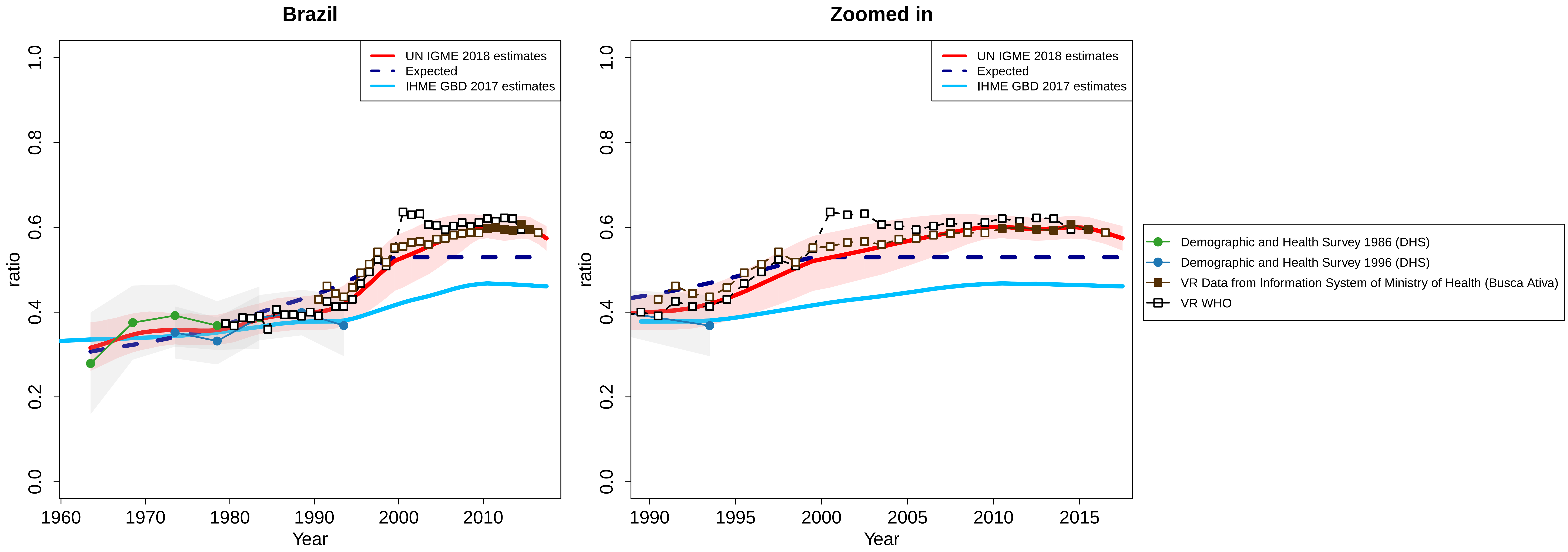

Brazil
Zoomed in
UN IGME 2018 estimates
Expected
IHME GBD 2017 estimates
ratio
Year
1960
1970
1980
1990
2000
2010
1995
2005
2015
0.0
0.2
0.4
0.6
0.8
1.0
Demographic and Health Survey 1986 (DHS)
Demographic and Health Survey 1996 (DHS)
VR Data from Information System of Ministry of Health (Busca Ativa)
VR WHO

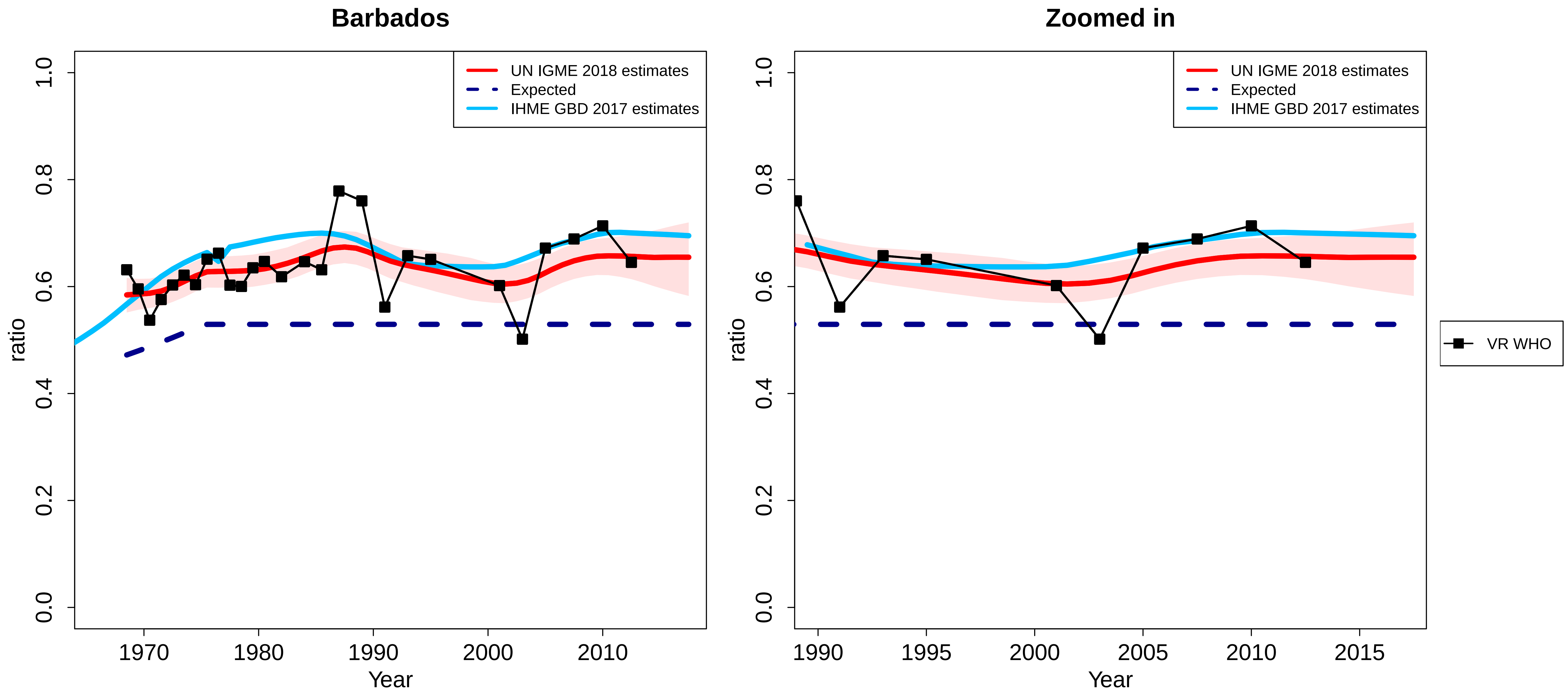
Barbados
Zoomed in
UN IGME 2018 estimates
Expected
IHME GBD 2017 estimates
VR WHO
ratio
Year

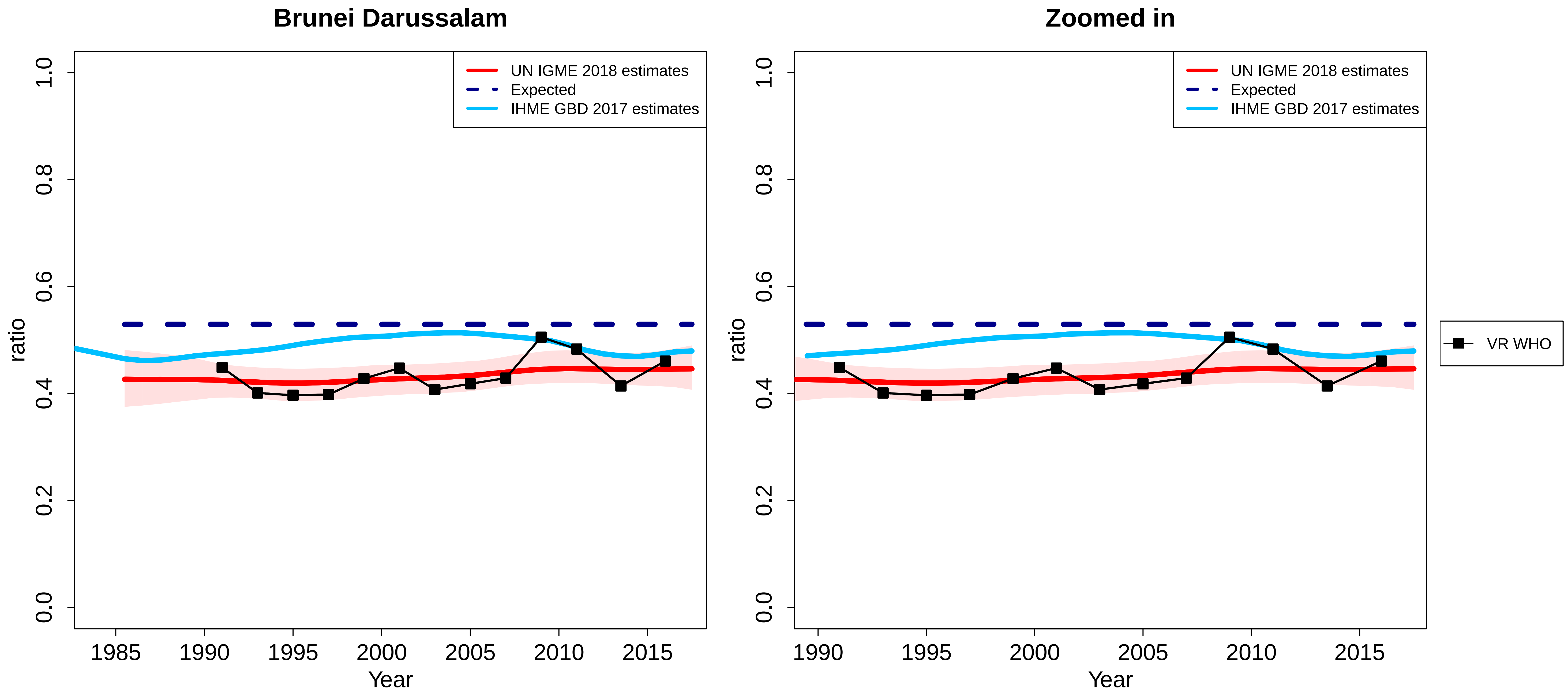

Brunei Darussalam
Zoomed in
UN IGME 2018 estimates
Expected
IHME GBD 2017 estimates
VR WHO
ratio
Year
0.0
0.2
0.4
0.6
0.8
1.0
1985
1990
1995
2000
2005
2010
2015

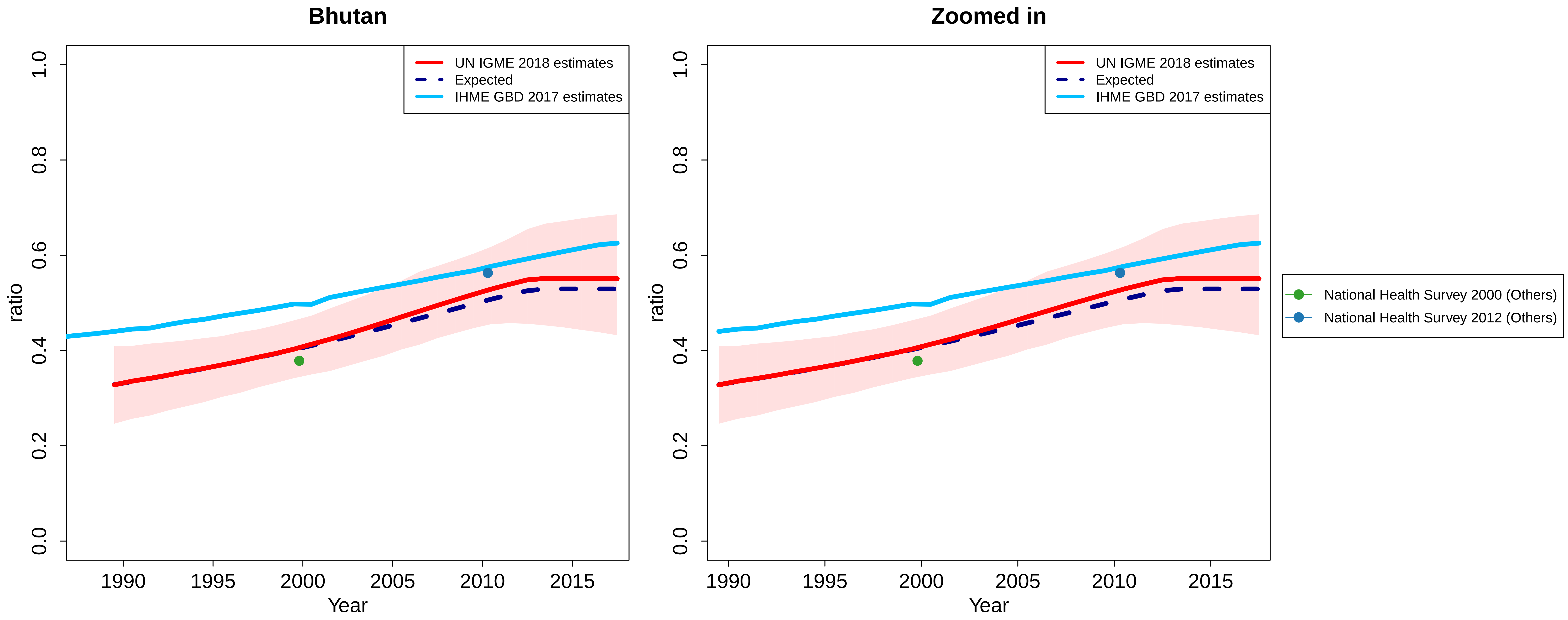

Bhutan
Zoomed in
ratio
Year
0.0
0.2
0.4
0.6
0.8
1.0
1990
1995
2000
2005
2010
2015
UN IGME 2018 estimates
Expected
IHME GBD 2017 estimates
National Health Survey 2000 (Others)
National Health Survey 2012 (Others)

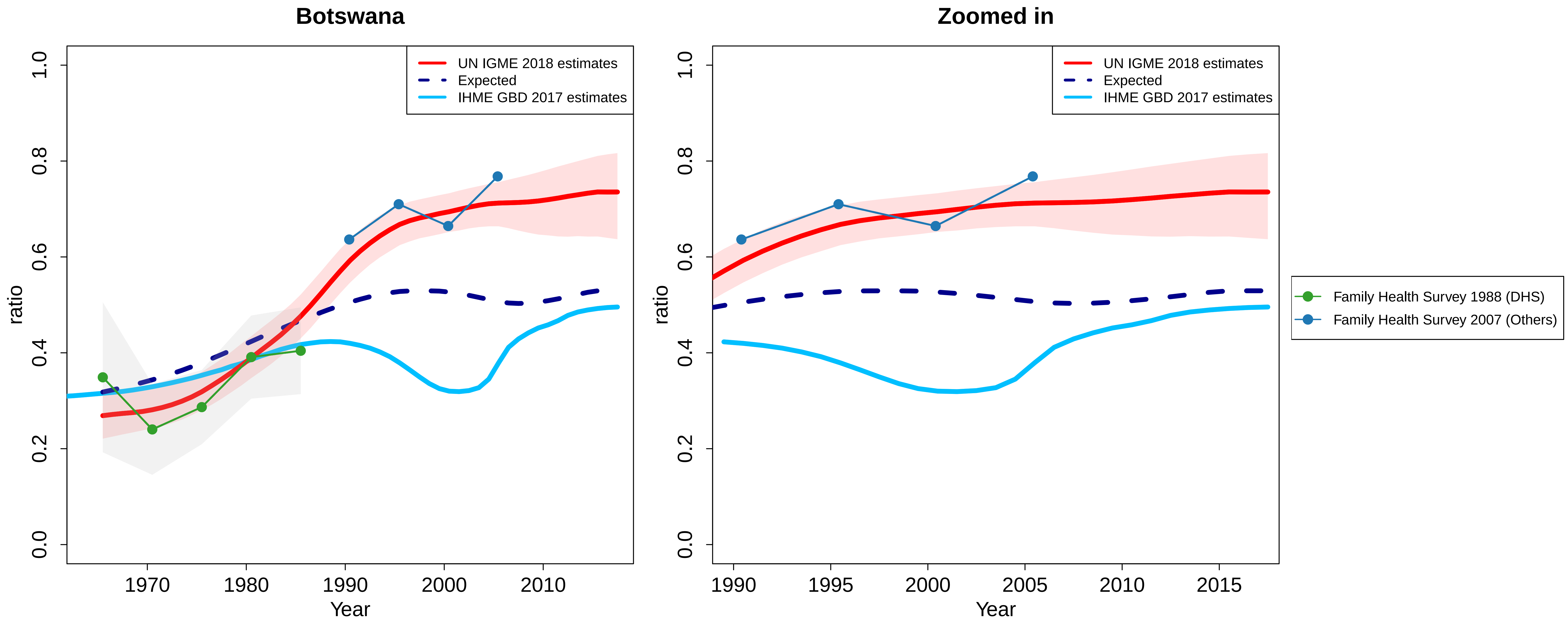
Botswana
Zoomed in
UN IGME 2018 estimates
Expected
IHME GBD 2017 estimates
Family Health Survey 1988 (DHS)
Family Health Survey 2007 (Others)
ratio
Year
0.0
0.2
0.4
0.6
0.8
1.0
1970
1980
1990
2000
2010
1995
2005
2015

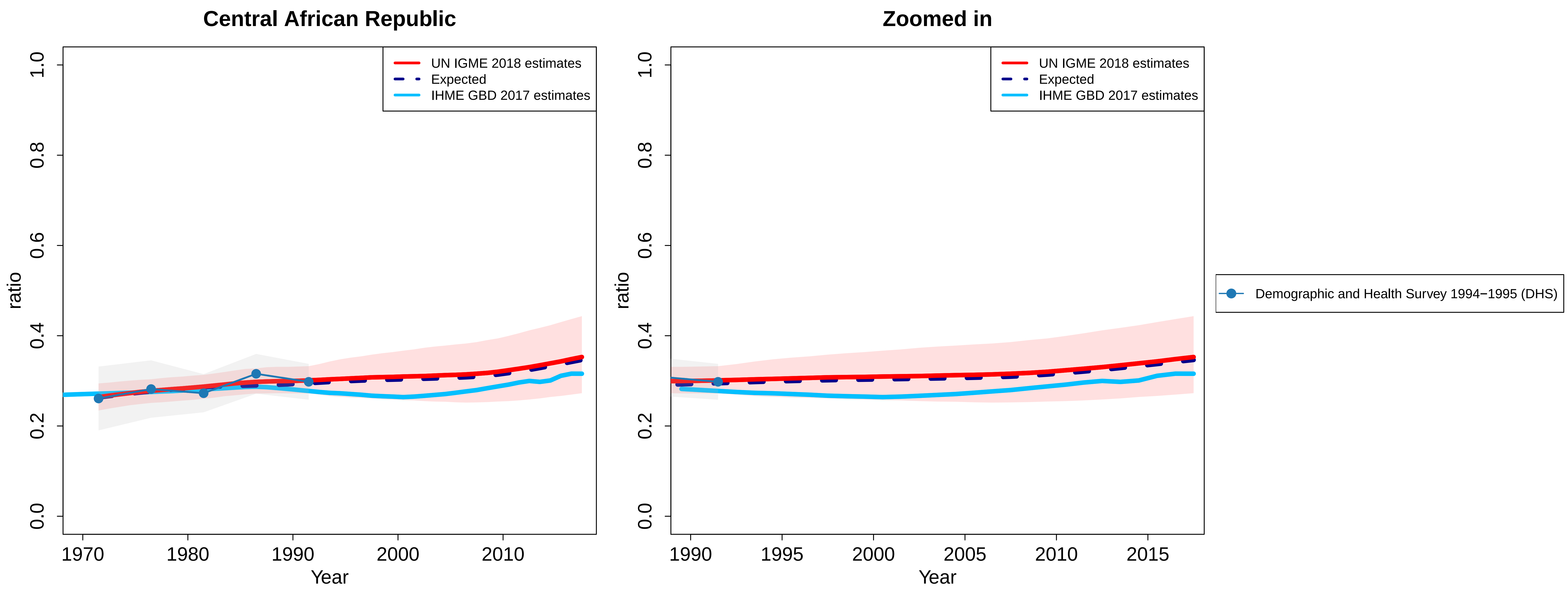

Central African Republic
Zoomed in
UN IGME 2018 estimates
Expected
IHME GBD 2017 estimates
Demographic and Health Survey 1994−1995 (DHS)
ratio
Year
0.0
0.2
0.4
0.6
0.8
1.0
1970
1980
1990
2000
2010
1995
2005
2015

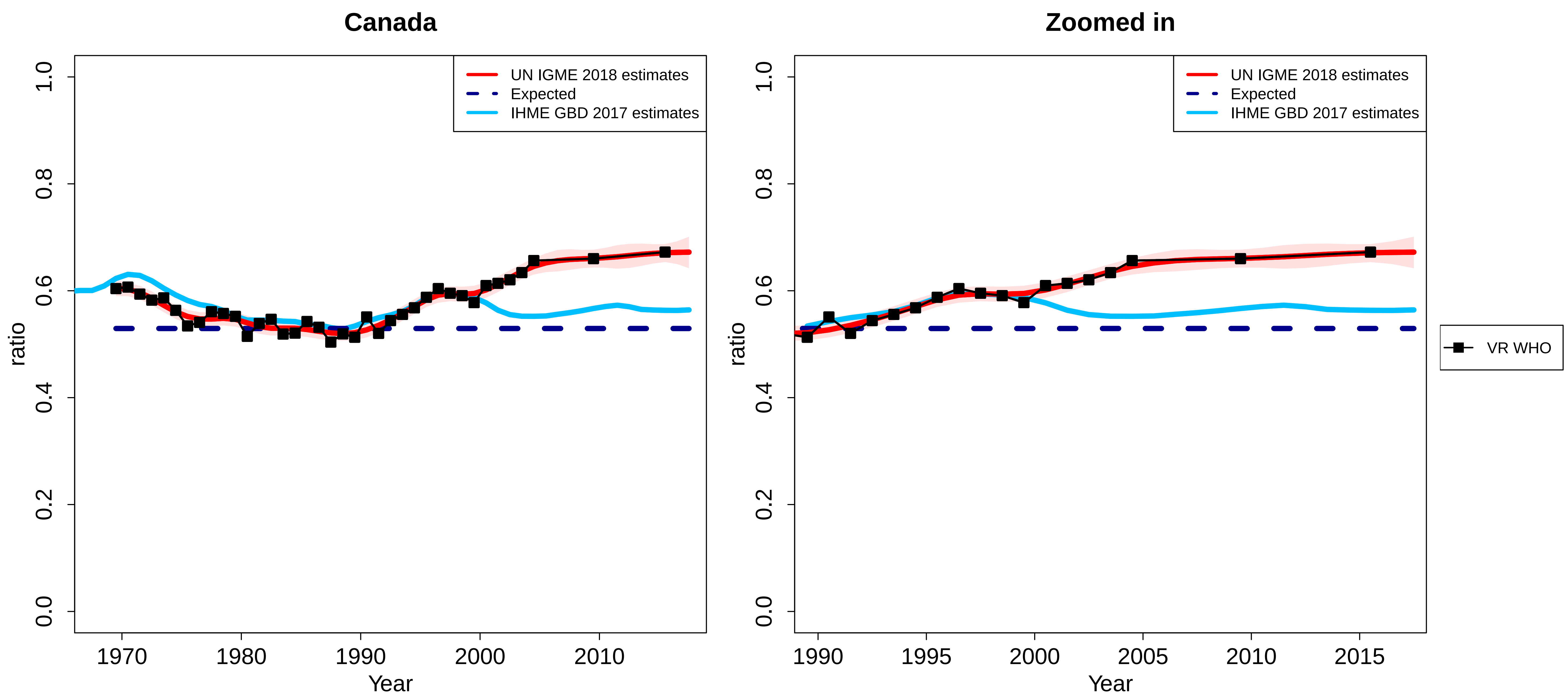
Canada
Zoomed in
UN IGME 2018 estimates
Expected
IHME GBD 2017 estimates
VR WHO
ratio
Year
1.0
0.8
0.6
0.4
0.2
0.0
1970
1980
1990
2000
2010
1995
2005
2015

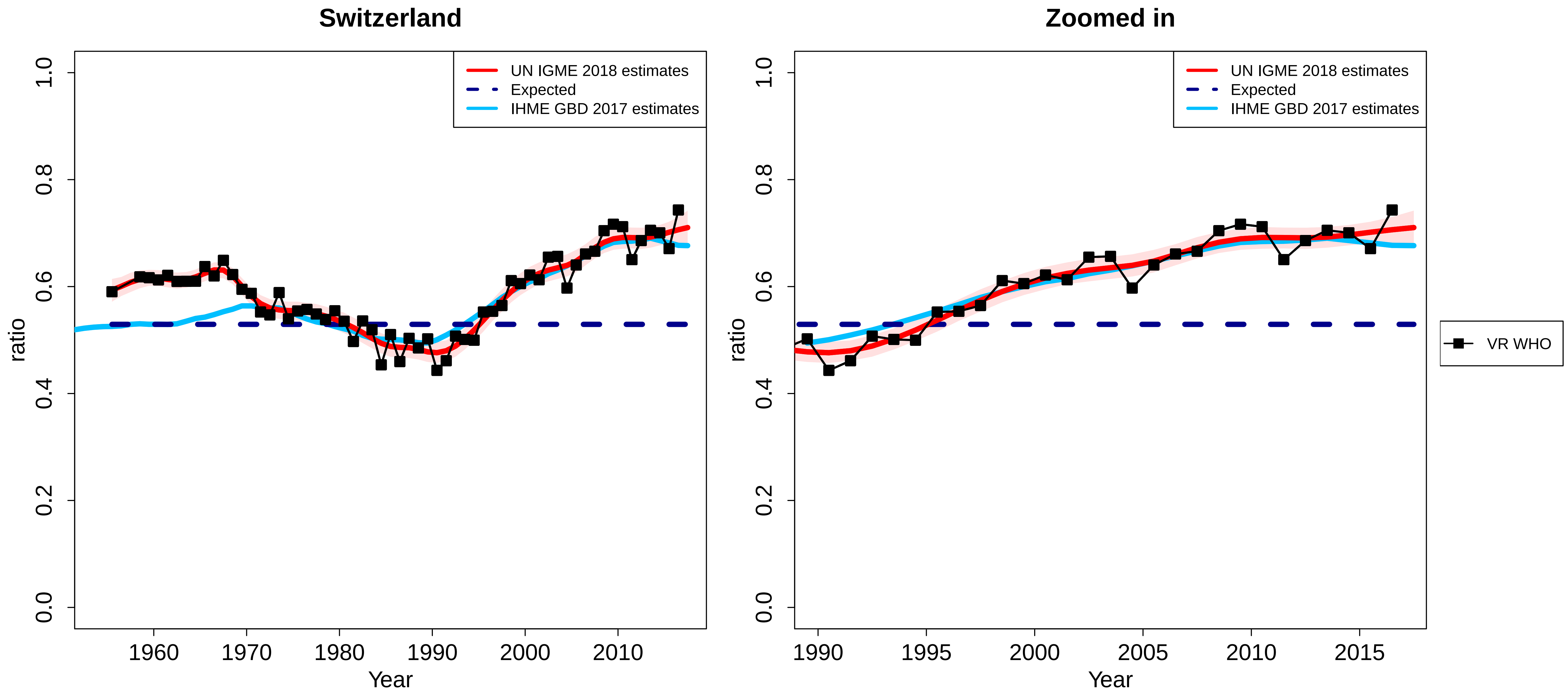
Switzerland
Zoomed in
UN IGME 2018 estimates
Expected
IHME GBD 2017 estimates
VR WHO
ratio
Year
0.0
0.2
0.4
0.6
0.8
1.0
1960
1970
1980
1990
2000
2010
1995
2005
2015

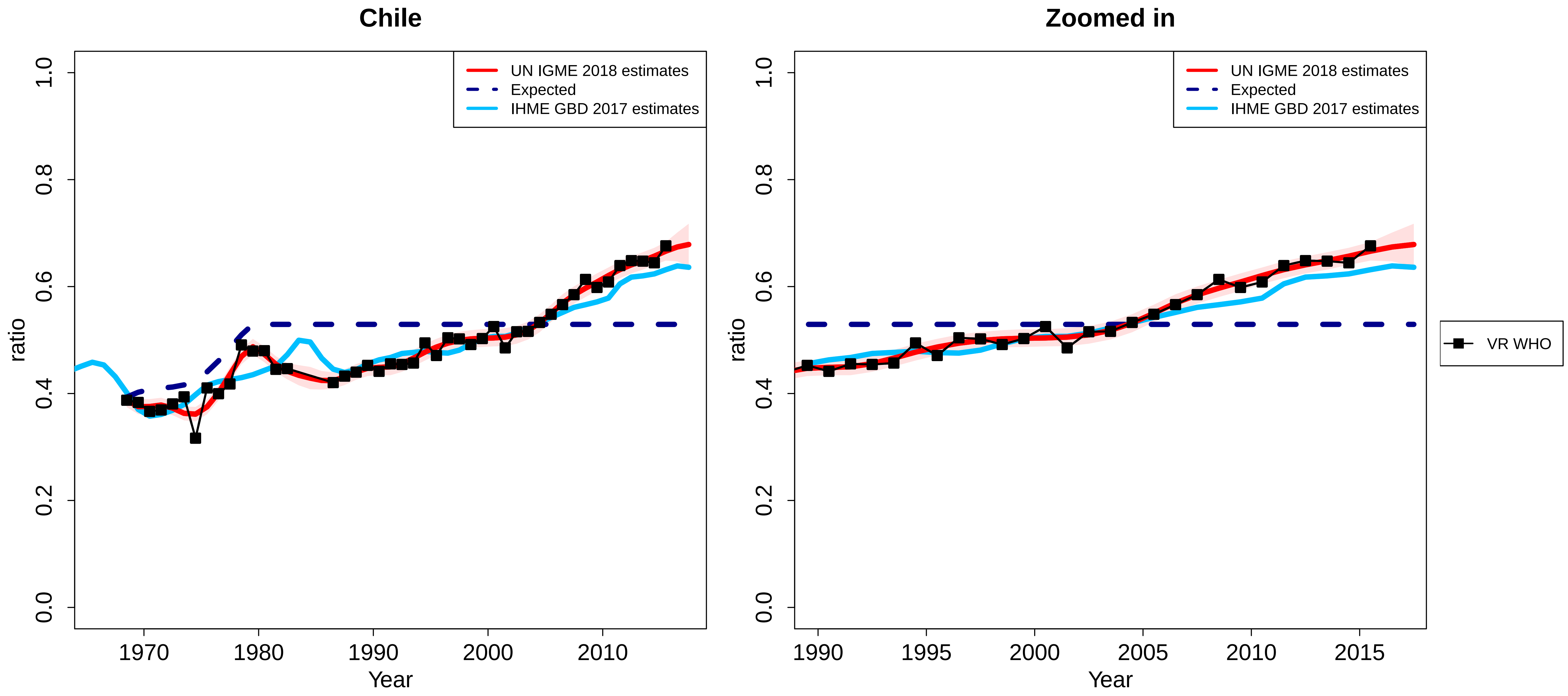
Chile
Zoomed in
UN IGME 2018 estimates
Expected
IHME GBD 2017 estimates
VR WHO
ratio
Year
0.0
0.2
0.4
0.6
0.8
1.0
1970
1980
1990
2000
2010
1995
2005
2015

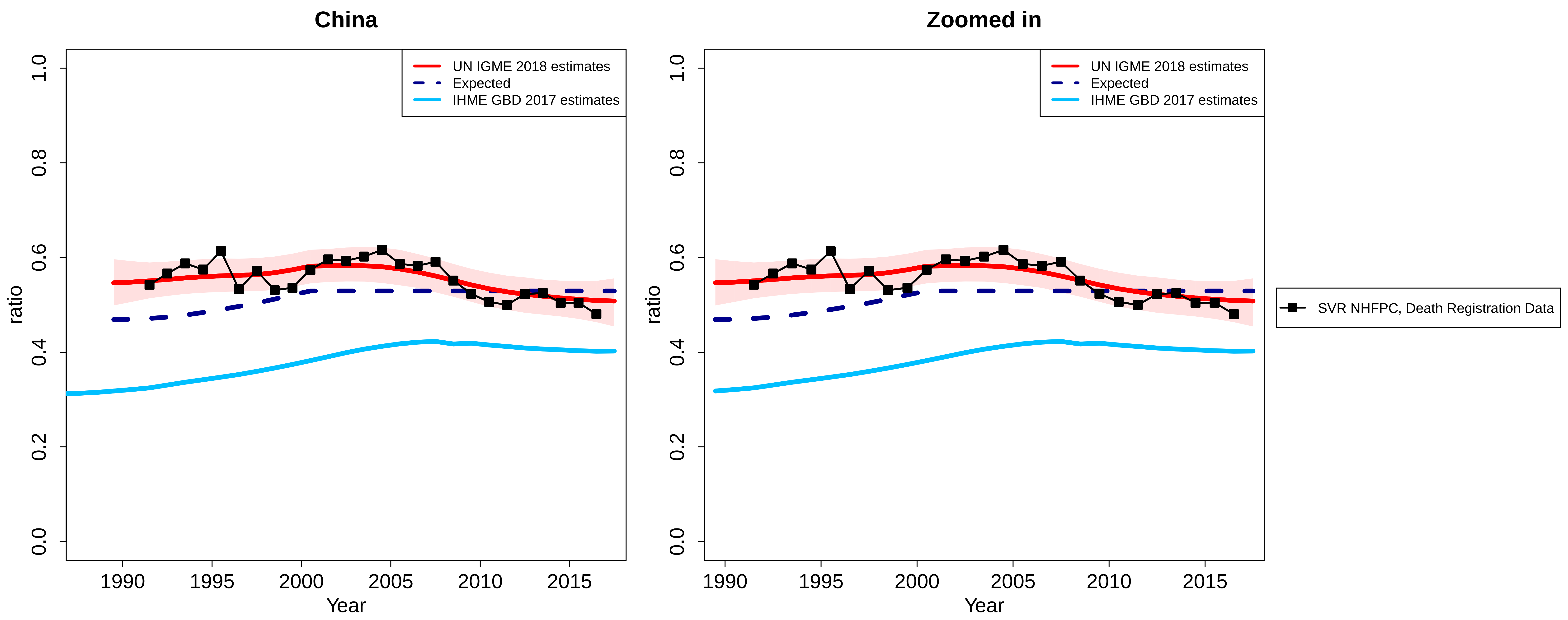

China
Zoomed in
UN IGME 2018 estimates
Expected
IHME GBD 2017 estimates
SVR NHFPC, Death Registration Data
ratio
Year
0.0
0.2
0.4
0.6
0.8
1.0
1990
1995
2000
2005
2010
2015

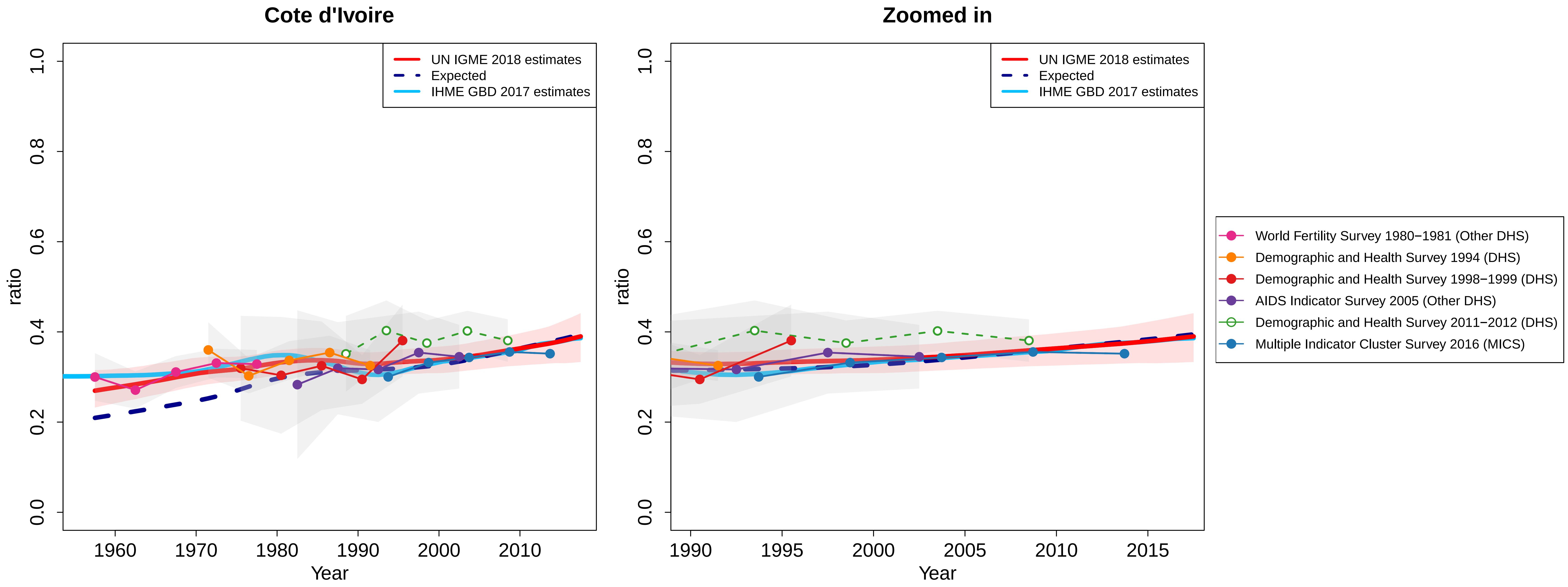
Cote d'Ivoire
Zoomed in
UN IGME 2018 estimates
Expected
IHME GBD 2017 estimates
ratio
Year
World Fertility Survey 1980−1981 (Other DHS)
Demographic and Health Survey 1994 (DHS)
Demographic and Health Survey 1998−1999 (DHS)
AIDS Indicator Survey 2005 (Other DHS)
Demographic and Health Survey 2011−2012 (DHS)
Multiple Indicator Cluster Survey 2016 (MICS)

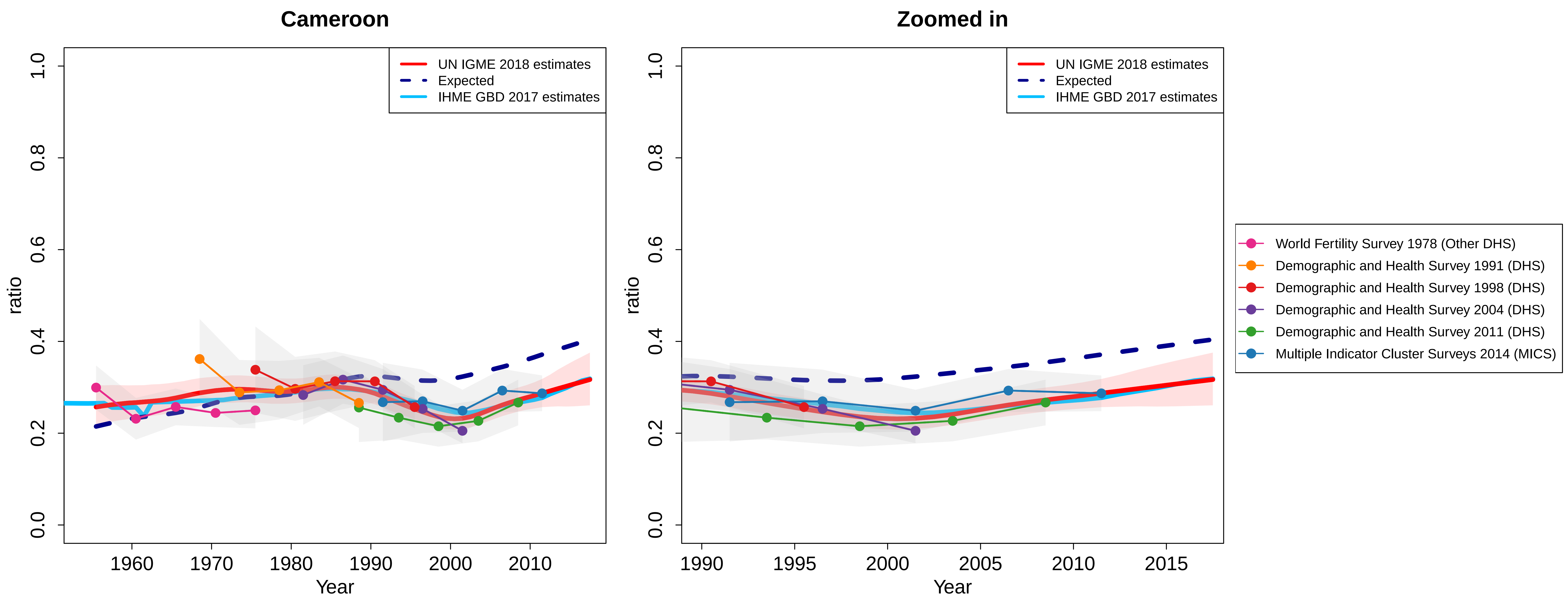

Cameroon
Zoomed in
UN IGME 2018 estimates
Expected
IHME GBD 2017 estimates
ratio
Year
1960
1970
1980
1990
2000
2010
1995
2005
2015
0.0
0.2
0.4
0.6
0.8
1.0
World Fertility Survey 1978 (Other DHS)
Demographic and Health Survey 1991 (DHS)
Demographic and Health Survey 1998 (DHS)
Demographic and Health Survey 2004 (DHS)
Demographic and Health Survey 2011 (DHS)
Multiple Indicator Cluster Surveys 2014 (MICS)

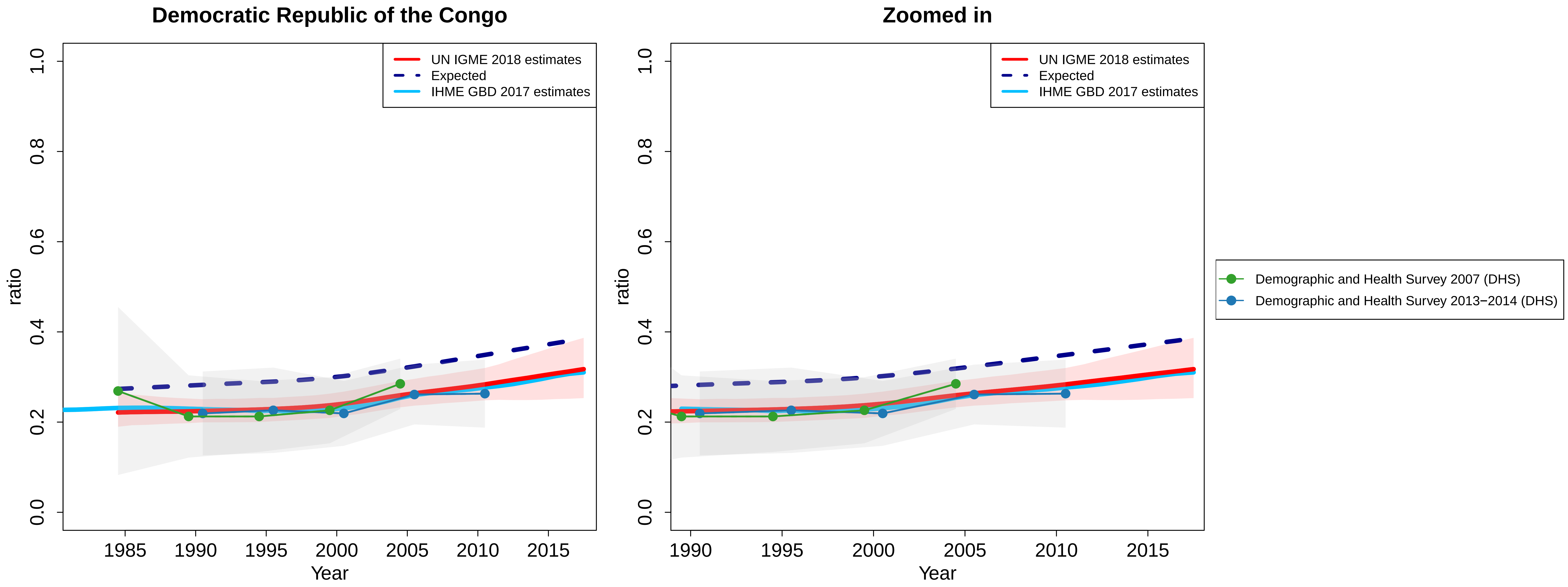

Democratic Republic of the Congo
Zoomed in
UN IGME 2018 estimates
Expected
IHME GBD 2017 estimates
Demographic and Health Survey 2007 (DHS)
Demographic and Health Survey 2013−2014 (DHS)
ratio
Year

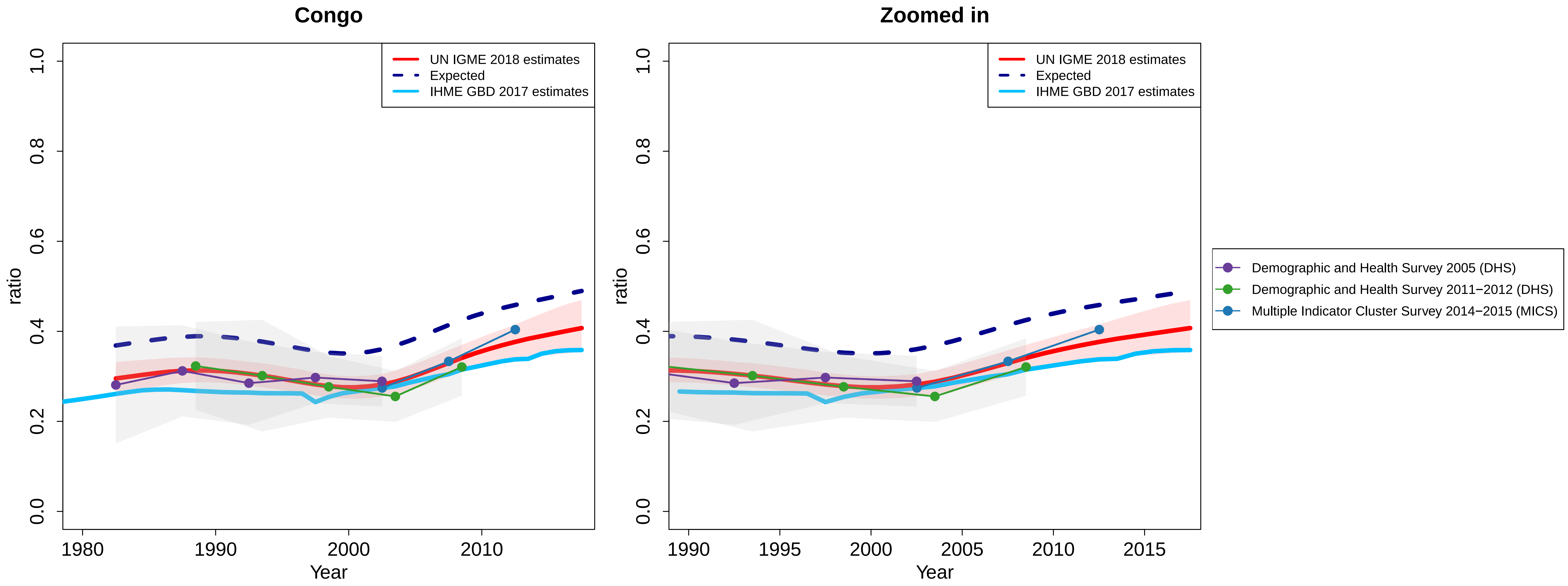

Congo
Zoomed in
UN IGME 2018 estimates
Expected
IHME GBD 2017 estimates
ratio
Year
0.0
0.2
0.4
0.6
0.8
1.0
1980
1990
2000
2010
1995
2005
2015
Demographic and Health Survey 2005 (DHS)
Demographic and Health Survey 2011−2012 (DHS)
Multiple Indicator Cluster Survey 2014−2015 (MICS)

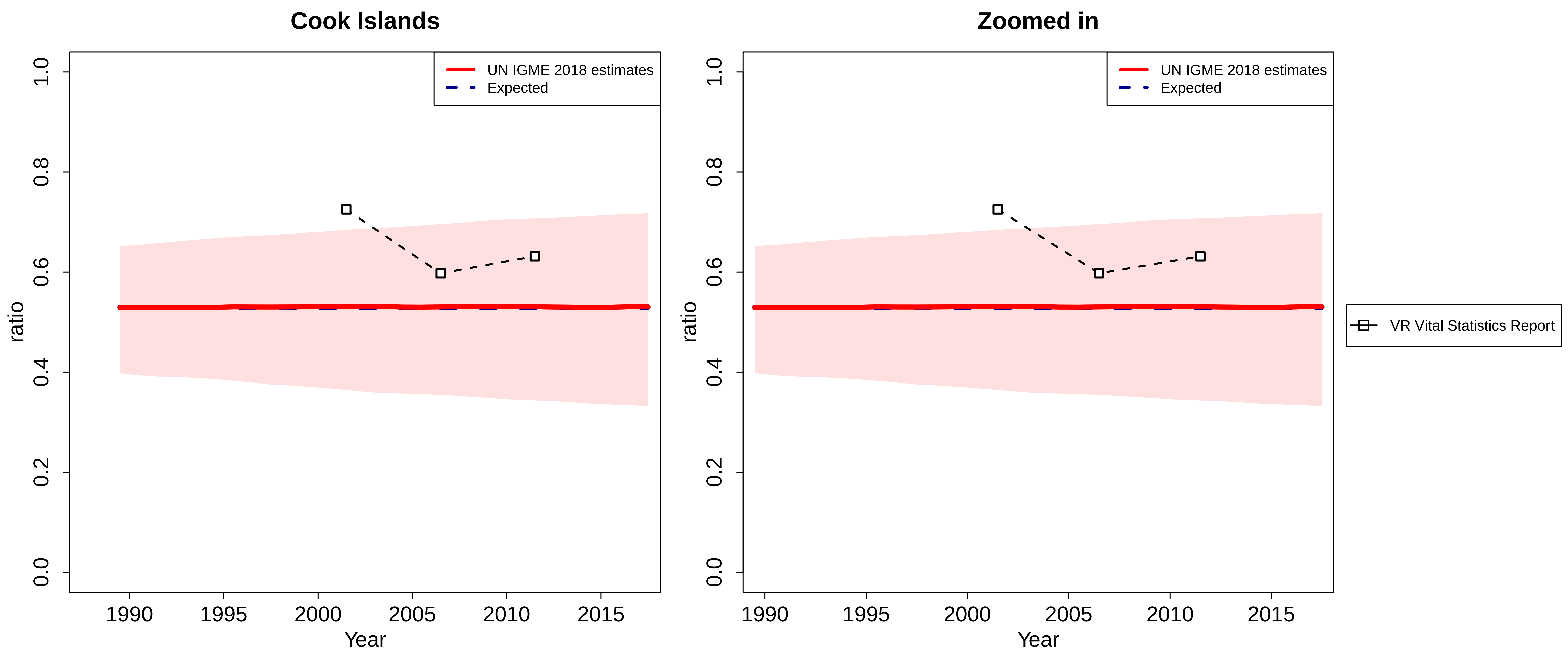

Cook Islands
Zoomed in
UN IGME 2018 estimates
Expected
VR Vital Statistics Report
ratio
Year
0.0
0.2
0.4
0.6
0.8
1.0
1990
1995
2000
2005
2010
2015

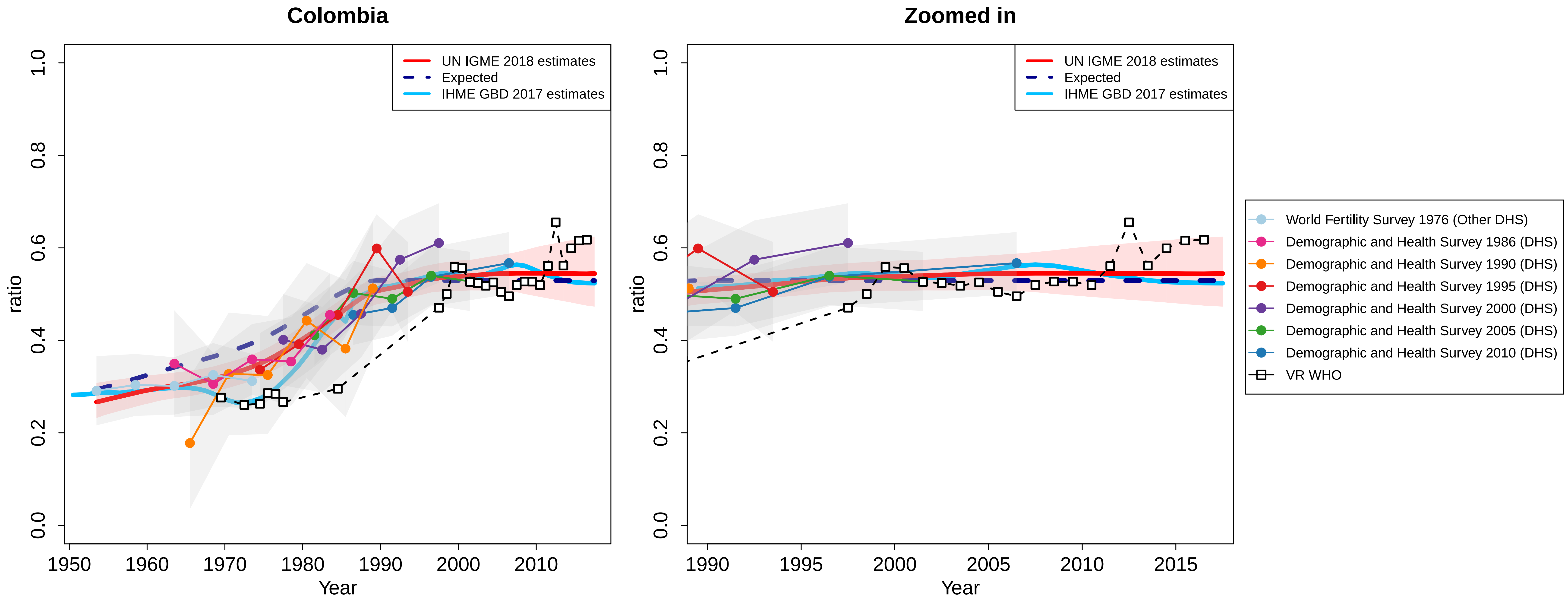

Colombia
Zoomed in
ratio
Year
1.0
0.8
0.6
0.4
0.2
0.0
1950
1960
1970
1980
1990
2000
2010
1995
2005
2015
UN IGME 2018 estimates
Expected
IHME GBD 2017 estimates
World Fertility Survey 1976 (Other DHS)
Demographic and Health Survey 1986 (DHS)
Demographic and Health Survey 1990 (DHS)
Demographic and Health Survey 1995 (DHS)
Demographic and Health Survey 2000 (DHS)
Demographic and Health Survey 2005 (DHS)
Demographic and Health Survey 2010 (DHS)
VR WHO

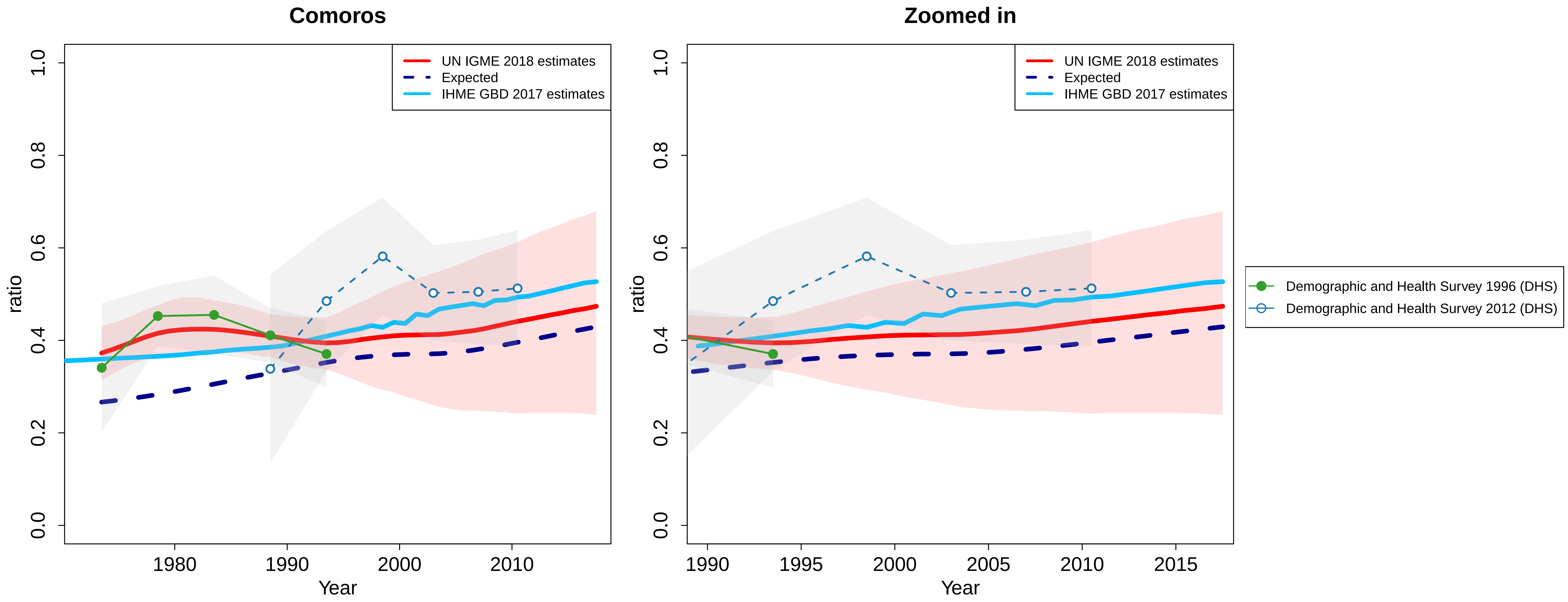
Comoros
Zoomed in
UN IGME 2018 estimates
Expected
IHME GBD 2017 estimates
ratio
Year
0.0
0.2
0.4
0.6
0.8
1.0
1980
1990
2000
2010
1995
2005
2015
Demographic and Health Survey 1996 (DHS)
Demographic and Health Survey 2012 (DHS)

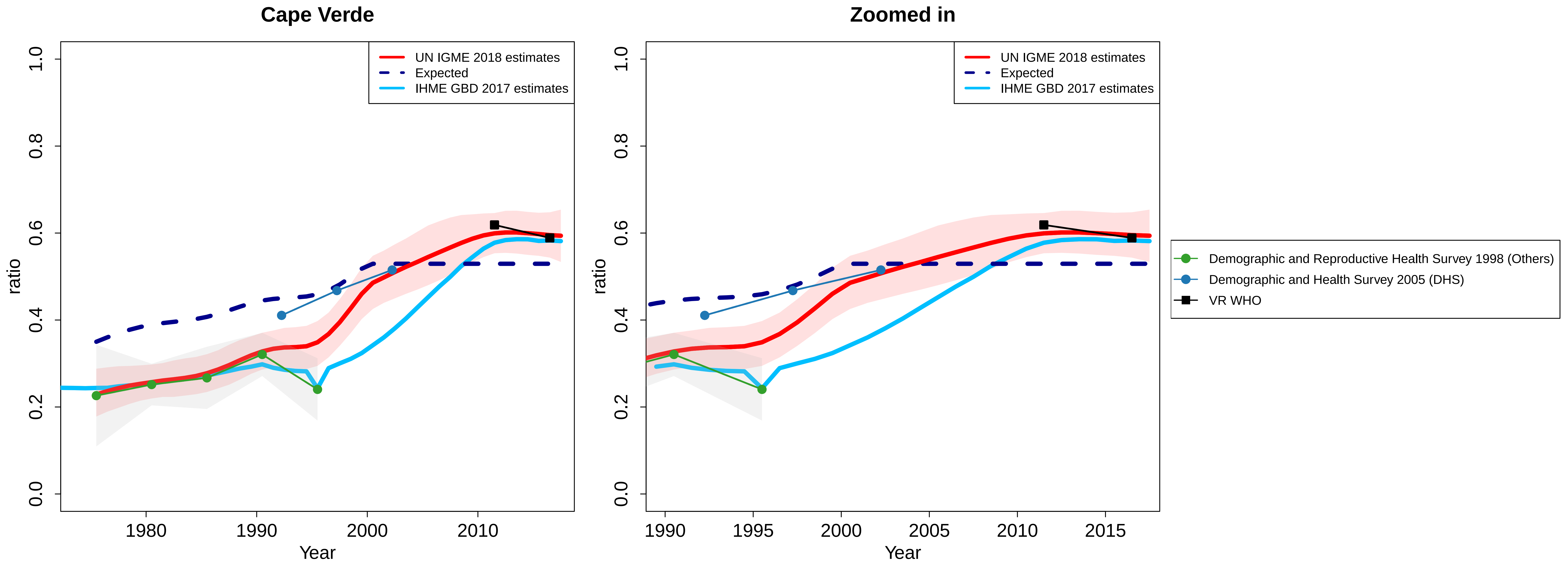
Cape Verde
Zoomed in
UN IGME 2018 estimates
Expected
IHME GBD 2017 estimates
ratio
Year
0.0
0.2
0.4
0.6
0.8
1.0
1980
1990
2000
2010
1995
2005
2015
Demographic and Reproductive Health Survey 1998 (Others)
Demographic and Health Survey 2005 (DHS)
VR WHO

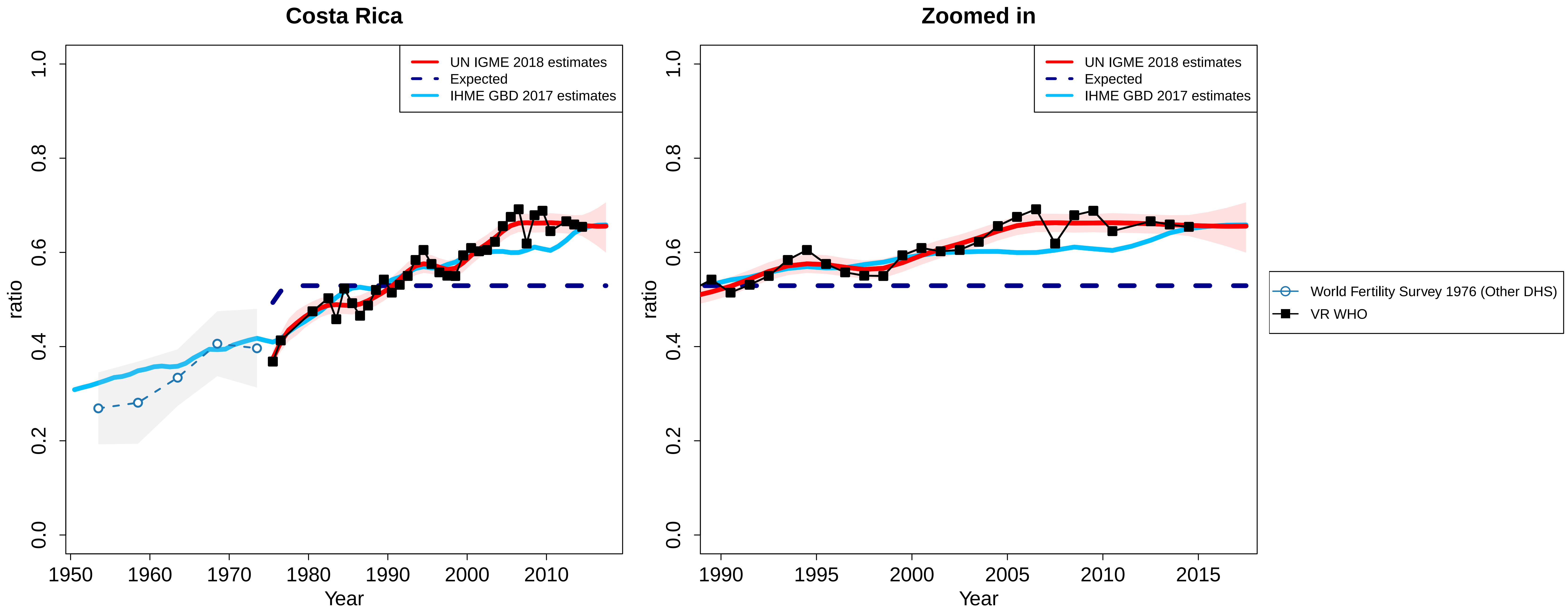

Costa Rica
Zoomed in
UN IGME 2018 estimates
Expected
IHME GBD 2017 estimates
ratio
Year
0.0
0.2
0.4
0.6
0.8
1.0
1950
1960
1970
1980
1990
2000
2010
1995
2005
2015
World Fertility Survey 1976 (Other DHS)
VR WHO

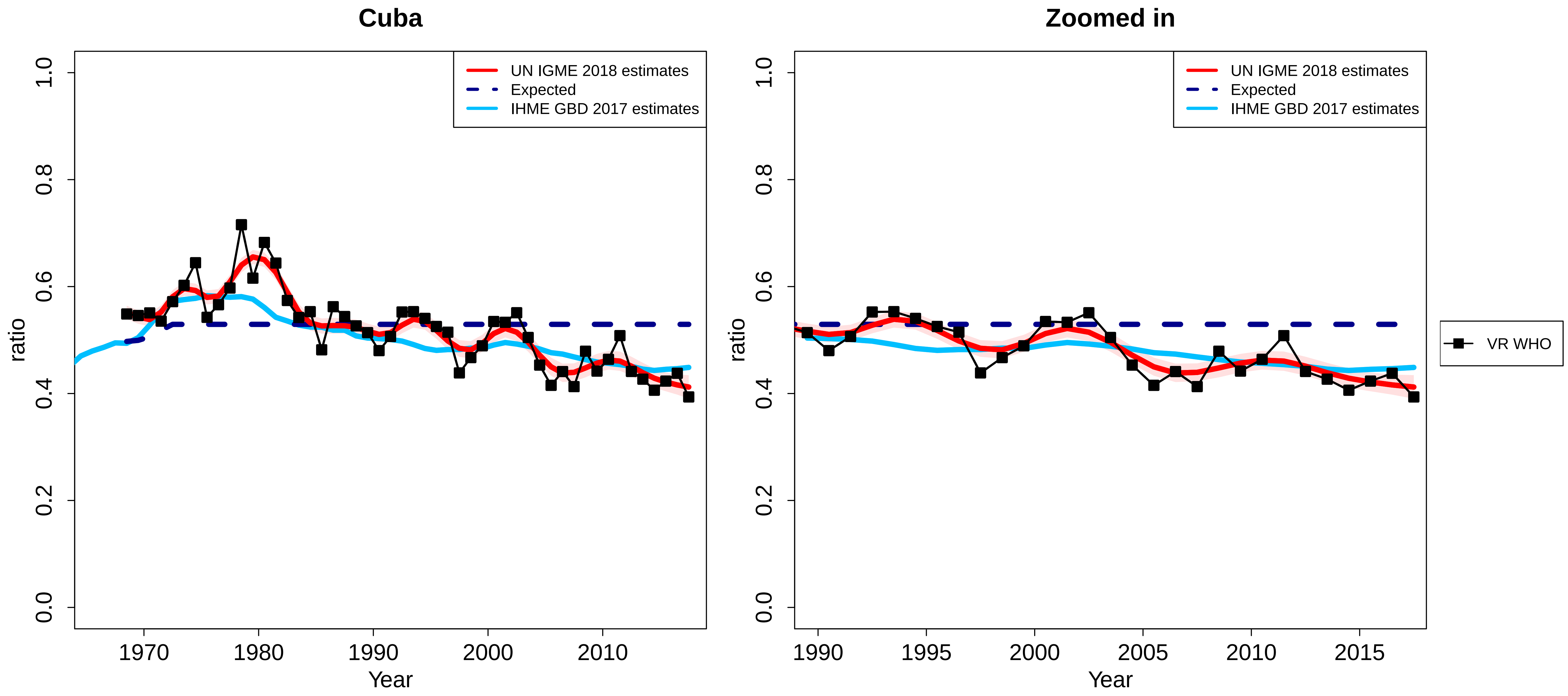
Cuba
Zoomed in
UN IGME 2018 estimates
Expected
IHME GBD 2017 estimates
VR WHO
ratio
Year
0.0
0.2
0.4
0.6
0.8
1.0
1970
1980
1990
2000
2010
1995
2005
2015

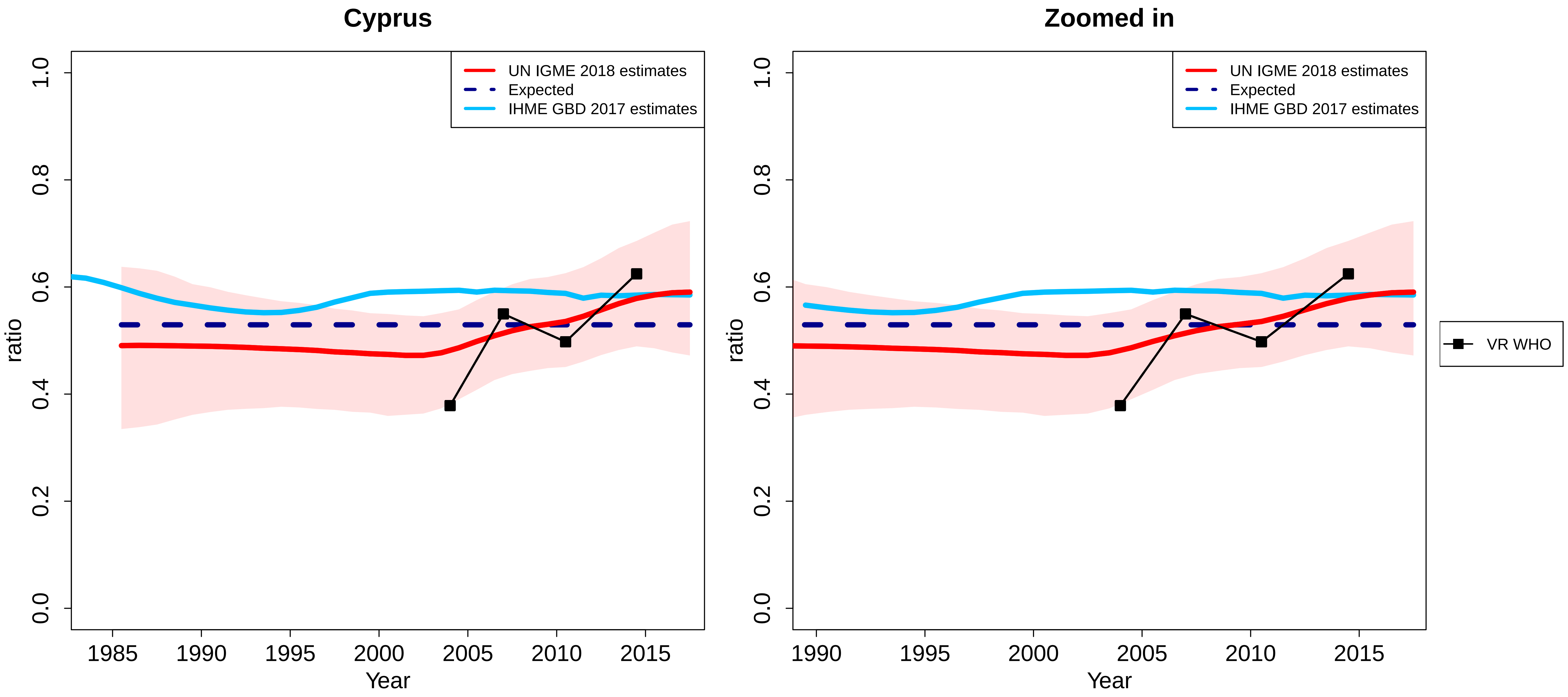
Cyprus
Zoomed in
UN IGME 2018 estimates
Expected
IHME GBD 2017 estimates
ratio
Year
0.0
0.2
0.4
0.6
0.8
1.0
1985
1990
1995
2000
2005
2010
2015
VR WHO

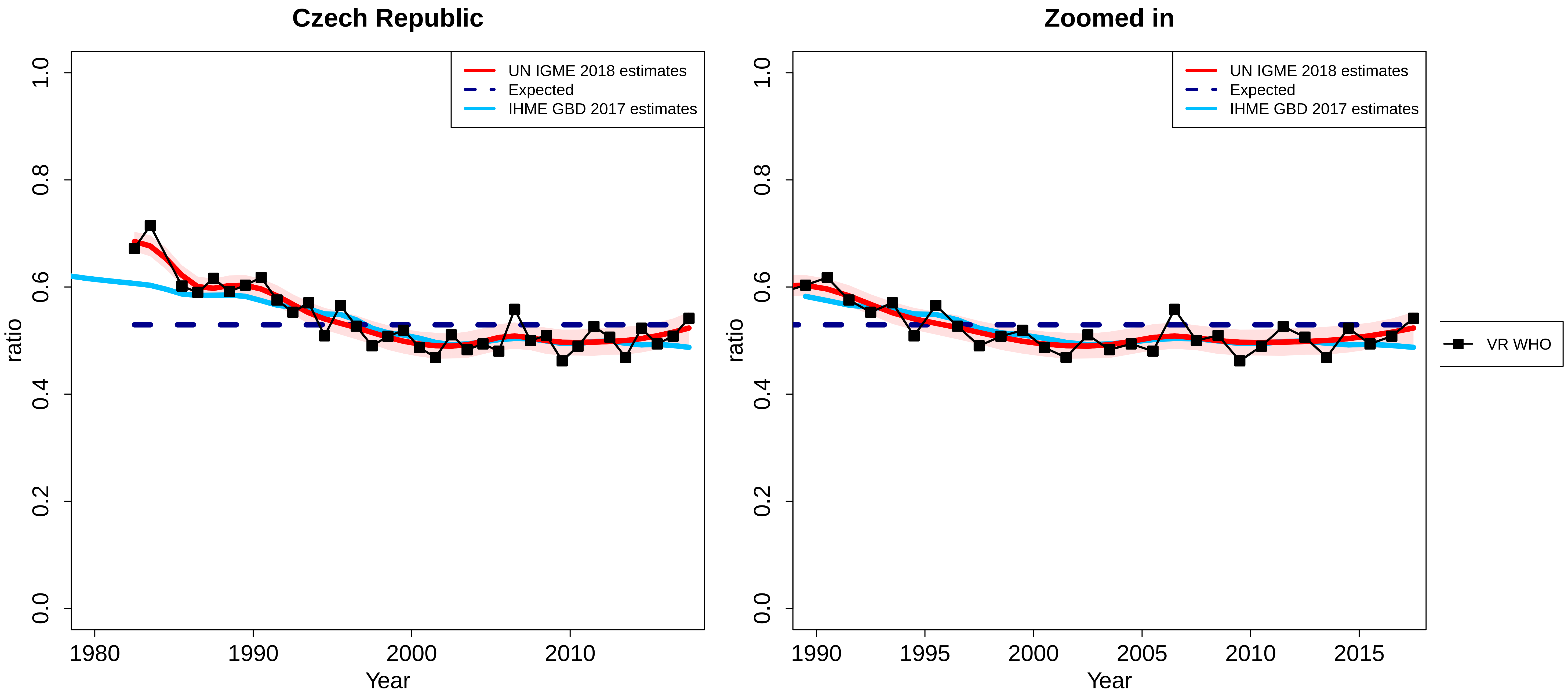
Czech Republic
Zoomed in
UN IGME 2018 estimates
Expected
IHME GBD 2017 estimates
VR WHO
ratio
Year
0.0
0.2
0.4
0.6
0.8
1.0
1980
1990
2000
2010
1995
2005
2015

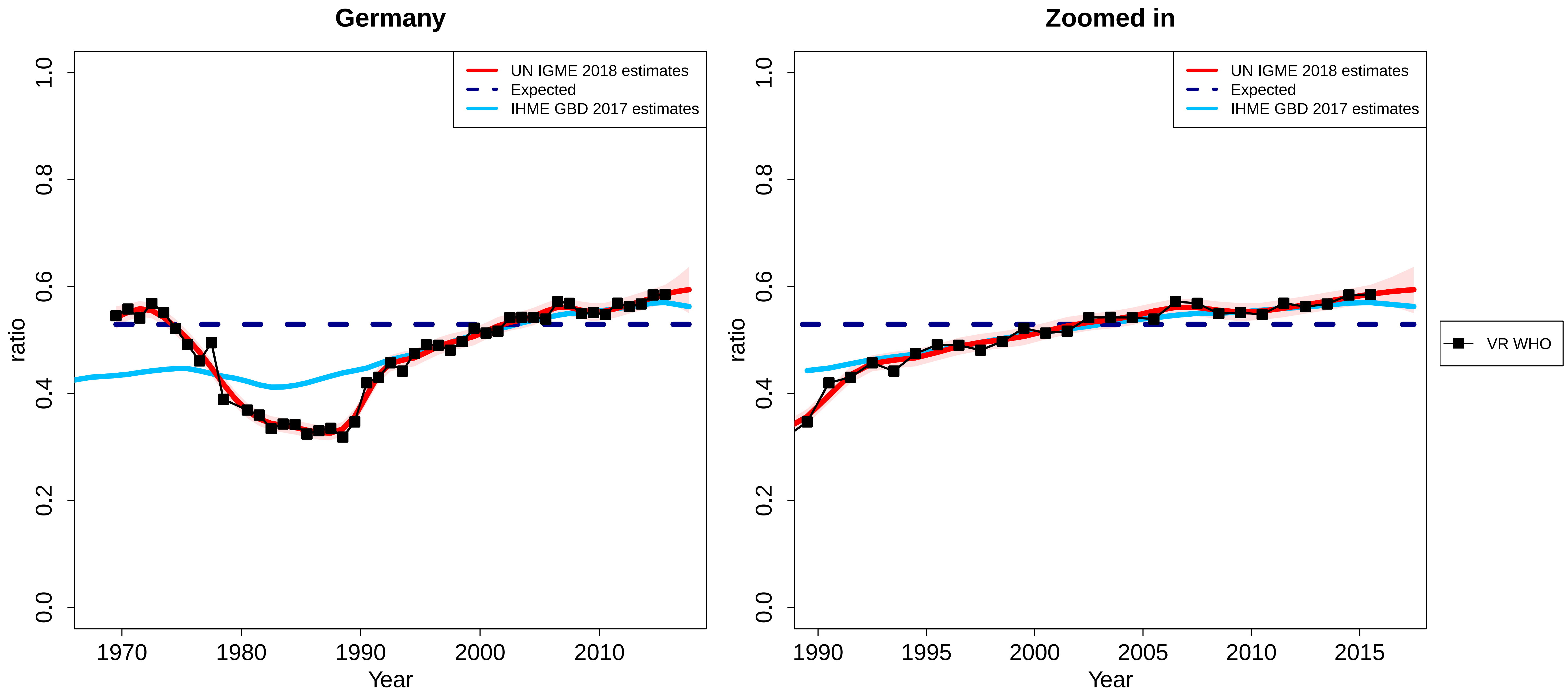
Germany
Zoomed in
UN IGME 2018 estimates
Expected
IHME GBD 2017 estimates
VR WHO
ratio
Year
0.0
0.2
0.4
0.6
0.8
1.0
1970
1980
1990
2000
2010
1990
1995
2000
2005
2010
2015

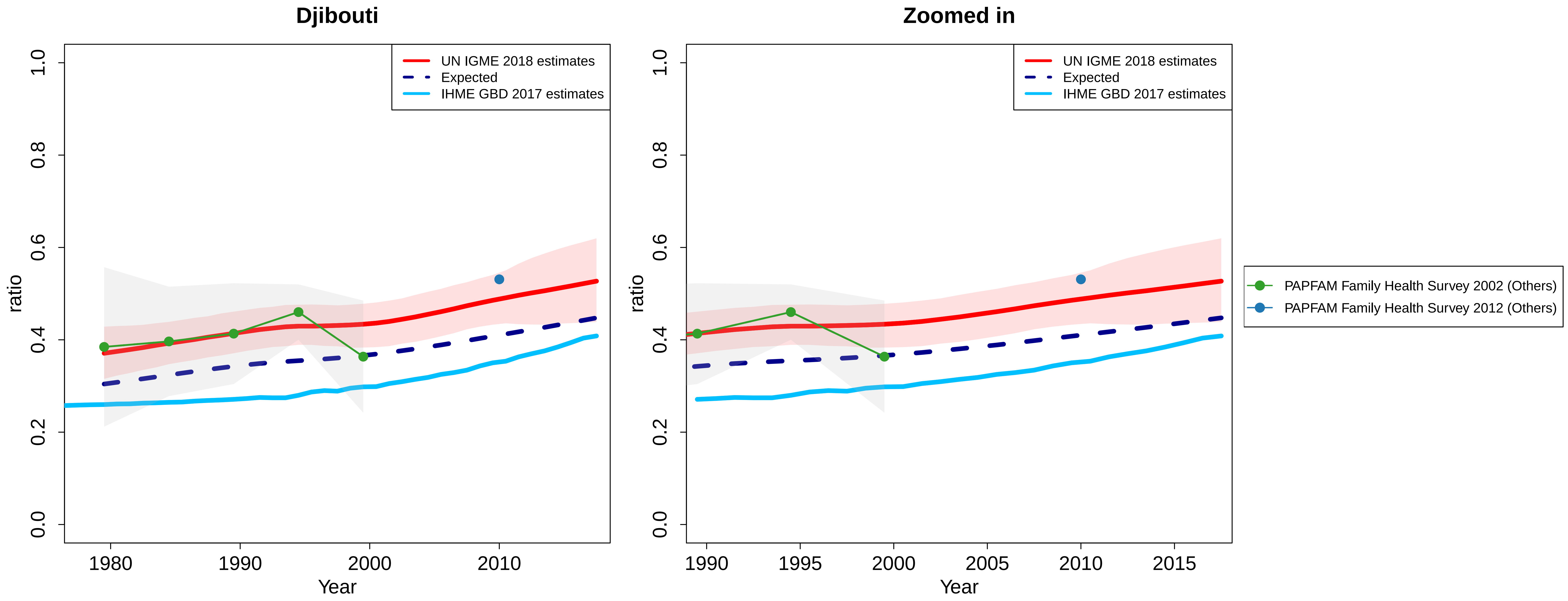
Djibouti
Zoomed in
UN IGME 2018 estimates
Expected
IHME GBD 2017 estimates
PAPFAM Family Health Survey 2002 (Others)
PAPFAM Family Health Survey 2012 (Others)
ratio
Year
0.0
0.2
0.4
0.6
0.8
1.0
1980
1990
2000
2010
1995
2005
2015

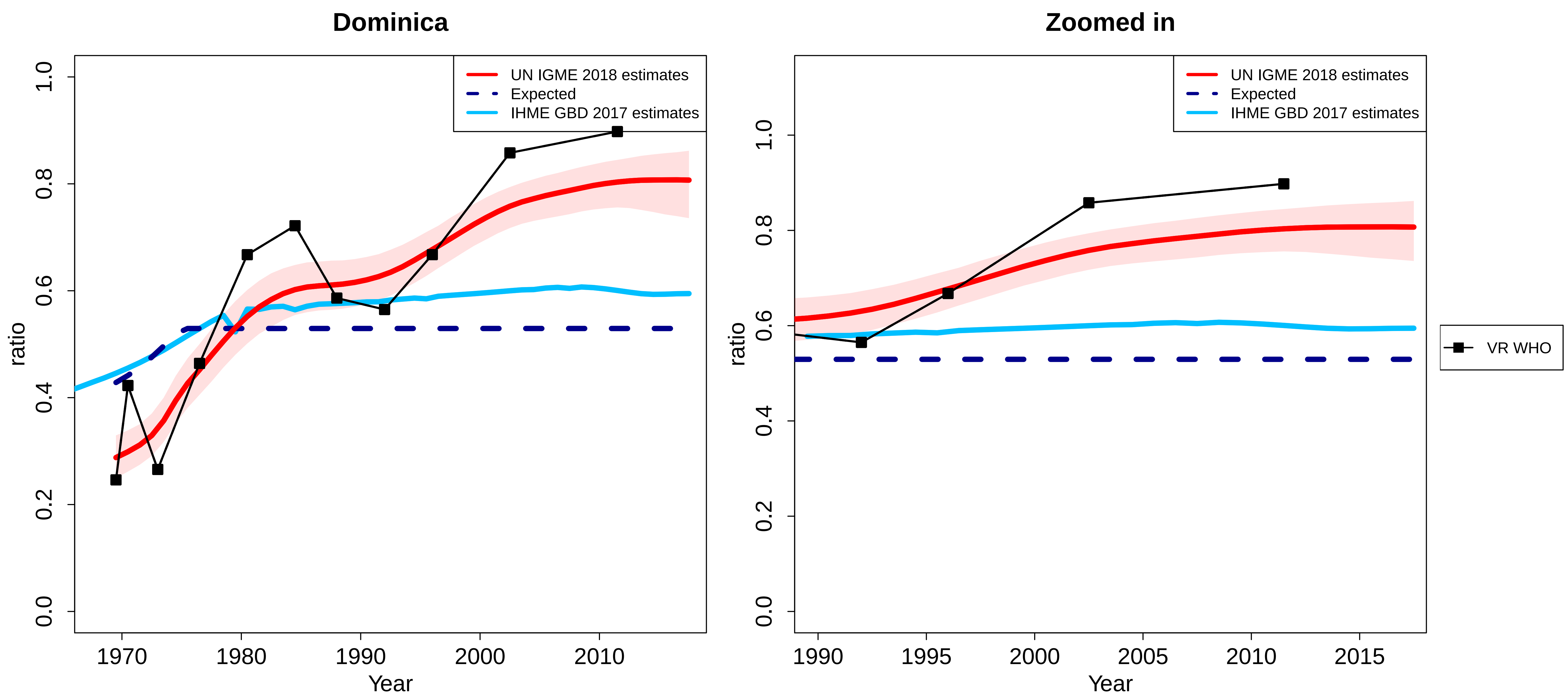

Dominica
Zoomed in
UN IGME 2018 estimates
Expected
IHME GBD 2017 estimates
VR WHO
ratio
Year
0.0
0.2
0.4
0.6
0.8
1.0
1970
1980
1990
2000
2010
1995
2005
2015

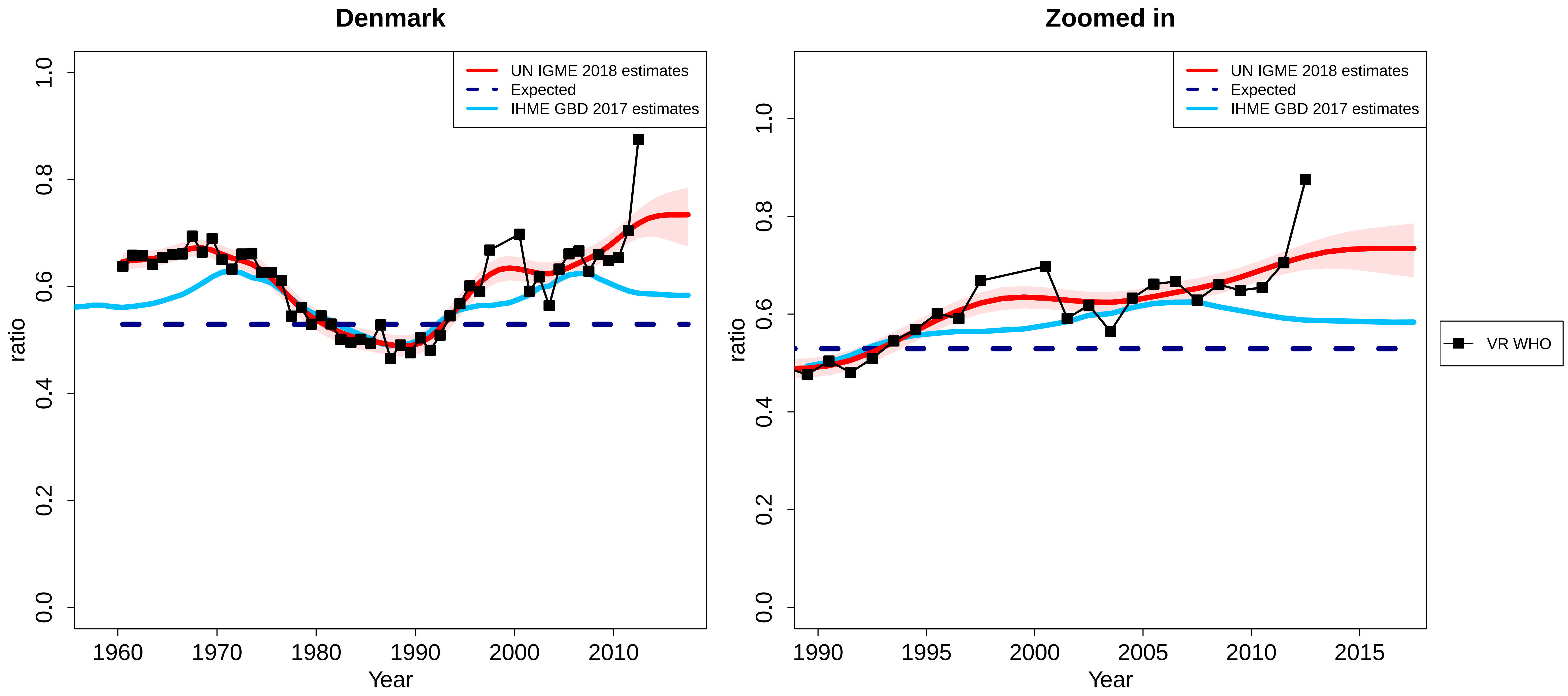

Denmark
Zoomed in
UN IGME 2018 estimates
Expected
IHME GBD 2017 estimates
VR WHO
ratio
Year
0.0
0.2
0.4
0.6
0.8
1.0
1960
1970
1980
1990
2000
2010
1990
1995
2000
2005
2010
2015

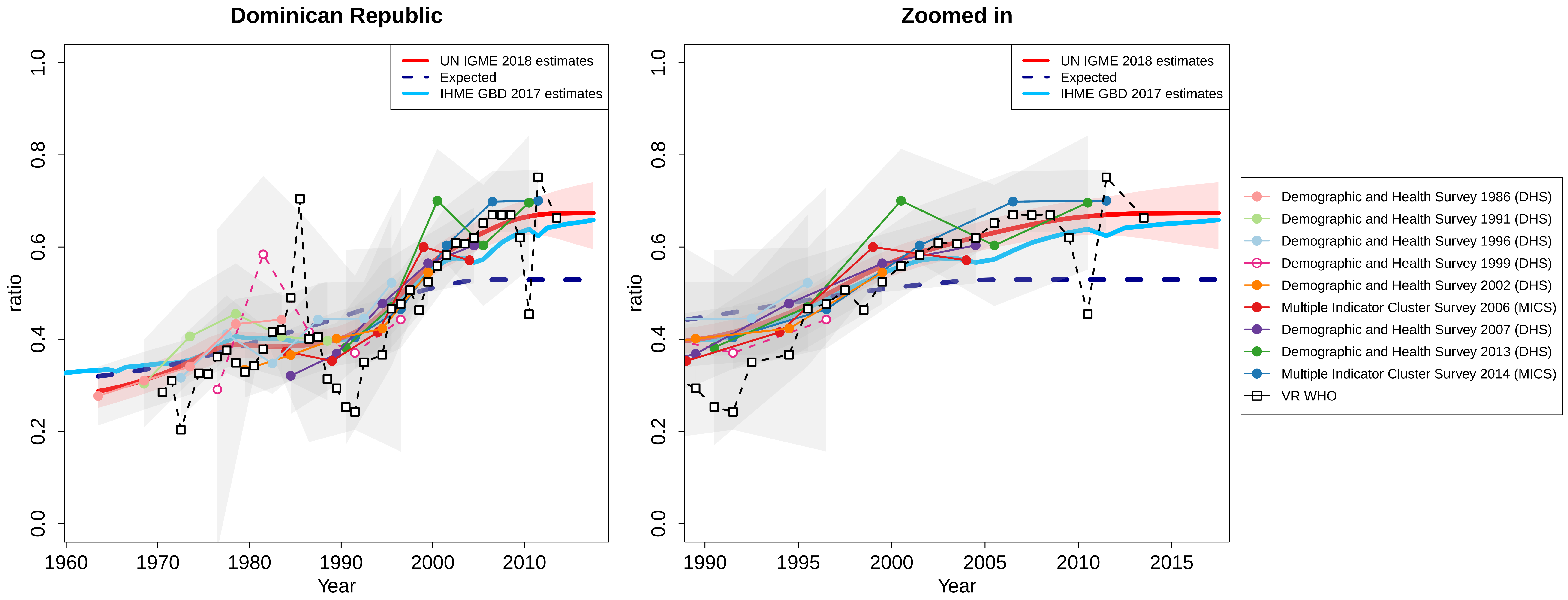
Dominican Republic
Zoomed in
UN IGME 2018 estimates
Expected
IHME GBD 2017 estimates
ratio
Year
1960
1970
1980
1990
2000
2010
1995
2005
2015
0.0
0.2
0.4
0.6
0.8
1.0
Demographic and Health Survey 1986 (DHS)
Demographic and Health Survey 1991 (DHS)
Demographic and Health Survey 1996 (DHS)
Demographic and Health Survey 1999 (DHS)
Demographic and Health Survey 2002 (DHS)
Multiple Indicator Cluster Survey 2006 (MICS)
Demographic and Health Survey 2007 (DHS)
Demographic and Health Survey 2013 (DHS)
Multiple Indicator Cluster Survey 2014 (MICS)
VR WHO

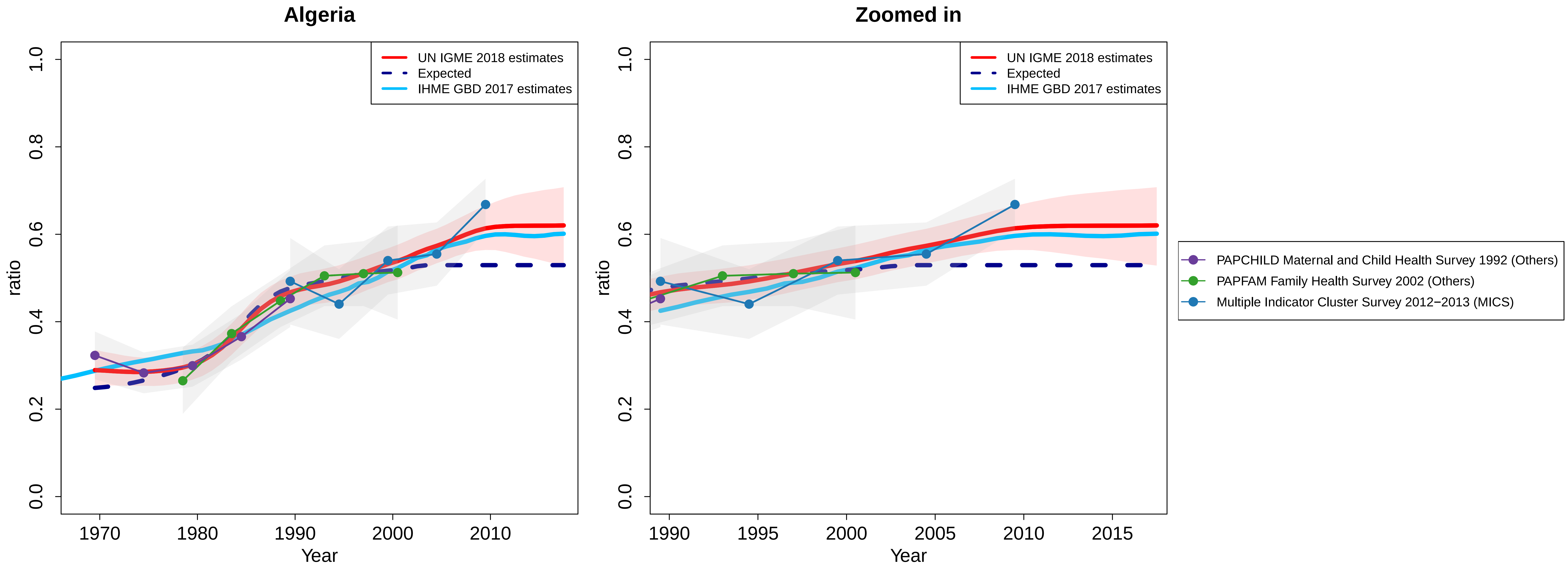
Algeria
Zoomed in
UN IGME 2018 estimates
Expected
IHME GBD 2017 estimates
ratio
Year
1970
1980
1990
2000
2010
1995
2005
2015
0.0
0.2
0.4
0.6
0.8
1.0
PAPCHILD Maternal and Child Health Survey 1992 (Others)
PAPFAM Family Health Survey 2002 (Others)
Multiple Indicator Cluster Survey 2012−2013 (MICS)

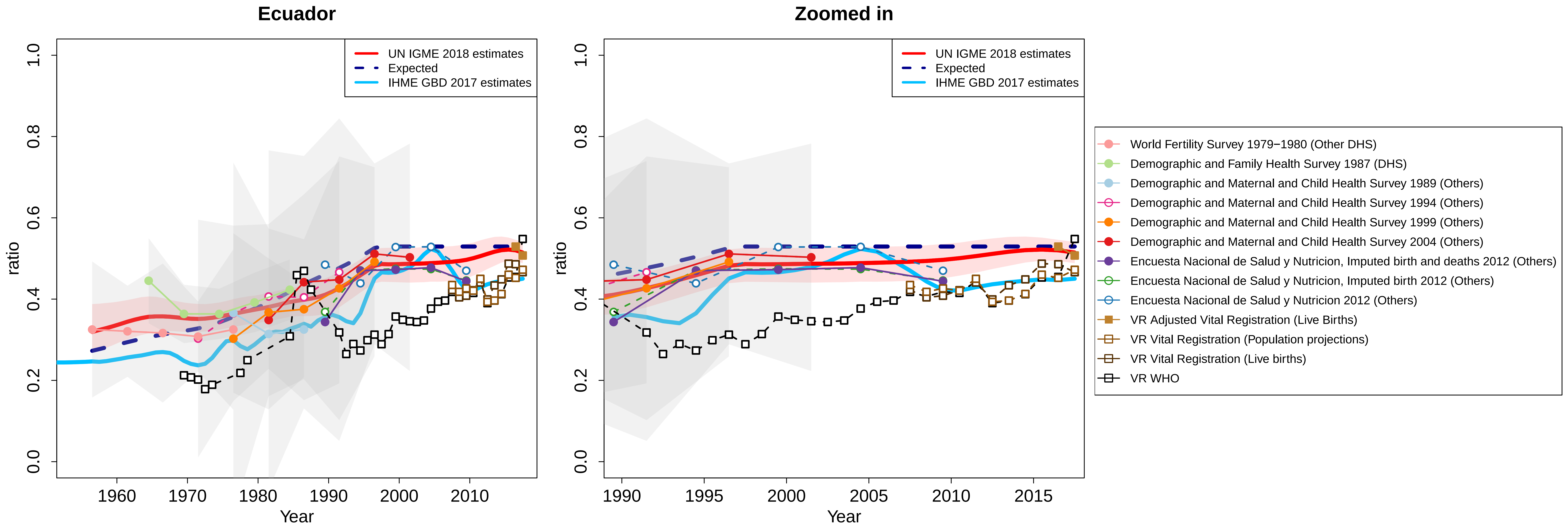

Ecuador
Zoomed in
ratio
Year
1.0
0.8
0.6
0.4
0.2
0.0
1960
1970
1980
1990
2000
2010
1995
2005
2015
UN IGME 2018 estimates
Expected
IHME GBD 2017 estimates
World Fertility Survey 1979−1980 (Other DHS)
Demographic and Family Health Survey 1987 (DHS)
Demographic and Maternal and Child Health Survey 1989 (Others)
Demographic and Maternal and Child Health Survey 1994 (Others)
Demographic and Maternal and Child Health Survey 1999 (Others)
Demographic and Maternal and Child Health Survey 2004 (Others)
Encuesta Nacional de Salud y Nutricion, Imputed birth and deaths 2012 (Others)
Encuesta Nacional de Salud y Nutricion, Imputed birth 2012 (Others)
Encuesta Nacional de Salud y Nutricion 2012 (Others)
VR Adjusted Vital Registration (Live Births)
VR Vital Registration (Population projections)
VR Vital Registration (Live births)
VR WHO

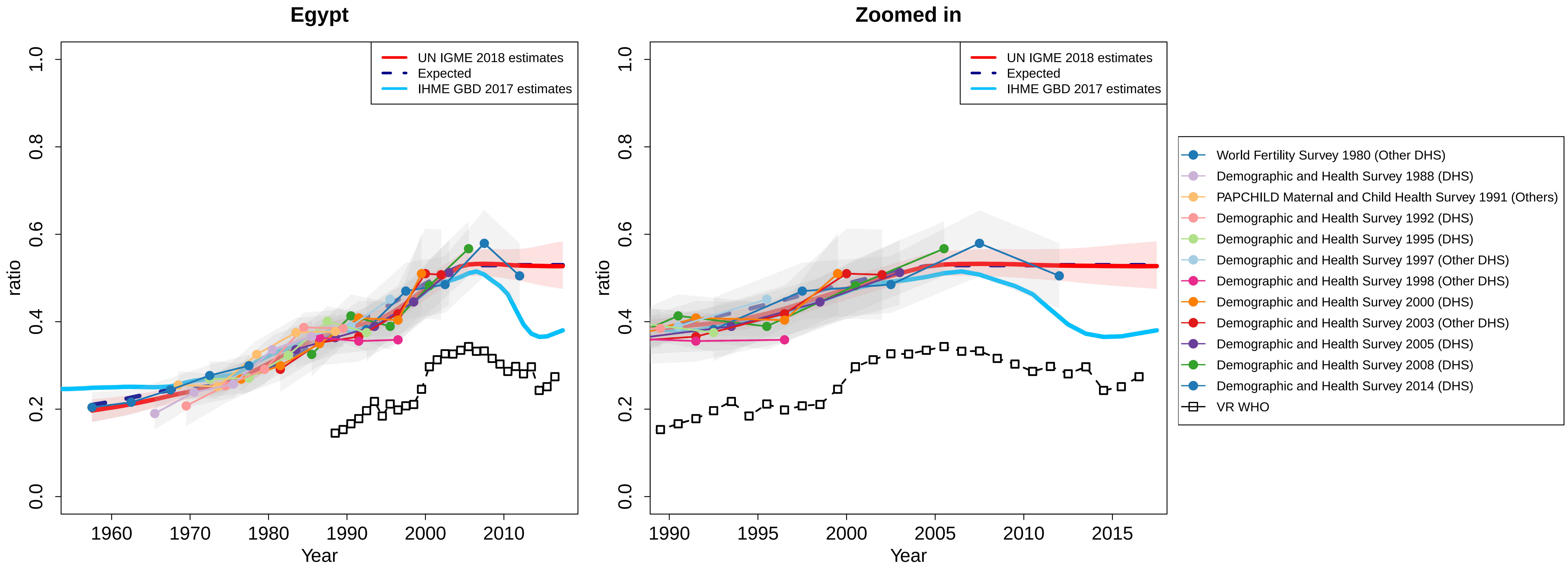

Egypt
Zoomed in
UN IGME 2018 estimates
Expected
IHME GBD 2017 estimates
ratio
Year
World Fertility Survey 1980 (Other DHS)
Demographic and Health Survey 1988 (DHS)
PAPCHILD Maternal and Child Health Survey 1991 (Others)
Demographic and Health Survey 1992 (DHS)
Demographic and Health Survey 1995 (DHS)
Demographic and Health Survey 1997 (Other DHS)
Demographic and Health Survey 1998 (Other DHS)
Demographic and Health Survey 2000 (DHS)
Demographic and Health Survey 2003 (Other DHS)
Demographic and Health Survey 2005 (DHS)
Demographic and Health Survey 2008 (DHS)
Demographic and Health Survey 2014 (DHS)
VR WHO

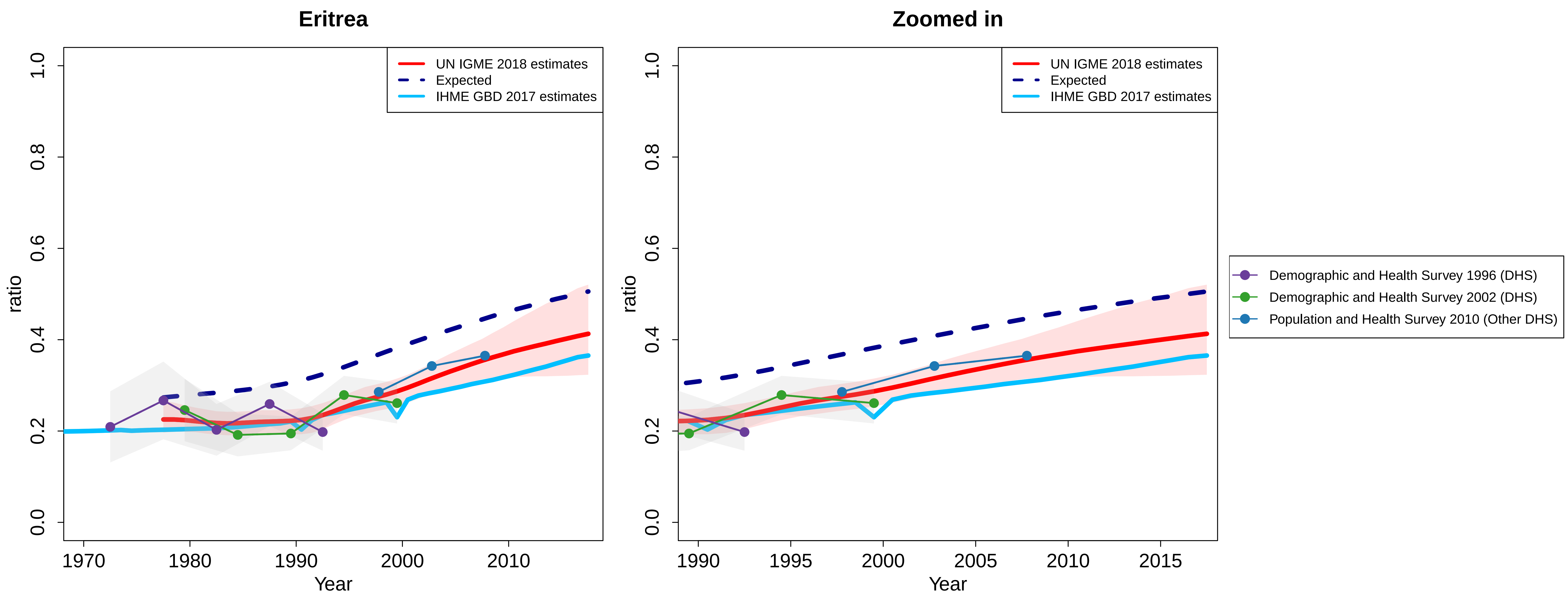
Eritrea
Zoomed in
UN IGME 2018 estimates
Expected
IHME GBD 2017 estimates
ratio
Year
0.0
0.2
0.4
0.6
0.8
1.0
1970
1980
1990
2000
2010
1995
2005
2015
Demographic and Health Survey 1996 (DHS)
Demographic and Health Survey 2002 (DHS)
Population and Health Survey 2010 (Other DHS)

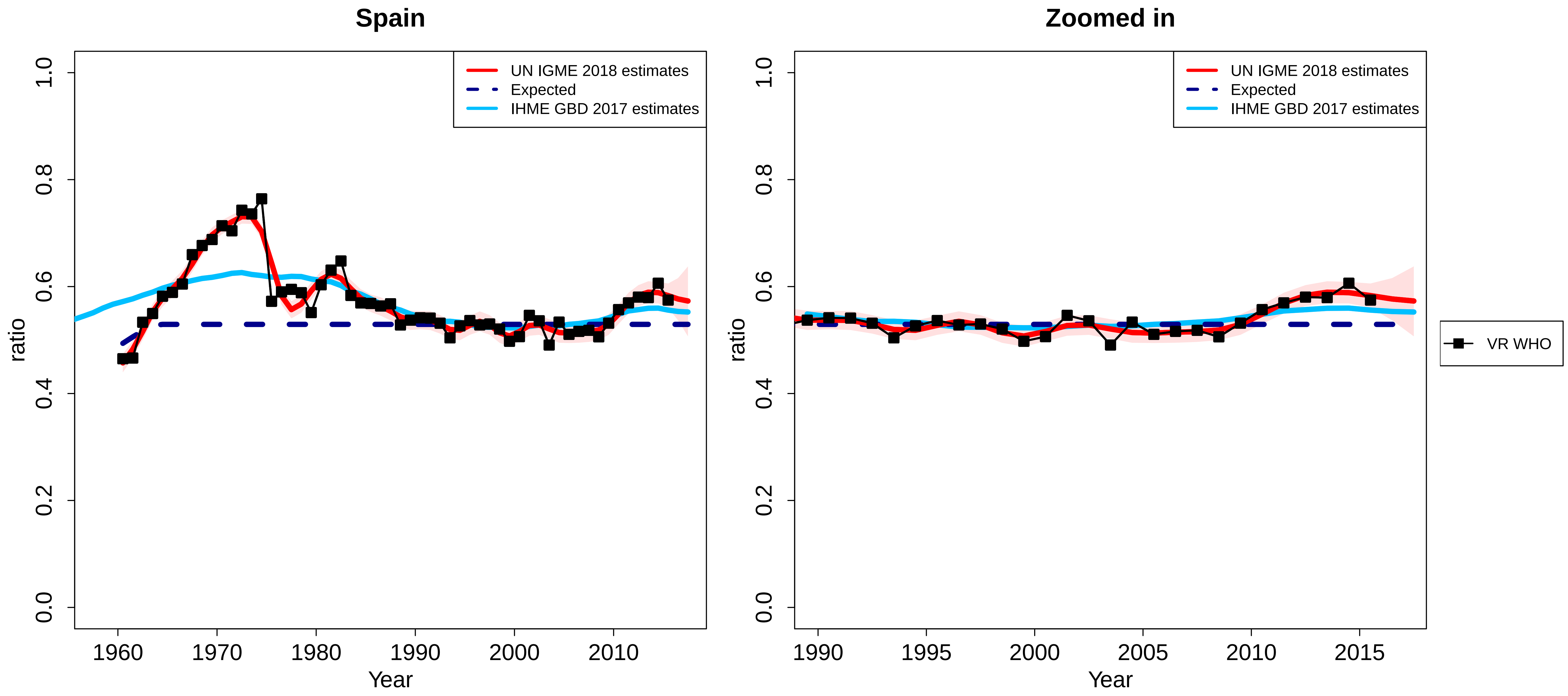
Spain
Zoomed in
UN IGME 2018 estimates
Expected
IHME GBD 2017 estimates
VR WHO
ratio
Year
0.0
0.2
0.4
0.6
0.8
1.0
1960
1970
1980
1990
2000
2010
1995
2005
2015

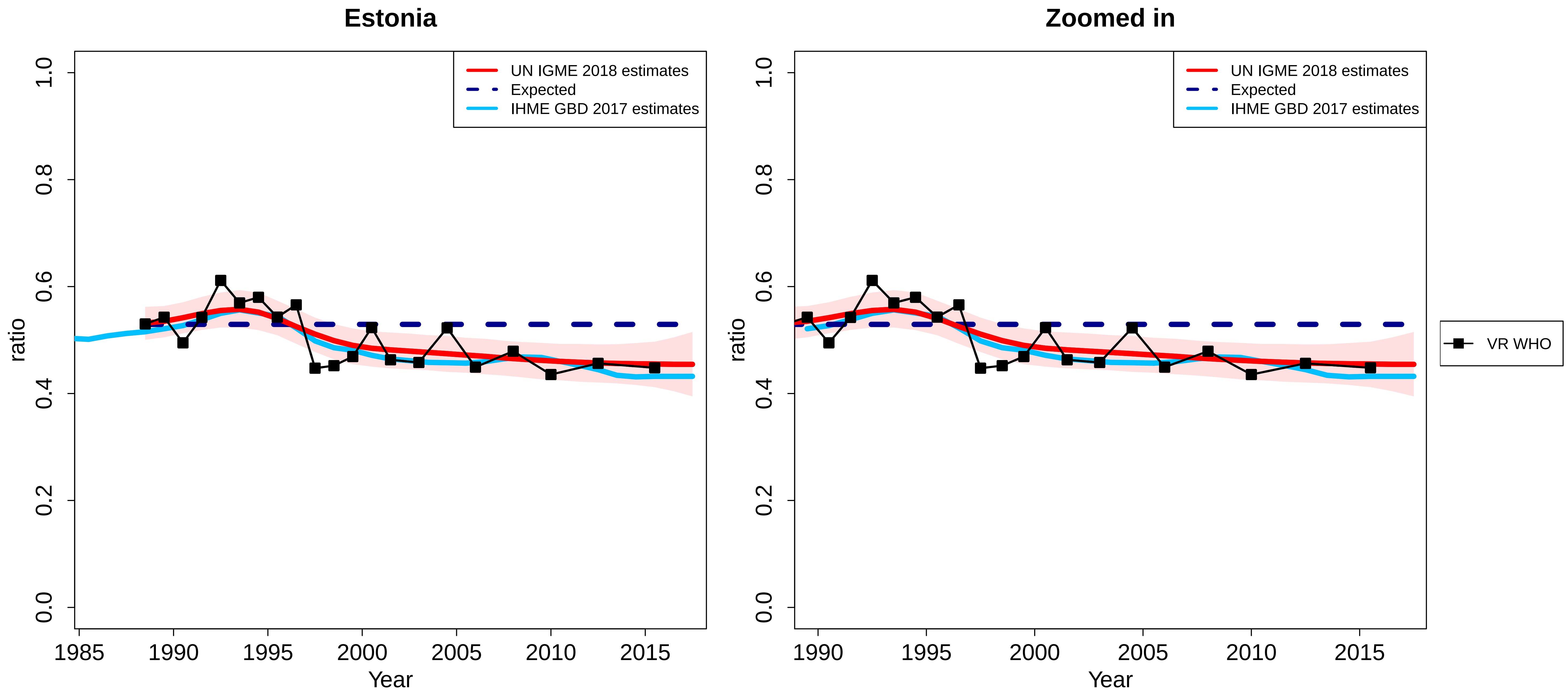
Estonia
Zoomed in
UN IGME 2018 estimates
Expected
IHME GBD 2017 estimates
VR WHO
ratio
Year
0.0
0.2
0.4
0.6
0.8
1.0
1985
1990
1995
2000
2005
2010
2015

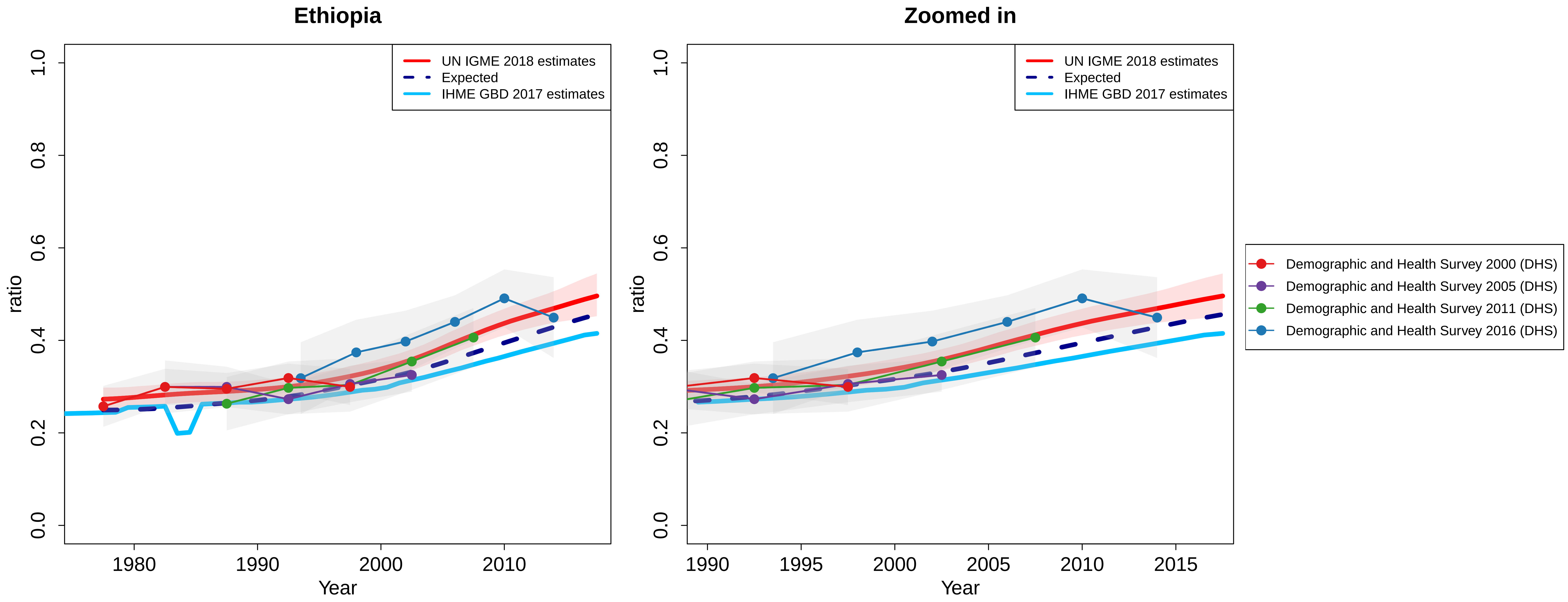

Ethiopia
Zoomed in
ratio
Year
UN IGME 2018 estimates
Expected
IHME GBD 2017 estimates
Demographic and Health Survey 2000 (DHS)
Demographic and Health Survey 2005 (DHS)
Demographic and Health Survey 2011 (DHS)
Demographic and Health Survey 2016 (DHS)
0.0
0.2
0.4
0.6
0.8
1.0
1980
1990
2000
2010
1995
2005
2015

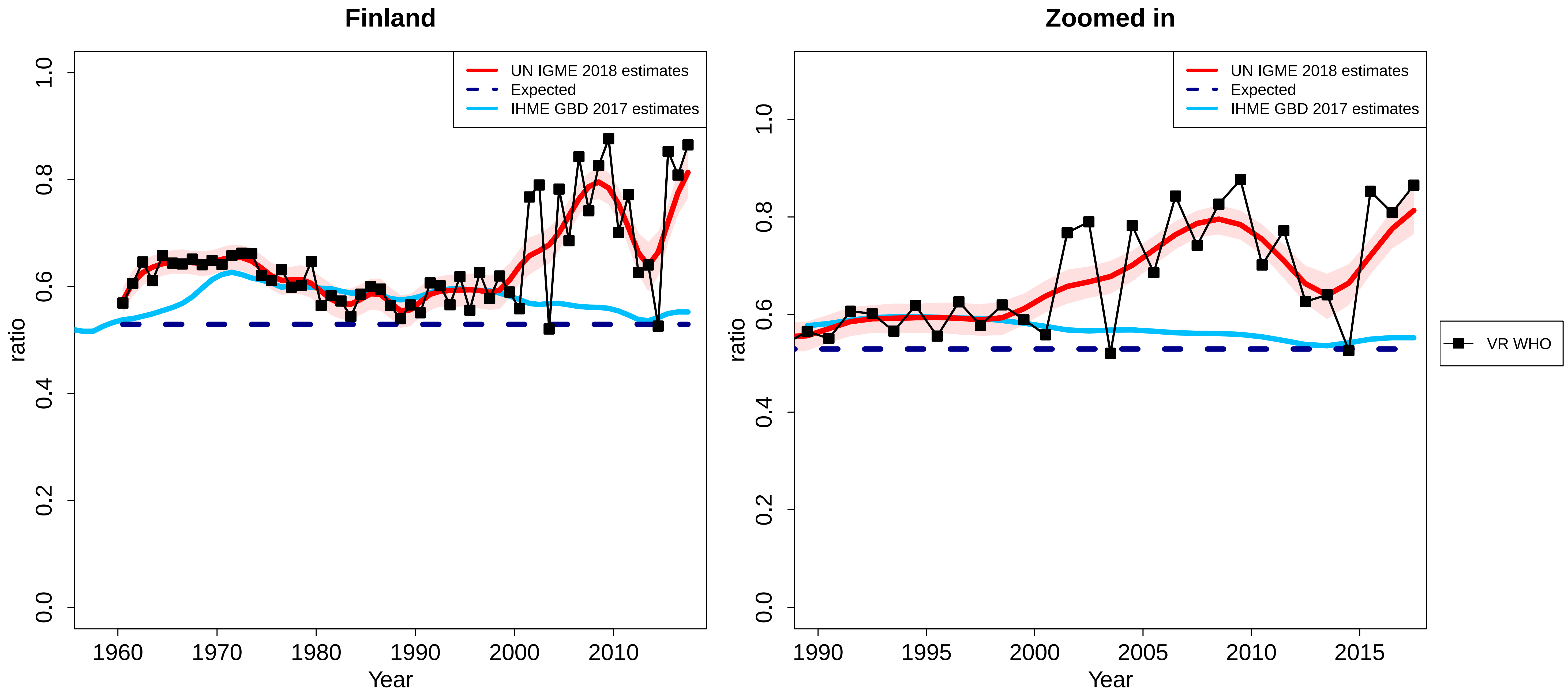

Finland
UN IGME 2018 estimates
Expected
IHME GBD 2017 estimates
ratio
0.0
0.2
0.4
0.6
0.8
1.0
1960
1970
1980
1990
2000
2010
Year
Zoomed in
1990
1995
2000
2005
2010
2015
VR WHO

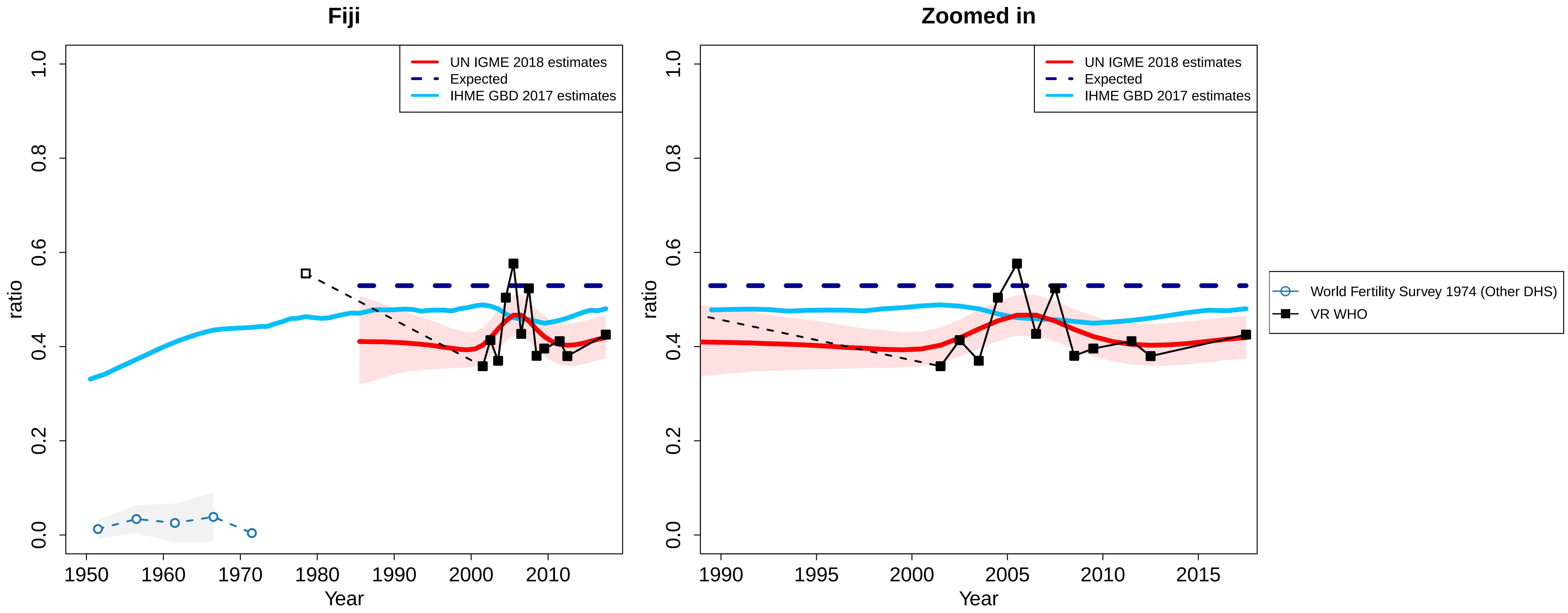
Fiji
Zoomed in
UN IGME 2018 estimates
Expected
IHME GBD 2017 estimates
World Fertility Survey 1974 (Other DHS)
VR WHO
ratio
Year
0.0
0.2
0.4
0.6
0.8
1.0
1950
1960
1970
1980
1990
2000
2010
1995
2005
2015

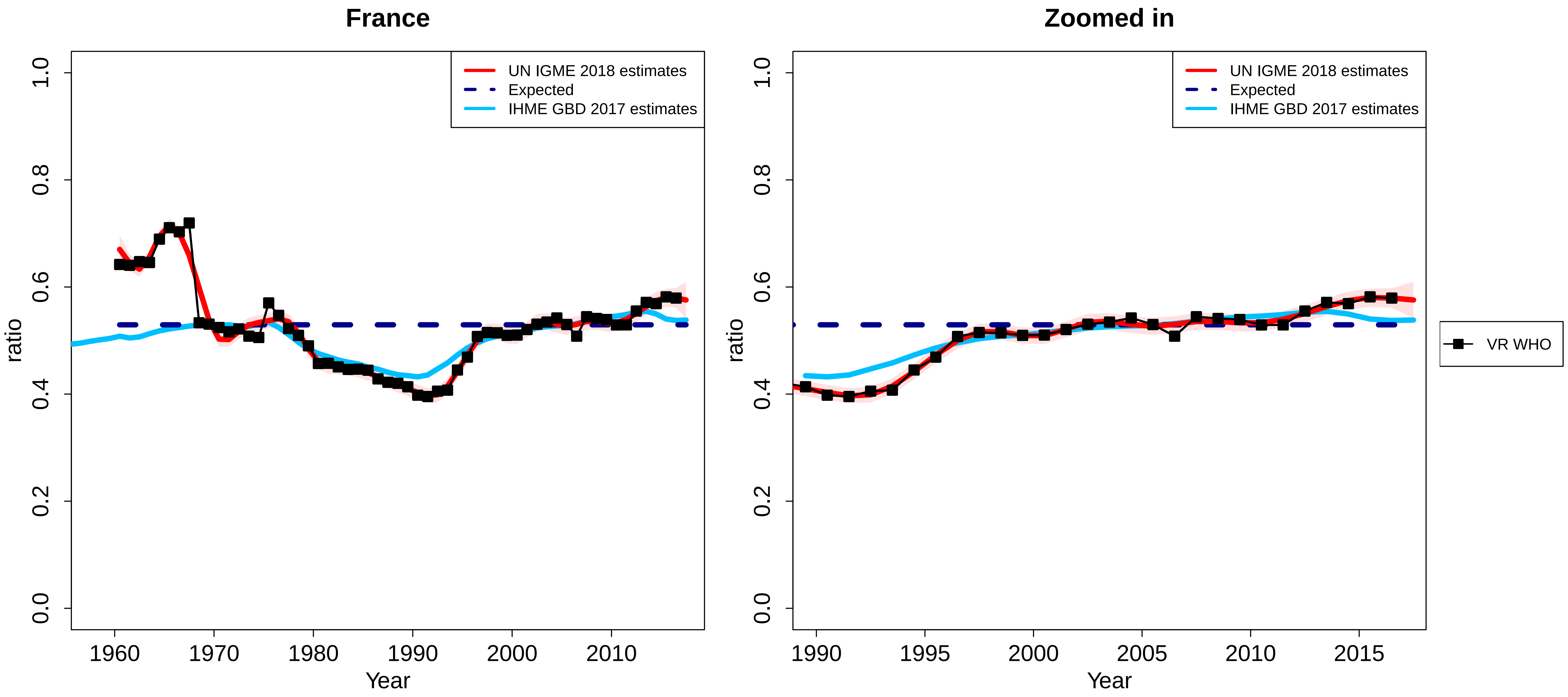
France
Zoomed in
UN IGME 2018 estimates
Expected
IHME GBD 2017 estimates
VR WHO
ratio
Year
0.0
0.2
0.4
0.6
0.8
1.0
1960
1970
1980
1990
2000
2010
1990
1995
2000
2005
2010
2015

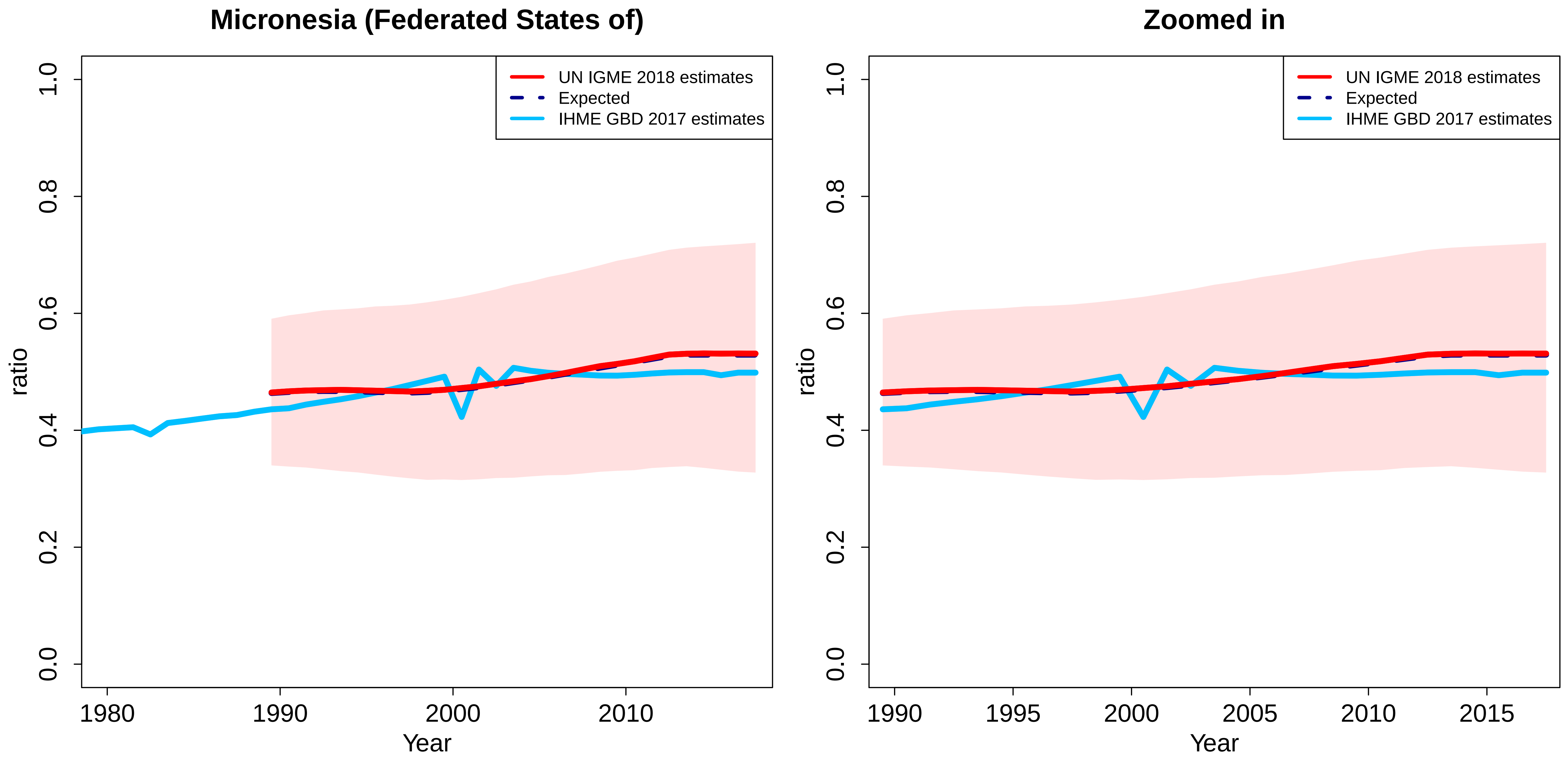
Micronesia (Federated States of)
Zoomed in
UN IGME 2018 estimates
Expected
IHME GBD 2017 estimates
ratio
Year
0.0
0.2
0.4
0.6
0.8
1.0
1980
1990
2000
2010
1995
2005
2015

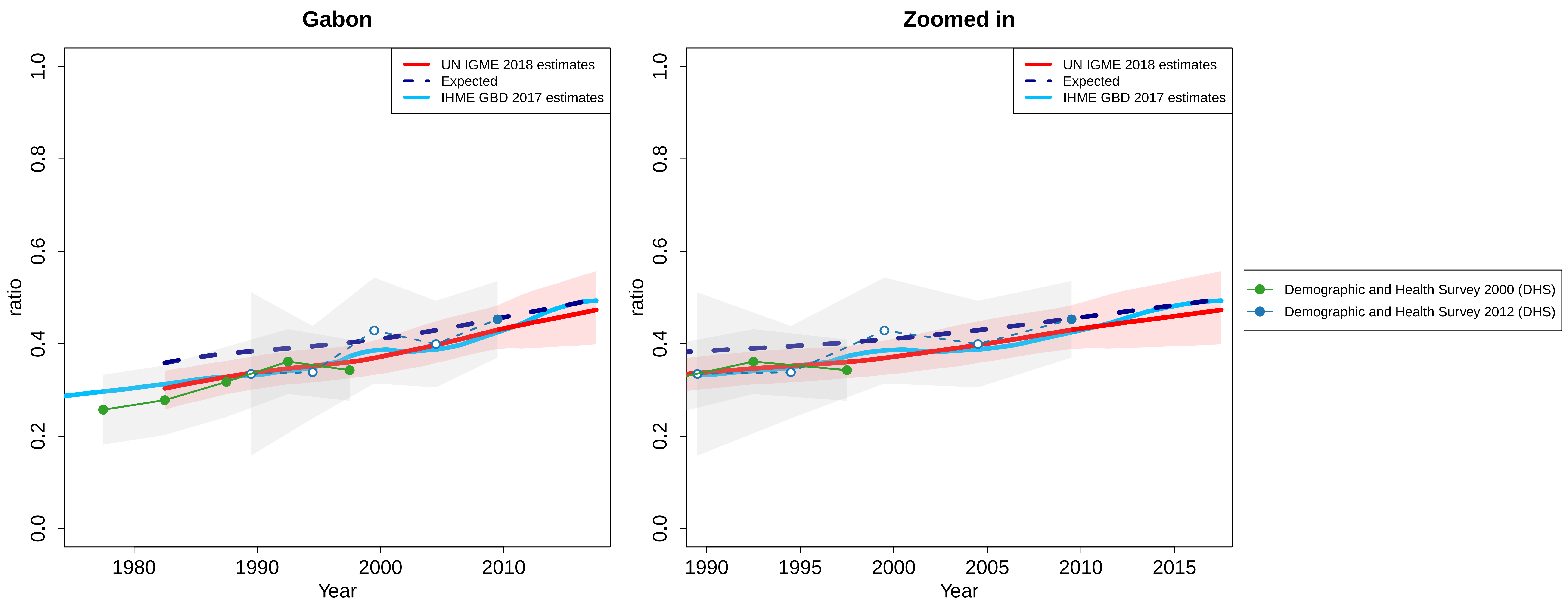

Gabon
Zoomed in
UN IGME 2018 estimates
Expected
IHME GBD 2017 estimates
ratio
Year
0.0
0.2
0.4
0.6
0.8
1.0
1980
1990
2000
2010
1995
2005
2015
Demographic and Health Survey 2000 (DHS)
Demographic and Health Survey 2012 (DHS)

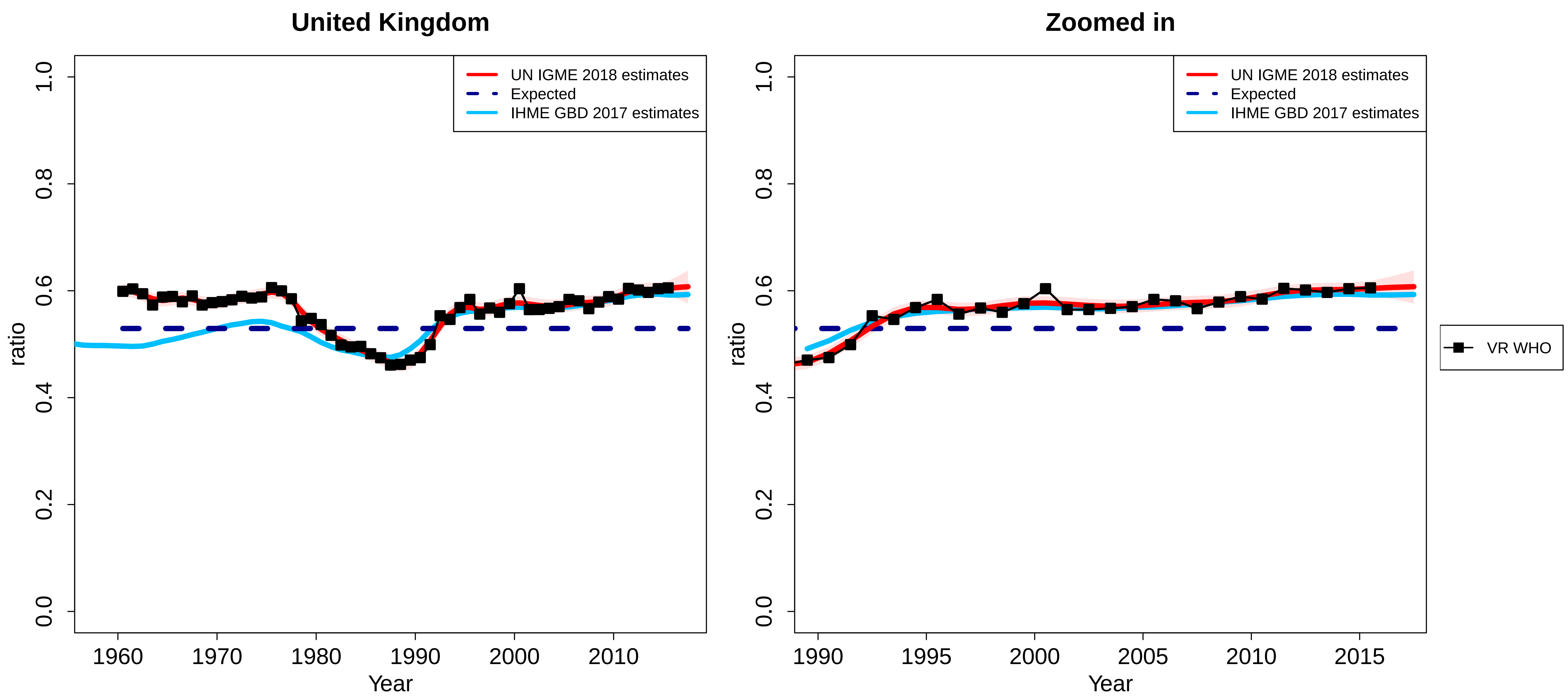
United Kingdom
Zoomed in
UN IGME 2018 estimates
Expected
IHME GBD 2017 estimates
VR WHO
ratio
Year
0.0
0.2
0.4
0.6
0.8
1.0
1960
1970
1980
1990
2000
2010
1990
1995
2000
2005
2010
2015

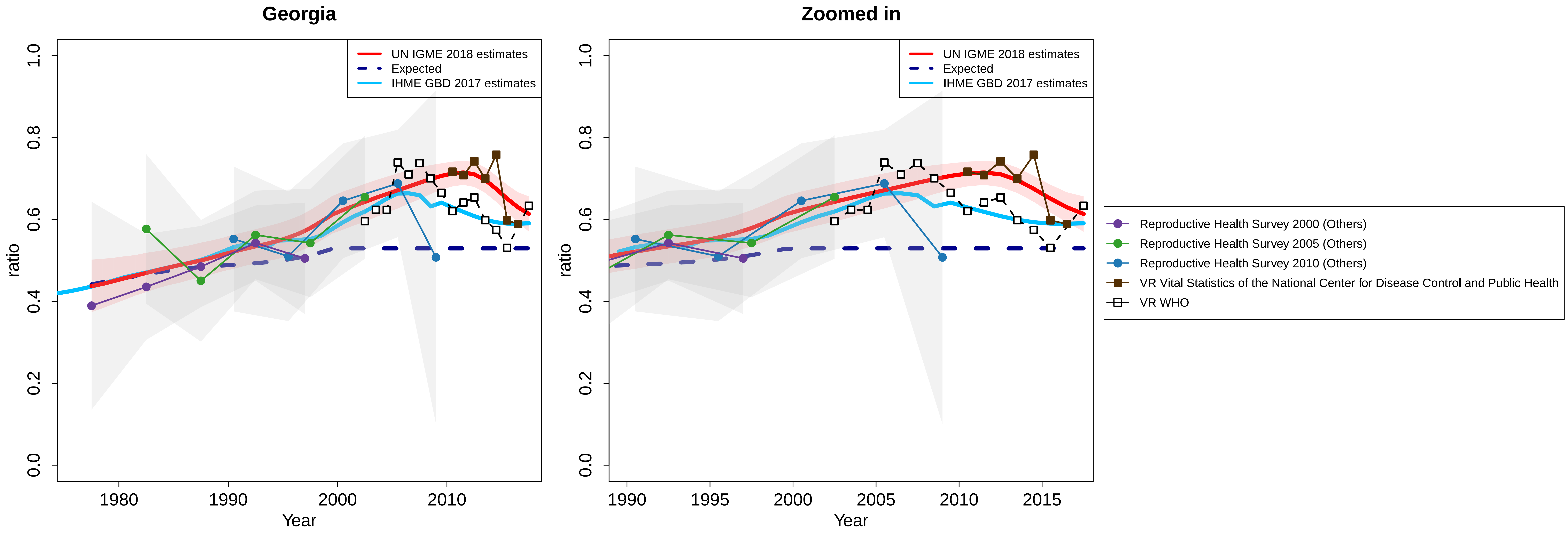

Georgia
Zoomed in
UN IGME 2018 estimates
Expected
IHME GBD 2017 estimates
ratio
Year
0.0
0.2
0.4
0.6
0.8
1.0
1980
1990
2000
2010
1995
2005
2015
Reproductive Health Survey 2000 (Others)
Reproductive Health Survey 2005 (Others)
Reproductive Health Survey 2010 (Others)
VR Vital Statistics of the National Center for Disease Control and Public Health
VR WHO

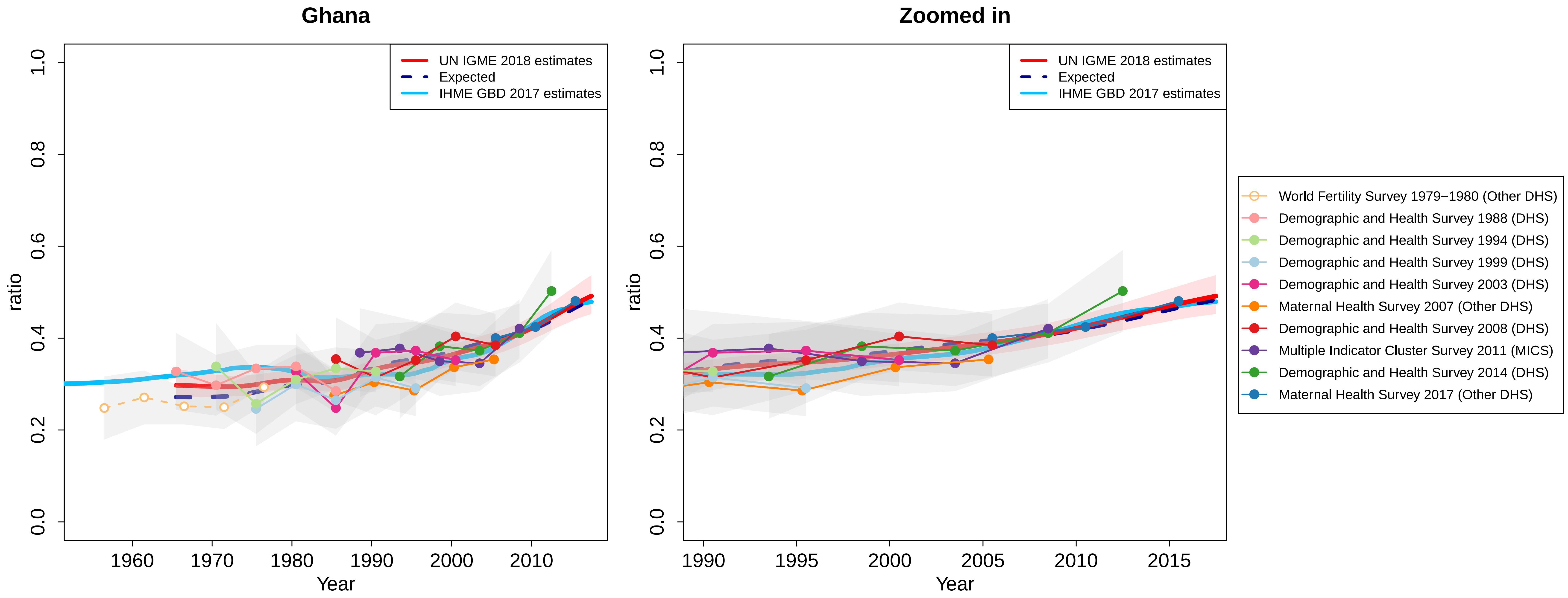
Ghana
Zoomed in
UN IGME 2018 estimates
Expected
IHME GBD 2017 estimates
ratio
Year
0.0
0.2
0.4
0.6
0.8
1.0
1960
1970
1980
1990
2000
2010
1995
2005
2015
World Fertility Survey 1979−1980 (Other DHS)
Demographic and Health Survey 1988 (DHS)
Demographic and Health Survey 1994 (DHS)
Demographic and Health Survey 1999 (DHS)
Demographic and Health Survey 2003 (DHS)
Maternal Health Survey 2007 (Other DHS)
Demographic and Health Survey 2008 (DHS)
Multiple Indicator Cluster Survey 2011 (MICS)
Demographic and Health Survey 2014 (DHS)
Maternal Health Survey 2017 (Other DHS)

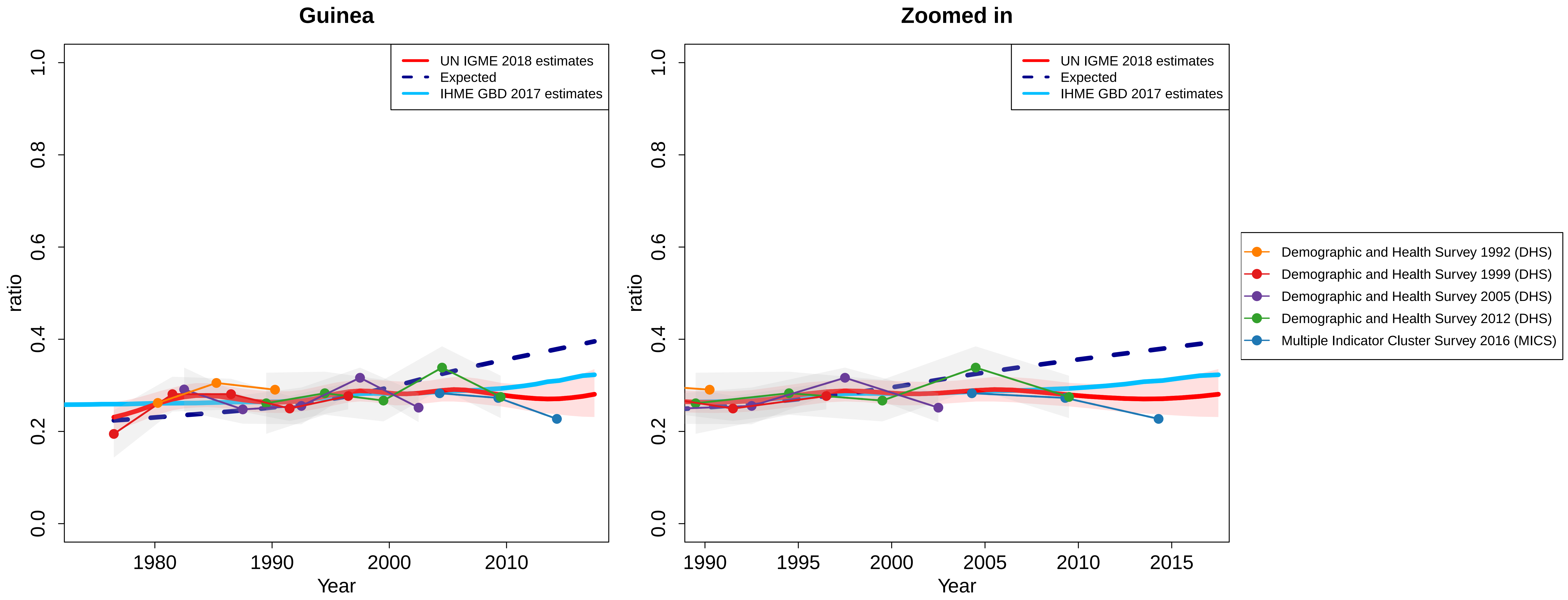
Guinea
Zoomed in
ratio
Year
UN IGME 2018 estimates
Expected
IHME GBD 2017 estimates
Demographic and Health Survey 1992 (DHS)
Demographic and Health Survey 1999 (DHS)
Demographic and Health Survey 2005 (DHS)
Demographic and Health Survey 2012 (DHS)
Multiple Indicator Cluster Survey 2016 (MICS)

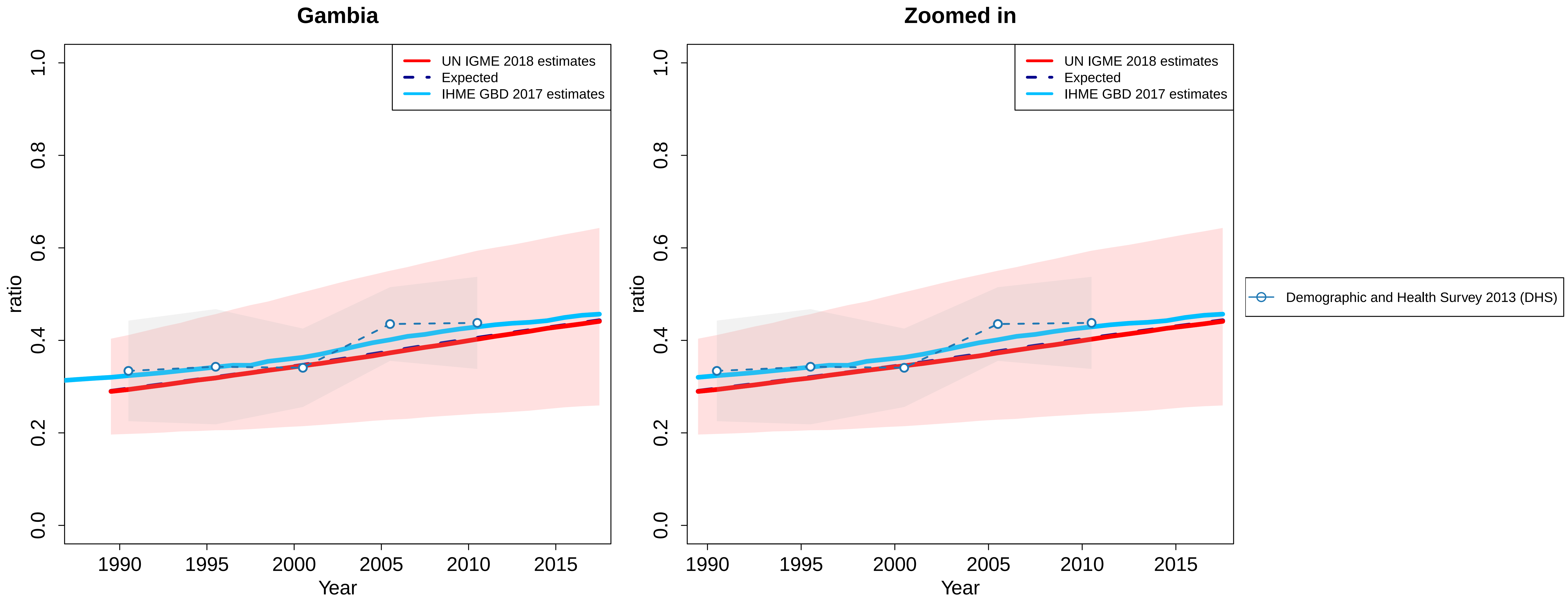

Gambia
Zoomed in
UN IGME 2018 estimates
Expected
IHME GBD 2017 estimates
Demographic and Health Survey 2013 (DHS)
ratio
Year
0.0
0.2
0.4
0.6
0.8
1.0
1990
1995
2000
2005
2010
2015

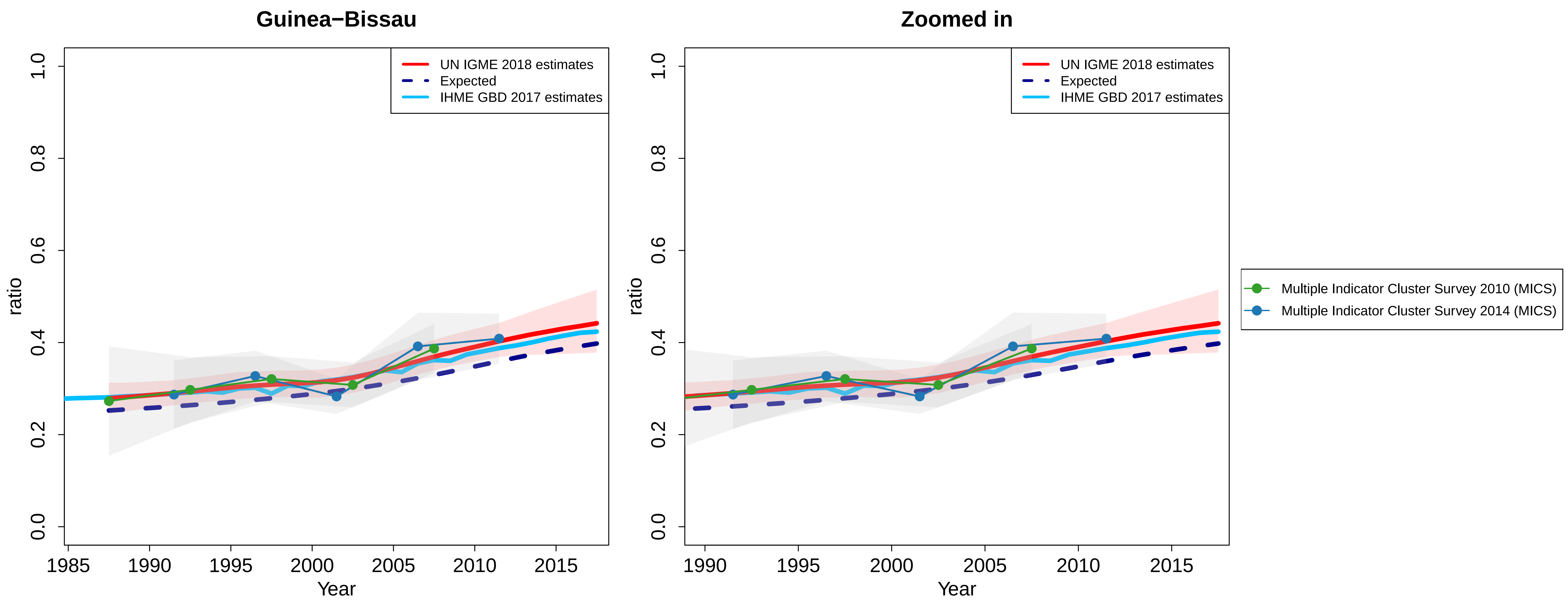

Guinea−Bissau
Zoomed in
UN IGME 2018 estimates
Expected
IHME GBD 2017 estimates
Multiple Indicator Cluster Survey 2010 (MICS)
Multiple Indicator Cluster Survey 2014 (MICS)
ratio
Year
0.0
0.2
0.4
0.6
0.8
1.0
1985
1990
1995
2000
2005
2010
2015

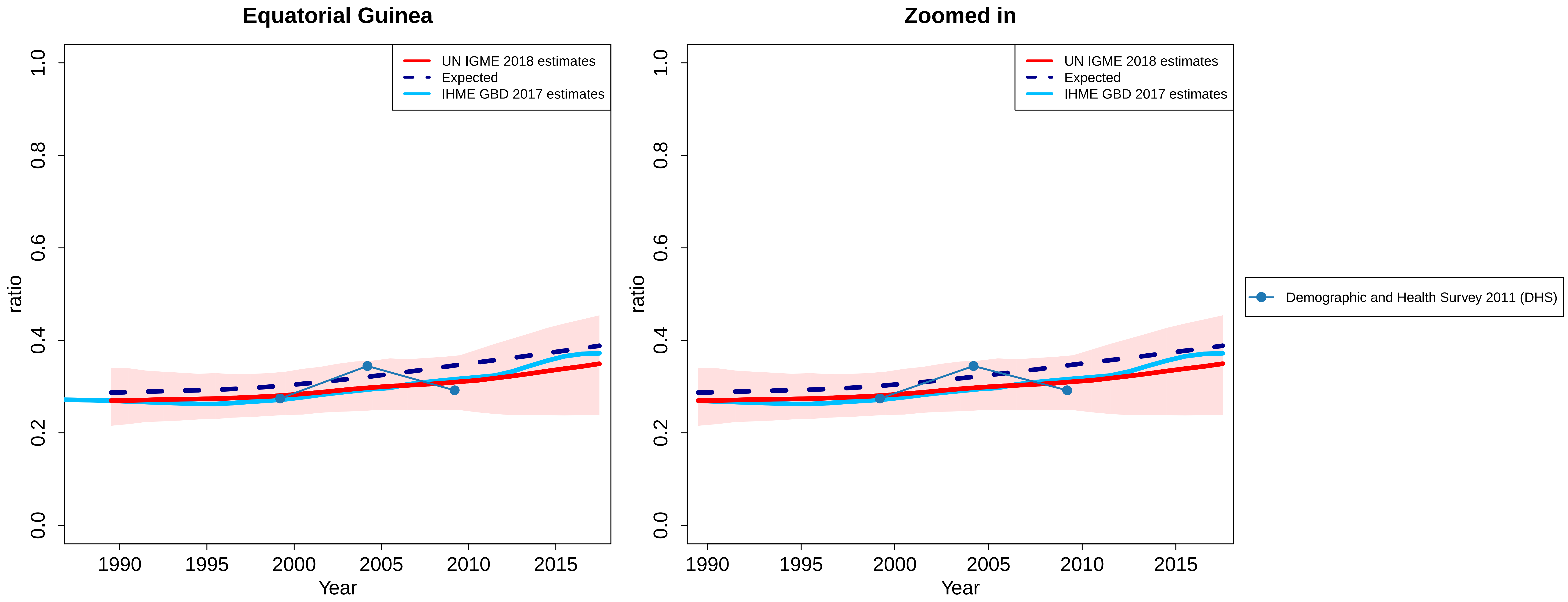

Equatorial Guinea
Zoomed in
ratio
Year
0.0
0.2
0.4
0.6
0.8
1.0
1990
1995
2000
2005
2010
2015
UN IGME 2018 estimates
Expected
IHME GBD 2017 estimates
Demographic and Health Survey 2011 (DHS)

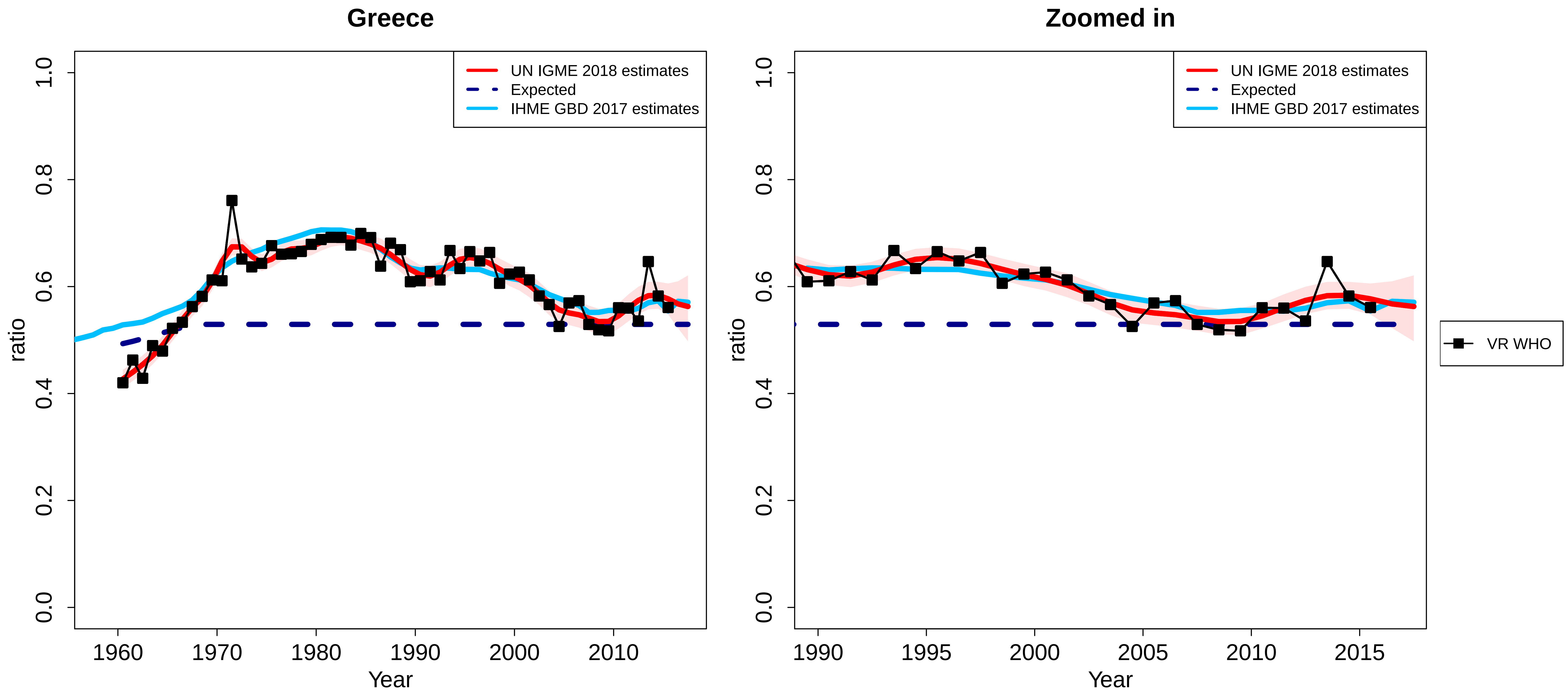
Greece
Zoomed in
UN IGME 2018 estimates
Expected
IHME GBD 2017 estimates
VR WHO
ratio
Year
0.0
0.2
0.4
0.6
0.8
1.0
1960
1970
1980
1990
2000
2010
1995
2005
2015

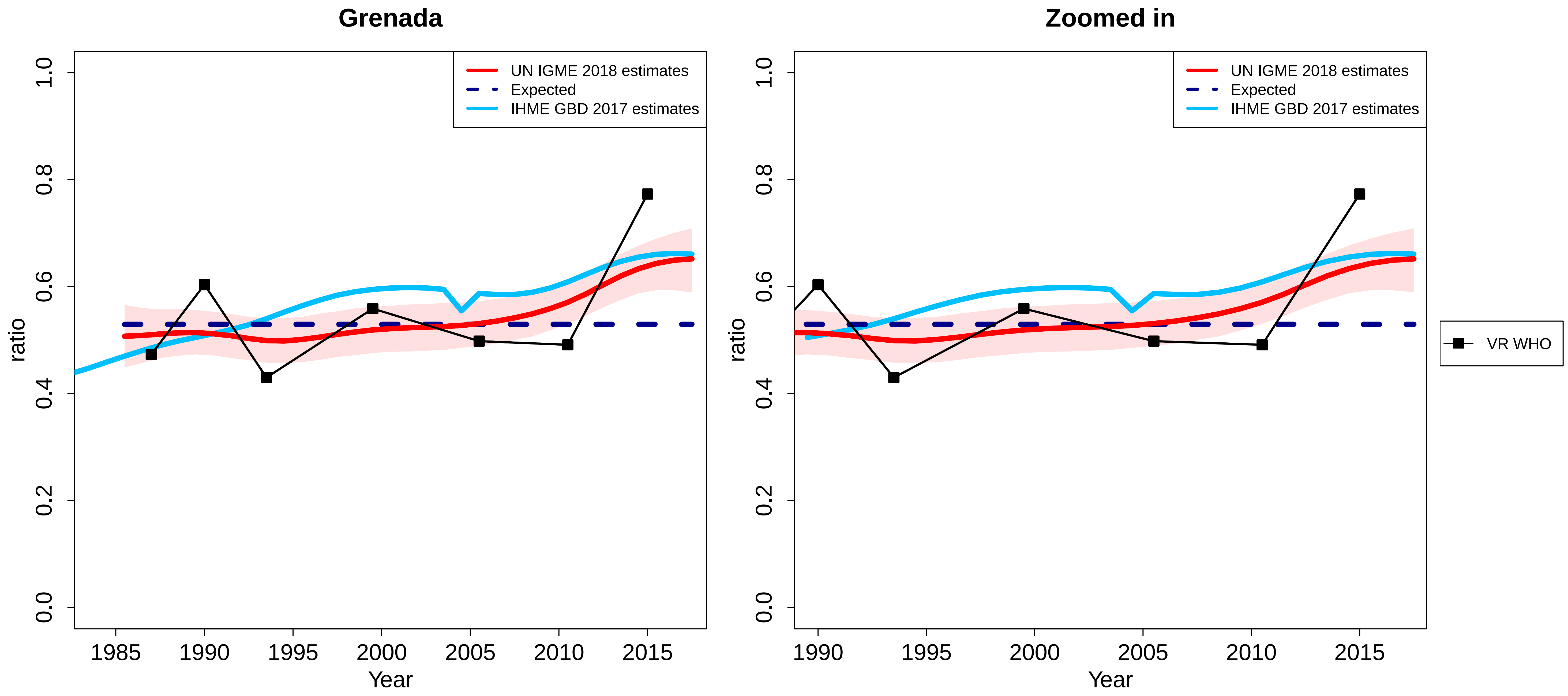

Grenada
Zoomed in
UN IGME 2018 estimates
Expected
IHME GBD 2017 estimates
ratio
Year
0.0
0.2
0.4
0.6
0.8
1.0
1985
1990
1995
2000
2005
2010
2015
VR WHO

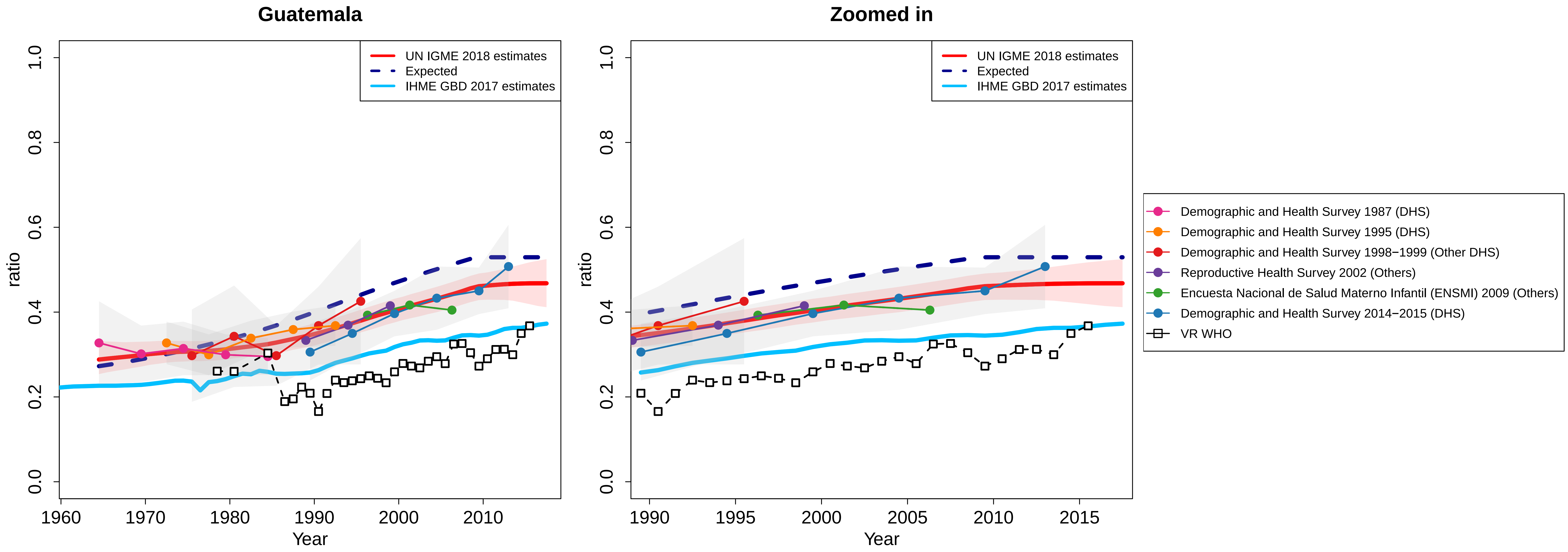
Guatemala
Zoomed in
UN IGME 2018 estimates
Expected
IHME GBD 2017 estimates
ratio
Year
1960
1970
1980
1990
2000
2010
1995
2005
2015
0.0
0.2
0.4
0.6
0.8
1.0
Demographic and Health Survey 1987 (DHS)
Demographic and Health Survey 1995 (DHS)
Demographic and Health Survey 1998−1999 (Other DHS)
Reproductive Health Survey 2002 (Others)
Encuesta Nacional de Salud Materno Infantil (ENSMI) 2009 (Others)
Demographic and Health Survey 2014−2015 (DHS)
VR WHO

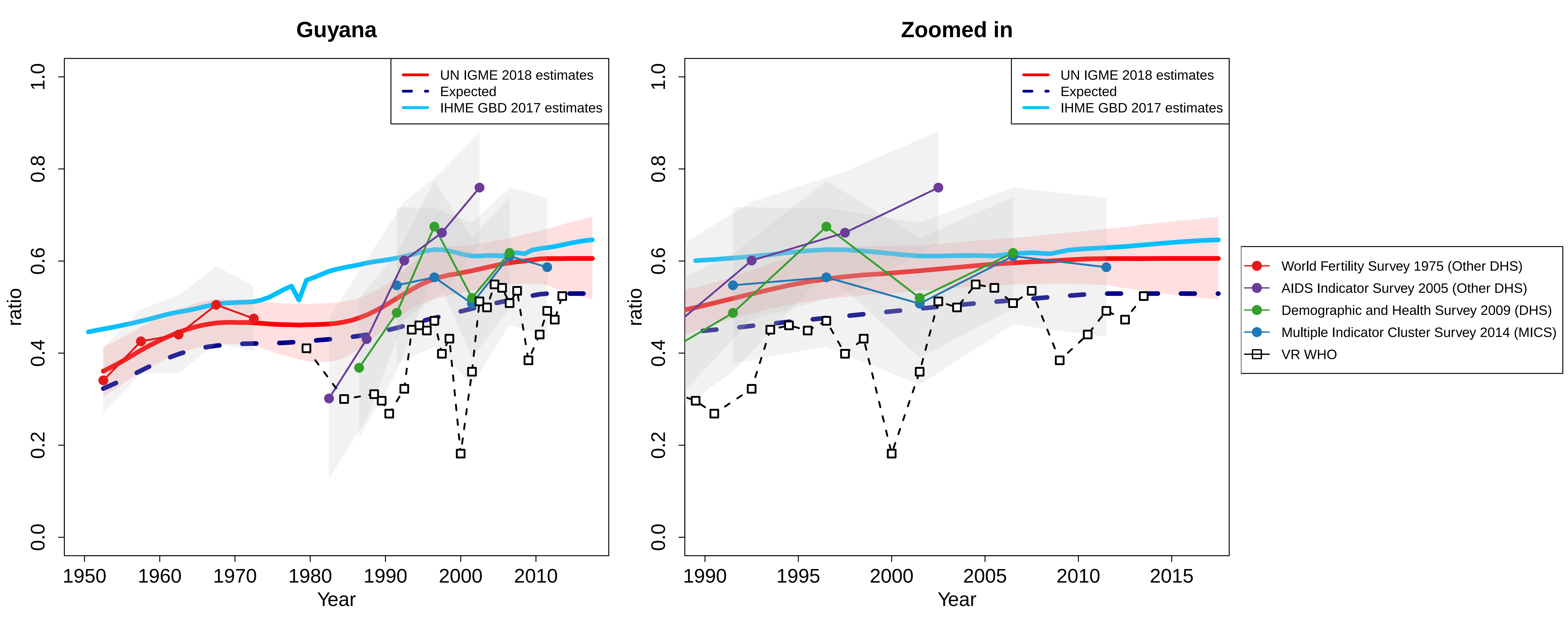

Guyana
Zoomed in
UN IGME 2018 estimates
Expected
IHME GBD 2017 estimates
ratio
Year
World Fertility Survey 1975 (Other DHS)
AIDS Indicator Survey 2005 (Other DHS)
Demographic and Health Survey 2009 (DHS)
Multiple Indicator Cluster Survey 2014 (MICS)
VR WHO

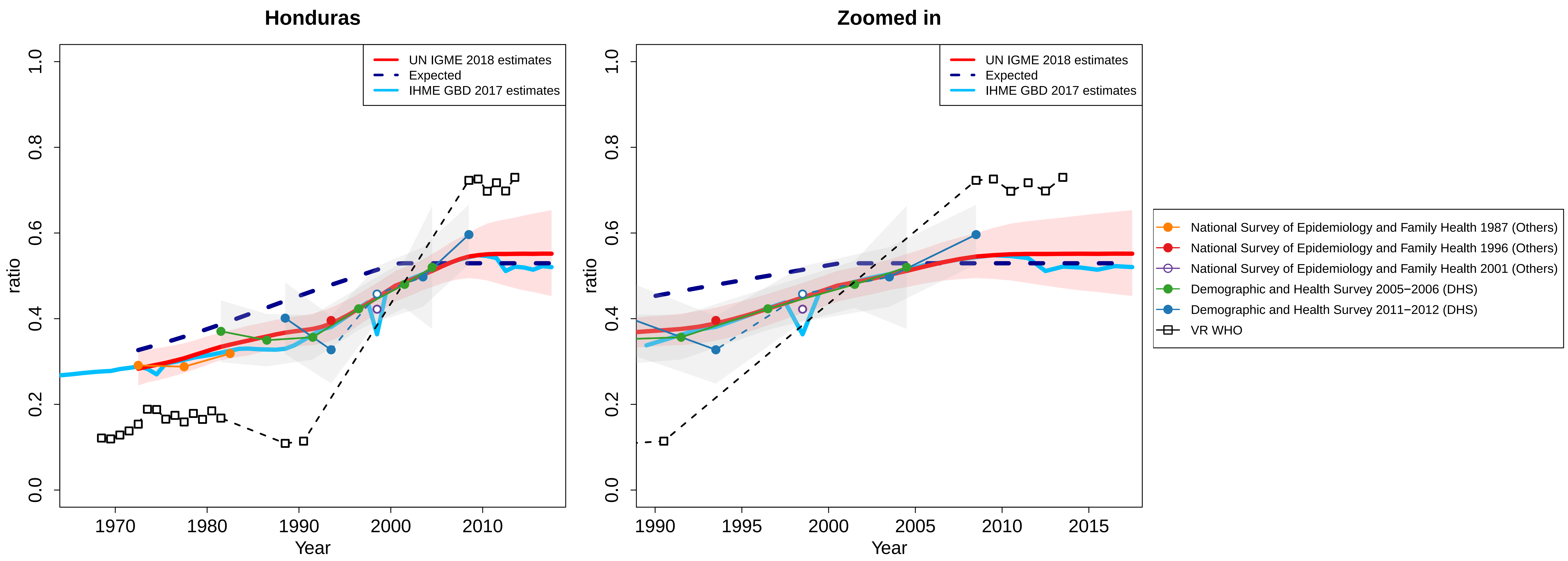

Honduras
Zoomed in
UN IGME 2018 estimates
Expected
IHME GBD 2017 estimates
ratio
Year
0.0
0.2
0.4
0.6
0.8
1.0
1970
1980
1990
2000
2010
1995
2005
2015
National Survey of Epidemiology and Family Health 1987 (Others)
National Survey of Epidemiology and Family Health 1996 (Others)
National Survey of Epidemiology and Family Health 2001 (Others)
Demographic and Health Survey 2005−2006 (DHS)
Demographic and Health Survey 2011−2012 (DHS)
VR WHO

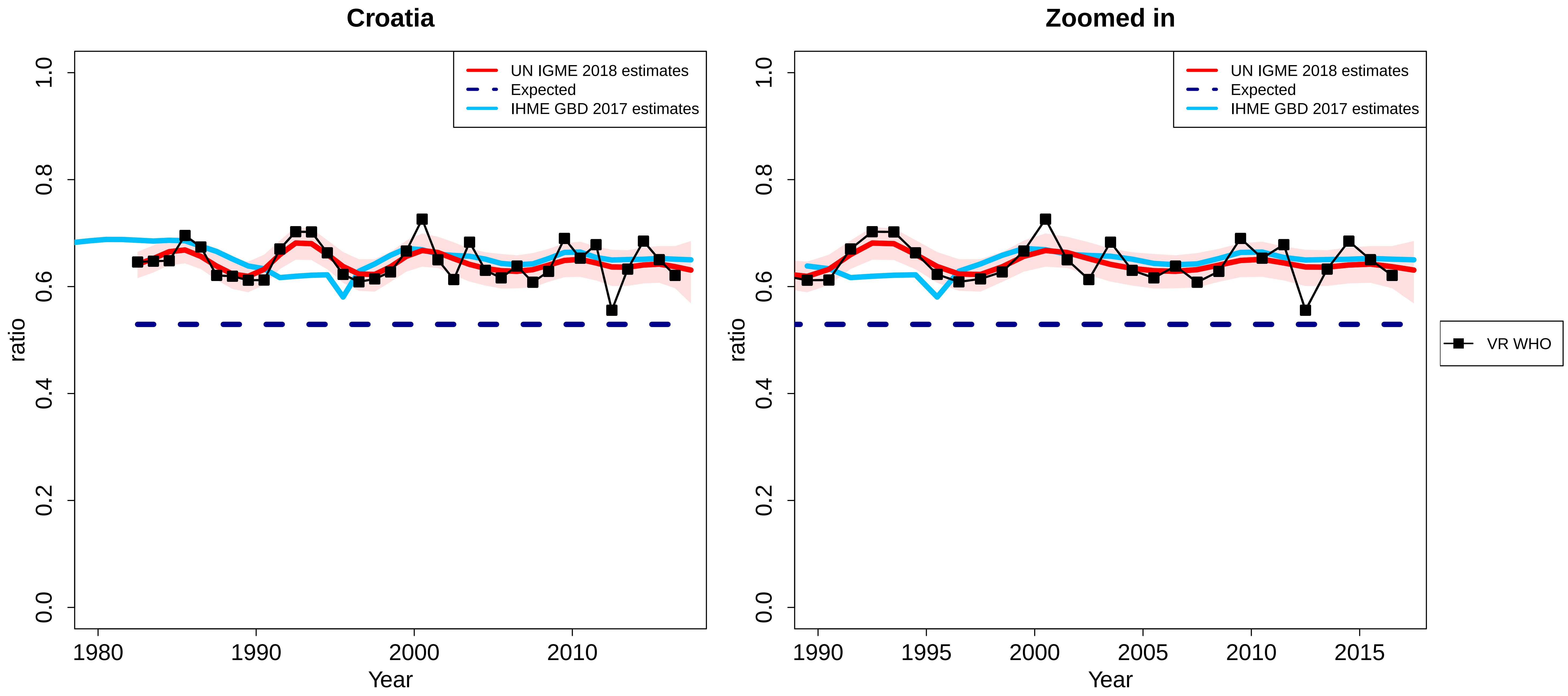

Croatia
Zoomed in
UN IGME 2018 estimates
Expected
IHME GBD 2017 estimates
VR WHO
ratio
Year
0.0
0.2
0.4
0.6
0.8
1.0
1980
1990
2000
2010
1995
2005
2015

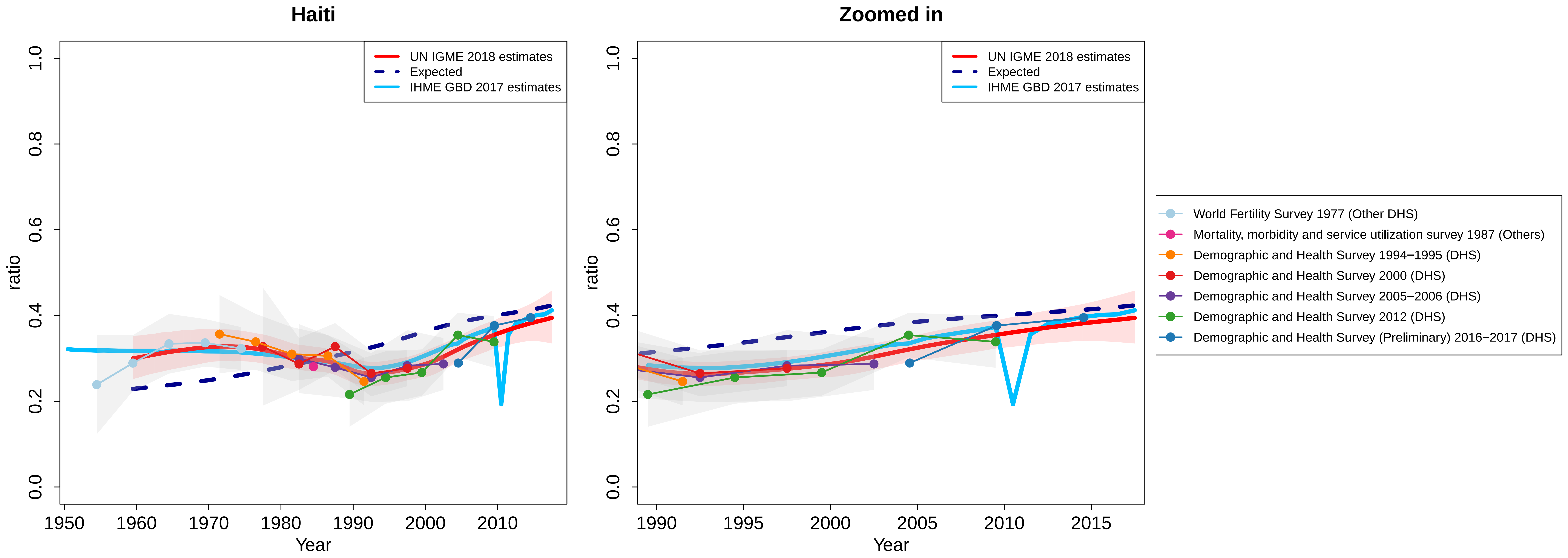
Haiti
Zoomed in
UN IGME 2018 estimates
Expected
IHME GBD 2017 estimates
ratio
Year
World Fertility Survey 1977 (Other DHS)
Mortality, morbidity and service utilization survey 1987 (Others)
Demographic and Health Survey 1994−1995 (DHS)
Demographic and Health Survey 2000 (DHS)
Demographic and Health Survey 2005−2006 (DHS)
Demographic and Health Survey 2012 (DHS)
Demographic and Health Survey (Preliminary) 2016−2017 (DHS)

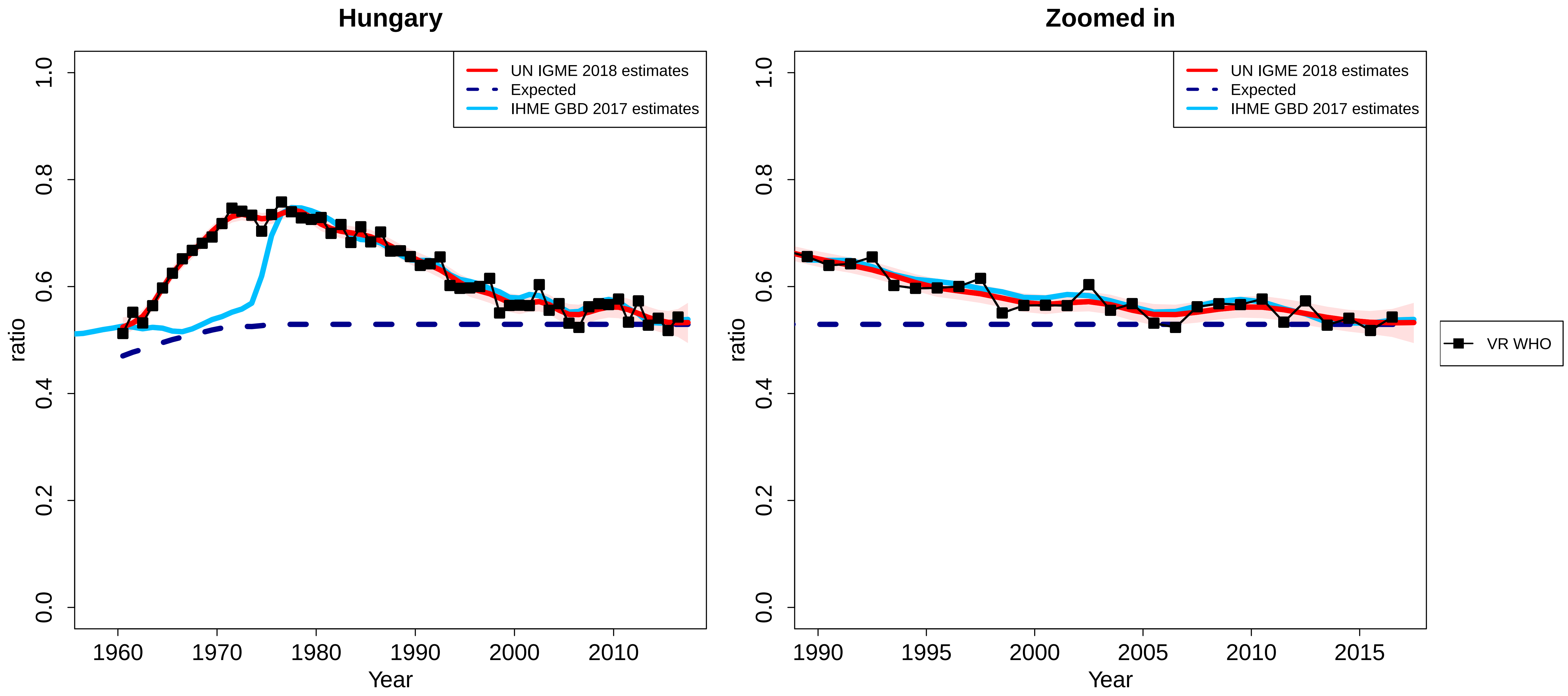
Hungary
Zoomed in
UN IGME 2018 estimates
Expected
IHME GBD 2017 estimates
VR WHO
ratio
Year
0.0
0.2
0.4
0.6
0.8
1.0
1960
1970
1980
1990
2000
2010
1995
2005
2015

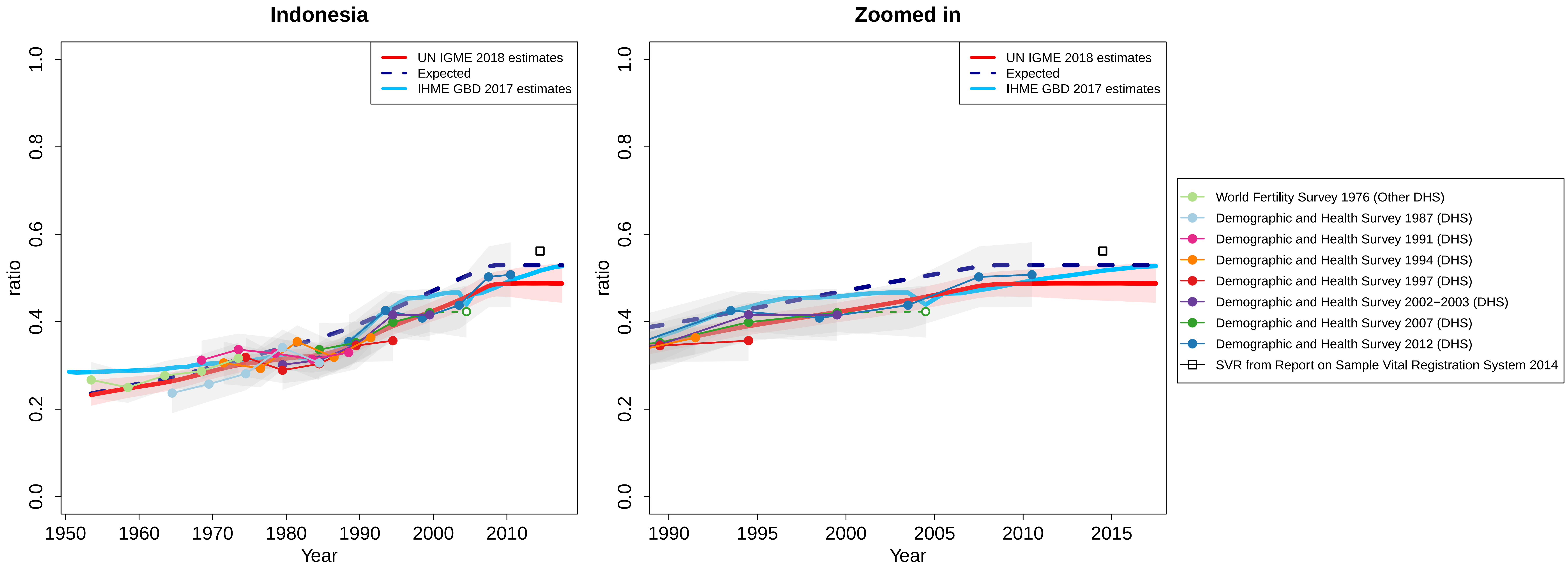

Indonesia
Zoomed in
UN IGME 2018 estimates
Expected
IHME GBD 2017 estimates
ratio
Year
World Fertility Survey 1976 (Other DHS)
Demographic and Health Survey 1987 (DHS)
Demographic and Health Survey 1991 (DHS)
Demographic and Health Survey 1994 (DHS)
Demographic and Health Survey 1997 (DHS)
Demographic and Health Survey 2002−2003 (DHS)
Demographic and Health Survey 2007 (DHS)
Demographic and Health Survey 2012 (DHS)
SVR from Report on Sample Vital Registration System 2014

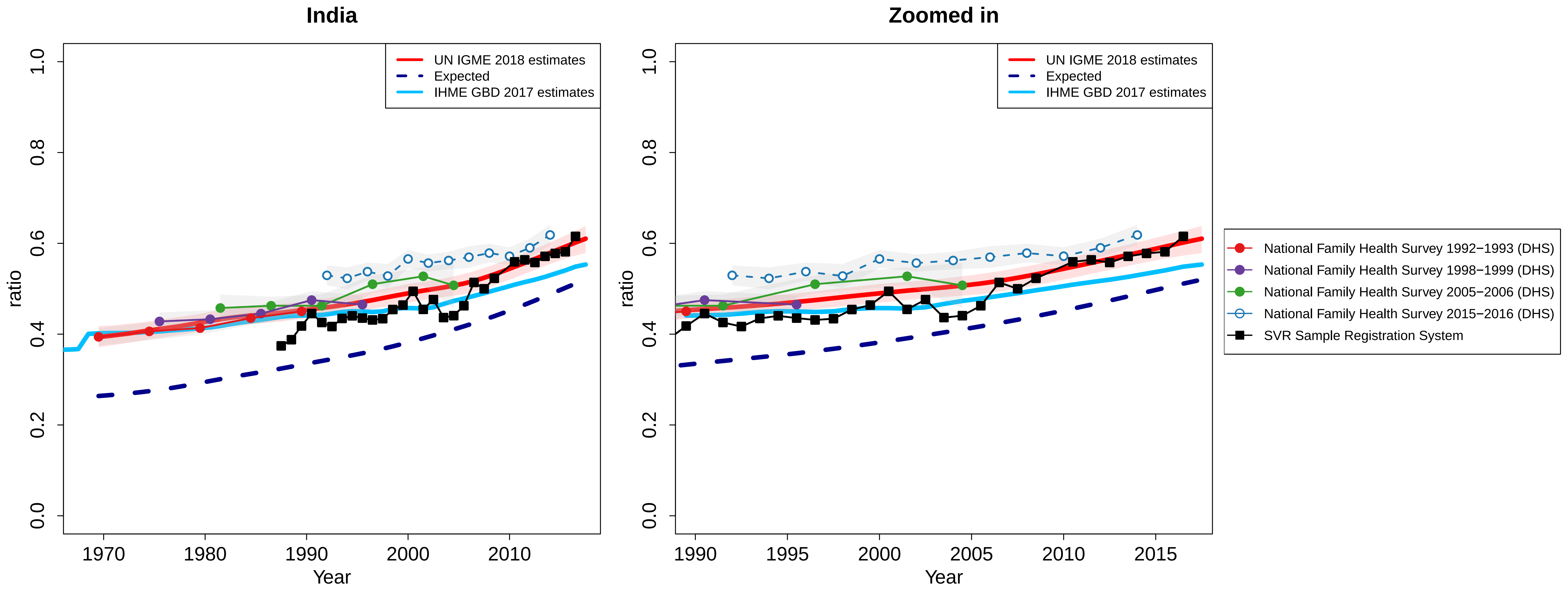
India
Zoomed in
UN IGME 2018 estimates
Expected
IHME GBD 2017 estimates
ratio
Year
National Family Health Survey 1992−1993 (DHS)
National Family Health Survey 1998−1999 (DHS)
National Family Health Survey 2005−2006 (DHS)
National Family Health Survey 2015−2016 (DHS)
SVR Sample Registration System

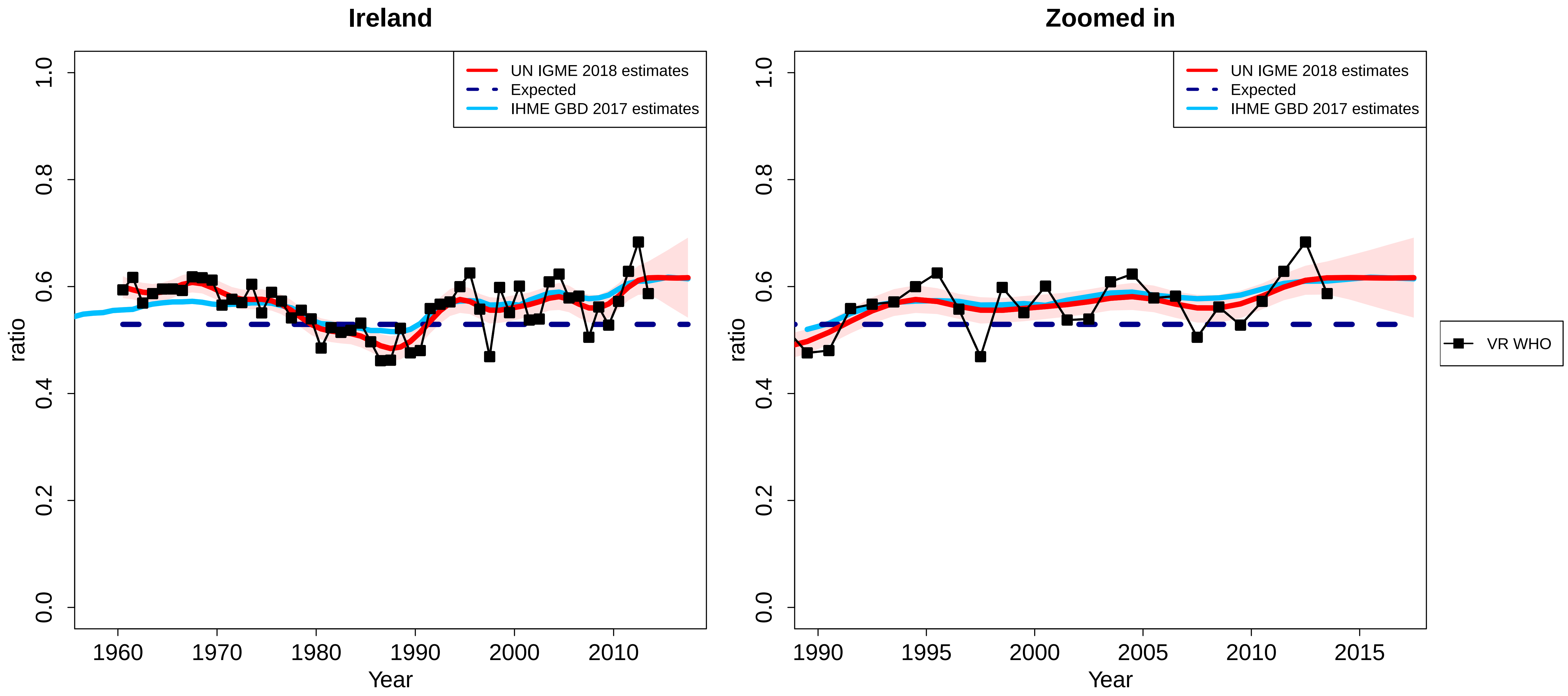
Ireland
Zoomed in
UN IGME 2018 estimates
Expected
IHME GBD 2017 estimates
VR WHO
ratio
Year
0.0
0.2
0.4
0.6
0.8
1.0
1960
1970
1980
1990
2000
2010
1990
1995
2000
2005
2010
2015

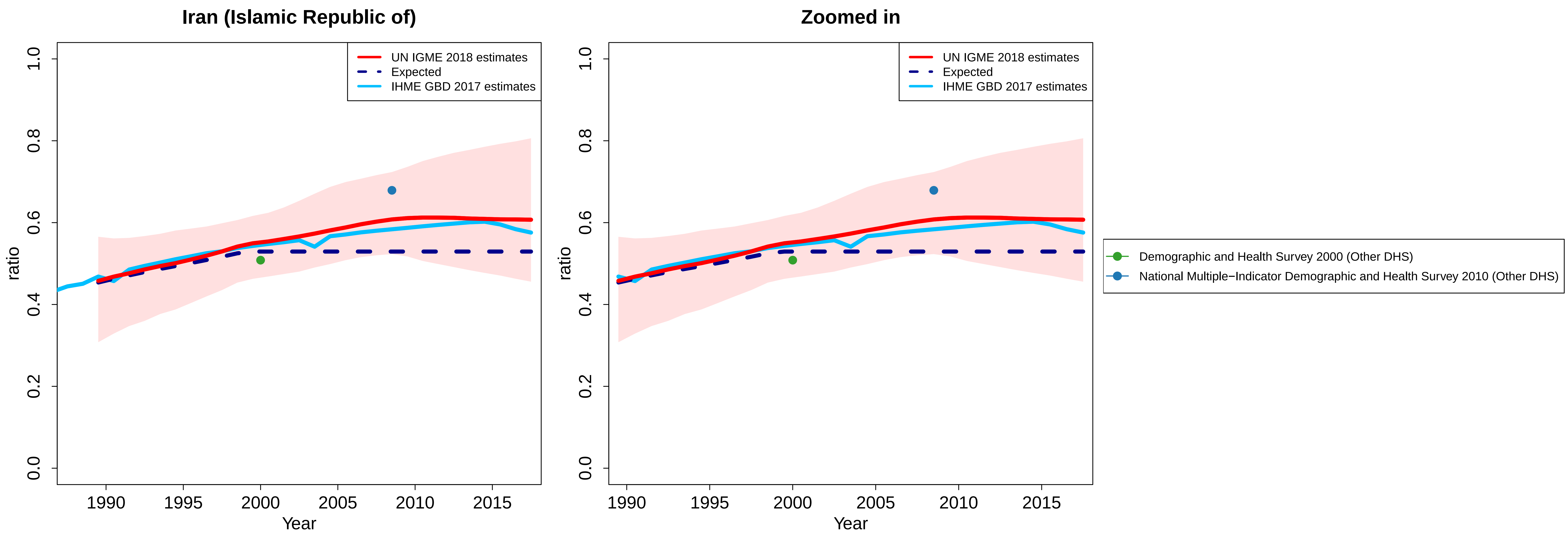

Iran (Islamic Republic of)
Zoomed in
UN IGME 2018 estimates
Expected
IHME GBD 2017 estimates
ratio
Year
0.0
0.2
0.4
0.6
0.8
1.0
1990
1995
2000
2005
2010
2015
Demographic and Health Survey 2000 (Other DHS)
National Multiple−Indicator Demographic and Health Survey 2010 (Other DHS)

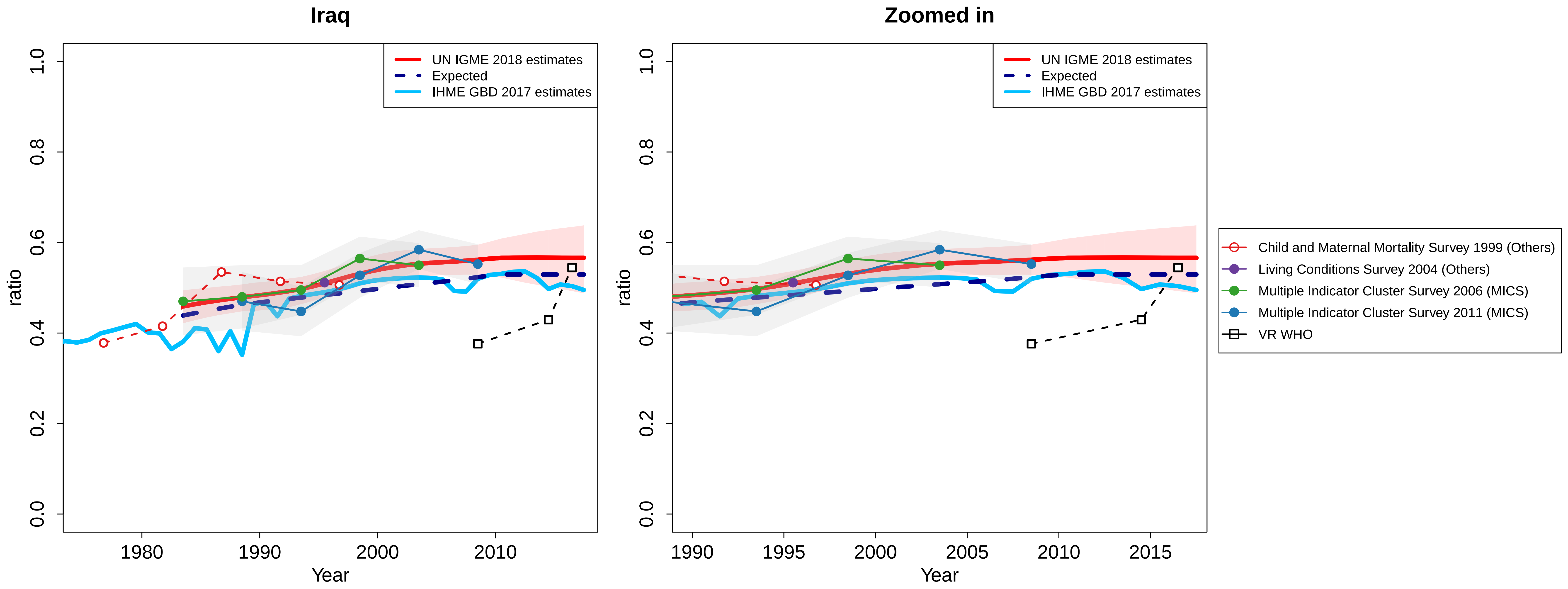
Iraq
Zoomed in
UN IGME 2018 estimates
Expected
IHME GBD 2017 estimates
ratio
Year
0.0
0.2
0.4
0.6
0.8
1.0
1980
1990
2000
2010
1995
2005
2015
Child and Maternal Mortality Survey 1999 (Others)
Living Conditions Survey 2004 (Others)
Multiple Indicator Cluster Survey 2006 (MICS)
Multiple Indicator Cluster Survey 2011 (MICS)
VR WHO

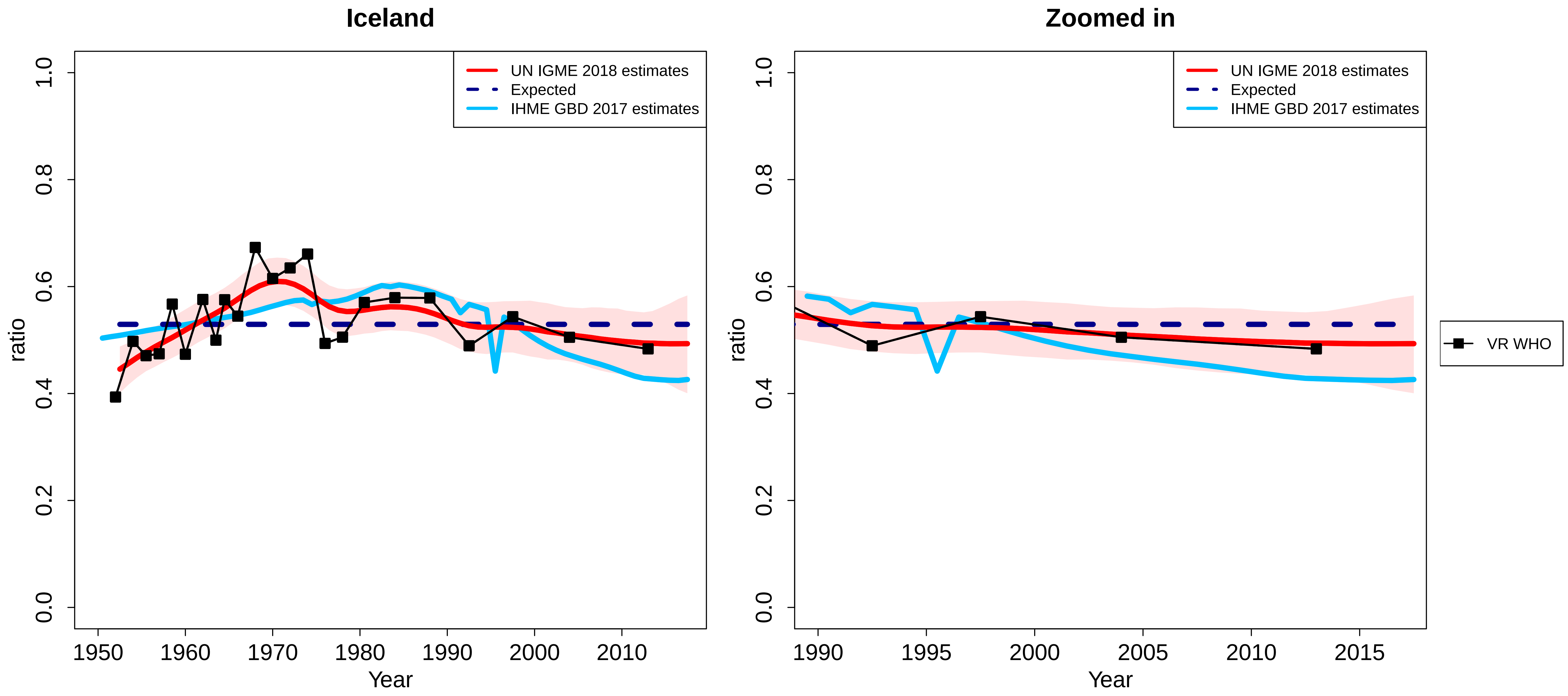
Iceland
Zoomed in
UN IGME 2018 estimates
Expected
IHME GBD 2017 estimates
VR WHO
ratio
Year
0.0
0.2
0.4
0.6
0.8
1.0
1950
1960
1970
1980
1990
2000
2010
1995
2005
2015

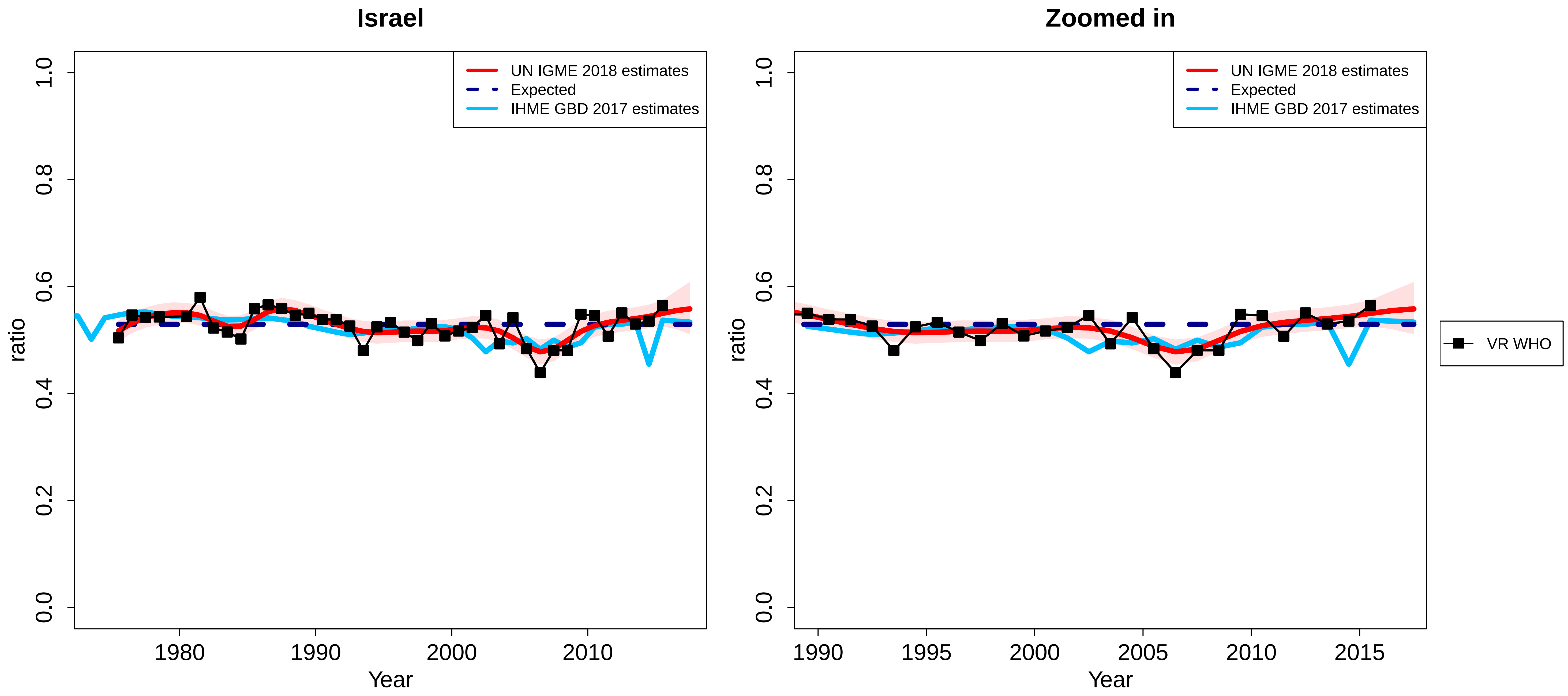
Israel
Zoomed in
UN IGME 2018 estimates
Expected
IHME GBD 2017 estimates
VR WHO
ratio
Year
0.0
0.2
0.4
0.6
0.8
1.0
1980
1990
2000
2010
1995
2005
2015

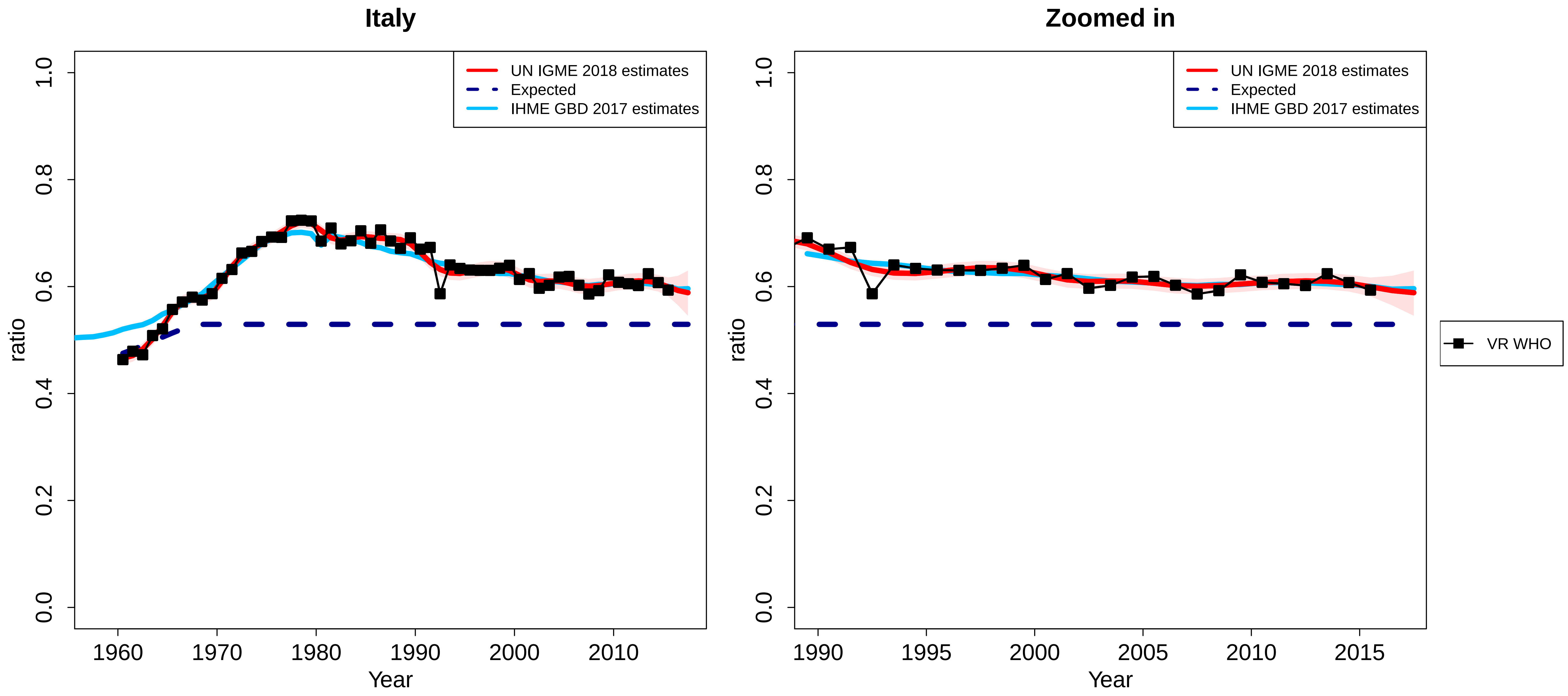
Italy
Zoomed in
UN IGME 2018 estimates
Expected
IHME GBD 2017 estimates
VR WHO
ratio
Year
0.0
0.2
0.4
0.6
0.8
1.0
1960
1970
1980
1990
2000
2010
1995
2005
2015

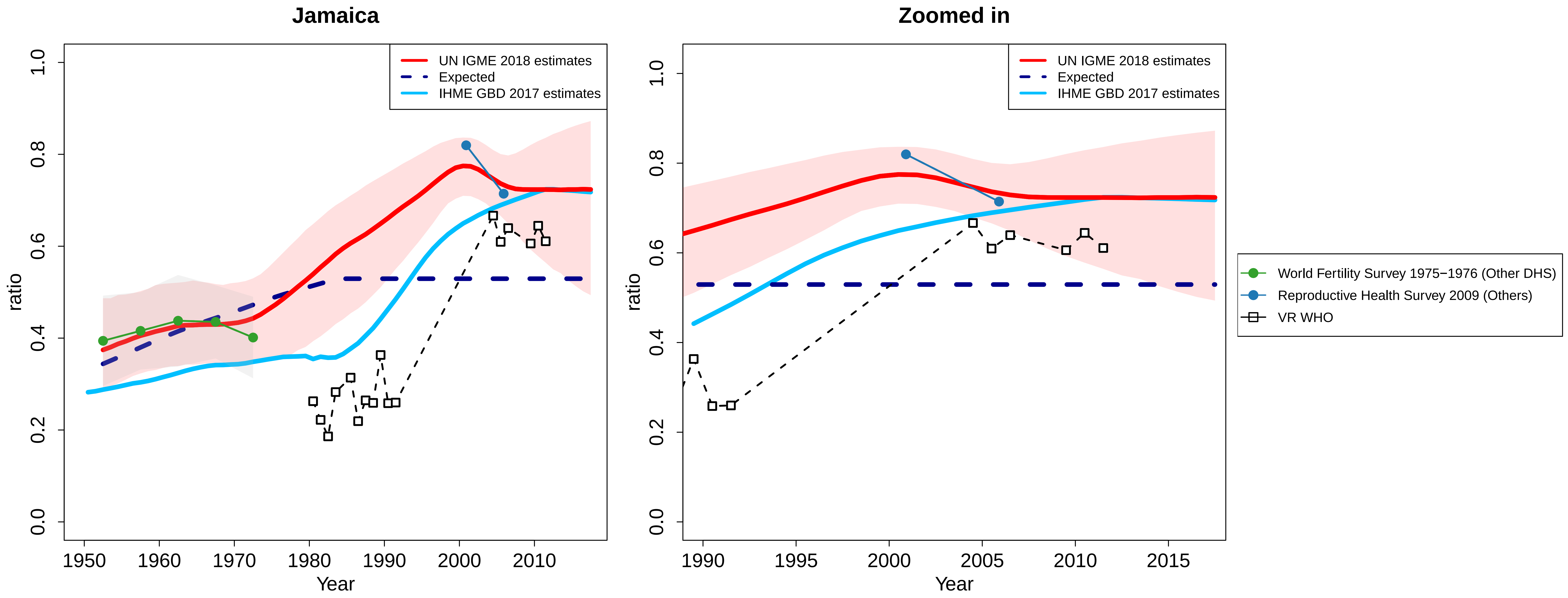

Jamaica
Zoomed in
UN IGME 2018 estimates
Expected
IHME GBD 2017 estimates
World Fertility Survey 1975−1976 (Other DHS)
Reproductive Health Survey 2009 (Others)
VR WHO
ratio
Year
0.0
0.2
0.4
0.6
0.8
1.0
1950
1960
1970
1980
1990
2000
2010
1995
2005
2015

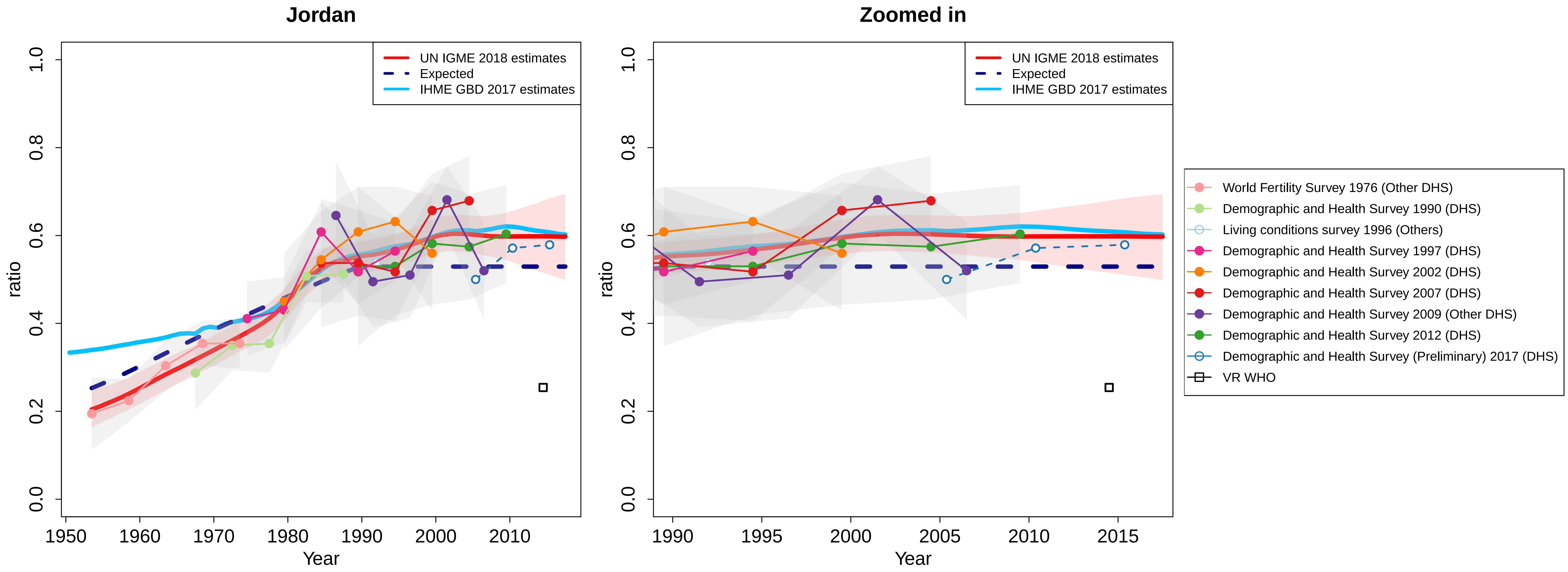

Jordan
Zoomed in
UN IGME 2018 estimates
Expected
IHME GBD 2017 estimates
ratio
Year
0.0
0.2
0.4
0.6
0.8
1.0
1950
1960
1970
1980
1990
2000
2010
1995
2005
2015
World Fertility Survey 1976 (Other DHS)
Demographic and Health Survey 1990 (DHS)
Living conditions survey 1996 (Others)
Demographic and Health Survey 1997 (DHS)
Demographic and Health Survey 2002 (DHS)
Demographic and Health Survey 2007 (DHS)
Demographic and Health Survey 2009 (Other DHS)
Demographic and Health Survey 2012 (DHS)
Demographic and Health Survey (Preliminary) 2017 (DHS)
VR WHO

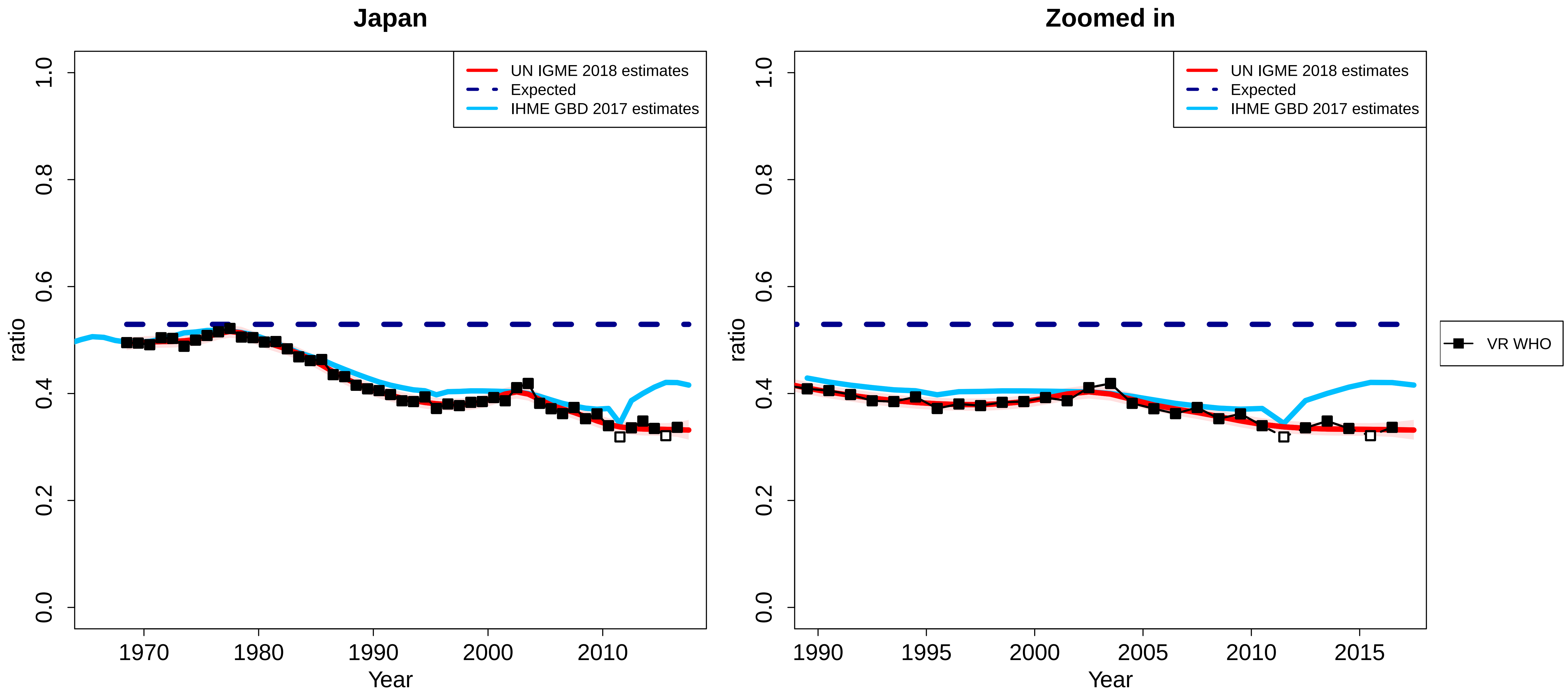
Japan
Zoomed in
UN IGME 2018 estimates
Expected
IHME GBD 2017 estimates
VR WHO
ratio
Year
0.0
0.2
0.4
0.6
0.8
1.0
1970
1980
1990
2000
2010
1995
2005
2015

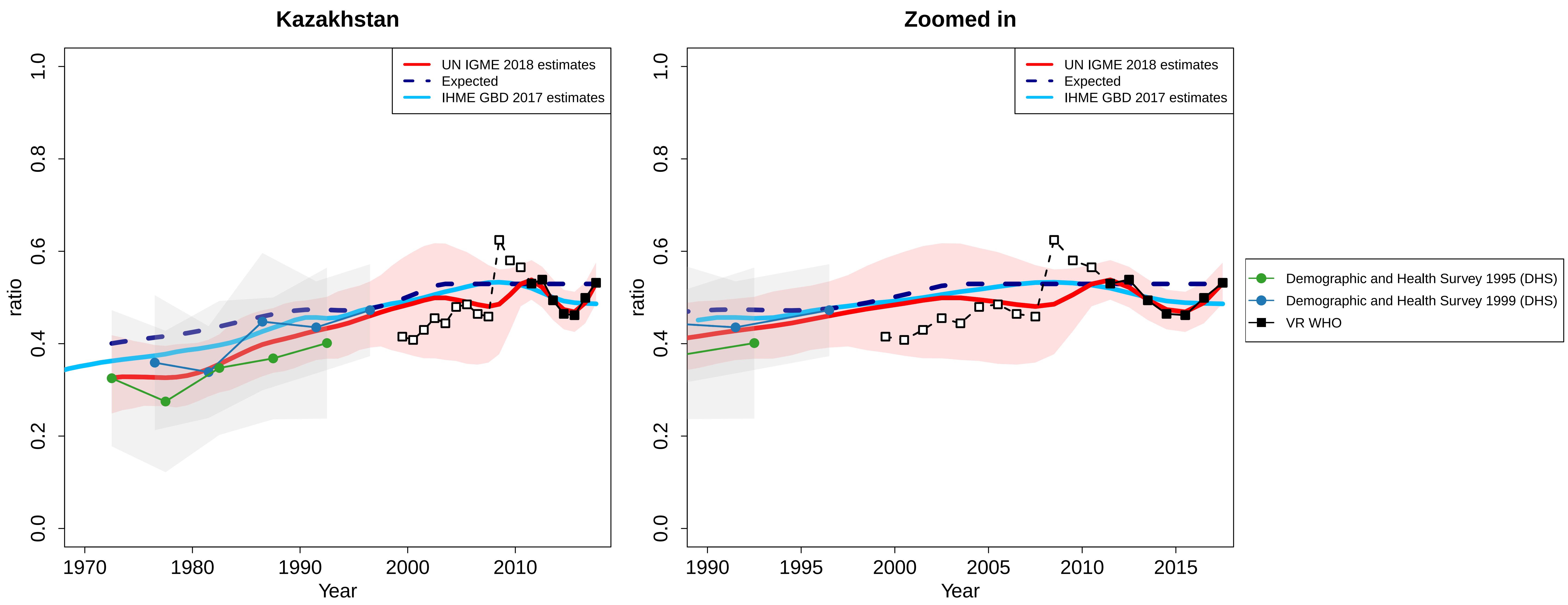

Kazakhstan
Zoomed in
UN IGME 2018 estimates
Expected
IHME GBD 2017 estimates
Demographic and Health Survey 1995 (DHS)
Demographic and Health Survey 1999 (DHS)
VR WHO
ratio
Year
0.0
0.2
0.4
0.6
0.8
1.0
1970
1980
1990
2000
2010
1995
2005
2015

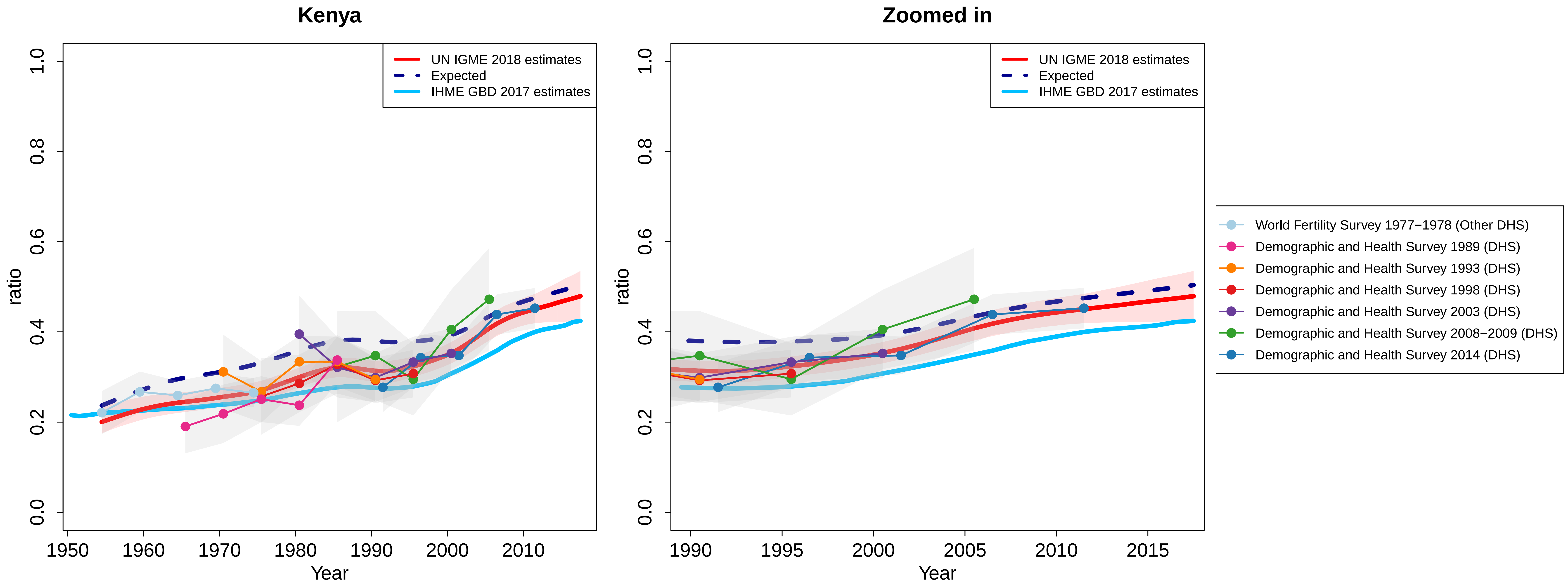
Kenya
Zoomed in
UN IGME 2018 estimates
Expected
IHME GBD 2017 estimates
ratio
Year
1950
1960
1970
1980
1990
2000
2010
1995
2005
2015
0.0
0.2
0.4
0.6
0.8
1.0
World Fertility Survey 1977−1978 (Other DHS)
Demographic and Health Survey 1989 (DHS)
Demographic and Health Survey 1993 (DHS)
Demographic and Health Survey 1998 (DHS)
Demographic and Health Survey 2003 (DHS)
Demographic and Health Survey 2008−2009 (DHS)
Demographic and Health Survey 2014 (DHS)

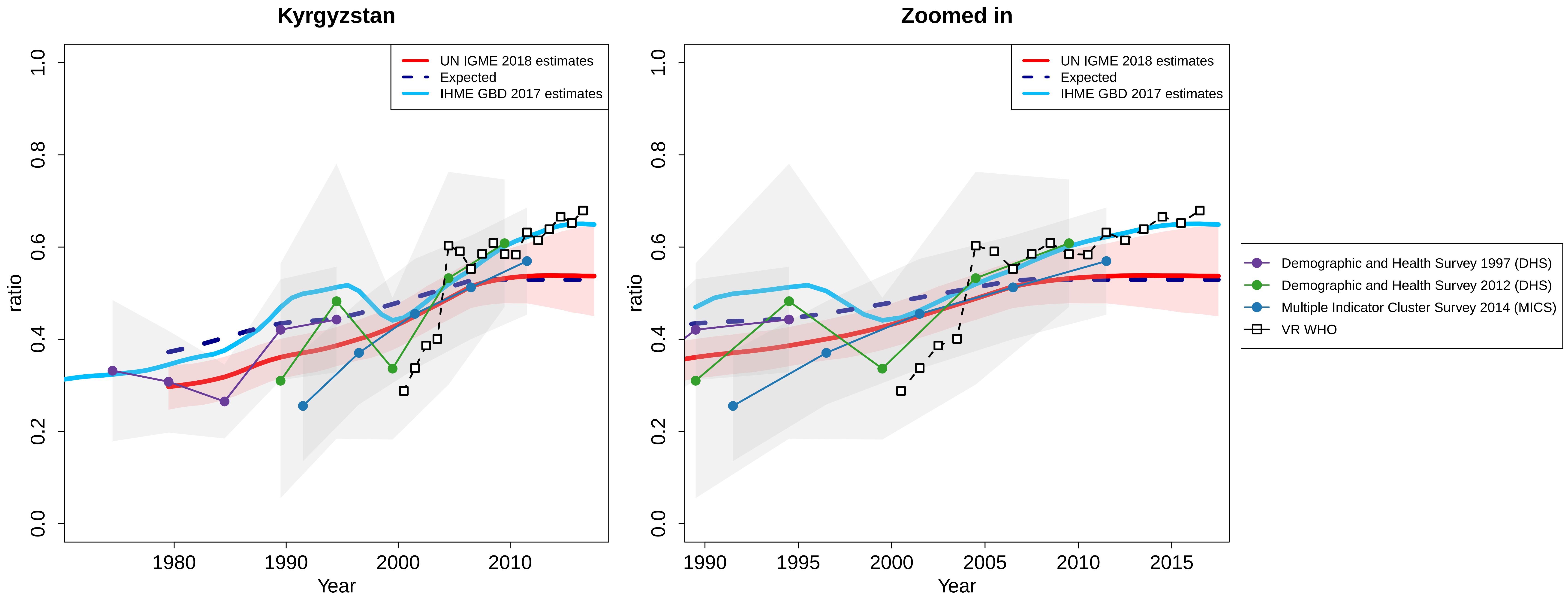

Kyrgyzstan
Zoomed in
UN IGME 2018 estimates
Expected
IHME GBD 2017 estimates
ratio
Year
1.0
0.8
0.6
0.4
0.2
0.0
1980
1990
2000
2010
1995
2005
2015
Demographic and Health Survey 1997 (DHS)
Demographic and Health Survey 2012 (DHS)
Multiple Indicator Cluster Survey 2014 (MICS)
VR WHO

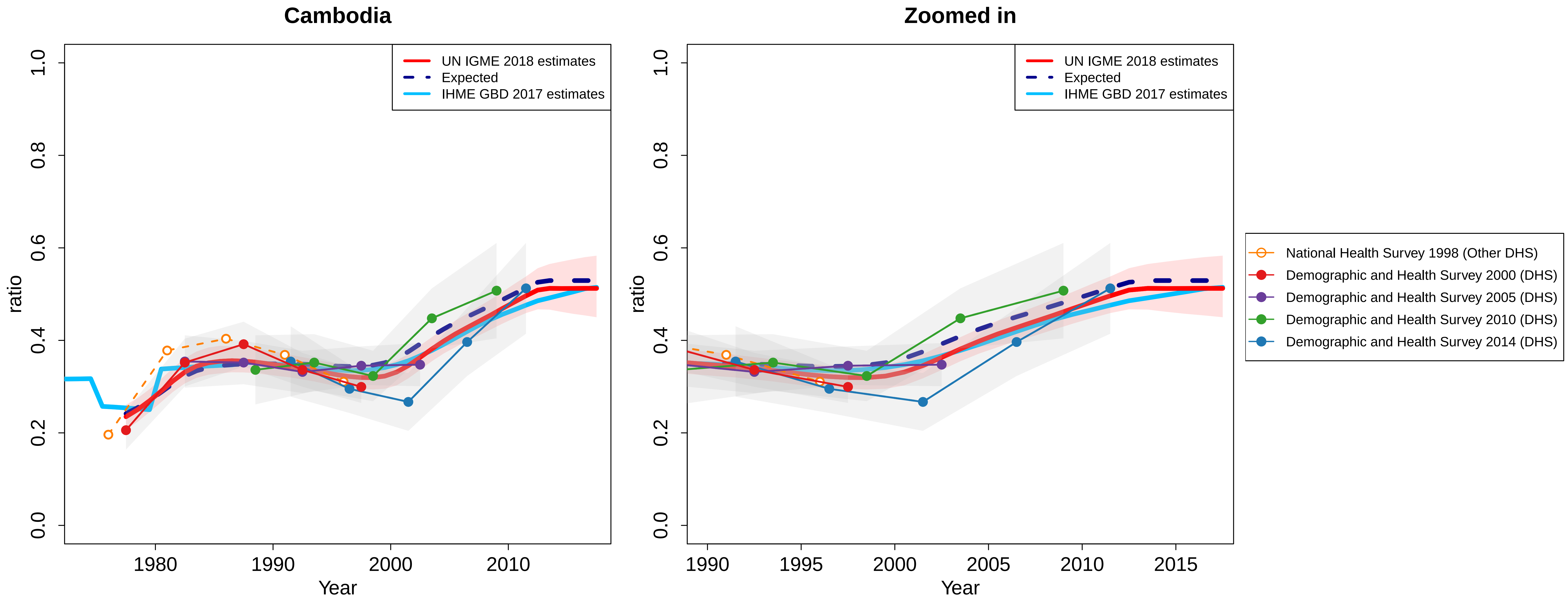

Cambodia
Zoomed in
UN IGME 2018 estimates
Expected
IHME GBD 2017 estimates
ratio
Year
0.0
0.2
0.4
0.6
0.8
1.0
1980
1990
2000
2010
1995
2005
2015
National Health Survey 1998 (Other DHS)
Demographic and Health Survey 2000 (DHS)
Demographic and Health Survey 2005 (DHS)
Demographic and Health Survey 2010 (DHS)
Demographic and Health Survey 2014 (DHS)

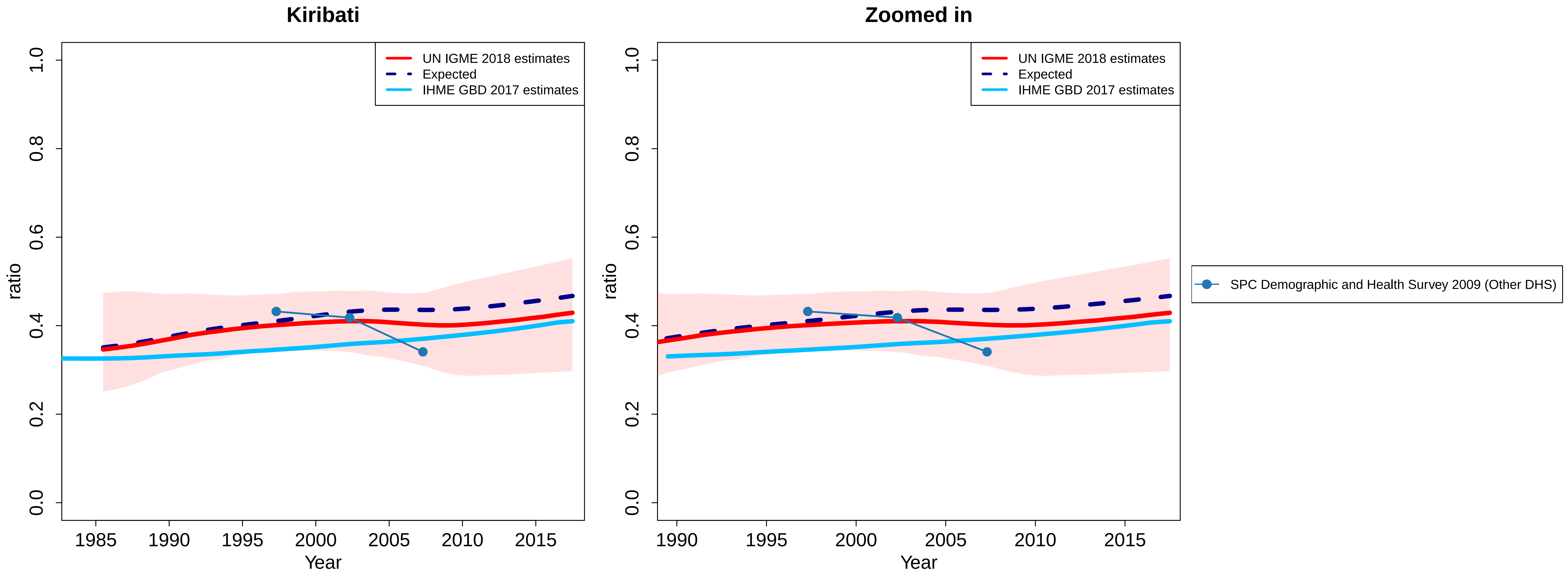
Kiribati
Zoomed in
UN IGME 2018 estimates
Expected
IHME GBD 2017 estimates
SPC Demographic and Health Survey 2009 (Other DHS)
ratio
Year
0.0
0.2
0.4
0.6
0.8
1.0
1985
1990
1995
2000
2005
2010
2015

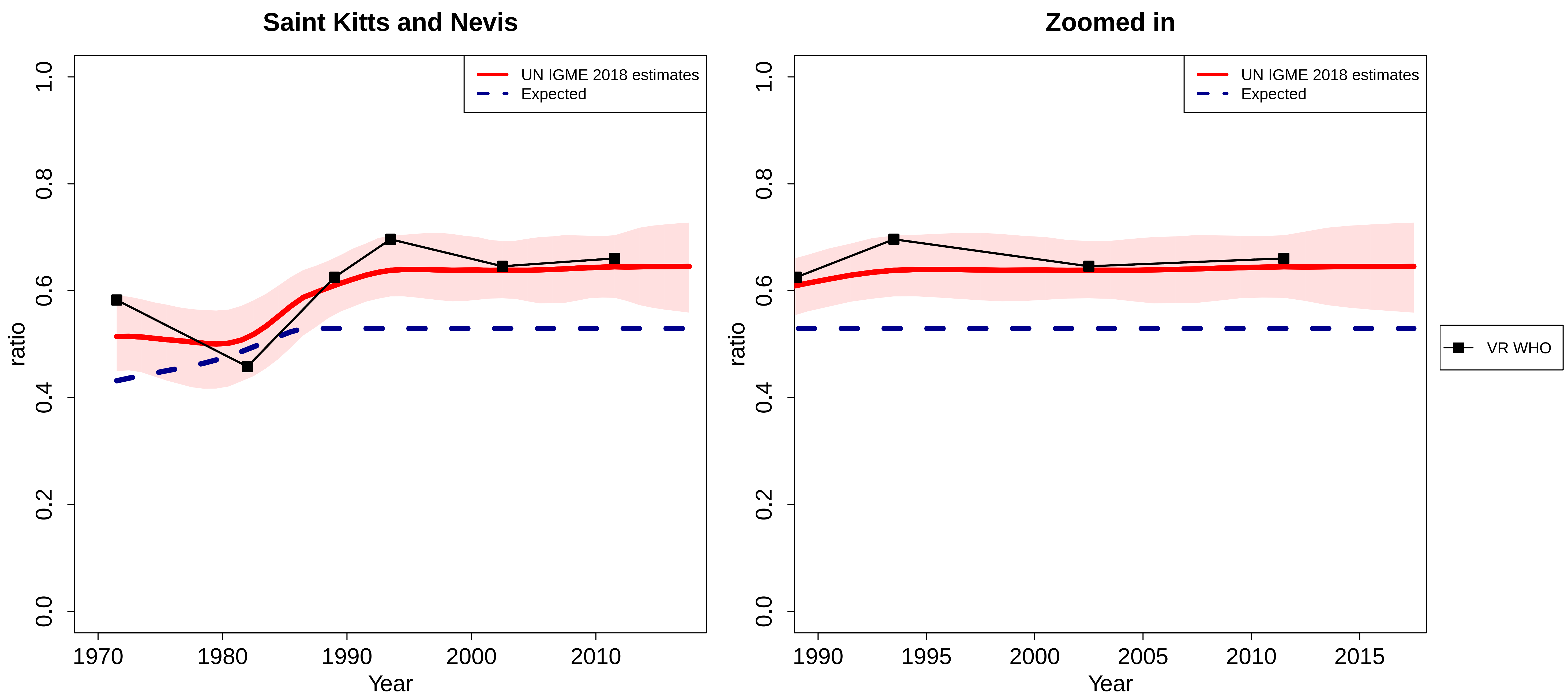

Saint Kitts and Nevis
Zoomed in
UN IGME 2018 estimates
Expected
VR WHO
ratio
Year
0.0
0.2
0.4
0.6
0.8
1.0
1970
1980
1990
2000
2010
1995
2005
2015

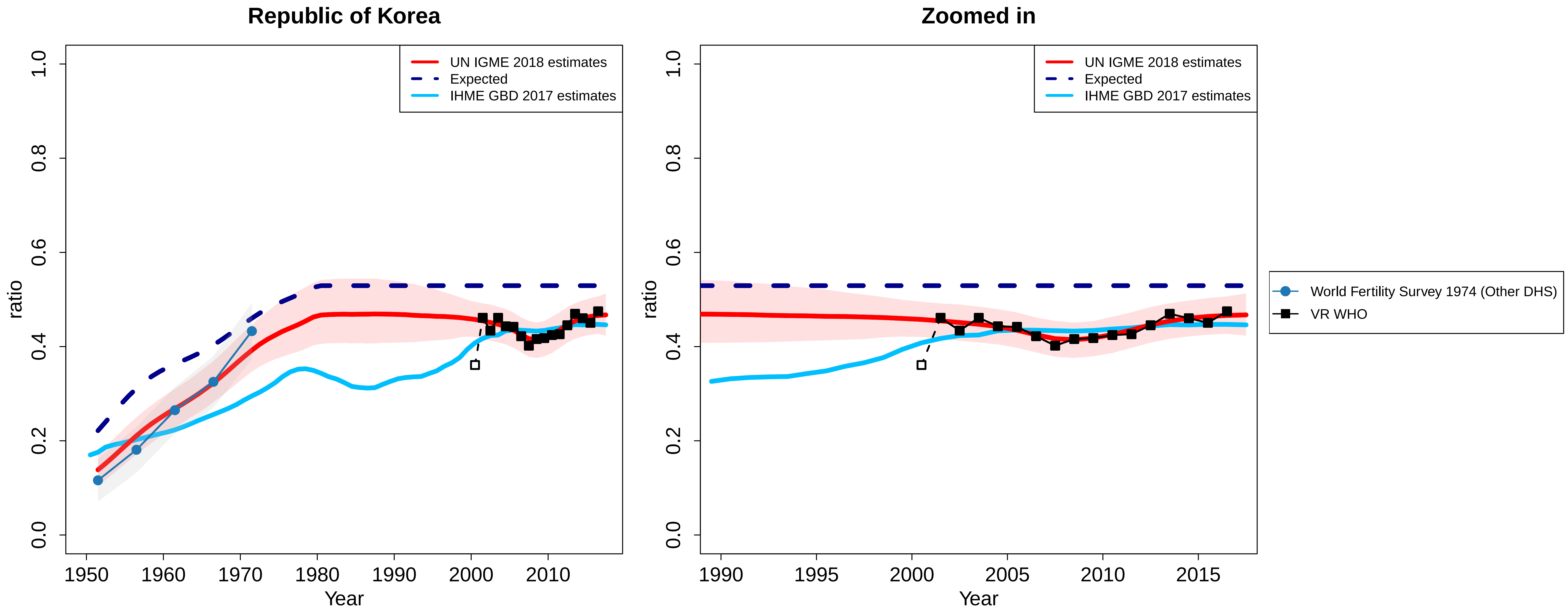

Republic of Korea
Zoomed in
UN IGME 2018 estimates
Expected
IHME GBD 2017 estimates
World Fertility Survey 1974 (Other DHS)
VR WHO
ratio
Year
0.0
0.2
0.4
0.6
0.8
1.0
1950
1960
1970
1980
1990
2000
2010
1990
1995
2000
2005
2010
2015

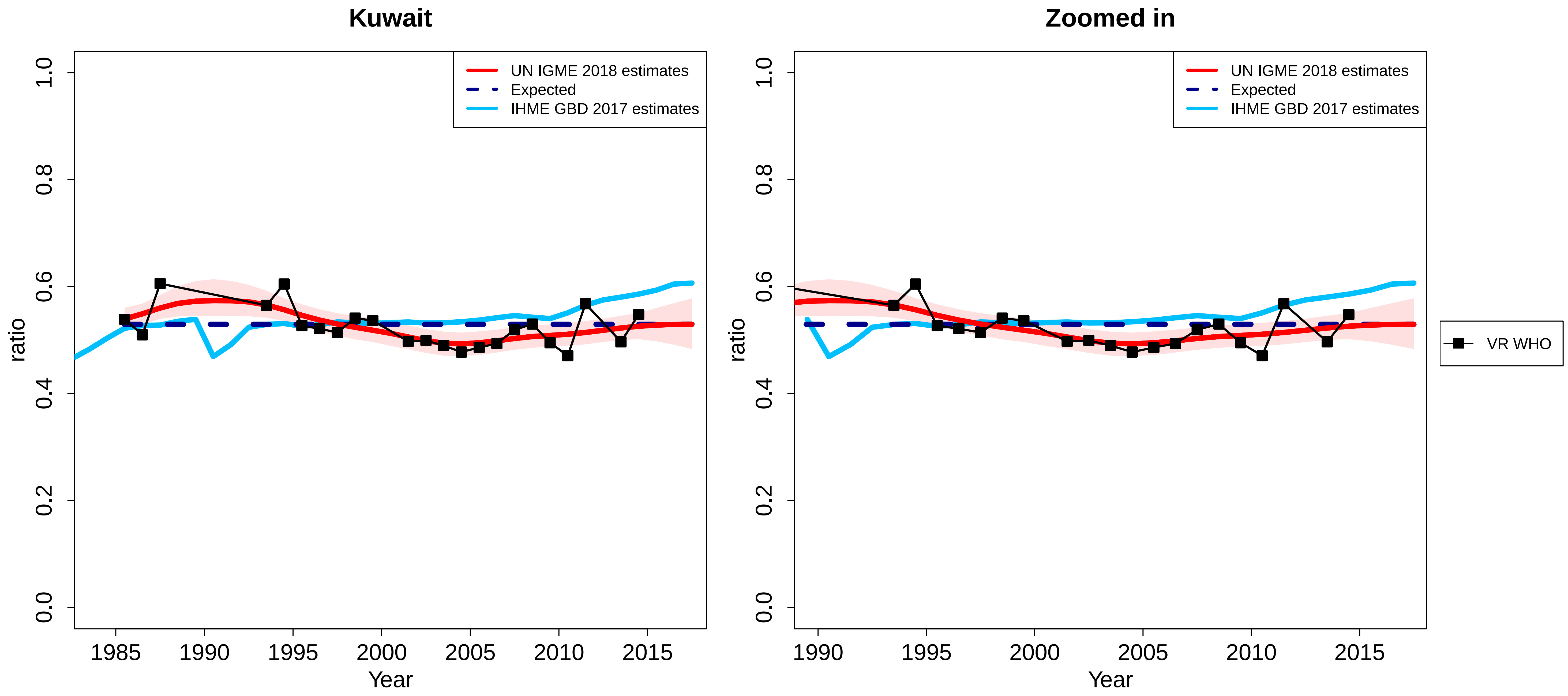

Kuwait
Zoomed in
UN IGME 2018 estimates
Expected
IHME GBD 2017 estimates
VR WHO
ratio
Year
0.0
0.2
0.4
0.6
0.8
1.0
1985
1990
1995
2000
2005
2010
2015

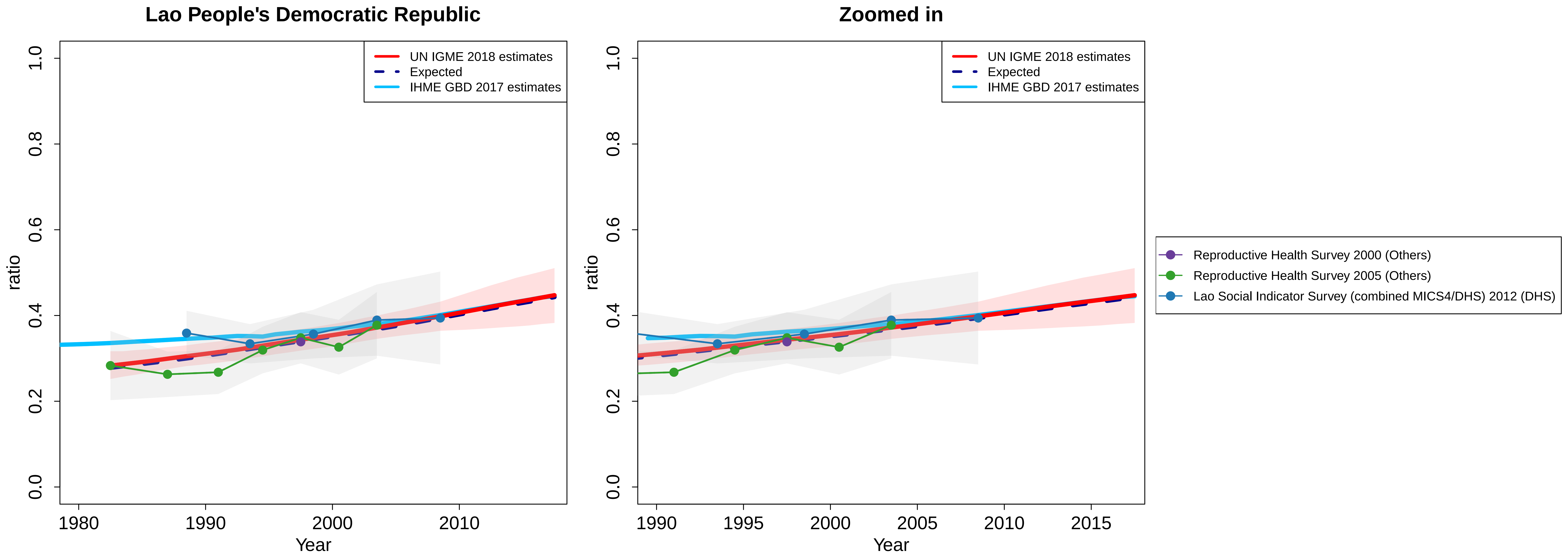

Lao People's Democratic Republic
Zoomed in
UN IGME 2018 estimates
Expected
IHME GBD 2017 estimates
ratio
Year
Reproductive Health Survey 2000 (Others)
Reproductive Health Survey 2005 (Others)
Lao Social Indicator Survey (combined MICS4/DHS) 2012 (DHS)

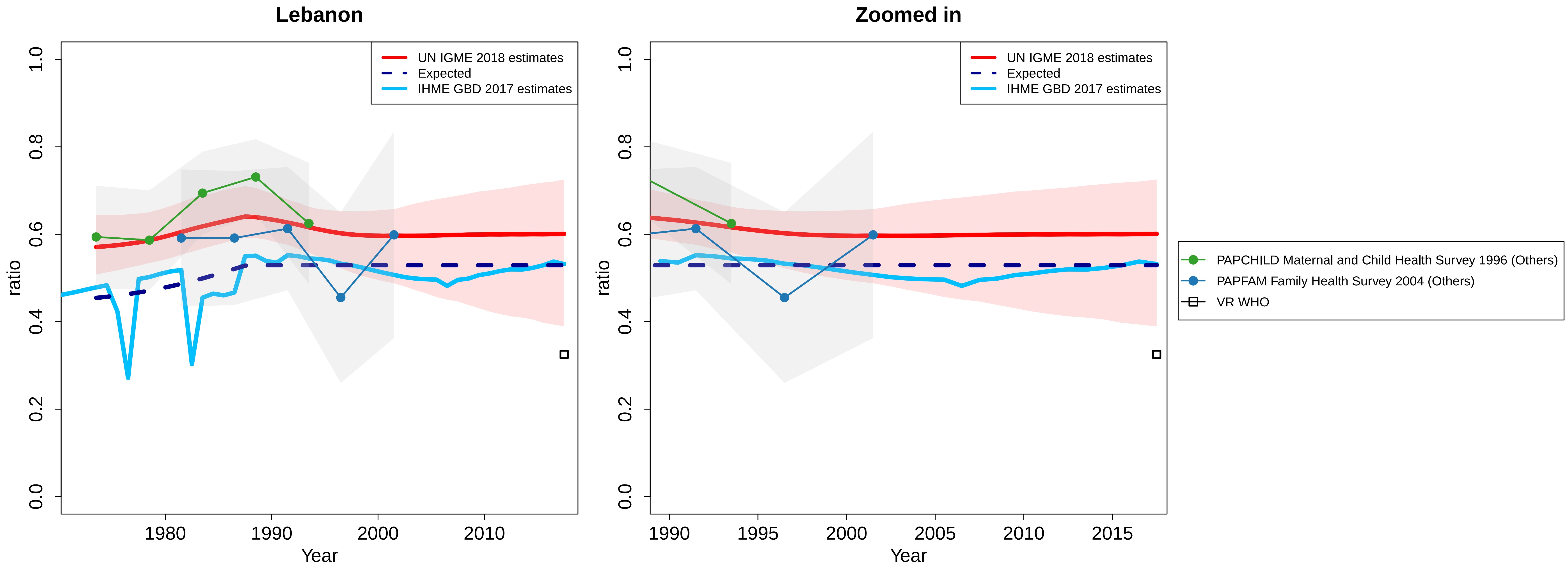

Lebanon
Zoomed in
UN IGME 2018 estimates
Expected
IHME GBD 2017 estimates
ratio
Year
1.0
0.8
0.6
0.4
0.2
0.0
1980
1990
2000
2010
1995
2005
2015
PAPCHILD Maternal and Child Health Survey 1996 (Others)
PAPFAM Family Health Survey 2004 (Others)
VR WHO

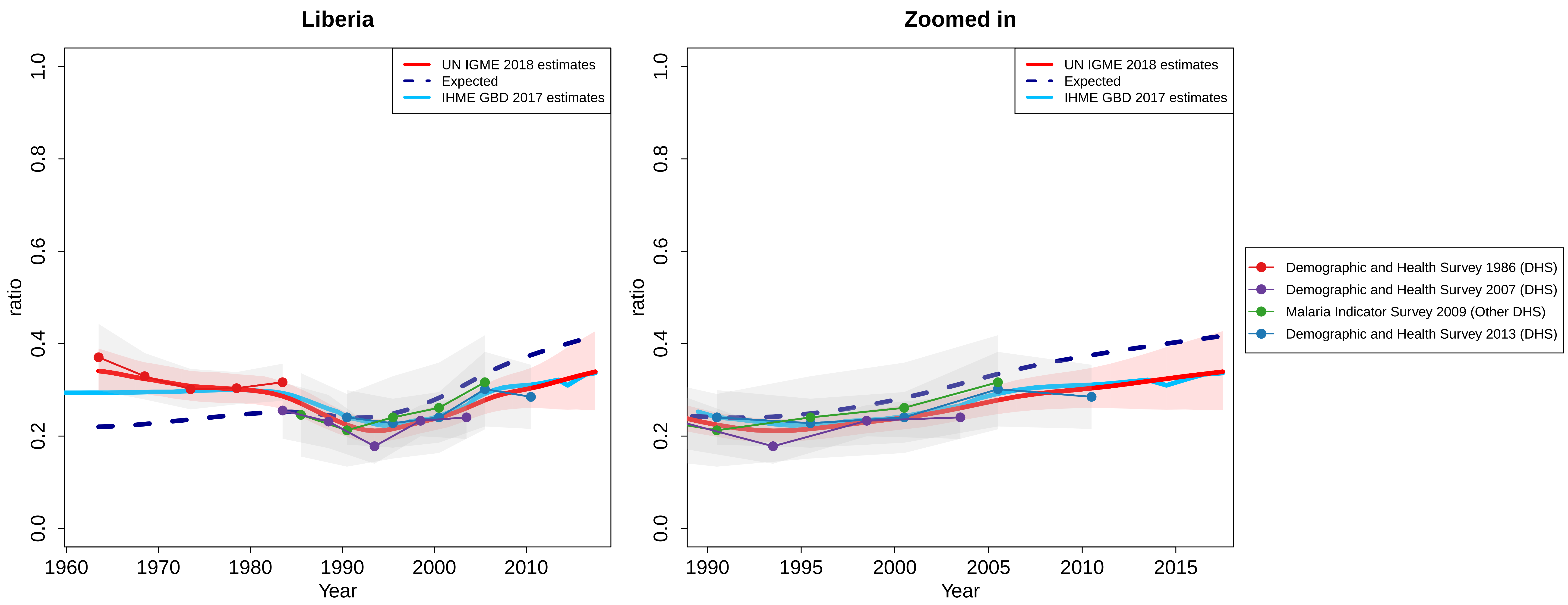
Liberia
Zoomed in
UN IGME 2018 estimates
Expected
IHME GBD 2017 estimates
ratio
Year
0.0
0.2
0.4
0.6
0.8
1.0
1960
1970
1980
1990
2000
2010
1995
2005
2015
Demographic and Health Survey 1986 (DHS)
Demographic and Health Survey 2007 (DHS)
Malaria Indicator Survey 2009 (Other DHS)
Demographic and Health Survey 2013 (DHS)

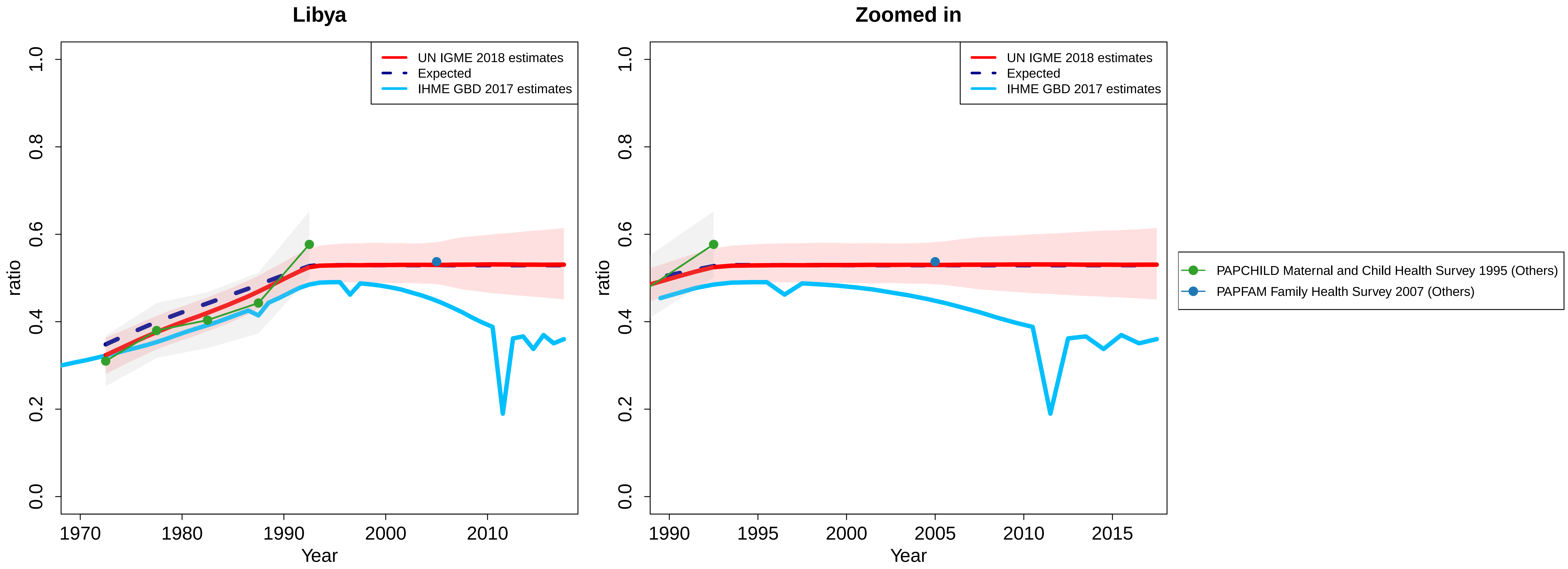

Libya
Zoomed in
UN IGME 2018 estimates
Expected
IHME GBD 2017 estimates
PAPCHILD Maternal and Child Health Survey 1995 (Others)
PAPFAM Family Health Survey 2007 (Others)
ratio
Year
0.0
0.2
0.4
0.6
0.8
1.0
1970
1980
1990
2000
2010
1995
2005
2015

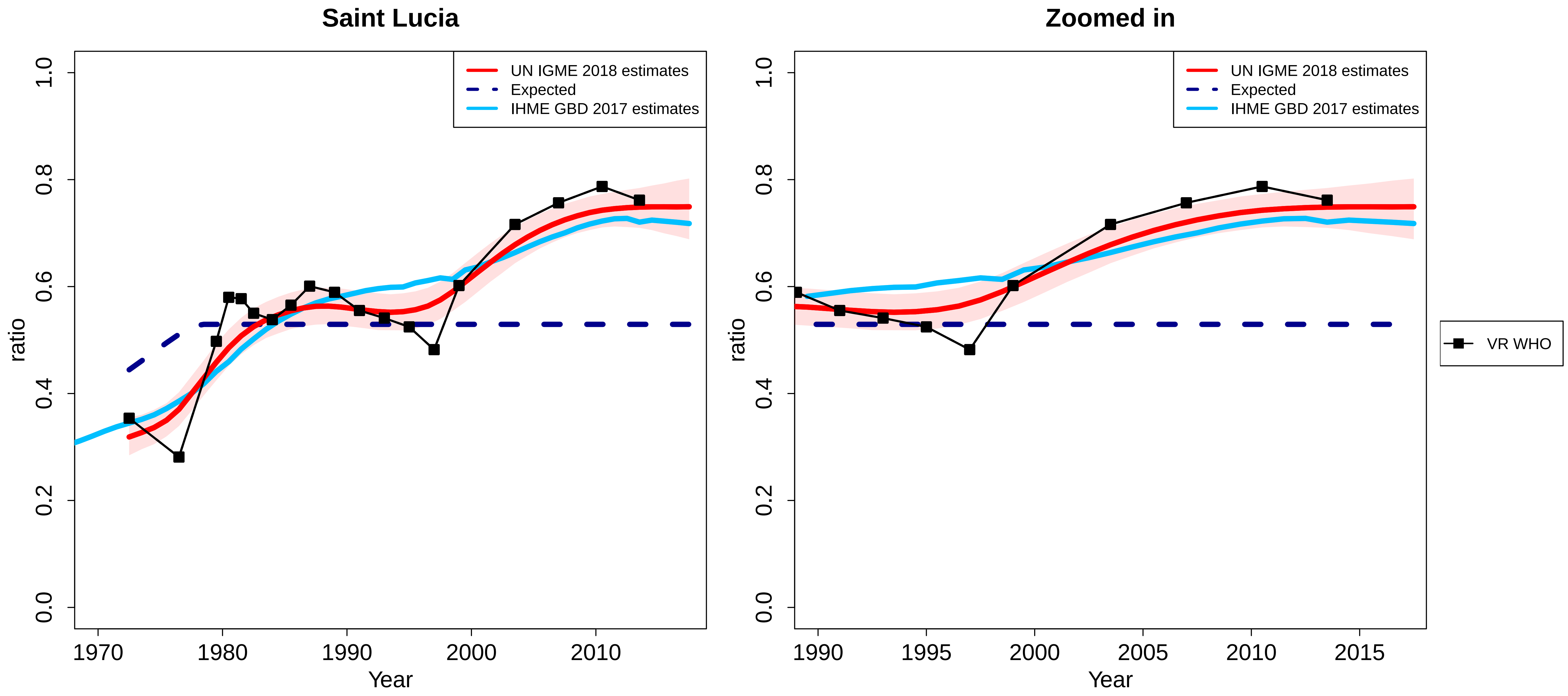

Saint Lucia
Zoomed in
UN IGME 2018 estimates
Expected
IHME GBD 2017 estimates
VR WHO
ratio
Year
0.0
0.2
0.4
0.6
0.8
1.0
1970
1980
1990
2000
2010
1995
2005
2015

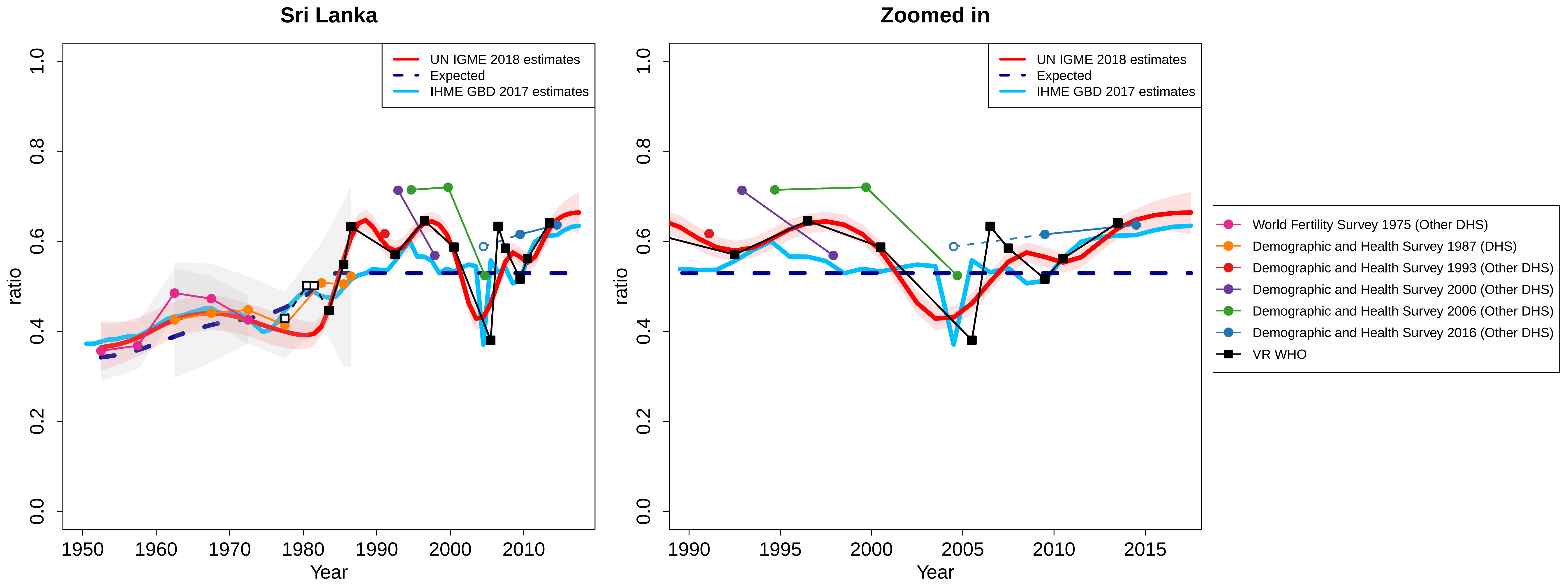

Sri Lanka
Zoomed in
UN IGME 2018 estimates
Expected
IHME GBD 2017 estimates
ratio
Year
0.0
0.2
0.4
0.6
0.8
1.0
1950
1960
1970
1980
1990
2000
2010
1995
2005
2015
World Fertility Survey 1975 (Other DHS)
Demographic and Health Survey 1987 (DHS)
Demographic and Health Survey 1993 (Other DHS)
Demographic and Health Survey 2000 (Other DHS)
Demographic and Health Survey 2006 (Other DHS)
Demographic and Health Survey 2016 (Other DHS)
VR WHO

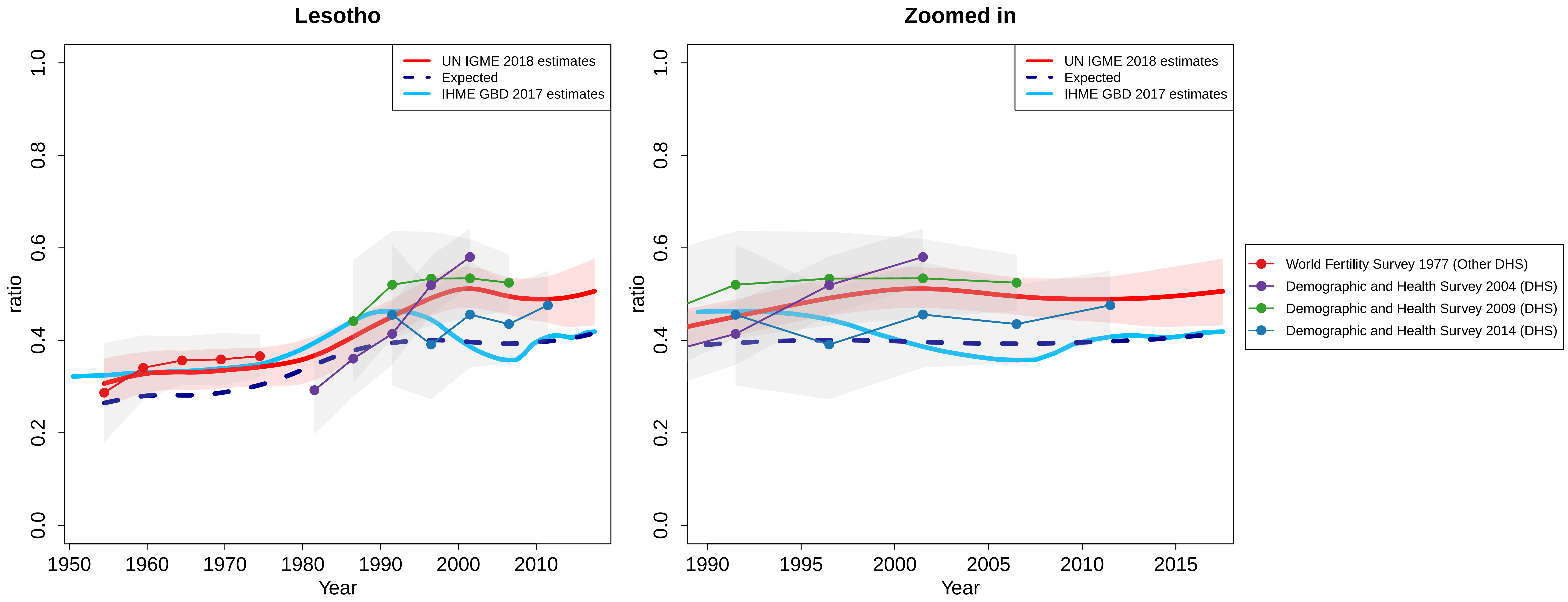
Lesotho
Zoomed in
UN IGME 2018 estimates
Expected
IHME GBD 2017 estimates
ratio
Year
World Fertility Survey 1977 (Other DHS)
Demographic and Health Survey 2004 (DHS)
Demographic and Health Survey 2009 (DHS)
Demographic and Health Survey 2014 (DHS)

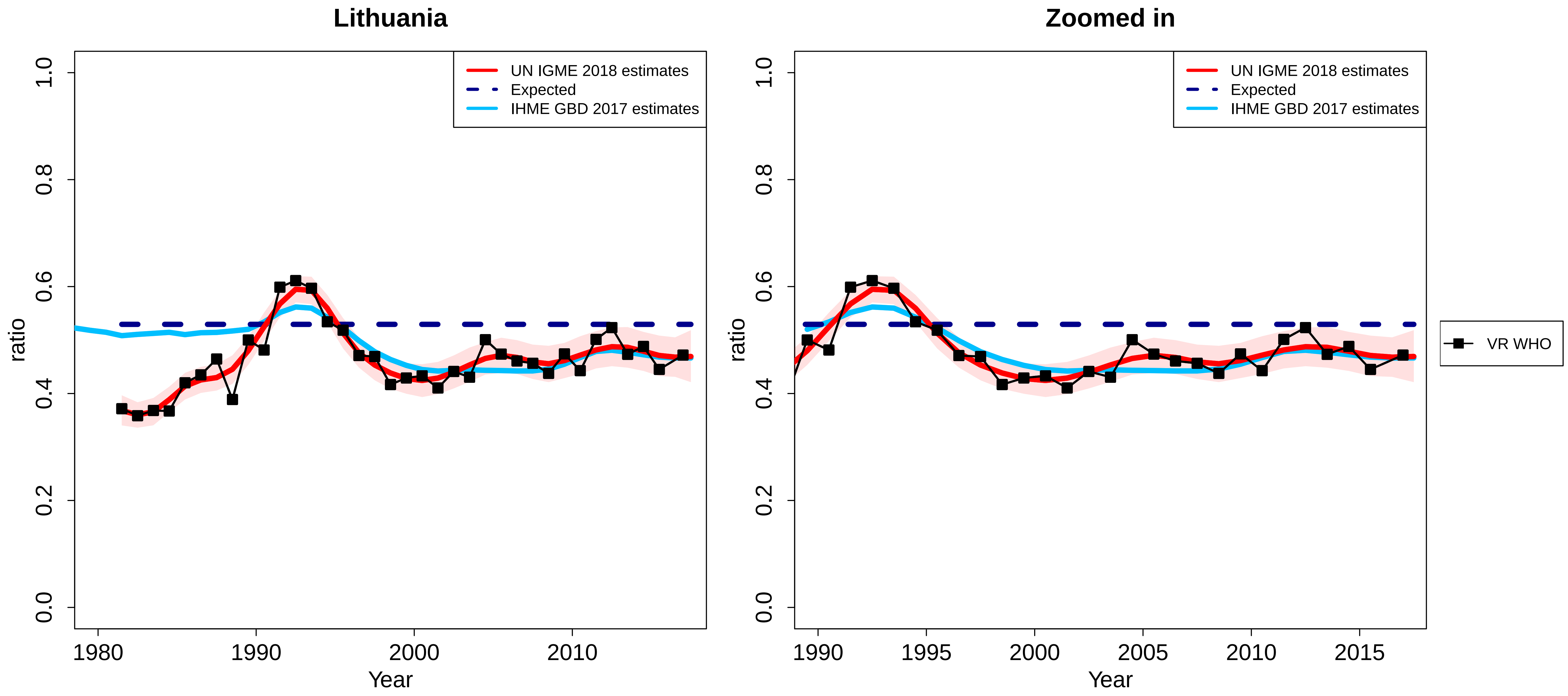

Lithuania
Zoomed in
UN IGME 2018 estimates
Expected
IHME GBD 2017 estimates
VR WHO
ratio
Year
0.0
0.2
0.4
0.6
0.8
1.0
1980
1990
2000
2010
1995
2005
2015

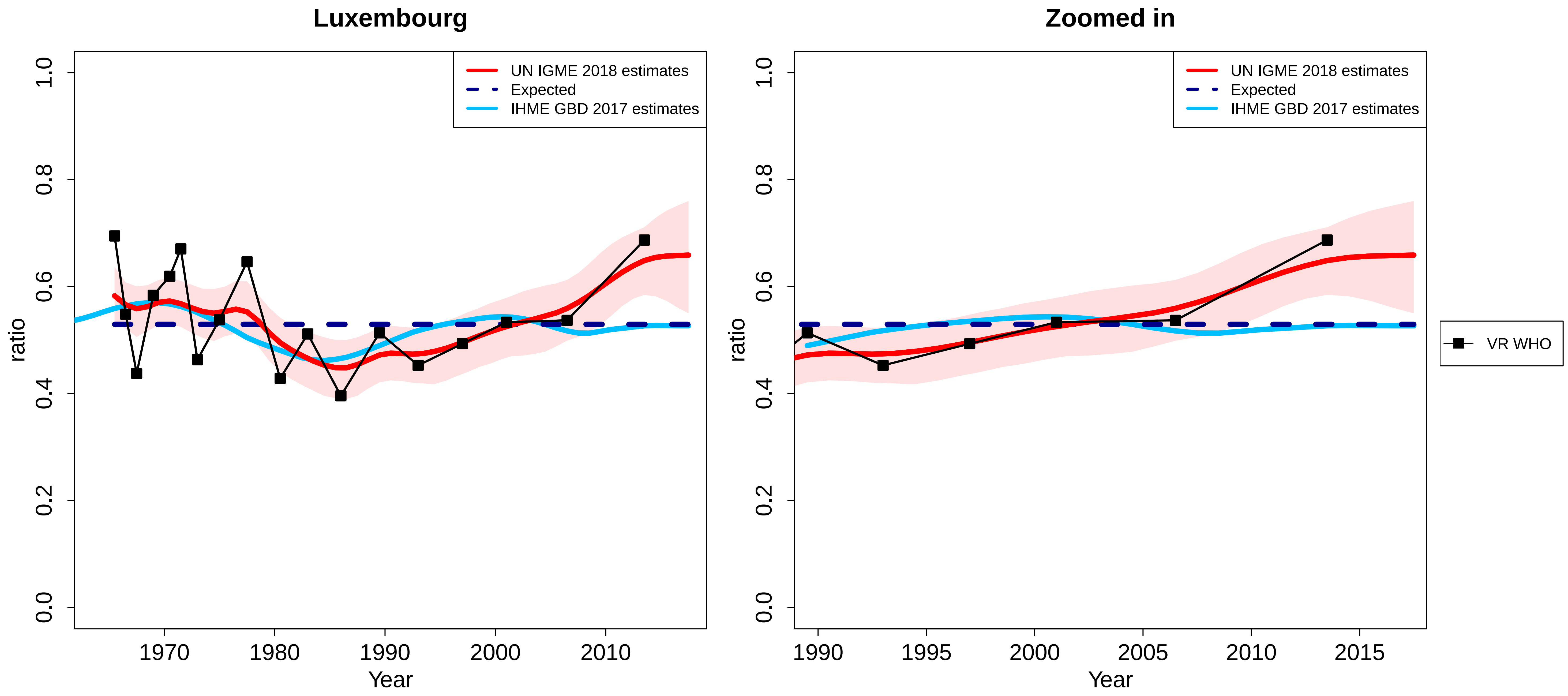

Luxembourg
Zoomed in
UN IGME 2018 estimates
Expected
IHME GBD 2017 estimates
VR WHO
ratio
Year
0.0
0.2
0.4
0.6
0.8
1.0
1970
1980
1990
2000
2010
1995
2005
2015

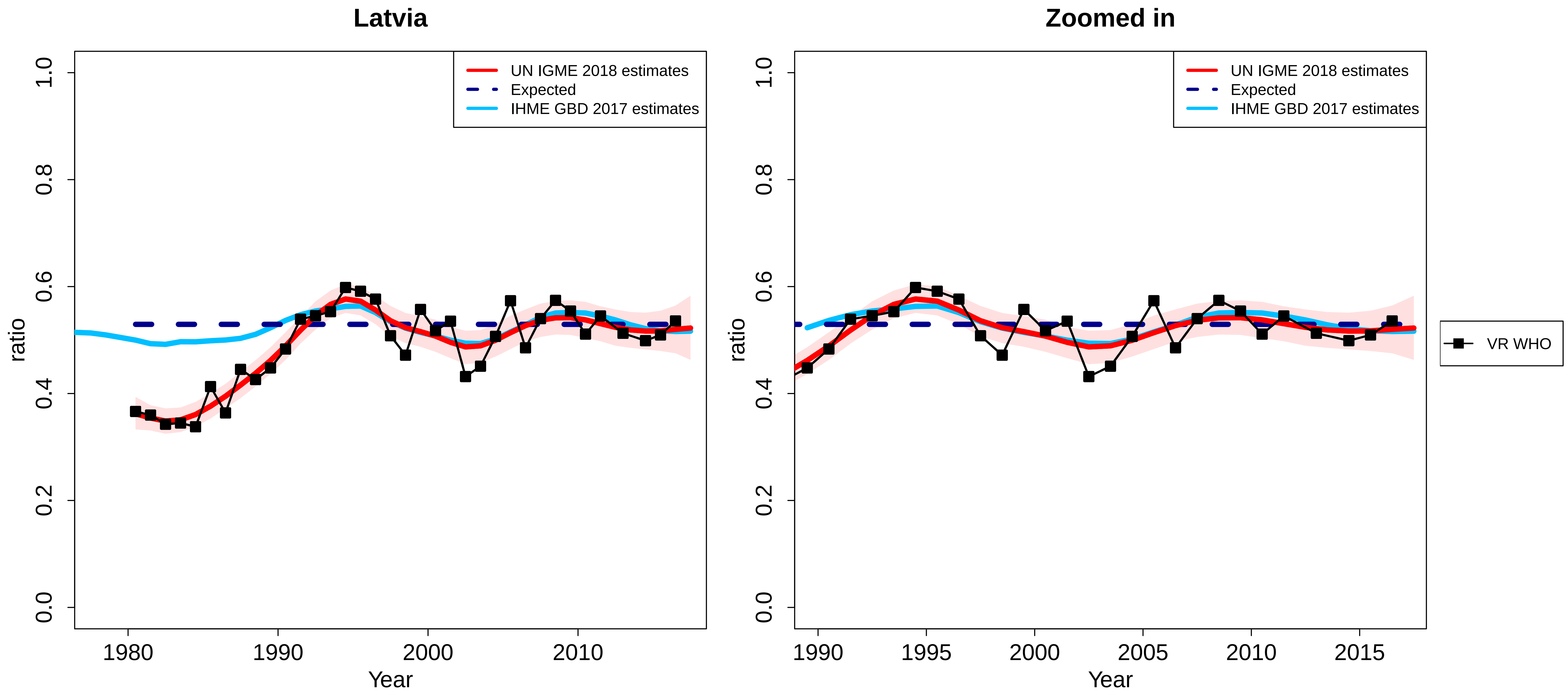

Latvia
Zoomed in
UN IGME 2018 estimates
Expected
IHME GBD 2017 estimates
VR WHO
ratio
Year
0.0
0.2
0.4
0.6
0.8
1.0
1980
1990
2000
2010
1995
2005
2015

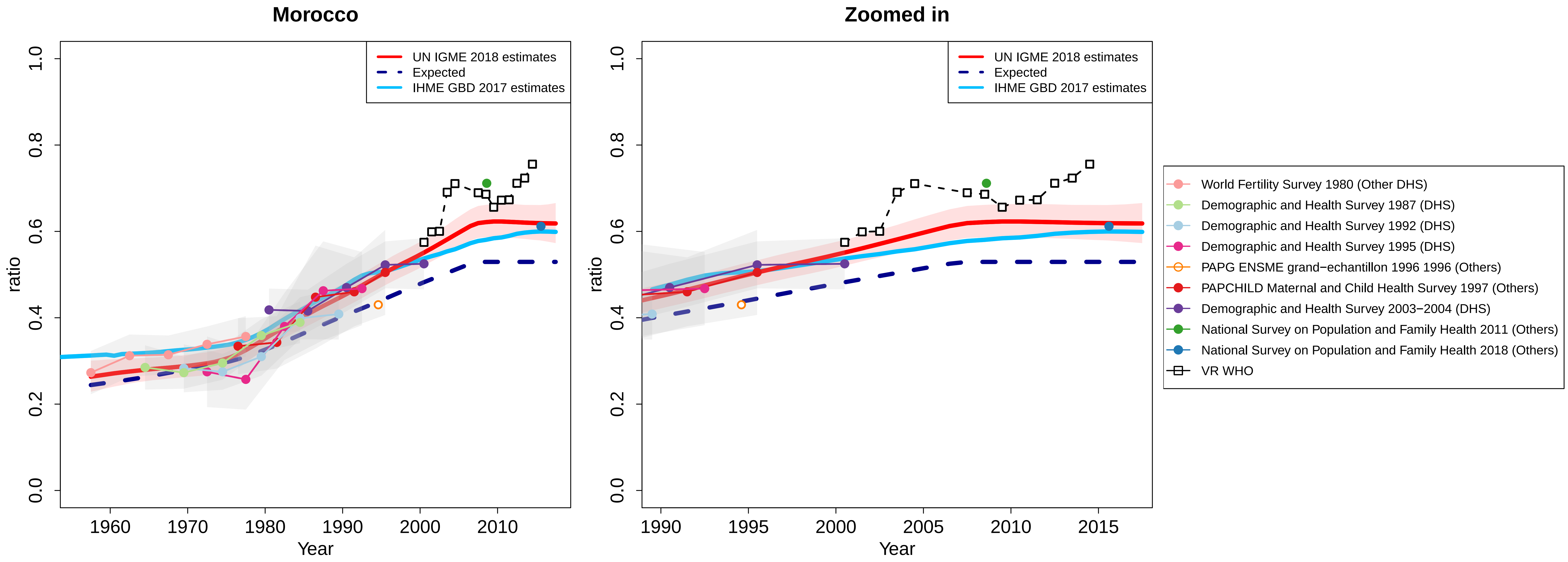
Morocco
Zoomed in
UN IGME 2018 estimates
Expected
IHME GBD 2017 estimates
ratio
Year
0.0
0.2
0.4
0.6
0.8
1.0
1960
1970
1980
1990
2000
2010
1990
1995
2000
2005
2010
2015
World Fertility Survey 1980 (Other DHS)
Demographic and Health Survey 1987 (DHS)
Demographic and Health Survey 1992 (DHS)
Demographic and Health Survey 1995 (DHS)
PAPG ENSME grand−echantillon 1996 1996 (Others)
PAPCHILD Maternal and Child Health Survey 1997 (Others)
Demographic and Health Survey 2003−2004 (DHS)
National Survey on Population and Family Health 2011 (Others)
National Survey on Population and Family Health 2018 (Others)
VR WHO

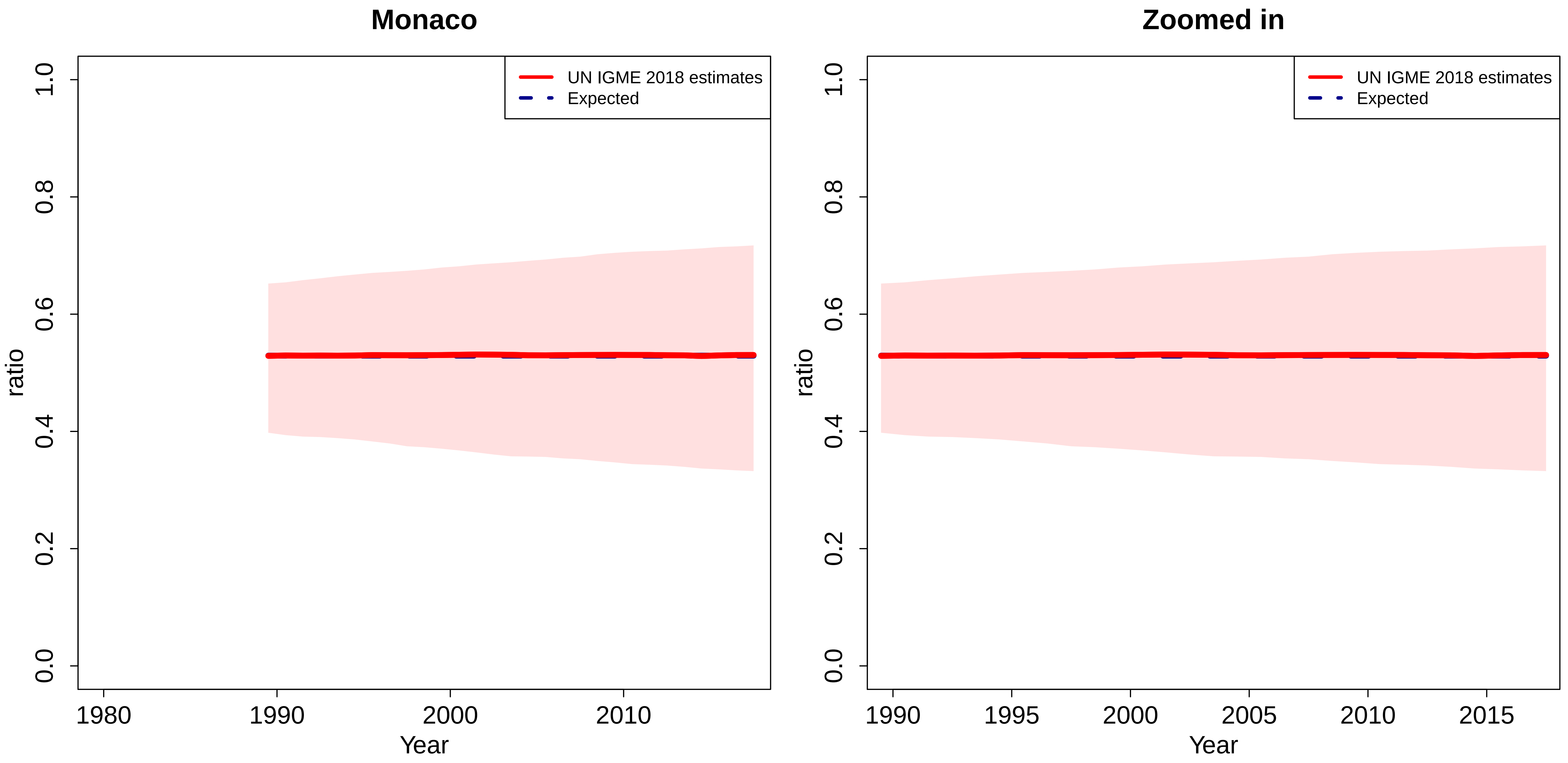

Monaco
Zoomed in
UN IGME 2018 estimates
Expected
ratio
Year
0.0
0.2
0.4
0.6
0.8
1.0
1980
1990
2000
2010
1995
2005
2015

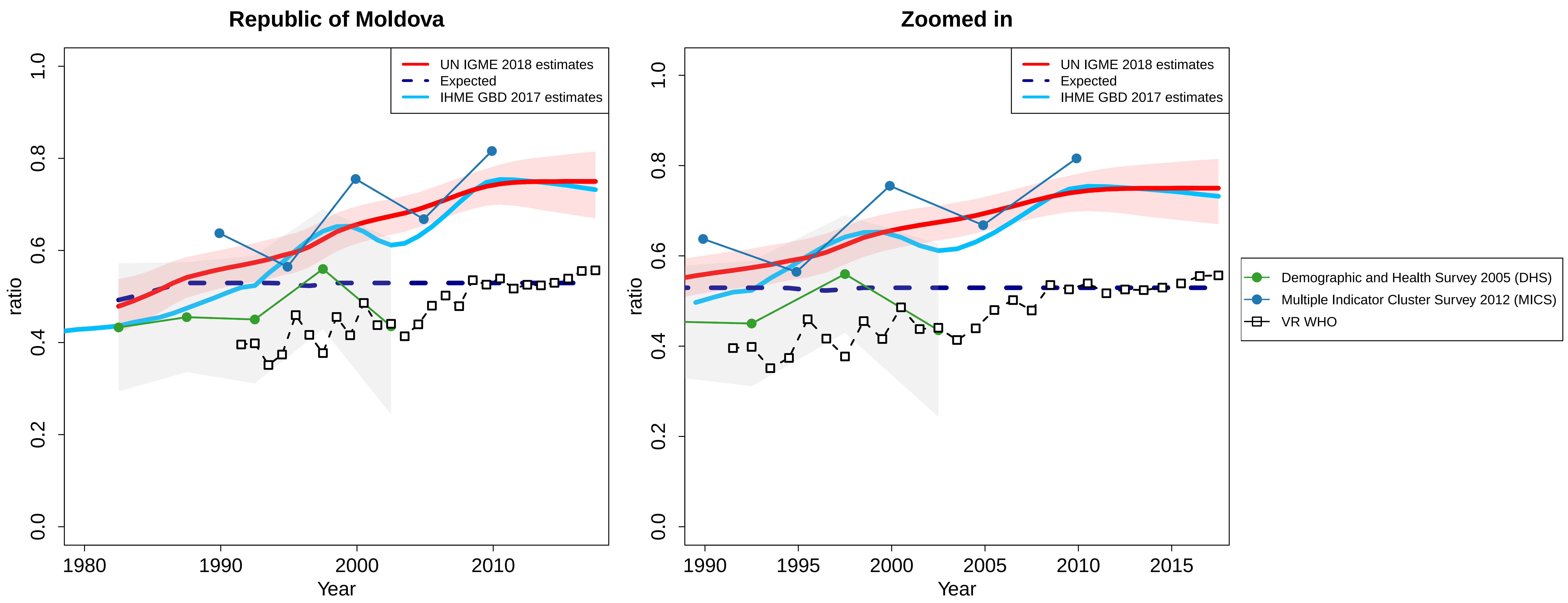

Republic of Moldova
Zoomed in
UN IGME 2018 estimates
Expected
IHME GBD 2017 estimates
ratio
Year
0.0
0.2
0.4
0.6
0.8
1.0
1980
1990
2000
2010
1995
2005
2015
Demographic and Health Survey 2005 (DHS)
Multiple Indicator Cluster Survey 2012 (MICS)
VR WHO

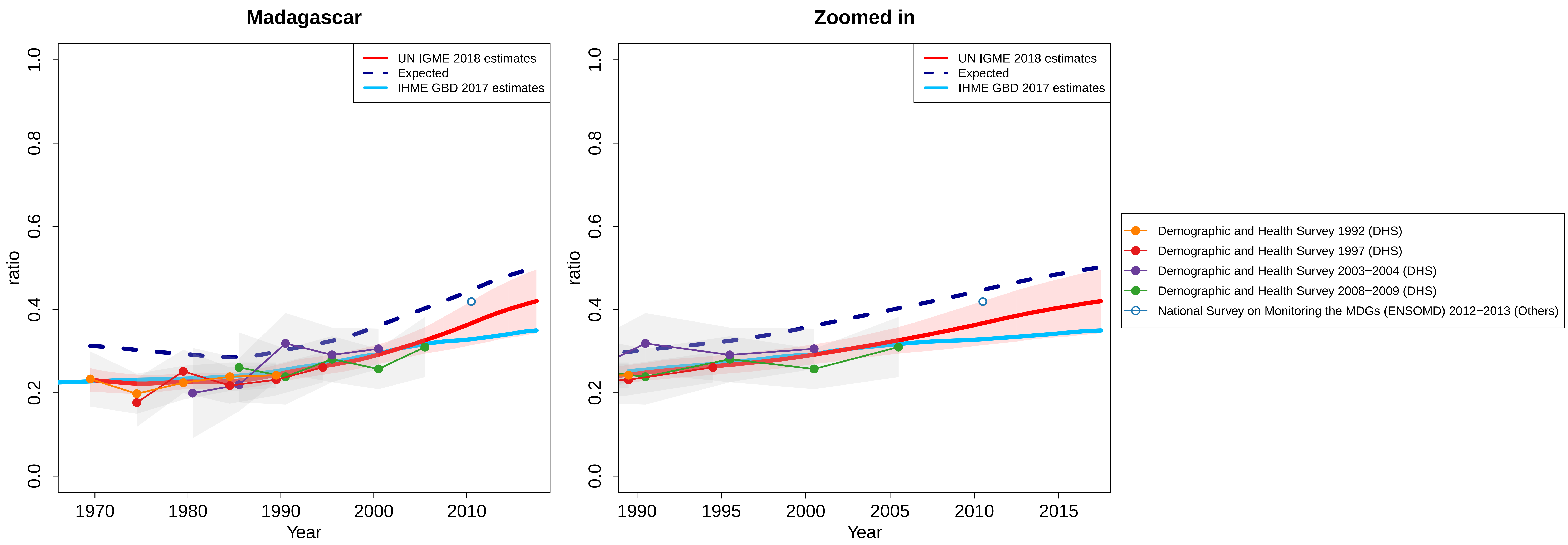

Madagascar
Zoomed in
ratio
Year
1.0
0.8
0.6
0.4
0.2
0.0
1970
1980
1990
2000
2010
1995
2005
2015
UN IGME 2018 estimates
Expected
IHME GBD 2017 estimates
Demographic and Health Survey 1992 (DHS)
Demographic and Health Survey 1997 (DHS)
Demographic and Health Survey 2003−2004 (DHS)
Demographic and Health Survey 2008−2009 (DHS)
National Survey on Monitoring the MDGs (ENSOMD) 2012−2013 (Others)

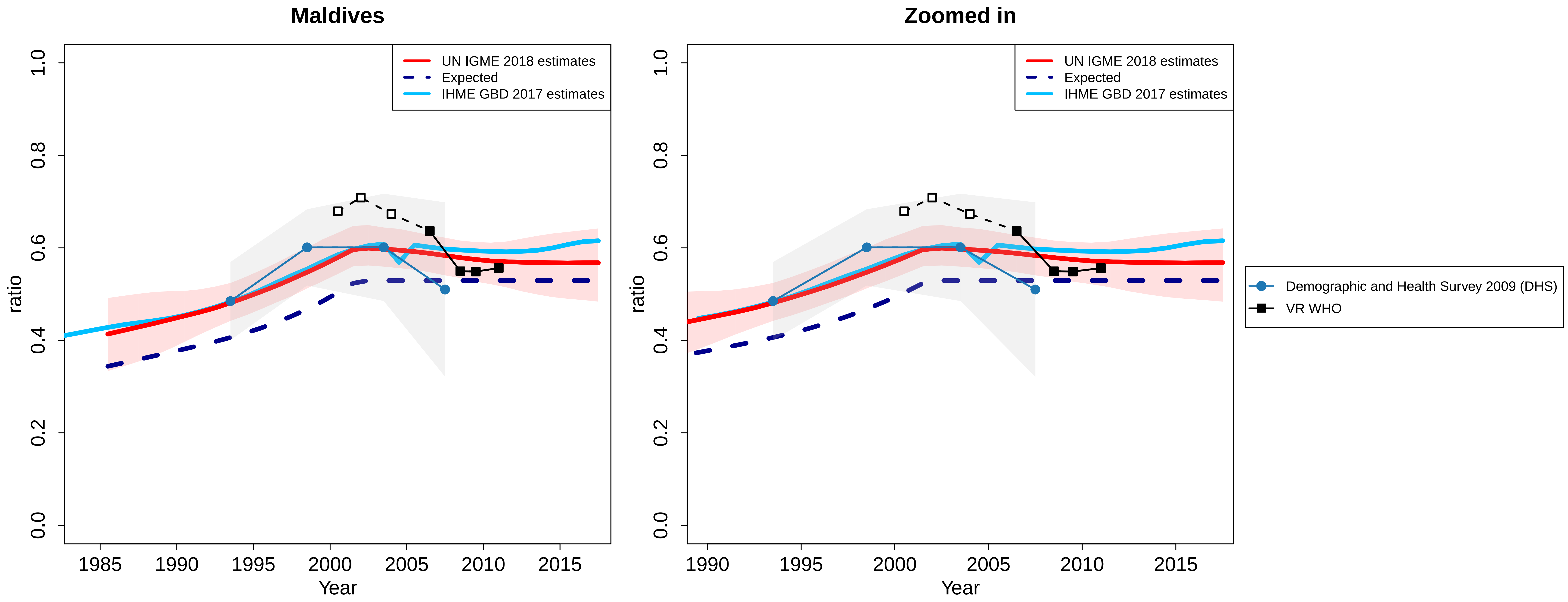

Maldives
Zoomed in
UN IGME 2018 estimates
Expected
IHME GBD 2017 estimates
Demographic and Health Survey 2009 (DHS)
VR WHO
ratio
Year
0.0
0.2
0.4
0.6
0.8
1.0
1985
1990
1995
2000
2005
2010
2015

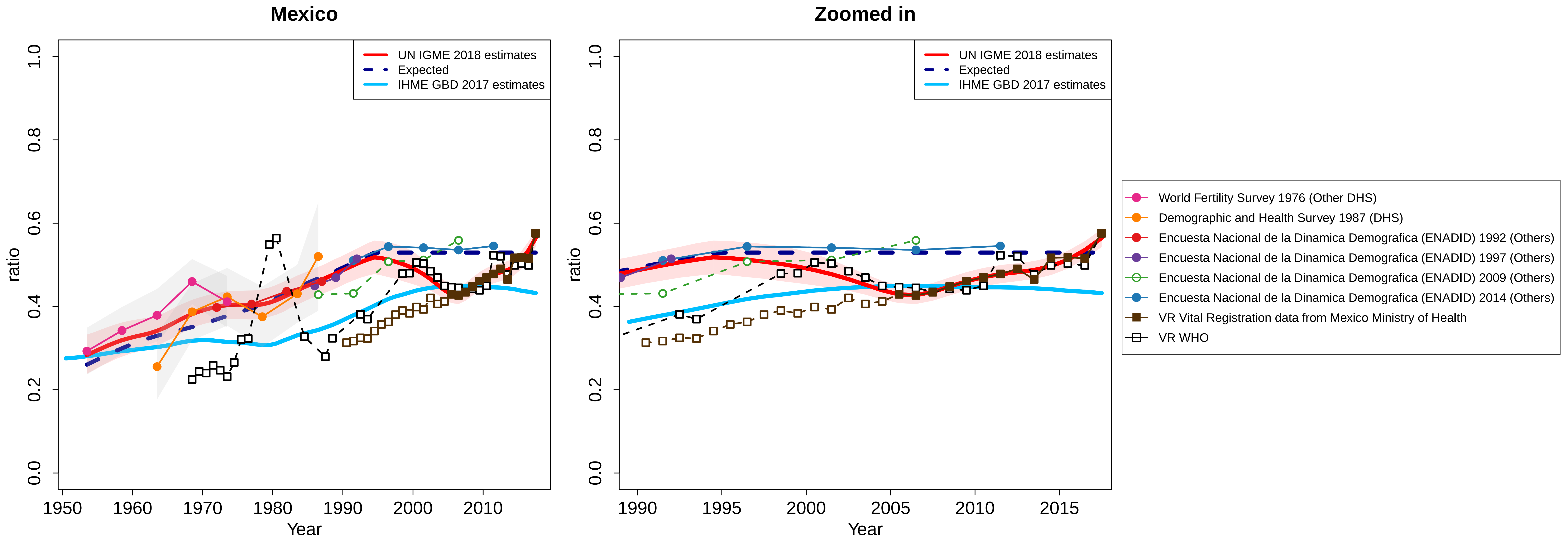

Mexico
Zoomed in
UN IGME 2018 estimates
Expected
IHME GBD 2017 estimates
ratio
Year
0.0
0.2
0.4
0.6
0.8
1.0
1950
1960
1970
1980
1990
2000
2010
1995
2005
2015
World Fertility Survey 1976 (Other DHS)
Demographic and Health Survey 1987 (DHS)
Encuesta Nacional de la Dinamica Demografica (ENADID) 1992 (Others)
Encuesta Nacional de la Dinamica Demografica (ENADID) 1997 (Others)
Encuesta Nacional de la Dinamica Demografica (ENADID) 2009 (Others)
Encuesta Nacional de la Dinamica Demografica (ENADID) 2014 (Others)
VR Vital Registration data from Mexico Ministry of Health
VR WHO

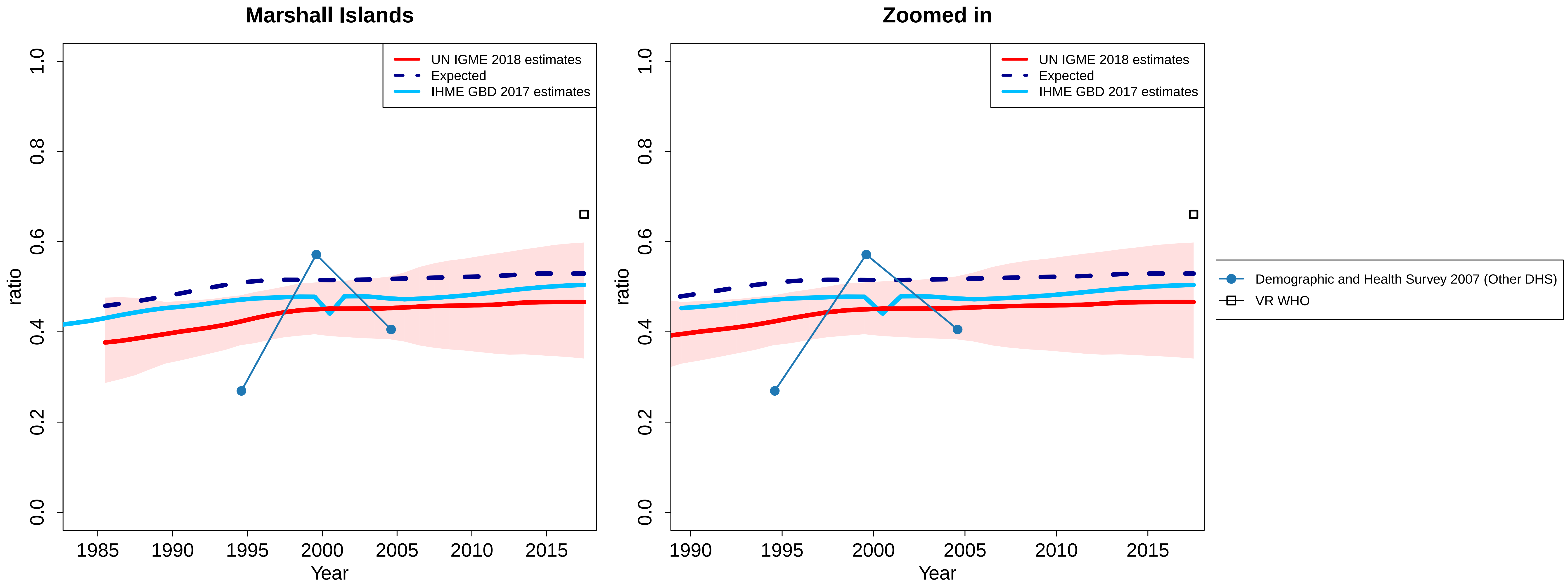

Marshall Islands
Zoomed in
UN IGME 2018 estimates
Expected
IHME GBD 2017 estimates
ratio
Year
0.0
0.2
0.4
0.6
0.8
1.0
1985
1990
1995
2000
2005
2010
2015
Demographic and Health Survey 2007 (Other DHS)
VR WHO

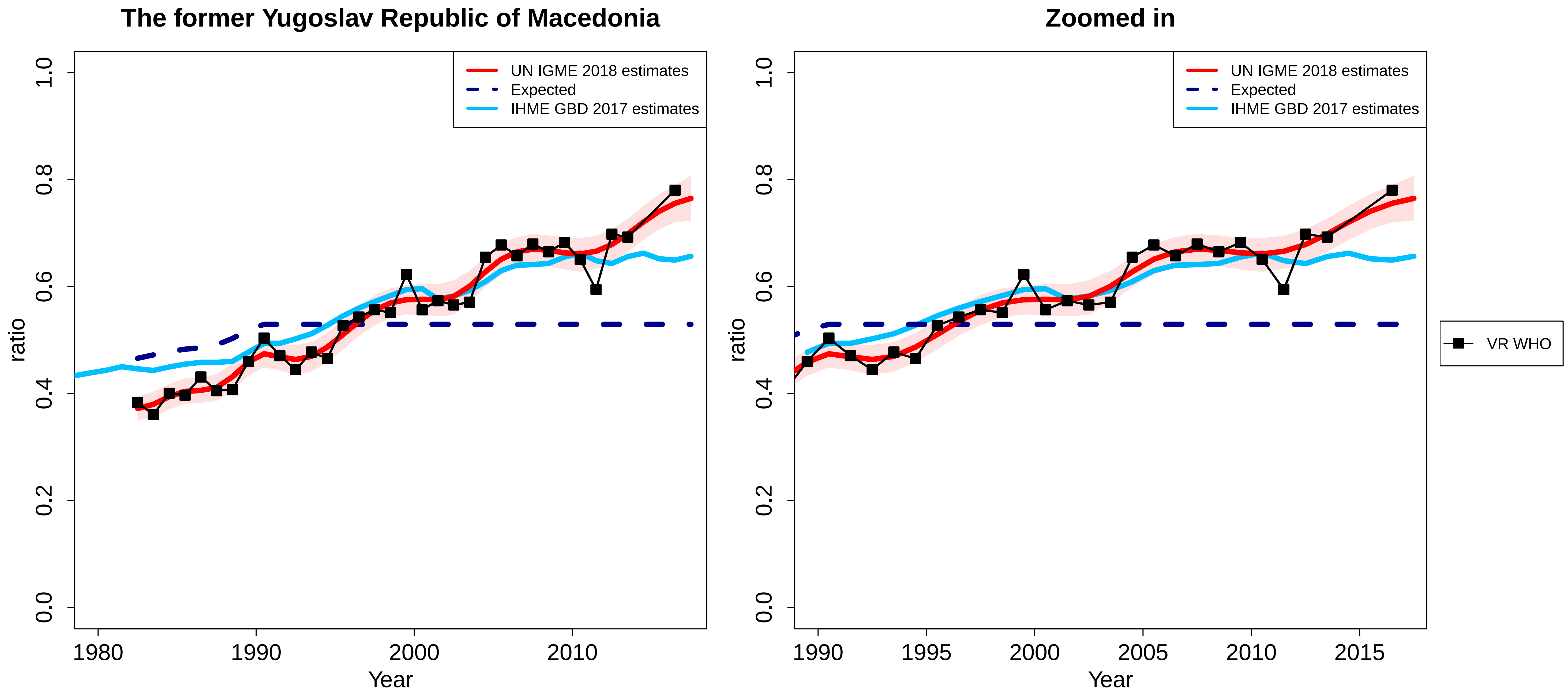

The former Yugoslav Republic of Macedonia
Zoomed in
UN IGME 2018 estimates
Expected
IHME GBD 2017 estimates
VR WHO
ratio
Year
0.0
0.2
0.4
0.6
0.8
1.0
1980
1990
2000
2010
1995
2005
2015

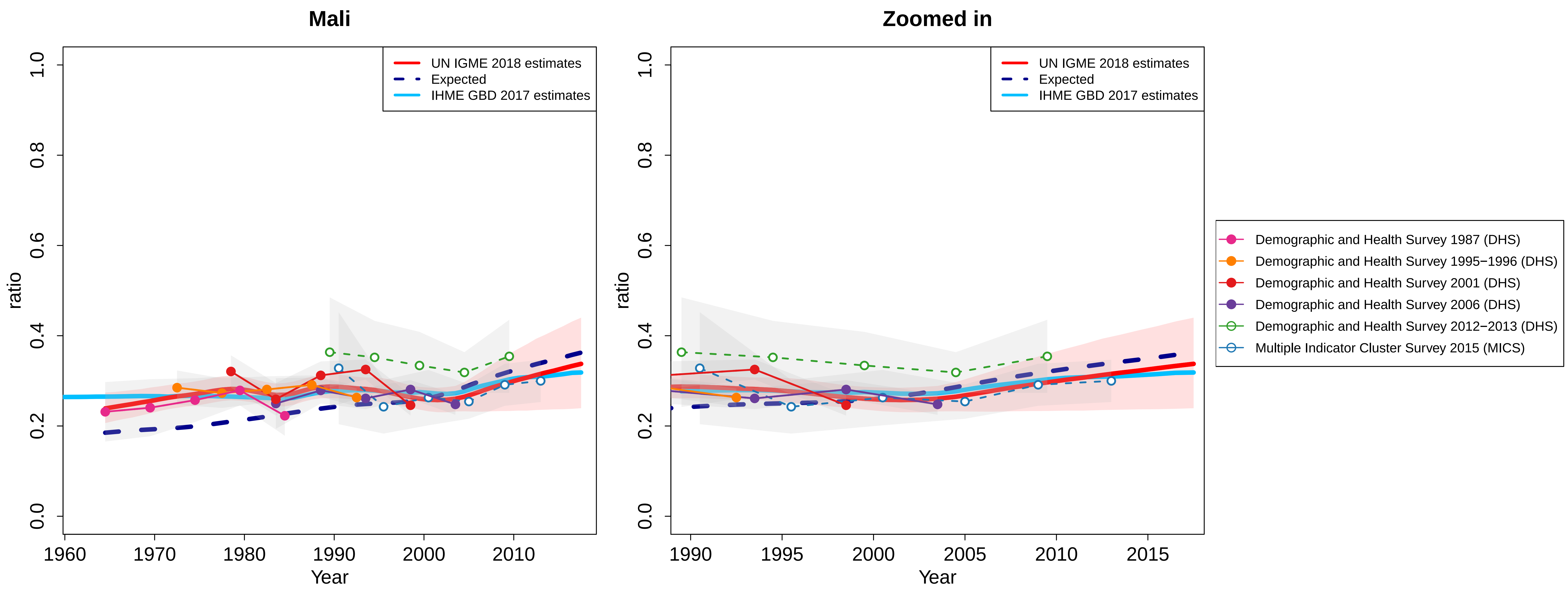

Mali
Zoomed in
UN IGME 2018 estimates
Expected
IHME GBD 2017 estimates
ratio
Year
0.0
0.2
0.4
0.6
0.8
1.0
1960
1970
1980
1990
2000
2010
1995
2005
2015
Demographic and Health Survey 1987 (DHS)
Demographic and Health Survey 1995−1996 (DHS)
Demographic and Health Survey 2001 (DHS)
Demographic and Health Survey 2006 (DHS)
Demographic and Health Survey 2012−2013 (DHS)
Multiple Indicator Cluster Survey 2015 (MICS)

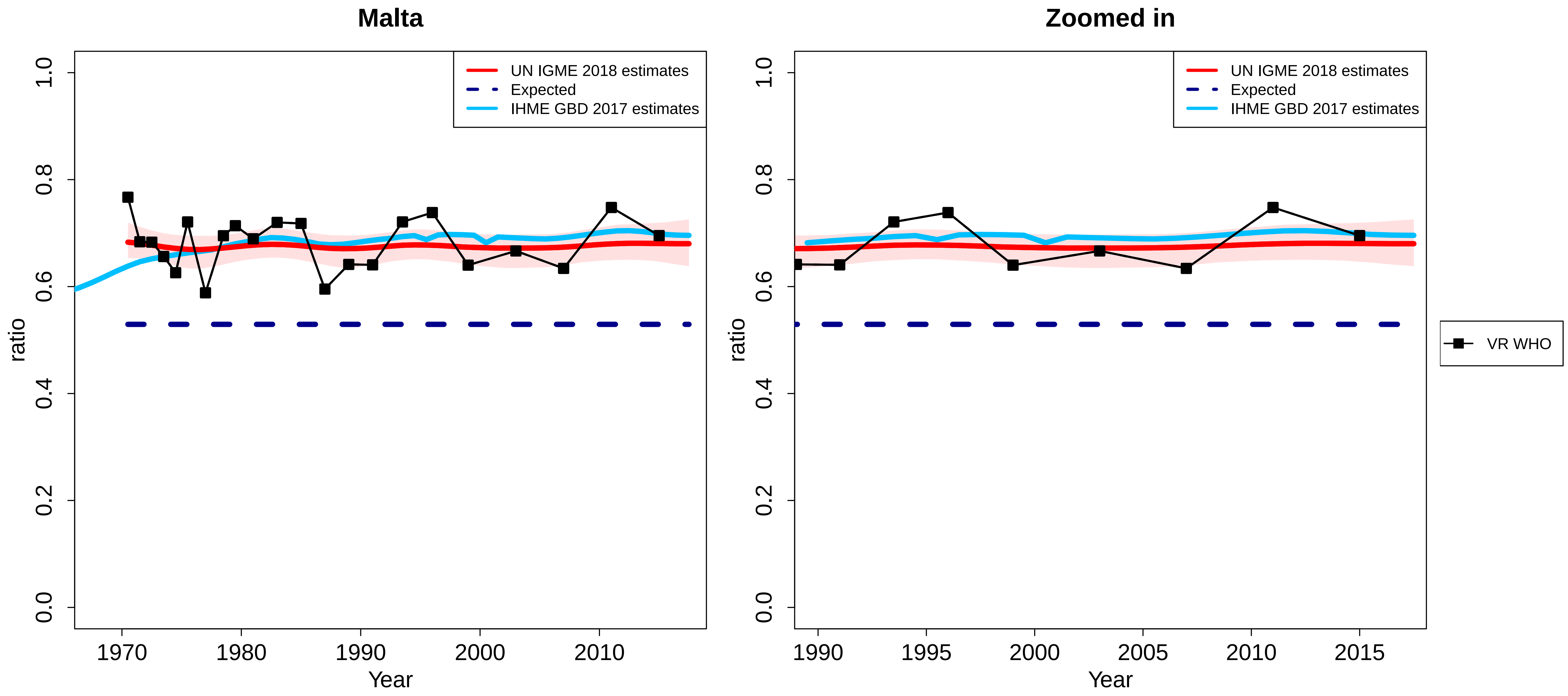

Malta
Zoomed in
UN IGME 2018 estimates
Expected
IHME GBD 2017 estimates
VR WHO
ratio
Year
0.0
0.2
0.4
0.6
0.8
1.0
1970
1980
1990
2000
2010
1995
2005
2015

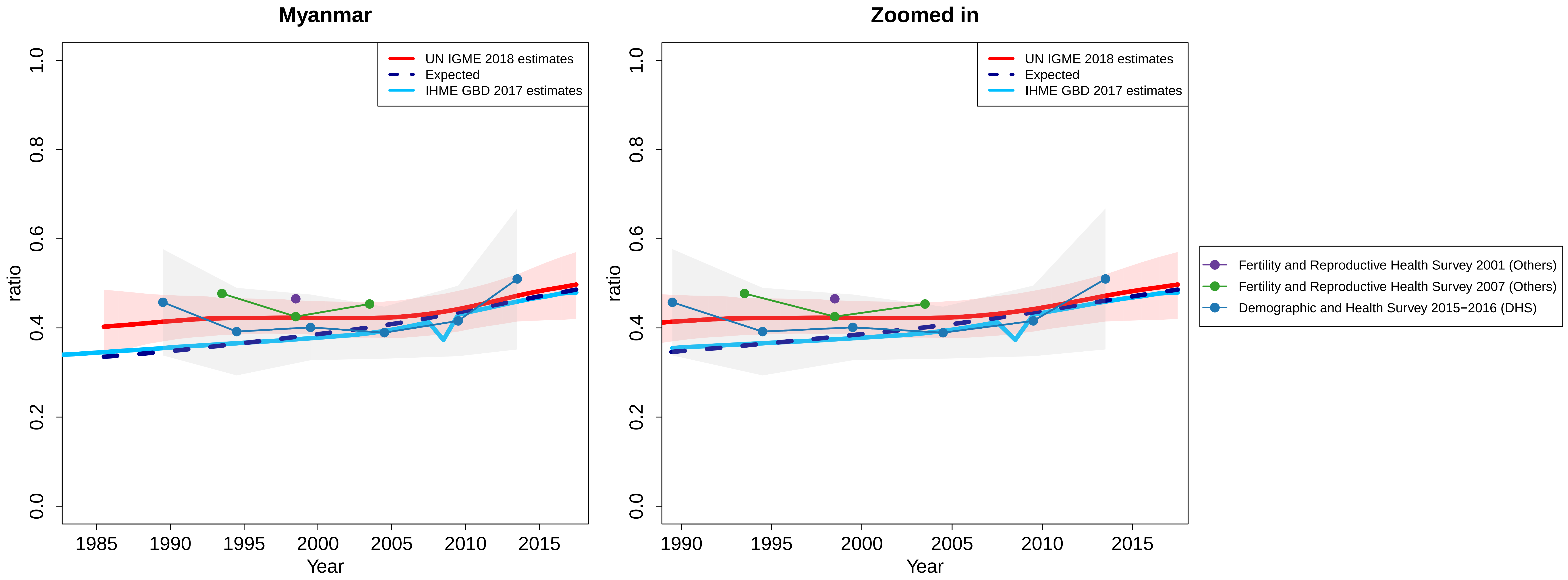

Myanmar
Zoomed in
UN IGME 2018 estimates
Expected
IHME GBD 2017 estimates
ratio
Year
0.0
0.2
0.4
0.6
0.8
1.0
1985
1990
1995
2000
2005
2010
2015
Fertility and Reproductive Health Survey 2001 (Others)
Fertility and Reproductive Health Survey 2007 (Others)
Demographic and Health Survey 2015−2016 (DHS)

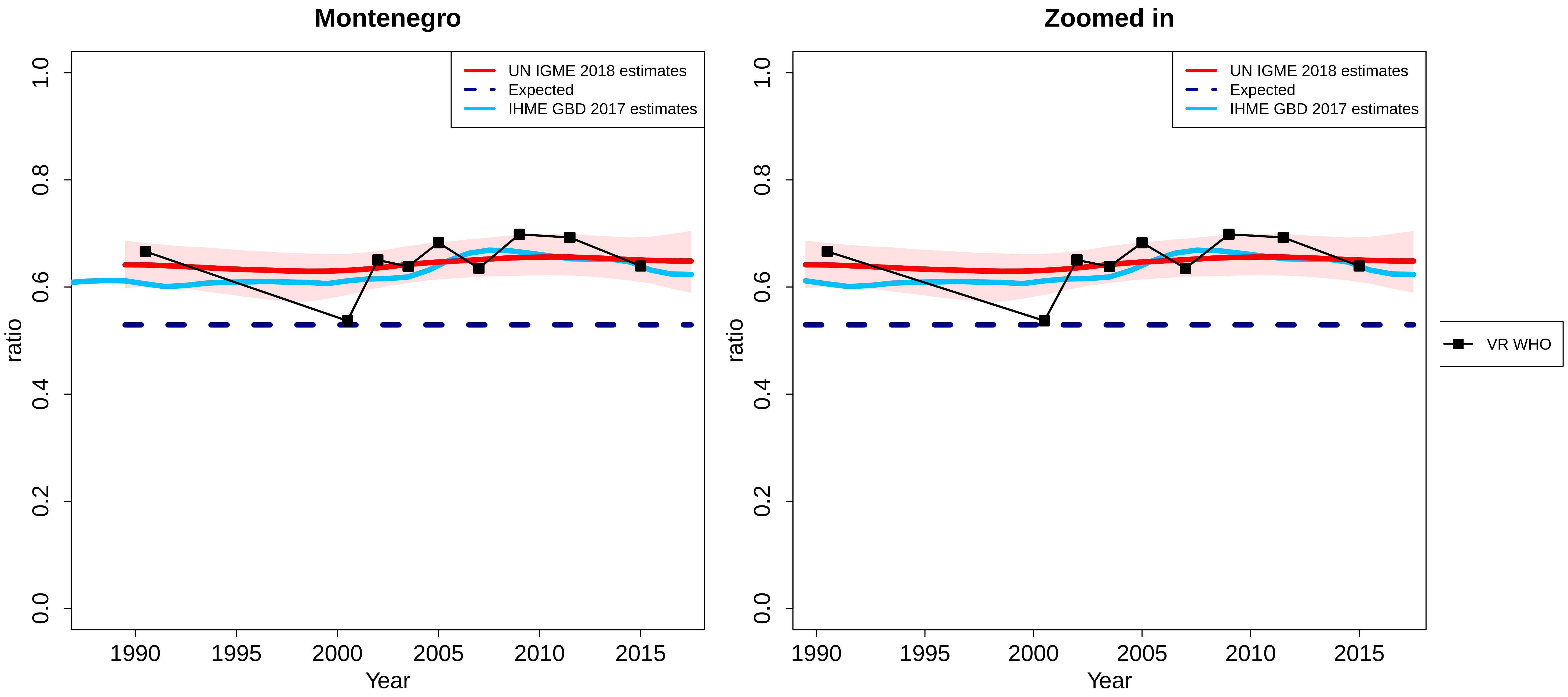

Montenegro
Zoomed in
UN IGME 2018 estimates
Expected
IHME GBD 2017 estimates
VR WHO
ratio
Year
0.0
0.2
0.4
0.6
0.8
1.0
1990
1995
2000
2005
2010
2015

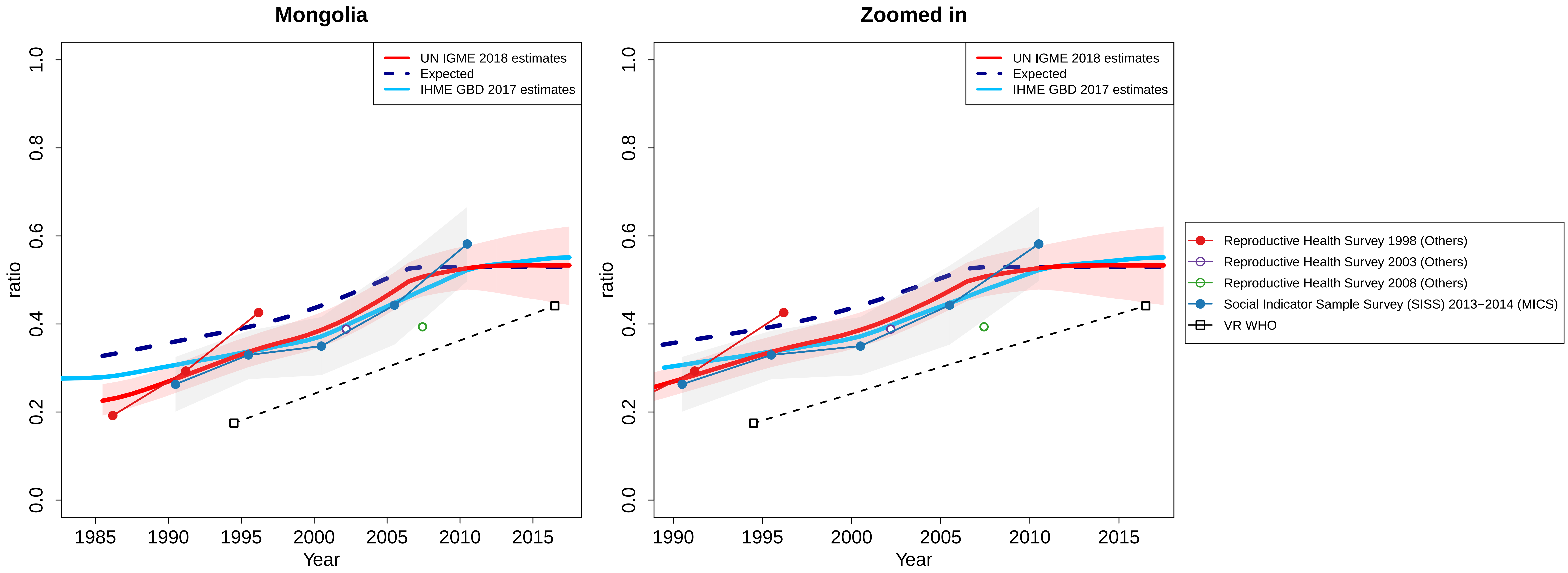

Mongolia
Zoomed in
UN IGME 2018 estimates
Expected
IHME GBD 2017 estimates
ratio
Year
0.0
0.2
0.4
0.6
0.8
1.0
1985
1990
1995
2000
2005
2010
2015
Reproductive Health Survey 1998 (Others)
Reproductive Health Survey 2003 (Others)
Reproductive Health Survey 2008 (Others)
Social Indicator Sample Survey (SISS) 2013−2014 (MICS)
VR WHO

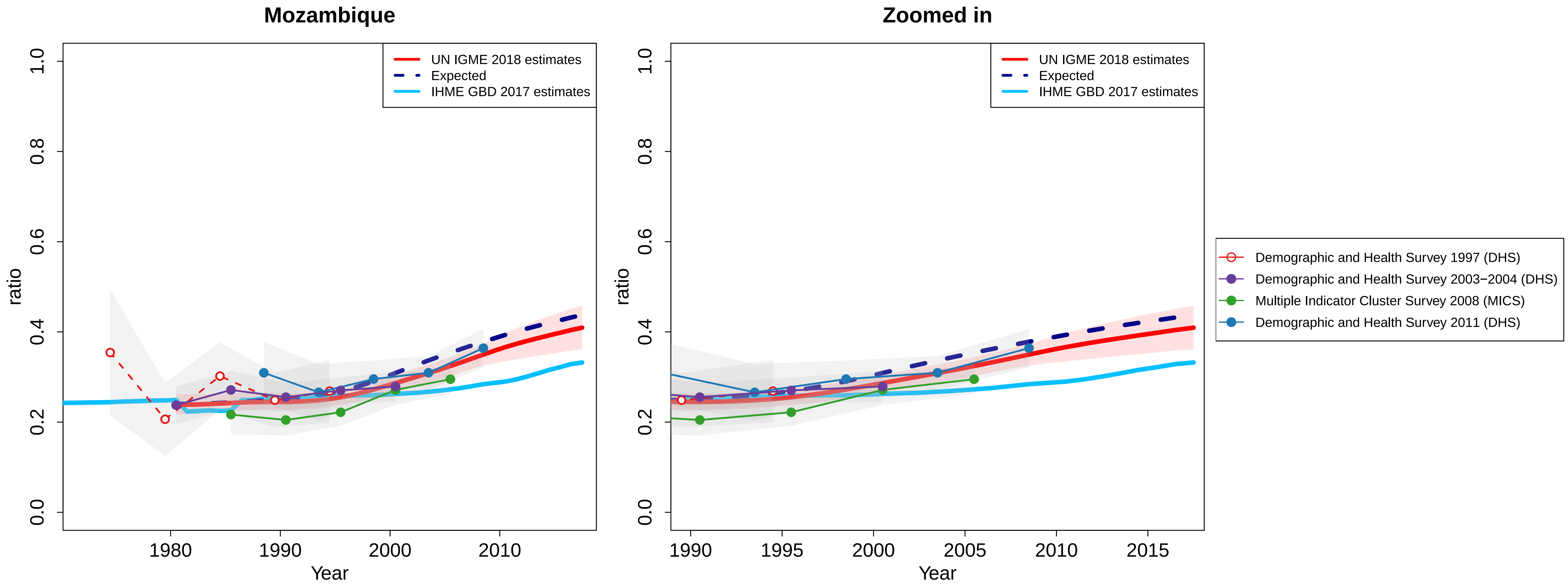
Mozambique
Zoomed in
ratio
Year
UN IGME 2018 estimates
Expected
IHME GBD 2017 estimates
Demographic and Health Survey 1997 (DHS)
Demographic and Health Survey 2003−2004 (DHS)
Multiple Indicator Cluster Survey 2008 (MICS)
Demographic and Health Survey 2011 (DHS)

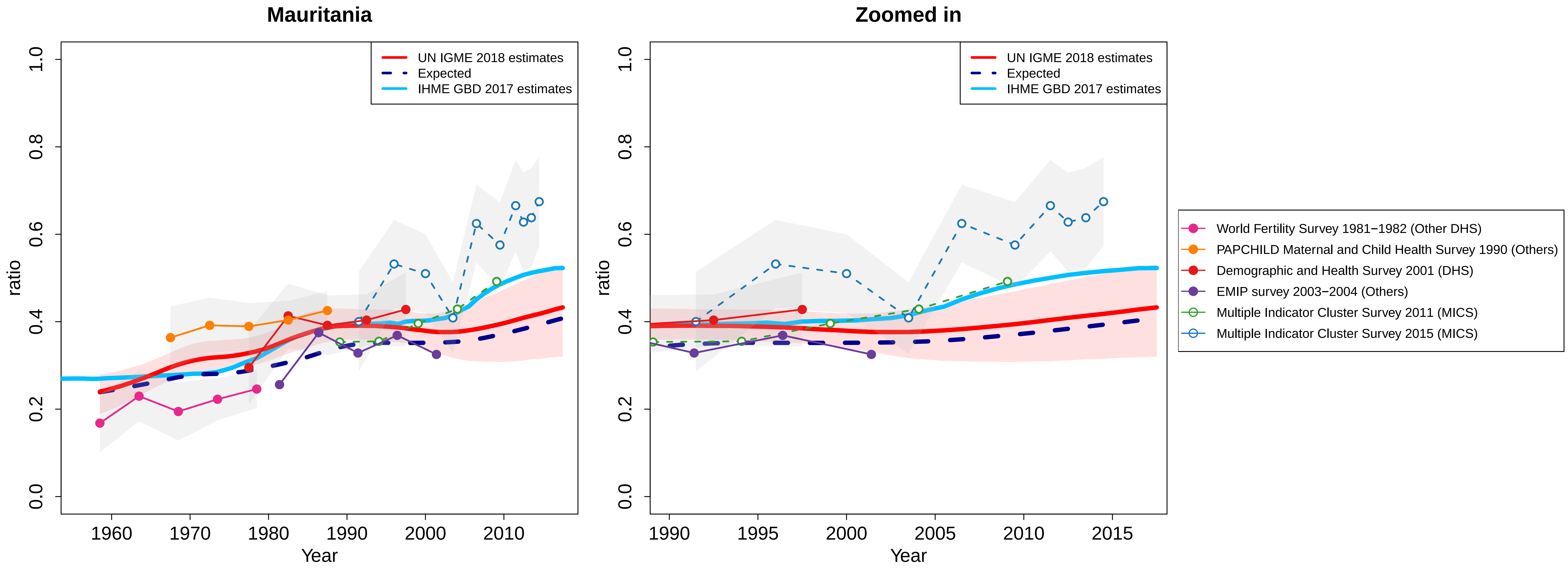

Mauritania
Zoomed in
UN IGME 2018 estimates
Expected
IHME GBD 2017 estimates
ratio
Year
World Fertility Survey 1981−1982 (Other DHS)
PAPCHILD Maternal and Child Health Survey 1990 (Others)
Demographic and Health Survey 2001 (DHS)
EMIP survey 2003−2004 (Others)
Multiple Indicator Cluster Survey 2011 (MICS)
Multiple Indicator Cluster Survey 2015 (MICS)

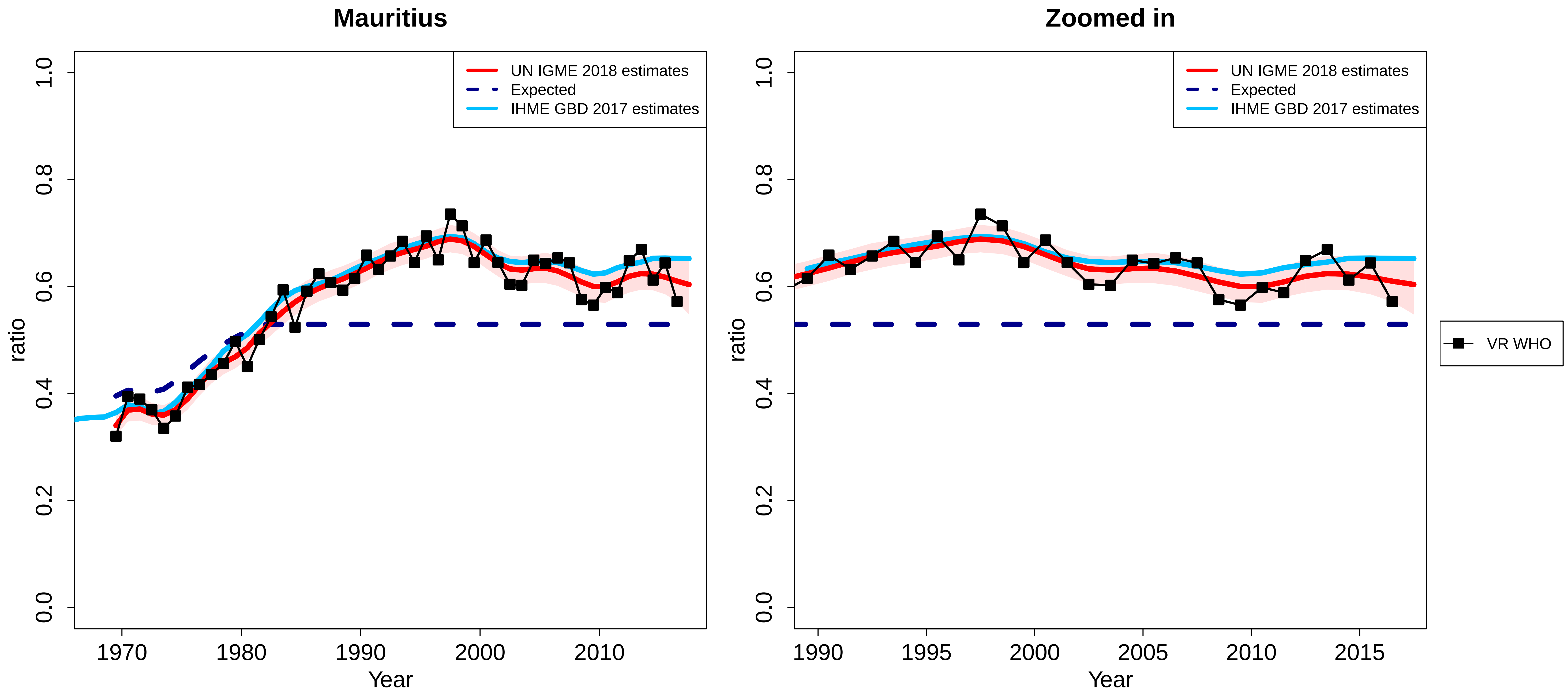
Mauritius
Zoomed in
UN IGME 2018 estimates
Expected
IHME GBD 2017 estimates
VR WHO
ratio
Year
0.0
0.2
0.4
0.6
0.8
1.0
1970
1980
1990
2000
2010
1990
1995
2000
2005
2010
2015

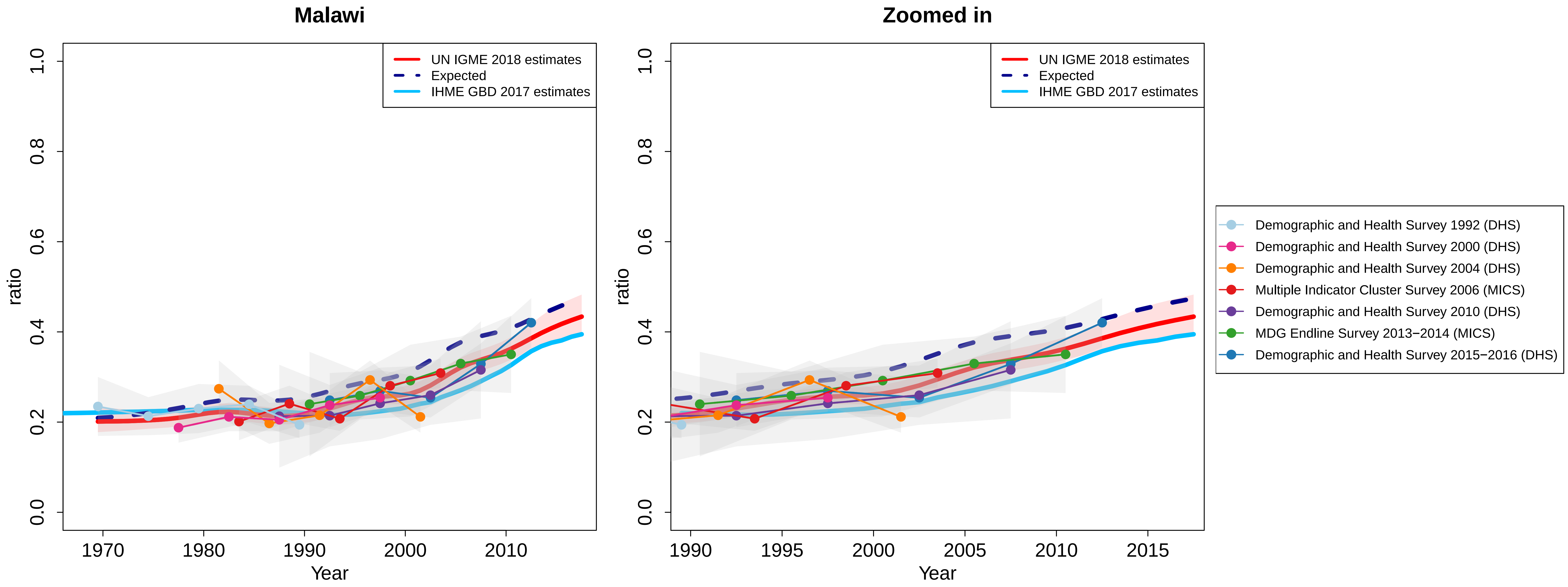
Malawi
Zoomed in
UN IGME 2018 estimates
Expected
IHME GBD 2017 estimates
ratio
Year
0.0
0.2
0.4
0.6
0.8
1.0
1970
1980
1990
2000
2010
1995
2005
2015
Demographic and Health Survey 1992 (DHS)
Demographic and Health Survey 2000 (DHS)
Demographic and Health Survey 2004 (DHS)
Multiple Indicator Cluster Survey 2006 (MICS)
Demographic and Health Survey 2010 (DHS)
MDG Endline Survey 2013−2014 (MICS)
Demographic and Health Survey 2015−2016 (DHS)

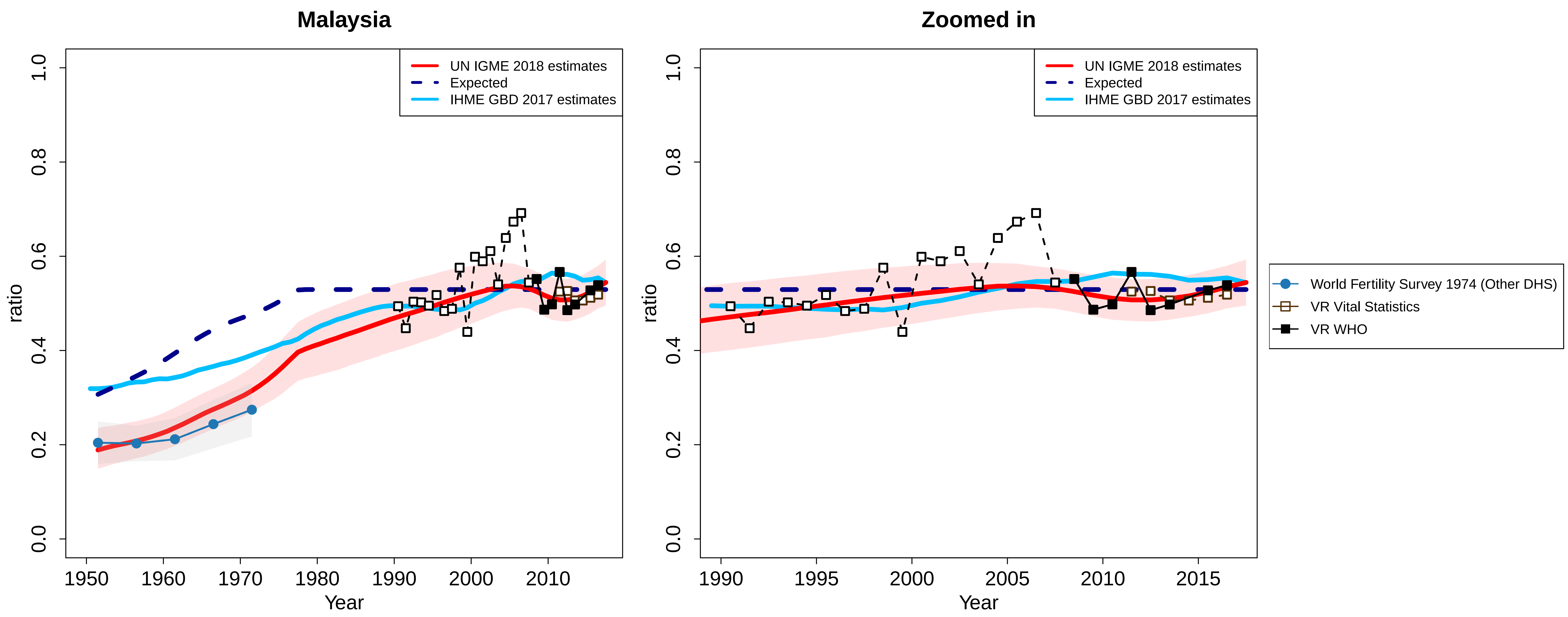

Malaysia
Zoomed in
UN IGME 2018 estimates
Expected
IHME GBD 2017 estimates
ratio
Year
0.0
0.2
0.4
0.6
0.8
1.0
1950
1960
1970
1980
1990
2000
2010
1995
2005
2015
World Fertility Survey 1974 (Other DHS)
VR Vital Statistics
VR WHO

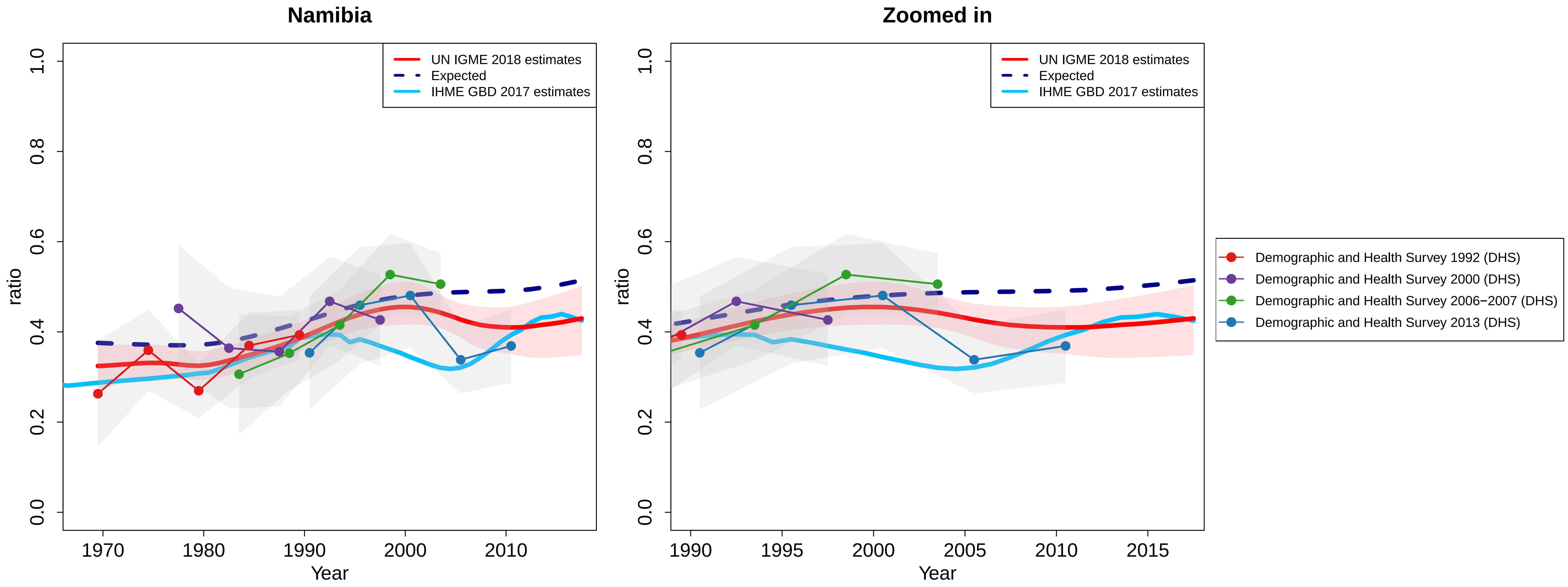

Namibia
Zoomed in
UN IGME 2018 estimates
Expected
IHME GBD 2017 estimates
ratio
Year
Demographic and Health Survey 1992 (DHS)
Demographic and Health Survey 2000 (DHS)
Demographic and Health Survey 2006−2007 (DHS)
Demographic and Health Survey 2013 (DHS)

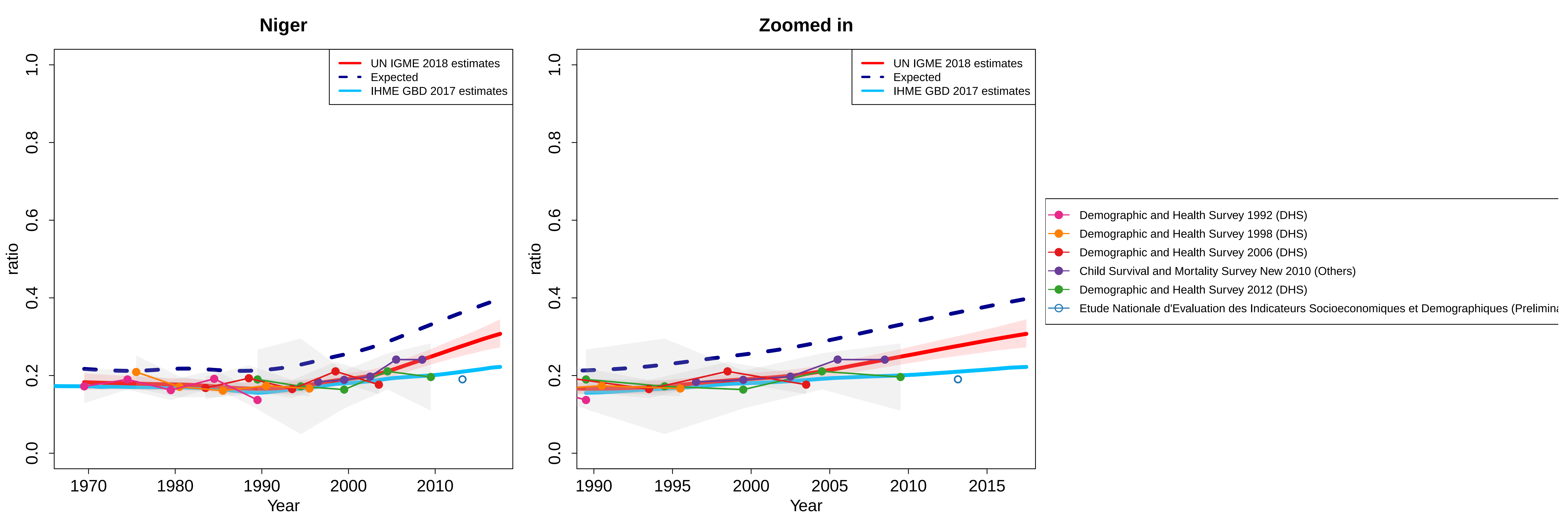

Niger
Zoomed in
UN IGME 2018 estimates
Expected
IHME GBD 2017 estimates
ratio
Year
Demographic and Health Survey 1992 (DHS)
Demographic and Health Survey 1998 (DHS)
Demographic and Health Survey 2006 (DHS)
Child Survival and Mortality Survey New 2010 (Others)
Demographic and Health Survey 2012 (DHS)
Etude Nationale d'Evaluation des Indicateurs Socioeconomiques et Demographiques (Prelimina

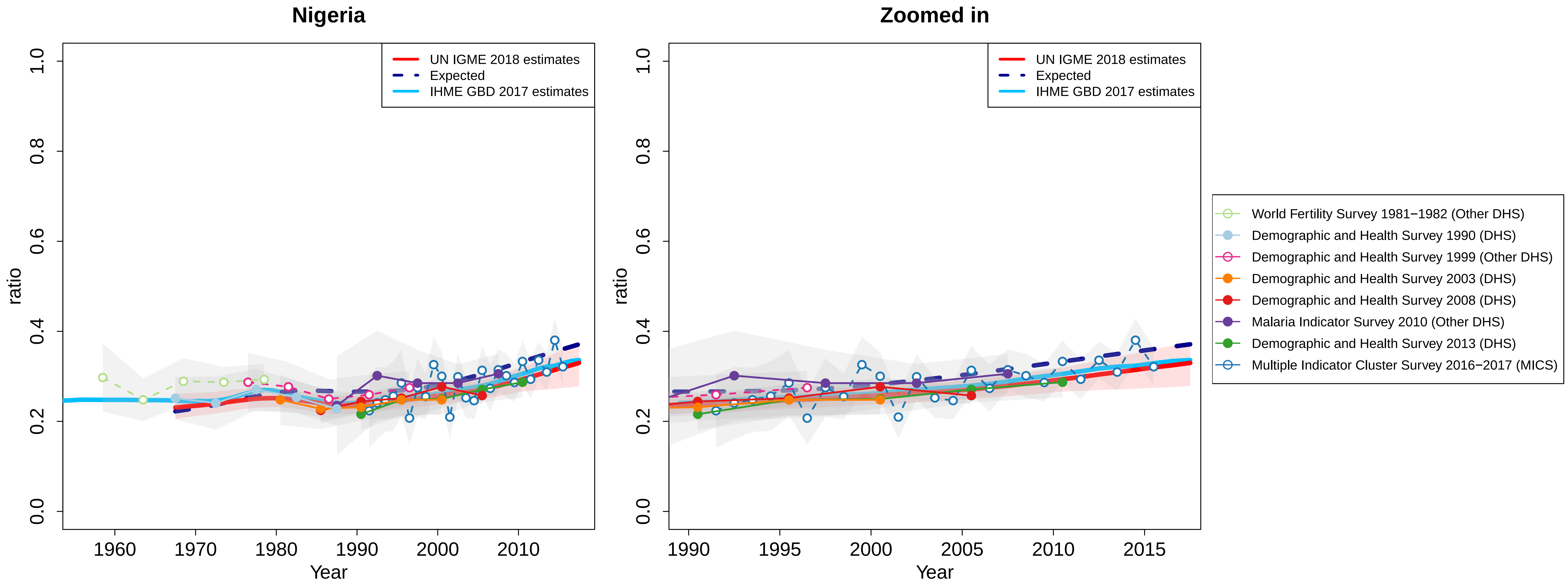

Nigeria
Zoomed in
UN IGME 2018 estimates
Expected
IHME GBD 2017 estimates
ratio
Year
0.0
0.2
0.4
0.6
0.8
1.0
1960
1970
1980
1990
2000
2010
1995
2005
2015
World Fertility Survey 1981−1982 (Other DHS)
Demographic and Health Survey 1990 (DHS)
Demographic and Health Survey 1999 (Other DHS)
Demographic and Health Survey 2003 (DHS)
Demographic and Health Survey 2008 (DHS)
Malaria Indicator Survey 2010 (Other DHS)
Demographic and Health Survey 2013 (DHS)
Multiple Indicator Cluster Survey 2016−2017 (MICS)

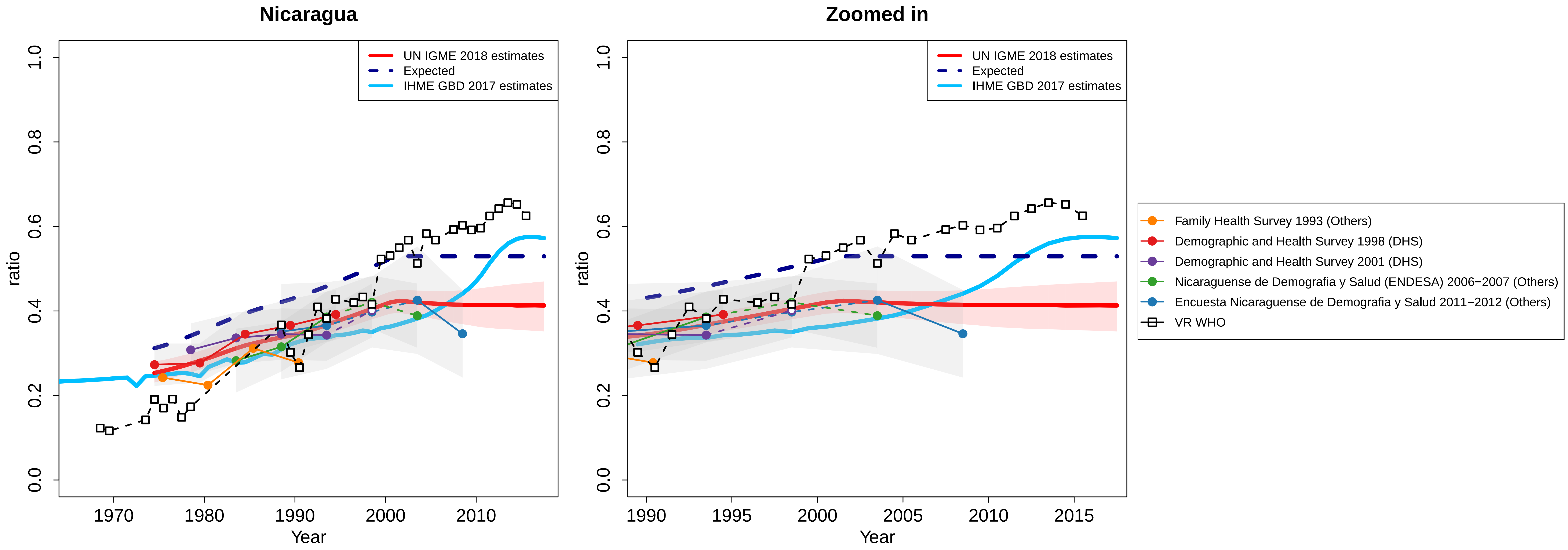

Nicaragua
Zoomed in
UN IGME 2018 estimates
Expected
IHME GBD 2017 estimates
ratio
Year
0.0
0.2
0.4
0.6
0.8
1.0
1970
1980
1990
2000
2010
1995
2005
2015
Family Health Survey 1993 (Others)
Demographic and Health Survey 1998 (DHS)
Demographic and Health Survey 2001 (DHS)
Nicaraguense de Demografia y Salud (ENDESA) 2006−2007 (Others)
Encuesta Nicaraguense de Demografia y Salud 2011−2012 (Others)
VR WHO

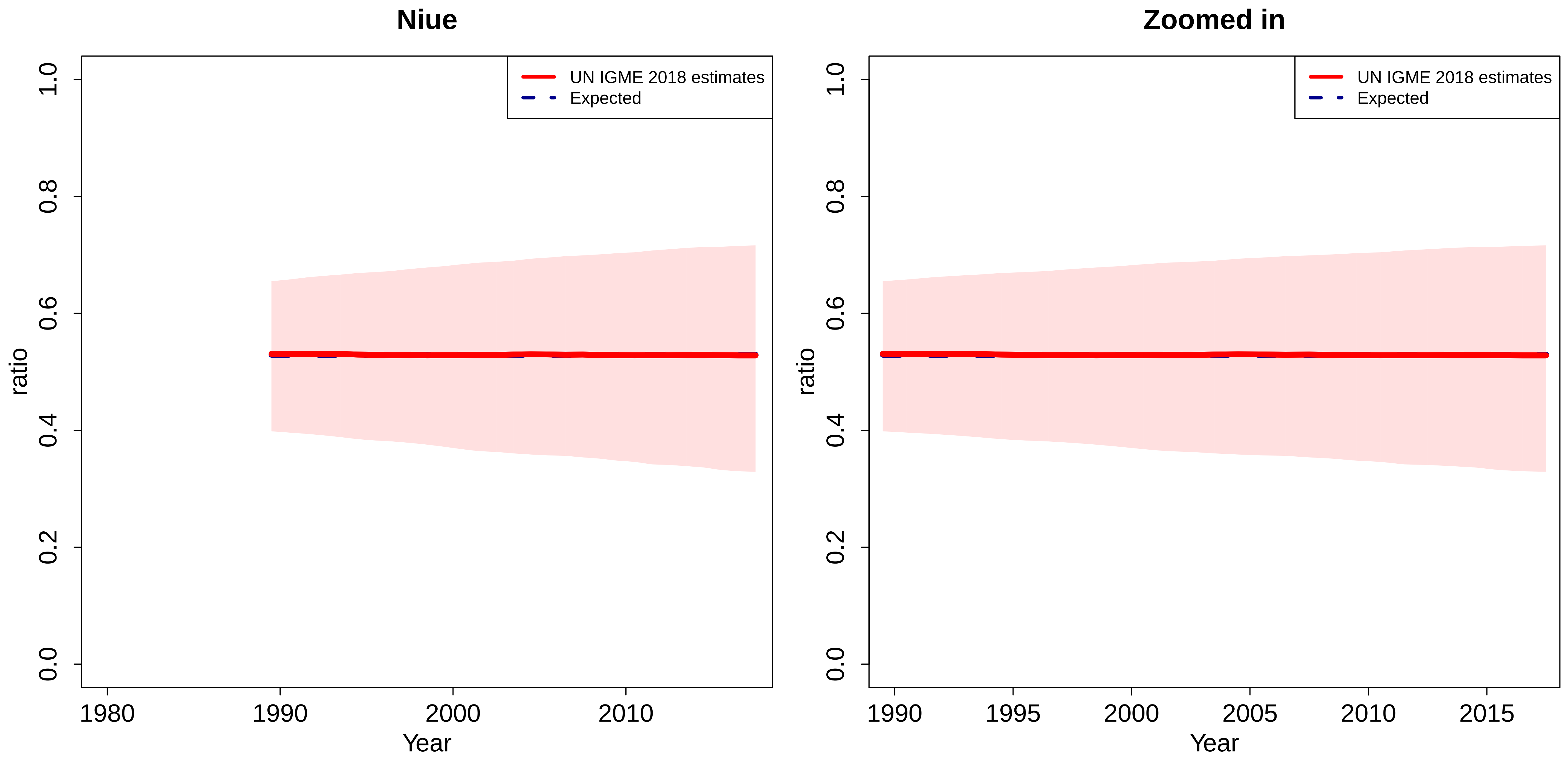

Niue
UN IGME 2018 estimates
Expected
ratio
0.0
0.2
0.4
0.6
0.8
1.0
1980
1990
2000
2010
Year
Zoomed in
UN IGME 2018 estimates
Expected
ratio
0.0
0.2
0.4
0.6
0.8
1.0
1990
1995
2000
2005
2010
2015
Year

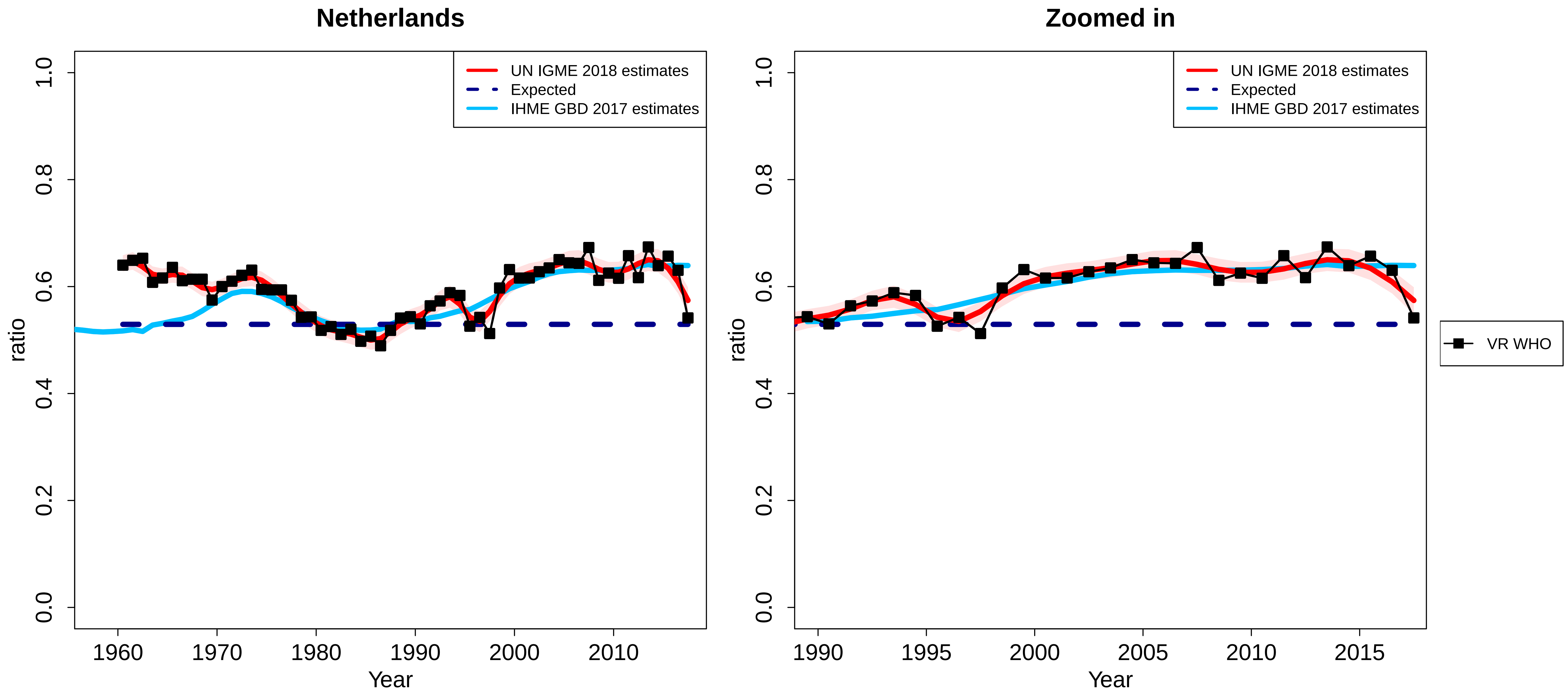
Netherlands
Zoomed in
UN IGME 2018 estimates
Expected
IHME GBD 2017 estimates
VR WHO
ratio
Year
0.0
0.2
0.4
0.6
0.8
1.0
1960
1970
1980
1990
2000
2010
1995
2005
2015

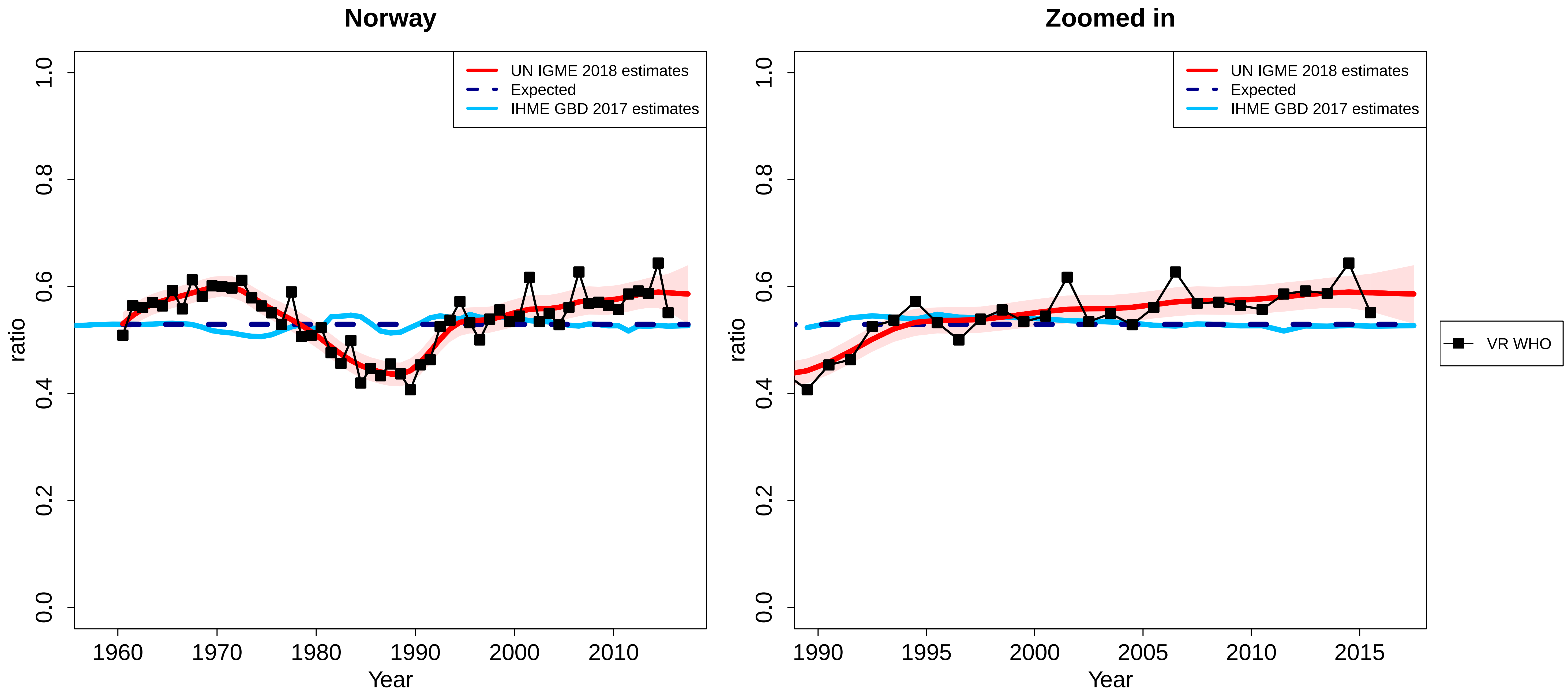

Norway
Zoomed in
UN IGME 2018 estimates
Expected
IHME GBD 2017 estimates
VR WHO
ratio
Year
0.0
0.2
0.4
0.6
0.8
1.0
1960
1970
1980
1990
2000
2010
1995
2005
2015

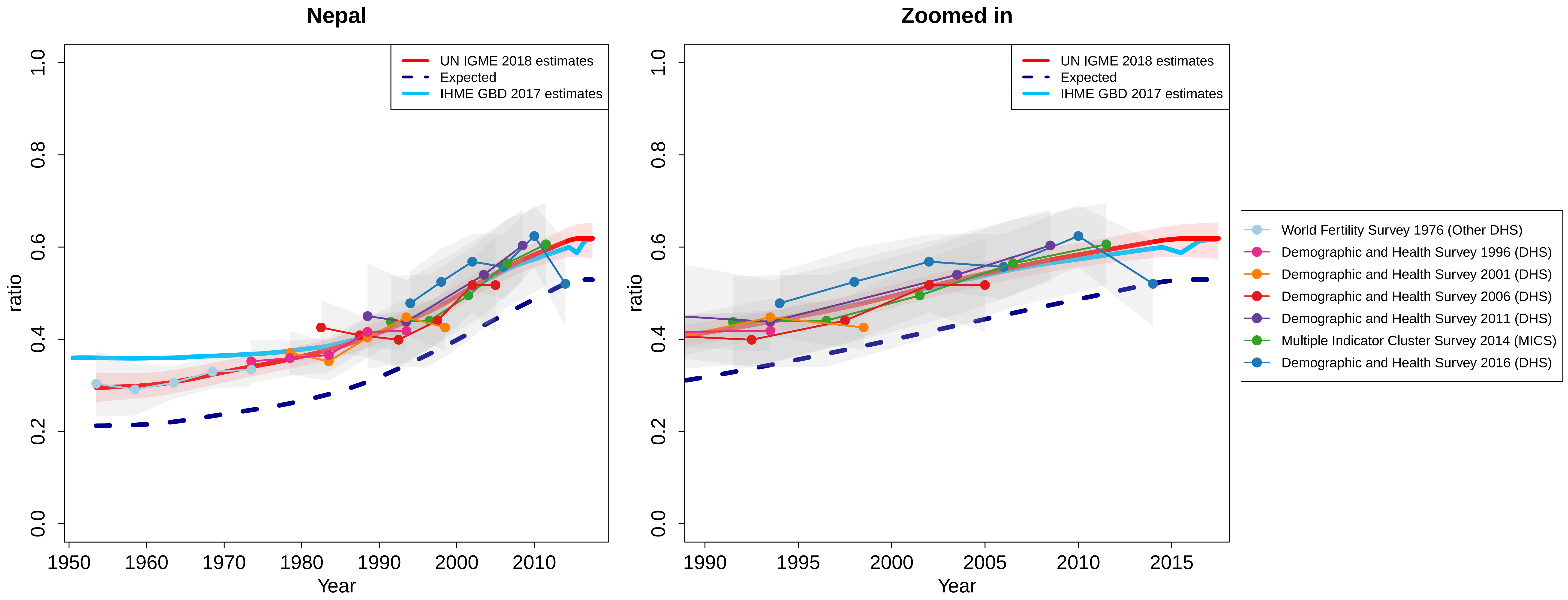
Nepal
Zoomed in
UN IGME 2018 estimates
Expected
IHME GBD 2017 estimates
ratio
Year
World Fertility Survey 1976 (Other DHS)
Demographic and Health Survey 1996 (DHS)
Demographic and Health Survey 2001 (DHS)
Demographic and Health Survey 2006 (DHS)
Demographic and Health Survey 2011 (DHS)
Multiple Indicator Cluster Survey 2014 (MICS)
Demographic and Health Survey 2016 (DHS)

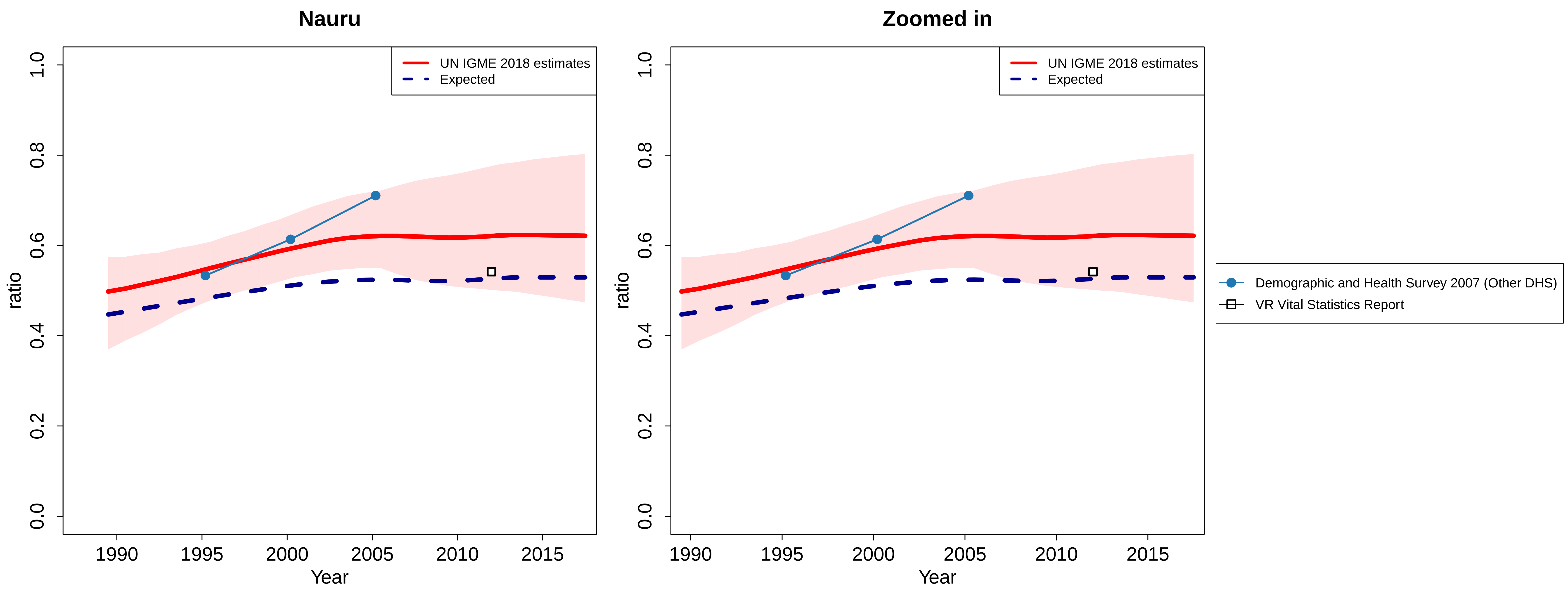

Nauru
Zoomed in
UN IGME 2018 estimates
Expected
ratio
Year
0.0
0.2
0.4
0.6
0.8
1.0
1990
1995
2000
2005
2010
2015
Demographic and Health Survey 2007 (Other DHS)
VR Vital Statistics Report

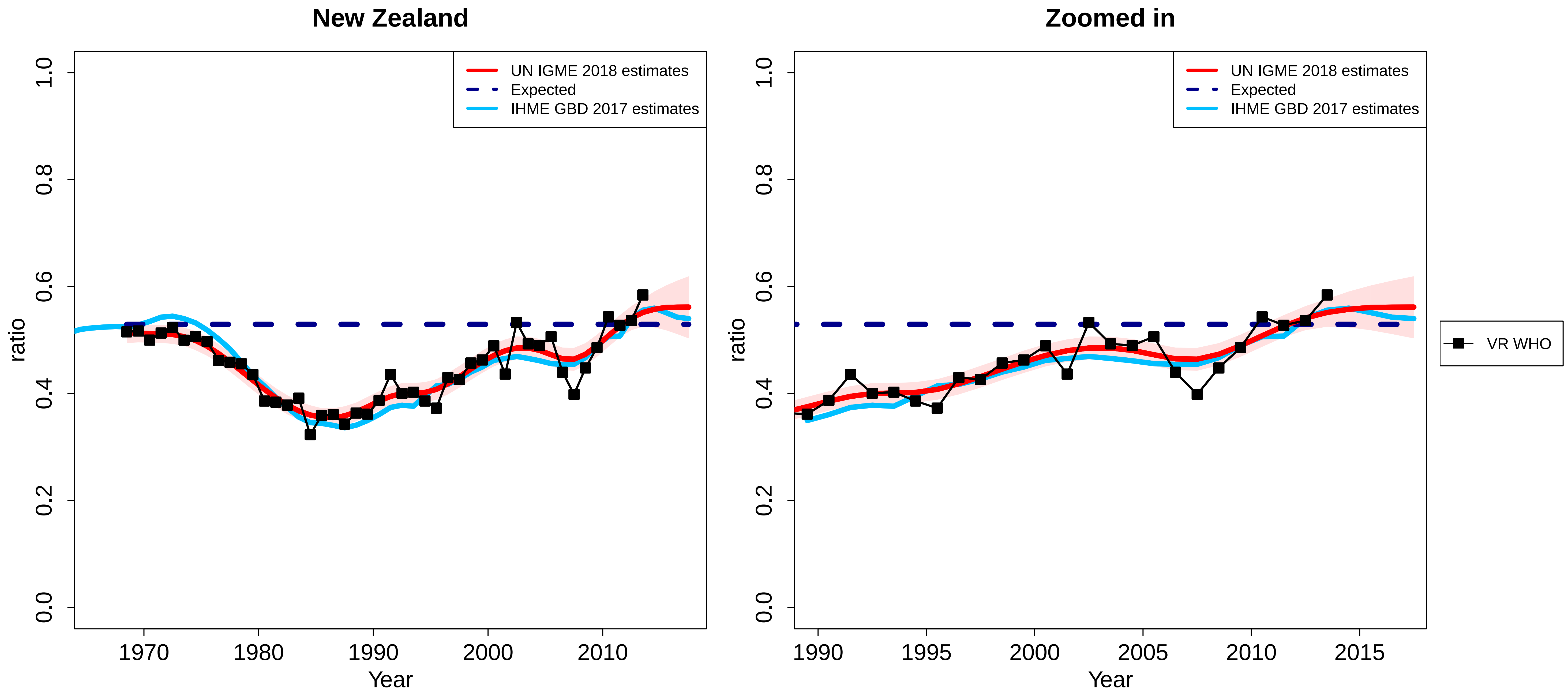

New Zealand
Zoomed in
UN IGME 2018 estimates
Expected
IHME GBD 2017 estimates
VR WHO
ratio
Year
0.0
0.2
0.4
0.6
0.8
1.0
1970
1980
1990
2000
2010
1990
1995
2000
2005
2010
2015

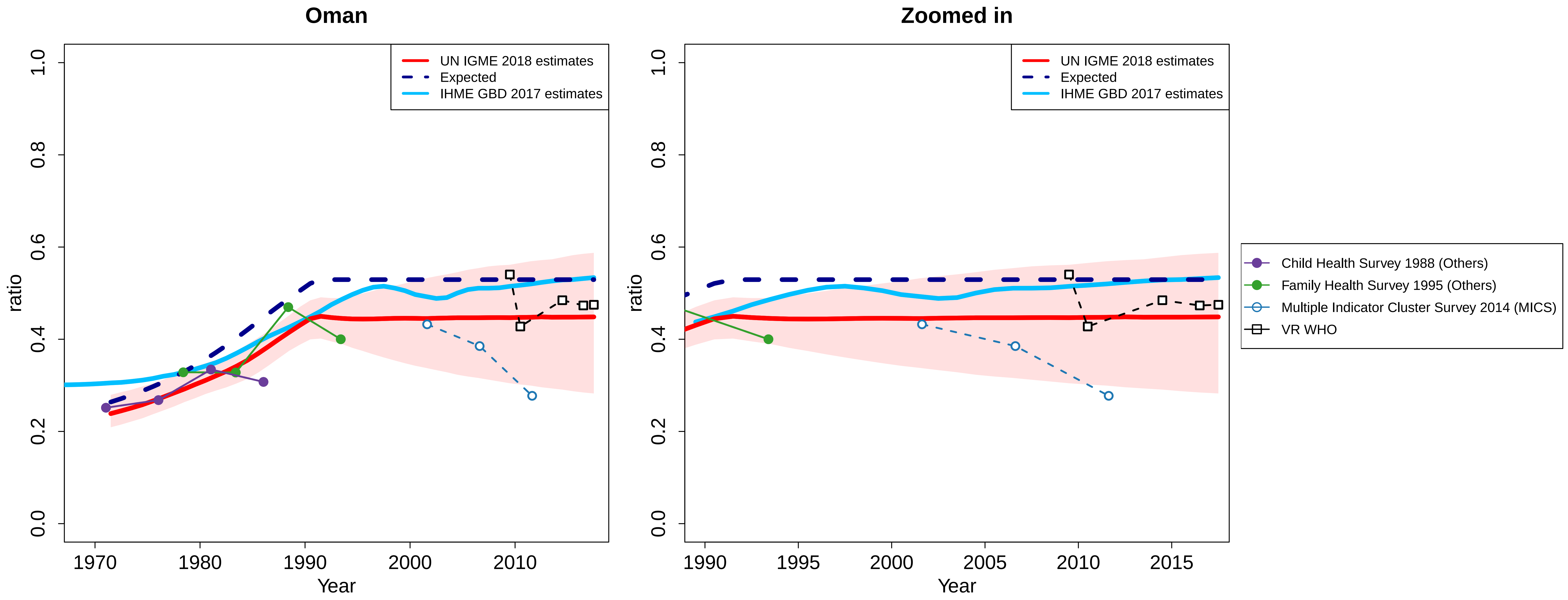
Oman
Zoomed in
UN IGME 2018 estimates
Expected
IHME GBD 2017 estimates
ratio
Year
Child Health Survey 1988 (Others)
Family Health Survey 1995 (Others)
Multiple Indicator Cluster Survey 2014 (MICS)
VR WHO

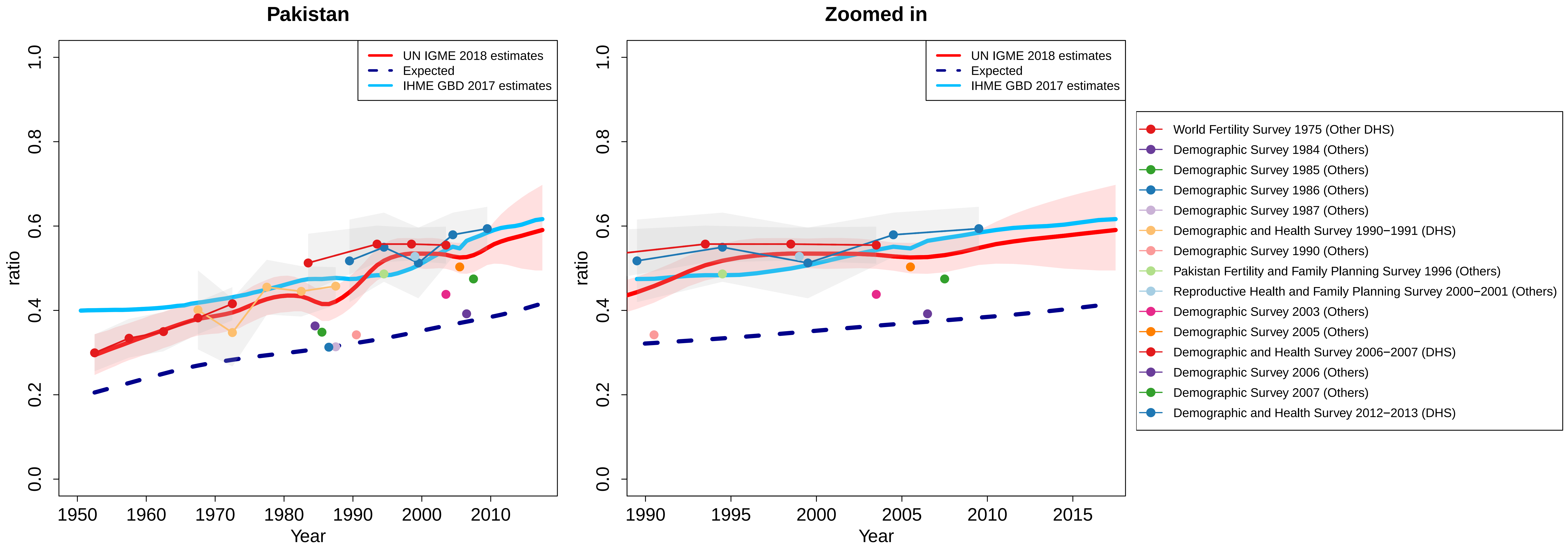

Pakistan
Zoomed in
ratio
Year
UN IGME 2018 estimates
Expected
IHME GBD 2017 estimates
World Fertility Survey 1975 (Other DHS)
Demographic Survey 1984 (Others)
Demographic Survey 1985 (Others)
Demographic Survey 1986 (Others)
Demographic Survey 1987 (Others)
Demographic and Health Survey 1990−1991 (DHS)
Demographic Survey 1990 (Others)
Pakistan Fertility and Family Planning Survey 1996 (Others)
Reproductive Health and Family Planning Survey 2000−2001 (Others)
Demographic Survey 2003 (Others)
Demographic Survey 2005 (Others)
Demographic and Health Survey 2006−2007 (DHS)
Demographic Survey 2006 (Others)
Demographic Survey 2007 (Others)
Demographic and Health Survey 2012−2013 (DHS)

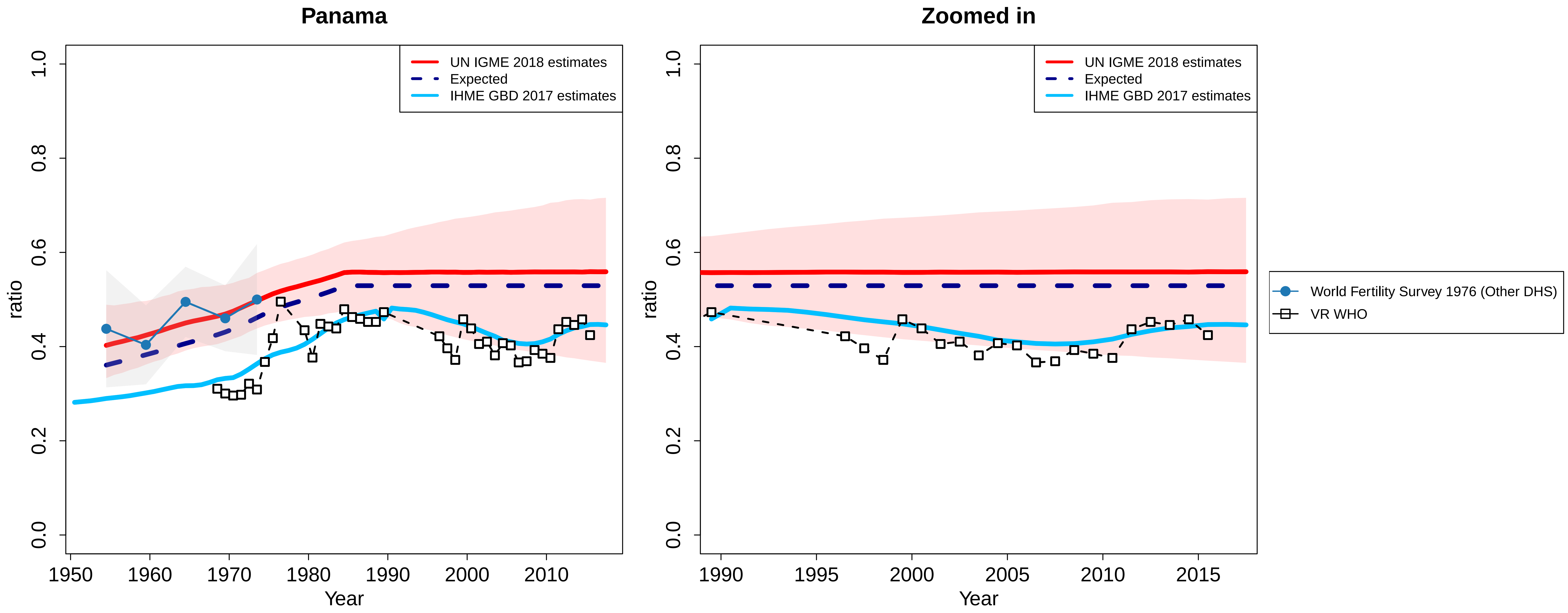

Panama
Zoomed in
ratio
Year
UN IGME 2018 estimates
Expected
IHME GBD 2017 estimates
World Fertility Survey 1976 (Other DHS)
VR WHO
0.0
0.2
0.4
0.6
0.8
1.0
1950
1960
1970
1980
1990
2000
2010
1995
2005
2015

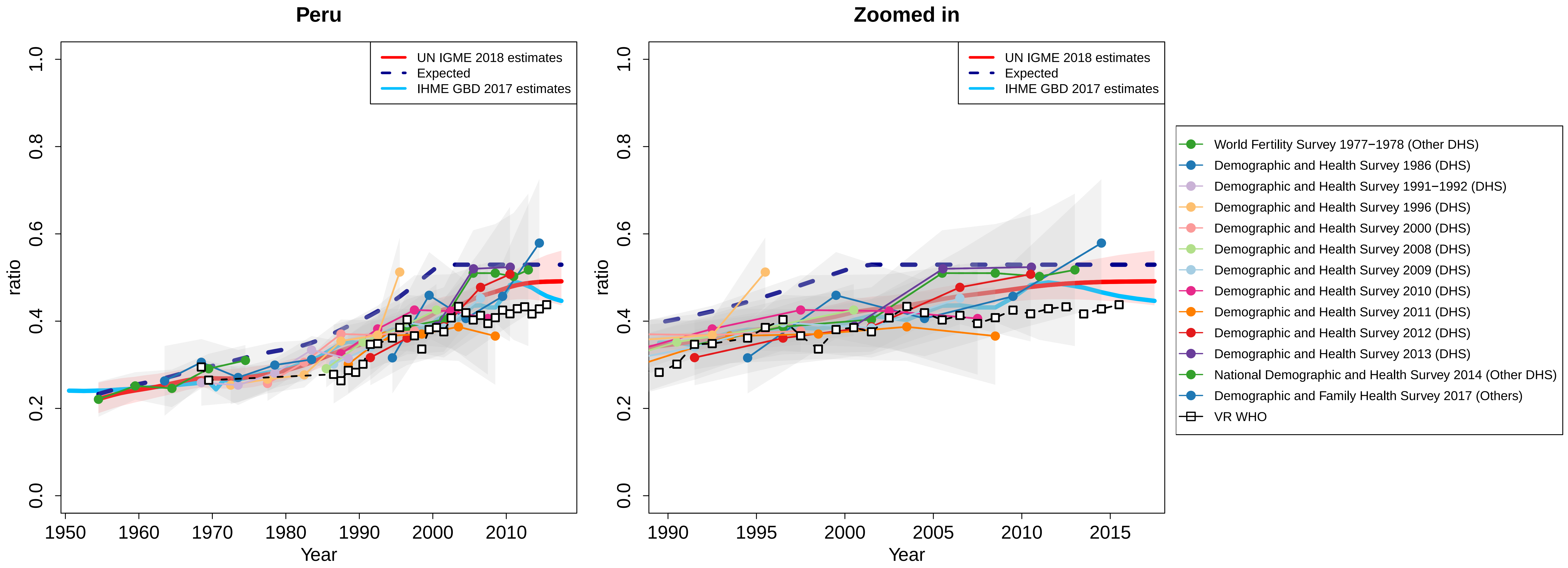

Peru
Zoomed in
UN IGME 2018 estimates
Expected
IHME GBD 2017 estimates
ratio
Year
World Fertility Survey 1977−1978 (Other DHS)
Demographic and Health Survey 1986 (DHS)
Demographic and Health Survey 1991−1992 (DHS)
Demographic and Health Survey 1996 (DHS)
Demographic and Health Survey 2000 (DHS)
Demographic and Health Survey 2008 (DHS)
Demographic and Health Survey 2009 (DHS)
Demographic and Health Survey 2010 (DHS)
Demographic and Health Survey 2011 (DHS)
Demographic and Health Survey 2012 (DHS)
Demographic and Health Survey 2013 (DHS)
National Demographic and Health Survey 2014 (Other DHS)
Demographic and Family Health Survey 2017 (Others)
VR WHO

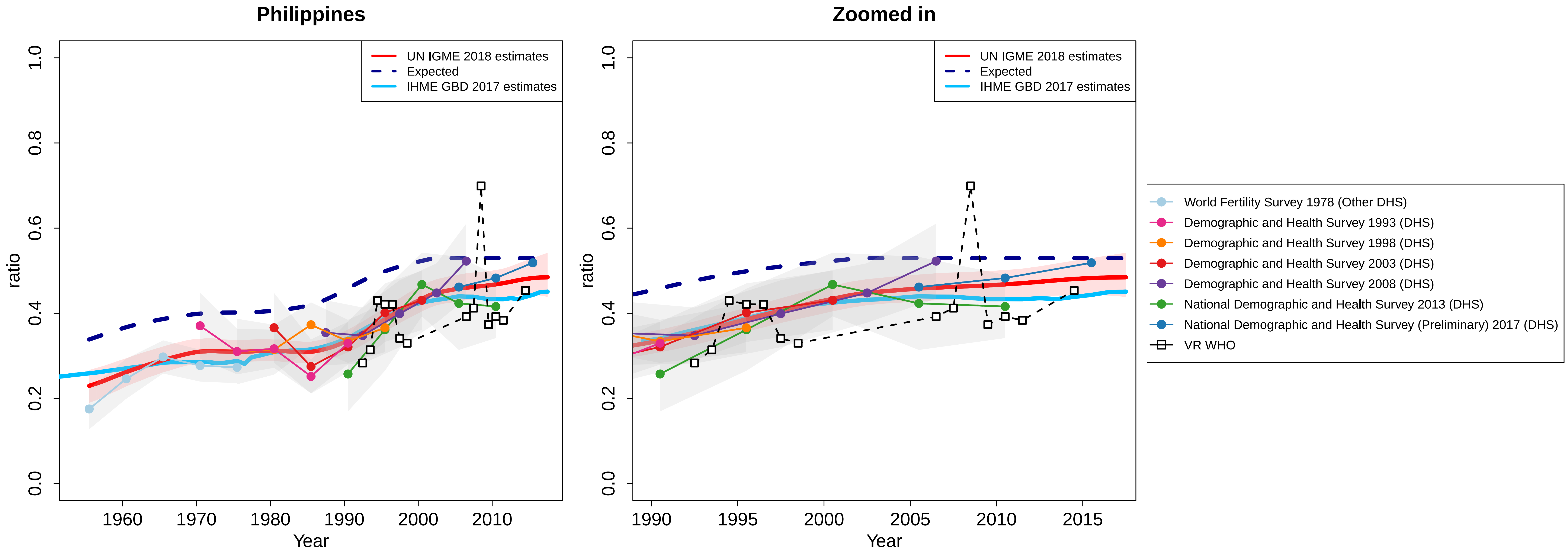

Philippines
UN IGME 2018 estimates
Expected
IHME GBD 2017 estimates
ratio
Year
Zoomed in
UN IGME 2018 estimates
Expected
IHME GBD 2017 estimates
ratio
Year
World Fertility Survey 1978 (Other DHS)
Demographic and Health Survey 1993 (DHS)
Demographic and Health Survey 1998 (DHS)
Demographic and Health Survey 2003 (DHS)
Demographic and Health Survey 2008 (DHS)
National Demographic and Health Survey 2013 (DHS)
National Demographic and Health Survey (Preliminary) 2017 (DHS)
VR WHO

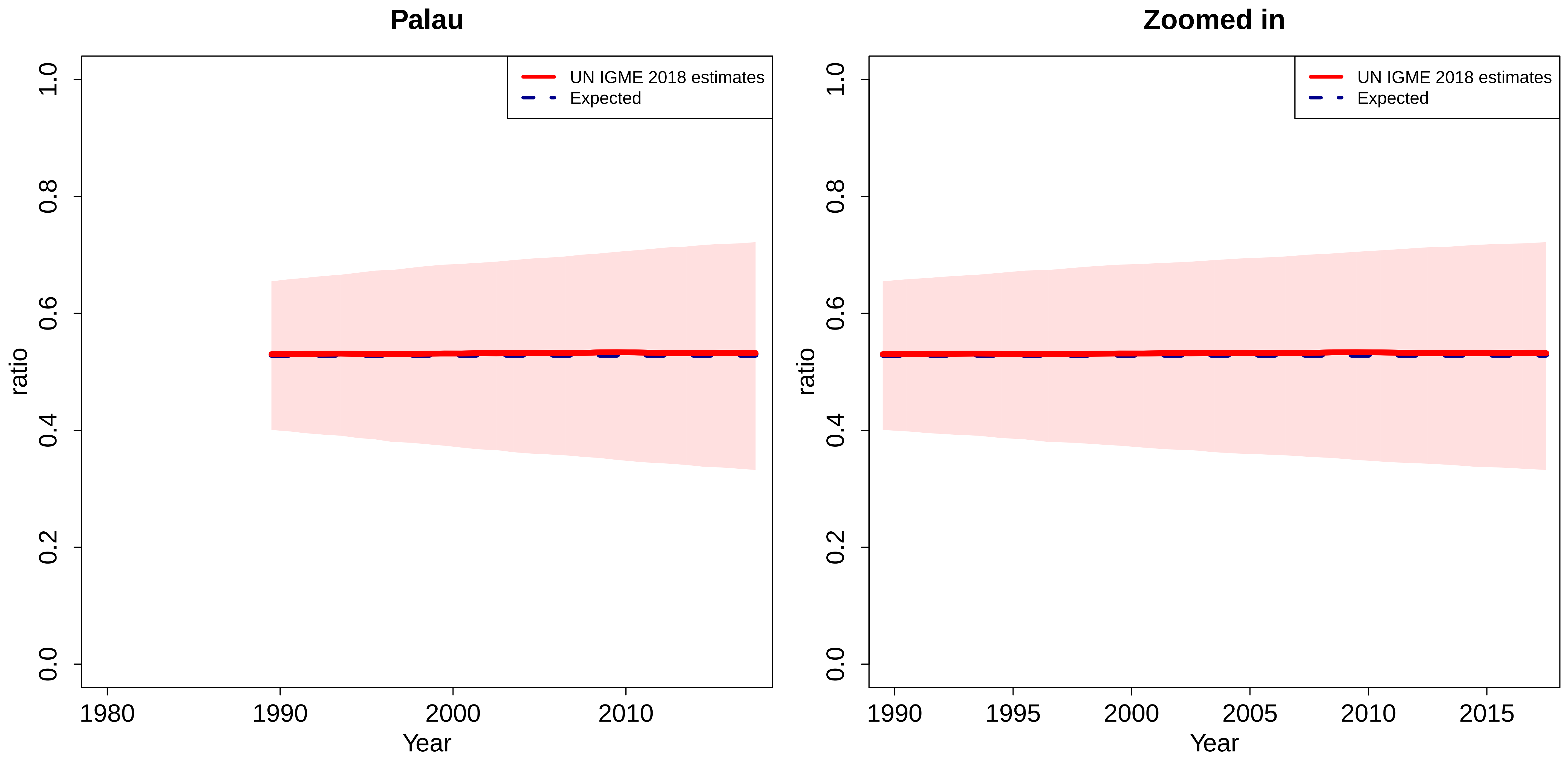

Palau
Zoomed in
UN IGME 2018 estimates
Expected
ratio
Year
0.0
0.2
0.4
0.6
0.8
1.0
1980
1990
2000
2010
1995
2005
2015

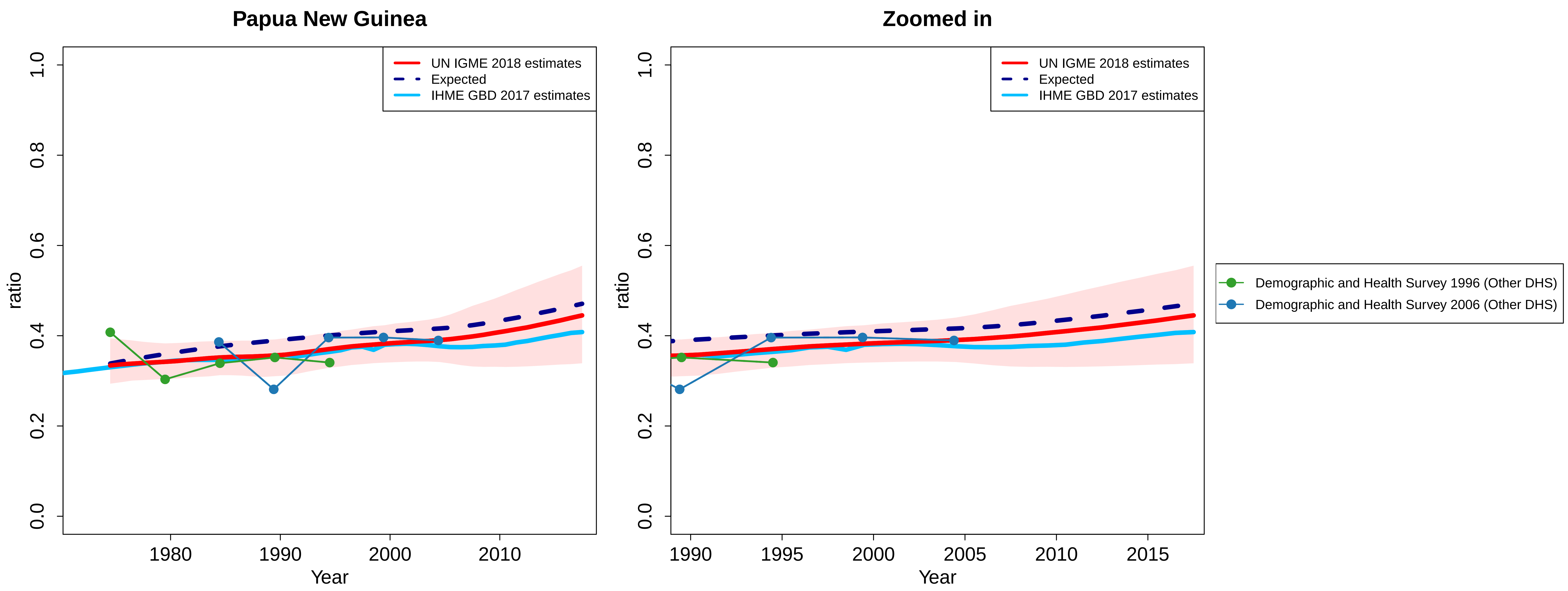
Papua New Guinea
Zoomed in
UN IGME 2018 estimates
Expected
IHME GBD 2017 estimates
ratio
Year
0.0
0.2
0.4
0.6
0.8
1.0
1980
1990
2000
2010
1995
2005
2015
Demographic and Health Survey 1996 (Other DHS)
Demographic and Health Survey 2006 (Other DHS)

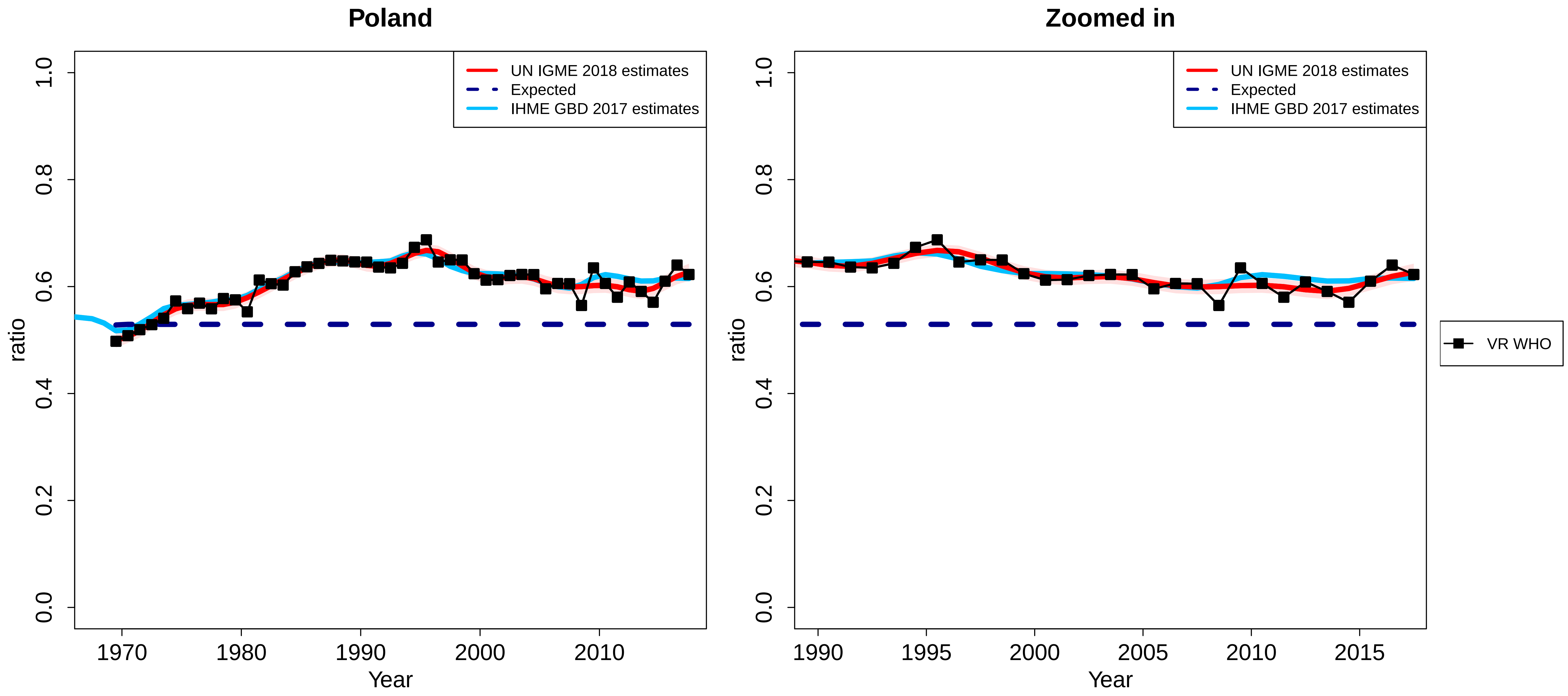
Poland
Zoomed in
UN IGME 2018 estimates
Expected
IHME GBD 2017 estimates
VR WHO
ratio
Year
0.0
0.2
0.4
0.6
0.8
1.0
1970
1980
1990
2000
2010
1995
2005
2015

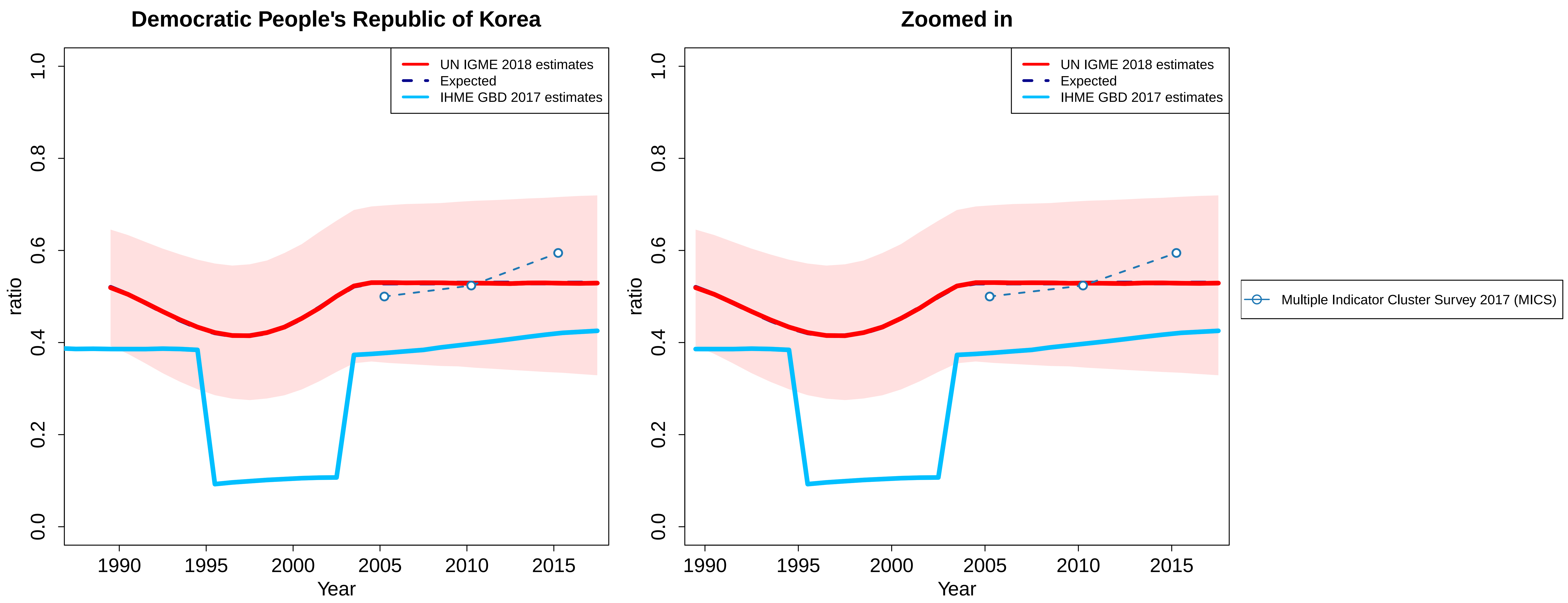
Democratic People's Republic of Korea
Zoomed in
UN IGME 2018 estimates
Expected
IHME GBD 2017 estimates
Multiple Indicator Cluster Survey 2017 (MICS)
ratio
Year
0.0
0.2
0.4
0.6
0.8
1.0
1990
1995
2000
2005
2010
2015

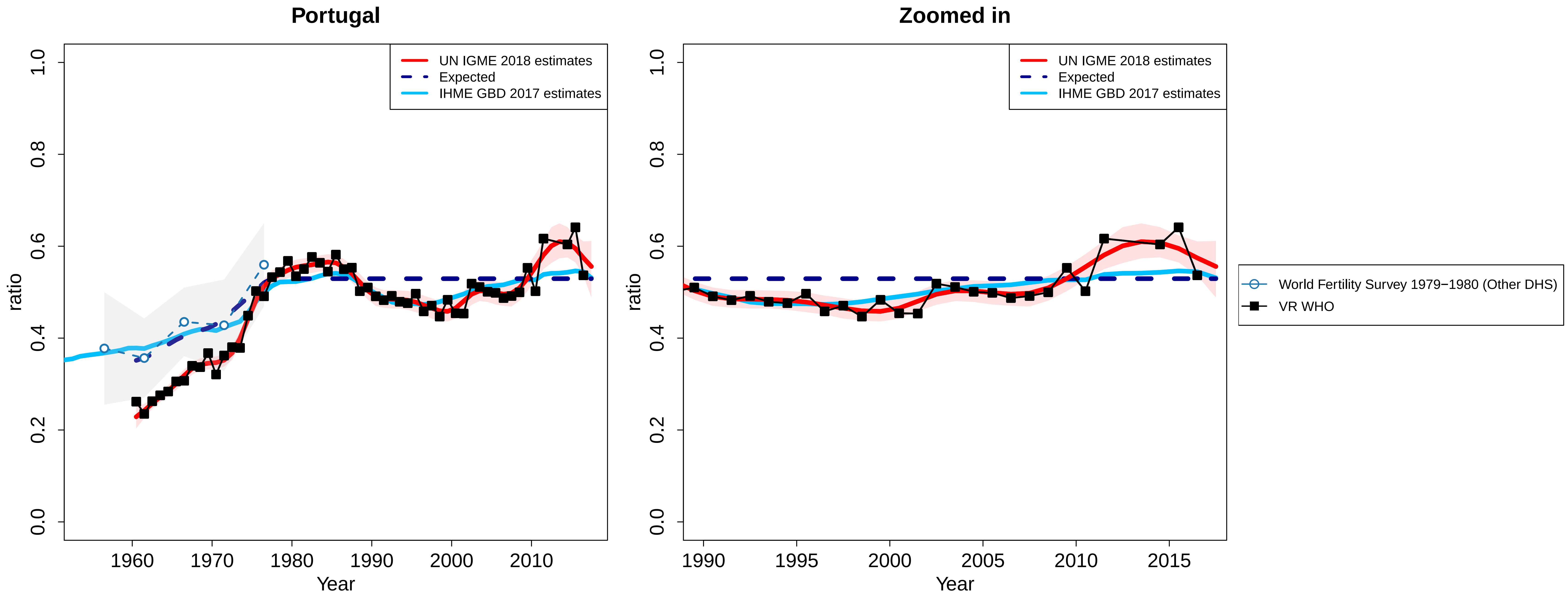
Portugal
Zoomed in
UN IGME 2018 estimates
Expected
IHME GBD 2017 estimates
World Fertility Survey 1979−1980 (Other DHS)
VR WHO
ratio
Year
0.0
0.2
0.4
0.6
0.8
1.0
1960
1970
1980
1990
2000
2010
1990
1995
2000
2005
2010
2015

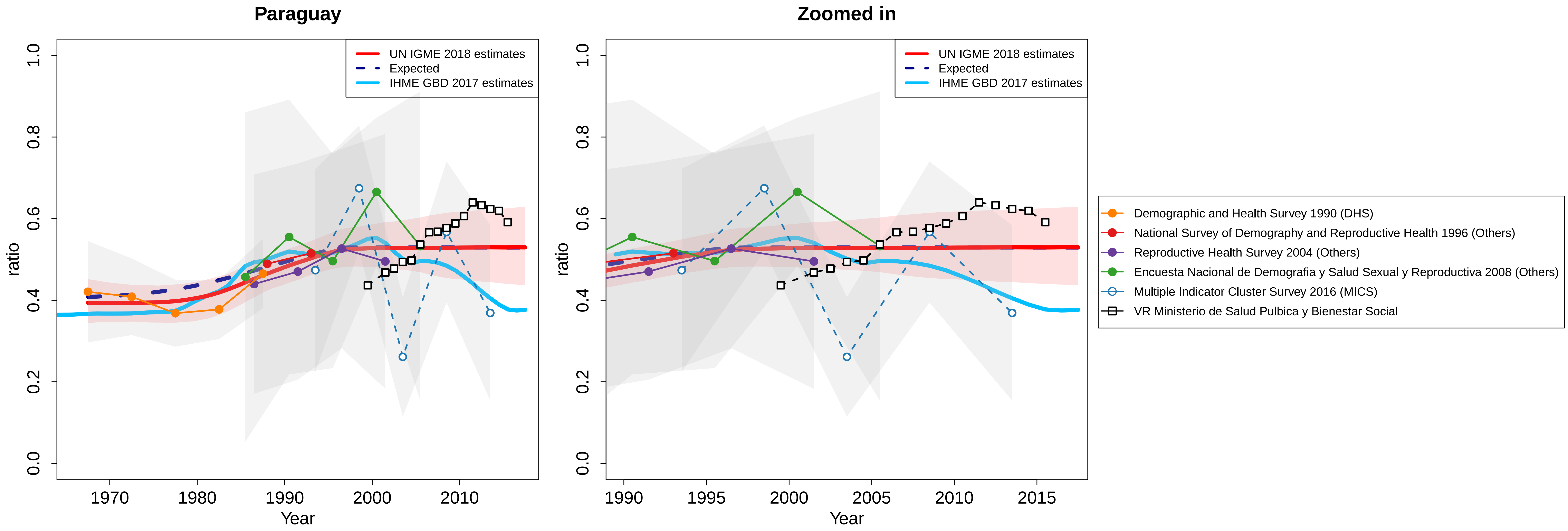
Paraguay
Zoomed in
ratio
Year
0.0
0.2
0.4
0.6
0.8
1.0
1970
1980
1990
2000
2010
1995
2005
2015
UN IGME 2018 estimates
Expected
IHME GBD 2017 estimates
Demographic and Health Survey 1990 (DHS)
National Survey of Demography and Reproductive Health 1996 (Others)
Reproductive Health Survey 2004 (Others)
Encuesta Nacional de Demografia y Salud Sexual y Reproductiva 2008 (Others)
Multiple Indicator Cluster Survey 2016 (MICS)
VR Ministerio de Salud Pulbica y Bienestar Social

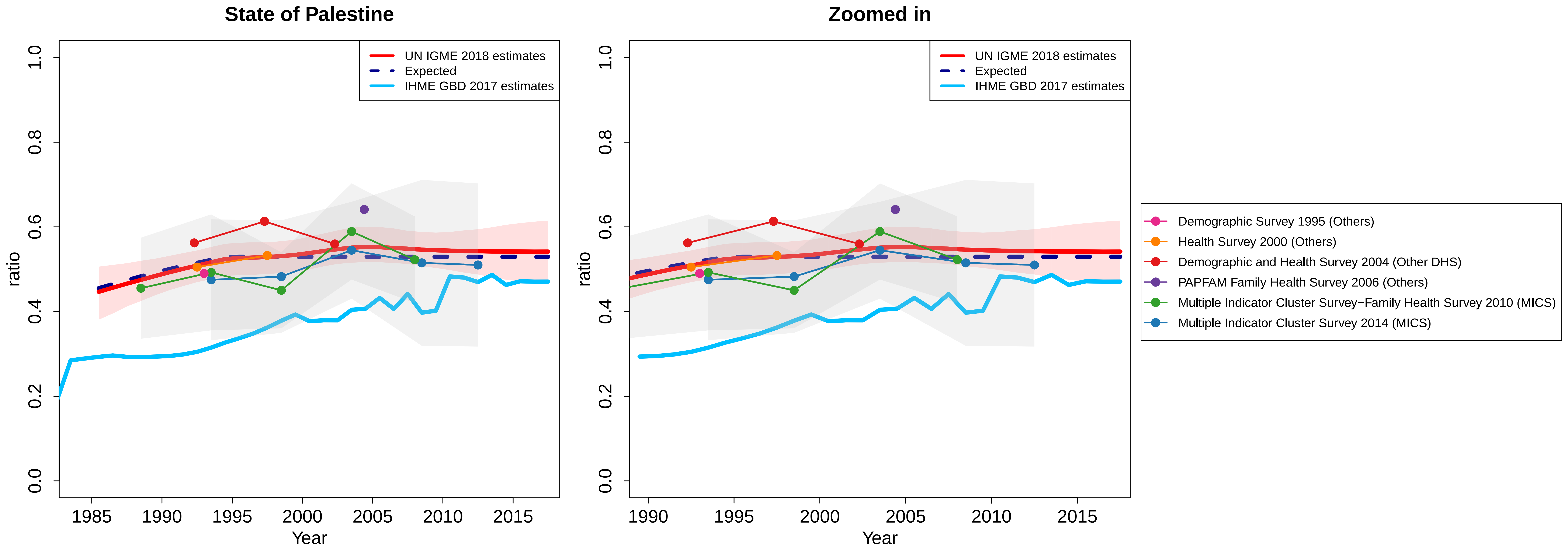
State of Palestine
Zoomed in
UN IGME 2018 estimates
Expected
IHME GBD 2017 estimates
ratio
Year
0.0
0.2
0.4
0.6
0.8
1.0
1985
1990
1995
2000
2005
2010
2015
Demographic Survey 1995 (Others)
Health Survey 2000 (Others)
Demographic and Health Survey 2004 (Other DHS)
PAPFAM Family Health Survey 2006 (Others)
Multiple Indicator Cluster Survey−Family Health Survey 2010 (MICS)
Multiple Indicator Cluster Survey 2014 (MICS)

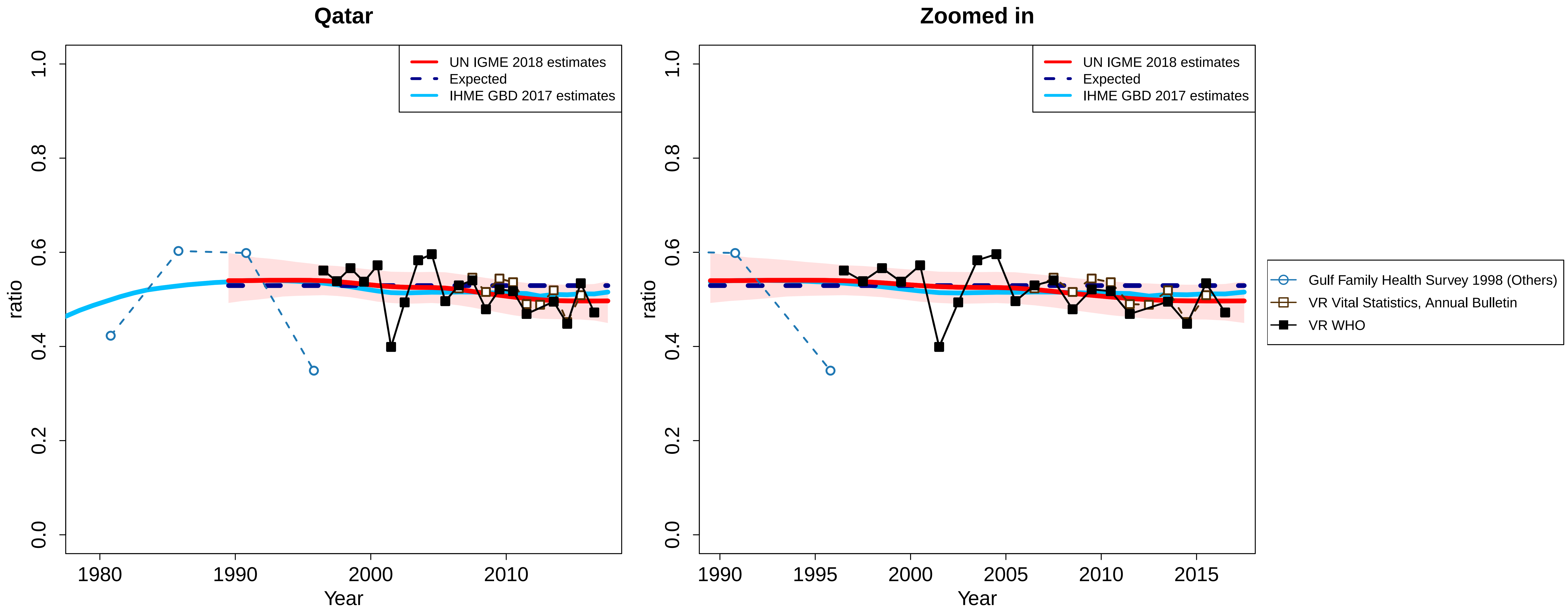

Qatar
Zoomed in
UN IGME 2018 estimates
Expected
IHME GBD 2017 estimates
ratio
Year
Gulf Family Health Survey 1998 (Others)
VR Vital Statistics, Annual Bulletin
VR WHO

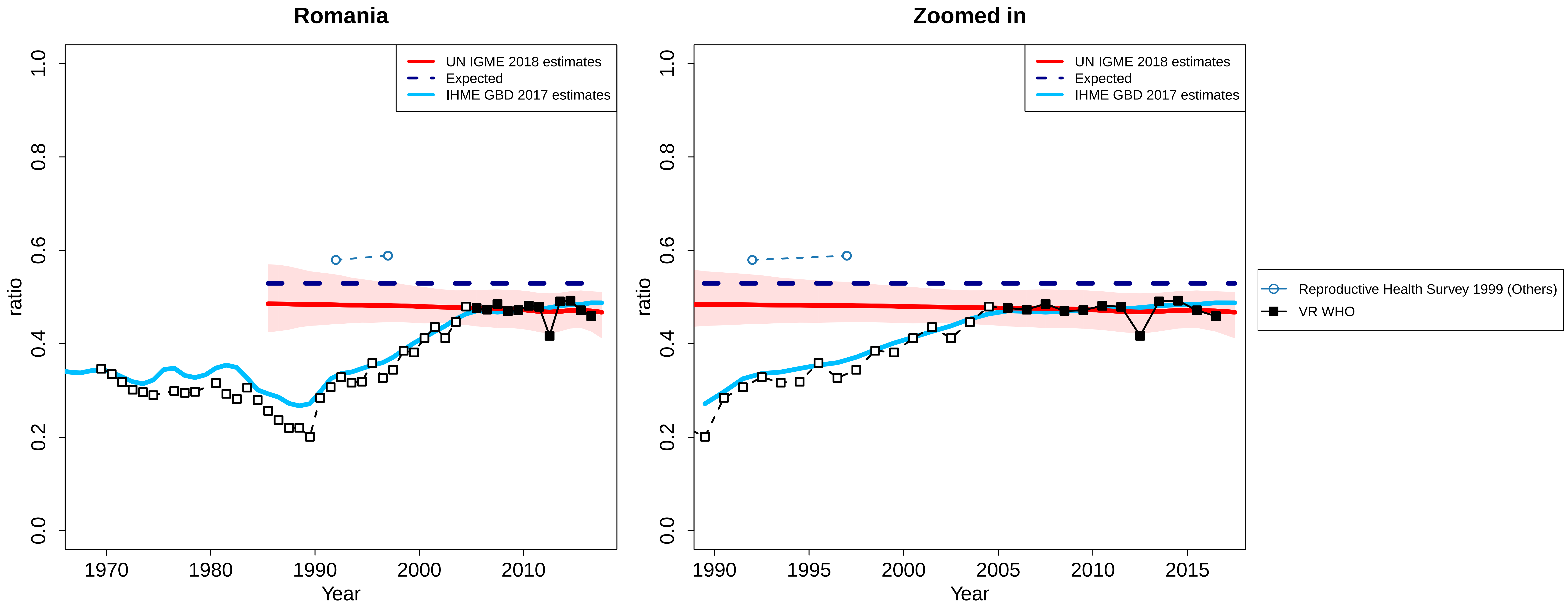

Romania
Zoomed in
UN IGME 2018 estimates
Expected
IHME GBD 2017 estimates
ratio
Year
0.0
0.2
0.4
0.6
0.8
1.0
1970
1980
1990
2000
2010
1995
2005
2015
Reproductive Health Survey 1999 (Others)
VR WHO

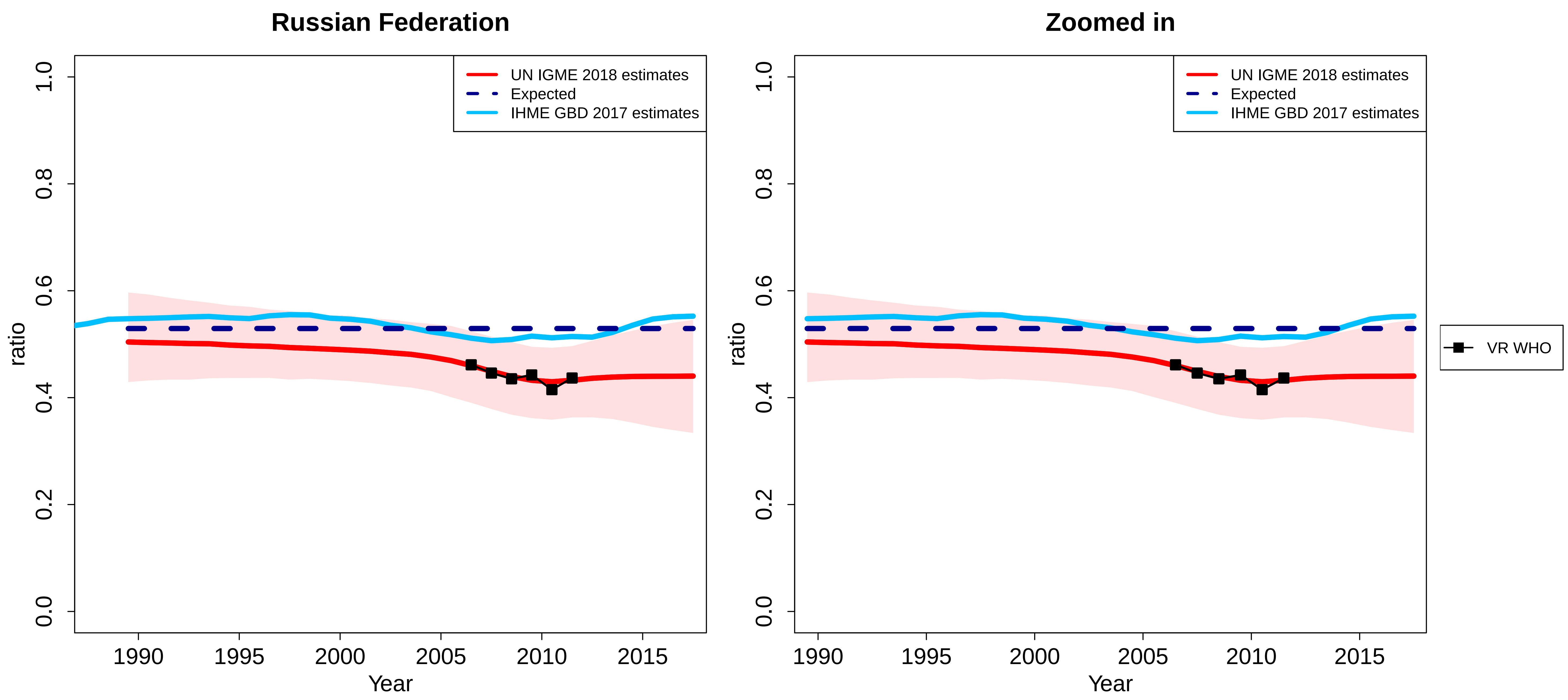
Russian Federation
Zoomed in
UN IGME 2018 estimates
Expected
IHME GBD 2017 estimates
VR WHO
ratio
Year
0.0
0.2
0.4
0.6
0.8
1.0
1990
1995
2000
2005
2010
2015

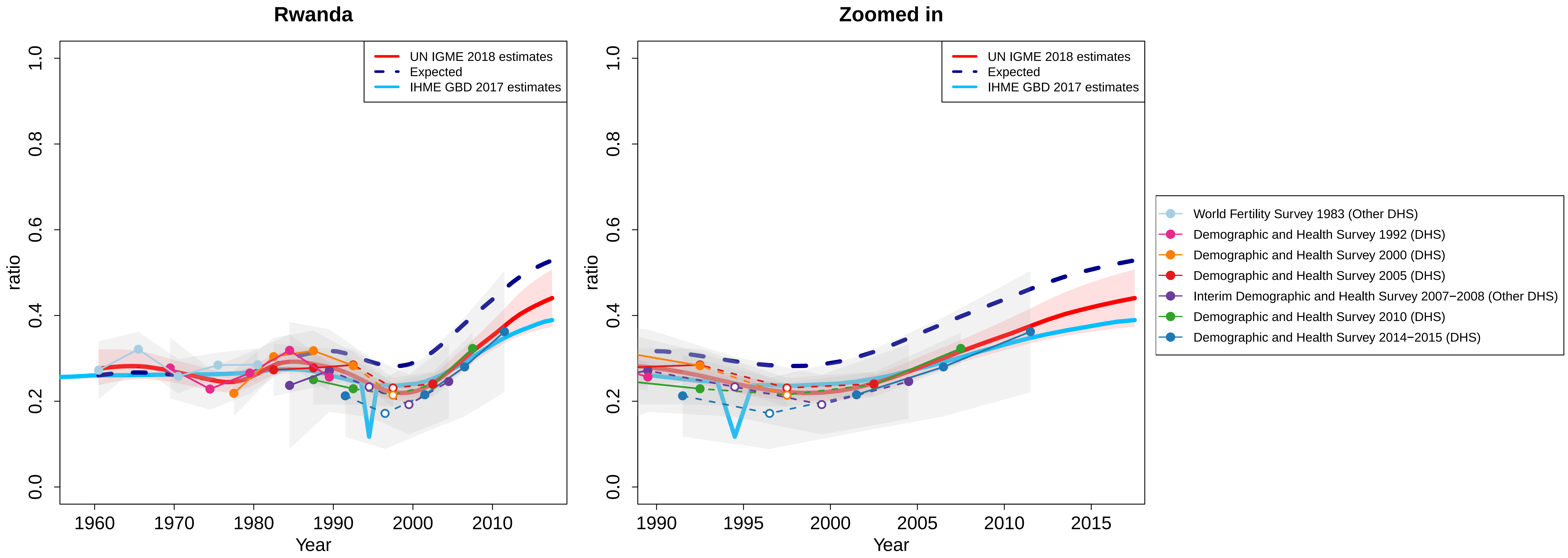
Rwanda
Zoomed in
UN IGME 2018 estimates
Expected
IHME GBD 2017 estimates
ratio
Year
1960
1970
1980
1990
2000
2010
1995
2005
2015
0.0
0.2
0.4
0.6
0.8
1.0
World Fertility Survey 1983 (Other DHS)
Demographic and Health Survey 1992 (DHS)
Demographic and Health Survey 2000 (DHS)
Demographic and Health Survey 2005 (DHS)
Interim Demographic and Health Survey 2007−2008 (Other DHS)
Demographic and Health Survey 2010 (DHS)
Demographic and Health Survey 2014−2015 (DHS)

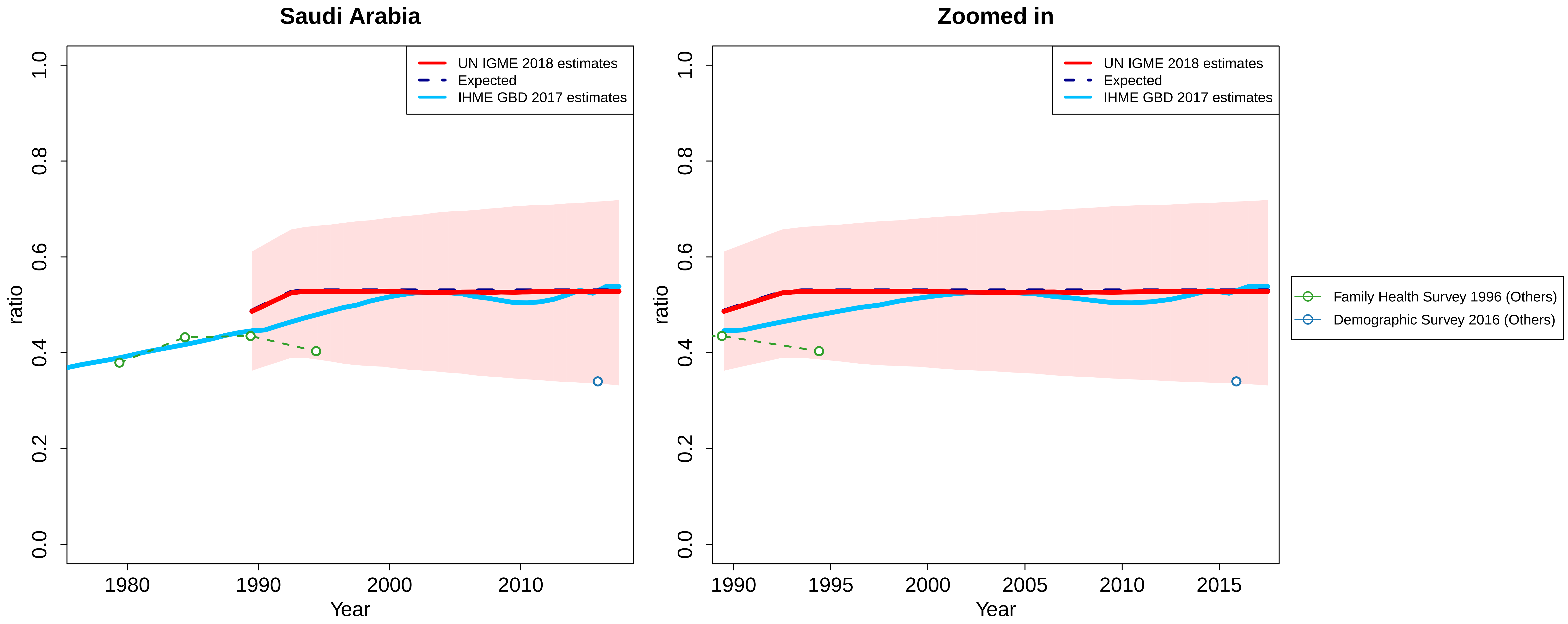

Saudi Arabia
Zoomed in
UN IGME 2018 estimates
Expected
IHME GBD 2017 estimates
Family Health Survey 1996 (Others)
Demographic Survey 2016 (Others)
ratio
Year
0.0
0.2
0.4
0.6
0.8
1.0
1980
1990
2000
2010
1995
2005
2015

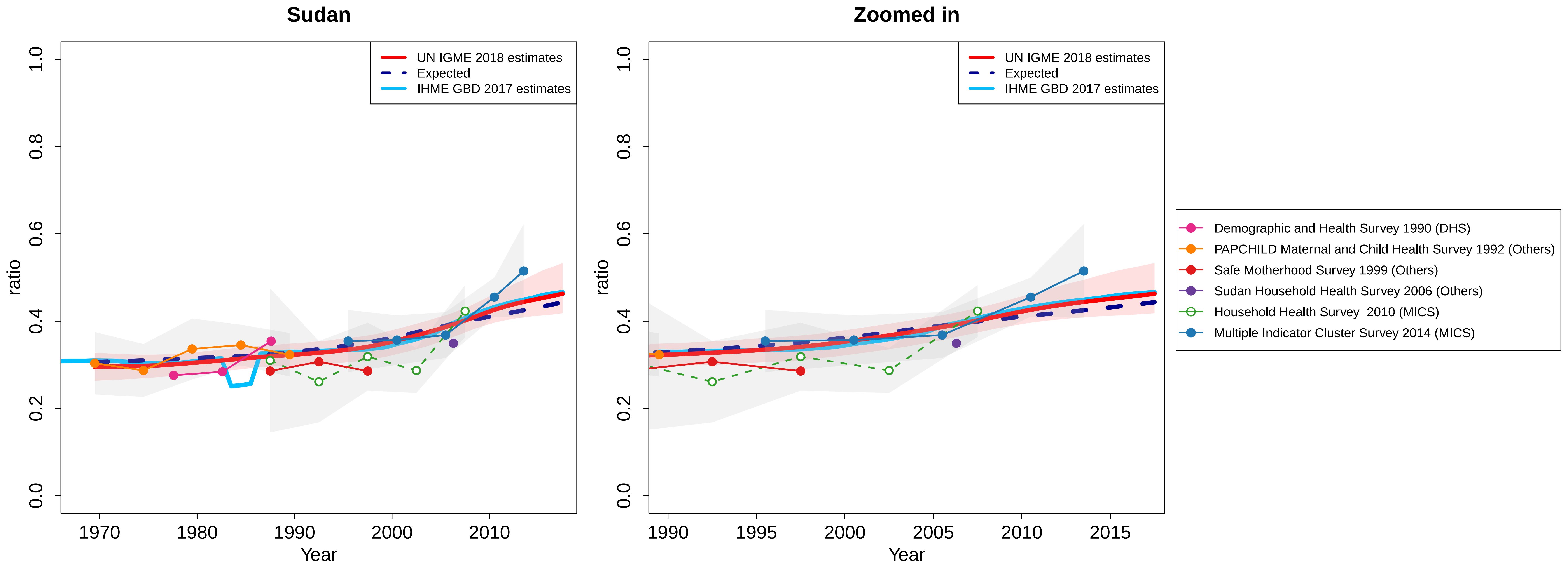

Sudan
Zoomed in
UN IGME 2018 estimates
Expected
IHME GBD 2017 estimates
ratio
Year
0.0
0.2
0.4
0.6
0.8
1.0
1970
1980
1990
2000
2010
1995
2005
2015
Demographic and Health Survey 1990 (DHS)
PAPCHILD Maternal and Child Health Survey 1992 (Others)
Safe Motherhood Survey 1999 (Others)
Sudan Household Health Survey 2006 (Others)
Household Health Survey 2010 (MICS)
Multiple Indicator Cluster Survey 2014 (MICS)

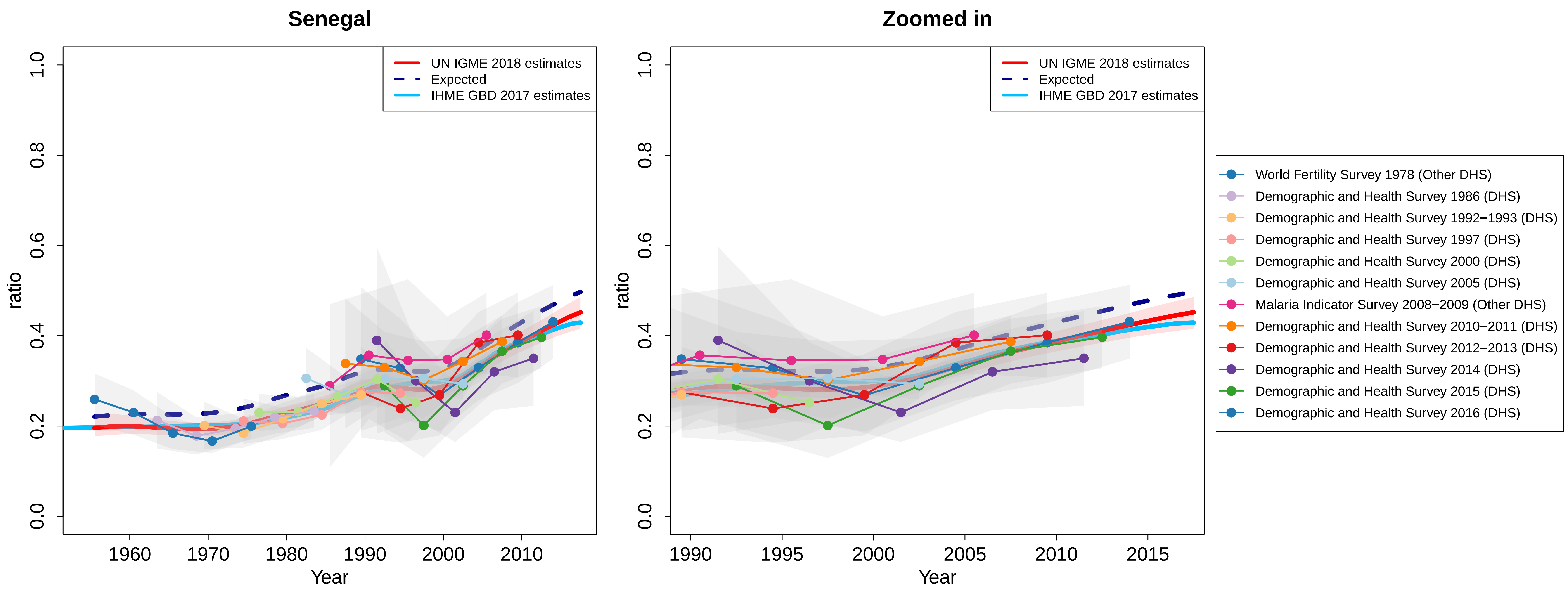

Senegal
Zoomed in
ratio
Year
UN IGME 2018 estimates
Expected
IHME GBD 2017 estimates
World Fertility Survey 1978 (Other DHS)
Demographic and Health Survey 1986 (DHS)
Demographic and Health Survey 1992−1993 (DHS)
Demographic and Health Survey 1997 (DHS)
Demographic and Health Survey 2000 (DHS)
Demographic and Health Survey 2005 (DHS)
Malaria Indicator Survey 2008−2009 (Other DHS)
Demographic and Health Survey 2010−2011 (DHS)
Demographic and Health Survey 2012−2013 (DHS)
Demographic and Health Survey 2014 (DHS)
Demographic and Health Survey 2015 (DHS)
Demographic and Health Survey 2016 (DHS)

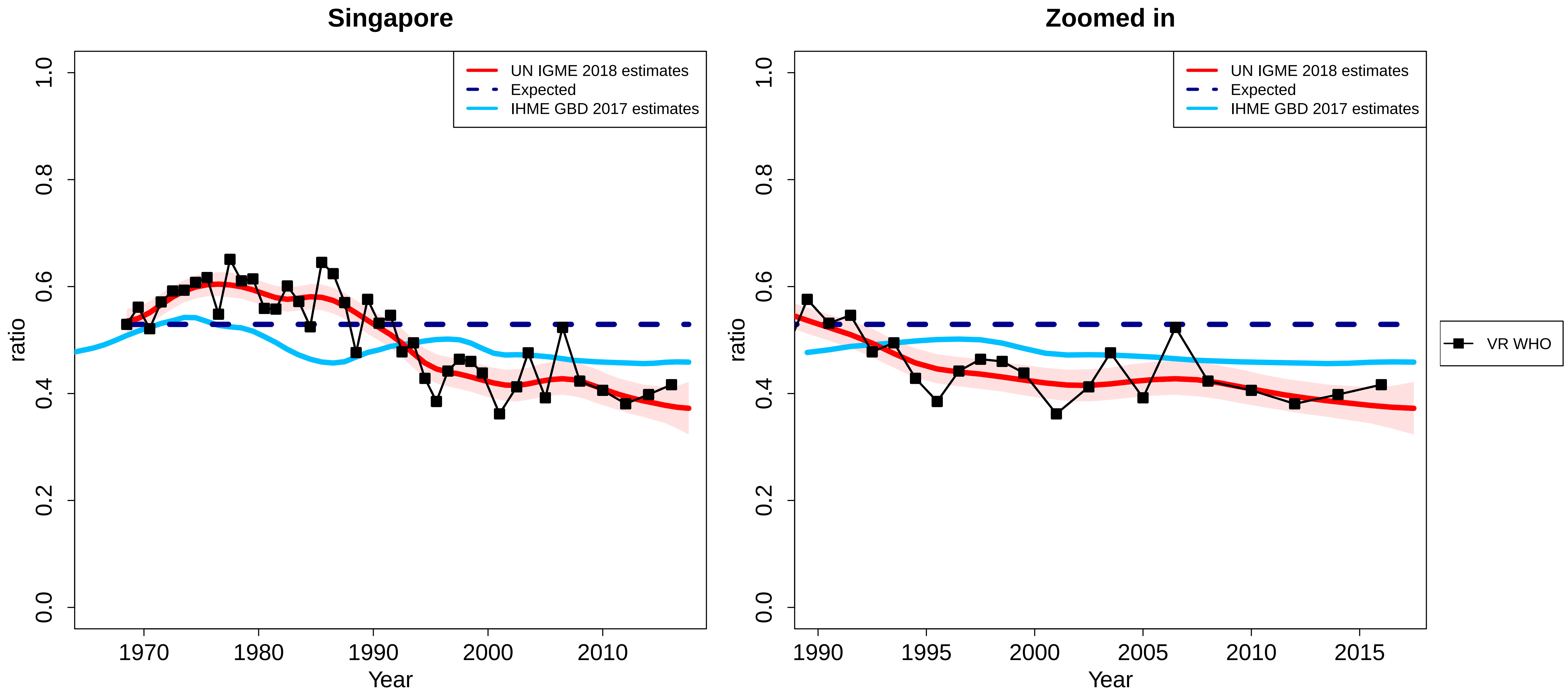

Singapore
Zoomed in
UN IGME 2018 estimates
Expected
IHME GBD 2017 estimates
ratio
Year
0.0
0.2
0.4
0.6
0.8
1.0
1970
1980
1990
2000
2010
1995
2005
2015
VR WHO

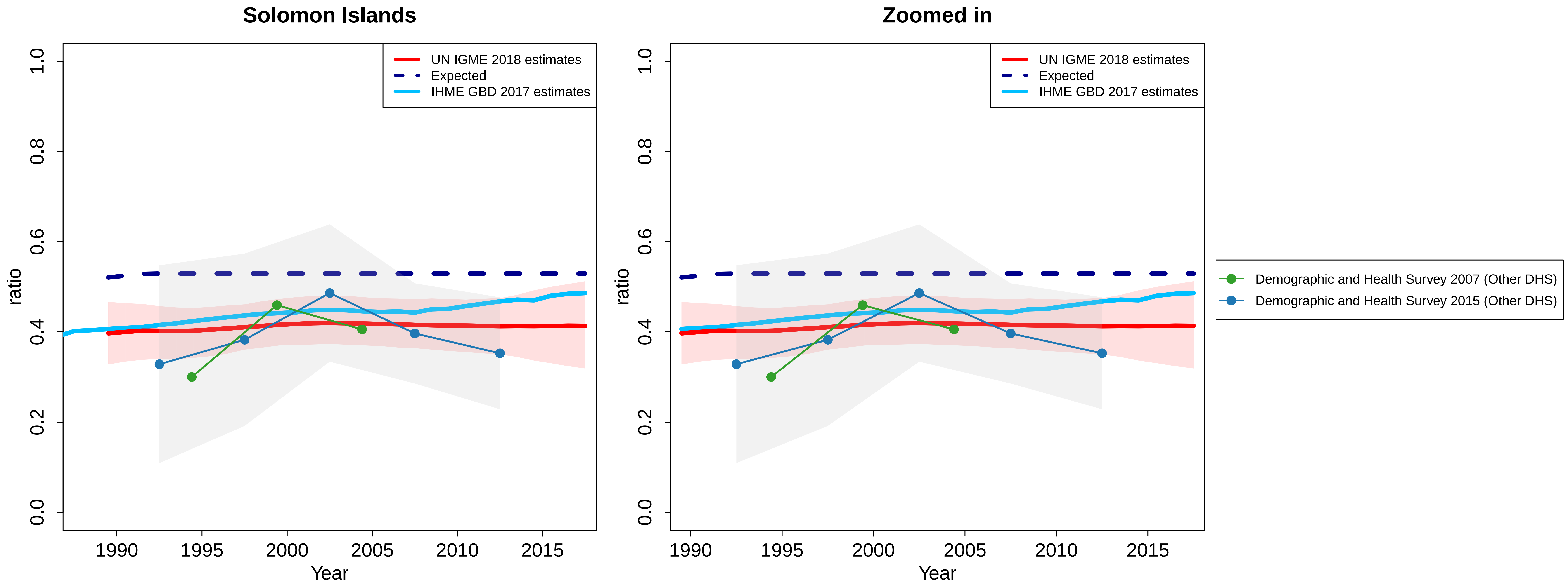
Solomon Islands
Zoomed in
UN IGME 2018 estimates
Expected
IHME GBD 2017 estimates
ratio
Year
0.0
0.2
0.4
0.6
0.8
1.0
1990
1995
2000
2005
2010
2015
Demographic and Health Survey 2007 (Other DHS)
Demographic and Health Survey 2015 (Other DHS)

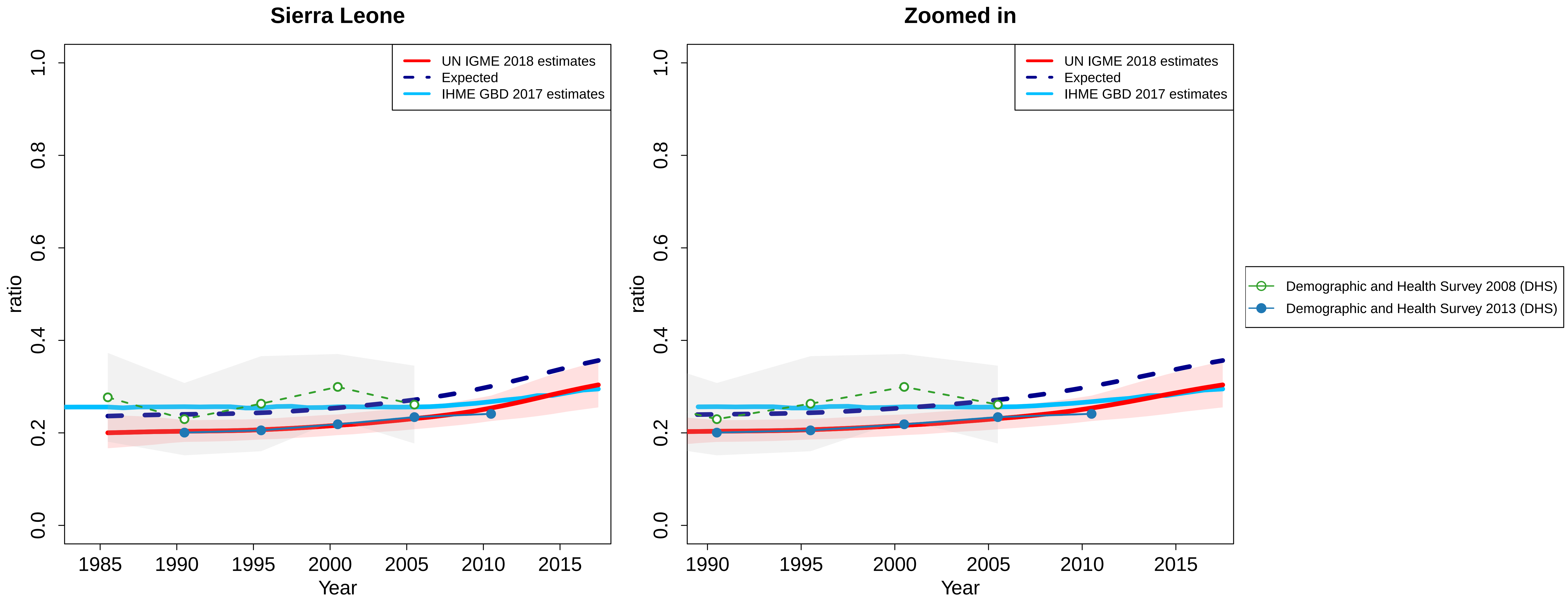

Sierra Leone
Zoomed in
UN IGME 2018 estimates
Expected
IHME GBD 2017 estimates
ratio
Year
1.0
0.8
0.6
0.4
0.2
0.0
1985
1990
1995
2000
2005
2010
2015
Demographic and Health Survey 2008 (DHS)
Demographic and Health Survey 2013 (DHS)

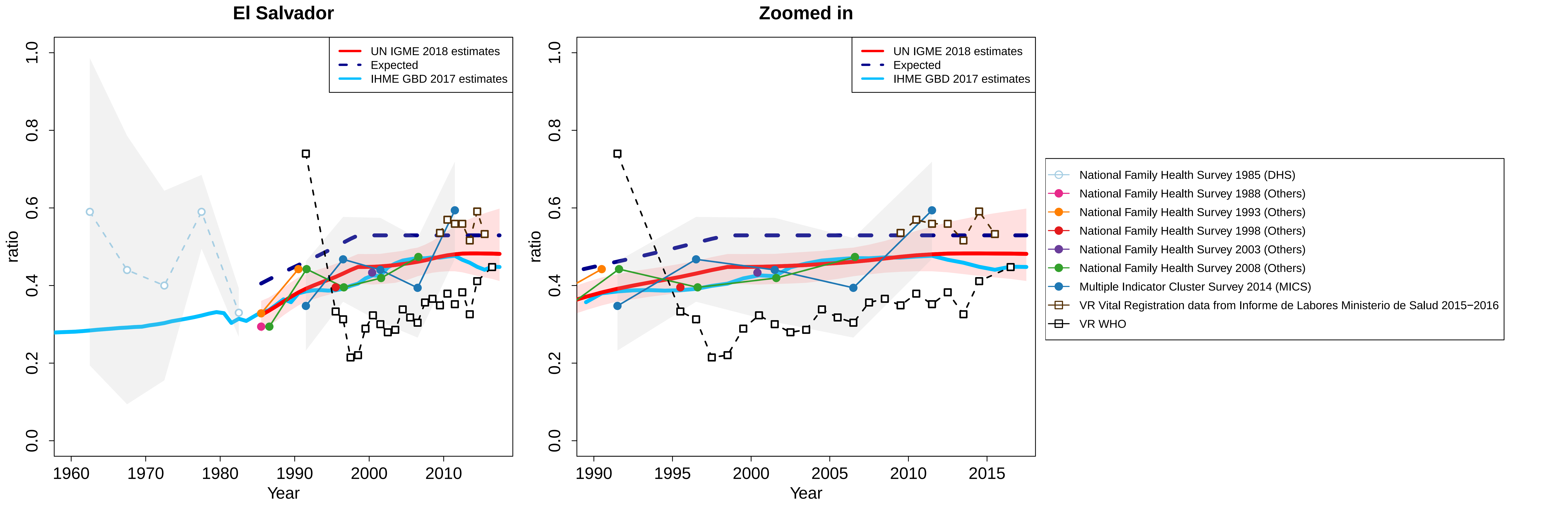
El Salvador
Zoomed in
UN IGME 2018 estimates
Expected
IHME GBD 2017 estimates
ratio
Year
0.0
0.2
0.4
0.6
0.8
1.0
1960
1970
1980
1990
2000
2010
1995
2005
2015
National Family Health Survey 1985 (DHS)
National Family Health Survey 1988 (Others)
National Family Health Survey 1993 (Others)
National Family Health Survey 1998 (Others)
National Family Health Survey 2003 (Others)
National Family Health Survey 2008 (Others)
Multiple Indicator Cluster Survey 2014 (MICS)
VR Vital Registration data from Informe de Labores Ministerio de Salud 2015−2016
VR WHO

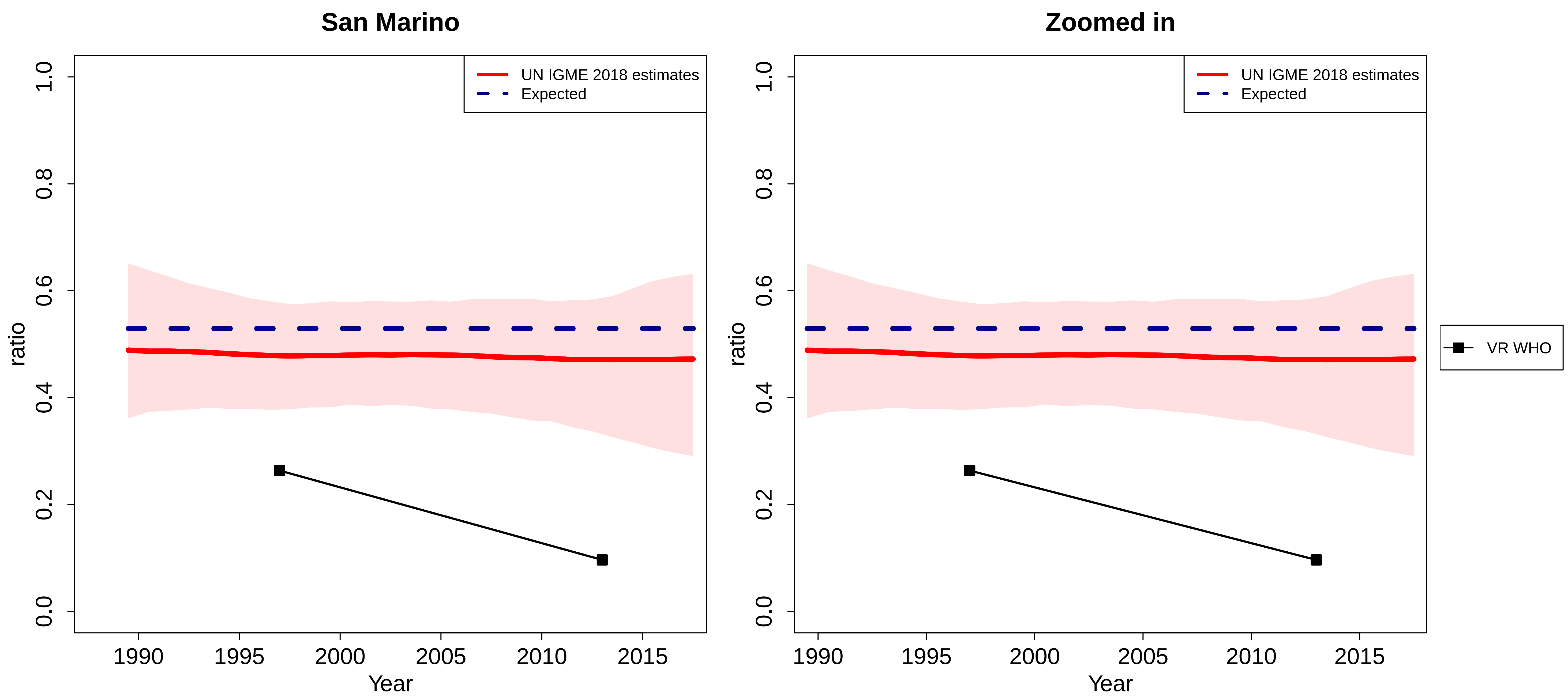

San Marino
Zoomed in
UN IGME 2018 estimates
Expected
VR WHO
ratio
Year
0.0
0.2
0.4
0.6
0.8
1.0
1990
1995
2000
2005
2010
2015

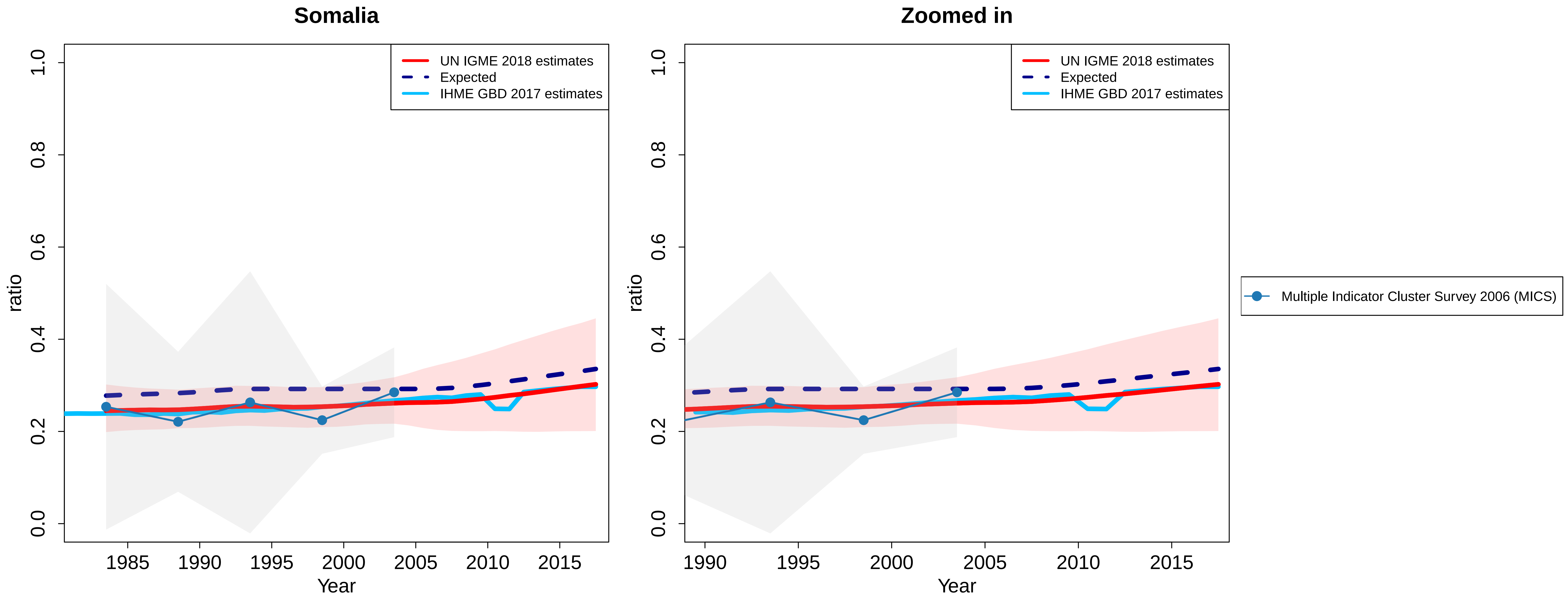

Somalia
Zoomed in
UN IGME 2018 estimates
Expected
IHME GBD 2017 estimates
Multiple Indicator Cluster Survey 2006 (MICS)
ratio
Year
0.0
0.2
0.4
0.6
0.8
1.0
1985
1990
1995
2000
2005
2010
2015

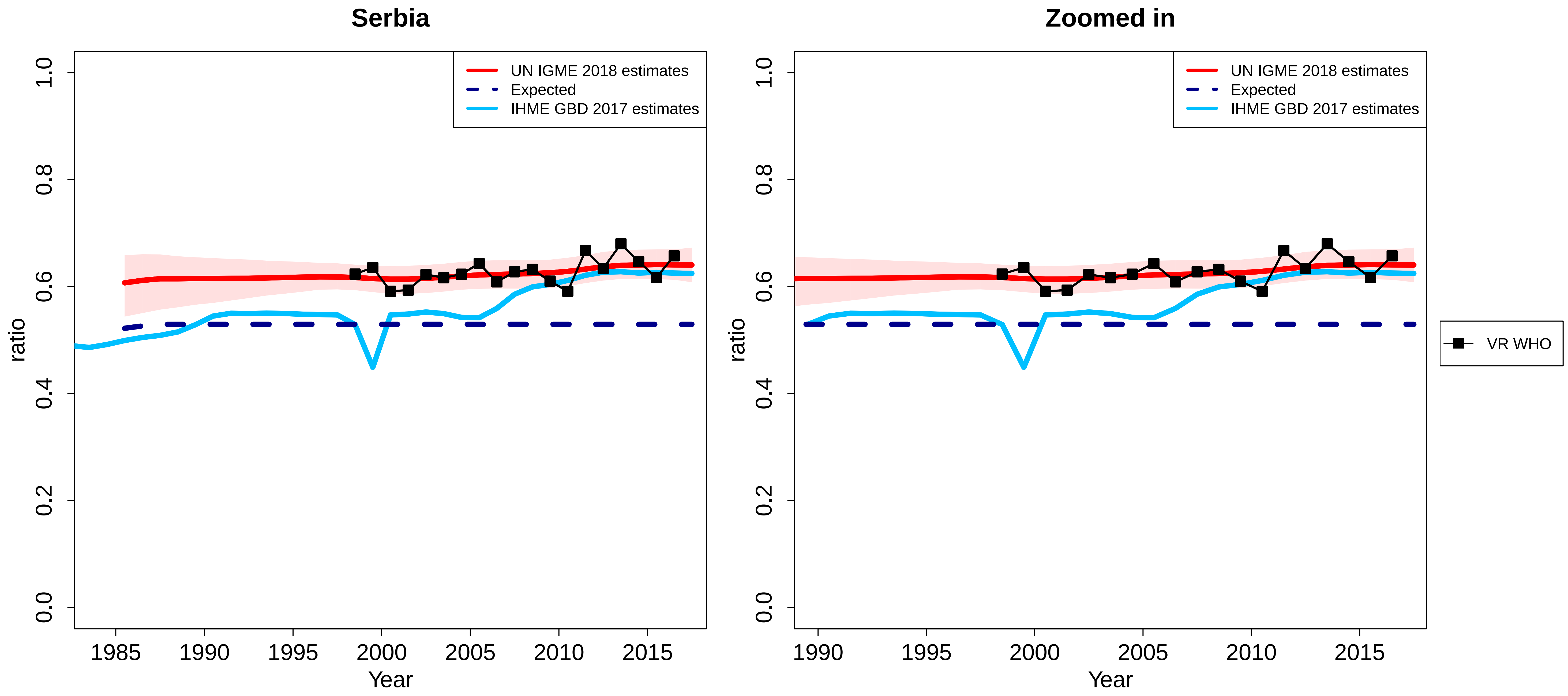

Serbia
Zoomed in
UN IGME 2018 estimates
Expected
IHME GBD 2017 estimates
VR WHO
ratio
Year
0.0
0.2
0.4
0.6
0.8
1.0
1985
1990
1995
2000
2005
2010
2015

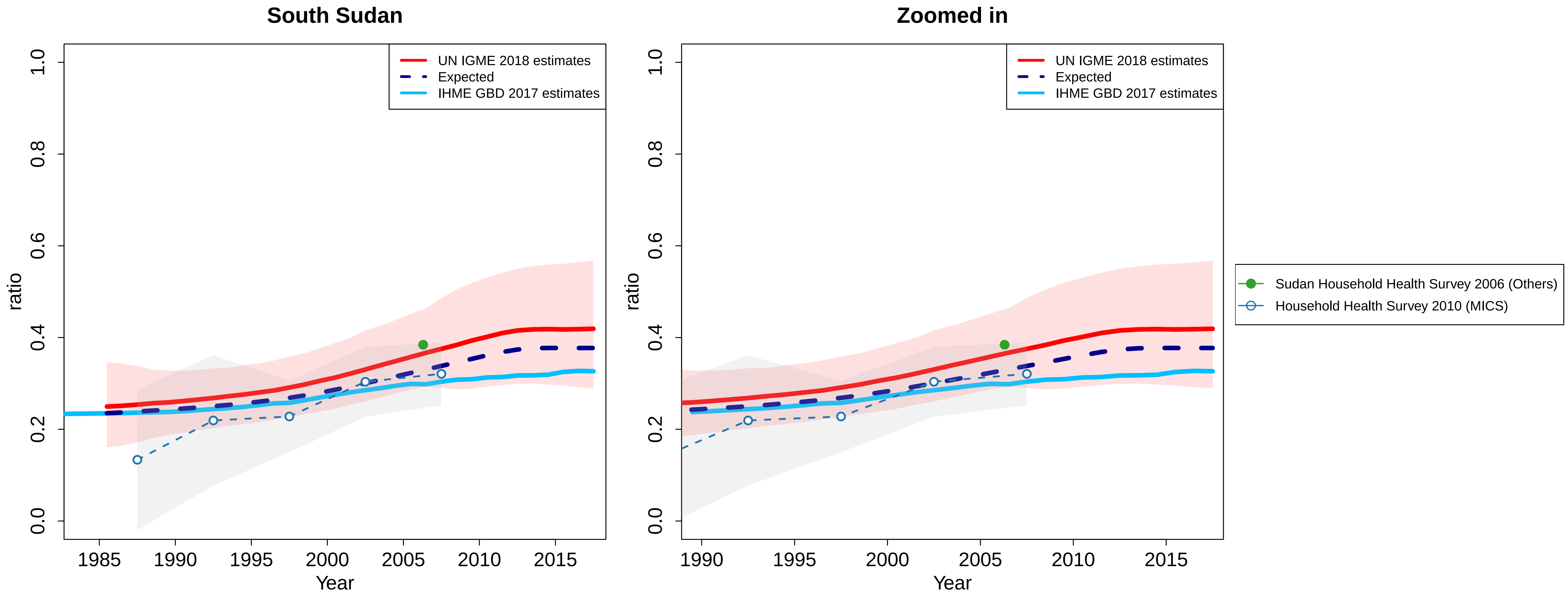

South Sudan
Zoomed in
UN IGME 2018 estimates
Expected
IHME GBD 2017 estimates
ratio
Year
0.0
0.2
0.4
0.6
0.8
1.0
1985
1990
1995
2000
2005
2010
2015
Sudan Household Health Survey 2006 (Others)
Household Health Survey 2010 (MICS)

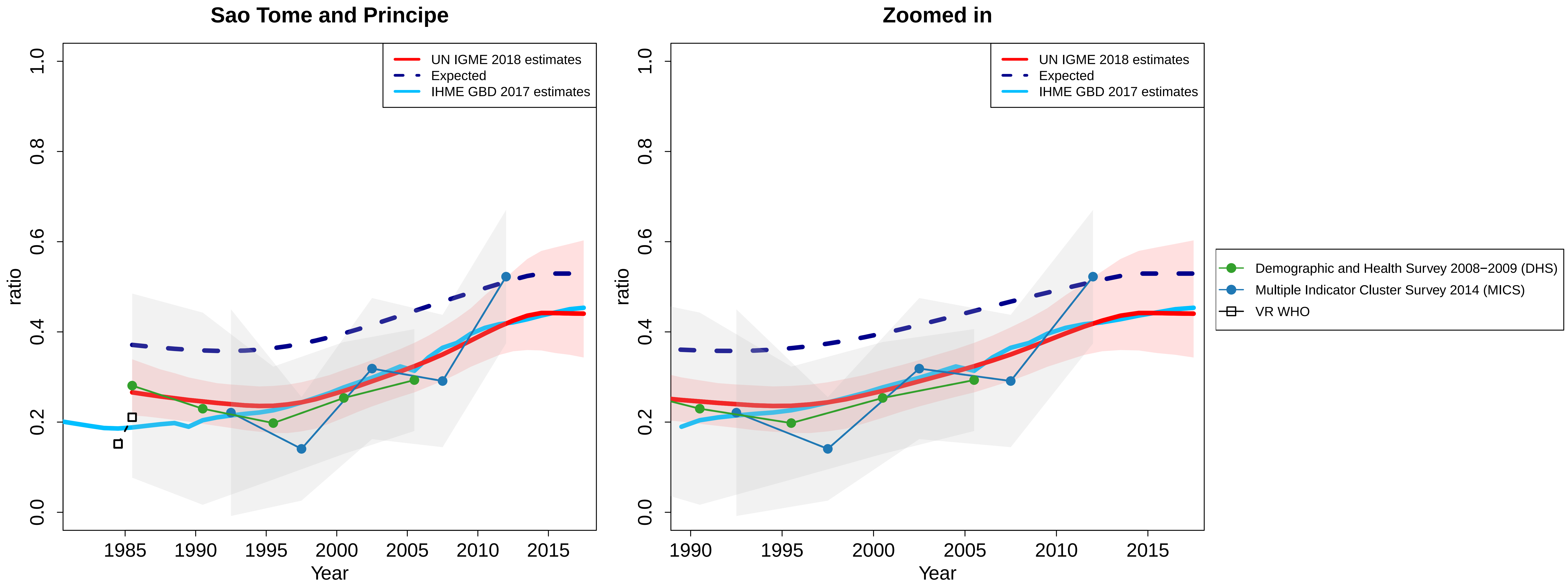

Sao Tome and Principe
Zoomed in
UN IGME 2018 estimates
Expected
IHME GBD 2017 estimates
ratio
Year
Demographic and Health Survey 2008−2009 (DHS)
Multiple Indicator Cluster Survey 2014 (MICS)
VR WHO

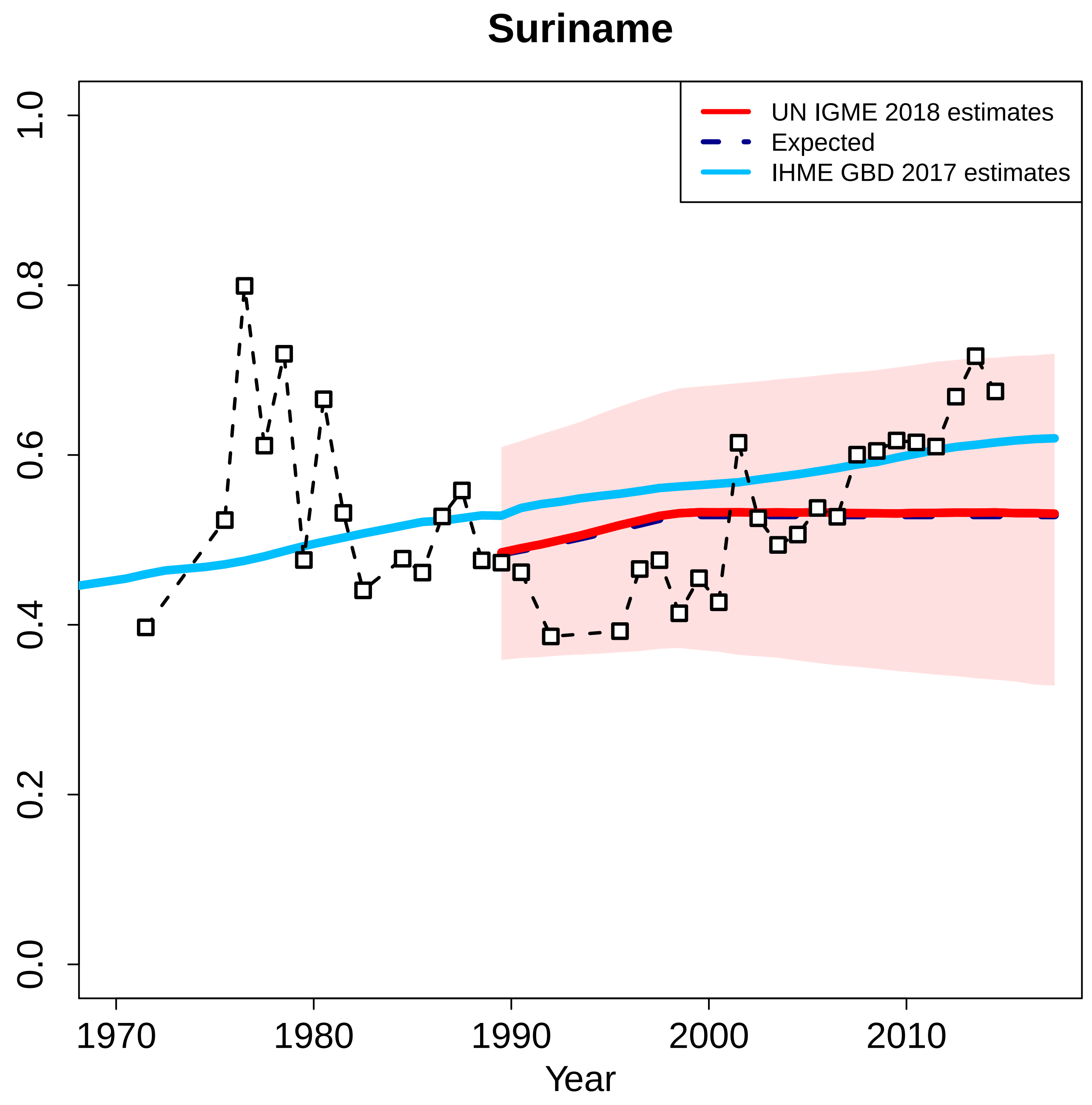

Suriname
ratio
Year
UN IGME 2018 estimates
Expected
IHME GBD 2017 estimates
0.0
0.2
0.4
0.6
0.8
1.0
1970
1980
1990
2000
2010

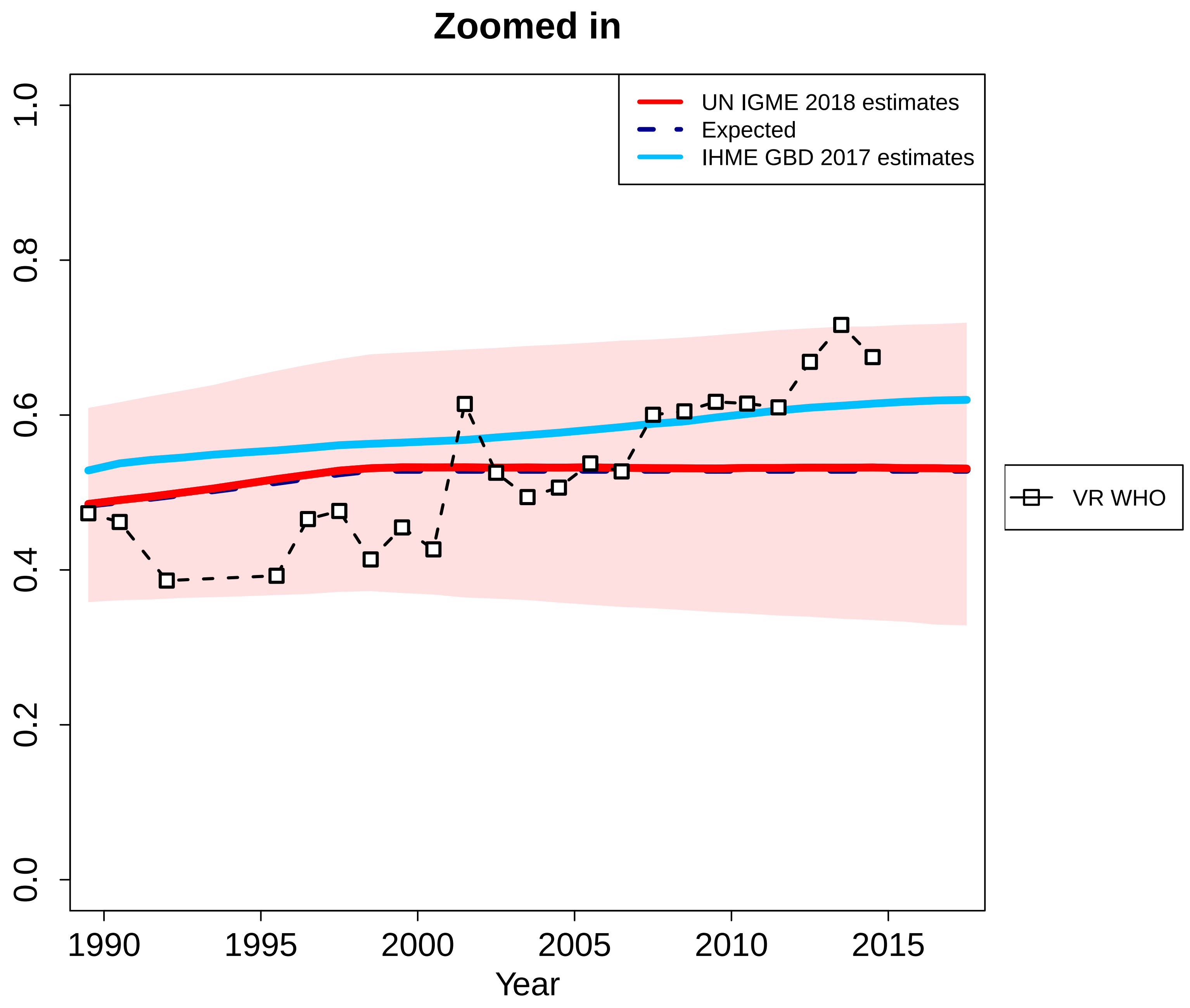

Zoomed in
ratio
Year
UN IGME 2018 estimates
Expected
IHME GBD 2017 estimates
0.0
0.2
0.4
0.6
0.8
1.0
1990
1995
2000
2005
2010
2015
VR WHO

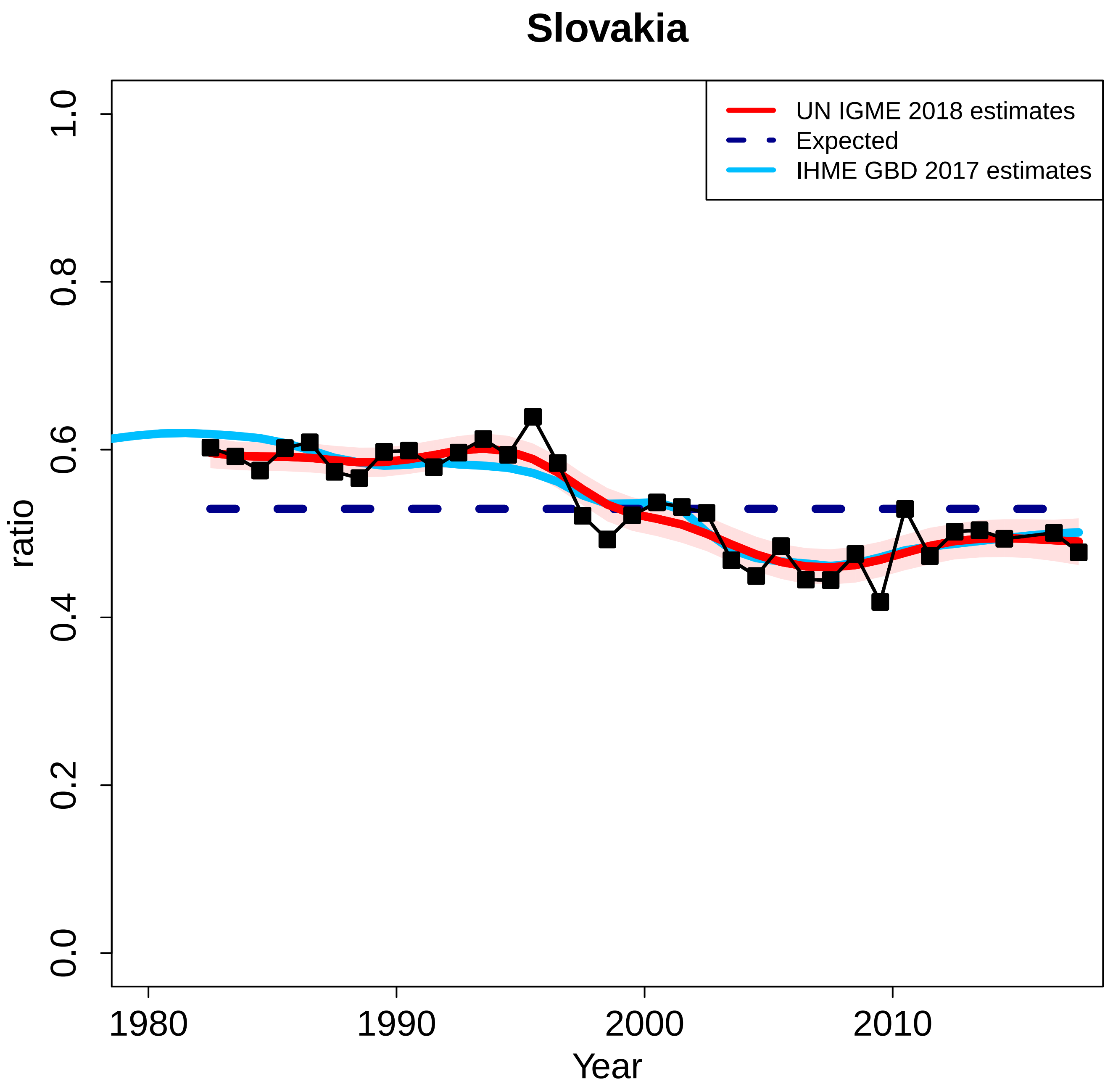

Slovakia
UN IGME 2018 estimates
Expected
IHME GBD 2017 estimates
ratio
0.0
0.2
0.4
0.6
0.8
1.0
1980
1990
2000
2010
Year

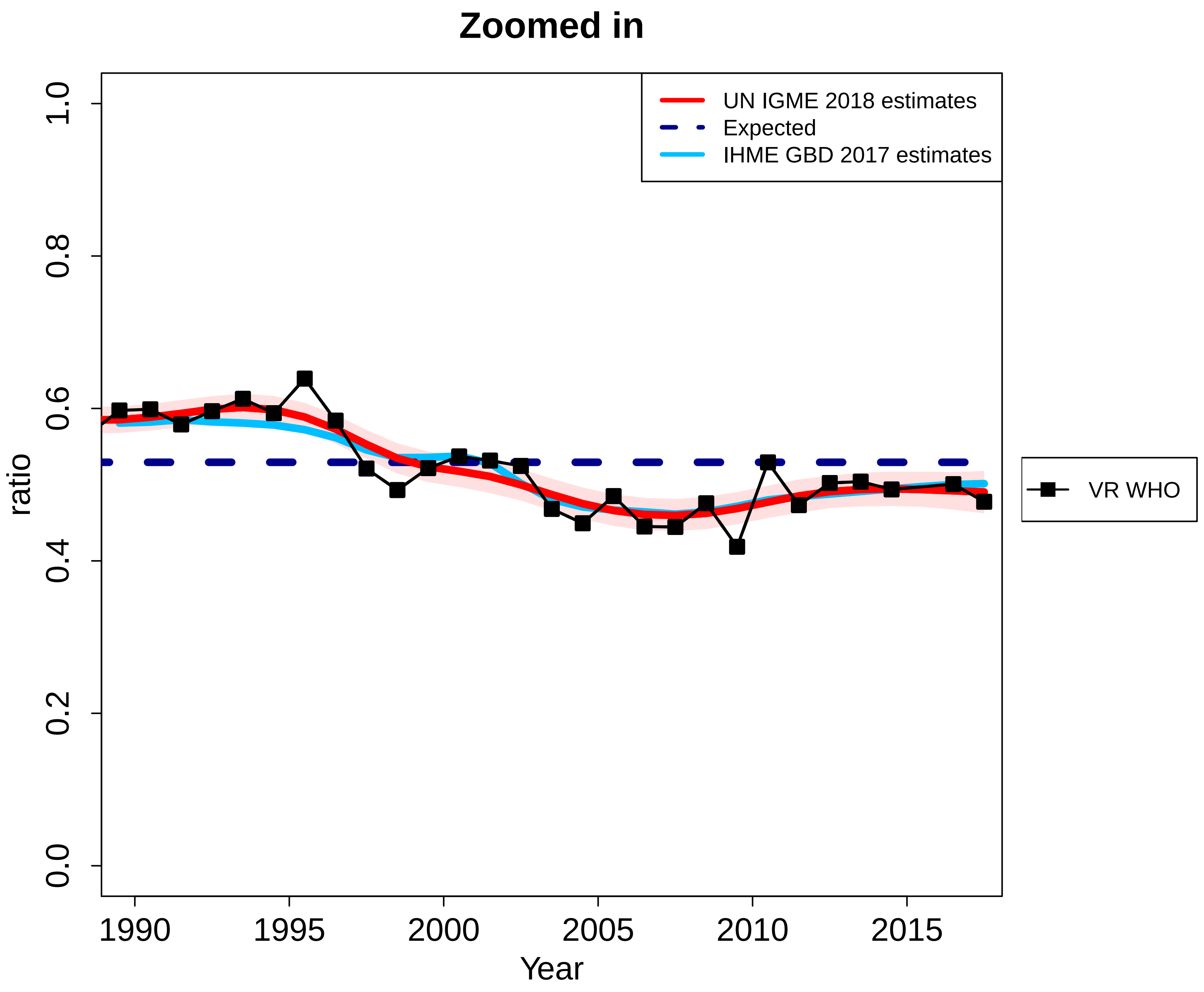

Zoomed in
UN IGME 2018 estimates
Expected
IHME GBD 2017 estimates
ratio
0.0
0.2
0.4
0.6
0.8
1.0
1990
1995
2000
2005
2010
2015
Year
VR WHO

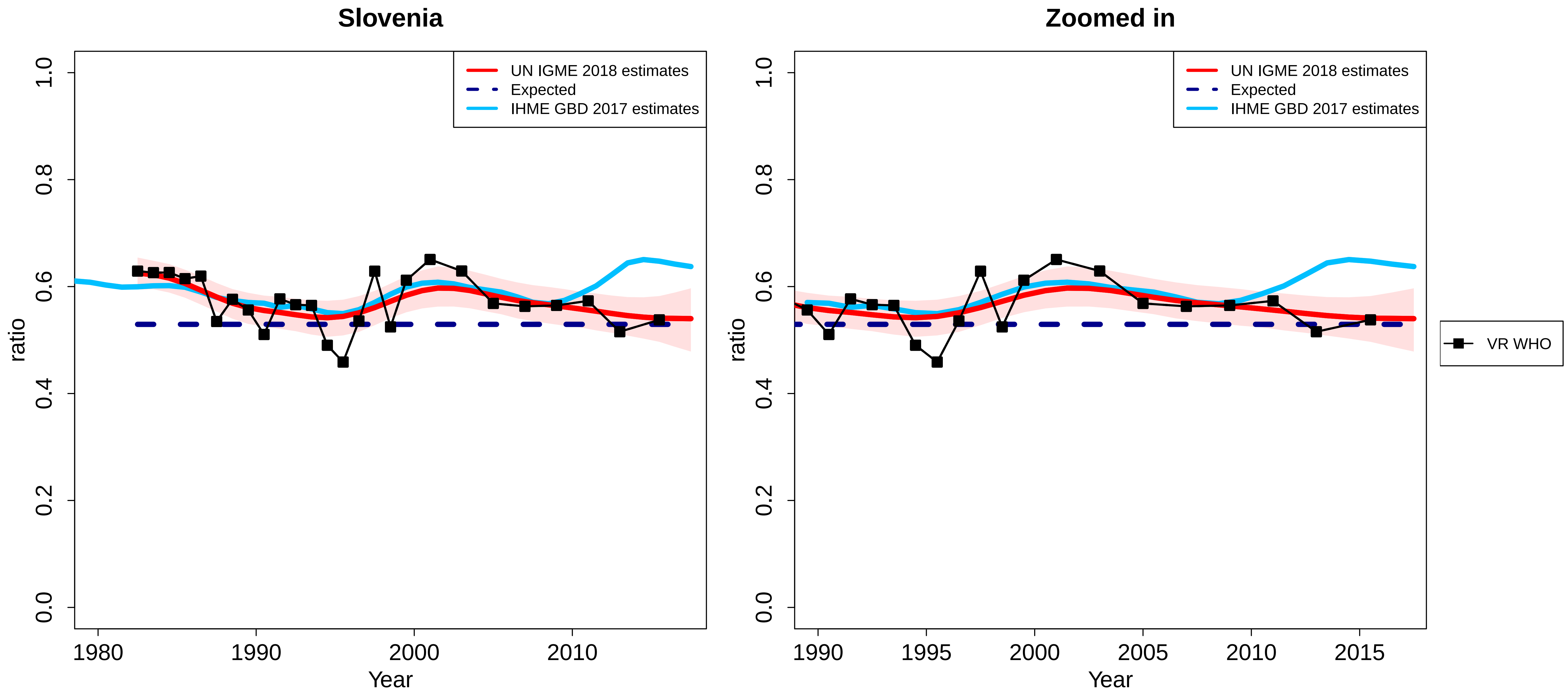
Slovenia
Zoomed in
UN IGME 2018 estimates
Expected
IHME GBD 2017 estimates
VR WHO
ratio
Year
0.0
0.2
0.4
0.6
0.8
1.0
1980
1990
2000
2010
1995
2005
2015

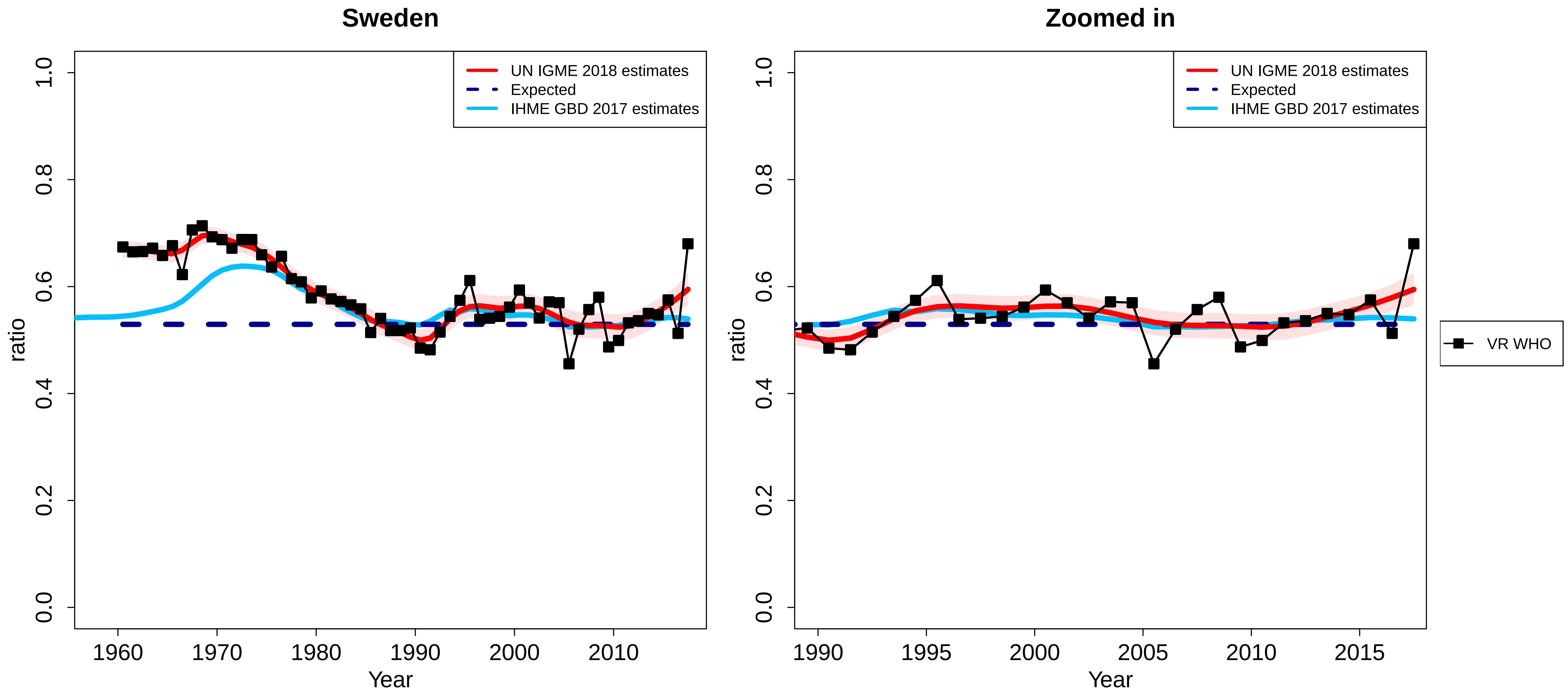

Sweden
Zoomed in
UN IGME 2018 estimates
Expected
IHME GBD 2017 estimates
VR WHO
ratio
Year
0.0
0.2
0.4
0.6
0.8
1.0
1960
1970
1980
1990
2000
2010
1990
1995
2000
2005
2010
2015

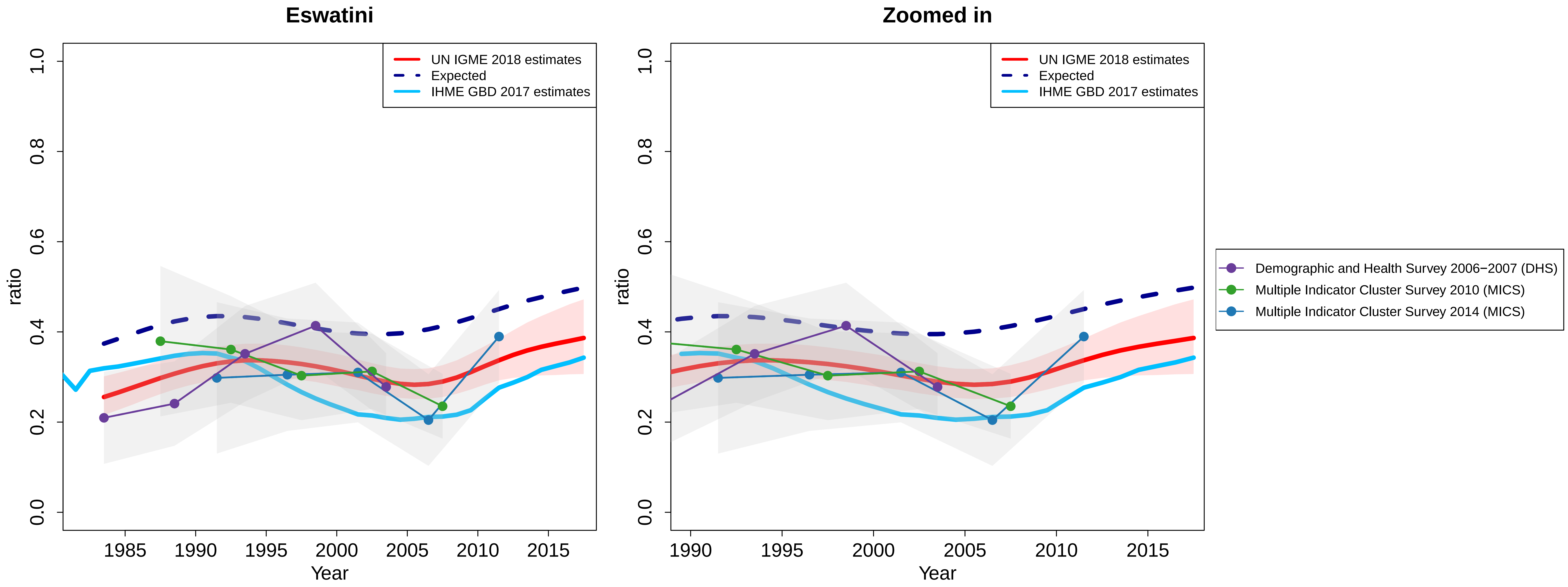
Eswatini
Zoomed in
UN IGME 2018 estimates
Expected
IHME GBD 2017 estimates
ratio
Year
0.0
0.2
0.4
0.6
0.8
1.0
1985
1990
1995
2000
2005
2010
2015
Demographic and Health Survey 2006−2007 (DHS)
Multiple Indicator Cluster Survey 2010 (MICS)
Multiple Indicator Cluster Survey 2014 (MICS)

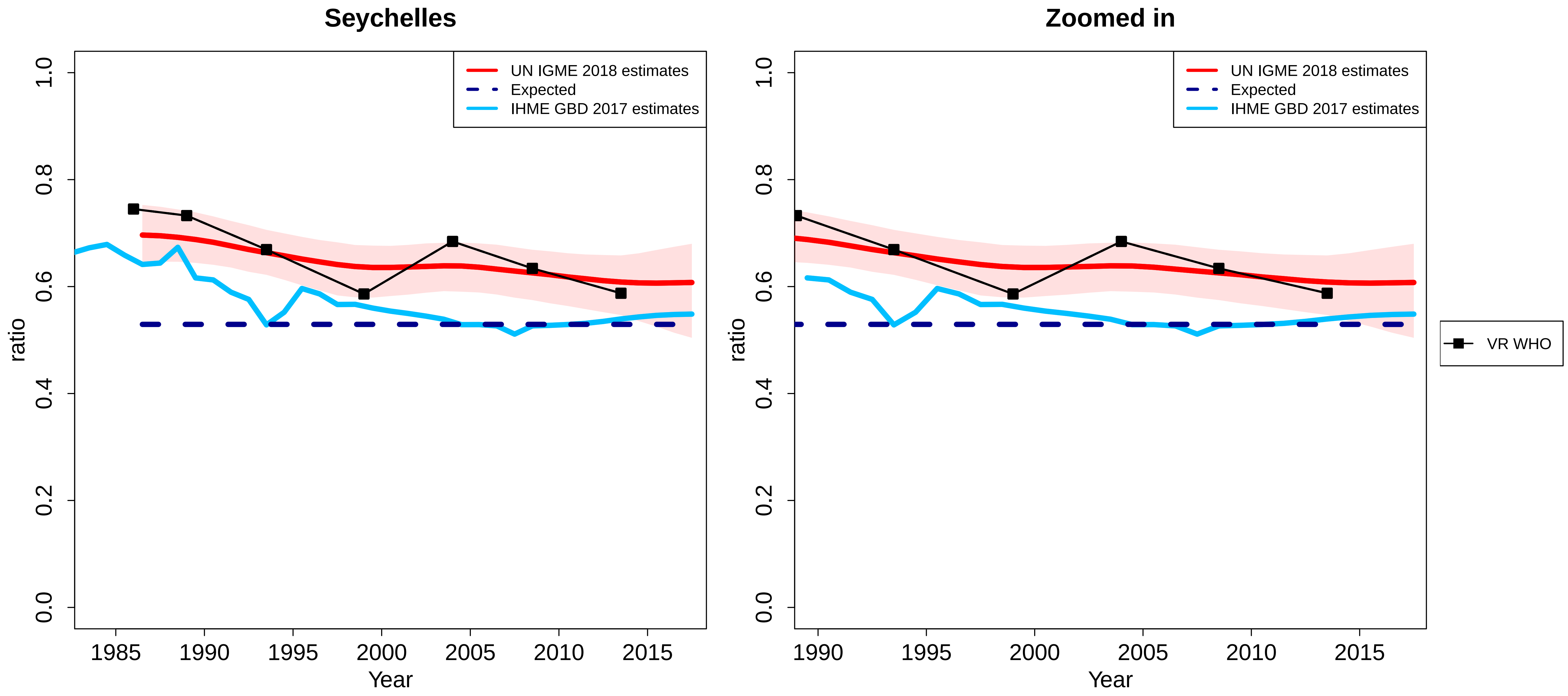

Seychelles
Zoomed in
UN IGME 2018 estimates
Expected
IHME GBD 2017 estimates
VR WHO
ratio
Year
0.0
0.2
0.4
0.6
0.8
1.0
1985
1990
1995
2000
2005
2010
2015

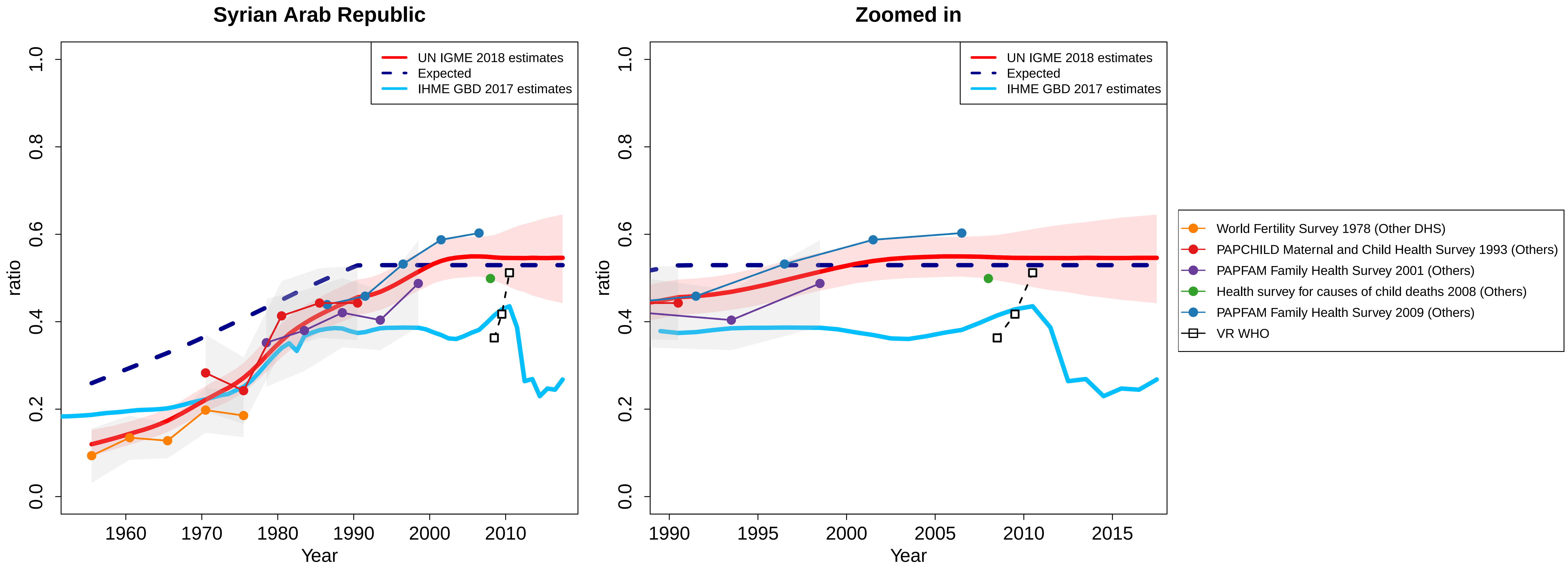

Syrian Arab Republic
Zoomed in
UN IGME 2018 estimates
Expected
IHME GBD 2017 estimates
ratio
Year
0.0
0.2
0.4
0.6
0.8
1.0
1960
1970
1980
1990
2000
2010
1995
2005
2015
World Fertility Survey 1978 (Other DHS)
PAPCHILD Maternal and Child Health Survey 1993 (Others)
PAPFAM Family Health Survey 2001 (Others)
Health survey for causes of child deaths 2008 (Others)
PAPFAM Family Health Survey 2009 (Others)
VR WHO

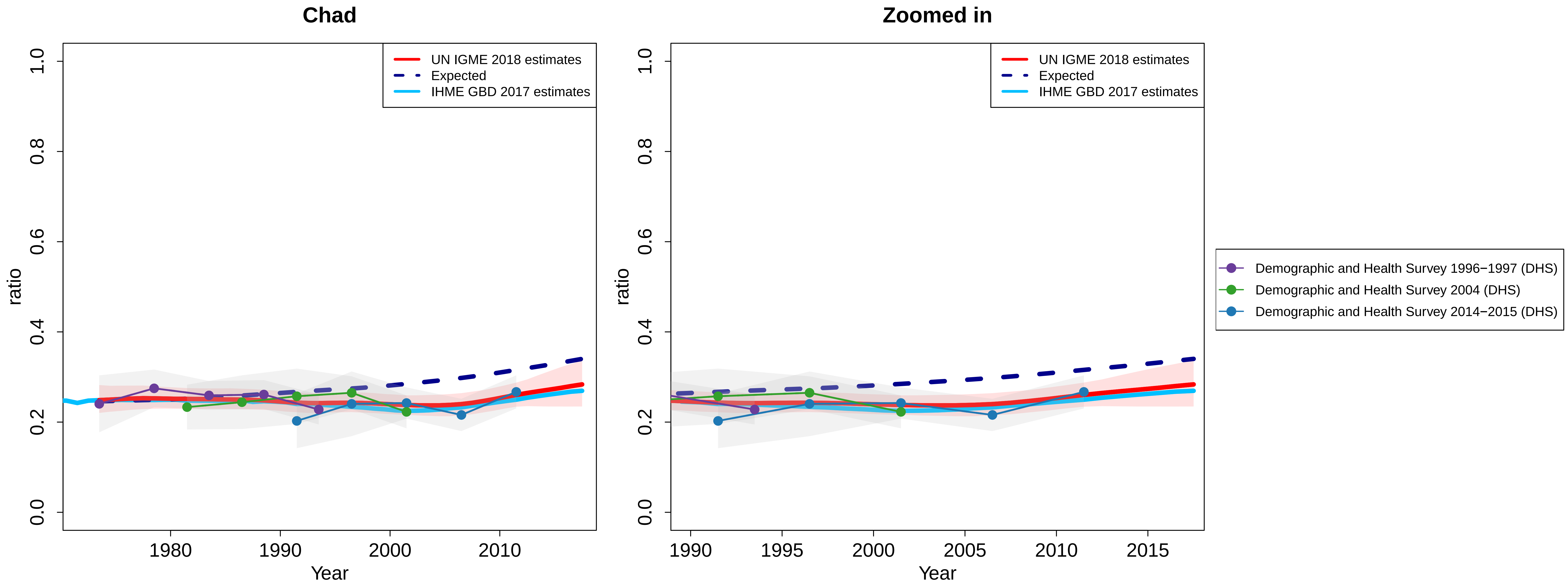

Chad
Zoomed in
UN IGME 2018 estimates
Expected
IHME GBD 2017 estimates
ratio
Year
0.0
0.2
0.4
0.6
0.8
1.0
1980
1990
2000
2010
1995
2005
2015
Demographic and Health Survey 1996−1997 (DHS)
Demographic and Health Survey 2004 (DHS)
Demographic and Health Survey 2014−2015 (DHS)

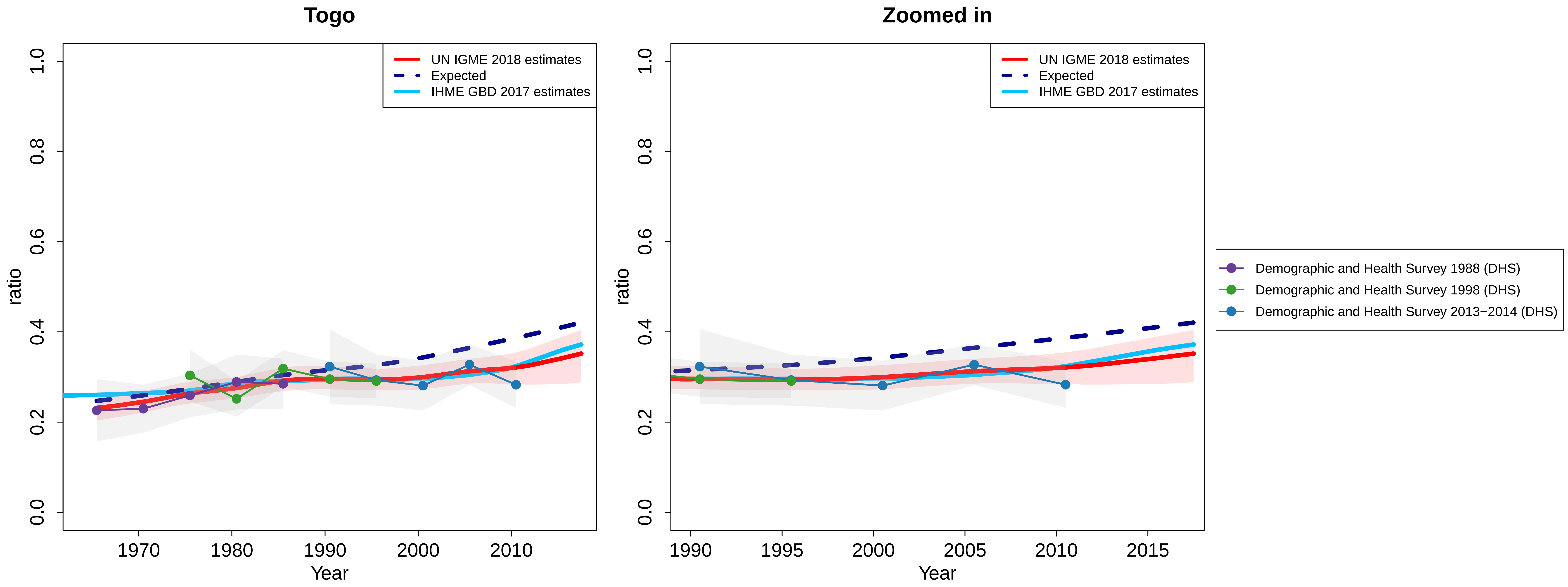

Togo
Zoomed in
UN IGME 2018 estimates
Expected
IHME GBD 2017 estimates
Demographic and Health Survey 1988 (DHS)
Demographic and Health Survey 1998 (DHS)
Demographic and Health Survey 2013−2014 (DHS)
ratio
Year
0.0
0.2
0.4
0.6
0.8
1.0
1970
1980
1990
2000
2010
1995
2005
2015

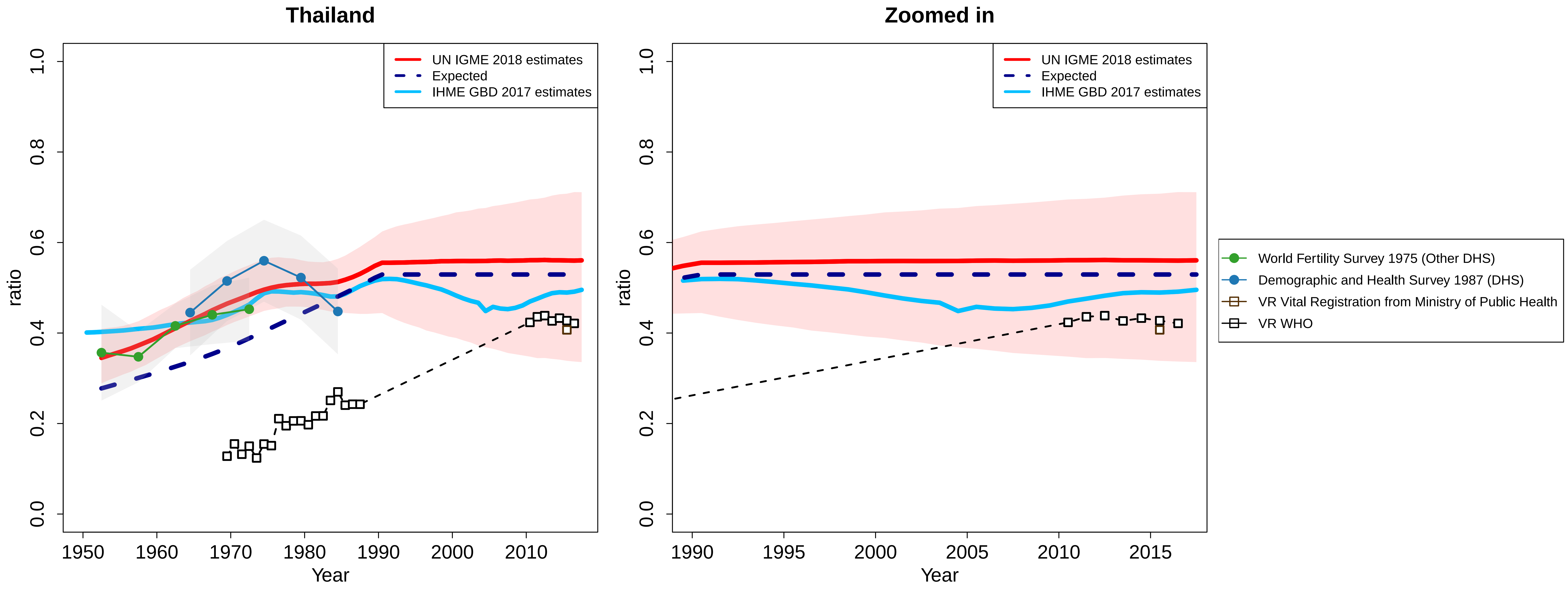

Thailand
Zoomed in
UN IGME 2018 estimates
Expected
IHME GBD 2017 estimates
ratio
Year
0.0
0.2
0.4
0.6
0.8
1.0
1950
1960
1970
1980
1990
2000
2010
1990
1995
2000
2005
2010
2015
World Fertility Survey 1975 (Other DHS)
Demographic and Health Survey 1987 (DHS)
VR Vital Registration from Ministry of Public Health
VR WHO

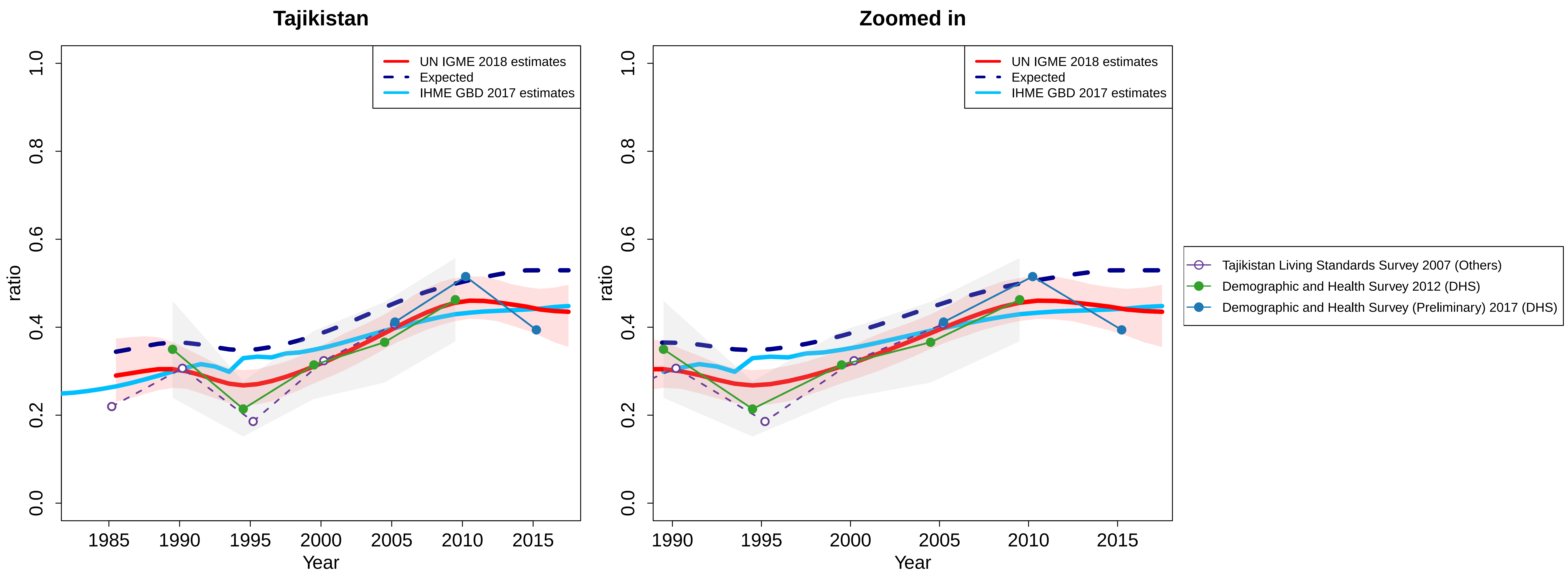
Tajikistan
Zoomed in
UN IGME 2018 estimates
Expected
IHME GBD 2017 estimates
ratio
Year
0.0
0.2
0.4
0.6
0.8
1.0
1985
1990
1995
2000
2005
2010
2015
Tajikistan Living Standards Survey 2007 (Others)
Demographic and Health Survey 2012 (DHS)
Demographic and Health Survey (Preliminary) 2017 (DHS)

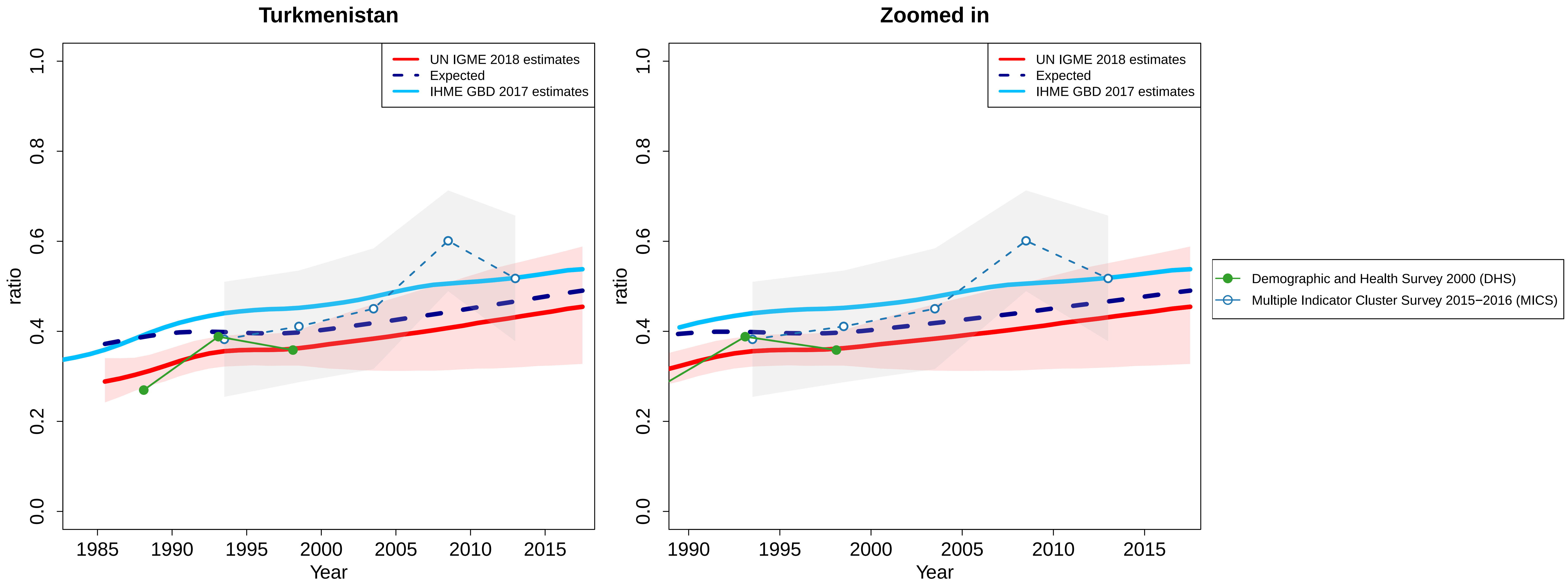

Turkmenistan
Zoomed in
UN IGME 2018 estimates
Expected
IHME GBD 2017 estimates
Demographic and Health Survey 2000 (DHS)
Multiple Indicator Cluster Survey 2015−2016 (MICS)
ratio
Year
0.0
0.2
0.4
0.6
0.8
1.0
1985
1990
1995
2000
2005
2010
2015

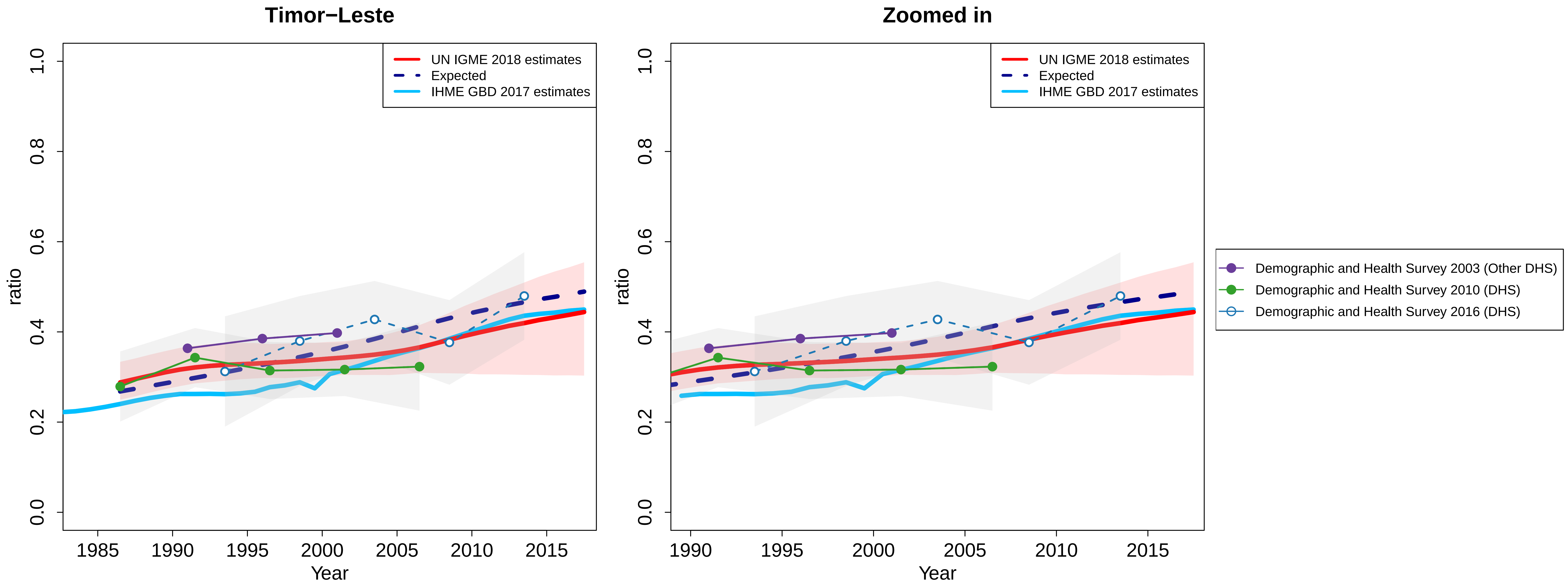

Timor−Leste
Zoomed in
UN IGME 2018 estimates
Expected
IHME GBD 2017 estimates
ratio
Year
0.0
0.2
0.4
0.6
0.8
1.0
1985
1990
1995
2000
2005
2010
2015
Demographic and Health Survey 2003 (Other DHS)
Demographic and Health Survey 2010 (DHS)
Demographic and Health Survey 2016 (DHS)

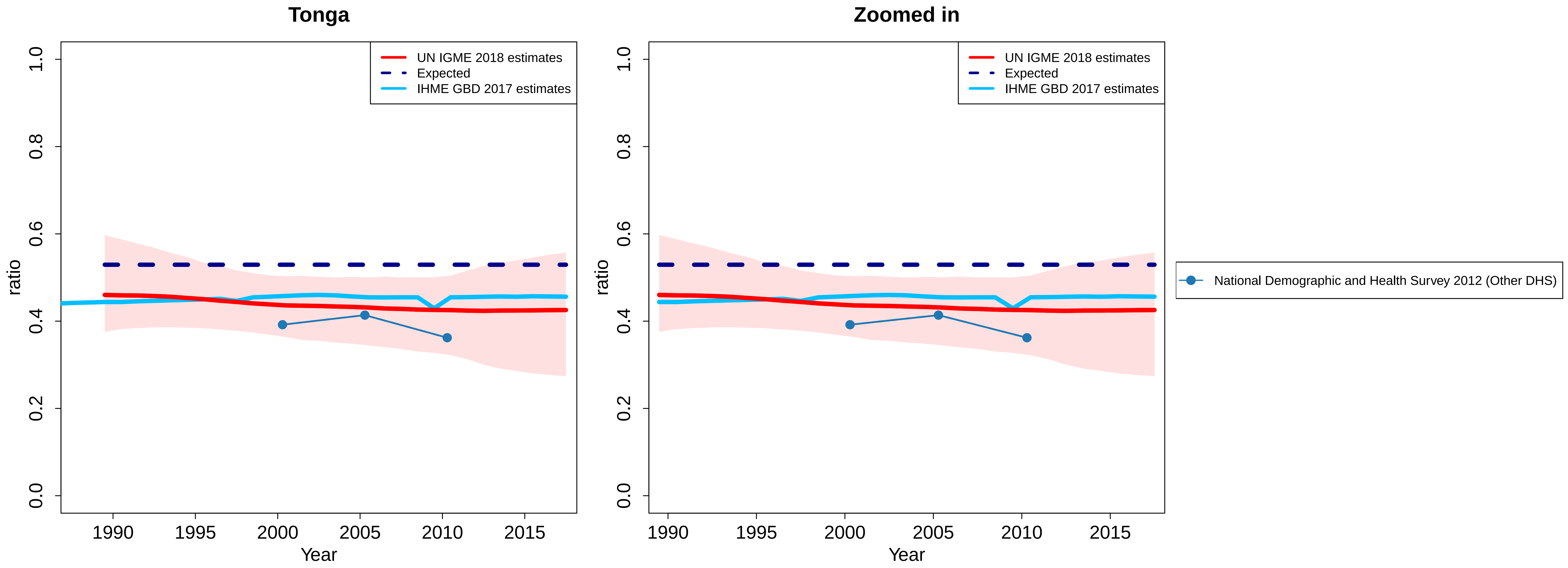
Tonga
Zoomed in
UN IGME 2018 estimates
Expected
IHME GBD 2017 estimates
National Demographic and Health Survey 2012 (Other DHS)
ratio
Year
0.0
0.2
0.4
0.6
0.8
1.0
1990
1995
2000
2005
2010
2015

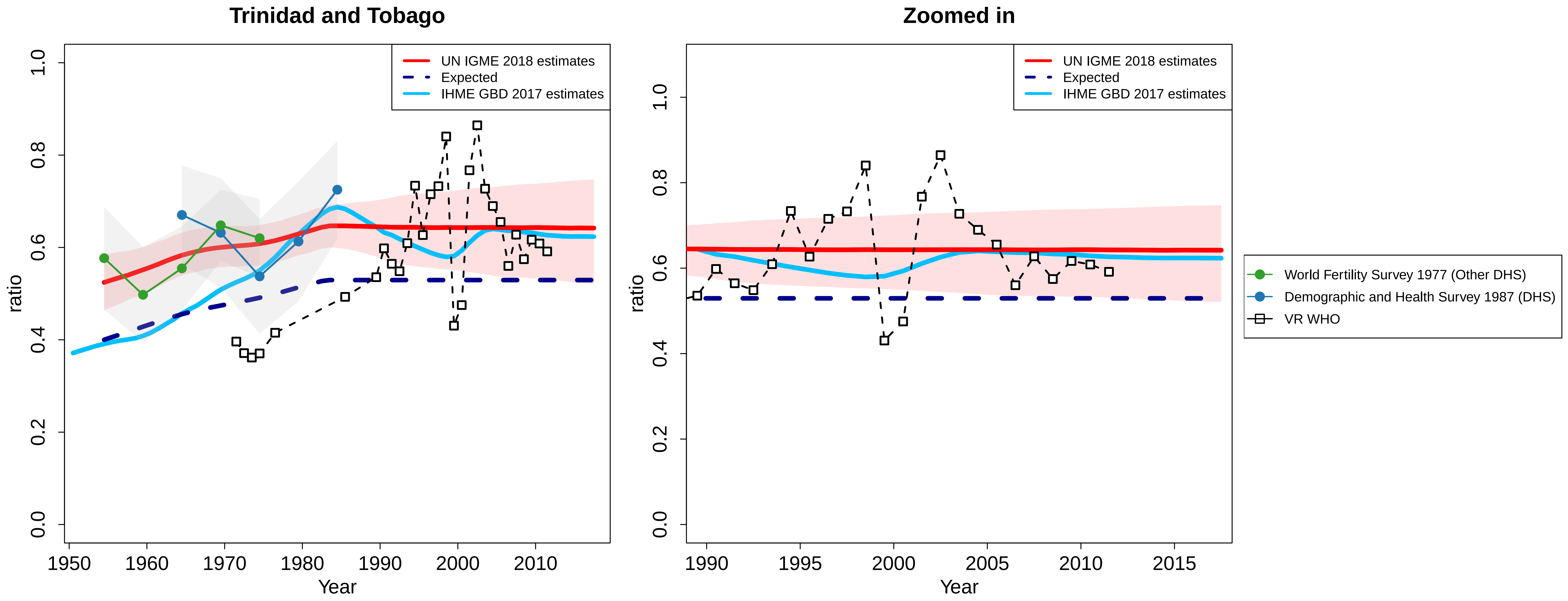

Trinidad and Tobago
Zoomed in
UN IGME 2018 estimates
Expected
IHME GBD 2017 estimates
World Fertility Survey 1977 (Other DHS)
Demographic and Health Survey 1987 (DHS)
VR WHO
ratio
Year
0.0
0.2
0.4
0.6
0.8
1.0
1950
1960
1970
1980
1990
2000
2010
1995
2005
2015

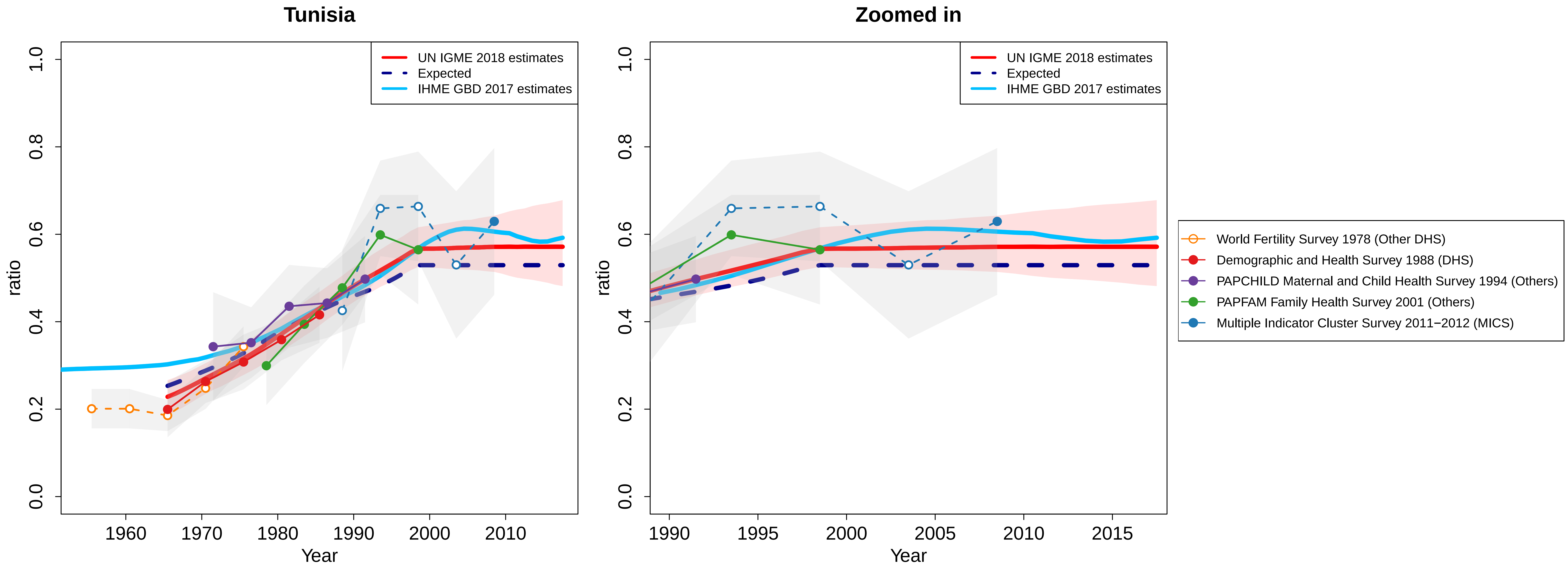

Tunisia
Zoomed in
UN IGME 2018 estimates
Expected
IHME GBD 2017 estimates
ratio
Year
1960
1970
1980
1990
2000
2010
1995
2005
2015
0.0
0.2
0.4
0.6
0.8
1.0
World Fertility Survey 1978 (Other DHS)
Demographic and Health Survey 1988 (DHS)
PAPCHILD Maternal and Child Health Survey 1994 (Others)
PAPFAM Family Health Survey 2001 (Others)
Multiple Indicator Cluster Survey 2011−2012 (MICS)

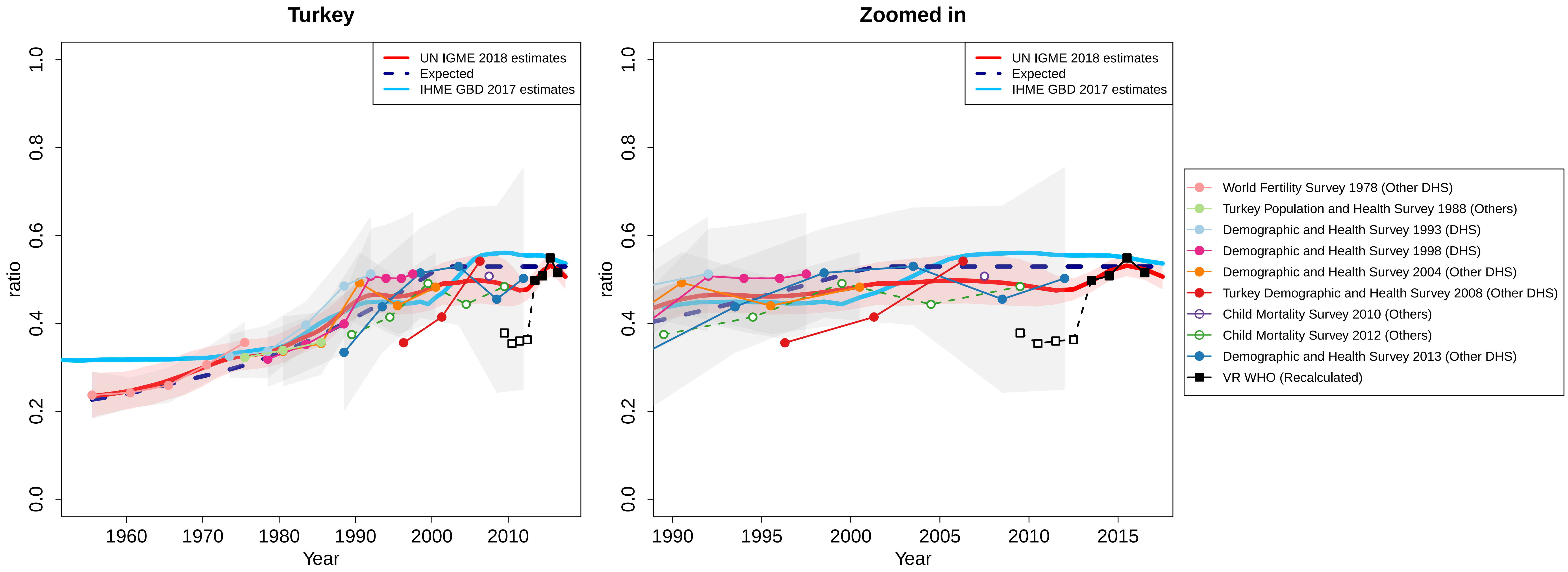
Turkey
Zoomed in
UN IGME 2018 estimates
Expected
IHME GBD 2017 estimates
ratio
Year
World Fertility Survey 1978 (Other DHS)
Turkey Population and Health Survey 1988 (Others)
Demographic and Health Survey 1993 (DHS)
Demographic and Health Survey 1998 (DHS)
Demographic and Health Survey 2004 (Other DHS)
Turkey Demographic and Health Survey 2008 (Other DHS)
Child Mortality Survey 2010 (Others)
Child Mortality Survey 2012 (Others)
Demographic and Health Survey 2013 (Other DHS)
VR WHO (Recalculated)

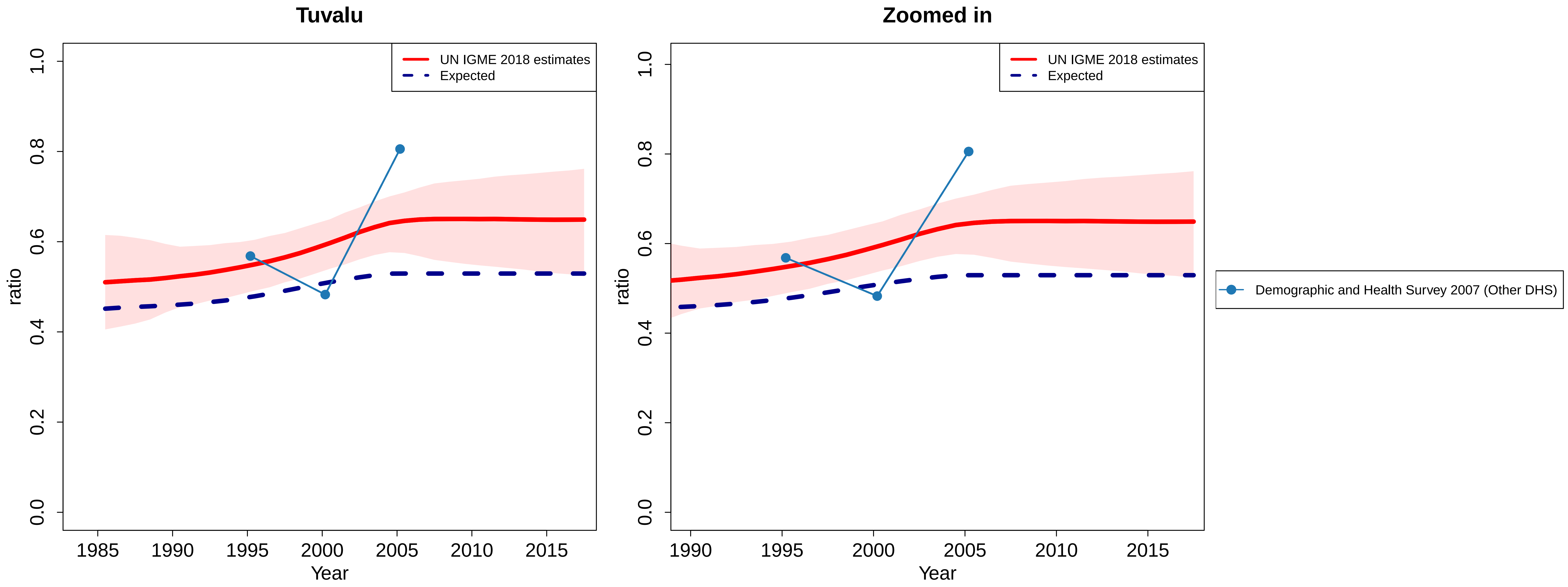

Tuvalu
Zoomed in
UN IGME 2018 estimates
Expected
ratio
Year
0.0
0.2
0.4
0.6
0.8
1.0
1985
1990
1995
2000
2005
2010
2015
Demographic and Health Survey 2007 (Other DHS)

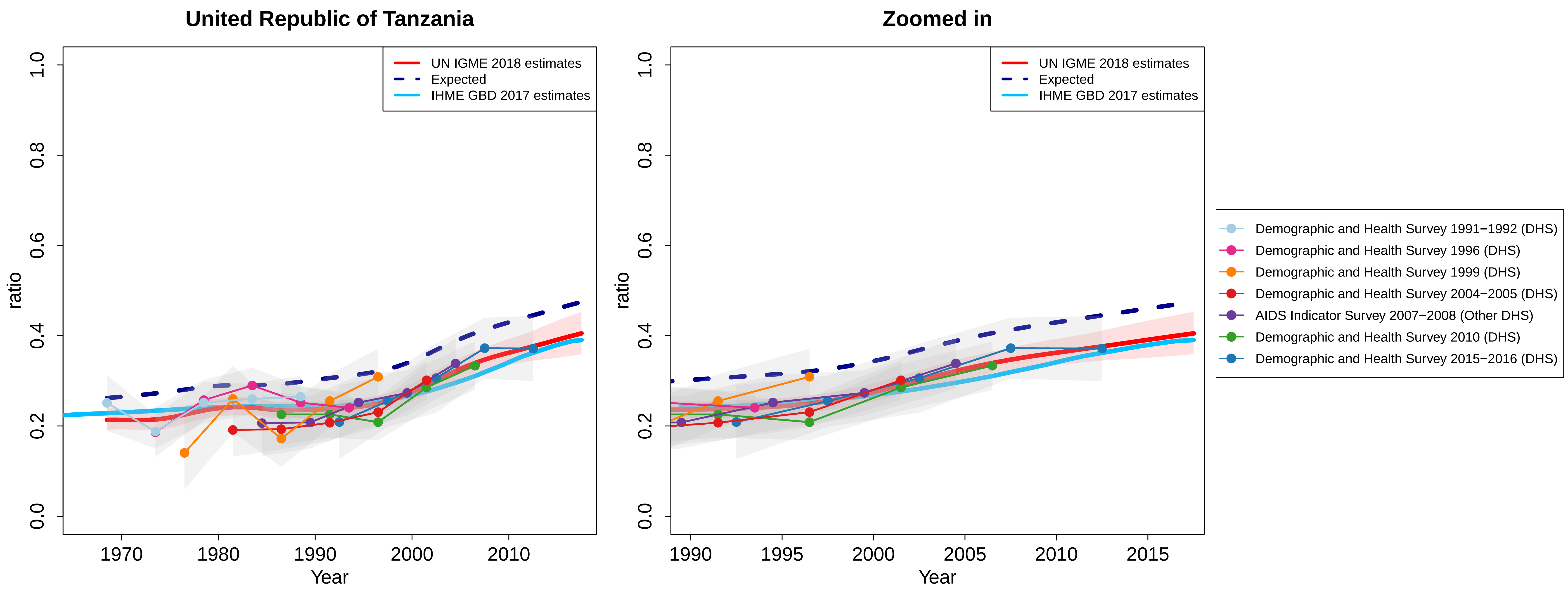
United Republic of Tanzania
Zoomed in
UN IGME 2018 estimates
Expected
IHME GBD 2017 estimates
ratio
Year
0.0
0.2
0.4
0.6
0.8
1.0
1970
1980
1990
2000
2010
1995
2005
2015
Demographic and Health Survey 1991−1992 (DHS)
Demographic and Health Survey 1996 (DHS)
Demographic and Health Survey 1999 (DHS)
Demographic and Health Survey 2004−2005 (DHS)
AIDS Indicator Survey 2007−2008 (Other DHS)
Demographic and Health Survey 2010 (DHS)
Demographic and Health Survey 2015−2016 (DHS)

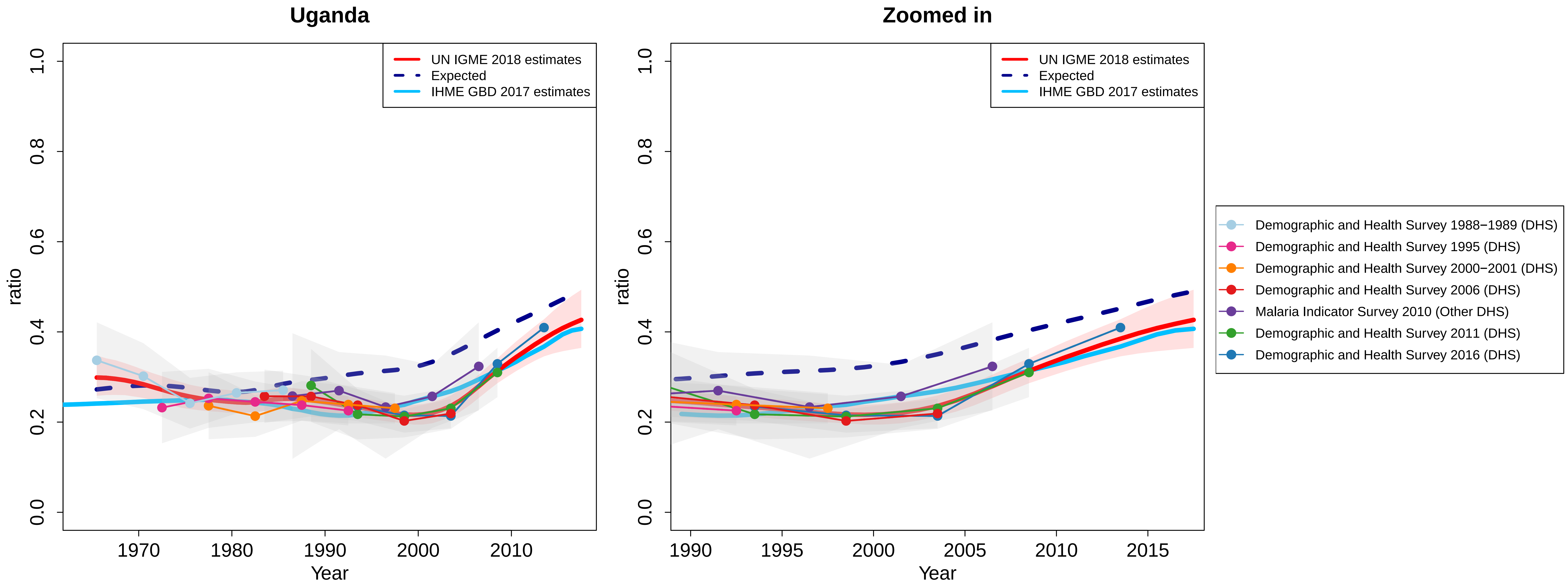

Uganda
Zoomed in
UN IGME 2018 estimates
Expected
IHME GBD 2017 estimates
ratio
Year
0.0
0.2
0.4
0.6
0.8
1.0
1970
1980
1990
2000
2010
1995
2005
2015
Demographic and Health Survey 1988−1989 (DHS)
Demographic and Health Survey 1995 (DHS)
Demographic and Health Survey 2000−2001 (DHS)
Demographic and Health Survey 2006 (DHS)
Malaria Indicator Survey 2010 (Other DHS)
Demographic and Health Survey 2011 (DHS)
Demographic and Health Survey 2016 (DHS)

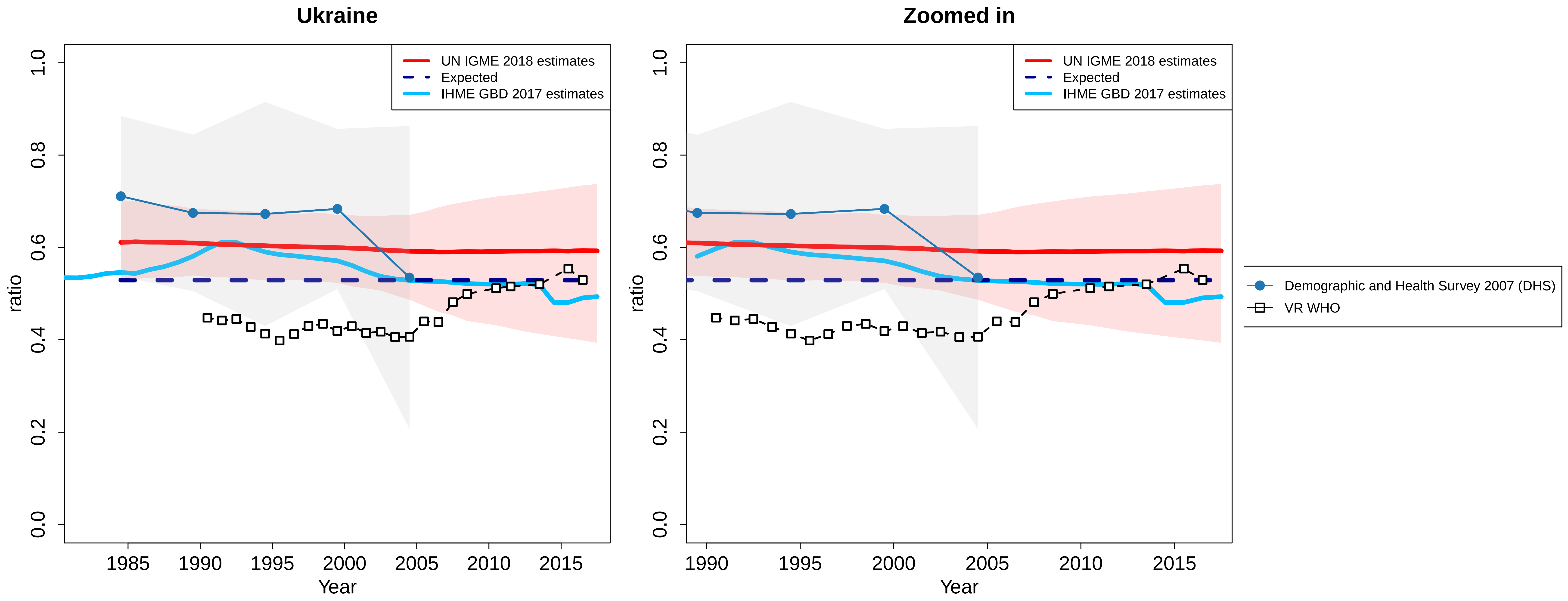
Ukraine
Zoomed in
UN IGME 2018 estimates
Expected
IHME GBD 2017 estimates
Demographic and Health Survey 2007 (DHS)
VR WHO
ratio
Year
0.0
0.2
0.4
0.6
0.8
1.0
1985
1990
1995
2000
2005
2010
2015

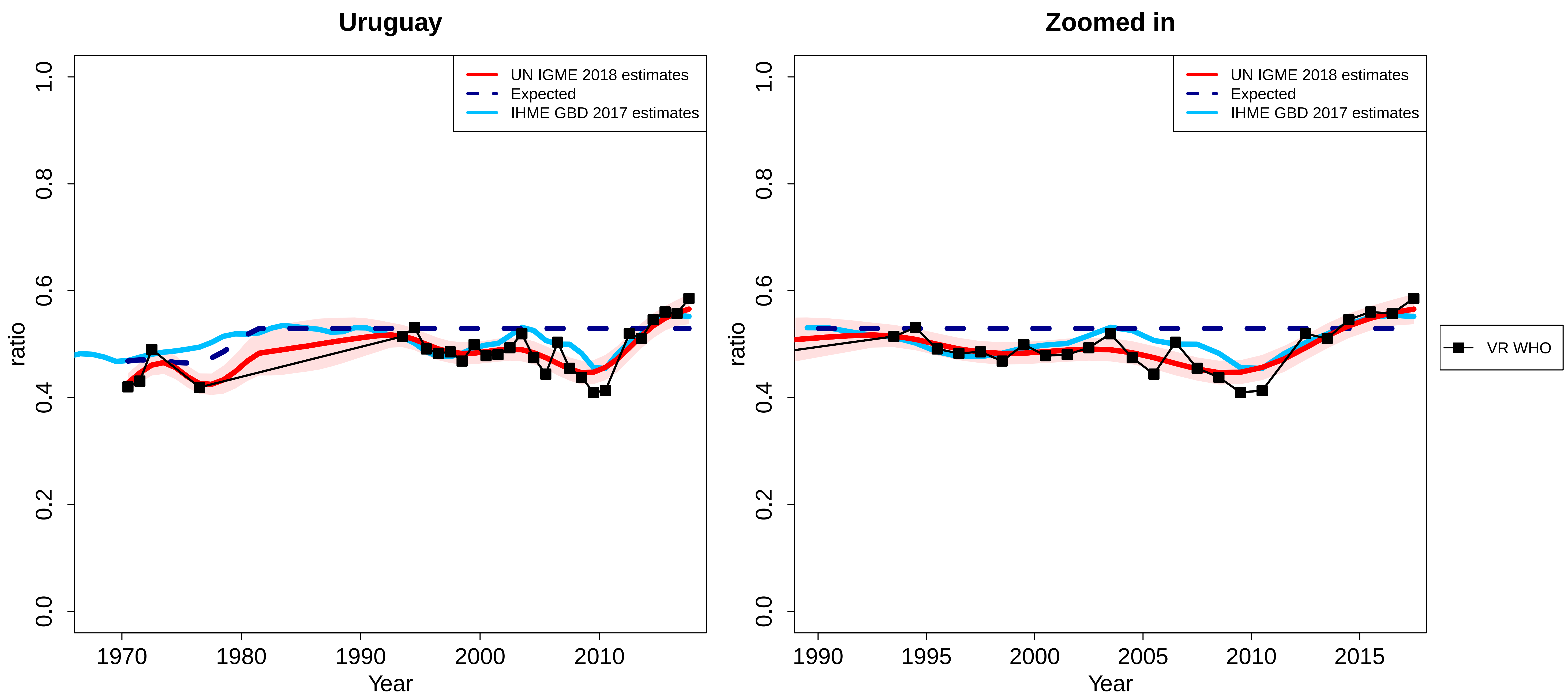

Uruguay
Zoomed in
UN IGME 2018 estimates
Expected
IHME GBD 2017 estimates
VR WHO
ratio
Year
0.0
0.2
0.4
0.6
0.8
1.0
1970
1980
1990
2000
2010
1995
2005
2015

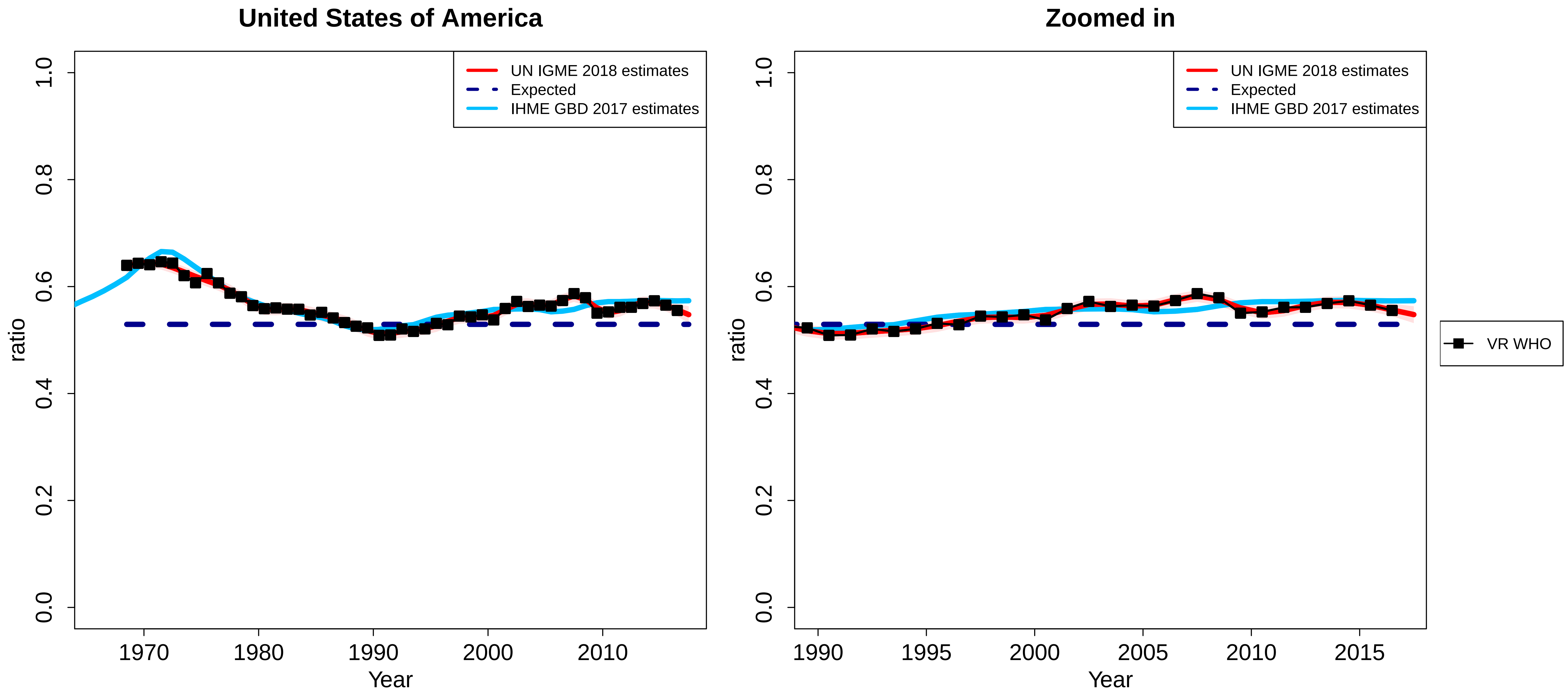

United States of America
Zoomed in
UN IGME 2018 estimates
Expected
IHME GBD 2017 estimates
VR WHO
ratio
Year
0.0
0.2
0.4
0.6
0.8
1.0
1970
1980
1990
2000
2010
1995
2005
2015

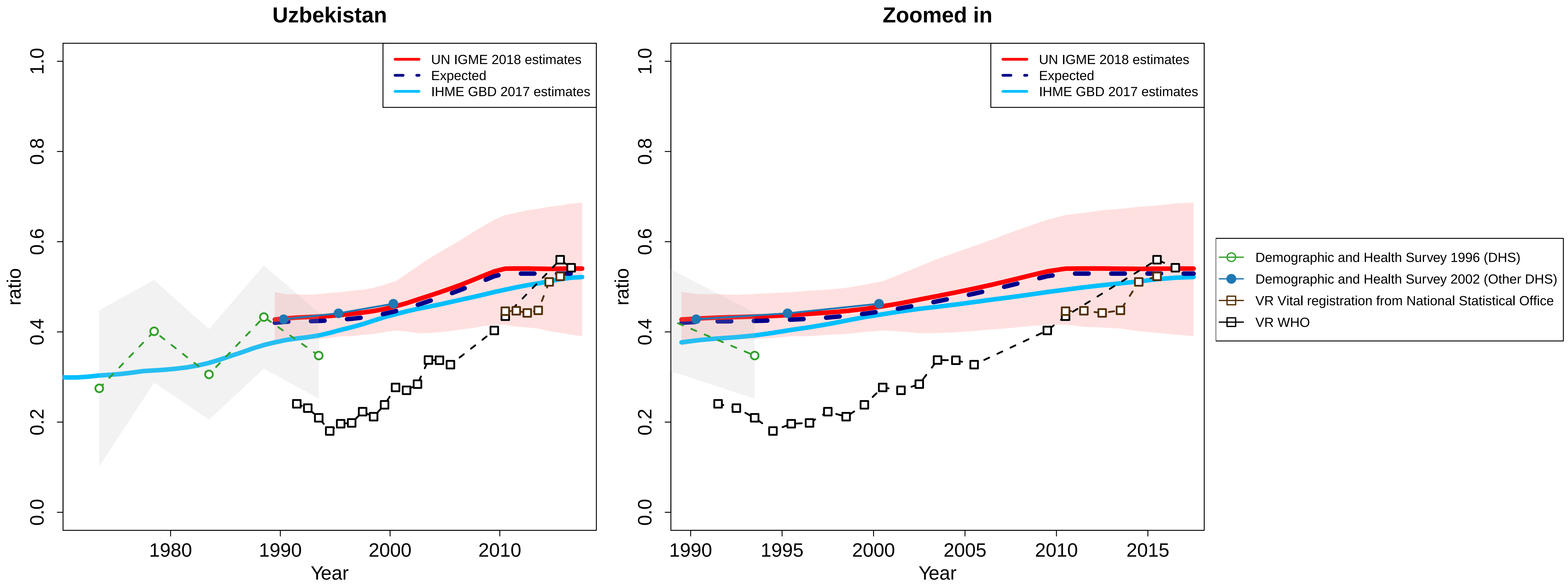
Uzbekistan
Zoomed in
UN IGME 2018 estimates
Expected
IHME GBD 2017 estimates
ratio
Year
Demographic and Health Survey 1996 (DHS)
Demographic and Health Survey 2002 (Other DHS)
VR Vital registration from National Statistical Office
VR WHO

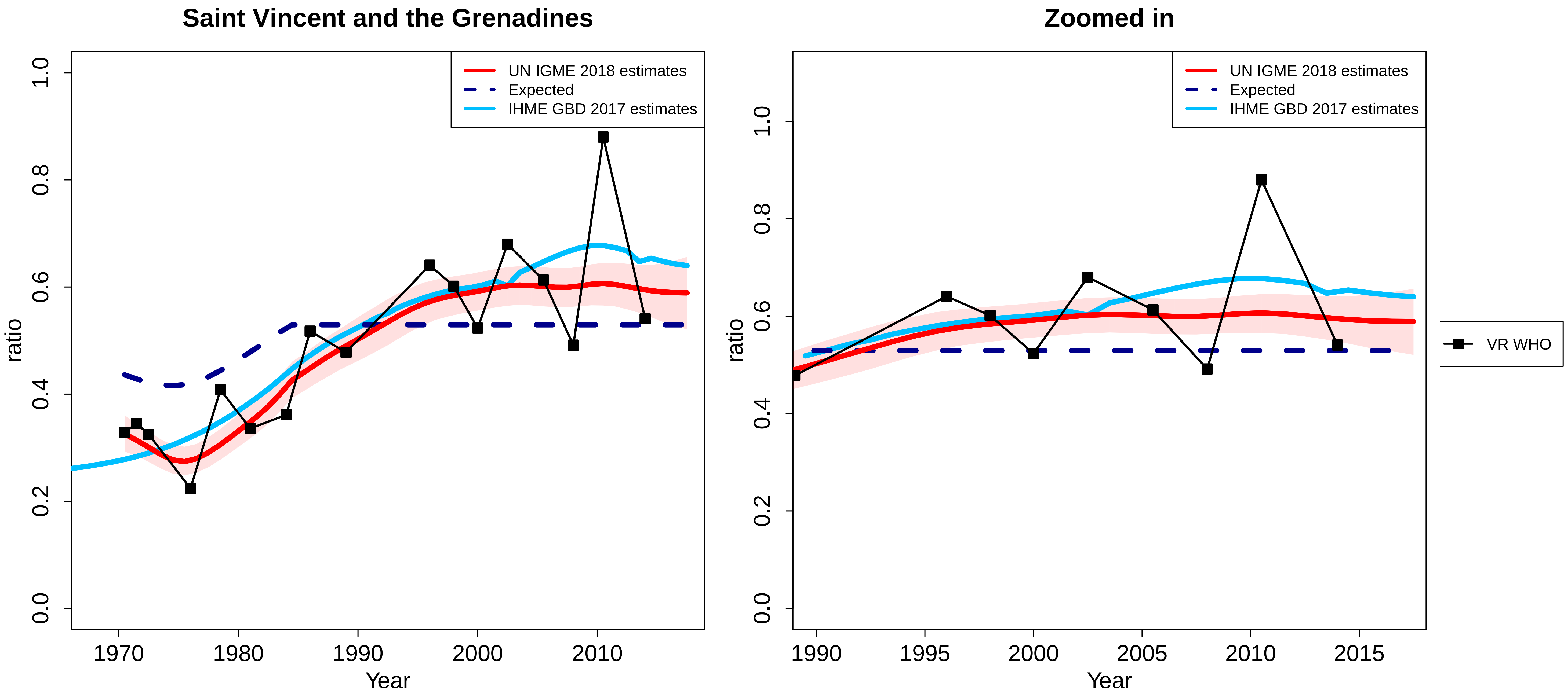
Saint Vincent and the Grenadines
Zoomed in
UN IGME 2018 estimates
Expected
IHME GBD 2017 estimates
VR WHO
ratio
Year
0.0
0.2
0.4
0.6
0.8
1.0
1970
1980
1990
2000
2010
1995
2005
2015

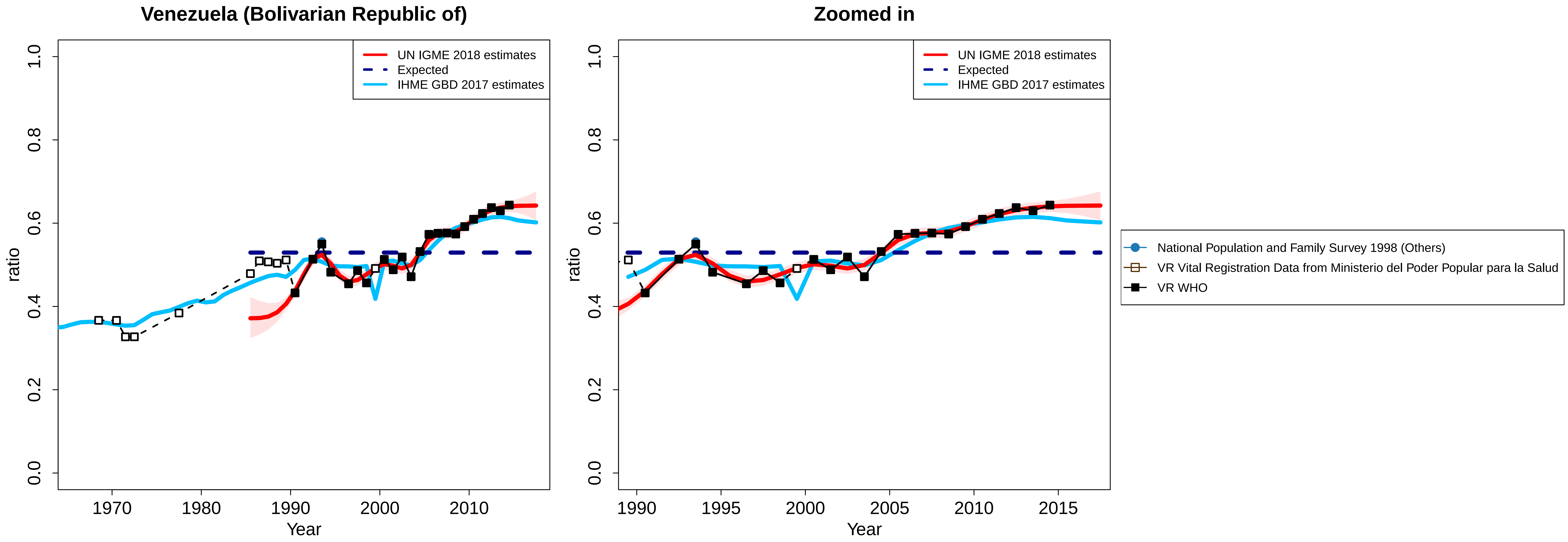

Venezuela (Bolivarian Republic of)
Zoomed in
UN IGME 2018 estimates
Expected
IHME GBD 2017 estimates
ratio
Year
0.0
0.2
0.4
0.6
0.8
1.0
1970
1980
1990
2000
2010
1990
1995
2000
2005
2010
2015
National Population and Family Survey 1998 (Others)
VR Vital Registration Data from Ministerio del Poder Popular para la Salud
VR WHO

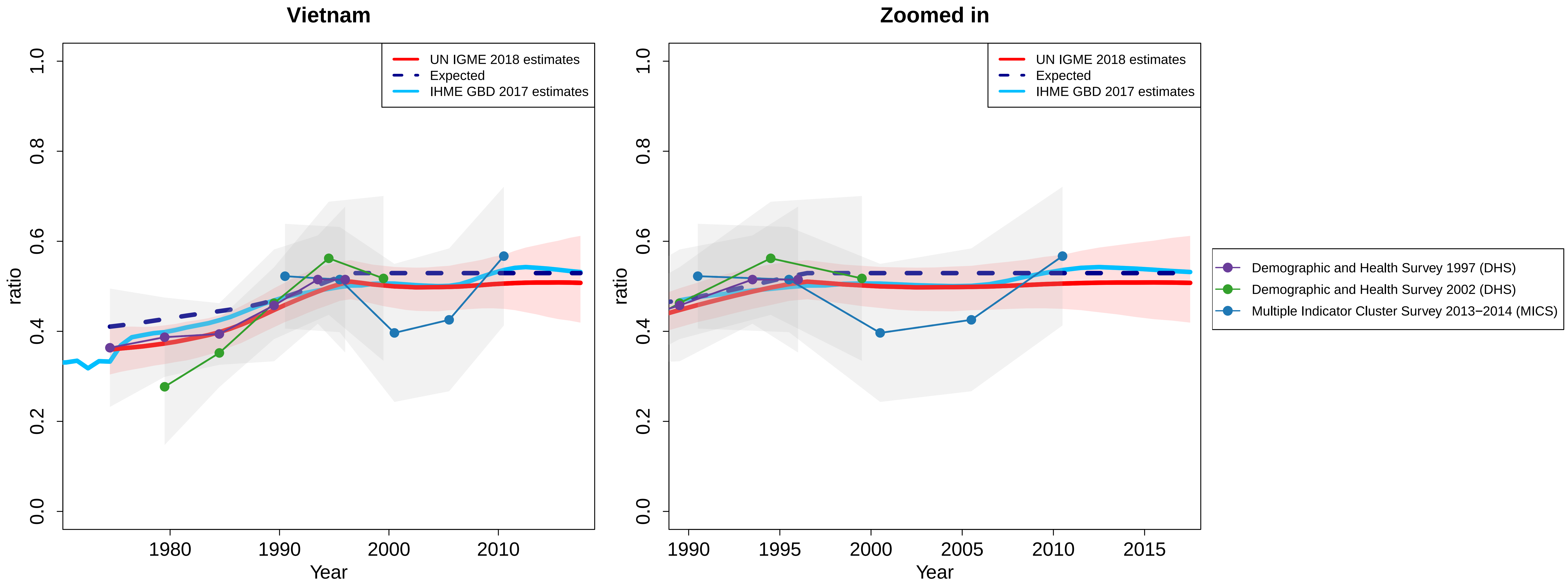

Vietnam
Zoomed in
ratio
Year
0.0
0.2
0.4
0.6
0.8
1.0
1980
1990
2000
2010
1995
2005
2015
UN IGME 2018 estimates
Expected
IHME GBD 2017 estimates
Demographic and Health Survey 1997 (DHS)
Demographic and Health Survey 2002 (DHS)
Multiple Indicator Cluster Survey 2013−2014 (MICS)

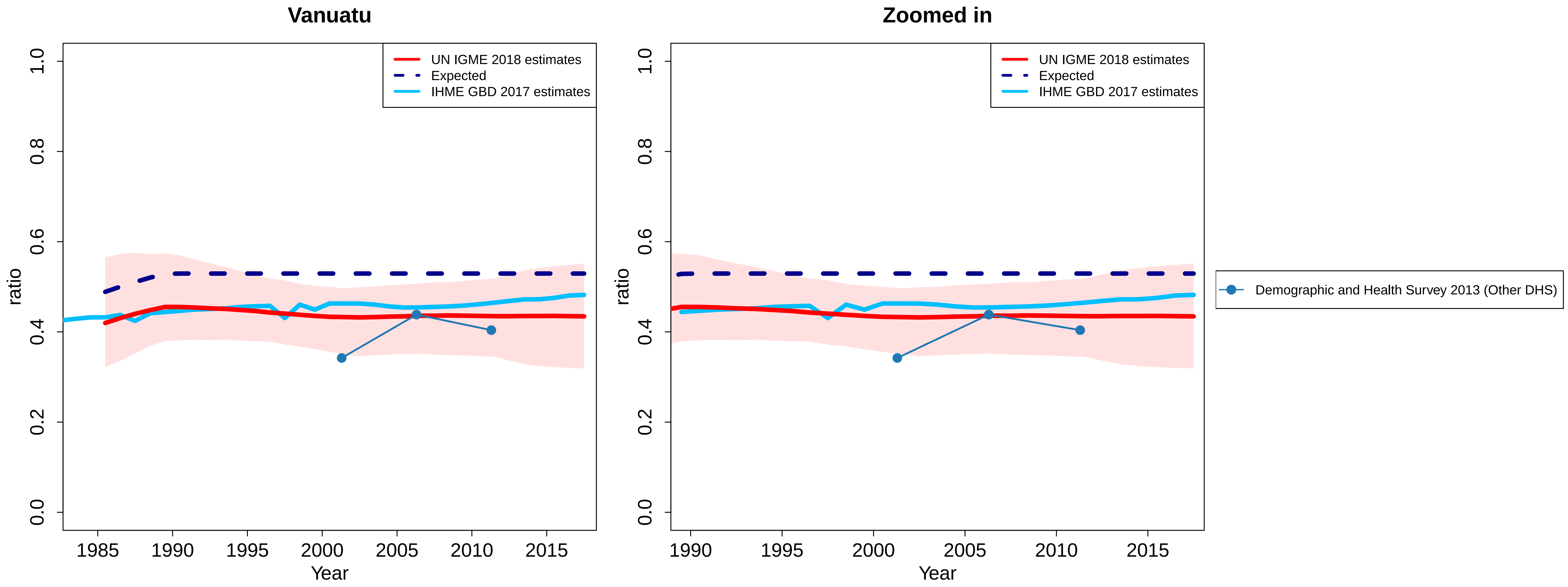
Vanuatu
Zoomed in
UN IGME 2018 estimates
Expected
IHME GBD 2017 estimates
ratio
Year
0.0
0.2
0.4
0.6
0.8
1.0
1985
1990
1995
2000
2005
2010
2015
Demographic and Health Survey 2013 (Other DHS)

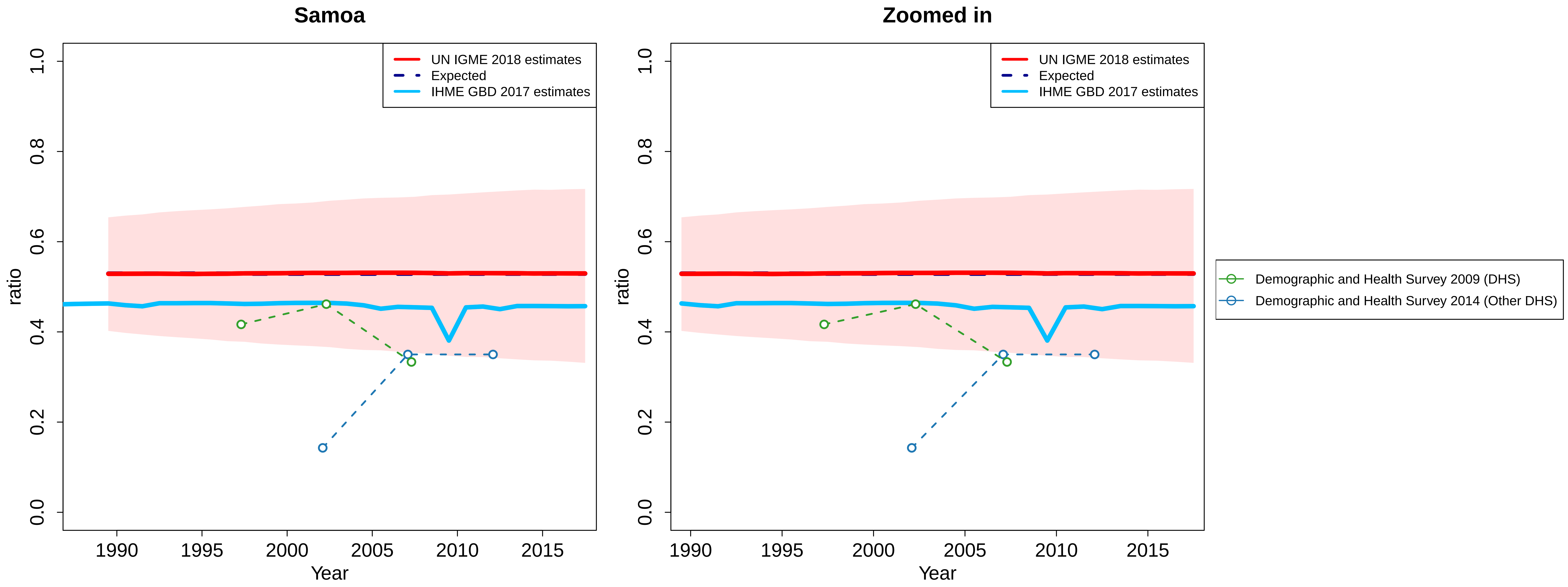
Samoa
Zoomed in
UN IGME 2018 estimates
Expected
IHME GBD 2017 estimates
ratio
0.0
0.2
0.4
0.6
0.8
1.0
1990
1995
2000
2005
2010
2015
Year
Demographic and Health Survey 2009 (DHS)
Demographic and Health Survey 2014 (Other DHS)

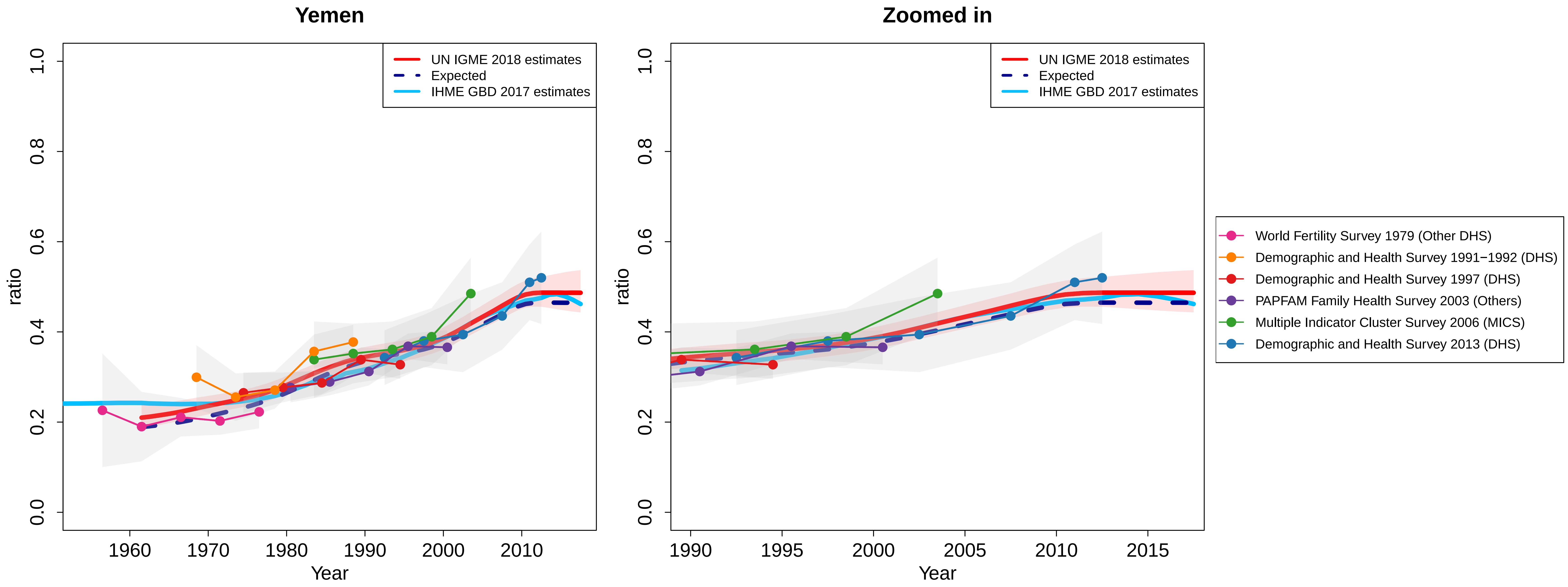

Yemen
Zoomed in
UN IGME 2018 estimates
Expected
IHME GBD 2017 estimates
ratio
Year
World Fertility Survey 1979 (Other DHS)
Demographic and Health Survey 1991−1992 (DHS)
Demographic and Health Survey 1997 (DHS)
PAPFAM Family Health Survey 2003 (Others)
Multiple Indicator Cluster Survey 2006 (MICS)
Demographic and Health Survey 2013 (DHS)

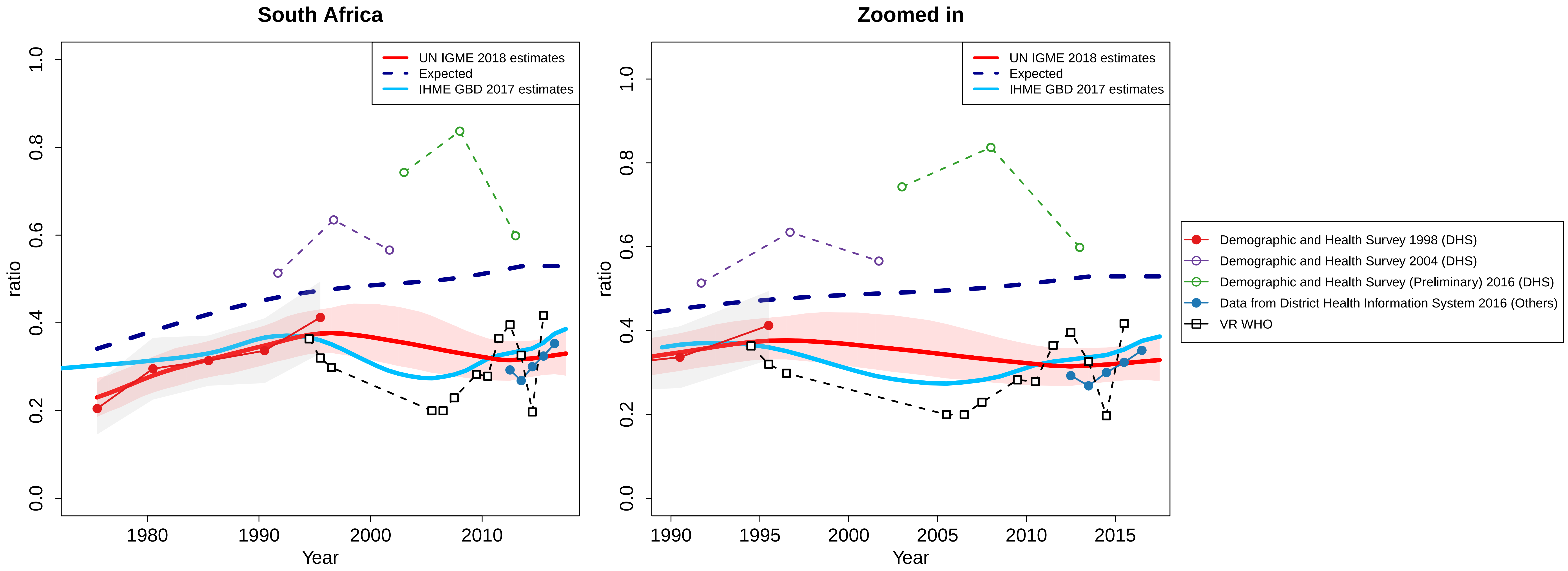
South Africa
Zoomed in
UN IGME 2018 estimates
Expected
IHME GBD 2017 estimates
ratio
Year
0.0
0.2
0.4
0.6
0.8
1.0
1980
1990
2000
2010
1995
2005
2015
Demographic and Health Survey 1998 (DHS)
Demographic and Health Survey 2004 (DHS)
Demographic and Health Survey (Preliminary) 2016 (DHS)
Data from District Health Information System 2016 (Others)
VR WHO

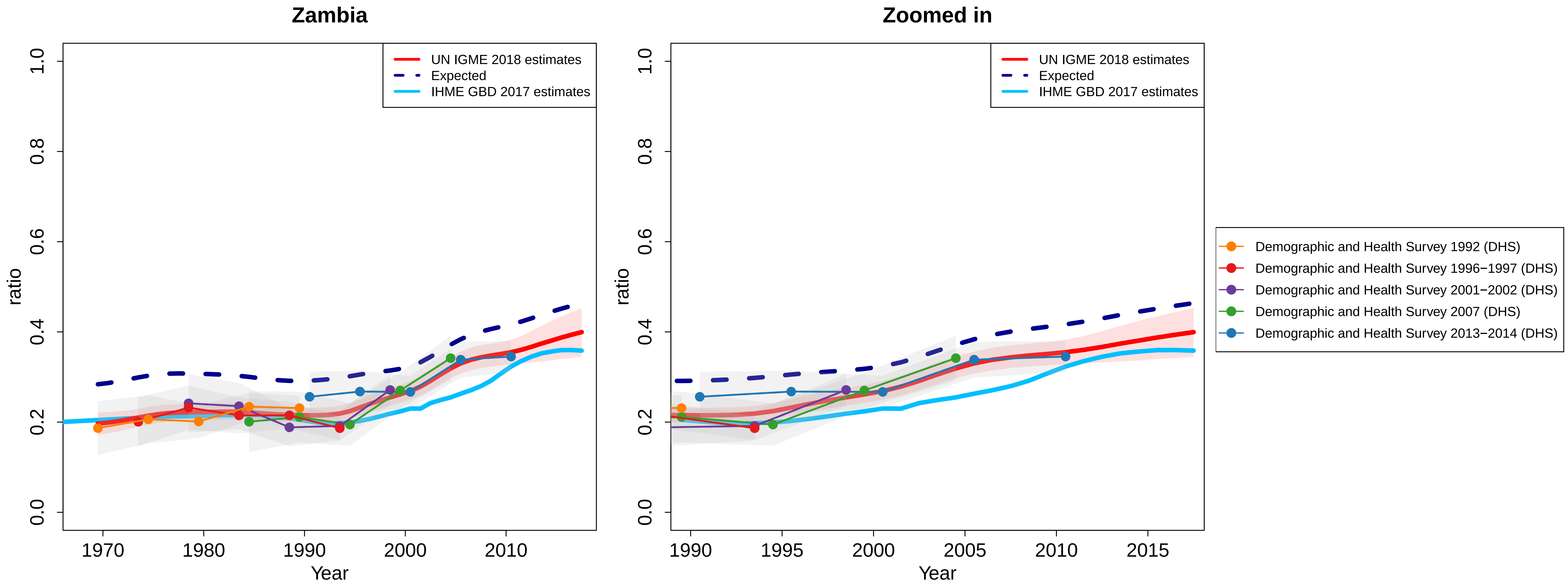
Zambia
Zoomed in
UN IGME 2018 estimates
Expected
IHME GBD 2017 estimates
ratio
Year
0.0
0.2
0.4
0.6
0.8
1.0
1970
1980
1990
2000
2010
1995
2005
2015
Demographic and Health Survey 1992 (DHS)
Demographic and Health Survey 1996−1997 (DHS)
Demographic and Health Survey 2001−2002 (DHS)
Demographic and Health Survey 2007 (DHS)
Demographic and Health Survey 2013−2014 (DHS)

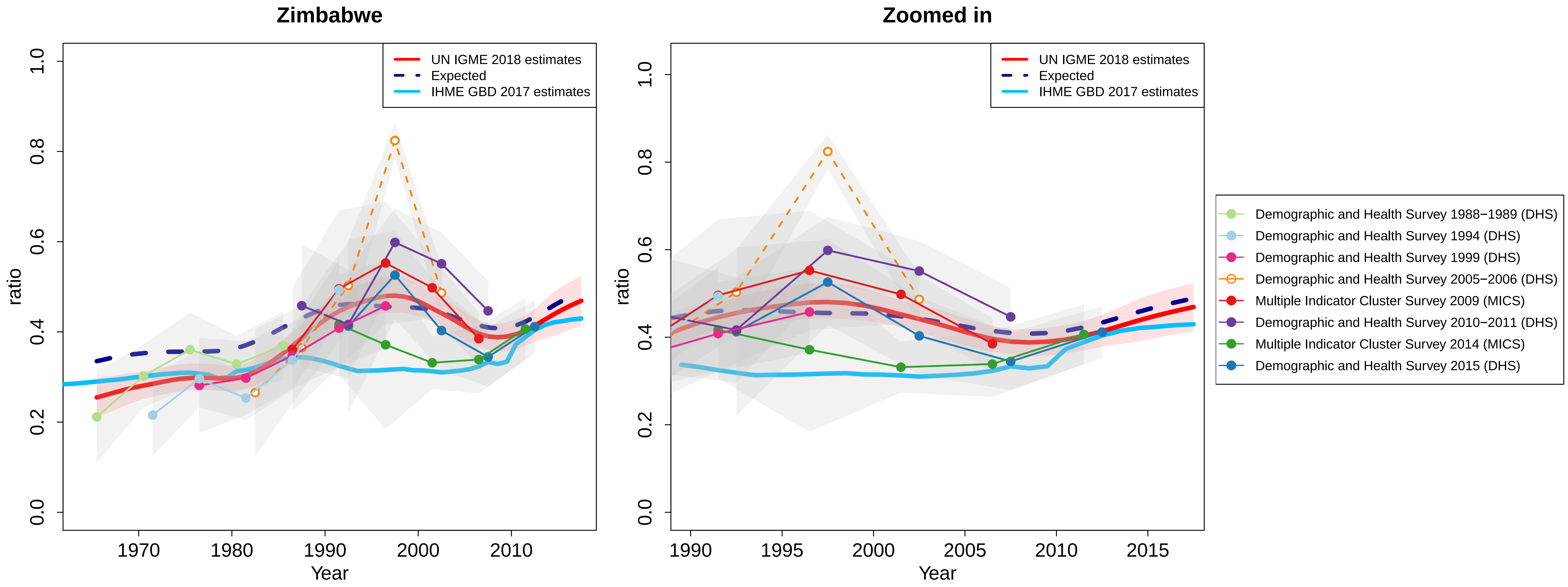
Zimbabwe
Zoomed in
ratio
Year
1970
1980
1990
2000
2010
1995
2005
2015
0.0
0.2
0.4
0.6
0.8
1.0
UN IGME 2018 estimates
Expected
IHME GBD 2017 estimates
Demographic and Health Survey 1988−1989 (DHS)
Demographic and Health Survey 1994 (DHS)
Demographic and Health Survey 1999 (DHS)
Demographic and Health Survey 2005−2006 (DHS)
Multiple Indicator Cluster Survey 2009 (MICS)
Demographic and Health Survey 2010−2011 (DHS)
Multiple Indicator Cluster Survey 2014 (MICS)
Demographic and Health Survey 2015 (DHS)
